# Clonal evolution after treatment pressure in multiple myeloma: heterogenous genomic aberrations and transcriptomic convergence

**DOI:** 10.1038/s41375-022-01597-y

**Published:** 2022-05-28

**Authors:** Kristine Misund, Davine Hofste op Bruinink, Eivind Coward, Remco M. Hoogenboezem, Even Holth Rustad, Mathijs A. Sanders, Morten Rye, Anne-Marit Sponaas, Bronno van der Holt, Sonja Zweegman, Eivind Hovig, Leonardo A. Meza-Zepeda, Anders Sundan, Ola Myklebost, Pieter Sonneveld, Anders Waage

**Affiliations:** 1 Department of Clinical and Molecular Medicine, Norwegian University of Science and Technology, Trondheim, Norway; 2 Department of Hematology, St.Olavs Hospital, Trondheim, Norway; 3 Department of Hematology, Erasmus MC Cancer Institute, Rotterdam, the Netherlands; 4 Bioinformatic Core Facility (BioCore), Norwegian University of Science and Technology, Trondheim, Norway; 5 K.G. Jebsen Center for Genetic Epidemiology, Norwegian University of Science and Technology, Trondheim, Norway; 6 Institute for Cancer Research, Oslo University Hospital, Oslo, Norway; 7 Clinic of Laboratory Medicine, St.Olavs Hospital, Trondheim, Norway; 8 Clinic of Surgery, St.Olavs Hospital, Trondheim, Norway; 9 HOVON Data Center, Department of Hematology, Erasmus MC Cancer Institute, Rotterdam, the Netherlands; 10 Department of Hematology, Amsterdam UMC, the Netherlands; 11 Department of Tumour Biology, Institute for Cancer Research, Oslo University Hospital, Oslo, Norway; 12 Centre for Bioinformatics, Department of Informatics, University of Oslo, Oslo, Norway; 13 Genomics Core Facility, Department of Core Facilities, Oslo University Hospital, Oslo, Norway; 14 Department of Clinical Science, University of Bergen, Bergen, Norway

**Keywords:** Myeloma, Translational research, Cancer genomics, Cancer metabolism, Tumour immunology

## Abstract

We investigated genomic and transcriptomic changes in paired tumor samples of 29 in-house multiple myeloma (MM) patients and 28 patients from the MMRF CoMMpass study before and after treatment. A change in clonal composition was found in 46/57 (82%) of patients, and single-nucleotide variants (SNVs) increased from median 67 to 86. The highest increase in prevalence of genetic aberrations was found in RAS genes (60% to 72%), amp1q21 (18% to 35%), and *TP53* (9% to 18%). The SBS-MM1 mutation signature was detected both in patients receiving high and low dose melphalan. A total of 2589 genes were differentially expressed between early and late samples (FDR < 0.05). Gene set enrichment analysis (GSEA) showed increased expression of E2F, MYC, and glycolysis pathways and a decreased expression in TNF-NFkB and TGFbeta pathways in late compared to early stage. Single sample GSEA (ssGSEA) scores of differentially expressed pathways revealed that these changes were most evident in end-stage disease. Increased expression of several potentially targetable genes was found at late disease stages, including cancer-testis antigens, *XPO1* and ABC transporters. Our study demonstrates a transcriptomic convergence of pathways supporting increased proliferation and metabolism during disease progression in MM.

## Introduction

Multiple myeloma (MM) is a hematological malignancy, which is characterized by uncontrolled proliferation of plasma cells. Novel treatments introduced the last two decades have improved overall survival (OS) of MM patients. Nevertheless, MM remains an incurable disease that in advanced stages is characterized by an aggressive phenotype and treatment resistance.

At the genetic level, MM is characterized by a limited number of driver mutations in genes including *NRAS* and *KRAS* and recurrent copy number aberrations (CNAs) including hyperdiploidy, deletion of chromosome 17p (del17p) and gain of chromosome 1q (gain1q), as well as primary translocations involving the immunoglobulin heavy locus (IgH) [1–5]. Still, the MM genome is also characterized by a high degree of inter- and intra-patient variability, which raises the possibility that diverse changes at the DNA level may lead to a common phenotype at the transcriptional level.

Disease progression from newly diagnosed MM to progressive disease (PD) is driven by selection of the fittest clones in the context of treatment pressure. At the DNA level, this can be observed as differential, linear or stable evolution and is associated with depth of response [1, 6–8]. Even though specific mutations in drug targets are rarely identified, biallelic events in tumor suppressor genes and gain1q have shown to be enriched in PD samples [9–11], and alkylating drugs like melphalan can have direct mutagenic effect [12].

Array-based gene expression profiling, and recently also RNA sequencing (RNA-seq), have so far provided useful information for classifying of MM patients according to prognostic subtypes [13]. Comprehensive studies have described transcriptomic changes related to specific mutations. Biallelic deletions of tumor suppressors and chr(1q) amplifications (amp1q) have shown the greatest impact on gene expression, deregulating pathways related to cell-cycle, proliferation and expression of immunotherapy targets [14]. Yet, it remains largely unknown how disease progression under treatment pressure is characterized at the transcriptomic level, and if there is any association with clonal evolution at the genomic level. Since MM eventually will progress in most patients, it is of great interest to identify specific tumor vulnerabilities at the time of PD [15, 16].

In this study, whole exome sequencing (WES) and RNA-seq were performed on paired early and late tumor samples of 57 MM patients to identify genomic and transcriptomic changes during disease progression.

## Methods

### Patients and samples

Two patient cohorts were used in this study. The in-house cohort consisted of sequential tumor samples (*n* = 69) from 29 MM patients (Table S1A) from the MM biobank at St. Olavs Hospital, Norway (Biobank1) (*n* = 18) and Erasmus MC Cancer Institute, the Netherlands. A subgroup of patients had been enrolled in the EMN02/HO95 trial [17] (*n* = 9) or HOVON-87/NMSG18 trial [18] (*n* = 2). For 23 patients, the first sample (early sample) was taken before start of first treatment (diagnosis), and for 6 patients at the time of PD. Nine patients had 3–4 samples. CD138-positive tumor cells were isolated using RoboSep (StemCell Technologies, Grenoble, France). Tumor purity was subsequently determined morphologically or by flow cytometry. Only samples with a tumor purity ≥80% were included in the study (range 80–100, median 95%) (Table S2). WES was performed on all samples (*n* = 69), and RNA-seq on 52 samples from 21 patients (Table S1A). The study was approved by Regional Committee for Medical and Health Research Ethics (2012/1915, 2017/2043).

The second cohort comprised 28 patients from the CoMMpass study [19], release IA13. First sample was taken at diagnosis and the second at PD (Table S1B). Two patients had 3–4 samples. Estimated tumor purity was >75% by CNV analysis. FASTQ files were downloaded from dbGap (Study Accession: phs000748), while processed data were downloaded from (http://research.themmrf.org).

All patients had received a proteasome inhibitor and/or immunomodulatory drug (IMiD) at least once between sampling. Clinical data for both cohorts are summarized in Table 1, S3A, S3B.

**Table 1:** Clinical characteristics of patients.

	In-house cohort	CoMMpass cohort
	*n* = 29	*n* = 28
Age at first treatment Median (range)	62 (42–84) years	68 (39–82) years
Male	52%	50%
Ig class	Patients	Patients
IgG	20	11
IgA	7	4
IgD	1	0
Light chain disease	1	3
Unknown	0	5
Missing	0	5
Kappa	20	15
Lambda	9	8
missing	0	5
ISS		
1	4	8
2	14	6
3	9	14
missing	2	0
R-ISS		
1	3	2
2	14	17
3	6	3
missing	6	6
FISH /FISH-seq^#^	Detected (# tested)	Detected (# tested)
t(11;14)	4 (12)	6 (27)
t(4;14)	5 (28)	6 (27)
t(14;16)	0 (11)	0 (27)
t(14;20)	0 (0)	1 (27)
Other primary transl.	0 (0)	3 (27)
del17p	4 (28)	1 (27)
Respons 1. Treatment	Patients	Patients
CR	4	5
VGPR	12	15
PR	9	6
SD	3	0
PD	0	2
PFS Median (range)	14 (2–113) months	17 (2–42) months
OS Median (range)	40 (8–245) months	48 (13–72) months
Interval Sample 1 and 2 Median (range)	16 (5–70) months	21 (3–45) months
Sample type		
Diagnosis-1.PD	17 patients	15 patients
Diagnosis-later PD	6	13
PD-PD	6*	

^*^In addition, 8 patients from in-house cohort and 2 patients from CoMMpass dataset had more than 2 samples.

^#^CoMMpass dataset: Primary translocations detected by long-insert WGS, del17p by CN data.

### WES

WES of DNA from bone marrow myeloma cells and matched germline controls has previously been described [20].

### Detection and filtering of somatic variants

Somatic variant detection was performed with Strelka2 (v2.9.10).

### Copy number variant (CNV) estimation

Allele-specific CNV estimation was performed with Sequenza [21], and Facets_cnv (v0.14.0) [22].

### Clonal evolution analysis

Subclonal reconstruction was performed in R with DPClust (v2.2.8) using default settings. Filtered Strelka2 variants and the allele specific CNV calls from Facets were used as input.

### Mutational signature analysis

Mutational signature fitting was performed with the *mmsig* package in R as previously described [23, 24].

### RNA-seq library construction and sequencing

RNA-seq libraries were prepared with the TruSeq Stranded mRNA kit (Illumina, San Diego, CA, USA) according to the manufacturer’s instructions, and run on NextSeq500 or HiSeq4000 (Illumina).

### Differential expression analysis, GSEA and ssGSEA

A DESeq2 (v1.28.1) paired analysis was performed in R Studio to identify differentially expressed genes. GSEA (v4.1.0; Broad Institute), using hallmark gene sets, was performed to identify enriched pathways. Proliferation index (PI) was calculated according to Zhan et al. [25] based on 11 genes that previously have been shown to be involved in proliferation. A PI change was defined as an increase if >0.4 and decrease if <−0.4, and high increase if >1.

ssGSEA was performed by sorting the genes in each sample in descending order according to their expression level, for each of the GSEA hallmark gene sets (core enriched genes). A ssGSEA score was calculated using a previously published algorithm [26]. To emphasize relative differences in ssGSEA scores between samples, expression levels (TPM) were centered and normalized across samples before sorting.

### Statistical analysis

Survival analysis was performed in GraphPad Prism9. *p*-values were determined with the log-rank (Mantel–Cox) test and hazard ratios (HR) with the Mantel-Haenszel method. Cox regression analysis was performed in SPSS (v27). Groupwise correlations were performed in Prism9 using the Spearman correlation test. We used Prism9 and R Studio (v3.6.3) for group comparisons, Wilcoxon rank-sum analysis for paired analysis, Mann-Whitney test for unpaired analysis and Kruskal-Wallis test for comparisons of groups. *p*-values <0.05 were considered significant.

Additional detailed information on all used methods is provided in Supplementary Methods.

## Results

### Increase in mutation load after treatment

**Fig. 1:**
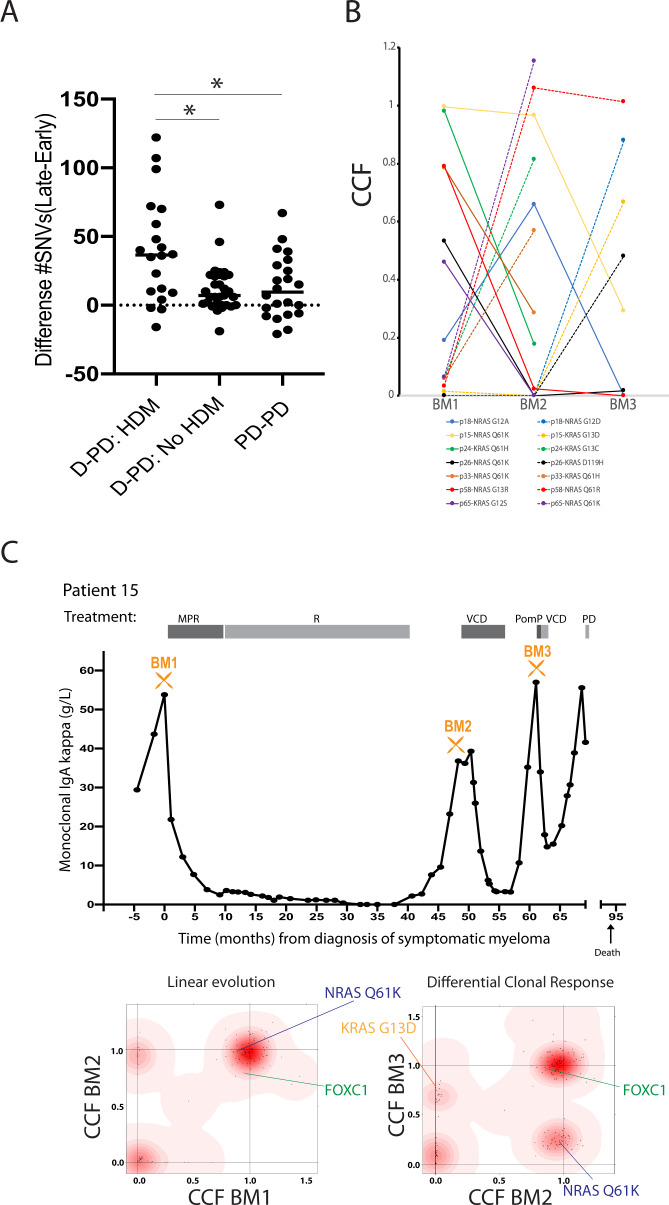
Increased mutation load and clonal evolution during the myeloma disease course. **A** Increase in number of mutations in samples taken at PD. There was a particular increase in patients that had received HDM (*p < 0.05, Kruskal–Wallis test). D diagnosis (start of treatment), PD: progressive disease, HDM: high-dose melphalan. For Diagnosis-PD pairs the first available PD sample was used in the analysis. **B** Alternating dominance in myeloma clones harboring RAS mutations. In all 7 patients with decreased clonal fraction of mutated *KRAS*/*NRAS* genes, another *NRAS*/*KRAS* mutation appeared. **C** Overview of treatment and M component in patient 15. Below is shown the estimated clonal composition from the DPclust analysis. The patient had a RAS shift. Also see Table S5 and Fig S3. CCF Cancer Cell Fraction, M Melphalan, R Revlimid, V Velcade, C Cyclophosphamide, Pom Pomalidomide, P Prednisolon, D Dexamethasone.

WES analysis showed an increased number of exonic SNVs between early and late stage disease samples from a median of 67 [range 29–204] to 86 [26–252] (nonsynonymous: 45 to 59) (both *p* < 0.01). The fold change (FC) in mutations was highly variable between patients (range 0.80–2.81), and a significant increase was seen when comparing both diagnosis-PD and PD-PD samples (Fig. S1A–D). The most prominent increase of mutations was found in patients receiving treatment with high-dose melphalan (HDM) (median 36 versus 7, *p* < 0.05, Fig. 1A) [27].

### Genomic alterations and clonal changes during disease progression

Ninety-eight percent (56/57) of the patients had a mutation in at least one of the 80 previously identified MM driver genes [4, 5] (Table S4) at one or more timepoints during the disease course. RAS gene mutations were the most frequent driver in our cohort [1–3, 28], and 74% of patients had a *KRAS* or *NRAS* mutation in at least one timepoint (Fig. S2A).

In 7 patients with a reduction or disappearance of a dominating clone with a RAS mutation, this was replaced by another clone with a different RAS mutation. Thus, the major clone still harbored a RAS mutation (Fig. 1B), indicating a benefit for the tumor to keep a consistently activated RAS pathway during disease progression.

Altogether, the most frequent events acquired or enriched for at PD were *KRAS* mutations (39% late versus 28% early), *NRAS* mutations (39% versus 33%), amp1q21 (35% late versus 18% early), and *TP53* mutations or deletions (18% late versus 9% early). Half of the patients with an amp1q21 at the later stage, had a gain1q21 at the earlier stage. Hyperdiploid (HRD) tumors were most likely to acquire RAS mutations at PD, accounting for 80% of acquired/enriched *NRAS*/*KRAS* SNVs (Table S5).

Seventy-five percent of patients in our cohort became refractory to immunomodulatory drugs (IMiDs) and/or proteasome inhibitors. However, few mutations were found in the IMiD pathway genes and proteasome subunits [29–31] (Tables S5, S6, Fig. S2A, B), and there was no obvious pattern associated with treatment.

Twenty-eight percent of all mutations in the in-house cohort were found to be expressed (Fig. S1E), and this increased to 66% when considering mutations in driver genes only. There was a slight increase in the fraction of expressed mutations at late stage compared to early (29% versus 22.5%, *p* < 0.001; Fisher’s exact test).

We further studied changes in clonal substructure during the myeloma disease course by DPclust. Fifty-eight percent (33/57), had a shift in clonal dominance (differential clonal response) from early to a later stage, 23% (13/57) acquired novel genomic aberrations (linear evolution) and 19% (11/57) had no/minor genomic changes (stable evolution) (Table S5) [8]. Evidently, the clonal patterns may change during the disease course as observed in patients with 3 or more samples (Table S5, Figs. 1C, and S3). Tumors changing their clonal dominance tended to select for a clone with more high-risk features later in the disease course, as illustrated by the loss of Chr17p or acquisition of a *TP53* mutation in 5 patients (including one bi-allelic TP53 event) [10].

### Mutational signature related to melphalan and depth of response

**Fig. 2:**
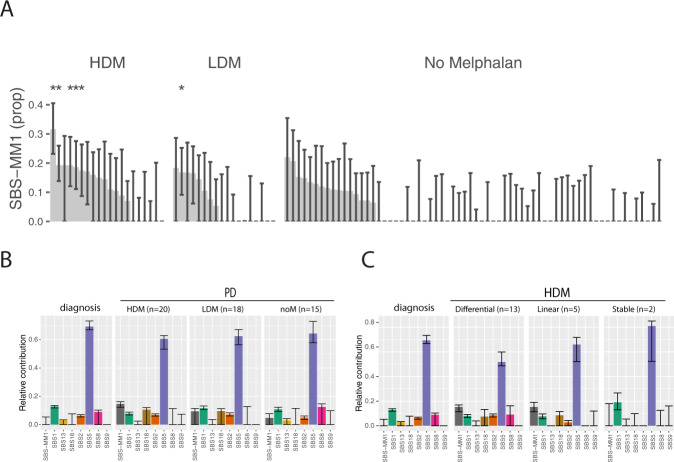
Mutational signatures after treatment with Melphalan. **A** Estimated SBS-MM1 contribution with 95% CI for individual patients. Only patients exposed to melphalan showed evidence of significant SBS-MM1 contribution. *statistically significant transcriptional strand bias in the typical pattern for SBS-MM1: C > T mutations in the trinucleotide contexts CCA, GCA, GCC, GCG, and GCT. **B** Mutation signature patterns at diagnosis versus PD divided into HDM (high dose melphalan), LDM (low dose melphalan) or noM (no melphalan) groups. **C** Mutational signature analysis of diagnosis samples and samples receiving HDM based on their evolutional pattern.

Analyses of mutational signatures have demonstrated a single base substitution signature that is associated with the use of HDM (SBS-MM1) [23, 24]. Performing mutational signature analysis using the *mmsig* algorithm, we found strong evidence of presence of SBS-MM1 (95% CI > 0) in eight patients; six received HDM and two had been treated with regimens containing low dose melphalan (LDM) (Figs. 2A and S4A, B). Six of these patients also had statistically significant transcriptional strand bias in the pattern typically associated with SBS-MM1 (five HDM patients). Group analysis according to melphalan exposure confirmed the presence of SBS-MM1 in the HDM group (*n* = 20), but not in the LDM (*n* = 18) or no melphalan (*n* = 15) groups (Fig. 2B). All of the eight individual patients with strong evidence of SBS-MM1 had either a differential clonal response or linear evolution and all but one achieved a VGPR or CR following melphalan therapy, however, the associations were not statistically significant (Figs. 2C and S4C). The results demonstrate that SBS-MM1 can be found after both HDM and LDM, and indicate an association with deep responses and shifts in clonal structure.

### Increased expression of cell cycle and proliferative genes at progression

**Fig. 3:**
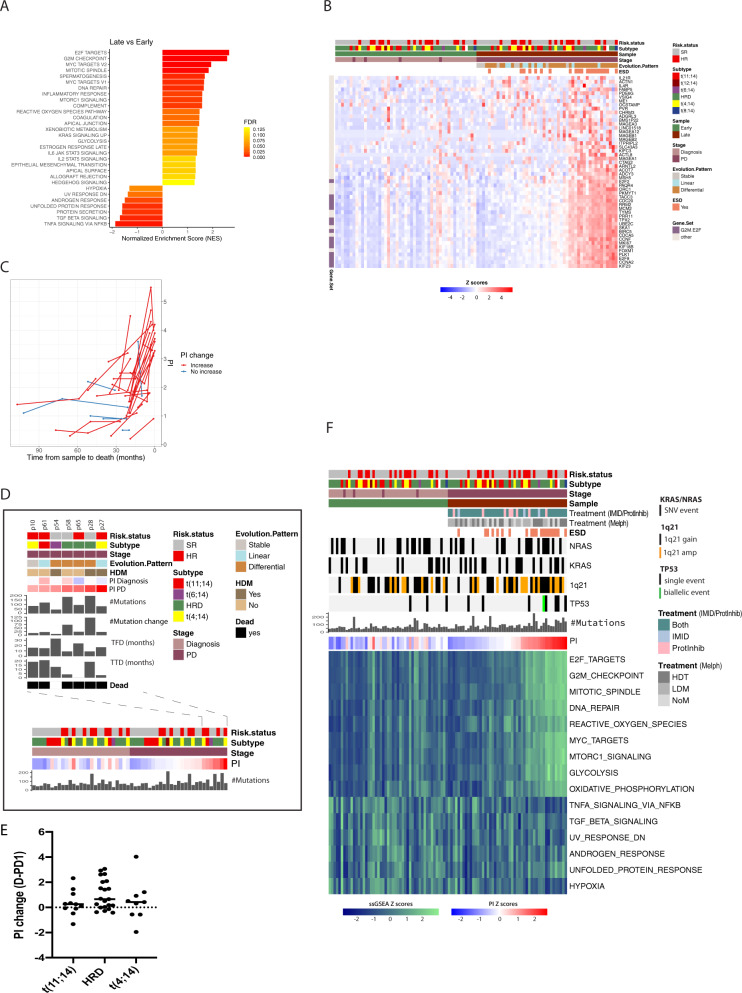
Increased expression of genes involved in proliferative and metabolic pathways at progression. **A** GSEA analysis using hallmark gene sets showed that 23 pathways were upregulated and 7 downregulated (FDR *q*-value<0.2, NES > 1.3) at late stage (latest available PD sample) versus early (1^st^ sample). The top upregulated pathways were E2F targets and G2M checkpoint involved in cell division and cell cycle. FDR: False discovery rate. **B** Heatmap showing the top 50 upregulated genes and their expression levels (log2(TPM + 1), z scored) in early and late samples. **C** The figure shows the evolvement in proliferative index (PI) between early and late samples in deceased patients. There was significant increase in PI in end-stage disease. **D** The figure shows diagnosis-1^st^ PD paired samples, and shows that patients with high PI at 1^st^ PD have different cytogenetic backgrounds. Mutation load and increase from diagnosis, time from diagnosis (TFD), and time to death/last control (TTD) are shown for patients with high PI at 1^st^ PD. **E** There was no significant difference in PI increase in diagnosis-first available PD sample between cytogenetic subgroups. This did not change when dividing the HRD group into cyclinD1 or cyclinD2 expressers (Fig. S6). CCND2, CCND3 and MAFs were not included due to few samples. **F** Heatmap for selected pathways showing ssGSEA for early-late pairs. Information on mutation load and presence of the most frequently enriched/acquired genomic aberrations at PD (*KRAS*, *NRAS*, amp1q21, and *TP53*) as well as intervening treatment received between the two BM sample timepoints are shown. In heatmaps patients are sorted horizontally by increasing PI of the late samples (right half) and the same patient order is used for the early samples (left half). SR standard risk according to ISS/R-ISS, HR High risk, ESD End-stage disease (<12 months from death). Cytogenetic subgroups; t(11;14)/CCND1, t(12;14)/ CCND2, t(6;14)/CCND3, t(4;14)/WHSC1(NSD2/MMSET), t(8;14)/MAFA are shown (Table S12). IMID Immunomodulatory drug, ProtInhib Proteasome inhibitor.

We went on to investigate whether the corresponding transcriptomic changes led to changes in specific biological processes. We started with a paired groupwise comparison between the transcriptomes of the latest available PD sample versus the early-stage samples (*n* = 49 patients). We found 2589 genes to be differentially expressed between these timepoints, 1562 by more than 1.5 FC (FDR < 0.05; Table S7). 1642 genes (1050, FC > 1.5) were upregulated and 947 (512, FC < 0.66) downregulated at the later disease stage. GSEA of the hallmark pathways [32] identified a significant enrichment of genes involved in cell division and growth-related pathways (G2M, E2F targets), as well as DNA repair and cell metabolism (MYC targets, glycolysis) in late stage disease (Fig. 3A). TNFα signaling via NFκΒ (TNF-NFκB) and TGFβ signaling were the most significantly downregulated pathways. A heatmap of the 50 most upregulated genes later in the disease course are shown in Fig. 3B.

### Increased proliferative status in end-stage disease

To further characterize cell division and proliferation, we calculated the PI [25] per tumor sample. We analyzed changes over time at both the individual tumor level and between disease stages. PI was significantly increased at later stages (*p* < 0.0001; Table S8). Fifty-seven percent (28/49) had increased PI at a later stage, 29% (14/49) had no change, and 14% (7/49) had decrease. In patients with end-stage disease (time to death <12 months) 80% patients (16/20) had increased PI (Fig. 3C). We further addressed whether PI at diagnosis was associated with clinical outcomes. For this purpose, we analyzed 767 patients in the CoMMpass dataset (IA13) of newly diagnosed MM patients. Indeed, patients with PI > 75^th^ percentile showed adverse progression free survival (PFS) (HR 1.8, *p* < 0.0001) and OS (HR 2.2/1.3;univariate/multivariate, *p* < 0.0001) (Fig. S5A, B).

### Phenotypic transformation independent of cytogenetic subgroup or initial risk status

To investigate whether certain tumor types were more prone to transform into a proliferative type, paired diagnosis-1^st^ PD samples were analyzed (*n* = 27 patients). We observed that the 7 patients with high PI (all PI > 3) at 1^st^ PD had different cytogenetic backgrounds (Fig. 3D). We did not find significant differences in levels of PI increase between the cytogenetic subgroups (Figs. 3E and S6). Thus the transformation into more proliferative tumors seem to be independent from the canonical IGH-background.

Out of 13 patients progressing within 2 years from diagnosis with a high PI increase, 7 were defined to have standard risk (SR), i.e. ISS stage I/II (Table S8, Fig. S7A). This shows that PI increase can occur irrespective of initial risk status. Five of the 7 patients died within 4 months from the time of this PD. Six were HRD patients (1 uncertain), two had undergone HDM. All had a shift in their dominating clone and mutational increase. There were no common genetic changes; two acquired a *FAM46C* mutation, one a del17p, one acquired and two had enrichment of a mutation in *NRAS Q61* at PD, for 3 of them in combination with either a gain (*n* = 2) or amp (*n* = 1) 1q21, exemplifying the heterogeneity in genetic aberrations associated with PI increase.

### High co-regulation of proliferative and metabolic pathways at late stage

To study changes in differentially expressed pathways over time both at the individual level and between diseases stages, variance in ssGSEA scores was compared between early and late samples. We found an increased expression of genes involved in MYC, glycolysis, oxidative phosphorylation, and MTORC1 pathways at a later stage in the majority of patients within all molecular subgroups (Fig. 3F). Interestingly, 81% of patients with increased glycolysis also had increased expression of genes involved in oxidative phosphorylation. As MYC is known to be regulated by structural rearrangements leading to increased gene expression levels [33, 34], we analyzed whether increased MYC target genes correlated with increased MYC transcription. This was observed in 59% (17/29) of patients with FC > 1.5, of these 27% had FC > 3 (Table S9). Thus, our data suggest that in 40% of patients MYC signaling was activated by other means than increased MYC transcript levels.

Altogether, 89% (34/38) of patients having increased PI at a later stage in the disease course, also had an increased expression of either the MYC, glycolysis, or MTORC1 pathway. For the majority of patients (76%; 29/38) all 3 pathways were increased (Table S9). This suggests a high correlation and co-regulation of proliferative and metabolic pathways in the disease course of MM.

### Decreased expression of genes involved in the TNF-NFκB pathway

ssGSEA showed that most tumors (64%) with an increased expression of proliferative and metabolic pathways had decreased expression of the TNF-NFκB gene set. For a subset of patients, this decrease was accompanied by a decrease in gene sets involved in TGF Beta signaling, unfolded protein response, and hypoxia (Fig. 3F and Table S9). Mutations in the NFκB pathway were found in 12% (6/49) of patients, with identical frequency early and late (10%), and affected CYLD and TRAF3. Activating mutations were found in two patients with decreased TNF-NFκB pathway activity. When measuring the NFκB activity with the NFκB index [35], 25% of the patients kept an active NFκB pathway at PD despite decreased TNF-NFκB, suggesting compensation by activating mutations [34, 35] (Table S9).

We were not able to find treatment-specific differences in the transcriptional patterns, and transcriptional changes were independent of having received HDM or not (Fig. S7B).

### Relation between transcriptomic changes and clonal evolution patterns

**Fig. 4:**
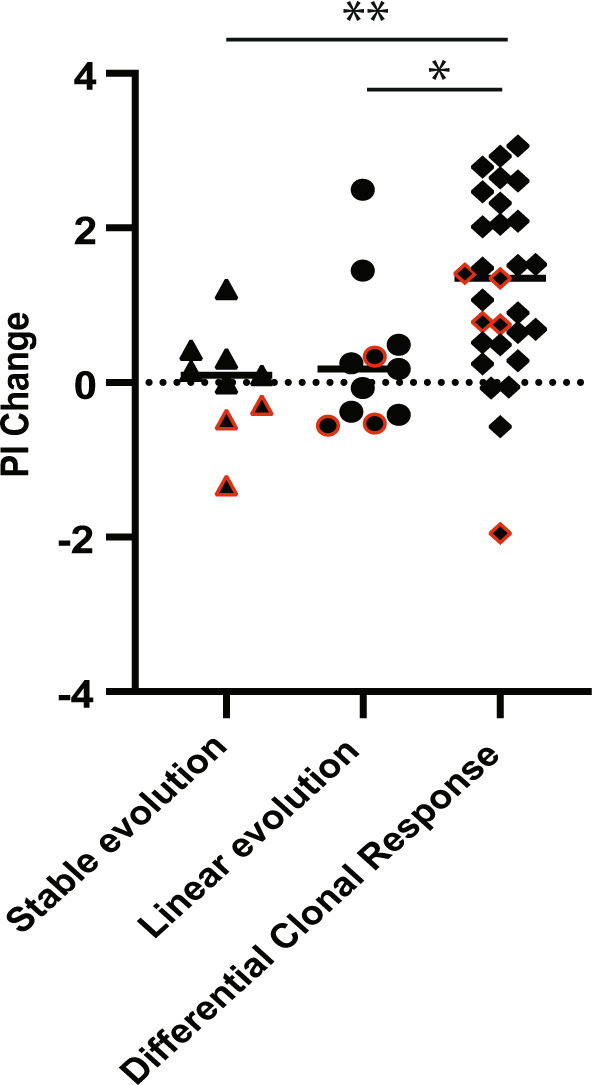
Changes in PI between late and early samples grouped by clonal evolutionary patterns. The figure indicates that most samples that have a change in their PI also have a change in their clonal composition of the malignant cells (*p < 0.05, Kruskal–Wallis test). Each black dot represents a patient, red border indicates top 25% PI in the earliest sample.

The majority of MM tumors with transcriptomic changes also had a change in clonal composition (32/35; 91%) (Figs. 4 and S2C, D and Table S10). This suggests that the increased PI could be a result of either a selection of a more proliferative clone later in the disease course (differential clonal response), or acquisition of new mutations at progression (linear pattern). However, 3 tumor pairs with a change in PI, one with a PI increase, were found to be evolutionary stable (Fig. S8).

### Expression of genes related to transportation of or sensitivity to drugs

**Fig. 5:**
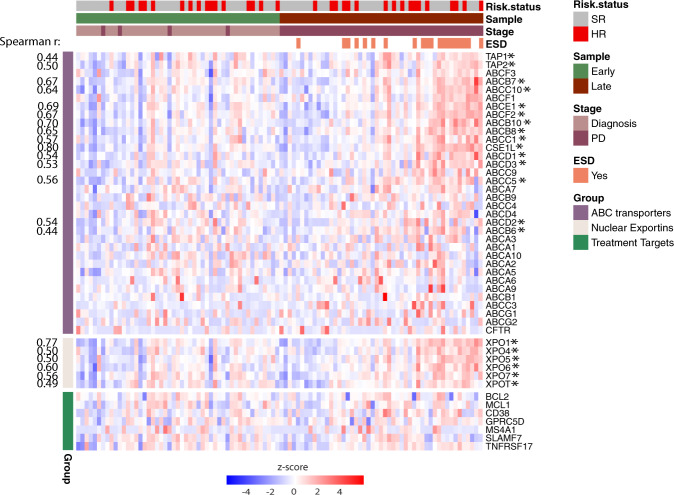
Increased expression of ABC transporters and exportins in PD samples. Heatmap showing expression levels (log2(TPM + 1), z scored) of ABC transporter genes, nuclear exportins, and other treatment relevant targets in early and late samples. Genes with Spearman correlation >0.4 are marked with a * and correlation value shown to the left, all having *p* < 1x10e−5. Patients are sorted horizontally by increasing PI of the late samples (right half) and the same patient order is used for the early samples (left half). SR standard risk according to ISS/R-ISS, HR High risk, ESD End-stage disease (<12 months from death).

We analyzed for expression of clinically relevant treatment targets in PD clones. Exportins are responsible for transportation of a number of substances from nucleus to cytoplasm and are of major interest as drug targets in cancer [36]. XPO1 is particularly relevant as a target for selinexor which is an approved drug for relapsed or refractory MM. Expression of *XPO1* and other exportins were increased later in the disease course, and were positively correlated to PI (Spearman r > 0.4, *p* < 0.00001) (Fig. 5). The ATP-binding cassette (ABC) transporters transport a variety of substrates across the membrane, many being involved in multidrug resistance. We observed increased expression of a range of ABC transporters at PD, especially prominent in samples with high PI (Fig. 5). However, *ABCB1* which has been related to proteasome inhibitor resistance in MM cell lines [37] and in extra-medullary myeloma [38], had low expression and was not upregulated at later disease stages.

For other relevant targets, *BCL2* (venetoclax), *CD38* (daratumumab, isatuximab), and *SLAMF7* (elotuzumab), we found no particular trend in expression in different disease stages (Fig. 5). Mutations in the relevant drug target genes were rare (Table S11).

*CRBN* expression levels have occasionally been linked to IMiD sensitivity [39, 40]. Gene expression levels of *CRBN* were not changed from diagnosis to IMiD resistant PD (*n* = 23). However, looking specifically into the exon 10 splicing transcript [31], and its ratio to the longest transcript variant, we found this significantly increased at the time of IMiD resistance (*p* = 0.04; Fig S9), indicating that this could be a relevant mechanism of resistance for some patients. For patients developing proteasome inhibitor resistance (*n* = 19), eight patients (42%) had increased transcript levels of proteasome B (*PSMB*) genes 5, 6, and 7, but the change was not statistically significant.

### Cancer germline antigens and immunotherapy targets

**Fig. 6:**
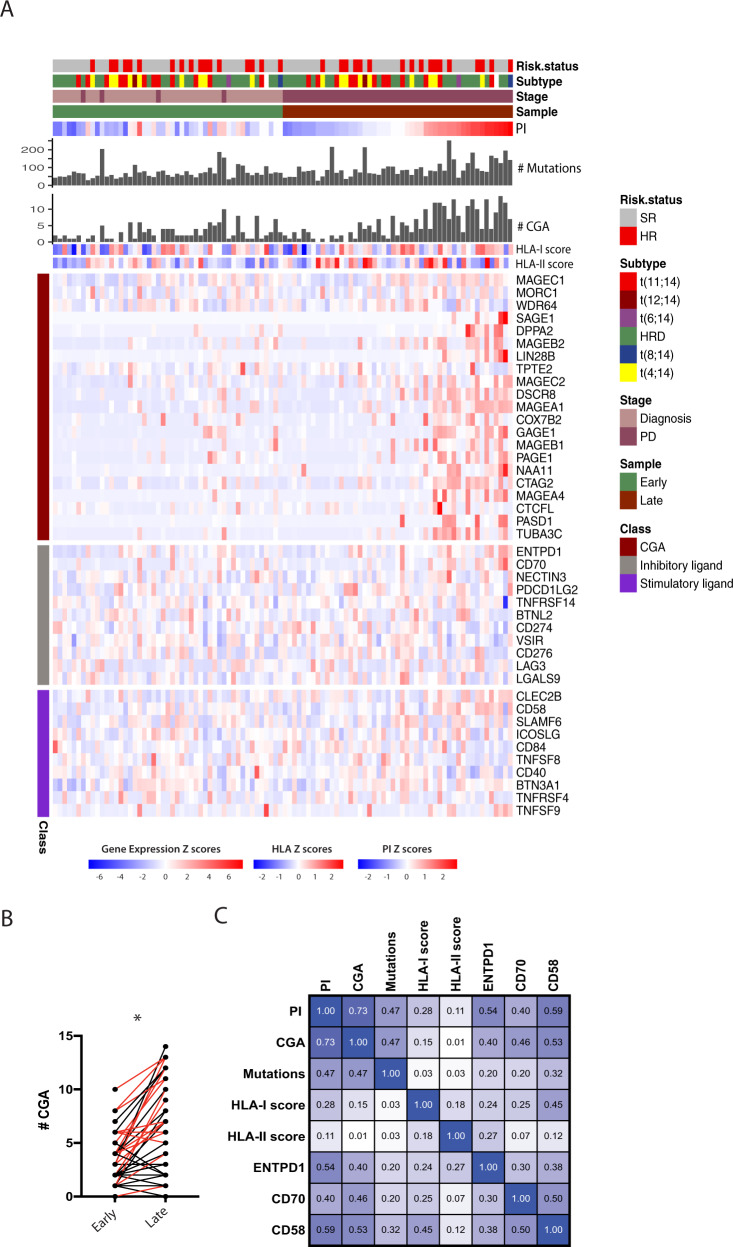
Cancer Germline Antigens and a few immune regulators increased during disease progression. **A** Heatmap showing the number and expression levels of CGAs in early and late samples, HLA scores, and selected inhibitory and stimulatory ligands/receptors. CGAs with expression >10 TPM in >1 sample were included in the heatmap. Patients are sorted horizontally by increasing PI of the late samples (right half) and the same patient order is used for the early samples (left half). **B** Number of expressed CGAs (TPM > 2) in paired early-late (latest available PD) samples increases significantly in the late samples (*p* < 0.0001, Wilcoxon rank test). Red color: paired samples including end-stage disease sample. See also Fig S10. **C** Correlation plot of PI, CGA, mutation load, and HLA scores. Inhibitory and stimulatory receptors from **A**, with correlation above 0.45 (Spearman r) to either PI or CGA, are included. All correlations >0.45 have *p*-value <1x10e−5. SR: standard risk according to ISS/R-ISS, HR: High risk.

We observed that 14% of the top 50 increased genes at progression were defined as cancer-testis antigens, including *MAGEB1* and *CTAG2* (Fig. 3B). We, therefore, analyzed whether expression of this group of genes was generally increased at later disease stages. To this end, we took advantage of a well-defined set of 27 cancer-testis antigens defined in a recent study as hematological Cancer Germline Antigens (CGAs) [41]. We found that 43% (22/49) had an increased expression (defined as an increase of #CGA≥3) at a later disease stage (*p* < 0.0001) and 65% in end-stage disease (Figs. 6A, B, and S10). Increased expression of CGAs correlated with elevated PI and mutation load (Spearman *r* = 0.73 and *r* = 0.47, respectively; Fig. 6C). CGA expression was also positively associated with gene expression signatures reflecting cell-cycle activity, MYC targets, and metabolic pathways, whereas the TNF-NFκB gene set was downregulated in CGA-high MM.

As CGA epitopes can be presented by MHC and recognized by T cells, we estimated expression of MHC I and II genes. We did not find any correlation between CGA expression and HLA-II score [41] (*r* = 0.01) or HLA-I score (*r* = 0.15) (Fig. 6A, C). Somatic mutations in immune checkpoints (1 patient) or CGAs (3 patients) were uncommon (Table S11).

We investigated whether T cell stimulatory ligands were downregulated or inhibitory ligands upregulated on the late-stage tumors. However, there were no obvious changes in expression of checkpoint molecules (Fig. 6A) [41, 42]. We found *CD58* (LFA-3), an adhesion molecule that binds CD2 on T cells [43] and *ENTPD1* to be positively correlated (*r* = 0.59 and 0.54, respectively) with increased PI (Fig. 6A, C). *ENTPD1* codes for CD39, an exoenzyme involved in generation of immunosuppressive adenosine. This may be an alternative immune evasion mechanism in a subset of patients [44].

## Discussion

In this study we find that changes at the transcriptomic level during disease progression show a uniform increase into a more proliferative phenotype as well as increased expression of genes that enable the tumor cells to tolerate increased cell growth and energy demand. This included metabolic pathways such as glycolysis, oxidative phosphorylation, MTORC1, and MYC signaling. Our data showed that the phenotypic transformation is more pronounced at end-stage disease. Increased PI was found to be a marker of end-stage/aggressive disease. Importantly, we identified increased expression of treatment targets that can be clinically relevant.

Increased expression of proliferative and metabolic pathways in extramedullary myeloma [45] and high-risk transcriptional profile during myeloma progression have recently been described [46]. Our data show a similar transformation in bone marrow at disease progression.

Almost all patients showing this phenotypic transformation had a predominant clonal change during their disease course, indicating that a more proliferative clone was selected for. In most cases the new clone had new driver mutations or CNAs, including *TP53*, *NRAS Q61* and amp1q21. Although our WES strategy did not allow detection of structural variants and other non-coding drivers [34, 47], acquisition of such drivers at progression would have been detected implicitly through a shift in clonal architecture.

This suggests that genomic changes are involved in the phenotypic transformation. However, one patient had increased PI, but no changes in the genome at the sensitivity level applied in our studies. This observation is principally important because it indicates that other mechanisms than genomic changes and clonal selection, such as microenvironmental factors, are involved in disease progression.

Our data demonstrate a heterogenous evolution of the genomic landscape based on the high number of mutations appearing and disappearing throughout the disease course as well as a considerable number of mutations appearing in only one patient. We confirmed a further increase of mutational load and the presence of the mutational signature SBS-MM1 in patients who had received HDM [23, 24]. Additionally, we found the mutational signature in two patients who received LDM. Absence of SBS-MM1 in the pooled analysis of LDM patients may be explained by a dilution effect because most patients lack the signature. Thus, our study provides proof of principle that melphalan can have a mutagenic effect on myeloma cells irrespective of dose-intensity. HDM consists of melphalan 150–200 mg/sqm given once whereas LDM is 36 mg/sqm given during 4 days and repeated every month. The accumulated dose will after 10 months be comparable to HDM, but with another profile of exposure.

SBS-MM1 could only be detected in patients who had a differential or linear pattern of clonal evolution. This observation supports the proposed model where chemotherapy-related signatures become detectable when one affected cell expands to clonal dominance [48]. Intensive therapy which induces a deep response provides fertile ground for such a clonal expansion but is not a pre-requisite. Indeed, one of the patients had a differential clonal response and was resistant to LDM.

The pathways that were transcriptionally upregulated at later disease stages were highly correlated. MYC is an important regulator of metabolic pathways, including glycolysis and oxidative phosphorylation (reviewed in [49]). Our data suggest that MYC was regulated partly at the post-transcriptional level. Indeed, MYC is regulated by MTORC1 signaling [50] and stabilized by activated RAS [51]. Both MYC activity and loss of *TP53* can promote glycolysis [52]. Most samples with increased transcription of glycolysis genes also had increased expression of genes involved in oxidative phosphorylation. This is in line with growing evidence that oxidative phosphorylation is active in many malignancies, including leukemias and lymphomas, and emerges as a potential treatment target [53].

Interestingly, TNF signaling via NFκB was downregulated later in the disease course, and was inversely correlated with the proliferative and metabolic pathways. TNF via NFκB signaling has in a recent study been found to be downregulated in patients with double refractory disease [11], and in extramedullary disease [45]. Our results also fit with a previous study showing complementary activation of MYC and NFκB in diagnostic tumors [34], and our results suggests more active MYC and less NFκB dependent tumors in late-stage disease.

We found significantly increased expression levels of the ABC transporters and nuclear exportins in relapsed patients with high PI (Fig. 5). XPO1 is an important transporter of more than 200 nuclear cargo proteins including many tumor suppressor proteins. Overexpression of *XPO1* has been demonstrated in myeloma cell lines as well as in myeloma cells from patients [36] and was a rationale for testing of the XPO1-inhibitor selinexor in patients. In 2019 this drug was approved for myeloma patients that had acquired resistance against 4 prior drugs. If the effect of selinexor is dependent on the level of expression, patients with highly proliferative tumors having high expression may benefit from this treatment. We have not identified studies that have examined the relation between *XPO1* expression and effect of selinexor in more detail.

Another potentially clinically relevant finding in this work is the increased expression of CGAs detected at later disease stages. CGA could be directly involved in regulating proliferation by preventing cyclin degradation or inhibition [54]. CGA expression and anti-CGA immune responses have been demonstrated in MM [55, 56], but have to the best of our knowledge not been analyzed in longitudinal samples before. CGAs can elicit immune responses [55] and are expressed in progressive tumors, yet this is not enough to reject the tumor. Whether these CGAs also are translated and presented to T cells with MHC molecules, needs to be verified. It is also not known whether T cells against CGAs are present in patients at later disease stages or if these cells are unable to reject the tumor due to immune suppression in the tumor microenvironment or insufficient T cell activation. One of the hallmarks of late disease is a suppressed immune system which may allow myeloma cells expressing CGAs to expand. Although these CGAs are potential targets for immunotherapy, clinical studies in MM so far have not been successful (NCT01245673). However, TCR-engineered T cells against MAGE may be more efficient (NCT03139370).

A main conclusion of this study is that disease progression eventually is characterized by a transcriptomic convergence into a more proliferative phenotype supported by expression of genes that enable the tumor cells to tolerate increased cell growth and energy demand.

## Supplementary information

Supplementary Methods

Supplementary Figures

Supplementary Tables

Table S6

Supplementary Tables Overview

Table S1

Table S2

Table S3A

Table S3B

Table S4

Table S5

Table S6

Table S7

Table S8

Table S9

Table S10

Table S11

Table S12

## Data Availability

Processed data from RNA sequencing could be found in GEO under accession number GSE179929. List of all SNVs detected in the in-house dataset can be found in Table S6. Pile-ups for all data, and list of detected InDels will be provided on request. For access to raw data, contact the authors. In-house scripts used in the methods, will be provided on request by contacting the authors.

## References

[1] Bolli N, Avet-Loiseau H, Wedge DC, Van Loo P, Alexandrov LB, Martincorena I (2014). Heterogeneity of genomic evolution and mutational profiles in multiple myeloma. Nat Commun.

[2] Chapman MA, Lawrence MS, Keats JJ, Cibulskis K, Sougnez C, Schinzel AC (2011). Initial genome sequencing and analysis of multiple myeloma. Nature.

[3] Lohr JG, Stojanov P, Carter SL, Cruz-Gordillo P, Lawrence MS, Auclair D (2014). Widespread genetic heterogeneity in multiple myeloma: implications for targeted therapy. Cancer Cell.

[4] Maura F, Bolli N, Angelopoulos N, Dawson KJ, Leongamornlert D, Martincorena I (2019). Genomic landscape and chronological reconstruction of driver events in multiple myeloma. Nat Commun.

[5] Walker BA, Mavrommatis K, Wardell CP, Ashby TC, Bauer M, Davies FE (2018). Identification of novel mutational drivers reveals oncogene dependencies in multiple myeloma. Blood.

[6] Corre J, Cleynen A, Robiou du Pont S, Buisson L, Bolli N, Attal M (2018). Multiple myeloma clonal evolution in homogeneously treated patients. Leukemia.

[7] Jones JR, Weinhold N, Ashby C, Walker BA, Wardell C, Pawlyn C, et al. Clonal evolution in myeloma: the impact of maintenance lenalidomide and depth of response on the genetics and sub-clonal structure of relapsed disease in uniformly treated newly diagnosed patients. Haematologica. 2019;104:1440–50. 10.3324/haematol.2018.202200 PMC6601103 30733268

[8] Keats JJ, Chesi M, Egan JB, Garbitt VM, Palmer SE, Braggio E (2012). Clonal competition with alternating dominance in multiple myeloma. Blood.

[9] Krönke J, Udeshi ND, Narla A, Grauman P, Hurst SN, McConkey M (2014). Lenalidomide causes selective degradation of IKZF1 and IKZF3 in multiple myeloma cells. Science.

[10] Weinhold N, Ashby C, Rasche L, Chavan SS, Stein C, Stephens OW (2016). Clonal selection and double-hit events involving tumor suppressor genes underlie relapse in myeloma. Blood.

[11] Ziccheddu B, Biancon G, Bagnoli F, De Philippis C, Maura F, Rustad EH (2020). Integrative analysis of the genomic and transcriptomic landscape of double-refractory multiple myeloma. Blood Adv.

[12] Maura F, Weinhold N, Diamond B, Kazandjian D, Rasche L, Morgan G (2021). The mutagenic impact of melphalan in multiple myeloma. Leukemia.

[13] Szalat R, Avet-Loiseau H, Munshi NC (2016). Gene Expression Profiles in Myeloma: Ready for the Real World?. Clin Cancer Res.

[14] Ziccheddu B, Da Vià MC, Lionetti M, Maeda A, Morlupi S, Dugo M (2021). Functional Impact of Genomic Complexity on the Transcriptome of Multiple Myeloma. Clin Cancer Res.

[15] Cohen YC, Zada M, Wang SY, Bornstein C, David E, Moshe A (2021). Identification of resistance pathways and therapeutic targets in relapsed multiple myeloma patients through single-cell sequencing. Nat Med.

[16] Dutta AK, Alberge JB, Sklavenitis-Pistofidis R, Lightbody ED, Getz G, Ghobrial IM, Single-cell profiling of tumour evolution in multiple myeloma - opportunities for precision medicine. Nat Rev Clin Oncol. 2022;19:223–36. 10.1038/s41571-021-00593-y 35017721

[17] Cavo M, Gay F, Beksac M, Pantani L, Petrucci MT, Dimopoulos MA (2020). Autologous haematopoietic stem-cell transplantation versus bortezomib-melphalan-prednisone, with or without bortezomib-lenalidomide-dexamethasone consolidation therapy, and lenalidomide maintenance for newly diagnosed multiple myeloma (EMN02/HO95): a multicentre, randomised, open-label, phase 3 study. Lancet Haematol.

[18] Zweegman S, van der Holt B, Mellqvist UH, Salomo M, Bos GM, Levin MD (2016). Melphalan, prednisone, and lenalidomide versus melphalan, prednisone, and thalidomide in untreated multiple myeloma. Blood.

[19] Skerget S, Penaherrera D, Chari A, Jagannath S, Siegel DS, Vij R (2021). Genom Basis Mult Myeloma Subtypes MMRD CoMMpass Study.

[20] Rustad EH, Dai HY, Hov H, Coward E, Beisvag V, Myklebost O (2015). BRAF V600E mutation in early-stage multiple myeloma: good response to broad acting drugs and no relation to prognosis. Blood Cancer J.

[21] Favero F, Joshi T, Marquard AM, Birkbak NJ, Krzystanek M, Li Q (2015). Sequenza: allele-specific copy number and mutation profiles from tumor sequencing data. Ann Oncol.

[22] Shen R, Seshan VE (2016). FACETS: allele-specific copy number and clonal heterogeneity analysis tool for high-throughput DNA sequencing. Nucleic Acids Res.

[23] Rustad EH, Nadeu F, Angelopoulos N, Ziccheddu B, Bolli N, Puente XS (2021). mmsig: a fitting approach to accurately identify somatic mutational signatures in hematological malignancies. Commun Biol.

[24] Rustad EH, Yellapantula V, Leongamornlert D, Bolli N, Ledergor G, Nadeu F (2020). Timing the initiation of multiple myeloma. Nat Commun.

[25] Zhan F, Huang Y, Colla S, Stewart JP, Hanamura I, Gupta S (2006). The molecular classification of multiple myeloma. Blood.

[26] Markert EK, Mizuno H, Vazquez A, Levine AJ (2011). Molecular classification of prostate cancer using curated expression signatures. Proc Natl Acad Sci USA.

[27] Mehmet S, Roncador M, Aktas-Samur A, Fulciniti M, Bazarbachi A, Szalat R (2020). High-Dose Melphalan Significantly Increases Mutational Burden In Multiple Myeloma Cells At Relapse: Results From A Randomized Study In Multiple Myeloma. Blood.

[28] Walker BA, Boyle EM, Wardell CP, Murison A, Begum DB, Dahir NM (2015). Mutational spectrum, copy number changes, and outcome: results of a sequencing study of patients with newly diagnosed myeloma. J Clin Oncol.

[29] Barrio S, Stuhmer T, Da-Via M, Barrio-Garcia C, Lehners N, Besse A (2019). Spectrum and functional validation of PSMB5 mutations in multiple myeloma. Leukemia.

[30] Kortum KM, Mai EK, Hanafiah NH, Shi CX, Zhu YX, Bruins L (2016). Targeted sequencing of refractory myeloma reveals a high incidence of mutations in CRBN and Ras pathway genes. Blood.

[31] Gooding S, Ansari-Pour N, Towfic F, Ortiz Estevez M, Chamberlain PP, Tsai KT, et al. Multiple Cereblon genetic changes associate with acquired resistance to Lenalidomide or Pomalidomide in Multiple Myeloma. Blood. 2021;137:232–7. 10.1182/blood.2020007081 PMC7893409 33443552

[32] Liberzon A, Birger C, Thorvaldsdottir H, Ghandi M, Mesirov JP, Tamayo P (2015). The Molecular Signatures Database (MSigDB) hallmark gene set collection. Cell Syst.

[33] Affer M, Chesi M, Chen WD, Keats JJ, Demchenko YN, Tamizhmani K (2014). Promiscuous MYC locus rearrangements hijack enhancers but mostly super-enhancers to dysregulate MYC expression in multiple myeloma. Leukemia.

[34] Misund K, Keane N, Stein CK, Asmann YW, Day G, Welsh S (2020). MYC dysregulation in the progression of multiple myeloma. Leukemia.

[35] Annunziata CM, Davis RE, Demchenko Y, Bellamy W, Gabrea A, Zhan F (2007). Frequent engagement of the classical and alternative NF-kappaB pathways by diverse genetic abnormalities in multiple myeloma. Cancer Cell.

[36] Tai YT, Landesman Y, Acharya C, Calle Y, Zhong MY, Cea M (2014). CRM1 inhibition induces tumor cell cytotoxicity and impairs osteoclastogenesis in multiple myeloma: molecular mechanisms and therapeutic implications. Leukemia.

[37] Soriano GP, Besse L, Li N, Kraus M, Besse A, Meeuwenoord N (2016). Proteasome inhibitor-adapted myeloma cells are largely independent from proteasome activity and show complex proteomic changes, in particular in redox and energy metabolism. Leukemia.

[38] Byrgazov K, Kraus M, Besse A, Slipicevic A, Lehmann F, Driessen C (2021). Up-regulation of multidrug resistance protein MDR1/ABCB1 in carfilzomib-resistant multiple myeloma differentially affects efficacy of anti-myeloma drugs. Leuk Res.

[39] Heintel D, Rocci A, Ludwig H, Bolomsky A, Caltagirone S, Schreder M (2013). High expression of cereblon (CRBN) is associated with improved clinical response in patients with multiple myeloma treated with lenalidomide and dexamethasone. Br J Haematol.

[40] Qian X, Dimopoulos MA, Amatangelo M, Bjorklund C, Towfic F, Flynt E (2019). Cereblon gene expression and correlation with clinical outcomes in patients with relapsed/refractory multiple myeloma treated with pomalidomide: an analysis of STRATUS. Leuk Lymphoma.

[41] Dufva O, Pölönen P, Brück O, Keränen MAI, Klievink J, Mehtonen J (2020). Immunogenomic Landscape of Hematological Malignancies. Cancer Cell.

[42] Pardoll DM (2012). The blockade of immune checkpoints in cancer immunotherapy. Nat Rev Cancer.

[43] Wang JH, Smolyar A, Tan K, Liu JH, Kim M, Sun ZY (1999). Structure of a heterophilic adhesion complex between the human CD2 and CD58 (LFA-3) counterreceptors. Cell.

[44] Yang R, Elsaadi S, Misund K, Abdollahi P, Vandsemb EN, Moen SH, et al. Conversion of ATP to adenosine by CD39 and CD73 in multiple myeloma can be successfully targeted together with adenosine receptor A2A blockade. J Immunother Cancer. 2020;8:e000610. 10.1136/jitc-2020-000610 PMC7239696 32409420

[45] Ryu D, Kim SJ, Hong Y, Jo A, Kim N, Kim HJ (2020). Alterations in the transcriptional programs of myeloma cells and the microenvironment during extramedullary progression affect proliferation and immune evasion. Clin Cancer Res.

[46] Boyle EM, Rosenthal A, Wang Y, Farmer P, Rutherford M, Ashby C, et al. High-risk transcriptional profiles in multiple myeloma are an acquired feature that can occur in any subtype and more frequently with each subsequent relapse. Br J Haematol. 2021;195:283–6. 10.1111/bjh.17670 34244996

[47] Rustad EH, Yellapantula VD, Glodzik D, Maclachlan KH, Diamond B, Boyle EM (2020). Revealing the impact of structural variants in multiple myeloma. Blood Cancer Disco.

[48] Landau HJ, Yellapantula V, Diamond BT, Rustad EH, Maclachlan KH, Gundem G (2020). Accelerated single cell seeding in relapsed multiple myeloma. Nat Commun.

[49] Goetzman ES, Prochownik EV (2018). The role for Myc in coordinating glycolysis, oxidative phosphorylation, glutaminolysis, and fatty acid metabolism in normal and neoplastic tissues. Front Endocrinol.

[50] Pourdehnad M, Truitt ML, Siddiqi IN, Ducker GS, Shokat KM, Ruggero D (2013). Myc and mTOR converge on a common node in protein synthesis control that confers synthetic lethality in Myc-driven cancers. Proc Natl Acad Sci USA.

[51] Sears R, Leone G, DeGregori J, Nevins JR (1999). Ras enhances Myc protein stability. Mol Cell.

[52] Lunt SY, Vander Heiden MG (2011). Aerobic glycolysis: meeting the metabolic requirements of cell proliferation. Annu Rev Cell Dev Biol.

[53] Ashton TM, McKenna WG, Kunz-Schughart LA, Higgins GS (2018). Oxidative phosphorylation as an emerging target in cancer therapy. Clin Cancer Res.

[54] Shires K, Van, Wyk T (2018). The role of Cancer/Testis Antigens in Multiple Myeloma pathogenesis and their application in disease monitoring and therapy. Crit Rev Oncol Hematol.

[55] Atanackovic D, Arfsten J, Cao Y, Gnjatic S, Schnieders F, Bartels K (2007). Cancer-testis antigens are commonly expressed in multiple myeloma and induce systemic immunity following allogeneic stem cell transplantation. Blood.

[56] van Duin M, Broyl A, de Knegt Y, Goldschmidt H, Richardson PG, Hop WC (2011). Cancer testis antigens in newly diagnosed and relapse multiple myeloma: prognostic markers and potential targets for immunotherapy. Haematologica.

