# Supplementary material for: Clonal evolution after treatment pressure in multiple myeloma: heterogenous genomic aberrations and transcriptomic convergence
Source: Leukemia. 2022 May 28;36(7):1887–97. doi: 10.1038/s41375-022-01597-y (PMC9252918; doi:10.1038/s41375-022-01597-y)
Supplement: Supplementary file 1 — Supplementary Methods [file 41375_2022_1597_MOESM1_ESM.docx]

**Supplementary Methods**

*Patient samples*

In-house cohort

CD138-positive tumor cells were isolated using RoboSep (StemCell Technologies, Grenoble, France). Tumor purity was subsequently determined by microscopy of MGG-stained cytopspin slides with CD138+ cells or by flow cytometry. Only samples with a tumor purity ≥80% were included in the study (range 80-100, median 95%) (Table S2). There was an average of 22 [range 5-70] months between samples for diagnosis-progression pairs (n=23), and 14 [range 2-38] months for progression-progression pairs (n=18) (Table S1A). Clinical data are summarized in Table 1.

MMRF CoMMpass cohort

RNA Seq expression data (E74GTF_Salmon_Gene_Counts and E74GTF_Salmon_Gene_TPM) from the CoMMpass IA13 release were downloaded from the MMRF webpage (http:://research.themmrf.org). Also RNA Seq (RNAseq_Canonical_Ig_Translocations.txt), WGS (LongInsert_Canonical_Ig_Translocation.txt) and WES data (CNA_Exome_FISH_CN.txt) were downloaded and used to define the presence of IgH translocations and to assess hyperdiploidy (Supplementary Table 12). For the MMRF cohort the average time between sampling was 25 [range 3-45] months for diagnosis/PD (n=28) and 18 [range 12-21] months for PD/PD pairs (n=3) (Table S1B). Overall survival data were updated with the IA16 release, using the file “STAND ALONE SURVIVAL.csv”.

*DNA and RNA extraction*

For the in-house cohort, tumor DNA and total RNA were extracted using All-Prep Kit (Qiagen, Hilden, Germany) according to the manufacturer's instructions. Germline DNA was extracted from peripheral blood using QIAamp DNA Mini kit (Qiagen).

*Whole Exome Sequencing (WES)*

WES of DNA from bone marrow myeloma cells and matched germline control have previously been described in detail [1]. In short, whole exome libraries were prepared using the Agilent SureSelectXT Human All Exon V5 kit and sequenced paired-end using an Illumina HiSeq2500 and HiSeq4000 instrument (Illumina Inc., San Diego, CA, USA). Libraries were sequenced to a mean coverage of >100 reads (length 100 and 150 bp, respectively) for 91% of the targeted exonic regions.

A table summarizing genomic quality metrics is given in Table S2.

*Alignment*

Alignment was performed using bwa mem (v0.7.15-r1140) using the Y option. The resulting BAM files were sorted using samtools sort. Duplicate reads were marked on the sorted BAM files using samtools markdup (1.10). Human genome build hg19 was used.

*Detection of somatic variants*

Somatic variant detection was performed using Strelka2 (v2.9.10). The targeted and call-regions options were used in combination with a padded interval file (BED) corresponding to the used capture. Subsequently the resulting variants were annotated using ANNOVAR (2019-10-24). An in-house developed tool was used to add fragment statistics from the tumor/normal BAM files (https://github.com/MathijsSanders/AnnotateBAMStatistics). A heuristic-based filtering was applied on the unfiltered variants lists. Variants with a strand bias over 95% , with less than 4 high quality tumor fragments, a coverage less than 8 high quality fragments and with a high percentage (40% or higher) of reads showing poor alignment scores were removed. Tumor in normal was estimated using the DeTiN package (v1.7.5.9) and somatic status of variants were adjusted accordingly. Somatic variant filtering was based upon the somatic status (judgement) and somatic p value (p_somatic_given_TiN) given by the DeTiN package. A somatic p value threshold of 0.5 was used. A panel of normals was applied to reduce the number of false positives in problematic regions. The selected normals all had no measurable tumor in normal load. Allele specific copy number estimated by sequenza was used as input for DeTiN.

*Calculation of Cancer Cell Fractions*

Cancer Cell Fractions (CCF) were computed using an in house developed program. Variant allele frequencies were measured based on fragments and if pairs overlap the base with the highest score was used. Allele specific copy number estimation was performed using Facets. The resulting segments files (cncf files) were used as an input. More specifically the cancer cell fraction, total copy number and minor copy number fields. Total copy number in the normal samples was assumed to be two except for chrX in male samples. Sample purity estimate from facets was also used as an input. The CCF or subclonal fraction for the somatic mutations was calculated using the formula below. Subsequently resulting CCF’s were used as an input to DPclust.

$$CCF=VAF\frac{P\cdot N_{T}+\left( 1-P \right)\cdot N_{N}}{P_{S}\cdot N_{B}}$$

$$\begin{aligned} P=Subclonalcellularfraction \\ N_{T}=Subclonalcopynumber \\ N_{N}=Normalcopynumber \\ P_{S}=Samplepurityestimate \\ N_{B}=Numberofallelesbearingthemutation \end{aligned}$$

When counting the number of SNVs present in a sample, all exonic SNVs with a CCF (Cancer Cell Fraction) > 5% were counted.

CCF have been used in all figures containing mutation data, except for Figure S1E where VAF (variant allele frequency) have been used.

*Cytogenetic subgroups*

We divided the patients into cytogenetic subgroups based on their RNA levels of the genes involved in primary translocations; CCND1 (t(11;14)), CCND2 (t(12;14)), CCND3 (t(6;14)), WHSC1 (NSD2/MMSET)(t(4;14)), MAF (t(14;16)), MAFA (t(8;14)), MAFB (t(14;20)), and based on hyperdiploidity (HRD) status, measured from Copy Number data; 3 or more extra odd number chromosomes (in-house cohort) or HRD Call “1” from the file “CNA_Exome_FISH_CN.txt” for the CoMMpass cohort. The estimated RNA cytogenetic subgroups correlated 88 % (7/8) with FISH data (in-house cohort) and 100% with long-insert WGS (CoMMpass cohort) (16/16) (Table S12). If both a translocation and a HRD is detected, the patient is grouped based on the translation group (2 cases). For specific subgroup analysis, the HRD group were further divided into Cyclin D1 expressers (HRD-C1), expressing low or medium levels of CCND1, or Cyclin D2 expressers (HRD-C2), expressing low or median levels of Cyclin D2 (Table S8).

*RNA Seq library construction and sequencing*

RNA concentration was measured using Qubit® RNA HS Assay Kit on a Qubit® 3.0 Fluorometer (Thermo Fisher Scientific Inc., Waltham, MA, USA). Integrity was assessed using Agilent RNA 6000 Pico Kit on a 2100 Bioanalyzer instrument (Agilent Technologies, Santa Clara, CA, USA).

RNA Seq libraries were prepared using TruSeq Stranded mRNA kit (Illumina, San Diego, CA, USA) according to the manufacturer's instructions. Briefly, 500 ng total RNA was used as starting material. First, index barcodes were ligated for identification of individual samples. mRNA was purified from the total RNA using poly-T oligo-attached magnetic beads, followed by random fragmentation using divalent cations at 94°C for 4 min. First and second strand cDNAs were synthesized using random oligonucleotides and SuperScript II, followed by DNA polymerase I and RNase H. Exonuclease/polymerase was used to produce blunted overhangs. Illumina SR adapter oligonucleotides were ligated to the cDNA after 3' end adenylation. DNA fragments were enriched by 15 cycles of PCR reaction. The libraries were purified using the AMPure XP (Beckman Coulter, Inc., Indianapolis, IN, USA), quantitated by qPCR using KAPA Library Quantification Kit (Kapa Biosystems, Inc., Wilmington, MA, USA) and validated using Agilent High Sensitivity DNA Kit on a Bioanalyzer (Agilent Technologies). The size range of the DNA fragments were measured to be in the range of app. 200-1000 bp and peaked around 310 bp.

Libraries were either

1) normalized and pooled to 2.2/2.5 pM (flowcell 1/flowcell2) and subjected to clustering on two NextSeq 500 high output flowcells. Finally paired end read sequencing was performed for 75X2 cycles on a NextSeq 500 instrument (Illumina, Inc. San Diego, CA, USA), according to the manufacturer's instructions. Base calling was done on the NS500 instrument by RTA 2.4.6.

or

2) normalized and pooled to 2.3 pM and subject to clustering by a cBot Cluster Generation System on a HiSeq4000 flowcell (Illumina Inc. San Diego, CA, USA), according to manufacturer’s instructions. Finally paired end read sequencing was performed for 75X2 cycles performed on a Illumina HiSeq4000 instrument, in accordance with the manufacturer’s instructions (Illumina, Inc., San Diego, CA, USA).

FASTQ files were created with bcl2fastq 2.17 (Illumina, Inc., San Diego, CA, USA).

Transcript expression values were generated by quasi alignment using salmon (http://salmon.readthedocs.io/en/latest/index.html) and the Ensembl (Hg19) human genome. Aggregation of transcript to gene expression was performed using tximport (http://bioconductor.org/packages/release/bioc/html/tximport.html) and normalized to transcripts per million (TPM) values.

A table summarizing genomic quality metrics from the RNA-seq is given in Table S2.

For the in-house cohort, identification of mutated RNA transcripts was done by comparing raw RNA variant files and bam files from alignment with called genomic variants using in-house scripts.

For detection of allelic expression of mutants, mutants that were detected at WES and where the coverage were >= 30 for both RNA and DNA were used in the analysis. Hence, on average, mutants with expressed allele frequency above 1/30 = 0.033. can be expected to be detected. Mutants with allele frequency of 0.1 and higher can be detected with more than 95% probability. For quantification of mutant allele expression, when the alternative allele count is $n_{A}$and the coverage is n, an approximate 95% confidence interval for each allele frequency, based on the binomial distribution, is given by $\hat{f}\pm1.96\hat{\sigma}$, where $\hat{f}=n_{A}/n$ is the estimated allele frequency with standard deviation $\hat{\sigma}=\sqrt{\hat{f}(1-\hat{f})/n}$.

*Differential expression analysis and GSEA*

A DESeq2 (v1.28.1) paired analysis was performed in R Studio to identify differentially expressed genes. GSEA (v4.1.0; Broad Institute), using hallmark gene sets, was performed to identify enriched pathways, by using a pre-ranked list of differential expressed genes from the DESeq analysis, sorted on (sign of logFC)(-log of the p-value).

*PI index, NFkB index, CGAs and HLA scores*

Proliferation Index (PI) was calculated according to Zhan F et al. [2] based on 11 genes previously been shown to be involved in proliferation. PI was the calculated average log2 Transcript Per Million (TPM) values of these 11 genes: TOP2A, BIRC5, CCNB2, NEK2, ANAPC7, STK6, BUB1, CDC2, C10orf3, ASPM, CDCA1. NFkB index (NFkBi) [3] was calculated using the average log2 transformed transcripts per million (TPM) for the eleven genes detailed in the original signature. For analysis of Cancer Germline Antigenes (CGA), we used a well-defined set of 27 CGA genes found to be recurrently expressed in hematological cancers, but not in normal hematopoietic cells. Their expression was linked to alterations in DNA methylation, as they were frequently hypomethylated[4]. HLA scores were estimated as previously described[4], specifically the geometric mean of the HLA II genes *HLA-DMA*, *HLA-DMB*, *HLA-DPA1*, *HLA-DPB1*, *HLA-DRA*, and *HLA-DRB1* was defined as HLA II score, and the geometric mean of known HLA I genes *B2M*, *HLA-A*, *HLA-B*, and *HLA-C* was used to detect HLA I expression in the samples.

1. Rustad EH, Dai HY, Hov H, Coward E, Beisvag V, Myklebost O*, et al.* BRAF V600E mutation in early-stage multiple myeloma: good response to broad acting drugs and no relation to prognosis. Blood Cancer J. 2015; 5**:** e299.

2. Zhan F, Huang Y, Colla S, Stewart JP, Hanamura I, Gupta S*, et al.* The molecular classification of multiple myeloma. Blood. 2006; 108**:** 2020-2028.

3. Annunziata CM, Davis RE, Demchenko Y, Bellamy W, Gabrea A, Zhan F*, et al.* Frequent engagement of the classical and alternative NF-kappaB pathways by diverse genetic abnormalities in multiple myeloma. Cancer Cell. 2007; 12**:** 115-130.

4. Dufva O, Pölönen P, Brück O, Keränen MAI, Klievink J, Mehtonen J*, et al.* Immunogenomic Landscape of Hematological Malignancies. Cancer Cell. 2020; 38**:** 380-399.e313.
