# Supplementary material for: Clonal evolution after treatment pressure in multiple myeloma: heterogenous genomic aberrations and transcriptomic convergence
Source: Leukemia. 2022 May 28;36(7):1887–97. doi: 10.1038/s41375-022-01597-y (PMC9252918; doi:10.1038/s41375-022-01597-y)
Supplement: Supplementary file 2 — Supplementary Figures [file 41375_2022_1597_MOESM2_ESM.pdf]

Supplementary Figure 1

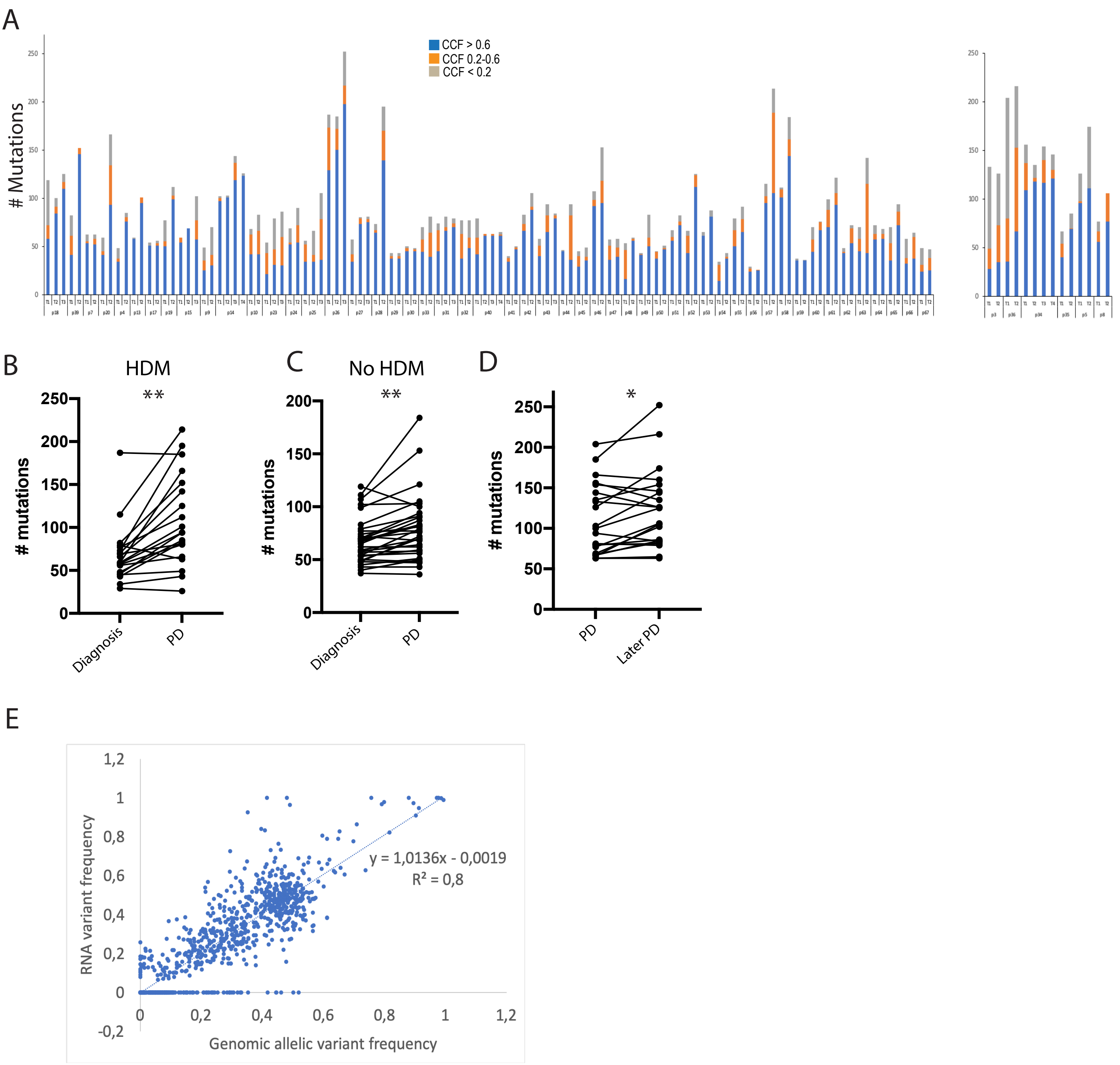

Fig. S1. Mutation changes and expression.
A) Total number of mutations and cancer cell fraction (CCF) in all included patients at all timepoints. CCF is divided in >60% (defined as a clonal mutation), 20-60% CCF (substantial mutation), and <20% (smaller subclonal mutation). All exonic SNVs (nonsynonymous and synonymous) with CCF>5% are included. Figure to the left shows patients with diagnostic-PD samples, to the right patients with PD-PD only. B) Mutation increase in treated versus untreated patients in patients receiving high dose melphalan (HDM) and those not (C). D) Patients with PD-PD samples. *p<0.05, **p<0.01, Wilcoxon rank sum test. For (B) and (C) the first available PD sample was used in the analysis. E). Correlation between the fraction of mutated variants in RNA and genomic variant allele frequency for the in-house samples. Each data point corresponds to one specific mutations, and only mutations detected in the genome where the total read depth (variant plus reference alleles) is at least 30 in both DNA and RNA are included. Most mutations appear around the diagonal, which suggests that the mutation does not skew the allelic expression. RNA was expressed in 49.8% of the mutated sites (50.3% at diagnosis, 49.5% at PD). Of these, mutated RNA alleles were found in 54.9% of cases (44.8%diagnosis, 59.8% PD).

Supplementary Figure 2

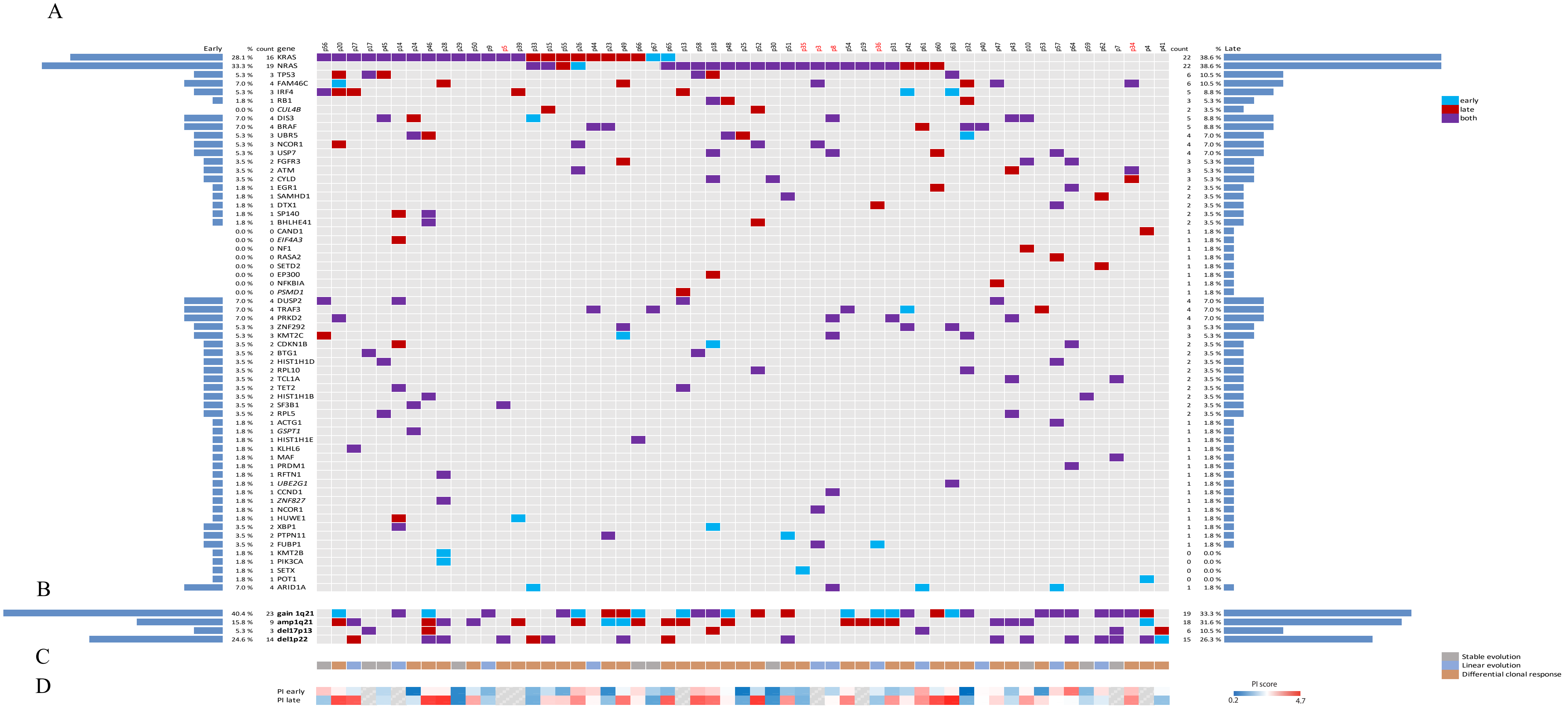

Fig. S2. Mutations, evolution pattern and PI change

A) Mutations (exonic and splicing) in 80 genes that has been defined as driver genes (Table S4) and drug resistance related genes. Only non-synonymous SNVs with CCF>5% and InDels with VAF>5% is included. When there are more than one late sample for a patient, mutations occurring in any of them are counted. Purple color means that a gene is mutated both early and late. The mutation prevalence is shown to the left (early) and right (late). B) Selected copy-number changes, C) Clonal evolution pattern, and D) PI (Proliferative index) in early and late samples. Patient ID is given on the top, red font means that the early sample is a PD sample. Gene names in italic means a drug resistant related gene, otherwise driver gene. For more details regarding subclonal mutations, SNV/InDel, see also Table S5.

Supplementary Figure 3

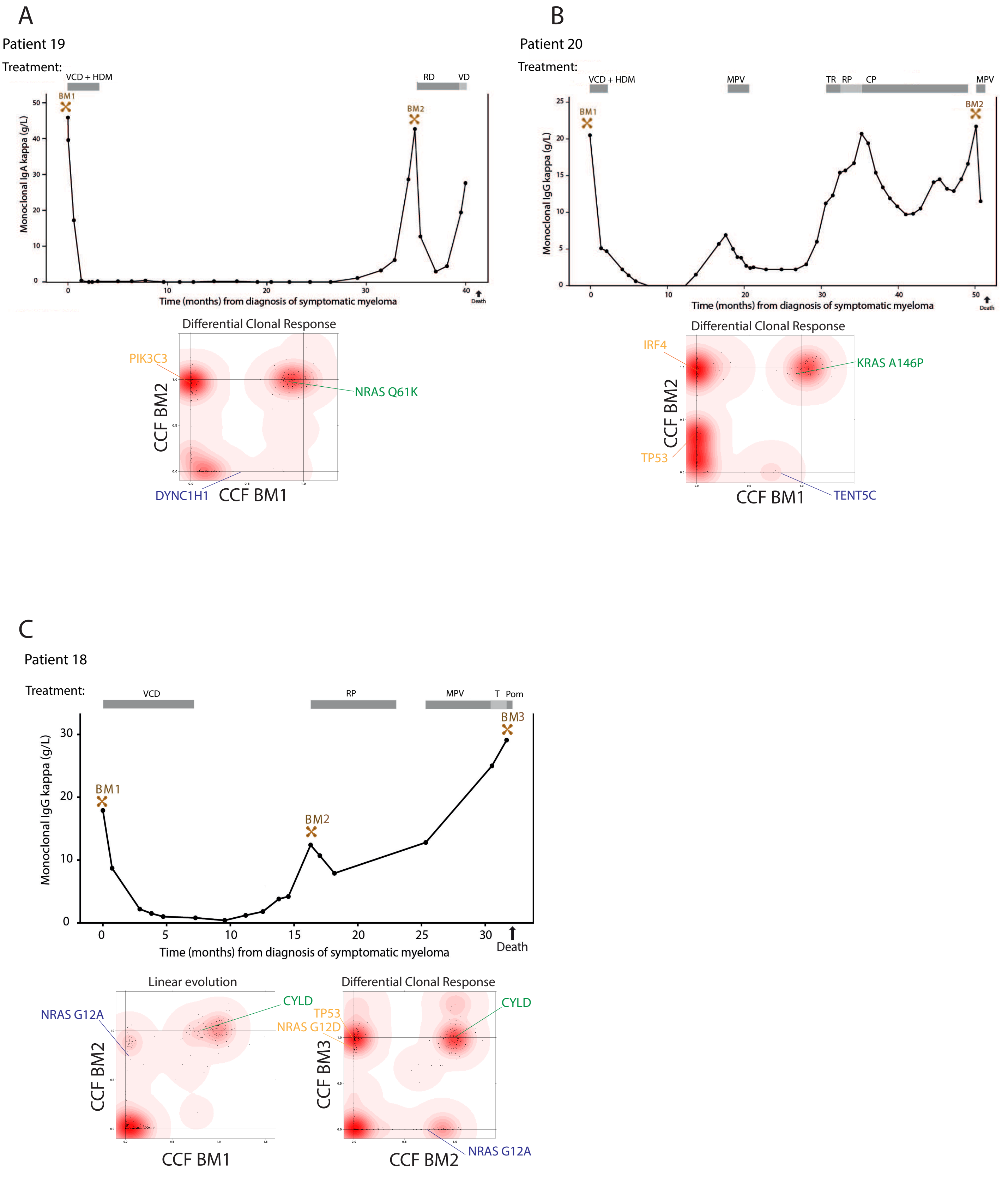

Fig. S3. Patient examples with shift in clonal dominance
Three patient examples with corresponding clonal evolution data. M component curves used to monitor the disease are shown for each of the patients and additional DPclust patterns below. For the DPclust figures: one black dot is one mutation. Orange: example of mutations appearing/increasing at the latest bone marrow, blue: example of mutation disappearing, green: example of mutations present in both samples. A) and B) Patients have a dominating clone at PD with a high increase of mutations compared to diagnosis. Both have undergone high-dose melphalan (HDM). C) Patients with a RAS shift. The patient had a dominating clone at late PD (BM3) with a TP53 mutation. Copy Number analysis showed a del17p, giving a biallelic TP53 aberration at end-stage disease. Treatment are stated above the M component curves. CCF: Cancer Cell Fraction, V: Velcade, C: Cyclophospamide, D: Dexamethasone, M: Melphalan, P: Prednisone, R: Revlimid, T:Thalidomide.

Supplementary Figure 4

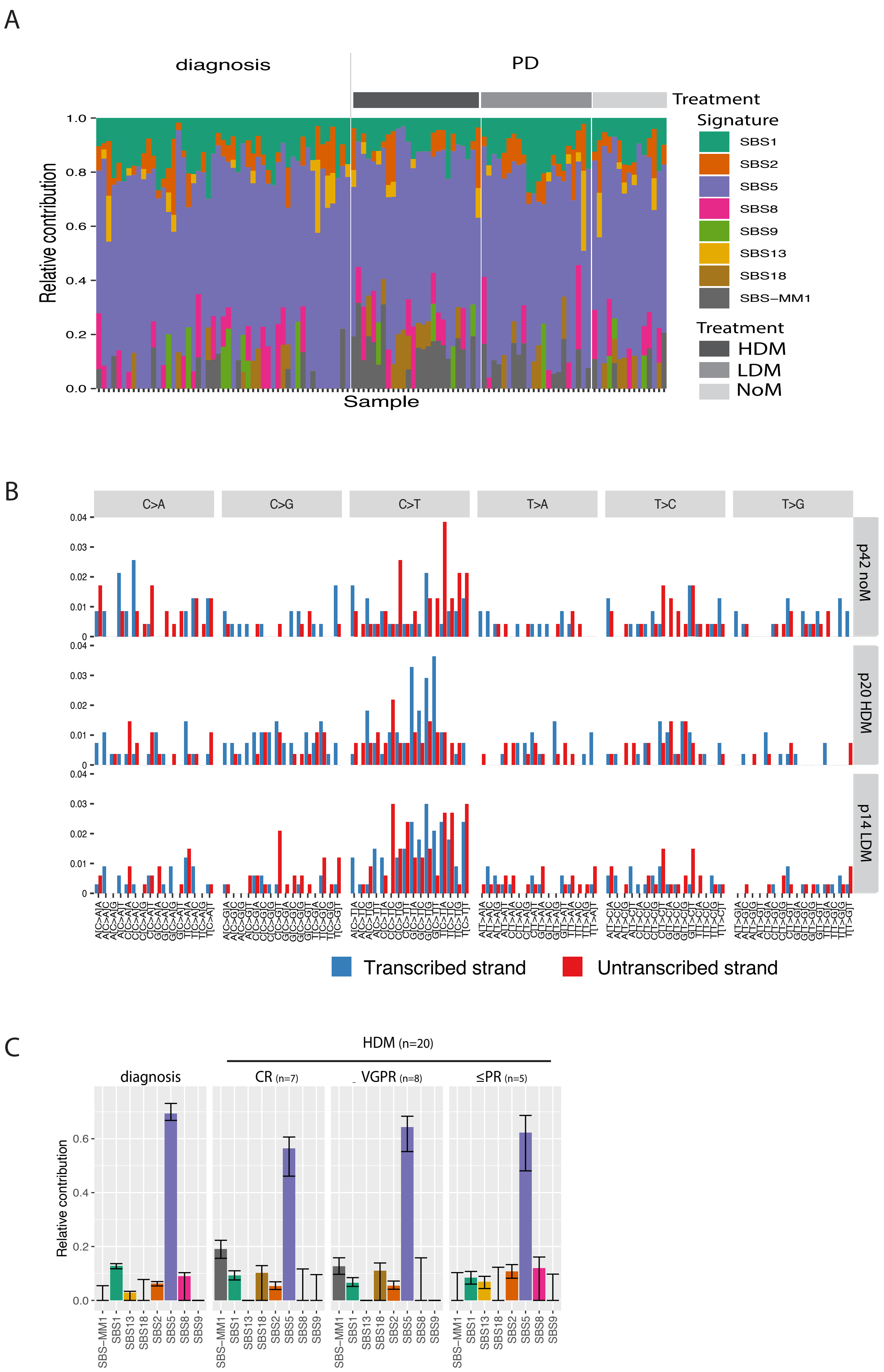

Fig. S4. Mutation signatures

A) Mutation signatures in individual patients before and after treatment. Patients are grouped based on treatment with HDM (high dose melphalan), LDM (low dose melphalan) and noM (no melphalan).

B) Relative contribution and pattern of the 96 mutational classes highlighting mutations on the transcribed (blue) and untranscribed (red) strands. Mutational profile of a relapsed patient not exposed to melphalan (top), exposed to HDM (middle) and exposed to LDM (bottom).Both patients exposed to melphalan showed strong evidence of SBS-MM1 by mmsig, including 95 % CI > 0 and statistically significant typical transcriptional strand bias. C) Group analysis of mutational signatures at diagnosis and after exposition to HDM divided in groups according to response. CR: complete response, VGPR: very good partial response. < PR: partial response or less.

Supplementary Figure 5

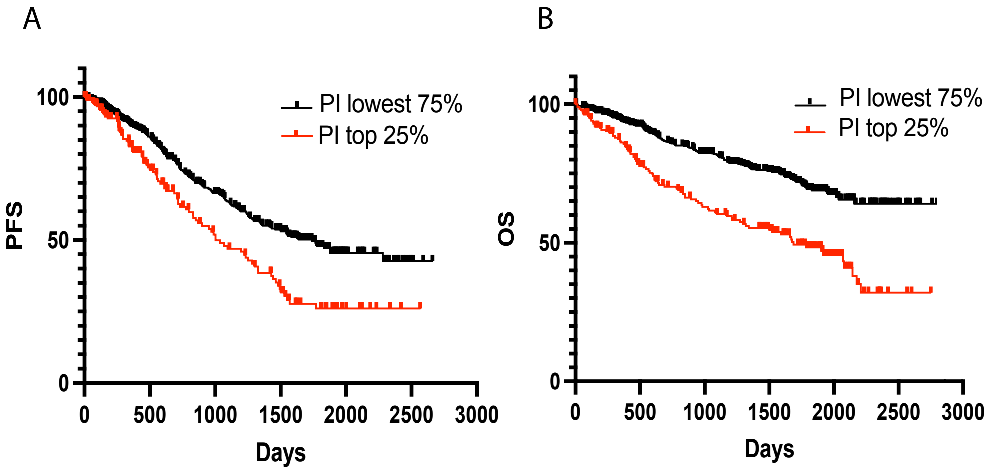

Multivariate Cox analysis on OS data	Significance	HR	95.0% CI for HR Lower	Upper
Proliferative index high (top 25%)	0.000	1.798	1.333	2.424
ISS	0.000	1.799	1.490	2.173
del17p	0.009	1.793	1.159	2.772
t(14;16)	0.784	0.904	0.442	1.852
t(4;14)	0.394	1.181	0.805	1.733

Fig. S5. High PI gives shorter OS and PFS
754 patients from the CoMMpass IA13 cohort divided in two groups according to the 75 % percentile of PI. A) PFS, B) OS. High PI gives a significantly shorter PFS (p<0.0001, HR:1.8(CI:1.4-2.5)) and OS (p<0.0001, HR:2.6(CI:1.9-3.6)). Significance is measured by log-rank Mantel-Cox test. Below is shown multivariate analysis using cox regression on OS data from B), showing that high PI gives significantly worse outcome.

Supplementary Figure 6

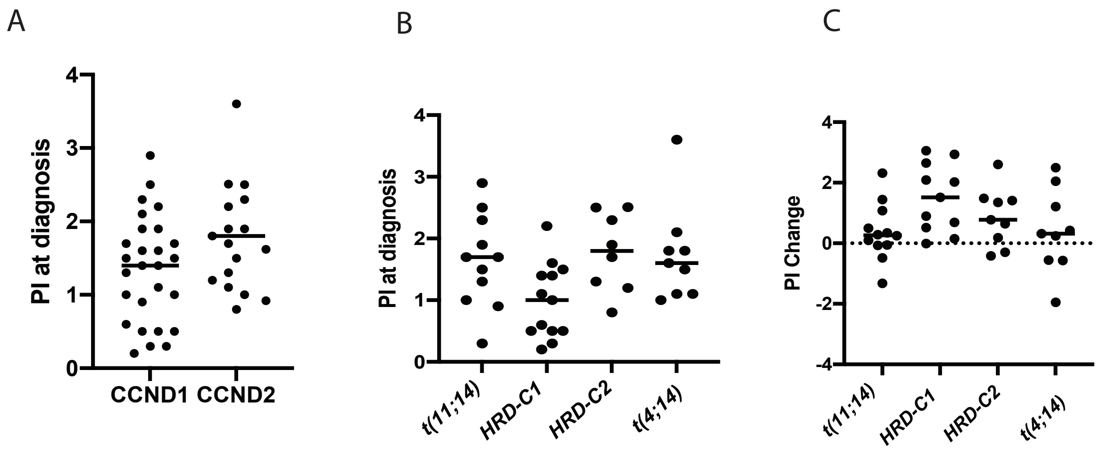

Fig. S6. PI levels and changes in different molecular subgroups
A) Tumors expressing Cyclin D2 (CCND2) had tendency of higher diagnostic PI levels (p=0.08; Mann Whitney test). B) Dividing the HRD tumors into CyclinD1 (CCND1) and CCND2 expressers (Table S8), the HRD group expressing Cyclin D1 (HRD-C1) had non-significant trend towards lower diagnostic PI levels than the other groups (p=0.08; Kruskal-Wallis test). C) No significant changes were found in PI increase from diagnosis to 1st PD between the different molecular subgroups.

Supplementary Figure 7

A

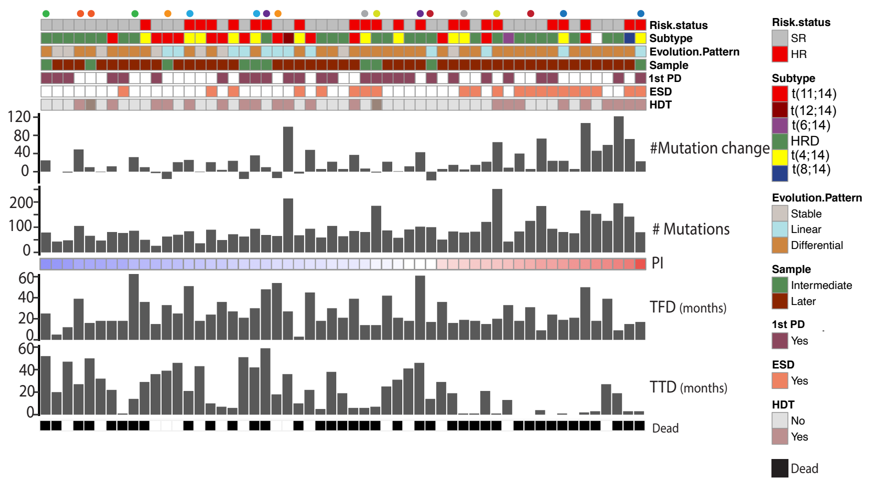

B

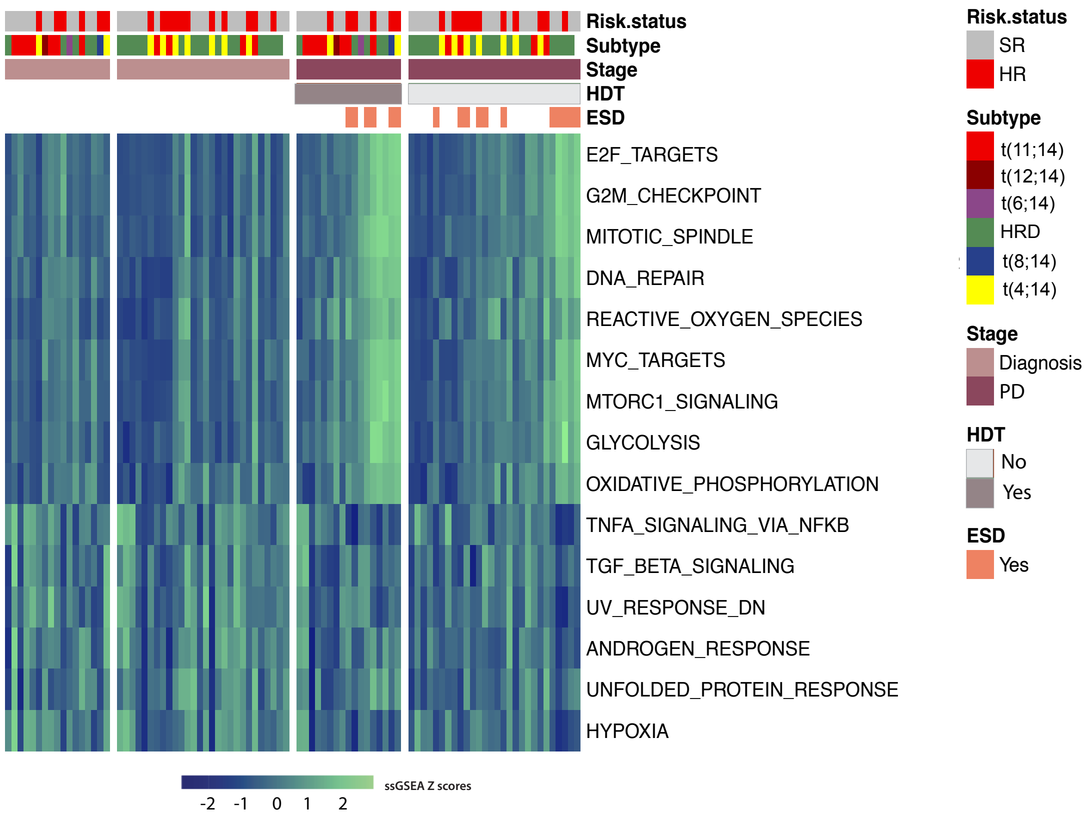

Fig. S7. Overview of included PD samples
A) An overview of analyzed PD samples and their PI, mutation load (mutations#), mutation change from diagnosis, and whether they had HDM during their disease course, and time from diagnosis and death/last control. Intermediate sample means that a later PD sample is available for this patient. Only patients that have an early sample taken at diagnosis are included. Colored dots show patients with >1 PD sample (identical color = same patient). The heatmap is sorted on increase in PI. HDM: High-dose melphalan, ESD: end stage disease, TFD: time from diagnosis, TTD; time from sampling to last control/death, black box below TTD shows dead patients. B) Heatmap for selected pathways showing single sample GSEA (ssGSEA) for early-late pairs, as in Fig. 3F), but divided into treatment groups HDM and no HDM. Patients are sorted by increased PI for latest sample. Paired samples are listed in order, the first patient; early sample to the far left, late sample far left of the late stage. Order: Diagnosis-HDM pair, Diagnosis – noHDM pair, PD-HDM pair, PD-noHDM pair. SR: standard risk according to ISS/R-ISS, HR: High risk, ESD: End-stage disease (<12 months from death). Cytogenetic subgroups; t(11;14)/CCND1, t(12;14)/ CCND2, t(6;14)/ CCND3, t(4;14)/WHSC1(NSD2/MMSET), t(8;14)/MAFA, are shown.

Supplementary Figure 8

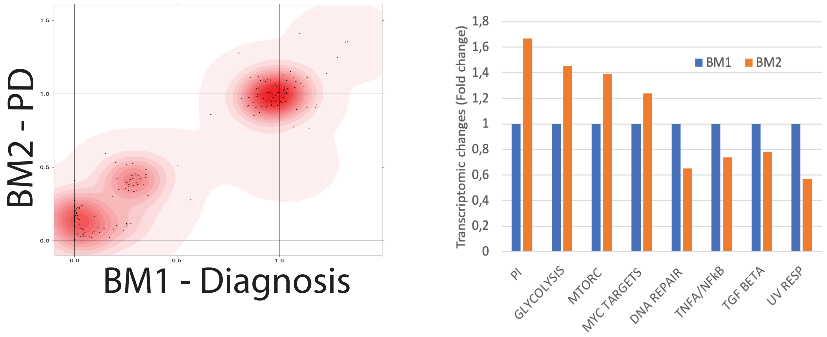

Fig. S8. Patient with stable evolution and transcriptomic changes

Example of a patient with a stable evolution shown by DPclust plot which shows distinct changes at the transcriptomic level. To the left is shown the DPclust plot showing no/few changes at the genomic level. One black dot is one mutation. The x-axis shows CCF (cancer cell fraction) at diagnosis, the y axis shows CCF at PD. The graph to the right shows to transcriptomic fold change in different pathways at PD compared to the level at diagnosis, using PI and ssGSEA scores (see also Tables S9 and S10).

Supplementary Figure 9

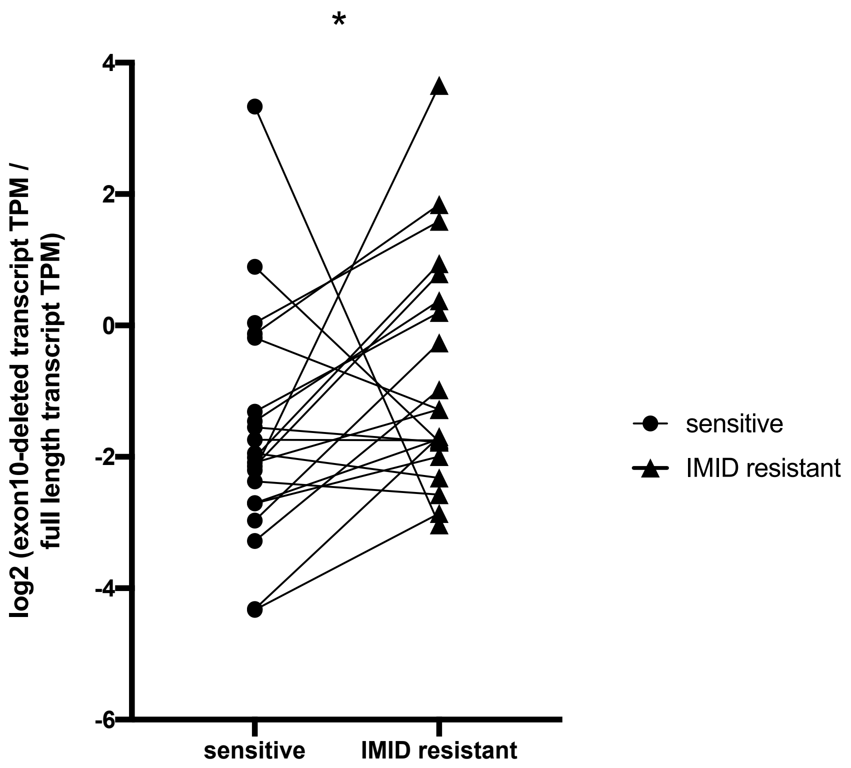

Fig. S9. Expression of CRBN transcripts.
The graph shows the ratio of expression (LOG2 TPM) of exon10 depleted CRBN transcript to full length CRBN transcript in patients at the time of IMID resistant PD and at an earlier timepoint. *<0.05 (Wilcoxon rank sum test).

Supplementary Figure 10

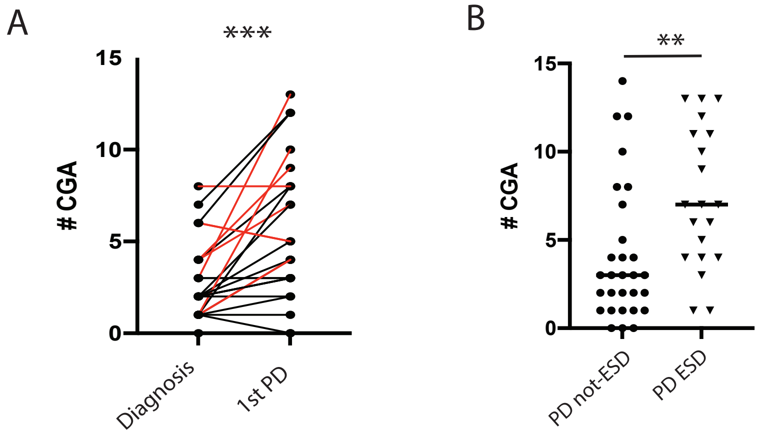

Fig S10. Cancer germline antigens at diagnosis and PD
A) Number of expressed CGAs (TPM>2) in paired samples diagnosis-1st PD. Red curves: paired samples with end-stage disease sample.(***p<0001; Wilcoxon) B) Number of CGA expressed at PD in samples not being end-stage versus end-stage disease (ESD). (**p<0.001; Mann Whitney). CGA: Cancer germline antigen
