# Supplementary material for: Clonal evolution after treatment pressure in multiple myeloma: heterogenous genomic aberrations and transcriptomic convergence
Source: Leukemia. 2022 May 28;36(7):1887–97. doi: 10.1038/s41375-022-01597-y (PMC9252918; doi:10.1038/s41375-022-01597-y)
Supplement: Supplementary file 5 — Supplementary Tables Overview [file 41375_2022_1597_MOESM5_ESM.pdf]

**Overview Supplementary Tables**

Table S1
Overview of included patient samples from A) "in-house" and B) the MMRF CoMMpass study.

Table S2
Table summarizing genomic quality metrics of samples, WES and RNAseq data.

Table S3
Clinical data from patients included from A) own-institutions and B) the MMRF CoMMpass study.

Table S4
List of Multiple Myeloma driver genes.

Table S5
Overview of clonal evolution, selected mutations and CNVs in all samples

Table S6
List of all detected exonic and splicing SNVs in all samples in the in-house cohort

Table S7
Results from differential expression analysis of late (latest available progression sample) versus early (1st) sample (paired DESeq analysis).
Only genes with Fold Change >1.5 and adj p-value <0.05 are shown.

Table S8
Proliferative index for all samples, combined with other clinical information including Cyclin D levels, PFS and risk status.

Table S9
ssGSEA levels of selected hallmark pathways. Also, transcript levels of MYC (TPM), measured NFkB index (NFkBi) and NFkB pathway mutations are shown.

Table S10
Proliferative index for earliest and latest sample and their clonal evolution pattern.

Table S11
Mutations in ABC transporters, nuclear exportins, relevant treatment targets including CD38, BCL2 and SLAMF7 (Figure 5), CGA genes, HLA I and II genes and inhibitory and stimulatory receptors (Figure 6A).
RNA expression are shown for samples with genomic mutation present, both whether the gene is expressed (RNA depth) and whether the mutated allele is expressed (RNA alt freq).

Table S12
Comparison of Cytogenetic subgroup with a) FISH data for in-house samples, and b) long-insert WGS for CoMMpass samples.
