# Supplementary material for: Clonal evolution after treatment pressure in multiple myeloma: heterogenous genomic aberrations and transcriptomic convergence
Source: Leukemia. 2022 May 28;36(7):1887–97. doi: 10.1038/s41375-022-01597-y (PMC9252918; doi:10.1038/s41375-022-01597-y)
Supplement: Supplementary file 6 — Table S1 [file 41375_2022_1597_MOESM6_ESM.pdf]

Table S1. Overview of analysis in samples from A) St.Olavs / Rotterdam, and B) IA13 CoMMpass cohort. PI: Proliferative index, TFD: Time from diagnosis of symtomatic MM (months).Sample type in Table A) All BM samples except *: PB. E: early, L: late, P1: 1st progression. PI: proliferative index, ssGSEA: single-sample GSEA.

A)

Patient_ID	Sample type	TFD	WES	RNAseq	DESeq (E-L)	DESeq (D-P1)	PI/ssGSEA	Clonality analysis
3	Progression 1	Month 29	x					x
	Progression 2	Month 44	x					x
4	Diagnosis		x	x	x		x	x
	Progression 3*	Month 39	x	x	x		x	x
5	Progression 5	Month 83	x					x
	Progression 6	Month 99	x					x
7	Diagnosis		x					x
	Progression 1	Month 11	x					x
8	Progression 1	Month 25	x	x	x		x	x
	Progression 5	Month 34	x	x	x		x	x
9	Diagnosis		x	x	x		x	x
	Progression 3	Month 25	x	x	x		x	x
10	Diagnosis		x	x	x	x	x	x
	Progression 1	Month 16	x	x	x	x	x	x
13	Diagnosis		x					x
	Progression 5	Month 70	x					x
14	Diagnosis		x					x
	Progression 1	Month 15	x					x
	Progression 2	Month 39	x					x
	Progression 3	Month 47	x					x
15	Diagnosis		x	x	x	x	x	x
	Progression 1	Month 48	x	x		x	x	x
	Progression 2	Month 61	x	x	x		x	x
17	Diagnosis		x					x
	Progression 1	Month 7	x					x
18	Diagnosis		x	x	x	x	x	x
	Progression 1	Month 17	x	x		x	x	x
	Progression 2	Month 31	x	x	x		x	x
19	Diagnosis		x					x
	Progression 1	Month 35	x					x
20	Diagnosis		x	x	x		x	x
	Progression 6	Month 50	x	x	x		x	x
23	Diagnosis		x	x	x	x	x	x
	Progression 1	Month 25	x	x		x	x	x
	Progression 2	Month 63	x	x	x		x	x
24	Diagnosis		x	x	x		x	x
	Progression 2	Month 24	x	x	x		x	x
25	Diagnosis		x	x	x		x	x
	Progression 2	Month 16	x	x			x	x
	Progression 3	Month 39	x	x	x		x	x
26	Diagnosis		x	x	x	x	x	x
	Progression 1	Month 14	x	x		x	x	x
	Progression 2	Month 20	x	x	x		x	x
27	Diagnosis		x	x	x	x	x	x
	Progression 1	Month 17	x	x		x	x	x
	Progression 2	Month 19	x	x	x		x	x
28	Diagnosis		x	x	x	x	x	x
	Progression 1	Month 9	x	x	x	x	x	x
29	Diagnosis		x	x	x	x	x	x
	Progression 1	Month 5	x	x	x	x	x	x
30	Diagnosis		x	x	x	x	x	x
	Progression 1	Month 12	x	x	x	x	x	x
31	Diagnosis		x	x	x	x	x	x
	Progression 1	Month 14	x	x		x	x	x
	Progression 2	Month 19	x	x	x		x	x
32	Diagnosis		x	x	x	x	x	x
	Progression 1	Month 18	x	x	x	x	x	x
33	Diagnosis		x	x	x	x	x	x
	Progression 1	Month 18	x	x	x	x	x	x
34	Progression 2	Month 113	x	x	x		x	x
	Progression 3	Month 122	x	x			x	x
	Progression 4	Month 129	x	x			x	x
	Progression 6	Month 136	x	x	x		x	x
35	Progression 1	Month 48	x	x	x		x	x
	Progression 2	Month 72	x	x	x		x	x
36	Progression 2	Month 138	x	x	x		x	x
	Progression 5	Month 174	x	x	x		x	x
39	Diagnosis		x					x
	Progression 1	Month 15	x					x

B)

Patient_ID	MMRF_ID	Sample	Sample type	TFD	WES	RNAseq	DESeq (E-L)	DESeq (D-P1)	PI/ssGSEA	Clonality analysis
40	MMRF_1157	MMRF_1157_1_BM	Diagnosis		x	x	x	x	x	x
		MMRF_1157_2_BM	Progression	Month 21	x	x		x	x	x
		MMRF_1157_4_BM	Progression	Month 33	x	x			x	x
		MMRF_1157_5_BM	Progression	Month 54	x	x	x		x	x
41	MMRF_1179	MMRF_1179_1_BM	Diagnosis		x	x	x		x	x
		MMRF_1179_2_BM	Progression	Year 3	x	x	x		x	x
42	MMRF_1285	MMRF_1285_1_BM	Diagnosis		x	x	x	x	x	x
		MMRF_1285_3_BM	Progression	Month 30	x	x	x	x	x	x
43	MMRF_1433	MMRF_1433_1_BM	Diagnosis		x	x	x	x	x	x
		MMRF_1433_2_BM	Progression	Month 30	x	x		x	x	x
		MMRF_1433_5_BM	Progression	Month 51	x	x	x		x	x
44	MMRF_1534	MMRF_1534_1_BM	Diagnosis		x	x	x		x	x
		MMRF_1534_5_BM	Progression	Month 45	x	x	x		x	x
45	MMRF_1565	MMRF_1565_1_BM	Diagnosis		x	x	x	x	x	x
		MMRF_1565_3_BM	Progression	Year 3	x	x	x	x	x	x
46	MMRF_1587	MMRF_1587_1_BM	Diagnosis		x	x	x		x	x
		MMRF_1587_2_BM	Progression	Month 18	x	x	x		x	x
47	MMRF_1627	MMRF_1627_1_BM	Diagnosis		x	x	x	x	x	x
		MMRF_1627_3_BM	Progression	Month 21	x	x	x	x	x	x
48	MMRF_1639	MMRF_1639_1_BM	Diagnosis		x	x	x	x	x	x
		MMRF_1639_3_BM	Progression	Month 18	x	x	x	x	x	x
49	MMRF_1656	MMRF_1656_1_BM	Diagnosis		x	x	x		x	x
		MMRF_1656_4_BM	Progression	Month 18	x	x	x		x	x
50	MMRF_1671	MMRF_1671_1_BM	Diagnosis		x	x	x		x	x
		MMRF_1671_5_BM	Progression	Year 3	x	x	x		x	x
51	MMRF_1700	MMRF_1700_1_BM	Diagnosis		x	x	x		x	x
		MMRF_1700_2_BM	Progression	Month 18	x	x	x		x	x
52	MMRF_1739	MMRF_1739_1_BM	Diagnosis		x	x	x		x	x
		MMRF_1739_2_BM	Progression	Month 39	x	x	x		x	x
53	MMRF_1795	MMRF_1795_1_BM	Diagnosis		x	x	x	x	x	x
		MMRF_1795_2_BM	Progression	Month 42	x	x	x	x	x	x
54	MMRF_1908	MMRF_1908_1_BM	Diagnosis		x	x	x	x	x	x
		MMRF_1908_3_BM	Progression	Month 33	x	x	x	x	x	x
55	MMRF_1931	MMRF_1931_1_BM	Diagnosis		x	x	x	x	x	x
		MMRF_1931_2_BM	Progression	Month 18	x	x	x	x	x	x
56	MMRF_1992	MMRF_1992_1_BM	Diagnosis		x	x	x	x	x	x
		MMRF_1992_2_BM	Progression	Month 15	x	x	x	x	x	x
57	MMRF_2059	MMRF_2059_1_BM	Diagnosis		x	x	x	x	x	x
		MMRF_2059_2_BM	Progression	Month 27	x	x	x	x	x	x
58	MMRF_2089	MMRF_2089_1_BM	Diagnosis		x	x	x	x	x	x
		MMRF_2089_2_BM	Progression	Month 9	x	x	x	x	x	x
59	MMRF_2122	MMRF_2122_1_BM	Diagnosis		x	x	x		x	x
		MMRF_2122_3_BM	Progression	Month 18	x	x	x		x	x
60	MMRF_2126	MMRF_2126_1_BM	Diagnosis		x	x	x		x	x
		MMRF_2126_2_BM	Progression	Month 21	x	x	x		x	x
61	MMRF_2194	MMRF_2194_1_BM	Diagnosis		x	x	x	x	x	x
		MMRF_2194_2_BM	Progression	Month 15	x	x	x	x	x	x
62	MMRF_2328	MMRF_2328_1_BM	Diagnosis		x	x	x		x	x
		MMRF_2328_2_BM	Progression	Month 27	x	x	x		x	x
63	MMRF_2373	MMRF_2373_1_BM	Diagnosis		x	x	x		x	x
		MMRF_2373_2_BM	Progression	Month 15	x	x	x		x	x
64	MMRF_2401	MMRF_2401_1_BM	Diagnosis		x	x	x	x	x	x
		MMRF_2401_2_BM	Progression	Month 3	x	x	x	x	x	x
65	MMRF_2523	MMRF_2523_1_BM	Diagnosis		x	x	x	x	x	x
		MMRF_2523_2_BM	Progression	Year 2	x	x	x	x	x	x
66	MMRF_2562	MMRF_2562_1_BM	Diagnosis		x	x	x		x	x
		MMRF_2562_2_BM	Progression	Month 21	x	x	x		x	x
67	MMRF_2601	MMRF_2601_1_BM	Diagnosis		x	x	x		x	x
		MMRF_2601_2_BM	Progression	Month 18	x	x	x		x	x
