# Supplementary material for: Clonal evolution after treatment pressure in multiple myeloma: heterogenous genomic aberrations and transcriptomic convergence
Source: Leukemia. 2022 May 28;36(7):1887–97. doi: 10.1038/s41375-022-01597-y (PMC9252918; doi:10.1038/s41375-022-01597-y)
Supplement: Supplementary file 7 — Table S2 [file 41375_2022_1597_MOESM7_ESM.pdf]

Table S2. CN Purity and Ploidy were performed using both Facets and Sequenza for all samples. Facets values are shown in the table and used in the dpClust analysis, exept *Sequenza values used (due to either sample failed or untrustable results in Facets). #not included in RNAseq analysis. *RNAseq not performed. Raw Counts files downloaded from MMRF Researcher gateway. PF: reads passing filter. M : Million

Patient_ID	Sample	WES	RNAseq	Morphology/Flow* Purity stain	Facets/Sequenza* Purity CN	Facets/Sequenca* Ploidy	WES Total reads (M)	WES PF reads aligned (M)	WES Median depth on target	WES - Normal (germline sample) Median depth on target Normal	RNAseq Reads (M)	RNAseq % PF	RNAseq % Duplicat	RNAseq % Aligned	RNAseq Aligned (M)
3	PD 1	x		92	0.79	2.01	139.8	139.7	184	83	#				
	PD 2	x		88	0.92	2	193.6	193.5	255	83	#				
4	Diagnosis	x	x	95	0.98	1.97	166.4	166.4	222	91	33.9	91.90 %	29.80 %	94.60 %	14.7
	PD 3*	x	x	84	0.85	1.94	142.5	142.4	194	91	38.1	91.50 %	22.20 %	95.20 %	16.6
5	PD 5	x		100	0.69	2.21	149.3	149.2	203	97	#				
	PD 6	x		88	0.7	2.18	165.2	165.1	226	97	#				
7	Diagnosis	x		98	0.85	1.95	157.5	157.5	208	49	#				
	PD 1	x		98	0.89	1.95	148.2	148.2	192	49	#				
8	PD 1	x	x	98	0.3	0.97	148.9	148.8	205	68	30.7	92.40 %	46.10 %	96.80 %	13.7
	PD 5	x	x	95	0.3	1.94	157.3	157.3	214	68	39.3	93.20 %	51.00 %	96.80 %	17.7
9	Diagnosis	x	x	98	0.93	1.98	144.4	144.4	193	62	103.2	88.40 %	78.10 %	96.90 %	44.2
	PD 3	x	x	98	0.77	2.14	203.4	203.3	333	62	160.0	73.80 %	86.40 %	96.90 %	57.3
10	Diagnosis	x	x	98	0.88	2	164.1	164.1	224	83	26.3	92.20 %	30.90 %	93.20 %	11.3
	PD 1	x	x	98	0.9	1.98	150.3	150.3	193	83	32.7	91.20 %	34.20 %	93.40 %	13.9
13	Diagnosis	x		98	0.94*	3.1*	167.6	167.5	216	65	#				
	PD 5	x		95	0.5*	3*	180.4	180.3	234	65	#				
14	Diagnosis	x		100	0.87	2.17	162.4	162.4	220	87	#				
	PD 1	x		98	0.92	2.17	183.0	182.9	245	87	#				
	PD 2	x		97	0.84	2.24	138.5	138.5	195	87	#				
	PD 3	x		*NA*	0.8	2.49	92.1	92.1	150	87	#				
15	Diagnosis	x	x	97	0.95	0.95	133.3	133.2	181	73	16.4	90.40 %	21.20 %	94.80 %	7.0
	PD 1	x	x	91	0.91	0.91	178.2	178.2	240	73	29.5	90.00 %	34.20 %	94.00 %	12.5
	PD 2	x	x	91	0.92	0.92	221.6	221.5	312	73	25.9	89.30 %	31.90 %	94.10 %	10.9
17	Diagnosis	x		86	0.74	1.95	185.5	185.5	257	96	#				
	PD 1	x		80	0.64	1.95	184.7	184.7	253	96	#				
18	Diagnosis	x	x	100	0.9	2.23	207.2	207.2	263	74	147.5	85.40 %	74.70 %	94.70 %	59.6
	PD 1	x	x	100	0.89	2.21	232.6	232.5	317	74	121.8	94.00 %	62.70 %	95.20 %	54.5
	PD 2	x	x	94	0.96	2.37	145.5	145.4	200	74	118.7	93.40 %	53.50 %	96.70 %	53.6
19	Diagnosis	x		100	0.95	2.45	217.9	217.9	275	103	#				
	PD 1	x		98	0.97	2.27	193.5	193.5	242	103	#				
20	Diagnosis	x	x	90	0.88	2.06	212.3	212.3	276	78	21.6	92.80 %	30.20 %	95.40 %	9.6
	PD 6	x	x	90	0.98	2.04	219.1	219.0	286	78	29.6	91.40 %	20.50 %	96.60 %	13.1
23	Diagnosis	x	x	91*	0.82	2.5	95.1	95.1	120	43	143.8	88.90 %	78.60 %	92.60 %	59.2
	PD 1	x	x	92*	0.77	2.44	110.9	110.8	138	43	157.9	86.00 %	73.20 %	89.90 %	61.0
	PD 2	x	x	*NA*	0.87	2.64	136.0	136.0	207	43	154.9	78.40 %	90.40 %	93.00 %	56.5
24	Diagnosis	x	x	98*	0.92	2.1	89.8	89.8	117	44	153.1	83.30 %	80.70 %	95.00 %	60.5
	PD 2	x	x	92*	0.68	2.15	110.4	110.3	146	44	130.3	92.90 %	74.00 %	96.70 %	58.5
25	Diagnosis	x	x	96*	0.87	2.48	144.9	144.9	184	42	143.6	83.60 %	79.60 %	94.00 %	56.4
	PD 2	x	x	94*	0.83	2.49	155.3	155.2	186	42	144.1	86.80 %	76.30 %	96.40 %	60.3
	PD 3	x	x	>80*	0.7	2.63	161.0	161.0	265	42	120.2	91.20 %	74.80 %	96.00 %	52.6
26	Diagnosis	x	x	99*	0.97*	2.3*	124.1	124.1	160	40	140.7	94.80 %	69.20 %	92.10 %	61.4
	PD 1	x	x	99*	0.98*	2.2*	132.5	132.5	166	40	173.4	89.20 %	73.60 %	93.40 %	72.2
	PD 2	x	x	98*	1*	2.5*	127.6	127.6	230	40	145.9	90.80 %	67.10 %	95.40 %	63.2
27	Diagnosis	x	x	93*	0.91	2.06	122.5	122.5	150	45	147.9	89.30 %	71.80 %	95.60 %	63.1
	PD 1	x	x	100*	0.96	2.12	129.7	129.6	160	45	152.1	94.20 %	58.90 %	98.50 %	70.6
	PD 2	x	x	99*	0.98	2.23	146.9	146.9	258	45	153.7	92.90 %	61.20 %	98.40 %	70.3
28	Diagnosis	x	x	92*	0.94	2.67	151.7	151.6	187	47	140.7	86.90 %	69.40 %	90.30 %	55.2
	PD 1	x	x	97	0.83	2.74	131.8	131.8	164	47	0.17	82.40 %	8.30 %	92.30 %	0.07
29	Diagnosis	x	x	97*	0.94	2.37	116.6	116.5	147	48	138.5	82.90 %	73.10 %	95.90 %	55.1
	PD 1	x	x	97*	0.91	2.41	145.1	145.1	183	48	134.7	82.80 %	83.70 %	94.00 %	52.4
30	Diagnosis	x	x	94*	0.95	2.31	117.9	117.8	156	35	149.3	80.00 %	84.80 %	97.60 %	58.3
	PD 1	x	x	96*	0.57	2.32	141.8	141.8	183	35	163.1	85.30 %	84.10 %	97.70 %	67.9
31	Diagnosis	x	x	84*	0.92	2.3	94.2	94.2	147	39	175.4	89.60 %	86.80 %	90.70 %	71.3
	PD 1	x	x	86*	0.84	2.12	90.0	89.9	115	39	102.5	90.10 %	71.30 %	94.20 %	43.5
	PD 2	x	x	80*	0.5	2.07	143.4	143.4	187	39	113.7	85.70 %	69.10 %	94.90 %	46.3
32	Diagnosis	x	x	93*	0.95	2.22	118.0	117.9	153	44	136.6	93.00 %	78.60 %	94.80 %	60.3
	PD 1	x	x	99*	0.97	2.23	124.9	124.9	162	44	151.6	88.40 %	82.00 %	95.70 %	64.2
33	Diagnosis	x	x	96*	0.96	1.97	98.7	98.7	161	74	195.9	68.60 %	91.70 %	97.80 %	65.7
	PD 1	x	x	92*	0.98	1.94	159.1	159.1	273	74	74.1	83.20 %	72.20 %	97.80 %	30.2
34	PD 2	x	x	90	0.86	2.48	198.7	198.7	279	52	132.6	71.40 %	85.60 %	97.50 %	46.2
	PD 3	x	x	85	0.66	4.92	139.2	139.2	227	52	110.8	81.20 %	75.50 %	97.60 %	43.9
	PD 4	x	x	85	0.87	2.51	79.3	79.3	119	52	101.8	86.20 %	72.30 %	98.20 %	43.1
	PD 6	x	x	85	0.92	2.53	124.0	124.0	205	52	112.1	87.00 %	75.40 %	98.30 %	47.9
35	PD 1	x	x	*NA*	0.8	2.19	111.4	111.4	198	71	109.4	83.10 %	85.20 %	93.80 %	42.7
	PD 2	x	x	80	0.72	2.32	241.7	241.7	333	71	161.8	32.90 %	87.70 %	95.10 %	25.3
36	PD 2	x	x	*NA*	0.79	2.31	149.3	149.3	195	99	105.3	86.30 %	74.70 %	91.40 %	41.5
	PD 5	x	x	*NA*	0.78	2.25	155.1	155.1	281	99	109.9	75.70 %	82.50 %	93.00 %	38.7
39	Diagnosis	x		*NA*	0.77	2.24	130.2	130.2	233	93	#				
	PD 1	x		80	0.55	2.43	110.2	110.2	155	93	#				
40	MMRF_1157_1_BM			*NA*	0.98	1.94	138.0	137.9	95	70	*				
	MMRF_1157_2_BM			*NA*	0.97	1.95	124.0	123.9	77	70	*				
	MMRF_1157_4_BM			*NA*	0.98	1.98	115.5	115.4	74	70	*				
	MMRF_1157_5_BM			*NA*	0.97	1.96	115.1	115.0	81	70	*				
41	MMRF_1179_1_BM			*NA*	0.97	1.92	136.1	136.0	85	88	*				
	MMRF_1179_2_BM			*NA*	0.97	1.97	113.8	113.8	72	88	*				
42	MMRF_1285_1_BM			*NA*	0.91	2.27	117.6	117.6	86	90	*				
	MMRF_1285_3_BM			*NA*	0.91	2.31	93.9	93.9	73	90	*				
43	MMRF_1433_1_BM			*NA*	0.97	2.04	56.7	56.7	44	80	*				
	MMRF_1433_2_BM			*NA*	0.82	2.04	101.1	101.1	68	80	*				
	MMRF_1433_5_BM			*NA*	0.97	1.99	98.9	98.9	75	80	*				
44	MMRF_1534_1_BM			*NA*	0.92	2.09	68.8	68.8	54	63	*				
	MMRF_1534_5_BM			*NA*	0.92	2.06	101.3	101.3	78	63	*				
45	MMRF_1565_1_BM			*NA*	0.94	2.06	120.7	120.6	78	63	*				
	MMRF_1565_3_BM			*NA*	0.86	2.1	117.3	117.2	84	63	*				
46	MMRF_1587_1_BM			*NA*	0.98	1.92	116.2	116.2	89	97	*				
	MMRF_1587_2_BM			*NA*	0.95	1.96	108.7	108.7	79	97	*				
47	MMRF_1627_1_BM			*NA*	0.97	2.03	127.9	127.8	78	76	*				
	MMRF_1627_3_BM			*NA*	0.95	2.04	112.3	112.2	72	76	*				
48	MMRF_1639_1_BM			*NA*	0.96	2.45	67.8	67.8	43	69	*				
	MMRF_1639_3_BM			*NA*	0.95	2.55	100.6	100.6	74	69	*				
49	MMRF_1656_1_BM			*NA*	0.89	2.26	82.9	82.8	59	59	*				
	MMRF_1656_4_BM			*NA*	0.95	2.21	113.4	113.3	85	59	*				
50	MMRF_1671_1_BM			*NA*	0.95	2.14	115.7	115.7	89	100	*				
	MMRF_1671_5_BM			*NA*	0.87	2.09	117.1	117.1	83	100	*				
51	MMRF_1700_1_BM			*NA*	0.91	2.26	98.5	98.4	61	72	*				
	MMRF_1700_2_BM			*NA*	0.84	2.36	102.8	102.8	79	72	*				
52	MMRF_1739_1_BM			*NA*	0.97*	2.5*	132.8	132.8	103	114	*				
	MMRF_1739_2_BM			*NA*	0.93*	2.4*	101.6	101.6	74	114	*				
53	MMRF_1795_1_BM			*NA*	0.97	2.63	109.3	109.3	67	72	*				
	MMRF_1795_2_BM			*NA*	0.93	2.60	99.8	99.8	73	72	*				
54	MMRF_1908_1_BM			*NA*	0.91	1.84	116.7	116.7	68	62	*				
	MMRF_1908_3_BM			*NA*	0.98	1.94	127.0	127.0	85	62	*				
55	MMRF_1931_1_BM			*NA*	0.83	2.23	81.2	81.1	50	50	*				
	MMRF_1931_2_BM			*NA*	0.86	2.16	118.5	118.5	81	50	*				
56	MMRF_1992_1_BM			*NA*	0.94*	2.2*	116.8	116.7	75	67	*				
	MMRF_1992_2_BM			*NA*	0.93*	2.2*	110.5	110.3	74	67	*				
57	MMRF_2059_1_BM			*NA*	0.88	2.03	99.6	99.5	63	72	*				
	MMRF_2059_2_BM			*NA*	0.95	2.47	119.9	119.9	84	72	*				
58	MMRF_2089_1_BM			*NA*	0.75	2.81	96.5	96.4	60	67	*				
	MMRF_2089_2_BM			*NA*	0.86	2.6	110.5	110.5	69	67	*				
59	MMRF_2122_1_BM			*NA*	0.96	1.92	109.9	109.8	68	54	*				
	MMRF_2122_3_BM			*NA*	0.91	1.89	115.7	115.7	79	54	*				
60	MMRF_2126_1_BM			*NA*	0.95	2.42	101.6	101.5	62	159	*				
	MMRF_2126_2_BM			*NA*	0.97	2.44	97.3	97.3	64	159	*				
61	MMRF_2194_1_BM			*NA*	0.84	1.93	96.6	96.6	61	62	*				
	MMRF_2194_2_BM			*NA*	0.75	1.95	111.7	111.7	76	62	*				
62	MMRF_2328_1_BM			*NA*	0.8	2.17	140.7	140.7	87	80	*				
	MMRF_2328_2_BM			*NA*	0.9	2.06	100.5	100.4	75	80	*				
63	MMRF_2373_1_BM			*NA*	0.96	2.06	109.8	109.7	68	73	*				
	MMRF_2373_2_BM			*NA*	0.98	2.01	139.2	139.1	93	73	*				
64	MMRF_2401_1_BM			*NA*	0.89	1.96	133.4	133.3	86	79	*				
	MMRF_2401_2_BM			*NA*	0.87	2	115.6	115.6	73	79	*				
65	MMRF_2523_1_BM			*NA*	0.98	2.35	103.6	103.6	79	88	*				
	MMRF_2523_2_BM			*NA*	0.96	2.29	105.3	105.3	73	88	*				
66	MMRF_2562_1_BM			*NA*	0.93	2.17	94.7	94.7	62	69	*				
	MMRF_2562_2_BM			*NA*	0.87	3.76	121.5	121.4	83	69	*				
67	MMRF_2601_1_BM			*NA*	0.9	2.26	98.9	98.9	63	69	*				
	MMRF_2601_2_BM			*NA*	0.92	2.18	127.4	127.3	85	69	*				
