# Supplementary material for: Clonal evolution after treatment pressure in multiple myeloma: heterogenous genomic aberrations and transcriptomic convergence
Source: Leukemia. 2022 May 28;36(7):1887–97. doi: 10.1038/s41375-022-01597-y (PMC9252918; doi:10.1038/s41375-022-01597-y)
Supplement: Supplementary file 8 — Table S3A [file 41375_2022_1597_MOESM8_ESM.pdf]

Table S3. A) Clinical data for the patients included ifrom St.Olavs hospital and Rotterdam (in-house cohort).

S: Sample, TSD: Time since diagnosis. D = Diagnosis = diagnosis symptomatic MM (before first treatment). P = progression

Abnormalities FISH: * = tested for t(4;14) and del17 only. ** = tested for t(4;14), del17 and del13. *** = tested for t(4;14), del17, del13, t(11;14). $ = tested for del13, del17p, t(4;14), t(11;14), t(14;16). nd = not detected

Patient	Patient age	Sex	Ig class	light chain	Abnormalities FISH	ISS	R-ISS	Response initial treatment S1 - TSD		Treatment prior to S1	IMID received S1-2	PI received S1-2	HDM received S1-2	Intervening treatment* S1-S2	Best response S1-S2	Interval S1-S2 (months)	IMID received S2-3	PI received S2-3	HDM received S2-3	Intervening treatment S2-S3	Best response S2-S3	Interval S2-S3 (months)	Intervening treatment S3-S4	Best response S3-S4	Intervening treatment S4-S5	PFS, months	death occured (y=1)	OS, months
17	84	Male	IgA	Lambda	del17p*	3	3	PR	0	0	x			MPR	PR	7										6	1	8
19	66	Female	IgA	Kappa	nd *	3		VGPR	0	0		x	x	VCD/HDM	VGPR	35					VGPR					32	1	40
15	67	Male	IgA	Kappa	t(4;14)*	3	3	VGPR	0	0	x			MPR / R maint	VGPR	48		x		VCD	VGPR	13		PR		45	1	94
9	73	Female	IgG	Kappa	nd*	1	1	PR	0	0	x	x		MPT,VCD	PR	25					PR					27	0	71
14	77	Female	IgG	Kappa	del13**	2	2	VGPR	0	0				MP	VGPR	15		x		MPV	VGPR	24	CVD	PR		14	1	64
10	76	Female	IgA	Kappa	t(4;14)*	2		PR	0	0	x			MPR	PR	16					SD					11	1	34
18	68	Male	IgG	Kappa	nd*	1	1	VGPR	0	0		x		VCD	VGPR	17	x			RP, MPV	SD					16	1	31
7	78	Female		Lambda	del17p*	3	3	PR	0	0		x		CVD	PR	11										6	1	21
31	67	Male	IgG	Lambda	t(4;14)$	3	3	VGPR	0	0	x			MPT/T maint	VGPR	14		x		VRD	VGPR	5				12	1	19
20	54	Female	IgG	Kappa	t(11;14) ***	2	3	VGPR	0	0	x	x	x	CVD/HDM,MPV,TP,RP,CP	cCR	50					PR					13	1	52
4	52	Female	IgG	Lambda	t(11;14), del17 (21%), del13***	2	2	PR	0	0		x	x	VCD/HDM, MPV	PR	39										21	1	44
32	71	Female	IgG	Kappa	del13q$	2	2	VGPR	0	0	x			MPT/T maint	VGPR	18										18	1	18
13	65	Male	IgD	Lambda	nd**	3	2	VGPR	0	0	x	x	x	CD/HDM, MPV, CVD, MPT, RP	CR	70					VGPR					34	1	71
39	60	Male	IgG	Kappa	t(4;14)	2	2	VGPR	0	0		x	x	VCD/HDM	VGPR	15										10	1	25
23	58	Male	IgA	Kappa	nd$	1	1	VGPR	0	0	x	x		VCD/VMP/R maint	VGPR	25						38				25	1	76
24	62	Female	IgG	Kappa	t(11:14)$	3	2	PR	0	0	x	x		VCD,RD,cyclo, MPT	<PR	24					SD					2	1	34
25	49	Male	IgG	Kappa	nd$	2	2	PR	0	0	x	x	x	VCD, RD, VCD/HDM	PR	16		x		VTD		23				9	1	66
26	51	Male	IgA	Lambda	nd$	3	2	CR	0	0	x	x	x	VCD/HDM/R maint	CR	14						6				13	1	18
27	51	Male	IgG	Kappa	t(4;14)$	3	3	VGPR	0	0	x	x	x	VCD/HDM/VRD/R maint	VGPR	17						2				17	1	19
28	56	Male	IgG	Kappa	nd$	2	2	VGPR	0	0	x	x	x	VCD/HDM/VRD/R maint	VGPR	9										9	1	27
29	42	Male	IgG	Kappa	nd$	2	2	<PR	0	0		x		VCD/VMP	<PR	5										5	1	25
30	62	Female	IgG	Kappa	nd$	2	2	PR	0	0		x		VCD/VMP	PR	12										11	0	59
33	64	Female	IgG	Kappa	t(11;14), del13q$	2	2	<PR	0	0	x	x	x	VCD/HDM/R maint	<PR	18										16	1	39
3	48	Female	IgA	Kappa	del17*			CR	29	VD/HDM	x		x	VCD/HDM	CR	15					CR					26	1	111
8	50	Male	IgG	Lambda	nd*	1		PR	25	VCD/HDM	x	x		RD,VCD,pom,TD	SD	9										10	1	38
35	76	Female	IgG	Kappa	del13**	2		SD	48	MPR		x		MPV	VGPR	24										14	1	88
34	48	Male	IgG	Lambda	nd *	2	2	CR	113	CD/HDM,VCD/HDM	x			CTP	PR	9	x			RD	VGPR	7	VD	PR	dara	113	1	154
36	51	Male	IgG	Kappa	nd *			CR	138	vAD/HDM,CD/HDM	x	x		VCD,RD,RD	PR	36										60	1	245
5	61	Female	IgG	Lambda	nd *	2	2	VGPR	83	CD/HDM,MPT,VD,VMD		x		VD	PR	16										18	1	107

C: cyclophosphamide
D:dexamethasone
P:prednisone
V:velcade/bortezomib.
R:revlimid/lenalidomide
T:thalidomide
pom:Imnovid/pomalidomide
v:vincristin
A:Adriamycine
HDM:high dose melphalan (ASCT)
benda:bendamustin
