# Supplementary material for: Clonal evolution after treatment pressure in multiple myeloma: heterogenous genomic aberrations and transcriptomic convergence
Source: Leukemia. 2022 May 28;36(7):1887–97. doi: 10.1038/s41375-022-01597-y (PMC9252918; doi:10.1038/s41375-022-01597-y)
Supplement: Supplementary file 9 — Table S3B [file 41375_2022_1597_MOESM9_ESM.pdf]

Table S3. B) Clinical data for the patients included from the IA13 CoMMpass study. FISHseq: Canonical Translocations identified by long-insert Whole Genome Sequencing. S:Sample, nd: not detected. NA: no data. Survival data was updated using the IA16 CoMMpass version.

Patient_ID	Patient Age	Sex	Ig class	light chain	FISHseq	CNV	ISS	R-ISS	IMID received S1-2	PI received S1-2	HDM received S1-2	Intervening treatment* S1-S2	Best response S1-S2	Interval S1-S2 (months)	Treatment S2-S3	Best response	Interval S2-S3	Treatment S3	Response	Interval S3-S4	Treatment S4	Response	PFS, months	death occured (y=1)	OS/Last follow-up (months)
40	69	Female	Unknown	Kappa	t(11;14)		1		x	x	x	VCD/VRCD/VCD/HDM/R/RD	VGPR	21	CarfIbu/CarfIbuD/CarfD	VGPR	12	PomDara	VGPR	21	SelD	VGPR	18	0	72
41	80	Male	IgA	Kappa	t(4;14)	del13q	2		x	x		VRD/VRCD/Carf/VRD/CarfT	VGPR	36	Dara	SD							18	1	65
42	75	Male	IgG	Kappa	nd	del13q	1			x		VCD/V	VGPR	30	VRD	VGPR							26	1	67
43	55	Male	IgG	Lambda	t(4;14)	del13q	1	2	x	x	x	VRD/HDM/HDM	VGPR	30	CarfRD/PomD/RPembD	PR	21	DIxaIDA	SD				29	1	72
44	64	Male	IgG	Kappa	t(11;14)		1	1	x	x	x	VRD/HDM/R/VenD	VGPR	45	Dara	PR							5	0	71
45	39	Female	Unknown	Kappa	t(11;14)		1	1		x	x	VRD/HDM/R/VenD	sCR	36	CarfRD	VGPR							37	0	43
46	74	Male	Unknown	Kappa	*NA*		3	2	x	x		VCD/VD/VCD/VRD	PR	18		*NA*							7	1	21
47	82	Female	Unknown	Lambda	t(4;14)		3	3	x	x		VD/R/RD/R	VGPR	21	R	VGPR							19	1	52
48	67	Female	Unknown	Kappa	nd	del13q	3	2	x	x		VD/R	VGPR	18	Pom	*NA*							17	1	23
49	70	Female	IgA	Lambda	nd		3	2	x	x	x	HDM/VRD/VCD	VGPR	18		*NA*							10	1	17
50	68	Male	IgG	Kappa	t(11;14)		1		x	x		VCD/R/CarfD/CarfPomD	PR	36	CarfPomD/Carf	SD							2	0	65
51	75	Male	IgG	Kappa	nd	del13q	3	2	x	x		VRD/VRCD/Carf/Pom	PR	18		*NA*							9	1	19
52	45	Female	IgG	Lambda	nd		3	2	x	x	x	VCD/VRCD/HDM/RD	CR	39	Dara/CarfPomD	PR							36	0	66
53	75	Female	IgG	Lambda	nd		3	2		x		CarfMPD	sCR	42		*NA*							42	0	67
54	68	Female			t(6;14)	del13q	1	2	x		x	HDM/T	CR	33		*NA*							32	0	45
55	72	Female			nd		3	2		x		VMP	VGPR	18	RD	VGPR							17	0	59
56	53	Female	IgG	Kappa	t(11;14)		3	2	x	x	x	HDM	PR	15	RPanoD	PR							15	0	51
57	57	Male	IgG	Lambda	t(12;14)		3	2	x	x	x	HDM	sCR	27	CarfPomD	VGPR							26	0	63
58	66	Female			nd	del13q	3	2		x		VMP	VGPR	9	RD	PD							8	1	15
59	67	Female	IgA	Lambda	t(4;14)	del13q	2	2	x	x		VRD	VGPR	18	IxaRD	VGPR							11	0	62
60	70	Male	IgG	Kappa	t(14;20)		2		x	x		VRD/RD	PR	21		*NA*							11	1	19
61	75	Male	Unknown	Kappa	t(11;14)	del17p	2	2		x		VMP	VGPR	15	RD	SD							15	1	36
62	66	Male	IgG	Kappa	t(4;14)	del13q	3	3	x	x		VCD/VD/RD	VGPR	27	RD	PD							21	1	33
63	67	Male	Unknown	Kappa	t(8;14)		1	2	x	x	x	HDM	PD	15	CarfRD	PD							3	1	18
64	64	Male			t(4;14)	del13q	3	3	x	x		VTD	PD	3	VCD	SD							3	1	13
65	55	Female	IgA	Lambda	nd		3		x	x		VD/VRD	VGPR	24	VD	*NA*							19	1	22
66	66	Male	Unknown	Kappa	nd	del13q	2	2	x	x		VRD/CarfD	PR	21		*NA*							7	1	40
67	70	Female			nd	del13q	2	2		x		VMP	VGPR	18	RD	PR							19	0	50

*Treatment:
C: cyclophosphamide
D:dexamethasone
P:prednisone
V:velcade/bortezomib.
R:revlimid/lenalidomide
T:thalidomide
Pom:Imnovid/pomalidomide
HDM:high dose melphalan (ASCT)
Carf: carfilzomib
Pano: panobinostat
Pemb: pembrolizumab
Sel: selinexor
IDA: IDASANUTLIN-D
Ven: venetoclax
Dara: daratumumab
