# Supplementary material for: Clonal evolution after treatment pressure in multiple myeloma: heterogenous genomic aberrations and transcriptomic convergence
Source: Leukemia. 2022 May 28;36(7):1887–97. doi: 10.1038/s41375-022-01597-y (PMC9252918; doi:10.1038/s41375-022-01597-y)
Supplement: Supplementary file 10 — Table S4 [file 41375_2022_1597_MOESM10_ESM.pdf]

Table S4. Driver genes according to references*

Gene	Category
MAX	Driver gene
TP53	Driver gene
FAM46C	Driver gene
DIS3	Driver gene
BRAF	Driver gene
KRAS	Driver gene
NRAS	Driver gene
TRAF3	Driver gene
DUSP2	Driver gene
TCL1A	Driver gene
TRAF2	Driver gene
CYLD	Driver gene
LTB	Driver gene
HIST1H1E	Driver gene
BCL7A	Driver gene
SP140	Driver gene
NFKBIA	Driver gene
EGR1	Driver gene
PABPC1	Driver gene
PRKD2	Driver gene
TBC1D29	Driver gene
IRF4	Driver gene
RB1	Driver gene
TGDS	Driver gene
PTPN11	Driver gene
FUBP1	Driver gene
RPL5	Driver gene
FGFR3	Driver gene
SAMHD1	Driver gene
ACTG1	Driver gene
HIST1H1B	Driver gene
NFKB2	Driver gene
KMT2B	Driver gene
KLHL6	Driver gene
RASA2	Driver gene
PIM1	Driver gene
PRDM1	Driver gene
DTX1	Driver gene
SETD2	Driver gene
BHLHE41	Driver gene
RPL10	Driver gene
BTG1	Driver gene
RPS3A	Driver gene
CCND1	Driver gene
RPRD1B	Driver gene
HIST1H1D	Driver gene
ZNF292	Driver gene
RFTN1	Driver gene
CDKN1B	Driver gene
LCE1D	Driver gene
XBP1	Driver gene
IRF1	Driver gene
HIST1H2BK	Driver gene
POT1	Driver gene
HUWE1	Driver gene
UBR5	Driver gene
USP7	Driver gene
MAN2C1	Driver gene
MAML2	Driver gene
ABCF1	Driver gene
CDKN2C	Driver gene
ATM	Driver gene
KMT2C	Driver gene
CREBBP	Driver gene
ARID1A	Driver gene
ATRX	Driver gene
NF1	Driver gene
EP300	Driver gene
TET2	Driver gene
KDM5C	Driver gene
ARID2	Driver gene
DNMT3A	Driver gene
KDM6A	Driver gene
SF3B1	Driver gene
MAF	Driver gene
NCOR1	Driver gene
MAFB	Driver gene
IDH1	Driver gene
IDH2	Driver gene
PIK3CA	Driver gene

*Driver gene: Identified myeloma driver gene (Maura et al, Nature Communications, 2019; Waker B et al, Blood, 2018) [4,5]
