# Supplementary material for: Clonal evolution after treatment pressure in multiple myeloma: heterogenous genomic aberrations and transcriptomic convergence
Source: Leukemia. 2022 May 28;36(7):1887–97. doi: 10.1038/s41375-022-01597-y (PMC9252918; doi:10.1038/s41375-022-01597-y)
Supplement: Supplementary file 11 — Table S5 [file 41375_2022_1597_MOESM11_ESM.pdf]

Table S5. Evolution patterns, SNVs and CNA in all samples

Patient_ID	Ig-T/HRD	Sample1	Sample2	Sample 3	Sample 4	Evolution pattern S1 - S2	Evolution pattern S2 - S3	Evolution pattern S3 - S4	Mutations/CNA# at Sample 1	Mutations/CNA# at Sample 2	Mutations#/CNA at Sample 3	Mutations/CNA# at Sample 4
31	t(4;14)	Diagnosis	Relapse 1	Relapse 2		Differential Clonal Respon	Differential Clonal Response		PRKD2, NRAS G12D(sub) / gain 1q21	PRKD2, NRAS G12D /amp1q21	PRKD2, NRAS G12D / amp1q21	
23	HRD	Diagnosis	Relapse 1	Relapse 2		Differential Clonal Respon	Differential Clonal Response		BRAF(sub) / amp1q21	BRAF(sub), PTPN11*, KRAS Q61H(sub) / amp1q21	PTPN11(sub), KRAS Q61H(sub) / gain 1q21	
26	HRD	Diagnosis	Relapse 1	Relapse 2		Differential Clonal Respon	Differential Clonal Response		ATM, NCOR1, NRAS Q61K(sub) / gain 1q21	ATM, NCOR1 / gain 1q21	ATM, NCOR1, KRAS D119H(sub) / amp1q21	
19	unknown	Diagnosis	Relapse 1			Differential Clonal Response			NRAS Q61K	NRAS Q61K/amp1q21		
20	t(11;14)	Diagnosis	Relapse 6			Differential Clonal Response			PRKD2, KRAS A146P, FAM46C / gain 1q21	PRKD2, KRAS A146P, NCOR1(sub), TP53(sub), IRF4* / amp1q21		
4	t(11;14)	Diagnosis	Relapse 3			Differential Clonal Response			POT1 / amp1q21, del1p22	*CAND1* / gain1q21, del1p22		
32	HRD	Diagnosis	Relapse 1			Differential Clonal Response			BRAF, KMT2C(InDel), RPL10(sub), UBR5(InDel, sub) / gain 1q21	BRAF, KMT2C(InDel), RPL10, RB1(sp), FAM46C(InDel, sub) / gain 1q21		
13	unknown	Diagnosis	Relapse 5			Differential Clonal Response			DUSP2, NRAS Q61R, TET2(sp) / gain 1q21	DUSP2, NRAS Q61R, TET2(sp), IRF4, *PSMD1 (InDel,sub)* / amp1q21		
39	t(4;14)	Diagnosis	Relapse 1			Differential Clonal Response			KRAS A146V, HUWE1(InDel, sub)	KRAS A146V, IRF4* / gain 1q21		
24	t(11;14)	Diagnosis	Relapse 2			Differential Clonal Response			*GSPT1*, UBR5(sp),SF3B1, KRAS Q61H, KRAS G13C (sub)	*GSPT1*, UBR5(sp,sub), SF3B1(sub), KRAS Q61H(sub), KRAS G13C, DIS3(sub)*		
28	HRD	Diagnosis	Relapse 1			Differential Clonal Response			KRAS G12D, RFTN1, *ZNF827*, PIK3CA, KMT2B(sub) / amp1q21, del1p22	KRAS G12D, RFTN1, *ZNF827*, FAM46C / amp1q21, del1p22		
33	t(11;14)	Diagnosis	Relapse 1			Differential Clonal Response			NRAS Q61K, ARID1A(InDel, sub), DIS3(InDel, sub)	NRAS Q61K(sub), KRAS Q61H(sub) / del1p22		
43	t(4;14)	Diagnosis	Relapse	Relapse		Differential Clonal Respon	Stable evolution		DIS3, PRKD2, TCL1A, RPL5(InDel, sub) / amp1q21	DIS3, PRKD2, TCL1A, RPL5(InDel, sub), ATM(sub) / amp1q21	DIS3, PRKD2,TCL1A, RPL5(InDel, sub), ATM / amp1q21	
50	t(11;14)	Diagnosis	Relapse			Differential Clonal Response			KRAS Q61H / amp1q21	KRAS Q61H / amp1q21		
54	t(6;14)	Diagnosis	Relapse			Differential Clonal Response			NRAS Q61R(sub), TRAF3(InDel) / gain1q21	NRAS Q61R, TRAF3(InDel) / amp1q21		
42	HRD	Diagnosis	Relapse			Differential Clonal Response			ZNF292, IRF4(sub), TRAF3(sub) / gain1q21	ZNF292, NRAS Q61K / gain1q21		
48	HRD	Diagnosis	Relapse			Differential Clonal Response			NRAS Q61K(sub), UBR5(sub) / gain1q21	NRAS Q61K, UBR5, RB1(sp) / amp1q21		
49	HRD	Diagnosis	Relapse			Differential Clonal Response			KMT2C(sub), ZNF292(InDel) / del1p22, amp1q21	ZNF292(InDel), FAM46C, FGFR3, KRAS Q61H / del1p22, gain1q21		
51	HRD	Diagnosis	Relapse			Differential Clonal Response			SAMHD1, NRAS Q61K(sub), PTPN11 / del1p22	SAMHD1, NRAS Q61K /del1p22, gain1q21		
52	HRD	Diagnosis	Relapse			Differential Clonal Response			NRAS Q61R, NCOR1(sp), RPL10(sub)	NRAS Q61R, NCOR1(sp), RPL10, BHLHE41(InDel), *CUL4B* / gain1q21		
53	HRD	Diagnosis	Relapse			Differential Clonal Response			/ gain1q21	TRAF3* / gain1q21		
55	HRD	Diagnosis	Relapse			Differential Clonal Response			/ gain1q21	KRAS G13R(sub), NRAS G12D(sub) / gain1q21		
58	HRD	Diagnosis	Relapse			Differential Clonal Response			TP53, BTG1, NRAS_G13R*/ gain1q21	TP53, BTG1, NRAS_Q61R*/ gain1q21		
60	HRD	Diagnosis	Relapse			Differential Clonal Response				NRAS Q61K, EGR1, USP7 / gain1q21		
65	HRD	Diagnosis	Relapse			Differential Clonal Response			NRAS_Q61H(sub), NRAS Q61K (sub-6%), KRAS G12S(sub)	NRAS_Q61K / del1p22, gain1q21		
63	t(8;14)	Diagnosis	Relapse			Differential Clonal Response			TP53, *UBE2G1(sub)*, IRF4, ZNF292(InDel) / del17p13§, gain1q21	TP53, *UBE2G1*, ZNF292(InDel) / del17p13§		
46	unknown	Diagnosis	Relapse			Differential Clonal Response			KRAS G13D, SP140, HIST1H1B,BHLHE41(InDel, sub) / del1p22, gain1q21	KRAS G13D, SP140, HIST1H1B, BHLHE41(InDel, sub), UBR5(sub) /del1p22, amp1q21, del17p13		
41	t(4;14)	Diagnosis	Relapse			Differential Clonal Response			/del1p22	/del17p13		
47	t(4;14)	Diagnosis	Relapse			Differential Clonal Response			DUSP2, FAM46C(InDel) / del1p22, amp1q21	DUSP2, FAM46C(InDel), NFKBIA(InDel, sub) / del1p22, amp1q21		
64	t(4;14)	Diagnosis	Relapse			Differential Clonal Response			FGFR3, EGR1, PRDM1(sub), CDKN1B / gain1q21, del1p22	FGFR3, EGR1, PRDM1(sub), CDKN1B(sub) / gain1q22, del1p22		
15	HRD	Diagnosis	Relapse 1	Relapse 2		Linear evolution	Differential Clonal Response		NRAS Q61K /del1p22	NRAS Q61K, *CUL4B(InDel, sub)* /del1p22	KRAS G13D, *CUL4B(InDel, sub)*, NRAS Q61K(sub) / del1p22	
18	HRD	Diagnosis	Relapse 1	Relapse 4		Linear evolution	Differential Clonal Response		CYLD, USP7, RB1(InDel), XBP1(InDel, sub), NRAS G12A(sub), CDKN1B (sub)* / gain 1q2	CYLD, USP7, RB1(InDel), NRAS G12A / gain 1q21	CYLD, USP7, RB1(InDel), NRAS G12D, EP300, TP53/del17p, gain 1q21	
25	HRD	Diagnosis	Relapse 2	Relapse 3		Linear evolution	Differential Clonal Response		NRAS Q61R	NRAS Q61R, UBR5(sub)	NRAS Q61R, UBRF(sub)	
27	t(4;14)	Diagnosis	Relapse 1	Relapse 2		Linear evolution	Linear evolution		KRAS G12A, KLHL6 / amp1q21	KRAS G12A, KLHL6, IRF4 / amp1q21, del1p22	KRAS G12A, KLHL6, IRF4 / amp1q21, del1p22	
40	t(11;14)	Diagnosis	Relapse	Relapse	Relapse	Linear evolution	Stable evolution	Stable evolution	BRAF	BRAF	BRAF	BRAF
9	t(11;14)	Diagnosis	Relapse 3			Linear evolution			KRAS G12S / gain 1q21	KRAS G12S / gain 1q21		
44	t(11;14)	Diagnosis	Relapse			Linear evolution			TRAF3, BRAF	TRAF3, BRAF(sub), KRAS Q61L(sub)		
61	t(11;14)	Diagnosis	Relapse			Linear evolution			ARID1A(InDel,sub) / del17p13	BRAF(sub), NRAS Y64D(sub)* / del17p13		
57	t(12;14)	Diagnosis	Relapse			Linear evolution			ACTG1, USP7, DTX1, HIST1H1D, ARID1A(InDel,sub) / gain1q21	ACTG1, USP7, DTX1, HIST1H1D, RASA2(sub) / gain1q21		
62	t(4;14)	Diagnosis	Relapse			Linear evolution			/ gain1q21, del1p22	SAMHD1, SETD2(sub) / gain1q21, del1p22		
14	unknown	Diagnosis	Relapse 1	Relapse 2	Relapse 3	Stable evolution	Linear evolution	Stable evolution	DUSP2, KRAS Q61H, XBP1, TET2 / gain 1q21	DUSP2, KRAS Q61H, XBP1, TET2 /gain 1q21	TET2, DUSP2, KRAS Q61H, XBP1, *EIF4A3(sub)*, CDKN1B(sub)*, SP140(InDel, sub) / gain 1q21	DUSP2, KRAS Q61H, XBP1, TET2, HUWE1(InDel, sub) / gain 1q21
17	t(11;14)	Diagnosis	Relapse 1			Stable evolution			BTG1, KRAS G12V, TP53 / del17p13	BTG1, KRAS G12V, TP53 / del17p13		
10	t(4;14)	Diagnosis	Relapse 1			Stable evolution			DIS3, FGFR3(InDel, sub) / amp1q21, del1p22	DIS3, FGFR3(InDel, sub), NF1(sub)* / amp1q21, del1p22		
7	unknown	Diagnosis	Relapse 1			Stable evolution			MAF, TCL1A /gain 1q21, del1p22, de17p13	MAF, TCL1A /gain 1q21, del1p22, del17p13		
29	HRD	Diagnosis	Relapse 1			Stable evolution			KRAS G13D	KRAS G13D		
30	HRD	Diagnosis	Relapse 1			Stable evolution			NRAS G13R, CYLD(sub)	NRAS G13R, CYLD(sub)		
45	t(11;14)	Diagnosis	Relapse			Stable evolution			KRAS Q61H, DIS3(sub), HIST1H1D, RPL5	KRAS Q61H, DIS3(sub), HIST1H1D, RPL5, TP53(sub)*		
56	t(11;14)	Diagnosis	Relapse			Stable evolution			KRAS Q61H, DUSP2, IRF4	KRAS Q61H, DUSP2, IRF4, KMT2C(InDel, sub)		
66	HRD	Diagnosis	Relapse			Stable evolution			HIST1H1E / gain1q21	HIST1H1E, KRAS Q61L(sub) / amp1q21		
67	HRD	Diagnosis	Relapse			Stable evolution			TRAF3, KRAS(InDel, sub)	TRAF3		
59	t(4;14)	Diagnosis	Relapse			Stable evolution			HIST1H1B(InDel)	HIST1H1B(InDel)		
35	HRD	Relapse 1	Relapse 2			Differential Clonal Response			NRAS Q61R(sub), SETX(sub)	NRAS Q61R		
3	unknown	Relapse 1	Relapse 2			Linear evolution			NRAS G12D, FAM46C(InDel), NCOR1(sub), FUBP1(sub)	NRAS G12D, FAM46C(InDel), NCOR1(sub), FUBP1(sub)		
8	t(11;14)	Relapse 1	Relapse 5			Linear evolution			NRAS Q61R, CCND1, DIS3, PRKD2, ARID1A(sub), USP7(sub), KMT2C(sub)	NRAS Q61R, CCND1, DIS3, PRKD2(sub), ARID1A, USP7, KMT2C		
34	HRD	Relapse 2	Relapse 3	Relapse 4	Relapse 6	Differential Clonal Respon	Differential Clonal Respons	Stable evolution	ATM, FAM46C / gain 1q21	ATM, FAM46C / gain 1q21	ATM, FAM46C, CYLD (sub) / gain 1q21	ATM, FAM46C / gain 1q21
36	HRD	Relapse 2	Relapse 5			Linear evolution			NRAS Q61K, FUBP1(sub) / gain 1q21	NRAS Q61K, DTX1(sub)* / amp1q21		
5	unknown	Relapse 5	Relapse 6			Differential Clonal Response			KRAS Q22K, SF3B1 / del1p22	KRAS Q22K, SF3B1(sub) / del1p22		

mutations/CNAs for all included tumor samples. Mutations included are 80 MM driver genes (Supplementary Table 4), non-italic, and mutations suggested to be involved in IMID/Proteasome Inhibitor drug resistance (REF [29,30,31] in manuscript), in italic. Only non-synonymous SNVs with CCF>5% and indels with VAF >5% are included, categorized to exonic or splicing(sp). SNVs with CCF < 60% or indels with VAF < 30% were classified as subclonal (sub).
*Found at low frequency (<5 % CCF(SNVs)/AF(InDel) at another sample timepoint.
Red: appearing at progression, or increasing from subclonal to clonal (at least 5x increase), and increase from gain1q to amp1q. Blue: Decreasing (at least 5x) or lost at later stage, or change from an amp1q to an gain1q
Included CN abberations: del17p13(§=not affecting TP53 gene), del1p22, gain1q21(>2 copies), amp1q(>=4 copies, including CKS1B region)
Ig-T/HRD: cytogenetic subroup, see Supplementary Table S12.
