# Supplementary material for: Clonal evolution after treatment pressure in multiple myeloma: heterogenous genomic aberrations and transcriptomic convergence
Source: Leukemia. 2022 May 28;36(7):1887–97. doi: 10.1038/s41375-022-01597-y (PMC9252918; doi:10.1038/s41375-022-01597-y)
Supplement: Supplementary file 12 — Table S6 [file 41375_2022_1597_MOESM12_ESM.pdf]

Patient_ID	Chr	Start	End	Ref	Alt	Func.refGene	Gene.refGene	GeneDetail.refGene	ExonicFunc.refGene	AAChange.refGene	avsnp150	cosmic88	T1_WT_count	T1_mut_count	T1_subclonal_fraction	T1_no_chrs_bearing_mut	T2_WT_count	T2_mut_count	T2_subclonal_fraction	T2_no_chrs_bearing_mut	T3_WT_count	T3_mut_count	T3_subclonal_fraction	T3_no_chrs_bearing_mut	T4_WT_count	T4_mut_count	T4_subclonal_fraction	T4_no_chrs_bearing_mut
3	chr1	236154337	236154337	G	A	exonic	NID1		nonsynonymous SNV	NID1:NM_002508:exon14:c.C2777T:p.P926	NOVEL		62	35	0.9142	1	67	49	0.990978	1	57	65	1.15353	1				
3	chr19	18119182	18119182	G	T	exonic	ARRDC2		nonsynonymous SNV	ARRDC2:NM_001286826:exon1:c.G63T:p.E	NOVEL		91	61	1.01679	1	124	103	1.06448	1	136	139	1.09436	1				
3	chr16	66956055	66956055	C	T	exonic	RRAD		nonsynonymous SNV	RRAD:NM_001128850:exon5:c.G851A:p.R	DBSNP+COSMIC	ID=COSM604622	205	124	0.954928	1	199	163	1.05634	1	211	201	1.05627	1				
3	chr10	129906849	129906849	C	T	exonic	MKI67		nonsynonymous SNV	MKI67:NM_001145966:exon12:c.G2175A:	COSMIC	ID=COSM393497	152	104	1.02929	1	182	162	1.1048	1	253	236	1.04491	1				
3	chr5	140789919	140789919	G	A	exonic	PCDHGB6		nonsynonymous SNV	PCDHGB6:NM_018926:exon1:c.G2150A:p.	NOVEL		303	138	1.1007	1	484	158	0.819219	1	516	256	1.04466	1				
3	chr5	178506679	178506679	G	A	exonic	ZNF354C		nonsynonymous SNV	ZNF354C:NM_014594:exon5:c.G1246A:p.E	DBSNP+COSMIC	ID=COSM301131	341	132	0.981611	1	422	177	0.983613	1	502	242	1.02469	1				
3	chr1	169697336	169697336	A	C	exonic	SELE		nonsynonymous SNV	SELE:NM_000450:exon8:c.T1142G:p.L381	NOVEL		181	98	0.889953	1	208	125	0.880626	1	229	198	1.00395	1				
3	chr5	170668080	170668080	C	A	exonic	RANBP17		nonsynonymous SNV	RANBP17:NM_022897:exon23:c.C2571A:p	DBSNP		220	88	1.00498	1	342	138	0.957008	1	430	201	1.0035	1				
3	chr10	55571379	55571379	C	T	exonic	PCDH15		nonsynonymous SNV	PCDH15:NM_001354429:exon35:c.G4426A	NOVEL		69	35	0.852668	1	75	57	1.01304	1	79	66	0.985491	1				
3	chr10	100148243	100148243	G	A	exonic	PYROXD2		nonsynonymous SNV	PYROXD2:NM_032709:exon13:c.C1315T:p.	COSMIC	ID=COSM205707	93	47	0.850579	1	108	79	0.991086	1	120	100	0.984133	1				
3	chr14	25288292	25288292	G	A	exonic	STXBP6		nonsynonymous SNV	STXBP6:NM_001304477:exon5:c.C560T:p.	DBSNP		91	55	0.954454	1	117	76	0.923809	1	117	97	0.981374	1				
3	chr15	51024863	51024863	C	A	exonic	SPPL2A		nonsynonymous SNV	SPPL2A:NM_032802:exon9:c.G951T:p.Q31	NOVEL		84	31	0.948179	1	113	48	0.992415	1	171	77	0.978115	1				
3	chr18	268006	268006	G	A	exonic	THOC1		nonsynonymous SNV	THOC1:NM_005131:exon1:c.C14T:p.P5L	DBSNP		181	104	0.924557	1	230	171	1.00041	1	266	216	0.970249	1				
3	chr16	72992049	72992049	T	C	exonic	ZFHX3		nonsynonymous SNV	ZFHX3:NM_006885:exon2:c.A1996G:p.N66	NOVEL		216	139	0.992045	1	303	231	1.01484	1	355	287	0.967884	1				
3	chr1	10710762	10710762	G	A	exonic	CASZ1		nonsynonymous SNV	CASZ1:NM_001079843:exon13:c.C2867T:p	COSMIC	ID=COSM581833	141	85	0.952918	1	177	131	0.997806	1	217	175	0.966559	1				
3	chr6	151726915	151726915	G	T	exonic	RMND1		nonsynonymous SNV	RMND1:NM_001271937:exon10:c.C747A:	NOVEL		62	40	0.993585	1	98	43	0.715443	1	89	71	0.96076	1				
3	chr2	111789235	111789235	C	A	exonic	ACOXL		nonsynonymous SNV	ACOXL:NM_001142807:exon15:c.C1313A:	NOVEL		208	113	0.891905	1	277	164	0.872431	1	270	215	0.959783	1				
3	chr7	83634814	83634814	T	C	exonic	SEMA3A		nonsynonymous SNV	SEMA3A:NM_006080:exon11:c.A1201G:p.	NOVEL		282	162	0.924436	1	402	243	0.883837	1	459	356	0.945734	1				
3	chr9	84606385	84606385	G	A	exonic	SPATA31D1		nonsynonymous SNV	SPATA31D1:NM_001001670:exon4:c.G100	DBSNP		298	169	0.916885	1	348	242	0.962253	1	400	309	0.943602	1				
3	chr15	79586109	79586109	C	T	exonic	ANKRD34C		synonymous SNV	ANKRD34C:NM_001146341:exon2:c.C483T	NOVEL		403	122	0.817386	1	472	205	1.00796	1	632	268	0.938087	1				
3	chr17	7193772	7193772	C	T	exonic	YBX2		nonsynonymous SNV	YBX2:NM_015982:exon5:c.G542A:p.R181H	DBSNP		90	46	0.856967	1	107	88	1.0587	1	135	103	0.936994	1				
3	chr5	50093056	50093056	C	G	exonic	PARP8		nonsynonymous SNV	PARP8:NM_001178056:exon14:c.C1564G:	DBSNP		92	25	0.75159	1	135	41	0.775442	1	168	71	0.935861	1				
3	chr1	115258747	115258747	C	T	exonic	NRAS		nonsynonymous SNV	NRAS:NM_002524:exon2:c.G35A:p.G12D	DBSNP+COSMIC	ID=COSM564;OC	116	59	0.854199	1	118	77	0.926364	1	148	110	0.923102	1				
3	chr2	179597261	179597261	G	A	exonic	TTN		synonymous SNV	TTN:NM_133378:exon53:c.C12795T:p.L42	NOVEL		209	108	0.770357	1	286	175	0.890559	1	341	252	0.920073	1				
3	chr4	151141898	151141898	G	A	exonic	DCLK2		nonsynonymous SNV	DCLK2:NM_001040260:exon6:c.G1100A:p.	NOVEL		216	130	0.951946	1	271	187	0.95786	1	349	242	0.886552	1				
3	chr15	58247466	58247466	C	T	exonic	ALDH1A2		nonsynonymous SNV	ALDH1A2:NM_170697:exon11:c.G1198A:p	COSMIC	ID=COSM592078	81	39	1.14317	1	96	49	1.12488	1	119	45	0.86441	1				
3	chr17	76230790	76230790	G	A	exonic	TMEM235		nonsynonymous SNV	TMEM235:NM_001204211:exon3:c.G307A	NOVEL		110	59	0.884525	1	140	65	0.74385	1	137	89	0.852625	1				
3	chr4	27004000	27004000	A	G	exonic	STIM2		nonsynonymous SNV	STIM2:NM_001169117:exon6:c.A787G:p.N	DBSNP		22	9	0.735573	1	24	24	1.17299	1	46	29	0.837169	1				
3	chr12	123425465	123425465	C	T	exonic	ABCB9		synonymous SNV	ABCB9:NM_019624:exon7:c.G1329A:p.Q4	NOVEL		217	4	0.0277582	1	228	71	0.319616	1	154	176	0.621383	1				
3	chr16	30371089	30371089	C	T	exonic	TBC1D10B		nonsynonymous SNV	TBC1D10B:NM_015527:exon5:c.G1345A:p	NOVEL		96	0	0	1	126	15	0.249573	1	116	46	0.614779	1				
3	chr2	234372881	234372881	G	A	exonic	DGKD		synonymous SNV	DGKD:NM_003648:exon26:c.G3126A:p.L1	NOVEL		226	1	0.0111614	1	298	34	0.240252	1	258	102	0.613443	1				
3	chr15	52672042	52672042	G	A	exonic	MYO5A		synonymous SNV	MYO5A:NM_000259:exon17:c.C2076T:p.I6	DBSNP		377	3	0.0277693	1	453	50	0.330887	1	532	128	0.610966	1				
3	chr15	40558602	40558602	G	A	exonic	BUB1B-PAK6;PAK		nonsynonymous SNV	PAK6:NM_001276717:exon3:c.G764A:p.S2	NOVEL		419	6	0.049658	1	532	51	0.291192	1	529	126	0.606011	1				
3	chr3	42727664	42727664	G	A	exonic	KLHL40		nonsynonymous SNV	KLHL40:NM_152393:exon1:c.G554A:p.G18	NOVEL		116	1	0.021655	1	141	24	0.341235	1	140	54	0.602655	1				
3	chr4	152510077	152510077	A	T	exonic	FAM160A1		nonsynonymous SNV	FAM160A1:NM_001348694:exon6:c.A977	NOVEL		287	3	0.0262101	1	360	50	0.286096	1	387	149	0.601864	1				
3	chr22	43280456	43280456	G	T	exonic	PACSIN2		nonsynonymous SNV	PACSIN2:NM_001349974:exon5:c.C598A:p	NOVEL		112	3	0.040008	1	97	22	0.248838	1	78	82	0.59711	1				
3	chr12	109537065	109537065	G	A	exonic	UNG		stopgain	UNG:NM_003362:exon2:c.G384A:p.W128	DBSNP		44	0	0	1	47	3	0.0807593	1	32	33	0.591509	1				
3	chr19	52002694	52002694	G	A	exonic	SIGLEC12		nonsynonymous SNV	SIGLEC12:NM_033329:exon2:c.C731T:p.S2	DBSNP+COSMIC	ID=COSM225781	127	2	0.0392812	1	134	20	0.304674	1	98	36	0.581667	1				
3	chr15	43021458	43021458	G	A	exonic	CDAN1		nonsynonymous SNV	CDAN1:NM_138477:exon18:c.C2510T:p.A8	NOVEL		229	0	0	1	321	19	0.186017	1	311	69	0.572027	1				
3	chr3	49050056	49050056	G	C	exonic	WDR6		synonymous SNV	WDR6:NM_001320546:exon2:c.G1011C:p.	NOVEL		306	1	0.0082529	1	321	50	0.316171	1	354	126	0.568337	1				
3	chr3	47036968	47036968	T	A	exonic	NBEAL2		nonsynonymous SNV	NBEAL2:NM_001365116:exon12:c.T1641A	NOVEL		313	1	0.00806892	1	386	50	0.269035	1	336	117	0.559196	1				
3	chr3	52883904	52883904	C	T	exonic	STIMATE;STIMATE		nonsynonymous SNV	STIMATE-MUSTN1:NM_001198974:exon4	DBSNP		140	1	0.0179691	1	186	26	0.287715	1	170	58	0.550769	1				
3	chr16	2025942	2025942	G	A	exonic	TBL3		nonsynonymous SNV	TBL3:NM_006453:exon11:c.G1121A:p.G37	COSMIC	ID=COSM124366	303	6	0.0491969	1	308	50	0.327652	1	345	117	0.548303	1				
3	chr19	46288855	46288855	C	T	exonic	DMWD		synonymous SNV	DMWD:NM_004943:exon3:c.G1899A:p.K6	NOVEL		79	0	0	1	100	17	0.34087	1	121	41	0.547956	1				
3	chr17	17124869	17124869	G	T	exonic	FLCN		nonsynonymous SNV	FLCN:NM_144606:exon8:c.C853A:p.Q285K	NOVEL		231	3	0.0324826	1	266	29	0.230623	1	255	86	0.546035	1				
3	chr13	20224229	20224229	G	C	exonic	MPHOSPH8		nonsynonymous SNV	MPHOSPH8:NM_017520:exon5:c.G1405C:	NOVEL		190	3	0.039383	1	227	34	0.305608	1	250	84	0.544514	1				
3	chr11	62678204	62678204	G	A	exonic	CHRM1		synonymous SNV	CHRM1:NM_000738:exon2:c.C369T:p.R12	NOVEL		408	1	0.0086001	1	543	48	0.270353	1	576	117	0.531868	1				
3	chr1	150040811	150040811	G	A	exonic	VPS45		nonsynonymous SNV	VPS45:NM_001279353:exon2:c.G110A:p.R	NOVEL		379	3	0.0198977	1	441	60	0.280957	1	579	188	0.530688	1				
3	chr5	159822289	159822289	G	C	exonic	ZBED8		nonsynonymous SNV	ZBED8:NM_022090:exon2:c.C209G:p.P70R	NOVEL		613	3	0.0171304	1	752	68	0.276041	1	967	194	0.526406	1				
3	chrX	47835632	47835632	T	G	exonic	ZNF182		synonymous SNV	ZNF182:NM_001007088:exon6:c.A1797C:	NOVEL		234	2	0.0107358	1	226	46	0.198374	1	165	154	0.522609	1				
3	chr16	29996580	29996580	A	G	exonic	TAOK2		nonsynonymous SNV	TAOK2:NM_001252043:exon14:c.A1469G:	NOVEL		147	1	0.0171192	1	156	23	0.30144	1	162	51	0.518403	1				
3	chr5	140603130	140603130	T	A	exonic	PCDHB14		nonsynonymous SNV	PCDHB14:NM_018934:exon1:c.T53A:p.L18	NOVEL		469	5	0.0371038	1	625	29	0.147604	1	712	140	0.517654	1				
3	chr15	43028130	43028130	C	T	exonic	CDAN1		nonsynonymous SNV	CDAN1:NM_138477:exon3:c.G716A:p.G23	NOVEL		398	2	0.0175872	1	509	42	0.253732	1	524	101	0.509087	1				
3	chr17	55926687	55926687	C	T	exonic	MRPS23		synonymous SNV	MRPS23:NM_016070:exon2:c.G129A:p.E4	NOVEL		121	2	0.0411974	1	150	28	0.369032	1	156	45	0.484722	1				
3	chr5	176813456	176813456	G	A	exonic	SLC34A1		nonsynonymous SNV	SLC34A1:NM_001167579:exon5:c.G421A:	DBSNP		329	3	0.0317841	1	433	27	0.195382	1	386	70	0.483598	1				
3	chr1	45116415	45116415	G	A	exonic	RNF220		nonsynonymous SNV	RNF220:NM_001319957:exon14:c.G1030A	NOVEL		192	1	0.0131277	1	243	28	0.24239	1	213	61	0.48201	1				
3	chr2	103334882	103334882	C	A	exonic	MFSD9		nonsynonymous SNV	MFSD9:NM_001322080:exon6:c.G1239T:p	NOVEL		213	1	0.0118394	1	259	35	0.279284	1	276	78	0.477054	1				
3	chr1	38082206	38082206	C	G	exonic	RSPO1		nonsynonymous SNV	RSPO1:NM_001242908:exon4:c.G236C:p.	NOVEL		157	1	0.0160357	1	207	21	0.216078	1	205	57	0.471032	1				
3	chr2	103334880	103334880	T	C	exonic	MFSD9		stoploss	MFSD9:NM_001322080:exon6:c.A1241G:p	NOVEL		206	1	0.0122398	1	255	33	0.268811	1	274	76	0.470134	1				
3	chr11	64521362	64521362	G	A	exonic	PYGM		nonsynonymous SNV	PYGM:NM_001164716:exon8:c.C964T:p.R	DBSNP		81	1	0.0428956	1	101	12	0.353493	1	104	18	0.464797	1				
3	chr17	49239140	49239140	C	T	exonic	NME1		synonymous SNV	NME1:NM_000269:exon5:c.C393T:p.G131	DBSNP+COSMIC	ID=COSM981362	82	1	0.0305258	1	110	9	0.177428	1	129	35	0.462063	1				
3	chr21	47776983	47776983	C	T	exonic	PCNT		synonymous SNV	PCNT:NM_001315529:exon13:c.C1677T:p.	NOVEL		189	0	0	1	261	25	0.290972	1	333	57	0.460427	1				
3	chr12	113402073	113402073	C	A	exonic	OAS3		nonsynonymous SNV	OAS3:NM_006187:exon11:c.C2263A:p.L75	DBSNP		37	1	0.040359	1	53	11	0.231342	1	35	22	0.449685	1				
3	chr17	8013595	8013595	G	A	exonic	ALOXE3		nonsynonymous SNV	ALOXE3:NM_001369446:exon9:c.C1117T:p	NOVEL		236	4	0.0422273	1	276	41	0.303424	1	264	69	0.448623	1				
3	chr19	58437645	58437645	G	A	exonic	ZNF418		nonsynonymous SNV	ZNF418:NM_001317030:exon2:c.C1649T:p	DBSNP		34	3	0.20543	1	34	5	0.300768	1	24	6	0.433019	1				
3	chr10	38241836	38241836	G	C	exonic	ZNF25		nonsynonymous SNV	ZNF25:NM_001329650:exon6:c.C590G:p.A	NOVEL		187	0	0	1	237	24	0.215723	1	313	77	0.427467	1				
3	chr2	97559702	97559702	C	T	exonic	FAM178B		synonymous SNV	FAM178B:NM_001172667:exon2:c.G114A	NOVEL		91	2	0.0544869	1	98	19	0.380972	1	102	25	0.426199	1				
3	chr16	16291979	16291979	C	A	exonic	ABCC6		nonsynonymous SNV	ABCC6:NM_001171:exon10:c.G1237T:p.V4	NOVEL		269	1	0.00938385	1	308	46	0.304846	1	293	65	0.393103	1				
3	chr17	38173156	38173156	C	G	exonic	CSF3		nonsynonymous SNV	CSF3:NM_001178147:exon4:c.C451G:p.L1	DBSNP		56	0	0	1	78	4	0.114438	1	57	12	0.376538	1				
3	chr17	15975504	15975504	A	G	exonic	NCOR1		nonsynonymous SNV	NCOR1:NM_001190440:exon28:c.T3898C:	NOVEL		235	32	0.303657	1	268	46	0.34368	1	353	70	0.35829	1				
3	chr5	101748740	101748740	G	C	exonic	SLCO6A1		nonsynonymous SNV	SLCO6A1:NM_001308014:exon4:c.C821G:	NOVEL		44	1	0.0781653	1	79	3	0.121783	1	91	11	0.339737	1				
3	chr21	46032235	46032235	C	T	exonic	KRTAP10-8		nonsynonymous SNV	KRTAP10-8:NM_198695:exon1:c.C218T:p.	DBSNP		134	0	0	1	179	1	0.0184929	1	161	12	0.218517	1				
3	chr4	114279939	114279939	A	C	exonic	ANK2		nonsynonymous SNV	ANK2:NM_001148:exon38:c.A10165C:p.K3	NOVEL		222	41	0.394978	1	311	55	0.352539	1	371	40	0.210715	1				
3	chr6	57372354	57372354	A	T	exonic	PRIM2		unknown	UNKNOWN	NOVEL		686	3	0.0110318	1	841	37	0.0988628	1	1112	106	0.188424	1				
3	chr9	115925141	115925141	G	T	exonic	SLC31A2		nonsynonymous SNV	SLC31A2:NM_001860:exon4:c.G376T:p.V1	NOVEL		48	0	0	1	57	0	0	1	60	5	0.166546	1				
3	chr5	175306932	175306932	C	T	exonic	CPLX2		nonsynonymous SNV	CPLX2:NM_001008220:exon4:c.C289T:p.P	DBSNP		180	2	0.0386532	1	268	7	0.0847312	1	308	15	0.146298	1				
3	chr16	48582010	48582010	T	G	exonic	N4BP1		nonsynonymous SNV	N4BP1:NM_153029:exon5:c.A2133C:p.L71	NOVEL		106	2	0.0469193	1	139	6	0.0970753	1	157	11	0.141762	1				
3	chr4	159566285	159566285	T	A	exonic	RXFP1		nonsynonymous SNV	RXFP1:NM_001253729:exon13:c.T1196A:	NOVEL		316	6	0.0472107	1	485	14	0.0658193	1	646	42	0.132171	1				
3	chr6	139165620	139165620	C	G	exonic	ECT2L		nonsynonymous SNV	ECT2L:NM_001195037:exon6:c.C667G:p.Q	NOVEL		154	7	0.110158	1	212	10	0.105675	1	260	16	0.125513	1				
3	chr5	150932761	150932761	C	T	exonic	FAT2		nonsynonymous SNV	FAT2:NM_001447:exon5:c.G4133A:p.G137	NOVEL		308	5	0.0561891	1	352	10	0.0919537	1	420	17	0.122551	1				
3	chr16	1831429	1831429	C	T	exonic	SPSB3		nonsynonymous SNV	SPSB3:NM_001324081:exon2:c.G56A:p.R1	DBSNP		126	4	0.0779582	1	223	18	0.175219	1	235	14	0.121732	1				
3	chr1	109953665	109953665	C	T	exonic	PSMA5		nonsynonymous SNV	PSMA5:NM_001199772:exon6:c.G355A:p.	NOVEL		155	0	0	1	212	0	0	1	269	16	0.121549	1				
3	chr3	25670552	25670552	C	G	exonic	TOP2B		nonsynonymous SNV	TOP2B:NM_001068:exon14:c.G1759C:p.E	NOVEL		110	0	0	1	139	5	0.0814579	1	187	11	0.120283	1				
3	chr20	30382220	30382220	G	C	exonic	TPX2		nonsynonymous SNV	TPX2:NM_012112:exon15:c.G1702C:p.A56	NOVEL		209	8	0.0934061	1	265	14	0.11772	1	338	19	0.115229	1				
3	chr20	585255	585255	G	A	exonic	TCF15		nonsynonymous SNV	TCF15:NM_004609:exon2:c.C580T:p.L194	NOVEL		124	6	0.116937	1	153	8	0.116571	1	143	8	0.114707	1				
3	chr8	144654796	144654796	G	A	exonic	MROH6		nonsynonymous SNV	MROH6:NM_001100878:exon1:c.C89T:p.A	NOVEL		65	5	0.109546	1	106	10	0.116033	1	98	10	0.107879	1				
3	chr15	89056188	89056188	G	A	exonic	DET1		synonymous SNV	DET1:NM_001144074:exon5:c.C1647T:p.C	NOVEL		296	2	0.023607	1	399	7	0.0573918	1	462	16	0.105449	1				
3	chr15	51792268	51792268	C	T	exonic	DMXL2		stopgain	DMXL2:NM_001174116:exon18:c.G3153A:	NOVEL		578	13	0.0773718	1	830	14	0.0552158	1	1013	34	0.102302	1				
3	chr7	98543380	98543380	C	G	exonic	TRRAP		nonsynonymous SNV	TRRAP:NM_001244580:exon32:c.C4484G:	NOVEL		213	6	0.0694148	1	316	9	0.0649658	1	369	18	0.100702	1				
3	chr1	158906899	158906899	G	A	exonic	PYHIN1		nonsynonymous SNV	PYHIN1:NM_152501:exon2:c.G199A:p.E67	COSMIC	ID=COSM229431	181	12	0.157532	1	273	11	0.0908657	1	335	16	0.0986937	1				
3	chr11	65339056	65339056	G	T	exonic	ZNRD2		nonsynonymous SNV	ZNRD2:NM_001303024:exon3:c.G340T:p.A	NOVEL		444	7	0.0545944	1	547	16	0.0945996	1	621	20	0.0982931	1				
3	chr15	76017489	76017489	C	T	exonic	ODF3L1		synonymous SNV	ODF3L1:NM_175881:exon2:c.C159T:p.G53	NOVEL		542	10	0.0637217	1	772	24	0.100364	1	943	30	0.0971313	1				
3	chr5	140750787	140750787	G	A	exonic	PCDHGB3		nonsynonymous SNV	PCDHGB3:NM_018924:exon1:c.G826A:p.E	COSMIC	ID=COSM455999	332	26	0.255456	1	488	29	0.186718	1	633	20	0.0964868	1				
3	chr11	60265049	60265049	A	T	exonic	MS4A12		nonsynonymous SNV	MS4A12:NM_001164470:exon2:c.A258T:p	NOVEL		270	6	0.0764661	1	412	9	0.0711604	1	508	16	0.0961921	1				
3	chr19	41939284	41939284	G	A	exonic	DMAC2		synonymous SNV	DMAC2:NM_001167871:exon4:c.C408T:p.	NOVEL		100	0	0	1	135	0	0	1	175	8	0.0946489	1				
3	chr11	123676791	123676791	G	T	exonic	OR6M1		synonymous SNV	OR6M1:NM_001005325:exon1:c.C267A:p.	NOVEL		574	10	0.0602301	1	761	25	0.105875	1	1024	31	0.0925678	1				
3	chr2	51254847	51254847	C	T	exonic	NRXN1		nonsynonymous SNV	NRXN1:NM_001135659:exon2:c.G565A:p.	NOVEL		304	8	0.0649651	1	496	9	0.0418097	1	474	21	0.0918524	1				
3	chr3	9991183	9991183	C	T	exonic	PRRT3		nonsynonymous SNV	PRRT3:NM_001318871:exon2:c.G617A:p.	NOVEL		240	31	0.289826	1	339	13	0.0866416	1	397	16	0.0838777	1				
3	chr21	41539192	41539192	C	T	exonic	DSCAM		nonsynonymous SNV	DSCAM:NM_001271534:exon16:c.G2971A	NOVEL		262	3	0.0398201	1	332	10	0.0973311	1	556	15	0.0827573	1				
3	chr20	39794434	39794434	G	A	exonic	PLCG1		synonymous SNV	PLCG1:NM_002660:exon16:c.G1767A:p.E	NOVEL		204	19	0.215871	1	299	15	0.112069	1	335	13	0.0808799	1				
3	chr19	58905931	58905931	G	A	exonic	RPS5		nonsynonymous SNV	RPS5:NM_001009:exon5:c.G515A:p.C172Y	NOVEL		173	3	0.0431871	1	208	6	0.0657754	1	284	11	0.0807323	1				
3	chr19	15302568	15302568	G	A	exonic	NOTCH3		nonsynonymous SNV	NOTCH3:NM_000435:exon5:c.C790T:p.P26	DBSNP		368	0	0	1	460	0	0	1	517	20	0.0806366	1				
3	chr1	75078450	75078450	G	C	exonic	ERICH3		nonsynonymous SNV	ERICH3:NM_001002912:exon9:c.C1044G:p	NOVEL		224	0	0	1	302	0	0	1	366	14	0.0797666	1				
3	chr22	43564879	43564879	C	T	exonic	TTLL12		nonsynonymous SNV	TTLL12:NM_015140:exon13:c.G1684A:p.A	NOVEL		76	3	0.0582395	1	94	7	0.0932863	1	85	6	0.0768193	1				
3	chr13	33253037	33253037	A	C	exonic	PDS5B		nonsynonymous SNV	PDS5B:NM_015032:exon10:c.A1028C:p.N	NOVEL		162	0	0	1	203	0	0	1	245	9	0.0767159	1				
3	chr19	51520480	51520480	C	G	exonic	KLK10		nonsynonymous SNV	KLK10:NM_001077500:exon3:c.G155C:p.C	NOVEL		30	8	0.533398	1	85	3	0.0799768	1	82	3	0.076415	1				
3	chr16	67964395	67964395	G	A	exonic	CTRL		nonsynonymous SNV	CTRL:NM_001907:exon5:c.C415T:p.P139S	NOVEL		324	0	0	1	487	1	0.00480735	1	603	22	0.0762113	1				
3	chr12	94543652	94543652	C	T	exonic	PLXNC1		nonsynonymous SNV	PLXNC1:NM_005761:exon1:c.C905T:p.A30	NOVEL		127	6	0.0691868	1	141	11	0.097407	1	144	10	0.0756554	1				
3	chr1	12091780	12091780	C	T	exonic	MIIP		nonsynonymous SNV	MIIP:NM_021933:exon10:c.C1082T:p.A361	NOVEL		238	3	0.0315391	1	315	13	0.0929812	1	341	12	0.0736009	1				
3	chr17	71746853	71746853	G	A	exonic	LOC100134391		nonsynonymous SNV	LOC100134391:NM_001278587:exon3:c.G	NOVEL		152	0	0	1	225	0	0	1	267	9	0.0706009	1				
3	chr18	2890927	2890927	G	C	exonic	EMILIN2		nonsynonymous SNV	EMILIN2:NM_032048:exon4:c.G802C:p.E2	NOVEL		230	26	0.257323	1	334	22	0.144977	1	448	15	0.0701434	1				
3	chr10	22606834	22606834	T	C	exonic	COMMD3;COMMD		nonsynonymous SNV	COMMD3-BMI1:NM_001204062:exon2:c.T	DBSNP+COSMIC	ID=COSM496591	244	0	0	1	344	0	0	1	500	16	0.0671347	1				
3	chr11	73584412	73584412	T	A	exonic	COA4		synonymous SNV	COA4:NM_016565:exon2:c.A12T:p.S4S	NOVEL		223	4	0.0619813	1	321	12	0.119954	1	380	8	0.0649545	1				
3	chr7	4014145	4014145	G	C	exonic	SDK1		synonymous SNV	SDK1:NM_152744:exon13:c.G1962C:p.G65	NOVEL		291	0	0	1	383	0	0	1	465	14	0.0632804	1				
3	chr1	161140736	161140736	C	T	exonic	PPOX		synonymous SNV	PPOX:NM_001350128:exon11:c.C1189T:p.	NOVEL		245	0	0	1	290	1	0.00806181	1	371	11	0.0623456	1				
3	chr9	100222749	100222749	C	G	exonic	TDRD7		nonsynonymous SNV	TDRD7:NM_001302884:exon6:c.C923G:p.A	DBSNP		409	0	0	1	541	0	0	1	676	20	0.0622153	1				
3	chr16	7743327	7743327	G	A	exonic	RBFOX1		nonsynonymous SNV	RBFOX1:NM_145893:exon12:c.G1069A:p.V	DBSNP+COSMIC	ID=COSM711255	388	0	0	1	532	0	0	1	578	17	0.0618598	1				
3	chr2	207175044	207175044	G	A	exonic	ZDBF2		nonsynonymous SNV	ZDBF2:NM_020923:exon5:c.G5792A:p.G19	DBSNP		283	24	0.19807	1	404	18	0.100066	1	648	19	0.0616743	1				
3	chr1	78429288	78429288	G	A	exonic	FUBP1		nonsynonymous SNV	FUBP1:NM_003902:exon13:c.C1154T:p.T3	NOVEL		125	5	0.0974477	1	176	9	0.114129	1	243	7	0.0606226	1				
3	chr22	50582641	50582641	T	C	exonic	MOV10L1		nonsynonymous SNV	MOV10L1:NM_001164104:exon18:c.T247	NOVEL		55	8	0.194748	1	80	7	0.108298	1	114	6	0.0582546	1				
3	chr5	102326096	102326096	G	A	exonic	PAM		stopgain	PAM:NM_138821:exon14:c.G1283A:p.W4	NOVEL		122	10	0.266473	1	185	6	0.104567	1	267	5	0.0579098	1				
3	chr9	100222752	100222752	C	G	exonic	TDRD7		stopgain	TDRD7:NM_001302884:exon6:c.C926G:p.S	NOVEL		411	0	0	1	548	0	0	1	679	18	0.0559134	1				
3	chr7	72738700	72738700	A	C	exonic	TRIM50		nonsynonymous SNV	TRIM50:NM_001281450:exon2:c.T86G:p.L	NOVEL		131	13	0.228731	1	188	10	0.118484	1	230	6	0.0550447	1				
3	chr1	902151	902151	G	A	exonic	PLEKHN1		nonsynonymous SNV	PLEKHN1:NM_001160184:exon2:c.G151A:	DBSNP+COSMIC	ID=COSM488986	132	12	0.211137	1	200	8	0.0902303	1	271	7	0.0545167	1				
3	chr1	103347283	103347283	A	G	exonic	COL11A1		synonymous SNV	COL11A1:NM_080630:exon63:c.T4662C:p.	NOVEL		163	12	0.173735	1	236	7	0.0675799	1	282	8	0.0540828	1				
3	chr1	86965558	86965558	A	G	exonic	CLCA1		nonsynonymous SNV	CLCA1:NM_001285:exon14:c.A2575G:p.I8	NOVEL		514	0	0	1	708	0	0	1	834	22	0.0503867	1				
3	chrX	27999308	27999308	G	C	exonic	DCAF8L1		synonymous SNV	DCAF8L1:NM_001017930:exon1:c.C144G:	DBSNP		173	0	0	1	212	0	0	1	220	10	0.0470672	1				
3	chr11	83194310	83194310	C	A	exonic	DLG2		nonsynonymous SNV	DLG2:NM_001142702:exon5:c.G356T:p.G1	NOVEL		406	28	0.226932	1	620	20	0.104023	1	811	12	0.0459338	1				
3	chr9	34623959	34623959	G	A	exonic	ARID3C		synonymous SNV	ARID3C:NM_001017363:exon3:c.C477T:p.	NOVEL		203	18	0.20636	1	294	14	0.106636	1	330	7	0.0449723	1				
3	chr1	186022170	186022170	G	C	exonic	HMCN1		nonsynonymous SNV	HMCN1:NM_031935:exon43:c.G6664C:p.G	NOVEL		440	35	0.186689	1	577	29	0.112267	1	849	17	0.0425018	1				
3	chr11	117309681	117309681	C	T	exonic	DSCAML1		nonsynonymous SNV	DSCAML1:NM_020693:exon24:c.G4163A:p	DBSNP+COSMIC	ID=COSM556447	361	0	0	1	460	17	0.118634	1	657	8	0.0378983	1				
3	chr3	14489177	14489177	A	G	exonic	SLC6A6		nonsynonymous SNV	SLC6A6:NM_001134368:exon5:c.A452G:p.	NOVEL		160	23	0.318436	1	228	11	0.107974	1	283	5	0.0375884	1				
3	chr1	1597380	1597380	C	G	exonic	SLC35E2B		synonymous SNV	SLC35E2B:NM_001110781:exon9:c.G1059	NOVEL		182	22	0.273236	1	322	18	0.124199	1	380	6	0.0336543	1				
3	chr11	119003872	119003872	C	T	exonic	HINFP		nonsynonymous SNV	HINFP:NM_001351963:exon8:c.C464T:p.T	NOVEL		276	9	0.111077	1	405	1	0.00819883	1	471	5	0.0330913	1				
3	chr7	24324934	24324934	G	C	exonic	NPY		synonymous SNV	NPY:NM_000905:exon2:c.G75C:p.L25L	NOVEL		103	11	0.244474	1	131	3	0.0525221	1	133	2	0.0320755	1				
3	chr15	75188636	75188636	G	A	exonic	MPI		nonsynonymous SNV	MPI:NM_001289156:exon5:c.G664A:p.A22	NOVEL		238	14	0.195413	1	347	11	0.102279	1	397	4	0.0314244	1				
3	chr20	34220842	34220842	C	T	exonic	CPNE1		nonsynonymous SNV	CPNE1:NM_001198863:exon2:c.G4A:p.A2	DBSNP		76	5	0.156398	1	131	10	0.166382	1	146	2	0.029258	1				
3	chr19	14092942	14092942	C	T	exonic	RFX1		synonymous SNV	RFX1:NM_002918:exon5:c.G612A:p.S204S	DBSNP		236	27	0.260108	1	334	11	0.0747996	1	467	6	0.0274642	1				
3	chr6	17281975	17281975	G	C	exonic	RBM24		nonsynonymous SNV	RBM24:NM_001143942:exon1:c.G163C:p.	NOVEL		89	5	0.134768	1	140	9	0.141704	1	160	2	0.0267295	1				
3	chr3	46753878	46753878	G	A	exonic	PRSS50		nonsynonymous SNV	PRSS50:NM_013270:exon6:c.C1016T:p.A3	COSMIC	ID=COSM161765	139	11	0.1858	1	255	6	0.0539307	1	251	3	0.025572	1				
3	chr2	74756587	74756587	G	T	exonic	AUP1		synonymous SNV	AUP1:NM_181575:exon2:c.C90A:p.L30L	NOVEL		216	15	0.164522	1	266	19	0.156399	1	330	3	0.0195053	1				
3	chr5	140730463	140730463	G	T	exonic	PCDHGB1		nonsynonymous SNV	PCDHGB1:NM_018922:exon1:c.G636T:p.M	NOVEL		363	9	0.0850993	1	529	4	0.0249811	1	594	3	0.0158306	1				
3	chr10	105810633	105810633	C	T	exonic	COL17A1		nonsynonymous SNV	COL17A1:NM_000494:exon26:c.G2065A:p	NOVEL		423	0	0	1	560	20	0.0808961	1	629	4	0.0136815	1				
3	chr5	146754707	146754707	T	C	exonic	STK32A		nonsynonymous SNV	STK32A:NM_001112724:exon11:c.T958C:p	NOVEL		401	22	0.18294	1	616	0	0	1	772	3	0.0121947	1				
3	chrX	54209412	54209412	C	G	exonic	FAM120C		nonsynonymous SNV	FAM120C:NM_001300788:exon1:c.G220C	NOVEL		63	7	0.126682	1	83	4	0.0539307	1	102	1	0.0105102	1				
3	chr5	54442537	54442537	C	A	exonic	CDC20B		stopgain	CDC20B:NM_001145734:exon3:c.G274T:p.	NOVEL		183	16	0.282809	1	296	0	0	1	394	1	0.00797543	1				
3	chr16	19725825	19725825	C	T	exonic	KNOP1		nonsynonymous SNV	KNOP1:NM_001012991:exon2:c.G533A:p.	NOVEL		507	19	0.0915193	1	675	21	0.0707841	1	815	3	0.00794044	1				
3	chr5	140348514	140348514	G	C	exonic	PCDHAC2		nonsynonymous SNV	PCDHAC2:NM_018899:exon1:c.G2163C:p.L	NOVEL		259	9	0.118123	1	335	10	0.0964848	1	442	1	0.00711127	1				
3	chrX	123518480	123518480	T	A	exonic	TENM1		nonsynonymous SNV	TENM1:NM_014253:exon29:c.A6280T:p.T	NOVEL		184	8	0.0527842	1	230	2	0.010112	1	310	2	0.0069394	1				
3	chr9	77442721	77442721	A	C	exonic	TRPM6		nonsynonymous SNV	TRPM6:NM_001177310:exon7:c.T799G:p.	NOVEL		212	7	0.0809839	1	278	17	0.135193	1	315	1	0.00685156	1				
3	chr1	49227023	49227023	G	A	exonic	BEND5		nonsynonymous SNV	BEND5:NM_024603:exon2:c.C346T:p.L116	NOVEL		208	8	0.0938385	1	323	10	0.0704501	1	367	1	0.0058834	1				
3	chr16	89347787	89347787	C	T	exonic	ANKRD11		synonymous SNV	ANKRD11:NM_001256183:exon9:c.G5163	NOVEL		225	8	0.086992	1	303	16	0.117667	1	373	1	0.00578902	1				
3	chr15	71124582	71124582	C	G	exonic	LARP6		nonsynonymous SNV	LARP6:NM_001286679:exon3:c.G733C:p.G	NOVEL		662	24	0.123059	1	877	18	0.0669464	1	1170	2	0.00537593	1				
3	chr19	16317190	16317190	G	T	exonic	AP1M1		nonsynonymous SNV	AP1M1:NM_001130524:exon3:c.G238T:p.	NOVEL		245	18	0.173405	1	357	0	0	1	402	1	0.00537244	1				
3	chr11	60184275	60184275	C	A	exonic	MS4A14		nonsynonymous SNV	MS4A14:NM_001079692:exon4:c.C1783A:	NOVEL		647	14	0.0744995	1	880	24	0.0883732	1	1226	2	0.00513077	1				
3	chr18	72997538	72997538	A	G	exonic	TSHZ1		nonsynonymous SNV	TSHZ1:NM_001308210:exon2:c.A176G:p.Y	NOVEL		200	17	0.198488	1	309	2	0.0150867	1	430	1	0.00502342	1				
3	chr1	156248784	156248784	C	G	exonic	SMG5		nonsynonymous SNV	SMG5:NM_001323614:exon2:c.G116C:p.C	NOVEL		249	9	0.0883828	1	366	1	0.00639234	1	477	1	0.00452948	1				
3	chr1	168066306	168066306	G	A	exonic	GPR161		nonsynonymous SNV	GPR161:NM_001267613:exon2:c.C305T:p.	NOVEL		313	14	0.108474	1	384	11	0.0653313	1	499	1	0.00433019	1				
3	chrX	118222894	118222894	G	T	exonic	KIAA1210		nonsynonymous SNV	KIAA1210:NM_020721:exon11:c.C2299A:p	NOVEL		150	6	0.0487239	1	205	1	0.00569414	1	258	1	0.00417972	1				
3	chr7	6554133	6554133	G	C	exonic	GRID2IP		synonymous SNV	GRID2IP:NM_001145118:exon8:c.C1296G:	DBSNP		315	14	0.107814	1	481	0	0	1	542	1	0.00398728	1				
3	chr19	49930040	49930040	T	A	exonic	GFY		nonsynonymous SNV	GFY:NM_001195256:exon1:c.T353A:p.L11	NOVEL		340	30	0.20543	1	485	0	0	1	592	1	0.00365108	1				
3	chr19	33247048	33247048	C	T	exonic	TDRD12		nonsynonymous SNV	TDRD12:NM_001110822:exon7:c.C733T:p.	NOVEL		404	21	0.125192	1	562	0	0	1	753	1	0.00287148	1				
3	chr7	20418851	20418851	G	C	exonic	ITGB8		nonsynonymous SNV	ITGB8:NM_002214:exon4:c.G566C:p.G189	NOVEL		417	26	0.148701	1	572	0	0	1	768	1	0.00281547	1				
3	chr1	235969887	235969887	G	C	exonic	LYST		nonsynonymous SNV	LYST:NM_000081:exon6:c.C2549G:p.S850	NOVEL		496	14	0.0695509	1	678	2	0.00689996	1	914	1	0.00236622	1				
3	chr15	73547054	73547054	C	T	exonic	NEO1		nonsynonymous SNV	NEO1:NM_001172623:exon13:c.C1976T:p.	COSMIC	ID=COSM630779	86	9	0.333231	1	157	0	0	1	187	0	0	1				
3	chr9	34613822	34613822	C	T	exonic	DCTN3		nonsynonymous SNV	DCTN3:NM_001281425:exon6:c.G434A:p.	NOVEL		155	16	0.237066	1	217	0	0	1	283	0	0	1				
3	chr15	75682022	75682022	C	G	exonic	SIN3A		nonsynonymous SNV	SIN3A:NM_001145357:exon16:c.G2992C:p	NOVEL		128	7	0.182386	1	165	0	0	1	200	0	0	1				
3	chr16	19775429	19775429	G	A	exonic	IQCK		synonymous SNV	IQCK:NM_001305121:exon6:c.G336A:p.K1	DBSNP		164	12	0.172748	1	220	0	0	1	293	0	0	1				
3	chr11	5729483	5729483	G	C	exonic	TRIM22		nonsynonymous SNV	TRIM22:NM_001199573:exon6:c.G842C:p.	NOVEL		315	16	0.170027	1	475	0	0	1	712	0	0	1				
3	chr15	34652402	34652402	C	T	exonic	LPCAT4		synonymous SNV	LPCAT4:NM_153613:exon12:c.G1152A:p.K	NOVEL		285	14	0.164696	1	378	0	0	1	466	0	0	1				
3	chr11	406876	406876	C	G	exonic	SIGIRR		synonymous SNV	SIGIRR:NM_001135053:exon8:c.G846C:p.L	NOVEL		165	8	0.162656	1	264	10	0.121486	1	314	0	0	1				
3	chr4	3129035	3129035	G	A	exonic	HTT		nonsynonymous SNV	HTT:NM_002111:exon12:c.G1447A:p.G483	NOVEL		122	8	0.155916	1	158	0	0	1	169	0	0	1				
3	chr18	55336611	55336611	G	A	exonic	ATP8B1		nonsynonymous SNV	ATP8B1:NM_005603:exon18:c.C2036T:p.A	NOVEL		247	16	0.154138	1	405	0	0	1	527	0	0	1				
3	chr11	89896496	89896496	G	A	exonic	NAALAD2		nonsynonymous SNV	NAALAD2:NM_001300930:exon9:c.G995A	DBSNP		455	20	0.148103	1	683	1	0.00486656	1	1016	0	0	1				
3	chr5	24509762	24509762	A	T	exonic	CDH10		nonsynonymous SNV	CDH10:NM_001317224:exon7:c.T1169A:p.	NOVEL		504	22	0.147117	1	703	0	0	1	946	0	0	1				
3	chr16	4943603	4943603	T	G	exonic	PPL		synonymous SNV	PPL:NM_002705:exon13:c.A1431C:p.A477	NOVEL		146	9	0.147115	1	220	0	0	1	258	0	0	1				
3	chr7	50135868	50135868	G	C	exonic	SPATA48		nonsynonymous SNV	SPATA48:NM_001161834:exon1:c.G187C:	NOVEL		503	31	0.147084	1	824	0	0	1	1023	0	0	1				
3	chr9	131745753	131745753	G	T	exonic	NUP188		nonsynonymous SNV	NUP188:NM_015354:exon19:c.G1885T:p.	NOVEL		244	15	0.146736	1	397	0	0	1	459	0	0	1				
3	chr19	2987969	2987969	C	T	exonic	TLE6		synonymous SNV	TLE6:NM_024760:exon9:c.C330T:p.V110V,	DBSNP+COSMIC	ID=COSM407654	104	6	0.138199	1	150	6	0.0902303	1	175	0	0	1				
3	chr10	50740980	50740980	G	C	exonic	ERCC6		nonsynonymous SNV	ERCC6:NM_000124:exon2:c.C31G:p.Q11E,	DBSNP		287	16	0.13379	1	391	14	0.0810959	1	581	0	0	1				
3	chr15	72691045	72691045	C	T	exonic	TMEM202		stopgain	TMEM202:NM_001080462:exon2:c.C133T	NOVEL		228	9	0.133574	1	328	11	0.108012	1	425	0	0	1				
3	chr21	45540602	45540602	G	C	exonic	LOC102724159;PW		synonymous SNV	LOC102724159:NM_001368238:exon12:c.	NOVEL		311	12	0.130679	1	503	9	0.0585127	1	580	0	0	1				
3	chr17	4577926	4577926	C	G	exonic	PELP1		nonsynonymous SNV	PELP1:NM_014389:exon13:c.G1461C:p.Q4	DBSNP		204	11	0.129628	1	299	0	0	1	341	0	0	1				
3	chr19	5923298	5923298	C	T	exonic	RANBP3		synonymous SNV	RANBP3:NM_001300865:exon12:c.G897A	DBSNP		282	15	0.127962	1	379	0	0	1	434	0	0	1				
3	chr4	115597329	115597329	A	C	exonic	UGT8		nonsynonymous SNV	UGT8:NM_003360:exon5:c.A1511C:p.K504	NOVEL		153	8	0.125895	1	225	0	0	1	309	0	0	1				
3	chrX	64709057	64709057	C	T	exonic	ZC3H12B		stopgain	ZC3H12B:NM_001010888:exon1:c.C376T:p	NOVEL		155	17	0.125209	1	211	0	0	1	228	0	0	1				
3	chr6	39846988	39846988	G	A	exonic	DAAM2		nonsynonymous SNV	DAAM2:NM_001201427:exon14:c.G1580A	NOVEL		235	12	0.123092	1	335	12	0.0811293	1	404	0	0	1				
3	chr1	23222966	23222966	G	A	exonic	EPHB2		nonsynonymous SNV	EPHB2:NM_001309193:exon9:c.G1760A:p.	NOVEL		263	13	0.119338	1	340	0	0	1	425	0	0	1				
3	chr11	61205548	61205548	G	A	exonic	SDHAF2		synonymous SNV	SDHAF2:NM_017841:exon3:c.G333A:p.E11	NOVEL		229	8	0.118732	1	321	0	0	1	432	0	0	1				
3	chr16	71967392	71967392	G	A	exonic	PKD1L3		unknown	UNKNOWN	NOVEL		169	8	0.114515	1	226	0	0	1	330	0	0	1				
3	chr6	50719014	50719014	C	T	exonic	TFAP2D		synonymous SNV	TFAP2D:NM_172238:exon7:c.C1116T:p.I37	DBSNP		557	25	0.108833	1	844	0	0	1	1192	0	0	1				
3	chr2	48722902	48722902	G	T	exonic	PPP1R21		nonsynonymous SNV	PPP1R21:NM_001135629:exon16:c.G1684	NOVEL		183	8	0.106121	1	221	0	0	1	273	0	0	1				
3	chr21	47957447	47957447	A	G	exonic	DIP2A		nonsynonymous SNV	DIP2A:NM_001146115:exon14:c.A1667G:p	NOVEL		450	12	0.0913621	1	631	17	0.0873277	1	786	0	0	1				
3	chr3	168802710	168802710	A	T	exonic	MECOM		nonsynonymous SNV	MECOM:NM_001163999:exon15:c.T3119A	NOVEL		83	3	0.0883828	1	109	6	0.122399	1	129	0	0	1				
3	chr6	142455819	142455819	A	C	exonic	GJE1		synonymous SNV	GJE1:NM_001358410:exon3:c.A378C:p.L1	NOVEL		309	10	0.0794245	1	426	5	0.0272156	1	545	0	0	1				
3	chr20	2293602	2293602	G	A	exonic	TGM3		nonsynonymous SNV	TGM3:NM_003245:exon5:c.G599A:p.R200	DBSNP+COSMIC	ID=COSM102516	251	8	0.0782592	1	319	14	0.0986301	1	451	0	0	1				
3	chr2	27565776	27565776	C	T	exonic	GTF3C2		synonymous SNV	GTF3C2:NM_001035521:exon3:c.G486A:p	NOVEL		130	4	0.0756311	1	174	8	0.10312	1	244	0	0	1				
3	chr8	21985222	21985222	G	T	exonic	HR		nonsynonymous SNV	HR:NM_005144:exon3:c.C733A:p.R245S,H	NOVEL		157	8	0.0743583	1	219	0	0	1	221	0	0	1				
3	chr6	131924333	131924333	G	T	exonic	MED23		nonsynonymous SNV	MED23:NM_001270521:exon16:c.C1768A:	NOVEL		289	8	0.0682462	1	358	16	0.100363	1	495	0	0	1				
3	chr8	95479746	95479746	T	G	exonic	RAD54B		nonsynonymous SNV	RAD54B:NM_001205262:exon2:c.A22C:p.S	NOVEL		228	10	0.0644387	1	320	1	0.00419311	1	435	0	0	1				
3	chr12	80222213	80222213	T	A	exonic	PPP1R12A		nonsynonymous SNV	PPP1R12A:NM_001143886:exon5:c.A418T	NOVEL		99	4	0.0595589	1	116	8	0.0868379	1	183	0	0	1				
3	chr8	42814397	42814397	A	C	exonic	HOOK3		synonymous SNV	HOOK3:NM_032410:exon8:c.A555C:p.L18	COSMIC	ID=COSM82182;	235	0	0	1	269	11	0.0528781	1	362	0	0	1				
3	chr10	105813676	105813676	A	C	splicing	COL17A1	NM_000494:exon22:c			NOVEL		102	0	0	1	151	18	0.249869	1	150	48	0.524871	1				
4	chr19	464062	464062	G	T	exonic	ODF3L2		nonsynonymous SNV	ODF3L2:NM_182577:exon4:c.C652A:p.R21	NOVEL		103	40	0.832544	1	58	32	1.17462	1								
4	chr16	2230843	2230843	G	A	exonic	CASKIN1		synonymous SNV	CASKIN1:NM_020764:exon18:c.C2526T:p.	NOVEL		32	41	1.14254	1	17	17	1.17025	1								
4	chr1	234509281	234509281	G	C	exonic	COA6		nonsynonymous SNV	COA6:NM_001206641:exon1:c.G69C:p.R23	NOVEL		221	6	0.0537694	1	59	59	1.17025	1								
4	chr6	38980079	38980079	C	T	exonic	DNAH8		nonsynonymous SNV	DNAH8:NM_001371:exon89:c.C12809T:p.S	NOVEL		93	84	0.965419	1	49	47	1.14587	1								
4	chr3	195306288	195306288	G	A	exonic	APOD		synonymous SNV	APOD:NM_001647:exon2:c.C45T:p.F15F	DBSNP+COSMIC	ID=COSM660242	104	100	0.997194	1	70	65	1.12691	1								
4	chr8	20110529	20110529	C	T	exonic	LZTS1		nonsynonymous SNV	LZTS1:NM_001362884:exon3:c.G913A:p.E	DBSNP		127	141	1.07027	1	80	73	1.11671	1								
4	chr4	119259434	119259434	C	T	exonic	PRSS12		nonsynonymous SNV	PRSS12:NM_003619:exon2:c.G538A:p.E18	NOVEL		314	0	0	1	97	86	1.09991	1								
4	chr19	4432037	4432037	G	C	exonic	CHAF1A		nonsynonymous SNV	CHAF1A:NM_005483:exon12:c.G2036C:p.R	NOVEL		233	115	0.983562	1	143	71	1.09606	1								
4	chr5	38942471	38942471	C	T	exonic	RICTOR		nonsynonymous SNV	RICTOR:NM_001285440:exon38:c.G4093A	NOVEL		538	2	0.00753435	1	164	142	1.08611	1								
4	chr15	62209658	62209658	T	A	exonic	VPS13C		nonsynonymous SNV	VPS13C:NM_017684:exon58:c.A7808T:p.D	NOVEL		237	226	0.992973	1	123	106	1.08338	1								
4	chr4	41526454	41526454	G	T	exonic	LIMCH1		nonsynonymous SNV	LIMCH1:NM_001112717:exon3:c.G196T:p.	NOVEL		525	1	0.00386744	1	153	126	1.057	1								
4	chr17	80543844	80543844	G	C	exonic	FOXK2		nonsynonymous SNV	FOXK2:NM_004514:exon7:c.G1344C:p.K44	NOVEL		409	0	0	1	140	115	1.05552	1								
4	chr3	194081503	194081503	C	T	exonic	LRRC15		synonymous SNV	LRRC15:NM_130830:exon2:c.G270A:p.S90	NOVEL		208	192	0.976452	1	125	102	1.05168	1								
4	chr9	27950339	27950339	G	A	exonic	LINGO2		nonsynonymous SNV	LINGO2:NM_001354574:exon6:c.C331T:p.	DBSNP+COSMIC	ID=COSM277180	252	230	0.970712	1	135	110	1.05084	1								
4	chr11	20178766	20178766	G	T	exonic	DBX1		synonymous SNV	DBX1:NM_001029865:exon3:c.C489A:p.P1	NOVEL		48	0	0	1	16	13	1.04919	1								
4	chr7	70255926	70255926	G	A	exonic	AUTS2		nonsynonymous SNV	AUTS2:NM_001127231:exon18:c.G3652A:	DBSNP		150	129	0.940579	1	94	76	1.04634	1								
4	chr7	135312809	135312809	C	A	exonic	NUP205		nonsynonymous SNV	NUP205:NM_001329434:exon34:c.C3808A	NOVEL		165	0	0	1	57	46	1.04527	1								
4	chr1	210857002	210857002	T	C	exonic	KCNH1		nonsynonymous SNV	KCNH1:NM_002238:exon11:c.A2510G:p.E	COSMIC	ID=COSM571206	1642	32	0.115351	1	606	149	1.04343	1								
4	chr18	6882141	6882141	C	T	exonic	ARHGAP28		synonymous SNV	ARHGAP28:NM_001010000:exon10:c.C81	NOVEL		62	60	1.00046	1	33	26	1.03141	1								
4	chr11	85627187	85627187	G	A	exonic	CCDC83		nonsynonymous SNV	CCDC83:NM_001286159:exon10:c.G991A:	DBSNP		258	239	0.978253	1	169	130	1.01761	1								
4	chr13	96519616	96519616	T	G	exonic	UGGT2		nonsynonymous SNV	UGGT2:NM_020121:exon30:c.A3535C:p.K	DBSNP+COSMIC	ID=COSM607487	3	53	0.978868	1	9	28	1.01443	1								
4	chr19	36124673	36124673	G	T	exonic	RBM42		nonsynonymous SNV	RBM42:NM_001319113:exon6:c.G682T:p.	NOVEL		158	9	0.109632	1	59	45	1.01272	1								
4	chr6	101247445	101247445	T	C	exonic	ASCC3		synonymous SNV	ASCC3:NM_001284271:exon7:c.A1131G:p.	NOVEL		260	236	0.967921	1	134	101	1.00592	1								
4	chr4	70921228	70921228	C	G	exonic	HTN1		stopgain	HTN1:NM_001368990:exon4:c.C95G:p.S32	NOVEL		108	95	0.952001	1	67	50	1.00021	1								
4	chr4	141320169	141320169	C	T	exonic	CLGN		synonymous SNV	CLGN:NM_004362:exon8:c.G720A:p.E240	NOVEL		437	0	0	1	114	85	0.999711	1								
4	chr17	79620204	79620204	C	A	exonic	PDE6G		nonsynonymous SNV	PDE6G:NM_001365724:exon2:c.G132T:p.K	NOVEL		125	138	1.06741	1	97	72	0.997136	1								
4	chr11	117710519	117710519	A	T	exonic	FXYD6;FXYD6-FXY		stoploss	FXYD6:NM_001164831:exon7:c.T286A:p.X	NOVEL		320	1	0.00633731	1	109	80	0.990687	1								
4	chr1	89658687	89658687	G	A	exonic	GBP4		synonymous SNV	GBP4:NM_052941:exon5:c.C570T:p.D190	DBSNP		159	0	0	1	27	76	0.989106	1								
4	chr9	108110708	108110708	G	A	exonic	SLC44A1		nonsynonymous SNV	SLC44A1:NM_001286730:exon5:c.G476A:p	NOVEL		681	0	0	1	231	168	0.985473	1								
4	chr9	136208445	136208445	C	T	exonic	MED22		synonymous SNV	MED22:NM_133640:exon5:c.G513A:p.L17	NOVEL		101	5	0.0959564	1	35	25	0.975208	1								
4	chr2	20478524	20478524	C	G	exonic	PUM2		nonsynonymous SNV	PUM2:NM_001282752:exon12:c.G1609C:p	NOVEL		127	0	0	1	19	50	0.971376	1								
4	chr20	5925488	5925488	T	A	exonic	TRMT6		nonsynonymous SNV	TRMT6:NM_015939:exon3:c.A329T:p.D11	NOVEL		153	0	0	1	54	38	0.966728	1								
4	chr12	72335468	72335468	G	C	exonic	TPH2		nonsynonymous SNV	TPH2:NM_173353:exon2:c.G210C:p.L70F	NOVEL		397	24	0.115968	1	138	96	0.960205	1								
4	chr12	114793639	114793639	C	A	exonic	TBX5		nonsynonymous SNV	TBX5:NM_080717:exon8:c.G1105T:p.D369	NOVEL		154	132	0.938896	1	93	64	0.954089	1								
4	chr1	205238967	205238967	G	A	exonic	TMCC2		nonsynonymous SNV	TMCC2:NM_001297611:exon2:c.G962A:p.	NOVEL		655	94	0.757306	1	266	58	0.946474	1								
4	chr7	103969656	103969656	G	A	exonic	LHFPL3		stopgain	LHFPL3:NM_199000:exon1:c.G429A:p.W14	NOVEL		147	146	1.01367	1	93	63	0.945202	1								
4	chr11	82997198	82997198	C	A	exonic	CCDC90B		synonymous SNV	CCDC90B:NM_001286116:exon1:c.G18T:p.	NOVEL		171	0	0	1	65	44	0.944789	1								
4	chr6	160679630	160679630	G	T	exonic	SLC22A2		nonsynonymous SNV	SLC22A2:NM_003058:exon1:c.C160A:p.P5	NOVEL		279	1	0.00726527	1	109	72	0.931027	1								
4	chr12	25148964	25148964	T	C	exonic	C12orf77		nonsynonymous SNV	C12orf77:NM_001101339:exon3:c.A184G:	NOVEL		194	207	1.05011	1	127	83	0.925054	1								
4	chr2	241979737	241979737	G	C	exonic	SNED1		nonsynonymous SNV	SNED1:NM_001080437:exon8:c.G1180C:p	NOVEL		120	0	0	1	27	59	0.919645	1								
4	chr7	100361681	100361681	T	G	exonic	ZAN		unknown	UNKNOWN	NOVEL		415	0	0	1	149	96	0.917094	1								
4	chr11	4148403	4148403	T	C	exonic	RRM1		nonsynonymous SNV	RRM1:NM_001318065:exon8:c.T595C:p.Y	NOVEL		284	0	0	1	114	73	0.913671	1								
4	chr1	216073533	216073533	A	C	exonic	USH2A		stopgain	USH2A:NM_206933:exon40:c.T7478G:p.L	NOVEL		334	0	NA	1	118	75	0.90952	1								
4	chr17	10404815	10404815	G	A	exonic	MYH1		nonsynonymous SNV	MYH1:NM_005963:exon27:c.C3350T:p.A1	NOVEL		332	0	0	1	112	71	0.908062	1								
4	chr3	143708495	143708495	T	C	exonic	DIPK2A		nonsynonymous SNV	DIPK2A:NM_001134470:exon3:c.T478C:p.S	NOVEL		154	149	1.00035	1	120	76	0.90754	1								
4	chr14	42360757	42360757	A	G	exonic	LRFN5		nonsynonymous SNV	LRFN5:NM_001346173:exon3:c.A1690G:p	COSMIC	ID=COSM623501	401	0	0	1	129	81	0.902764	1								
4	chr3	151090416	151090416	A	G	exonic	MED12L		synonymous SNV	MED12L:NM_053002:exon25:c.A3621G:p.	NOVEL		497	0	0	1	161	101	0.902253	1								
4	chr14	81744600	81744600	C	G	exonic	STON2		nonsynonymous SNV	STON2:NM_001256430:exon6:c.G1055C:p	NOVEL		488	0	0	1	177	111	0.902067	1								
4	chr17	49353272	49353272	C	T	exonic	UTP18		nonsynonymous SNV	UTP18:NM_016001:exon6:c.C757T:p.R253	DBSNP+COSMIC	ID=COSM473999	191	160	0.927305	1	122	76	0.898373	1								
4	chr16	85699699	85699699	A	T	exonic	GSE1		nonsynonymous SNV	GSE1:NM_001134473:exon12:c.A2564T:p.	NOVEL		307	0	0	1	104	64	0.891619	1								
4	chr19	45117070	45117070	G	T	exonic	IGSF23		nonsynonymous SNV	IGSF23:NM_001205280:exon1:c.G115T:p.	NOVEL		328	0	0	1	144	88	0.887776	1								
4	chr12	67700006	67700006	G	A	exonic	CAND1		nonsynonymous SNV	CAND1:NM_018448:exon10:c.G2558A:p.G	NOVEL		382	0	0	1	123	75	0.886553	1								
4	chr7	1579714	1579714	A	G	exonic	MAFK		synonymous SNV	MAFK:NM_002360:exon3:c.A174G:p.T58T	DBSNP		116	80	0.830317	1	71	43	0.88282	1								
4	chr4	15780143	15780143	G	C	exonic	CD38		nonsynonymous SNV	CD38:NM_001775:exon1:c.G106C:p.V36L	NOVEL		342	0	0	1	139	84	0.881623	1								
4	chr14	77579971	77579971	G	T	exonic	CIPC		nonsynonymous SNV	CIPC:NM_033426:exon4:c.G510T:p.R170S	NOVEL		310	1	0.00654108	1	136	82	0.880371	1								
4	chr2	169038571	169038571	C	A	exonic	STK39		nonsynonymous SNV	STK39:NM_013233:exon2:c.G238T:p.A80S	NOVEL		397	0	0	1	155	93	0.877687	1								
4	chr11	4929455	4929455	A	G	exonic	OR51A7		nonsynonymous SNV	OR51A7:NM_001004749:exon1:c.A856G:p	NOVEL		311	0	0	1	133	79	0.872167	1								
4	chr5	168098480	168098480	T	A	exonic	SLIT3		nonsynonymous SNV	SLIT3:NM_001271946:exon34:c.A3871T:p.	NOVEL		167	0	0	1	58	34	0.864967	1								
4	chr14	37736288	37736288	A	C	exonic	MIPOL1		nonsynonymous SNV	MIPOL1:NM_138731:exon5:c.A165C:p.E55	NOVEL		462	0	0	1	160	93	0.860342	1								
4	chr16	20359802	20359802	T	C	exonic	UMOD		nonsynonymous SNV	UMOD:NM_001008389:exon3:c.A821G:p.	DBSNP		147	118	0.905828	1	82	47	0.85274	1								
4	chr6	13618364	13618364	T	C	exonic	NOL7		nonsynonymous SNV	NOL7:NM_001317724:exon5:c.T493C:p.S1	NOVEL		48	60	1.13015	1	42	24	0.851091	1								
4	chr1	26387751	26387751	G	A	exonic	TRIM63		nonsynonymous SNV	TRIM63:NM_032588:exon3:c.C407T:p.T13	DBSNP		79	79	1.01714	1	60	34	0.846563	1								
4	chr7	23299622	23299622	C	T	exonic	GPNMB		stopgain	GPNMB:NM_001005340:exon5:c.C565T:p.	DBSNP+COSMIC	ID=COSM108858	143	120	0.928187	1	103	58	0.843161	1								
4	chr20	43108865	43108865	C	G	exonic	TTPAL		nonsynonymous SNV	TTPAL:NM_001039199:exon2:c.C226G:p.L	NOVEL		264	0	0	1	93	52	0.839351	1								
4	chr7	107572706	107572706	A	T	exonic	LAMB1		synonymous SNV	LAMB1:NM_002291:exon28:c.T4305A:p.T	NOVEL		411	0	0	1	168	93	0.833971	1								
4	chr14	31142486	31142486	C	G	exonic	SCFD1		stopgain	SCFD1:NM_001283033:exon9:c.C464G:p.S	NOVEL		123	0	0	1	44	24	0.826058	1								
4	chr3	50141685	50141685	G	A	exonic	RBM5		nonsynonymous SNV	RBM5:NM_005778:exon8:c.G572A:p.C191	NOVEL		210	1	0.00964112	1	75	40	0.814087	1								

4	chr16	24801929	24801929	A	G	exonic	TNRC6A		nonsynonymous SNV	TNRC6A:NM_001330520:exon6:c.A1966G:	DBSNP		163	140	0.939929	1	115	61	0.811196	1
4	chr1	203317198	203317198	A	G	exonic	FMOD		synonymous SNV	FMOD:NM_002023:exon2:c.T201C:p.S67S	NOVEL		754	1	0.00799242	1	336	49	0.806973	1
4	chr5	133747408	133747408	G	A	exonic	CDKN2AIPNL		synonymous SNV	CDKN2AIPNL:NM_080656:exon1:c.C138T:p	NOVEL		71	60	0.931729	1	50	26	0.800697	1
4	chr1	152484143	152484143	C	T	exonic	LCE5A		nonsynonymous SNV	LCE5A:NM_178438:exon2:c.C133T:p.P45S	COSMIC	ID=COSM675281	607	173	0.894782	1	404	92	0.795283	1
4	chr6	12718995	12718995	G	A	exonic	PHACTR1		nonsynonymous SNV	PHACTR1:NM_001322310:exon1:c.G19A:p	COSMIC	ID=COSM362016	169	0	0	1	69	31	0.725555	1
4	chrX	30261146	30261146	T	G	exonic	MAGEB4		nonsynonymous SNV	MAGEB4:NM_002367:exon1:c.T894G:p.N2	NOVEL		238	253	0.71358	1	166	118	0.658304	1
4	chrX	148038132	148038132	C	A	exonic	AFF2		nonsynonymous SNV	AFF2:NM_001170628:exon8:c.C1480A:p.R	NOVEL		201	1	0.00685571	1	65	46	0.656596	1
4	chrX	69503155	69503155	G	A	exonic	RAB41		nonsynonymous SNV	RAB41:NM_001032726:exon4:c.G272A:p.R	NOVEL		129	138	0.715767	1	92	62	0.637873	1
4	chr7	100635283	100635283	T	C	exonic	MUC12		nonsynonymous SNV	MUC12:NM_001164462:exon2:c.T1439C:p	NOVEL		471	0	0	1	206	76	0.630773	1
4	chr9	120475507	120475507	T	C	exonic	TLR4		synonymous SNV	TLR4:NM_138557:exon2:c.T501C:p.F167F,	NOVEL		245	0	0	1	102	35	0.597938	1
4	chr2	219885907	219885907	A	G	exonic	CFAP65		nonsynonymous SNV	CFAP65:NM_194302:exon19:c.T3226C:p.W	NOVEL		224	0	0	1	100	32	0.567394	1
4	chr7	150066848	150066848	G	C	exonic	REPIN1		synonymous SNV	REPIN1:NM_001099695:exon2:c.G48C:p.G	NOVEL		164	0	0	1	66	21	0.564948	1
4	chr16	31425858	31425858	C	T	exonic	ITGAD		nonsynonymous SNV	ITGAD:NM_001318185:exon17:c.C2086T:p	DBSNP+COSMIC	ID=COSM350922	328	0	0	1	171	21	0.255992	1
4	chr6	146480538	146480538	G	T	exonic	GRM1		nonsynonymous SNV	GRM1:NM_001278064:exon2:c.G755T:p.G	NOVEL		146	0	0	1	96	6	0.137676	1
4	chr4	187070791	187070791	C	G	exonic	FAM149A		nonsynonymous SNV	FAM149A:NM_001367768:exon3:c.C722G	NOVEL		284	0	0	1	152	8	0.117025	1
4	chr2	56149499	56149499	T	A	exonic	EFEMP1		nonsynonymous SNV	EFEMP1:NM_001039349:exon2:c.A77T:p.Y	NOVEL		324	0	0	1	199	10	0.111986	1
4	chr22	46653617	46653617	T	A	exonic	PKDREJ		nonsynonymous SNV	PKDREJ:NM_006071:exon1:c.A5603T:p.Y18	NOVEL		300	0	0	1	169	9	0.067778	1
4	chr2	230633383	230633383	G	A	exonic	TRIP12		stopgain	TRIP12:NM_001284216:exon38:c.C4921T:	NOVEL		275	0	0	1	54	7	0.0652534	1
4	chr7	124532325	124532325	C	T	exonic	POT1		nonsynonymous SNV	POT1:NM_015450:exon6:c.G119A:p.G40E	COSMIC	ID=COSM474592	301	153	0.68556	1	263	0	0	1
4	chr9	128069812	128069812	C	T	exonic	GAPVD1		stopgain	GAPVD1:NM_001282681:exon5:c.C1237T:	NOVEL		170	80	0.650968	1	120	0	0	1
4	chr8	55540293	55540293	A	T	exonic	RP1		nonsynonymous SNV	RP1:NM_006269:exon4:c.A3851T:p.D1284	NOVEL		499	152	0.474977	1	393	0	0	1
4	chr19	3613526	3613526	G	A	exonic	CACTIN		stopgain	CACTIN:NM_001080543:exon8:c.C1414T:p	NOVEL		173	18	0.280493	1	95	0	0	1
4	chr3	57131817	57131817	G	A	exonic	IL17RD		synonymous SNV	IL17RD:NM_017563:exon12:c.C1914T:p.D6	DBSNP+COSMIC	ID=COSM690795	245	28	0.208644	1	150	0	0	1
4	chr15	70349802	70349802	C	T	exonic	TLE3		nonsynonymous SNV	TLE3:NM_001105192:exon13:c.G1247A:p.	NOVEL		158	16	0.18706	1	124	0	0	1
4	chr7	130357670	130357670	C	T	exonic	TSGA13		nonsynonymous SNV	TSGA13:NM_052933:exon6:c.G434A:p.R14	DBSNP+COSMIC	ID=COSM367479	296	28	0.175802	1	201	0	0	1
4	chr7	45719361	45719361	G	T	exonic	ADCY1		nonsynonymous SNV	ADCY1:NM_021116:exon11:c.G1952T:p.C6	NOVEL		330	18	0.105221	1	254	0	0	1
4	chr1	108299898	108299898	A	G	exonic	VAV3		synonymous SNV	VAV3:NM_006113:exon11:c.T1071C:p.A35	DBSNP		103	9	0.0831114	1	60	0	0	1
4	chr14	101200501	101200501	C	T	exonic	DLK1		synonymous SNV	DLK1:NM_001317172:exon5:c.C420T:p.H14	DBSNP		286	11	0.0753435	1	208	0	0	1
4	chr7	4917367	4917367	C	T	exonic	RADIL		nonsynonymous SNV	RADIL:NM_018059:exon2:c.G404A:p.G135	NOVEL		379	13	0.0674632	1	270	0	0	1
4	chr1	91866577	91866577	T	A	exonic	HFM1		nonsynonymous SNV	HFM1:NM_001017975:exon2:c.A63T:p.E21	NOVEL		127	6	0.046659	1	82	0	0	1
4	chr2	32693852	32693852	G	T	splicing	BIRC6	NM_016252:exon29:c.			NOVEL		12	52	0.840349	1	43	0	0	1
5	chr1	32150441	32150441	C	T	exonic	COL16A1		nonsynonymous SNV	COL16A1:NM_001856:exon30:c.G2033A:p	DBSNP		75	33	0.890189	1	74	58	1.26242	1
5	chr11	64108145	64108145	G	A	exonic	CCDC88B		nonsynonymous SNV	CCDC88B:NM_032251:exon2:c.G127A:p.E4	NOVEL		85	81	1.1967	2	136	63	1.22616	1
5	chr19	11132501	11132501	G	A	exonic	SMARCA4		nonsynonymous SNV	SMARCA4:NM_001128845:exon18:c.G271	COSMIC	ID=COSM557600	233	97	1.15029	1	232	103	1.19083	1
5	chr2	121742319	121742319	G	C	exonic	GLI2		synonymous SNV	GLI2:NM_001371271:exon12:c.G1956C:p.(	NOVEL		63	33	1.00146	1	54	38	1.18671	1
5	chr17	39580351	39580351	C	A	exonic	KRT37		nonsynonymous SNV	KRT37:NM_003770:exon1:c.G425T:p.C142	NOVEL		246	157	1.13498	1	255	177	1.17717	1
5	chr1	43897541	43897541	G	T	exonic	SZT2		nonsynonymous SNV	SZT2:NM_015284:exon35:c.G5072T:p.G16	NOVEL		65	0	0	1	28	45	1.15465	1
5	chr3	182755119	182755119	T	C	exonic	MCCC1		nonsynonymous SNV	MCCC1:NM_001293273:exon11:c.A1130G:	NOVEL		166	1	0.0174452	1	90	58	1.12594	1
5	chr6	167571176	167571176	C	T	exonic	GPR31		synonymous SNV	GPR31:NM_005299:exon1:c.G144A:p.P48P	DBSNP+COSMIC	ID=COSM107595	49	57	1.0555	1	99	63	1.11731	1
5	chr6	167717547	167717547	C	T	exonic	UNC93A		nonsynonymous SNV	UNC93A:NM_001143947:exon4:c.C640T:p	NOVEL		111	106	0.958816	1	178	113	1.11567	1
5	chr11	13031593	13031593	C	A	exonic	RASSF10		nonsynonymous SNV	RASSF10:NM_001080521:exon1:c.C470A:p	NOVEL		321	5	0.0446832	1	309	123	1.10276	1
5	chr1	113655242	113655242	G	T	exonic	LRIG2		nonsynonymous SNV	LRIG2:NM_014813:exon14:c.G1940T:p.R6	COSMIC	ID=COSM621371	235	7	0.0558255	1	98	140	1.10182	1
5	chrX	107978952	107978952	G	A	exonic	IRS4		nonsynonymous SNV	IRS4:NM_003604:exon1:c.C623T:p.T208M	DBSNP		286	185	1.14431	1	283	176	1.10167	1
5	chr10	127753535	127753535	G	A	exonic	ADAM12		synonymous SNV	ADAM12:NM_001288973:exon14:c.C1449T	NOVEL		50	25	0.971116	1	52	32	1.09451	1
5	chr3	47041486	47041486	C	T	exonic	NBEAL2		synonymous SNV	NBEAL2:NM_001365116:exon26:c.C3795T	DBSNP		247	127	0.989292	1	271	165	1.08729	1
5	chr12	25398255	25398255	G	T	exonic	KRAS		nonsynonymous SNV	KRAS:NM_001369786:exon2:c.C64A:p.Q22	DBSNP+COSMIC	ID=COSM543,CO	116	53	0.913653	1	116	69	1.07159	1
5	chr10	410518	410518	A	T	exonic	DIP2C		nonsynonymous SNV	DIP2C:NM_014974:exon20:c.T2273A:p.F75	NOVEL		148	79	1.0139	1	150	89	1.0699	1
5	chr7	132174144	132174144	C	T	exonic	PLXNA4		synonymous SNV	PLXNA4:NM_001105543:exon3:c.G1278A:	DBSNP		129	1	0.0224104	1	78	46	1.06582	1
5	chr3	196134159	196134159	C	G	exonic	UBXN7		nonsynonymous SNV	UBXN7:NM_015562:exon2:c.G179C:p.S60T	NOVEL		349	167	0.942886	1	326	192	1.06493	1
5	chr13	70281903	70281903	T	C	exonic	KLHL1		nonsynonymous SNV	KLHL1:NM_001286725:exon9:c.A1858G:p.	NOVEL		203	73	0.770559	1	179	105	1.06224	1
5	chr19	57985327	57985327	C	T	exonic	ZNF772		nonsynonymous SNV	ZNF772:NM_001330613:exon3:c.G449A:p	NOVEL		282	1	0.0138281	1	140	82	1.06123	1
5	chr7	4259801	4259801	G	A	exonic	SDK1		nonsynonymous SNV	SDK1:NM_001079653:exon13:c.G1061A:p.	DBSNP		157	84	1.01544	1	161	94	1.0591	1
5	chr11	73686682	73686682	C	T	exonic	UCP2		synonymous SNV	UCP2:NM_003355:exon7:c.G669A:p.G223	NOVEL		95	121	1.37385	2	88	105	1.05356	2
5	chr22	36902070	36902070	C	G	exonic	FOXRED2		nonsynonymous SNV	FOXRED2:NM_001102371:exon2:c.G400C:	NOVEL		200	109	1.02769	1	232	134	1.0519	1
5	chr1	39340546	39340546	A	T	exonic	GJA9		nonsynonymous SNV	GJA9:NM_030772:exon2:c.T1225A:p.F409	NOVEL		321	3	0.0269754	1	196	112	1.04476	1
5	chr17	40094922	40094922	C	T	exonic	TTC25		unknown	UNKNOWN	DBSNP+COSMIC	ID=COSM258263	212	2	0.0272275	1	169	96	1.04082	1
5	chr1	161042582	161042582	C	T	exonic	NECTIN4		nonsynonymous SNV	NECTIN4:NM_030916:exon9:c.G1402A:p.A	NOVEL		313	3	0.0276584	1	216	122	1.03703	1
5	chr7	141341122	141341122	G	T	exonic	AGK		nonsynonymous SNV	AGK:NM_001364948:exon12:c.G801T:p.E2	NOVEL		301	3	0.0287501	1	190	107	1.03509	1
5	chr12	58109584	58109584	C	T	exonic	OS9		synonymous SNV	OS9:NM_001261421:exon5:c.C522T:p.I174	DBSNP+COSMIC	ID=COSM471034	256	159	1.1162	1	285	160	1.03302	1
5	chr1	45271901	45271901	C	T	exonic	TCTEX1D4		nonsynonymous SNV	TCTEX1D4:NM_001013632:exon2:c.G440A	DBSNP		60	43	0.798776	1	56	68	1.02718	1
5	chr11	118940966	118940966	A	C	exonic	VPS11		unknown	UNKNOWN	NOVEL		333	72	0.871998	1	308	111	1.02605	1
5	chr8	22006247	22006247	C	T	exonic	LGI3		nonsynonymous SNV	LGI3:NM_139278:exon8:c.G1073A:p.R358	COSMIC	ID=COSM716184	91	68	1.24596	1	110	61	1.0249	1
5	chr6	170597531	170597531	C	A	exonic	DLL1		nonsynonymous SNV	DLL1:NM_005618:exon4:c.G466T:p.G156C	NOVEL		202	5	0.0474121	1	207	114	1.02035	1
5	chr15	67768039	67768039	G	C	exonic	IQCH		synonymous SNV	IQCH:NM_001284347:exon13:c.G1653C:p.	NOVEL		166	0	0	1	174	46	1.01813	1
5	chr7	98574287	98574287	C	T	exonic	TRRAP		nonsynonymous SNV	TRRAP:NM_003496:exon53:c.C8066T:p.T2	NOVEL		258	130	0.976121	1	272	149	1.01684	1
5	chrX	135291549	135291549	G	T	exonic	FHL1		nonsynonymous SNV	FHL1:NM_001159702:exon7:c.G836T:p.W2	NOVEL		104	0	0	1	81	44	1.01133	1
5	chr8	2832075	2832075	C	T	exonic	CSMD1		nonsynonymous SNV	CSMD1:NM_033225:exon56:c.G8638A:p.V	DBSNP+COSMIC	ID=COSM487373	196	4	0.0582669	1	128	69	1.00631	1
5	chr17	46675444	46675444	G	A	exonic	HOXB6		synonymous SNV	HOXB6:NM_001369397:exon2:c.C69T:p.G2	NOVEL		167	3	0.051412	1	123	66	1.0033	1
5	chr19	11453642	11453642	T	A	exonic	TMEM205		nonsynonymous SNV	TMEM205:NM_198536:exon3:c.A419T:p.Y	NOVEL		249	1	0.0156534	1	212	74	1.00213	1
5	chr19	42841325	42841325	G	T	exonic	MEGF8		stopgain	MEGF8:NM_001271938:exon8:c.G1480T:p	NOVEL		90	30	0.978337	1	73	39	1.00045	1
5	chr9	116131995	116131995	C	G	exonic	BSPRY		nonsynonymous SNV	BSPRY:NM_001317943:exon6:c.C797G:p.A	NOVEL		571	3	0.020453	1	494	171	0.995938	1
5	chr20	36869530	36869530	G	T	exonic	KIAA1755		synonymous SNV	KIAA1755:NM_001029864:exon3:c.C1003A	NOVEL		248	2	0.0233068	1	164	87	0.995853	1
5	chr2	219506784	219506784	G	A	exonic	ZNF142		synonymous SNV	ZNF142:NM_001366291:exon7:c.C4455T:p	DBSNP		187	1	0.0154965	1	134	71	0.995071	1
5	chr21	38522462	38522462	A	G	exonic	TTC3		nonsynonymous SNV	TTC3:NM_001330681:exon15:c.A1273G:p.	NOVEL		178	32	0.59632	1	152	52	0.987259	1
5	chr14	23828979	23828979	G	C	exonic	EFS		synonymous SNV	EFS:NM_032459:exon3:c.C429G:p.P143P,E	NOVEL		225	2	0.0256683	1	157	82	0.985747	1
5	chr17	77921596	77921596	C	G	exonic	TBC1D16		nonsynonymous SNV	TBC1D16:NM_001271844:exon5:c.G451C:	NOVEL		307	6	0.0558469	1	213	111	0.9843	1
5	chr7	70597813	70597813	G	A	exonic	GALNT17		nonsynonymous SNV	GALNT17:NM_022479:exon1:c.G25A:p.V9	NOVEL		272	120	0.891841	1	290	150	0.979464	1
5	chr5	3600105	3600105	C	T	exonic	IRX1		nonsynonymous SNV	IRX1:NM_024337:exon2:c.C1043T:p.A348V	COSMIC	ID=COSM740135	367	12	0.123905	1	302	203	0.979443	2
5	chr7	83675749	83675749	T	A	exonic	SEMA3A		nonsynonymous SNV	SEMA3A:NM_006080:exon6:c.A558T:p.L18	NOVEL		225	107	0.93894	1	244	126	0.978405	1
5	chr12	78513645	78513645	C	T	exonic	NAV3		synonymous SNV	NAV3:NM_001024383:exon15:c.C3669T:p.	NOVEL		278	94	0.736168	1	291	150	0.977243	1
5	chr1	57535075	57535075	G	A	exonic	DAB1		synonymous SNV	DAB1:NM_001353983:exon8:c.C621T:p.H2	DBSNP		183	6	0.062313	1	84	91	0.974008	1
5	chr1	235967872	235967872	C	T	exonic	LYST		nonsynonymous SNV	LYST:NM_000081:exon7:c.G3487A:p.A116	NOVEL		328	0	0	1	260	133	0.972319	1
5	chr22	26117345	26117345	A	C	exonic	GRK3		nonsynonymous SNV	GRK3:NM_001362778:exon19:c.A1547C:p.	NOVEL		294	6	0.0582669	1	228	116	0.968834	1
5	chr20	59829905	59829905	T	A	exonic	CDH4		synonymous SNV	CDH4:NM_001794:exon2:c.T81A:p.T27T	NOVEL		357	6	0.0481545	1	246	125	0.968023	1
5	chr14	75329467	75329467	G	A	exonic	PROX2		synonymous SNV	PROX2:NM_001243007:exon1:c.C1071T:p.	NOVEL		449	2	0.0129195	1	272	138	0.967041	1
5	chr17	59445767	59445767	C	G	exonic	BCAS3		synonymous SNV	BCAS3:NM_001320470:exon23:c.C2505G:	NOVEL		202	0	0	1	127	64	0.962712	1
5	chr1	154989075	154989075	G	T	exonic	ZBTB7B		nonsynonymous SNV	ZBTB7B:NM_001256455:exon3:c.G1534T:	NOVEL		161	0	0	1	137	69	0.962347	1
5	chr18	67993143	67993143	C	T	exonic	SOCS6		synonymous SNV	SOCS6:NM_004232:exon2:c.C1239T:p.D41	DBSNP		156	74	0.937338	1	141	71	0.962215	1
5	chr17	78082526	78082526	G	A	exonic	GAA		nonsynonymous SNV	GAA:NM_000152:exon8:c.G1225A:p.D409	NOVEL		126	62	0.960785	1	126	63	0.957698	1
5	chr6	41755451	41755451	G	C	exonic	TOMM6		nonsynonymous SNV	TOMM6:NM_001134493:exon1:c.G16C:p.V	NOVEL		163	3	0.0526508	1	128	64	0.957698	1
5	chr21	47860882	47860882	A	G	exonic	PCNT		nonsynonymous SNV	PCNT:NM_001315529:exon43:c.A8917G:p	NOVEL		419	156	1.06171	1	431	140	0.94962	1
5	chr19	7595254	7595254	A	C	exonic	MCOLN1		nonsynonymous SNV	MCOLN1:NM_020533:exon12:c.A1442C:p.	NOVEL		591	10	0.0651139	1	472	301	0.948772	2
5	chr15	56388054	56388054	T	G	exonic	RFX7		synonymous SNV	RFX7:NM_001368073:exon9:c.A1581C:p.L5	NOVEL		1101	7	0.0247233	1	815	520	0.948328	2
5	chr19	11569392	11569392	C	T	exonic	ELAVL3		nonsynonymous SNV	ELAVL3:NM_001420:exon4:c.G368A:p.R12	DBSNP+COSMIC	ID=COSM665317	251	95	1.07447	1	293	95	0.948309	1
5	chr11	65305936	65305936	C	T	exonic	SCYL1		nonsynonymous SNV	SCYL1:NM_001048218:exon18:c.C2275T:p	DBSNP		420	2	0.0232464	1	286	92	0.942658	1
5	chr18	54354105	54354105	A	T	exonic	WDR7		nonsynonymous SNV	WDR7:NM_015285:exon7:c.A617T:p.E206	NOVEL		197	2	0.0292799	1	125	60	0.931814	1
5	chr7	99764169	99764169	G	A	exonic	GAL3ST4		nonsynonymous SNV	GAL3ST4:NM_024637:exon3:c.C385T:p.P1	NOVEL		291	3	0.029728	1	219	105	0.931095	1
5	chr5	59064264	59064264	C	T	exonic	PDE4D		stopgain	PDE4D:NM_001197218:exon1:c.G72A:p.W	NOVEL		575	10	0.0668948	1	318	152	0.929171	1
5	chr3	12645693	12645693	G	A	exonic	RAF1		nonsynonymous SNV	RAF1:NM_001354695:exon5:c.C434T:p.S1	DBSNP+COSMIC	ID=COSM153653	136	0	0	1	103	49	0.926194	1
5	chr4	15709249	15709249	G	T	exonic	BST1		nonsynonymous SNV	BST1:NM_004334:exon3:c.G431T:p.C144F	NOVEL		398	2	0.0145667	1	272	129	0.924262	1
5	chr7	107866726	107866726	T	A	exonic	NRCAM		nonsynonymous SNV	NRCAM:NM_001371180:exon6:c.A359T:p.	NOVEL		372	1	0.00781058	1	262	124	0.922963	1
5	chrX	21613144	21613144	C	A	exonic	CNKSR2		nonsynonymous SNV	CNKSR2:NM_001330771:exon14:c.C1646A	NOVEL		108	39	0.772929	1	119	56	0.91939	1
5	chr19	56369427	56369427	C	T	exonic	NLRP4		nonsynonymous SNV	NLRP4:NM_134444:exon3:c.C668T:p.P223	NOVEL		387	118	0.914406	1	284	133	0.916358	1
5	chr15	84651924	84651924	C	T	exonic	ADAMTSL3		stopgain	ADAMTSL3:NM_001301110:exon21:c.C354	NOVEL		786	14	0.0684836	1	430	386	0.916062	2
5	chr17	49118872	49118872	G	A	exonic	SPAG9		nonsynonymous SNV	SPAG9:NM_001251971:exon2:c.C302T:p.T	NOVEL		390	5	0.0368778	1	265	124	0.915845	1
5	chr10	102689689	102689689	G	A	exonic	SLF2		synonymous SNV	SLF2:NM_001136123:exon8:c.G2268A:p.Q	NOVEL		183	3	0.0469895	1	120	56	0.914166	1
5	chr2	219677043	219677043	T	C	exonic	CYP27A1		nonsynonymous SNV	CYP27A1:NM_000784:exon3:c.T545C:p.I18	DBSNP		484	5	0.0297888	1	355	165	0.911655	1
5	chr11	64418098	64418098	G	A	exonic	NRXN2		synonymous SNV	NRXN2:NM_138732:exon13:c.C2811T:p.Y9	NOVEL		644	0	0	1	442	136	0.911316	1
5	chr12	48963696	48963696	A	G	exonic	LALBA		synonymous SNV	LALBA:NM_002289:exon1:c.T108C:p.G36G	DBSNP		197	2	0.0292799	1	136	63	0.909572	1
5	chr12	65004446	65004446	G	A	exonic	RASSF3		nonsynonymous SNV	RASSF3:NM_178169:exon1:c.G34A:p.A12T	NOVEL		170	2	0.0338761	1	145	67	0.908006	1
5	chr12	80943452	80943452	T	G	exonic	PTPRQ		synonymous SNV	PTPRQ:NM_001145026:exon22:c.T3708G:	NOVEL		304	119	0.819594	1	323	149	0.906972	1
5	chr5	52370894	52370894	A	T	exonic	ITGA2		nonsynonymous SNV	ITGA2:NM_002203:exon22:c.A2668T:p.T89	NOVEL		97	0	0	1	56	17	0.901953	1
5	chr4	6711192	6711192	G	A	exonic	MRFAP1L1		synonymous SNV	MRFAP1L1:NM_203462:exon1:c.C165T:p.S	NOVEL		306	1	0.00948973	1	206	94	0.900236	1
5	chr3	74383900	74383900	C	G	exonic	CNTN3		nonsynonymous SNV	CNTN3:NM_020872:exon12:c.G1654C:p.E5	NOVEL		278	1	0.0104421	1	194	88	0.896568	1
5	chr1	205693069	205693069	C	T	exonic	NUCKS1		nonsynonymous SNV	NUCKS1:NM_022731:exon4:c.G214A:p.D7	NOVEL		107	1	0.0269754	1	75	34	0.896194	1
5	chr2	173427517	173427517	C	T	exonic	PDK1		synonymous SNV	PDK1:NM_001278549:exon4:c.C459T:p.A1	NOVEL		64	34	1.01075	1	80	36	0.89165	1
5	chr12	8610535	8610535	G	A	exonic	CLEC6A		nonsynonymous SNV	CLEC6A:NM_001007033:exon2:c.G73A:p.G	NOVEL		177	3	0.0485558	1	171	76	0.884029	1
5	chr16	25255514	25255514	G	C	exonic	ZKSCAN2		nonsynonymous SNV	ZKSCAN2:NM_001012981:exon6:c.C1573G	NOVEL		261	4	0.043975	1	189	84	0.884029	1
5	chr12	9251315	9251315	G	T	exonic	A2M		nonsynonymous SNV	A2M:NM_001347425:exon14:c.C1289A:p.A	COSMIC	ID=COSM465780	161	0	0	1	126	56	0.884029	1
5	chr20	36953181	36953181	A	G	exonic	BPI		synonymous SNV	BPI:NM_001725:exon9:c.A951G:p.P317P	NOVEL		230	2	0.0251151	1	160	71	0.883072	1
5	chr2	61510552	61510552	C	T	exonic	USP34		nonsynonymous SNV	USP34:NM_014709:exon36:c.G4865A:p.R1	NOVEL		116	6	0.143279	1	95	42	0.880802	1
5	chr6	10704819	10704819	T	G	exonic	PAK1IP1		synonymous SNV	PAK1IP1:NM_017906:exon6:c.T576G:p.L19	NOVEL		348	5	0.0412655	1	237	103	0.870378	1
5	chr15	34147054	34147054	G	A	exonic	RYR3		nonsynonymous SNV	RYR3:NM_001243996:exon97:c.G13933A:	DBSNP+COSMIC	ID=COSM622923	630	2	0.012384	1	529	152	0.864479	1
5	chr15	22947021	22947021	G	C	exonic	CYFIP1		nonsynonymous SNV	CYFIP1:NM_001324125:exon10:c.G928C:p	DBSNP		469	2	0.0166172	1	439	126	0.863734	1
5	chr10	48438600	48438600	G	A	exonic	GDF10		synonymous SNV	GDF10:NM_004962:exon1:c.C111T:p.A37A	DBSNP		104	3	0.0816826	1	117	49	0.848082	1
5	chr7	117232156	117232156	G	A	exonic	CFTR		nonsynonymous SNV	CFTR:NM_000492:exon14:c.G1935A:p.M6	NOVEL		419	4	0.0275494	1	338	140	0.841492	1
5	chr18	21508689	21508689	C	T	exonic	LAMA3		nonsynonymous SNV	LAMA3:NM_001127718:exon26:c.C3401T:	NOVEL		481	6	0.0358934	1	340	140	0.837985	1
5	chr4	47944147	47944147	C	A	exonic	CNGA1		synonymous SNV	CNGA1:NM_001142564:exon8:c.G675T:p.V	NOVEL		367	3	0.0236217	1	246	100	0.830374	1
5	chr16	30977292	30977292	A	C	exonic	SETD1A		nonsynonymous SNV	SETD1A:NM_014712:exon8:c.A2090C:p.K6	NOVEL		265	1	0.0109524	1	219	89	0.830212	1
5	chr15	33218415	33218415	C	G	exonic	FMN1		nonsynonymous SNV	FMN1:NM_001103184:exon6:c.G2517C:p.	NOVEL		236	0	0	1	170	46	0.824825	1
5	chr18	59828478	59828478	T	A	exonic	PIGN		nonsynonymous SNV	PIGN:NM_012327:exon3:c.A109T:p.T37S,F	NOVEL		258	6	0.0662124	1	214	86	0.82362	1
5	chr9	136531916	136531916	C	A	exonic	SARDH		nonsynonymous SNV	SARDH:NM_001134707:exon20:c.G2572T:	COSMIC	ID=COSM566726	487	2	0.0160055	1	479	129	0.821758	1
5	chr2	153001452	153001452	T	A	exonic	STAM2		nonsynonymous SNV	STAM2:NM_005843:exon6:c.A467T:p.K156	NOVEL		215	2	0.0268511	1	180	72	0.820884	1
5	chr19	23542498	23542498	T	C	exonic	ZNF91		nonsynonymous SNV	ZNF91:NM_001300951:exon3:c.A3187G:p	NOVEL		476	3	0.0245095	1	385	196	0.811747	2
5	chr19	33182974	33182974	G	A	exonic	NUDT19		synonymous SNV	NUDT19:NM_001105570:exon1:c.G108A:p	NOVEL		99	1	0.0391335	1	127	17	0.811407	1
5	chr22	50893488	50893488	G	C	exonic	SBF1		nonsynonymous SNV	SBF1:NM_001365819:exon33:c.C4563G:p.	COSMIC	ID=COSM483275	220	5	0.064741	1	180	70	0.804466	1
5	chr4	128716972	128716972	G	T	exonic	HSPA4L		nonsynonymous SNV	HSPA4L:NM_001317382:exon3:c.G137T:p.	COSMIC	ID=COSM573709	651	2	0.00892296	1	451	171	0.78987	1
5	chr15	67457632	67457632	G	T	exonic	SMAD3		nonsynonymous SNV	SMAD3:NM_001145102:exon3:c.G127T:p.	NOVEL		763	5	0.0254775	1	851	162	0.778703	1
5	chr19	17392894	17392894	G	T	exonic	ANKLE1		nonsynonymous SNV	ANKLE1:NM_001278443:exon2:c.G58T:p.A	NOVEL		151	1	0.0257457	1	155	37	0.746377	1
5	chr7	72987717	72987717	T	G	exonic	TBL2		synonymous SNV	TBL2:NM_001362661:exon4:c.A36C:p.T12	NOVEL		191	0	0	1	174	61	0.745782	1
5	chr11	88042423	88042423	C	A	exonic	CTSC		nonsynonymous SNV	CTSC:NM_001814:exon4:c.G549T:p.K183N	NOVEL		757	3	0.0193948	1	482	106	0.698211	1
5	chr19	44981130	44981130	C	A	exonic	ZNF180		nonsynonymous SNV	ZNF180:NM_001278508:exon4:c.G1493T:	NOVEL		272	1	0.0143346	1	156	50	0.697353	1
5	chr5	140166131	140166131	G	C	exonic	PCDHA1		nonsynonymous SNV	PCDHA1:NM_018900:exon1:c.G256C:p.V86	NOVEL		153	42	0.842875	1	91	8	0.151361	1
5	chr7	2978455	2978455	C	A	exonic	CARD11		nonsynonymous SNV	CARD11:NM_032415:exon7:c.G875T:p.R29	NOVEL		198	0	0	1	202	11	0.148376	1
5	chr17	27061925	27061925	A	C	exonic	NEK8		nonsynonymous SNV	NEK8:NM_178170:exon3:c.A389C:p.K130T	NOVEL		374	0	0	1	439	23	0.143033	1
5	chr17	19843117	19843117	C	T	exonic	AKAP10		nonsynonymous SNV	AKAP10:NM_001330152:exon8:c.G1231A:	NOVEL		246	0	0	1	261	13	0.136315	1
5	chr8	145165834	145165834	G	A	exonic	WDR97		nonsynonymous SNV	WDR97:NM_001316309:exon9:c.G2239A:	NOVEL		252	0	0	1	241	12	0.136273	1
5	chr21	45919773	45919773	C	T	exonic	TSPEAR		nonsynonymous SNV	TSPEAR:NM_144991:exon12:c.G1903A:p.V	DBSNP		92	30	0.962298	1	113	4	0.132413	1
5	chr15	81585074	81585074	C	G	exonic	IL16		nonsynonymous SNV	IL16:NM_001172128:exon12:c.C1598G:p.P	NOVEL		157	41	0.81034	1	199	7	0.13161	1
5	chr10	48389821	48389821	G	A	exonic	RBP3		nonsynonymous SNV	RBP3:NM_002900:exon1:c.C1057T:p.H353	NOVEL		392	0	0	1	357	16	0.123243	1
5	chr2	237074981	237074981	C	A	exonic	GBX2		nonsynonymous SNV	GBX2:NM_001485:exon2:c.G623T:p.S208I	NOVEL		395	0	0	1	385	17	0.121499	1
5	chrX	57318951	57318951	T	G	exonic	FAAH2		synonymous SNV	FAAH2:NM_001353840:exon2:c.T213G:p.V	NOVEL		419	0	0	1	399	17	0.11741	1
5	chr11	46789174	46789174	T	G	exonic	CKAP5		nonsynonymous SNV	CKAP5:NM_001008938:exon27:c.A3362C:p	NOVEL		145	52	0.769005	1	293	9	0.115423	1
5	chr10	81060489	81060489	G	T	exonic	ZMIZ1		nonsynonymous SNV	ZMIZ1:NM_020338:exon17:c.G1809T:p.R6	NOVEL		31	15	0.950004	1	48	2	0.114924	1
5	chr13	86369739	86369739	G	A	exonic	SLITRK6		nonsynonymous SNV	SLITRK6:NM_032229:exon2:c.C905T:p.S30	NOVEL		284	103	0.775387	1	261	16	0.114887	1
5	chr4	154868	154868	T	G	exonic	ZNF718		nonsynonymous SNV	ZNF718:NM_001039127:exon5:c.T393G:p.	NOVEL		260	0	0	1	280	11	0.108605	1
5	chrX	53349967	53349967	G	A	exonic	IQSEC2		nonsynonymous SNV	IQSEC2:NM_001111125:exon1:c.C355T:p.R	NOVEL		135	60	0.896414	1	207	8	0.106906	1
5	chr13	103402258	103402258	A	C	exonic	CCDC168		synonymous SNV	CCDC168:NM_001146197:exon4:c.T789G:	NOVEL		391	1	0.00743201	1	300	17	0.106665	1
5	chr10	123325060	123325060	C	T	exonic	FGFR2		nonsynonymous SNV	FGFR2:NM_001144913:exon2:c.G268A:p.E	NOVEL		305	141	0.921036	1	458	17	0.102826	1
5	chr1	26601497	26601497	G	A	exonic	CEP85		nonsynonymous SNV	CEP85:NM_001281518:exon5:c.G133A:p.A	NOVEL		139	85	1.10551	1	222	8	0.0999337	1
5	chr15	66824660	66824660	C	A	exonic	ZWILCH		nonsynonymous SNV	ZWILCH:NM_001287822:exon12:c.C889A:p	NOVEL		343	100	0.883374	1	539	11	0.0973859	1
5	chr19	9068198	9068198	C	A	exonic	MUC16		nonsynonymous SNV	MUC16:NM_024690:exon3:c.G19248T:p.K	DBSNP		358	121	0.988549	1	471	12	0.0962259	1
5	chr5	167553835	167553835	C	T	exonic	TENM2		synonymous SNV	TENM2:NM_001080428:exon9:c.C1590T:p	DBSNP		112	43	1.08564	1	111	6	0.0960561	1
5	chr21	35468269	35468269	G	T	exonic	SLC5A3		nonsynonymous SNV	SLC5A3:NM_006933:exon2:c.G772T:p.D25	NOVEL		465	131	0.860148	1	675	17	0.0951482	1
5	chr11	126174181	126174181	C	G	exonic	DCPS		nonsynonymous SNV	DCPS:NM_001350236:exon1:c.C206G:p.P6	NOVEL		210	51	0.958446	1	242	6	0.0937039	1
5	chr4	48567637	48567637	C	T	exonic	FRYL		nonsynonymous SNV	FRYL:NM_015030:exon29:c.G3241A:p.A10	NOVEL		202	76	0.796455	1	241	8	0.0923082	1
5	chr8	27297857	27297857	G	T	exonic	PTK2B		nonsynonymous SNV	PTK2B:NM_173175:exon21:c.G1938T:p.K6	NOVEL		88	32	0.776893	1	126	4	0.0884029	1
5	chr19	35510296	35510296	C	T	exonic	GRAMD1A		nonsynonymous SNV	GRAMD1A:NM_001136199:exon12:c.C131	DBSNP		269	89	0.972871	1	502	9	0.0858275	1
5	chr4	184618704	184618704	A	G	exonic	TRAPPC11		nonsynonymous SNV	TRAPPC11:NM_021942:exon24:c.A2677G:	NOVEL		158	94	1.08672	1	260	8	0.085764	1
5	chr2	198273139	198273139	C	A	exonic	SF3B1		nonsynonymous SNV	SF3B1:NM_012433:exon8:c.G1071T:p.K35	NOVEL		79	34	0.876582	1	130	4	0.085764	1
5	chr1	89847335	89847335	G	A	exonic	GBP6		synonymous SNV	GBP6:NM_001320257:exon5:c.G564A:p.L1	NOVEL		74	54	0.828082	1	150	7	0.0835137	1
5	chr2	241815405	241815405	C	T	exonic	AGXT		nonsynonymous SNV	AGXT:NM_000030:exon8:c.C830T:p.A277V	NOVEL		167	74	0.894555	1	269	8	0.0829774	1
5	chr1	152733214	152733214	C	A	exonic	KPRP		nonsynonymous SNV	KPRP:NM_001025231:exon2:c.C1150A:p.P	NOVEL		313	124	0.826671	1	409	12	0.0818934	1
5	chr15	36983920	36983920	C	A	exonic	C15orf41		nonsynonymous SNV	C15orf41:NM_001321758:exon4:c.C270A:p	NOVEL		704	189	0.828245	1	927	20	0.0817971	1
5	chr11	93430108	93430108	C	T	exonic	CEP295		nonsynonymous SNV	CEP295:NM_033395:exon15:c.C2030T:p.A	NOVEL		1022	268	1.02076	1	1077	23	0.0809829	1
5	chr17	8132648	8132648	C	T	exonic	CTC1		nonsynonymous SNV	CTC1:NM_025099:exon19:c.G3128A:p.C10	NOVEL		124	53	0.872358	1	210	6	0.0798081	1
5	chr6	117609725	117609725	G	A	exonic	ROS1		nonsynonymous SNV	ROS1:NM_002944:exon43:c.C6974T:p.P23	NOVEL		513	0	0	1	491	14	0.0796501	1
5	chr1	3410348	3410348	G	A	exonic	MEGF6		synonymous SNV	MEGF6:NM_001409:exon34:c.C4374T:p.A	NOVEL		106	79	1.24408	1	213	6	0.0787149	1
5	chr2	218700822	218700822	G	A	exonic	TNS1		synonymous SNV	TNS1:NM_001308022:exon17:c.C2745T:p.	NOVEL		89	59	1.1614	1	178	5	0.0784998	1
5	chr5	70806535	70806535	G	A	exonic	BDP1		nonsynonymous SNV	BDP1:NM_018429:exon17:c.G3616A:p.E12	NOVEL		1170	1	0.00334188	1	891	25	0.0784141	1
5	chr9	140357218	140357218	C	G	exonic	PNPLA7		synonymous SNV	PNPLA7:NM_152286:exon29:c.G3336C:p.T	NOVEL		253	96	1.07645	1	437	9	0.0781566	1
5	chr12	133146813	133146813	C	T	exonic	FBRSL1		synonymous SNV	FBRSL1:NM_001142641:exon7:c.C993T:p.(	DBSNP		61	27	0.893868	1	108	3	0.0776512	1
5	chr3	138121065	138121065	G	A	exonic	MRAS		nonsynonymous SNV	MRAS:NM_001252091:exon5:c.G353A:p.G	NOVEL		191	93	0.954019	1	293	8	0.0763613	1
5	chr16	67321017	67321017	A	C	exonic	PLEKHG4		nonsynonymous SNV	PLEKHG4:NM_001129731:exon16:c.A2797	NOVEL		129	59	0.914295	1	220	6	0.0762768	1
5	chr16	31341699	31341699	A	C	exonic	ITGAM		nonsynonymous SNV	ITGAM:NM_000632:exon27:c.A3131C:p.N	NOVEL		397	183	0.919211	1	677	18	0.074411	1
5	chr16	71712879	71712879	G	T	exonic	PHLPP2		synonymous SNV	PHLPP2:NM_001289003:exon8:c.C1047A:p	NOVEL		275	107	0.816042	1	377	10	0.0742401	1
5	chr16	71712878	71712878	G	T	exonic	PHLPP2		nonsynonymous SNV	PHLPP2:NM_001289003:exon8:c.C1048A:p	NOVEL		279	107	0.807586	1	381	10	0.0734806	1
5	chr3	169540070	169540070	C	T	exonic	LRRIQ4		nonsynonymous SNV	LRRIQ4:NM_001080460:exon1:c.C361T:p.R	NOVEL		507	213	0.861865	1	737	19	0.0722074	1
5	chr10	95385361	95385361	T	A	exonic	PDE6C		synonymous SNV	PDE6C:NM_006204:exon5:c.T894A:p.L298	NOVEL		230	115	0.971116	1	352	9	0.0716284	1
5	chr4	141315105	141315105	C	A	exonic	CLGN		stopgain	CLGN:NM_004362:exon11:c.G1240T:p.E41	NOVEL		183	83	0.909052	1	317	8	0.0707223	1
5	chr5	170737282	170737282	C	T	exonic	TLX3		nonsynonymous SNV	TLX3:NM_021025:exon2:c.C550T:p.R184C	NOVEL		152	55	1.03978	1	102	4	0.0706828	1
5	chr11	103057150	103057150	T	G	exonic	DYNC2H1		synonymous SNV	DYNC2H1:NM_001080463:exon42:c.T6813	NOVEL		548	125	0.912583	1	594	11	0.0704199	1
5	chr5	133725989	133725989	G	C	exonic	UBE2B		nonsynonymous SNV	UBE2B:NM_003337:exon6:c.G404C:p.R13	NOVEL		403	110	0.839119	1	290	11	0.0684519	1
5	chr5	93752992	93752992	C	A	exonic	KIAA0825		nonsynonymous SNV	KIAA0825:NM_001145678:exon15:c.G257	NOVEL		241	71	0.890537	1	267	6	0.0631449	1
5	chr19	57062038	57062038	A	T	exonic	ZFP28		nonsynonymous SNV	ZFP28:NM_001308440:exon7:c.A962T:p.K	NOVEL		150	49	0.963588	1	138	3	0.0611296	1
5	chr19	49364901	49364901	C	G	exonic	PLEKHA4		nonsynonymous SNV	PLEKHA4:NM_001161354:exon4:c.G241C:p	NOVEL		209	86	1.14084	1	281	6	0.0600647	1
5	chr5	36953859	36953859	G	A	exonic	NIPBL		nonsynonymous SNV	NIPBL:NM_015384:exon2:c.G61A:p.D21N,	NOVEL		357	99	0.849608	1	458	7	0.0583046	1
5	chr2	43783651	43783651	A	C	exonic	THADA		nonsynonymous SNV	THADA:NM_001083953:exon17:c.T2507G:	NOVEL		257	101	0.821922	1	392	8	0.0574619	1
5	chr2	169759058	169759058	G	C	exonic	G6PC2		nonsynonymous SNV	G6PC2:NM_001081686:exon2:c.G322C:p.G	NOVEL		413	179	0.880894	1	643	13	0.0569363	1
5	chr13	41515329	41515329	C	T	exonic	ELF1		synonymous SNV	ELF1:NM_001145353:exon7:c.G912A:p.R3	NOVEL		421	194	0.919007	1	624	12	0.0542093	1
5	chr17	56083796	56083796	C	T	exonic	SRSF1		nonsynonymous SNV	SRSF1:NM_001078166:exon2:c.G287A:p.G	NOVEL		124	70	1.05121	1	210	4	0.0537027	1
5	chr19	10224792	10224792	C	G	exonic	P2RY11;PPAN-P2R		nonsynonymous SNV	P2RY11:NM_002566:exon2:c.C503G:p.S16	NOVEL		383	101	0.816628	1	572	8	0.053422	1
5	chr13	45602019	45602019	G	T	exonic	GPALPP1		nonsynonymous SNV	GPALPP1:NM_001316951:exon8:c.G904T:	NOVEL		97	43	0.894814	1	159	3	0.0532054	1
5	chr1	186643626	186643626	C	G	exonic	PTGS2		synonymous SNV	PTGS2:NM_000963:exon10:c.G1674C:p.V5	NOVEL		471	197	0.859176	1	694	13	0.0528291	1
5	chr15	72691170	72691170	G	C	exonic	TMEM202		nonsynonymous SNV	TMEM202:NM_001080462:exon2:c.G258C	NOVEL		122	39	0.947954	1	152	2	0.0502999	1
5	chr8	133883698	133883698	C	G	exonic	TG		nonsynonymous SNV	TG:NM_003235:exon4:c.C380G:p.A127G	NOVEL		173	82	0.936841	1	289	5	0.0488621	1
5	chr19	39913733	39913733	C	T	exonic	PLEKHG2		nonsynonymous SNV	PLEKHG2:NM_001351693:exon18:c.C1862	COSMIC	ID=COSM632497	358	106	0.893997	1	465	8	0.0485935	1
5	chrX	70779204	70779204	G	C	exonic	OGT		nonsynonymous SNV	OGT:NM_181672:exon13:c.G1690C:p.D56	NOVEL		351	148	0.864079	1	533	9	0.0477082	1
5	chr19	44238612	44238612	G	C	exonic	SMG9		nonsynonymous SNV	SMG9:NM_019108:exon10:c.C1031G:p.T3	NOVEL		156	42	0.830104	1	201	3	0.0422514	1
5	chr11	123989780	123989780	G	A	exonic	VWA5A		nonsynonymous SNV	VWA5A:NM_014622:exon6:c.G744A:p.M2	NOVEL		239	66	1.06141	1	281	3	0.040913	1
5	chr11	5411592	5411592	A	C	exonic	OR51M1		nonsynonymous SNV	OR51M1:NM_001004756:exon1:c.A964C:p	NOVEL		89	58	1.14948	1	201	2	0.0381586	1
5	chr11	73141798	73141798	G	C	exonic	FAM168A		nonsynonymous SNV	FAM168A:NM_001286050:exon3:c.C88G:p	NOVEL		104	28	1.04045	1	133	1	0.0289037	1
5	chr5	140230307	140230307	A	T	exonic	PCDHA9		nonsynonymous SNV	PCDHA9:NM_014005:exon1:c.A2227T:p.S7	DBSNP		205	64	0.931057	1	158	2	0.0234137	1
5	chr1	55638055	55638055	T	G	exonic	USP24		nonsynonymous SNV	USP24:NM_015306:exon4:c.A697C:p.T233	NOVEL		218	9	0.0778226	1	237	3	0.0234137	1
5	chr11	59271227	59271227	A	C	exonic	OR4D11		nonsynonymous SNV	OR4D11:NM_001004706:exon1:c.A179C:p	NOVEL		539	142	1.02277	1	592	3	0.0195282	1
5	chr8	21979182	21979182	G	T	exonic	HR		nonsynonymous SNV	HR:NM_005144:exon9:c.C2147A:p.P716Q,	COSMIC	ID=COSM278576	135	8	0.162984	1	164	1	0.0174127	1
5	chr17	21217497	21217497	G	C	exonic	MAP2K3		synonymous SNV	MAP2K3:NM_002756:exon12:c.G912C:p.T	NOVEL		257	38	0.375279	1	371	2	0.0154053	1
5	chr9	96714526	96714526	T	C	exonic	BARX1		nonsynonymous SNV	BARX1:NM_021570:exon4:c.A685G:p.T229	NOVEL		410	20	0.182016	1	472	1	0.00818836	1
5	chr2	206874487	206874487	T	A	exonic	INO80D		nonsynonymous SNV	INO80D:NM_017759:exon9:c.A1574T:p.K5	NOVEL		88	37	0.862351	1	167	0	0	1
5	chr8	24253281	24253281	G	A	exonic	ADAMDEC1		nonsynonymous SNV	ADAMDEC1:NM_001145272:exon4:c.G175	NOVEL		88	6	0.185958	1	97	0	0	1
5	chr22	36681231	36681231	C	A	exonic	MYH9		nonsynonymous SNV	MYH9:NM_002473:exon38:c.G5419T:p.A1	COSMIC	ID=COSM480135	139	9	0.177163	1	130	0	0	1
5	chr20	377015	377015	C	G	exonic	TRIB3		nonsynonymous SNV	TRIB3:NM_001301188:exon4:c.C758G:p.T	NOVEL		108	6	0.153334	1	134	0	0	1
5	chr20	1219967	1219967	C	A	exonic	RAD21L1		nonsynonymous SNV	RAD21L1:NM_001136566:exon7:c.C658A:p	NOVEL		158	7	0.123597	1	189	0	0	1
5	chr12	25380275	25380275	T	C	exonic	KRAS		synonymous SNV	KRAS:NM_001369786:exon3:c.A183G:p.Q	COSMIC	ID=COSM732533	185	8	0.12076	1	198	0	0	1
5	chr20	1435734	1435734	C	A	exonic	NSFL1C		nonsynonymous SNV	NSFL1C:NM_016143:exon4:c.G322T:p.G10	NOVEL		192	8	0.116534	1	250	0	0	1
5	chr8	124357133	124357133	C	T	exonic	ATAD2		synonymous SNV	ATAD2:NM_014109:exon19:c.G2709A:p.L9	NOVEL		712	28	0.110235	1	780	0	0	1
5	chrX	63488522	63488522	C	A	exonic	MTMR8		nonsynonymous SNV	MTMR8:NM_017677:exon14:c.G2010T:p.L	NOVEL		223	8	0.100895	1	206	0	0	1
5	chr4	30724202	30724202	G	A	exonic	PCDH7		nonsynonymous SNV	PCDH7:NM_001173523:exon1:c.G1158A:p	NOVEL		671	20	0.0843226	1	700	0	0	1
5	chr5	161116271	161116271	A	T	exonic	GABRA6		nonsynonymous SNV	GABRA6:NM_000811:exon5:c.A458T:p.N1	NOVEL		973	21	0.0826763	1	598	0	0	1
5	chrX	153049206	153049206	G	A	splicing	SRPK3	NM_014370:exon9:c.7			NOVEL		41	17	0.853912	1	40	85	1.16843	2
5	chr1	227218172	227218172	C	A	splicing	CDC42BPA	NM_001366011:exon2			NOVEL		112	70	1.12052	1	125	71	1.04076	1
5	chr12	43840516	43840516	C	T	splicing	ADAMTS20	NM_025003:exon15:c.			NOVEL		428	3	0.0202785	1	332	156	0.918448	1
5	chr12	93236359	93236359	T	G	splicing	EEA1	NM_003566:exon10:c.			NOVEL		124	2	0.0462436	1	96	38	0.814758	1
5	chrX	148059986	148059986	G	C	splicing	AFF2	NM_001169122:exon1			NOVEL		265	115	0.881671	1	373	9	0.0676907	1
7	chr1	181690946	181690946	A	C	exonic	CACNA1E		nonsynonymous SNV	CACNA1E:NM_000721:exon16:c.A2009C:p	NOVEL		159	71	0.901216	1	178	53	0.655942	1
7	chr1	214814788	214814788	A	G	exonic	CENPF		nonsynonymous SNV	CENPF:NM_016343:exon12:c.A3107G:p.Y1	NOVEL		532	15	0.0916137	1	460	27	0.179515	1
7	chr10	5955730	5955730	A	G	exonic	FBH1		nonsynonymous SNV	FBH1:NM_001258452:exon7:c.A1010G:p.Y	NOVEL		105	89	1.07389	1	81	60	0.952309	1
7	chr10	96022375	96022375	G	T	exonic	PLCE1		synonymous SNV	PLCE1:NM_001165979:exon13:c.G3015T:p	NOVEL		112	98	1.0924	1	113	91	0.998291	1
7	chr11	212638	212638	G	A	exonic	RIC8A		synonymous SNV	RIC8A:NM_001286134:exon7:c.G1089A:p.	COSMIC	ID=COSM613224	16	131	1.1949	1	14	127	1.11501	1
7	chr11	2608824	2608824	G	A	exonic	KCNQ1		nonsynonymous SNV	KCNQ1:NM_000218:exon9:c.G1153A:p.E3	DBSNP		32	250	1.18869	1	21	203	1.12187	1
7	chr11	3681283	3681283	C	A	exonic	ART1		nonsynonymous SNV	ART1:NM_004314:exon3:c.C534A:p.F178L	NOVEL		43	295	1.17027	1	19	277	1.15847	1
7	chr11	18956268	18956268	G	C	exonic	MRGPRX1		nonsynonymous SNV	MRGPRX1:NM_147199:exon1:c.C64G:p.L2	COSMIC	ID=COSM613231	14	156	1.23042	1	9	178	1.17835	1
7	chr11	95571305	95571305	A	G	exonic	MTMR2		synonymous SNV	MTMR2:NM_016156:exon13:c.T1546C:p.L	NOVEL		132	106	1.04256	1	123	107	1.04112	1
7	chr12	32751475	32751475	G	A	exonic	FGD4		synonymous SNV	FGD4:NM_001370297:exon3:c.G93A:p.L31	NOVEL		122	99	1.04861	1	86	114	1.27562	1
7	chr14	59992092	59992092	G	A	exonic	CCDC175		nonsynonymous SNV	CCDC175:NM_001164399:exon16:c.C1903	COSMIC	ID=COSM554375	96	52	0.82246	1	89	24	0.475312	1
7	chr14	63749934	63749934	G	A	exonic	RHOJ		synonymous SNV	RHOJ:NM_020663:exon4:c.G498A:p.A166A	DBSNP+COSMIC	ID=COSM604279	304	253	1.06326	1	245	224	1.06886	1
7	chr14	74086475	74086475	C	T	exonic	ACOT6		stopgain	ACOT6:NM_001037162:exon2:c.C556T:p.Q	COSMIC	ID=COSM463998	198	141	0.973627	1	155	139	1.05807	1
7	chr14	74824052	74824052	C	A	exonic	VRTN		nonsynonymous SNV	VRTN:NM_018228:exon2:c.C566A:p.S189Y	NOVEL		416	316	1.01053	1	357	331	1.07668	1
7	chr14	96178653	96178653	T	C	exonic	TCL1A		nonsynonymous SNV	TCL1A:NM_001098725:exon2:c.A201G:p.I	NOVEL		124	88	0.971673	1	94	80	1.02893	1
7	chr15	26026240	26026240	G	A	exonic	ATP10A		nonsynonymous SNV	ATP10A:NM_024490:exon2:c.C580T:p.H19	COSMIC	ID=COSM350018	115	82	0.974363	1	71	75	1.14962	1
7	chr15	40727707	40727707	G	A	exonic	IVD		nonsynonymous SNV	IVD:NM_001354598:exon13:c.G1312A:p.E	NOVEL		142	119	1.06728	1	105	100	1.09167	1
7	chr15	75499387	75499387	C	T	exonic	C15orf39		nonsynonymous SNV	C15orf39:NM_015492:exon2:c.C998T:p.P3	DBSNP		154	99	0.915984	1	108	112	1.13931	1
7	chr16	25258530	25258530	C	T	exonic	ZKSCAN2		synonymous SNV	ZKSCAN2:NM_001012981:exon5:c.G987A:	NOVEL		366	297	1.04861	1	303	249	1.0095	1
7	chr16	30941778	30941778	G	A	exonic	FBXL19		nonsynonymous SNV	FBXL19:NM_001099784:exon7:c.G1234A:	DBSNP		145	132	1.11549	1	126	120	1.09167	1
7	chr16	74339197	74339197	G	C	exonic	PSMD7		nonsynonymous SNV	PSMD7:NM_002811:exon7:c.G541C:p.D18	NOVEL		16	130	1.19391	1	7	103	1.15915	1
7	chr16	79633495	79633495	T	A	exonic	MAF		nonsynonymous SNV	MAF:NM_001031804:exon1:c.A305T:p.N1	NOVEL		13	74	1.14049	1	12	60	1.03161	1
7	chr16	79633520	79633520	T	C	exonic	MAF		nonsynonymous SNV	MAF:NM_001031804:exon1:c.A280G:p.M9	NOVEL		9	71	1.19	1	10	61	1.06357	1
7	chr17	8133943	8133943	G	A	exonic	CTC1		nonsynonymous SNV	CTC1:NM_025099:exon16:c.C2696T:p.S899	COSMIC	ID=COSM448336	16	113	1.20335	1	10	98	1.1233	1
7	chr17	37667867	37667867	G	A	exonic	CDK12		nonsynonymous SNV	CDK12:NM_015083:exon8:c.G2752A:p.D91	COSMIC	ID=COSM484315	274	293	1.30489	1	239	226	1.08768	1
7	chr17	38451644	38451644	C	A	exonic	CDC6		nonsynonymous SNV	CDC6:NM_001254:exon8:c.C1120A:p.Q374	DBSNP		203	157	1.10125	1	222	152	0.909532	1
7	chr17	40476982	40476982	T	C	exonic	STAT3		nonsynonymous SNV	STAT3:NM_001369512:exon16:c.A1463G:	NOVEL		355	119	0.838736	1	334	99	0.740311	1
7	chr17	40939916	40939916	C	T	exonic	WNK4		nonsynonymous SNV	WNK4:NM_001321299:exon7:c.C854T:p.S	DBSNP+COSMIC	ID=COSM406666	226	591	1.20835	2	165	531	1.23516	2
7	chr17	41131581	41131581	G	A	exonic	PTGES3L;PTGES3L		synonymous SNV	PTGES3L-AARSD1:NM_001136042:exon2:	NOVEL		34	174	1.26148	1	11	137	1.29877	1
7	chr17	80680756	80680756	C	T	exonic	FN3KRP		synonymous SNV	FN3KRP:NM_024619:exon4:c.C462T:p.L15	DBSNP		137	12	0.188525	1	111	14	0.250648	1
7	chr18	77917697	77917697	G	A	exonic	PARD6G		nonsynonymous SNV	PARD6G:NM_032510:exon3:c.C1088T:p.P3	DBSNP		101	85	1.06974	1	89	84	1.08662	1
7	chr19	47127028	47127028	G	A	exonic	PTGIR		nonsynonymous SNV	PTGIR:NM_000960:exon2:c.C455T:p.A152	DBSNP+COSMIC	ID=COSM246861	179	10	0.123854	1	174	7	0.0865497	1
7	chr19	58485780	58485780	A	G	exonic	C19orf18		synonymous SNV	C19orf18:NM_152474:exon1:c.T21C:p.G7	NOVEL		167	117	0.964363	1	157	94	0.838108	1
7	chr2	187534492	187534492	T	C	exonic	ITGAV		nonsynonymous SNV	ITGAV:NM_001145000:exon24:c.T2549C:p	NOVEL		155	129	1.06327	1	132	105	0.991487	1
7	chr20	20486081	20486081	C	G	exonic	RALGAPA2		nonsynonymous SNV	RALGAPA2:NM_020343:exon34:c.G5026C:	NOVEL		266	206	1.02164	1	219	181	0.916554	1
7	chr20	45014834	45014834	C	G	exonic	ELMO2		nonsynonymous SNV	ELMO2:NM_182764:exon8:c.G606C:p.L202	NOVEL		293	229	1.02692	1	227	227	1.11896	1
7	chr22	25315907	25315907	A	T	exonic	SGSM1		nonsynonymous SNV	SGSM1:NM_001098498:exon23:c.A2957T:	NOVEL		117	93	1.03666	1	98	83	1.02623	1

7	chr3	55513491	55513491	T	C	exonic	WNT5A		nonsynonymous SNV	WNT5A:NM_001256105:exon3:c.A197G:p	NOVEL		86	73	1.07473	1	73	64	1.04546	1				
7	chr3	74548926	74548926	G	A	exonic	CNTN3		synonymous SNV	CNTN3:NM_020872:exon2:c.C66T:p.L22L	DBSNP		208	168	1.04591	1	195	126	0.878439	1				
7	chr3	184910834	184910834	G	C	exonic	EHHADH		nonsynonymous SNV	EHHADH:NM_001166415:exon7:c.C1064G	NOVEL		528	18	0.0771708	1	456	30	0.138144	1				
7	chr4	6107403	6107403	G	A	exonic	JAKMIP1		nonsynonymous SNV	JAKMIP1:NM_001099433:exon3:c.C421T:p	NOVEL		59	47	1.03792	1	74	59	0.992765	1				
7	chr4	84519325	84519325	C	G	exonic	GPAT3		nonsynonymous SNV	GPAT3:NM_032717:exon10:c.C1118G:p.T3	NOVEL		125	101	1.04613	1	123	73	0.833514	1				
7	chr6	335134	335134	C	T	exonic	DUSP22		synonymous SNV	DUSP22:NM_001286555:exon4:c.C159T:p.	NOVEL		182	54	0.535618	1	193	41	0.392115	1				
7	chr6	15496974	15496974	G	C	exonic	JARID2		nonsynonymous SNV	JARID2:NM_001267040:exon7:c.G1002C:p	NOVEL		204	190	1.12884	1	180	181	1.12206	1				
7	chr6	27778143	27778143	G	A	exonic	HIST1H3H		nonsynonymous SNV	HIST1H3H:NM_003536:exon1:c.G292A:p.E	COSMIC	ID=COSM386107	163	140	1.08158	1	172	135	0.984105	1				
7	chr6	36179152	36179152	G	A	exonic	BRPF3		nonsynonymous SNV	BRPF3:NM_015695:exon7:c.G2297A:p.R76	DBSNP		178	84	0.750501	1	172	90	0.768754	1				
7	chr6	128643348	128643348	G	A	exonic	PTPRK		synonymous SNV	PTPRK:NM_001135648:exon3:c.C331T:p.L	NOVEL		275	251	1.11702	1	291	242	1.01609	1				
7	chr7	1542664	1542664	G	T	exonic	INTS1		synonymous SNV	INTS1:NM_001080453:exon3:c.C222A:p.L7	NOVEL		161	135	1.06762	1	114	102	1.0568	1				
7	chr7	6590800	6590800	C	T	exonic	GRID2IP		nonsynonymous SNV	GRID2IP:NM_001145118:exon1:c.G268A:p	NOVEL		18	15	1.06402	1	14	18	1.25883	1				
7	chr7	100486851	100486851	C	T	exonic	UFSP1		stopgain	UFSP1:NM_001015072:exon1:c.G42A:p.W	NOVEL		44	45	1.18357	1	29	40	1.29735	1				
7	chr7	102517969	102517969	A	G	exonic	FBXL13		nonsynonymous SNV	FBXL13:NM_001111038:exon16:c.T1580C:	NOVEL		44	51	1.25667	1	27	24	1.05314	1				
7	chr7	136700530	136700530	C	A	exonic	CHRM2		nonsynonymous SNV	CHRM2:NM_001006629:exon2:c.C918A:p.	NOVEL		237	13	0.121724	1	201	26	0.256326	1				
7	chr8	24771489	24771489	C	T	exonic	NEFM		synonymous SNV	NEFM:NM_005382:exon1:c.C183T:p.L61L	NOVEL		23	189	1.19538	1	17	192	1.13723	1				
7	chr8	113318319	113318319	C	A	exonic	CSMD3		nonsynonymous SNV	CSMD3:NM_001363185:exon47:c.G7388T:	NOVEL		168	131	1.02559	1	138	119	1.03624	1				
7	chr9	33294658	33294658	C	T	exonic	NFX1		nonsynonymous SNV	NFX1:NM_001318758:exon2:c.C266T:p.S8	NOVEL		280	221	1.03259	1	228	211	1.07563	1				
7	chr9	33364069	33364069	G	C	exonic	NFX1		nonsynonymous SNV	NFX1:NM_001318758:exon20:c.G2938C:p	NOVEL		124	115	1.12635	1	116	120	1.13793	1				
7	chrX	65483514	65483514	C	A	exonic	HEPH		synonymous SNV	HEPH:NM_001367243:exon15:c.C2328A:p.	NOVEL		341	287	1.06978	1	325	284	1.04363	1				
7	chrX	122757772	122757772	G	A	exonic	THOC2		synonymous SNV	THOC2:NM_001081550:exon28:c.C3369T:	NOVEL		192	185	1.14869	1	194	161	1.01495	1				
7	chrX	153906546	153906546	C	G	exonic	GAB3		nonsynonymous SNV	GAB3:NM_001282283:exon9:c.G1556C:p.	NOVEL		212	173	1.05186	1	179	179	1.11896	1				
7	chr1	55509571	55509571	T	A	exonic	PCSK9		nonsynonymous SNV	PCSK9:NM_174936:exon2:c.T263A:p.L88H	NOVEL		169	9	0.118357	1	158	27	0.326616	1				
7	chr21	10914364	10914364	G	T	exonic	TPTE		stopgain	TPTE:NM_001290224:exon16:c.C941A:p.S	NOVEL		349	84	0.454114	1	244	73	0.515359	1				
7	chr7	98592427	98592427	C	T	exonic	TRRAP		nonsynonymous SNV	TRRAP:NM_003496:exon65:c.C10136T:p.A	NOVEL		199	9	0.101287	1	192	10	0.110788	1				
8	chrX	54784520	54784520	G	C	exonic	ITIH6		nonsynonymous SNV	ITIH6:NM_198510:exon8:c.C1987G:p.P663	NOVEL		153	16	NA	1	161	13	NA	1				
8	chrX	106069394	106069394	G	T	exonic	TBC1D8B		nonsynonymous SNV	TBC1D8B:NM_017752:exon6:c.G962T:p.C3	NOVEL		157	0	NA	1	182	19	NA	1				
8	chrX	107380354	107380354	G	T	exonic	ATG4A		nonsynonymous SNV	ATG4A:NM_001321288:exon7:c.G270T:p.L	NOVEL		120	0	NA	1	118	10	NA	1				
8	chrX	129149038	129149038	G	A	exonic	BCORL1		nonsynonymous SNV	BCORL1:NM_001184772:exon3:c.G2290A:	NOVEL		397	0	NA	1	306	23	NA	1				
8	chrX	130410975	130410975	C	T	exonic	IGSF1		nonsynonymous SNV	IGSF1:NM_001170962:exon13:c.G2519A:p	NOVEL		247	0	NA	1	153	15	NA	1				
8	chr7	151877998	151877998	C	T	exonic	KMT2C		nonsynonymous SNV	KMT2C:NM_170606:exon36:c.G6947A:p.S	NOVEL		322	5	0.102278	1	236	67	1.47415	1				
8	chr15	54305250	54305250	C	A	exonic	UNC13C		synonymous SNV	UNC13C:NM_001080534:exon1:c.C150A:p	DBSNP		511	2	0.0260778	1	360	98	1.42649	1				
8	chr5	118264398	118264398	C	T	exonic	DTWD2		nonsynonymous SNV	DTWD2:NM_001308081:exon4:c.G233A:p.	DBSNP		72	61	1.02262	3	75	56	1.42494	2				
8	chr4	123664890	123664890	G	A	exonic	BBS12		nonsynonymous SNV	BBS12:NM_152618:exon2:c.G1843A:p.A61	NOVEL		426	3	0.0467759	1	355	95	1.40741	1				
8	chr1	40433489	40433489	T	A	exonic	MFSD2A		nonsynonymous SNV	MFSD2A:NM_001287808:exon9:c.T641A:p	NOVEL		228	7	0.199245	1	168	44	1.38365	1				
8	chr17	54588152	54588152	C	A	exonic	ANKFN1		nonsynonymous SNV	ANKFN1:NM_001365758:exon21:c.C2759A	NOVEL		798	5	0.0416498	1	591	153	1.37097	1				
8	chr17	17696562	17696562	C	A	exonic	RAI1		stopgain	RAI1:NM_030665:exon3:c.C300A:p.Y100X	NOVEL		154	1	0.0431546	1	121	31	1.35965	1				
8	chr11	100858277	100858277	G	T	exonic	ARHGAP42		nonsynonymous SNV	ARHGAP42:NM_152432:exon23:c.G2490T	NOVEL		282	81	1.26937	1	239	161	1.34167	2				
8	chr11	1466858	1466858	G	A	exonic	BRSK2		synonymous SNV	BRSK2:NM_001256627:exon11:c.G1035A:	DBSNP		283	65	1.24937	1	182	122	1.33772	2				
8	chr8	10480257	10480257	C	T	exonic	RP1L1		nonsynonymous SNV	RP1L1:NM_178857:exon2:c.G455A:p.R152	DBSNP+COSMIC	ID=COSM472249	206	55	1.40955	1	144	94	1.31653	2				
8	chr16	29913171	29913171	C	T	exonic	ASPHD1		synonymous SNV	ASPHD1:NM_181718:exon1:c.C879T:p.S29	DBSNP+COSMIC	ID=COSM435084	268	73	1.43195	1	222	141	1.29477	2				
8	chr22	42306498	42306498	G	A	exonic	SHISA8		nonsynonymous SNV	SHISA8:NM_001207020:exon3:c.C725T:p.F	NOVEL		63	0	0	1	50	12	1.29032	1				
8	chr1	115256529	115256529	T	C	exonic	NRAS		nonsynonymous SNV	NRAS:NM_002524:exon3:c.A182G:p.Q61R	DBSNP+COSMIC	ID=COSM584;OC	248	47	1.0657	1	170	107	1.28761	2				
8	chr4	158281174	158281174	T	G	exonic	GRIA2		nonsynonymous SNV	GRIA2:NM_000826:exon13:c.T2170G:p.L7	NOVEL		301	0	0	1	255	61	1.28692	1				
8	chr17	64641580	64641580	G	A	exonic	PRKCA		synonymous SNV	PRKCA:NM_002737:exon5:c.G480A:p.G16	NOVEL		258	3	0.0768845	1	193	45	1.2605	1				
8	chr19	50905065	50905065	C	A	exonic	POLD1		nonsynonymous SNV	POLD1:NM_001308632:exon3:c.C347A:p.P	NOVEL		174	49	1.46977	1	144	87	1.25541	2				
8	chr2	121106849	121106849	A	G	exonic	INHBB		nonsynonymous SNV	INHBB:NM_002193:exon2:c.A623G:p.H208	NOVEL		275	99	0.885303	2	212	128	1.2549	2				
8	chr1	27106970	27106970	A	T	exonic	ARID1A		nonsynonymous SNV	ARID1A:NM_006015:exon20:c.A6581T:p.N	NOVEL		303	3	0.065578	1	272	63	1.25373	1				
8	chr5	169533111	169533111	G	C	exonic	FOXI1		nonsynonymous SNV	FOXI1:NM_012188:exon1:c.G150C:p.E50D	NOVEL		196	213	1.16116	3	145	183	1.23984	3				
8	chr12	49224385	49224385	T	A	exonic	DDX23		nonsynonymous SNV	DDX23:NM_004818:exon17:c.A2330T:p.Y7	NOVEL		149	2	0.0885954	1	114	26	1.2381	1				
8	chr12	55614925	55614925	G	T	exonic	OR10A7		nonsynonymous SNV	OR10A7:NM_001005280:exon1:c.G117T:p	DBSNP		514	105	1.13464	1	378	223	1.23683	2				
8	chr11	13984627	13984627	C	T	exonic	SPON1		unknown	UNKNOWN	NOVEL		249	58	1.26371	1	174	102	1.23188	2				
8	chr7	128358946	128358946	T	C	exonic	FAM71F1		synonymous SNV	FAM71F1:NM_001282788:exon3:c.T496C:	NOVEL		240	2	0.0552806	1	218	49	1.22347	1				
8	chr13	73346338	73346338	C	T	exonic	DIS3		nonsynonymous SNV	DIS3:NM_001322348:exon9:c.G1093A:p.D	COSMIC	ID=COSM158635	318	70	1.20677	1	222	128	1.21905	2				
8	chr18	67992405	67992405	C	G	exonic	SOCS6		synonymous SNV	SOCS6:NM_004232:exon2:c.C501G:p.A167	NOVEL		245	67	1.43641	1	193	109	1.20309	2				
8	chr8	37729516	37729516	G	T	exonic	RAB11FIP1		nonsynonymous SNV	RAB11FIP1:NM_001002814:exon4:c.C2804	NOVEL		367	79	1.18482	1	267	150	1.19904	2				
8	chr10	70945805	70945805	G	A	exonic	SUPV3L1		nonsynonymous SNV	SUPV3L1:NM_003171:exon2:c.G334A:p.D1	NOVEL		180	0	0	1	146	32	1.1985	1				
8	chr17	6945553	6945553	C	T	exonic	SLC16A11		synonymous SNV	SLC16A11:NM_153357:exon3:c.G876A:p.G	NOVEL		64	75	1.20305	3	61	71	1.19529	3				
8	chr16	8992445	8992445	T	G	exonic	USP7		nonsynonymous SNV	USP7:NM_001286457:exon24:c.A2535C:p.	NOVEL		474	5	0.0698221	1	386	84	1.19149	1				
8	chr5	9063091	9063091	C	T	exonic	SEMA5A		nonsynonymous SNV	SEMA5A:NM_003966:exon18:c.G2426A:p.	NOVEL		276	76	1.44421	1	218	120	1.18343	2				
8	chr4	90743435	90743435	C	A	exonic	SNCA		nonsynonymous SNV	SNCA:NM_000345:exon4:c.G268T:p.A90S,	NOVEL		160	41	1.36441	1	109	58	1.15768	2				
8	chr10	102738882	102738882	C	T	exonic	SEMA4G		synonymous SNV	SEMA4G:NM_001203244:exon8:c.C837T:p	NOVEL		345	89	1.3717	1	250	133	1.15753	2				
8	chr8	133053862	133053862	C	T	exonic	OC90		nonsynonymous SNV	OC90:NM_001080399:exon5:c.G254A:p.C8	NOVEL		242	50	1.14537	1	164	87	1.15538	2				
8	chr12	121442194	121442194	T	C	exonic	C12orf43		nonsynonymous SNV	C12orf43:NM_001286197:exon5:c.A455G:	NOVEL		268	1	0.024866	1	216	45	1.14943	1				
8	chr17	75208153	75208153	C	A	exonic	SEC14L1		nonsynonymous SNV	SEC14L1:NM_001144001:exon13:c.C1631A	NOVEL		111	104	1.07853	3	94	100	1.14548	3				
8	chr11	46430097	46430097	C	T	exonic	AMBRA1		synonymous SNV	AMBRA1:NM_001300731:exon16:c.G3189	DBSNP		78	17	1.19697	1	65	34	1.14478	2				
8	chr3	124641126	124641126	G	T	exonic	MUC13		stopgain	MUC13:NM_033049:exon4:c.C659A:p.S22	NOVEL		296	72	1.30871	1	249	130	1.14336	2				
8	chr7	107423770	107423770	C	G	exonic	SLC26A3		synonymous SNV	SLC26A3:NM_000111:exon9:c.G999C:p.V3	NOVEL		401	1	0.0166392	1	305	63	1.1413	1				
8	chr1	151739288	151739288	C	T	exonic	OAZ3		unknown	UNKNOWN	DBSNP		244	35	0.839116	1	189	98	1.13821	2				
8	chr10	15697398	15697398	G	C	exonic	ITGA8		nonsynonymous SNV	ITGA8:NM_001291494:exon10:c.C911G:p.	NOVEL		129	29	1.22772	1	118	61	1.13594	2				
8	chr3	44882668	44882668	C	T	exonic	KIF15		nonsynonymous SNV	KIF15:NM_020242:exon29:c.C3523T:p.H11	NOVEL		89	14	0.909178	1	68	35	1.13269	2				
8	chr20	61937348	61937348	G	A	exonic	COL20A1		synonymous SNV	COL20A1:NM_020882:exon5:c.G453A:p.E1	NOVEL		253	58	1.24746	1	179	90	1.11524	2				
8	chr1	76365378	76365378	C	T	exonic	MSH4		nonsynonymous SNV	MSH4:NM_002440:exon19:c.C2606T:p.T86	DBSNP+COSMIC	ID=COSM424151	100	19	1.06798	1	72	36	1.11111	2				
8	chr17	19645524	19645524	T	A	exonic	ALDH3A1		nonsynonymous SNV	ALDH3A1:NM_001135168:exon4:c.A482T:	NOVEL		153	135	1.04515	3	131	131	1.11111	3				
8	chr22	42377782	42377782	C	T	exonic	SEPTIN3		synonymous SNV	SEPTIN3:NM_019106:exon2:c.C144T:p.I48	DBSNP+COSMIC	ID=COSM355500	174	3	0.113372	1	125	25	1.11111	1				
8	chr3	71096195	71096195	T	G	exonic	FOXP1		nonsynonymous SNV	FOXP1:NM_001244813:exon4:c.A262C:p.N	NOVEL		96	102	1.14861	3	93	91	1.09903	3				
8	chr7	39990885	39990885	G	C	exonic	CDK13		nonsynonymous SNV	CDK13:NM_003718:exon1:c.G645C:p.E215	NOVEL		63	8	0.753685	1	39	19	1.09195	2				
8	chr6	83075084	83075084	G	A	exonic	TPBG		nonsynonymous SNV	TPBG:NM_001166392:exon2:c.G406A:p.G	NOVEL		234	210	1.05457	3	227	219	1.09118	3				
8	chr16	3447384	3447384	G	C	exonic	ZSCAN32		synonymous SNV	ZSCAN32:NM_001284527:exon2:c.C174G:	NOVEL		211	149	1.38424	2	201	192	1.08567	3				
8	chr6	54735272	54735272	A	G	exonic	FAM83B		synonymous SNV	FAM83B:NM_001010872:exon2:c.A228G:p	NOVEL		313	301	1.09304	3	304	284	1.07332	3				
8	chr11	69456218	69456218	A	C	exonic	CCND1		nonsynonymous SNV	CCND1:NM_053056:exon1:c.A137C:p.K46T	COSMIC	ID=COSM373340	131	93	1.38856	2	132	122	1.06737	3				
8	chr7	14613932	14613932	C	A	exonic	DGKB		nonsynonymous SNV	DGKB:NM_001350720:exon18:c.G1621T:p	NOVEL		410	87	1.1709	1	308	144	1.06195	2				
8	chr9	13107093	13107093	G	A	exonic	MPDZ		synonymous SNV	MPDZ:NM_001261407:exon45:c.C5898T:p	DBSNP+COSMIC	ID=COSM390440	207	86	0.981656	2	159	144	1.05611	3				
8	chr1	35227190	35227190	A	T	exonic	GJB4		nonsynonymous SNV	GJB4:NM_153212:exon2:c.A335T:p.N112I	COSMIC	ID=COSM348881	368	352	1.09005	3	338	305	1.05409	3				
8	chr20	60922003	60922003	C	A	exonic	LAMA5		nonsynonymous SNV	LAMA5:NM_005560:exon7:c.G1038T:p.K34	NOVEL		227	3	0.0872473	1	167	31	1.04377	1				
8	chr12	21531220	21531220	C	T	exonic	IAPP		nonsynonymous SNV	IAPP:NM_000415:exon3:c.C130T:p.R44C,IA	DBSNP+COSMIC	ID=COSM392848	277	285	1.1307	3	301	266	1.04252	3				
8	chr2	142012135	142012135	C	G	exonic	LRP1B		nonsynonymous SNV	LRP1B:NM_018557:exon4:c.G419C:p.C140	NOVEL		200	1	0.0332784	1	162	30	1.04167	1				
8	chr9	117810690	117810690	C	T	exonic	TNC		synonymous SNV	TNC:NM_002160:exon16:c.G4701A:p.G15	COSMIC	ID=COSM541024	184	28	0.883447	1	127	57	1.03261	2				
8	chr15	42115947	42115947	C	T	exonic	MAPKBP1		nonsynonymous SNV	MAPKBP1:NM_014994:exon29:c.C3901T:p	DBSNP		352	388	1.16906	3	378	328	1.03242	3				
8	chr2	128331518	128331518	C	T	exonic	MYO7B		nonsynonymous SNV	MYO7B:NM_001080527:exon7:c.C616T:p.	DBSNP+COSMIC	ID=COSM372637	222	187	1.01943	3	219	190	1.03233	3				
8	chr12	11905413	11905413	G	A	exonic	ETV6		synonymous SNV	ETV6:NM_001987:exon2:c.G63A:p.E21E	NOVEL		111	58	1.14781	2	134	24	1.01266	1				
8	chr7	21781578	21781578	T	A	exonic	DNAH11		nonsynonymous SNV	DNAH11:NM_001277115:exon49:c.T7948A	NOVEL		430	1	0.0155196	1	378	67	1.00375	1				
8	chr11	102826169	102826169	C	G	exonic	MMP13		nonsynonymous SNV	MMP13:NM_002427:exon2:c.G174C:p.E58	DBSNP		251	107	0.850124	2	334	58	0.986395	1				
8	chr1	36786203	36786203	C	T	exonic	SH3D21		nonsynonymous SNV	SH3D21:NM_024676:exon11:c.C1606T:p.P	DBSNP		255	1	0.0261287	1	241	41	0.969267	1				
8	chr1	192993042	192993042	C	T	exonic	UCHL5		nonsynonymous SNV	UCHL5:NM_001199261:exon8:c.G664A:p.A	NOVEL		424	2	0.0314035	1	351	59	0.95935	1				
8	chr4	174169580	174169580	A	G	exonic	GALNT7		synonymous SNV	GALNT7:NM_017423:exon2:c.A576G:p.L19	COSMIC	ID=COSM609989	146	1	0.0455031	1	150	23	0.88632	1				
8	chr11	102826045	102826045	C	G	exonic	MMP13		nonsynonymous SNV	MMP13:NM_002427:exon2:c.G298C:p.D10	NOVEL		261	112	0.854065	2	360	52	0.841424	1				
8	chr6	16290733	16290733	G	C	exonic	GMPR		synonymous SNV	GMPR:NM_006877:exon8:c.G738C:p.S246	NOVEL		186	66	0.875935	2	243	35	0.839329	1				
8	chr1	170136772	170136772	G	T	exonic	METTL11B		nonsynonymous SNV	METTL11B:NM_001136107:exon4:c.G726T	NOVEL		204	64	0.798681	2	218	31	0.829987	1				
8	chr9	17466775	17466775	A	T	exonic	CNTLN		synonymous SNV	CNTLN:NM_001365029:exon23:c.A3738T:	NOVEL		276	100	0.889489	2	341	45	0.777202	1				
8	chr12	132380214	132380214	G	A	exonic	ULK1		synonymous SNV	ULK1:NM_003565:exon2:c.G180A:p.L60L	DBSNP		195	2	0.049695	1	173	52	0.77037	2				
8	chr13	28624235	28624235	T	C	exonic	FLT3		nonsynonymous SNV	FLT3:NM_004119:exon6:c.A739G:p.I247V	NOVEL		281	101	0.884273	2	290	37	0.754332	1				
8	chr9	85640744	85640744	C	T	exonic	RASEF		nonsynonymous SNV	RASEF:NM_152573:exon2:c.G524A:p.R175	DBSNP		428	150	0.867944	2	516	65	0.745841	1				
8	chr2	182763750	182763750	C	G	exonic	ITPRID2		nonsynonymous SNV	ITPRID2:NM_001130445:exon6:c.C414G:p.	NOVEL		186	83	1.03194	2	254	31	0.725146	1				
8	chr11	55944472	55944472	G	T	exonic	OR5J2		nonsynonymous SNV	OR5J2:NM_001005492:exon1:c.G379T:p.V	NOVEL		213	90	0.993409	2	262	31	0.705347	1				
8	chr10	70432654	70432654	G	A	exonic	TET1		nonsynonymous SNV	TET1:NM_030625:exon8:c.G4676A:p.R155	DBSNP+COSMIC	ID=COSM697834	135	50	0.903913	2	137	15	0.657895	1				
8	chr13	46549476	46549476	G	A	exonic	ZC3H13		stopgain	ZC3H13:NM_001076788:exon12:c.C2410T:	COSMIC	ID=COSM682566	611	208	0.849391	2	697	72	0.624187	1				
8	chr19	47201073	47201073	G	C	exonic	PRKD2		nonsynonymous SNV	PRKD2:NM_001079882:exon7:c.C685G:p.Q	NOVEL		211	88	0.984328	2	222	21	0.576132	1				
8	chr19	47201081	47201081	C	A	exonic	PRKD2		nonsynonymous SNV	PRKD2:NM_001079882:exon7:c.G677T:p.R	NOVEL		211	87	0.976408	2	223	21	0.57377	1				
8	chr19	47201078	47201078	A	C	exonic	PRKD2		nonsynonymous SNV	PRKD2:NM_001079882:exon7:c.T680G:p.V	NOVEL		211	87	0.976408	2	220	20	0.555556	1				
8	chr5	149514490	149514490	T	G	exonic	PDGFRB		nonsynonymous SNV	PDGFRB:NM_001355016:exon3:c.A262C:p	NOVEL		103	0	0	1	98	8	0.503145	1				
8	chr14	33205024	33205024	A	G	exonic	AKAP6		nonsynonymous SNV	AKAP6:NM_004274:exon11:c.A3308G:p.N	NOVEL		254	3	0.0780812	1	237	19	0.494792	1				
8	chr12	88390235	88390235	C	G	exonic	C12orf50		nonsynonymous SNV	C12orf50:NM_001363616:exon6:c.G397C:	NOVEL		145	0	0	1	152	12	0.487805	1				
8	chr19	9582030	9582030	C	T	exonic	ZNF560		nonsynonymous SNV	ZNF560:NM_152476:exon6:c.G263A:p.S88	NOVEL		78	4	0.326291	1	65	5	0.47619	1				
8	chr10	18787309	18787309	G	A	exonic	CACNB2		nonsynonymous SNV	CACNB2:NM_000724:exon3:c.G194A:p.R6	DBSNP		259	1	0.0257268	1	237	18	0.470588	1				
8	chr19	9076857	9076857	A	G	exonic	MUC16		nonsynonymous SNV	MUC16:NM_024690:exon3:c.T10589C:p.V	NOVEL		306	0	0	1	292	22	0.467091	1				
8	chr18	59854921	59854921	G	A	exonic	RELCH		synonymous SNV	RELCH:NM_001346229:exon1:c.G183A:p.V	NOVEL		258	0	0	1	227	17	0.464481	1				
8	chr19	549075	549075	C	G	exonic	GZMM		nonsynonymous SNV	GZMM:NM_001258351:exon4:c.C385G:p.L	NOVEL		171	0	0	1	175	13	0.460993	1				
8	chr11	110007637	110007637	G	A	exonic	ZC3H12C		nonsynonymous SNV	ZC3H12C:NM_033390:exon2:c.G271A:p.E9	DBSNP		409	0	0	1	357	26	0.452567	1				
8	chr2	1926904	1926904	C	T	exonic	MYT1L		nonsynonymous SNV	MYT1L:NM_001303052:exon10:c.G637A:p	NOVEL		520	0	0	1	470	33	0.437376	1				
8	chr15	52563964	52563964	T	C	exonic	MYO5C		synonymous SNV	MYO5C:NM_018728:exon7:c.A828G:p.K27	NOVEL		588	0	0	1	490	34	0.43257	1				
8	chr14	24901440	24901440	C	T	exonic	KHNYN		nonsynonymous SNV	KHNYN:NM_001290256:exon3:c.C1096T:p	NOVEL		229	0	0	1	233	16	0.42838	1				
8	chr5	137501645	137501645	T	G	exonic	BRD8		nonsynonymous SNV	BRD8:NM_001300962:exon10:c.A1027C:p	NOVEL		319	0	0	1	271	18	0.415225	1				
8	chr16	8622144	8622144	G	C	exonic	TMEM114		unknown	UNKNOWN	NOVEL		167	0	0	1	136	9	0.413793	1				
8	chr7	37956111	37956111	C	A	exonic	SFRP4		nonsynonymous SNV	SFRP4:NM_003014:exon1:c.G29T:p.C10F	DBSNP		378	2	0.035205	1	363	24	0.413437	1				
8	chr3	169835114	169835114	G	C	exonic	PHC3		nonsynonymous SNV	PHC3:NM_001308116:exon10:c.C2057G:p.	NOVEL		474	0	0	1	444	29	0.408739	1				
8	chr14	38261600	38261600	G	A	exonic	TTC6		nonsynonymous SNV	TTC6:NM_001310135:exon19:c.G3737A:p.	DBSNP		415	0	0	1	367	23	0.393162	1				
8	chr6	116441669	116441669	G	A	exonic	COL10A1		nonsynonymous SNV	COL10A1:NM_000493:exon3:c.C1610T:p.T	NOVEL		241	0	0	1	237	14	0.371846	1				
8	chr2	189899670	189899670	C	T	exonic	COL5A2		nonsynonymous SNV	COL5A2:NM_000393:exon53:c.G4325A:p.R	DBSNP		587	20	0.220394	1	576	34	0.371585	1				
8	chr13	108955692	108955692	T	A	exonic	TNFSF13B		synonymous SNV	TNFSF13B:NM_001145645:exon3:c.T516A	NOVEL		250	0	0	1	280	16	0.36036	1				
8	chr17	66352894	66352894	G	C	exonic	ARSG		nonsynonymous SNV	ARSG:NM_001352909:exon5:c.G605C:p.S	NOVEL		285	0	0	1	266	15	0.355872	1				
8	chr11	106810698	106810698	C	A	exonic	GUCY1A2		stopgain	GUCY1A2:NM_000855:exon4:c.G694T:p.E	NOVEL		374	25	0.356434	1	436	23	0.33406	1				
8	chr5	40981658	40981658	G	A	exonic	C7		nonsynonymous SNV	C7:NM_000587:exon18:c.G2515A:p.A839T	NOVEL		229	11	0.306577	1	225	11	0.310734	1				
8	chr1	111854826	111854826	C	A	exonic	CHIA		nonsynonymous SNV	CHIA:NM_201653:exon4:c.C70A:p.L24M	NOVEL		141	5	0.229074	1	146	7	0.305011	1				
8	chr4	126239257	126239257	C	T	exonic	FAT4		nonsynonymous SNV	FAT4:NM_001291285:exon1:c.C1691T:p.S	NOVEL		453	1	0.0147334	1	481	21	0.278884	1				
8	chr7	21654815	21654815	G	A	exonic	DNAH11		synonymous SNV	DNAH11:NM_001277115:exon21:c.G3936	NOVEL		271	18	0.416613	1	258	9	0.224719	1				
8	chr14	56763726	56763726	T	A	exonic	PELI2		nonsynonymous SNV	PELI2:NM_021255:exon6:c.T1105A:p.C369	NOVEL		343	12	0.226106	1	304	2	0.043573	1				
8	chr15	25925334	25925334	G	T	exonic	ATP10A		nonsynonymous SNV	ATP10A:NM_024490:exon20:c.C3800A:p.A	COSMIC	ID=COSM433652	125	7	0.354717	1	153	1	0.04329	1				
8	chr15	25925335	25925335	C	T	exonic	ATP10A		nonsynonymous SNV	ATP10A:NM_024490:exon20:c.G3799A:p.A	NOVEL		127	7	0.349423	1	153	1	0.04329	1				
8	chr3	50513587	50513587	C	T	exonic	CACNA2D2		nonsynonymous SNV	CACNA2D2:NM_001005505:exon2:c.G250	DBSNP		148	7	0.302082	1	160	1	0.0414079	1				
8	chr19	12858331	12858331	C	T	exonic	GET3		synonymous SNV	GET3:NM_004317:exon6:c.C840T:p.L280L	DBSNP		263	12	0.291882	1	232	1	0.0286123	1				
8	chr5	139866541	139866541	A	G	splicing	ANKHD1;ANKHD1-	NM_017747:exon14:c			NOVEL		159	0	0	1	139	9	0.405405	1				
9	chr16	88873797	88873797	C	T	exonic	CDT1		nonsynonymous SNV	CDT1:NM_030928:exon9:c.C1384T:p.R462	DBSNP		47	45	1.04827	1	77	70	1.24286	1				
9	chr19	6535064	6535064	C	T	exonic	TNFSF9		nonsynonymous SNV	TNFSF9:NM_003811:exon3:c.C752T:p.P25	DBSNP+COSMIC	ID=COSM272779	69	70	1.07927	1	90	80	1.22824	1				
9	chr7	157959802	157959802	G	A	exonic	PTPRN2		nonsynonymous SNV	PTPRN2:NM_001308267:exon5:c.C617T:p.	DBSNP+COSMIC	ID=COSM330618	101	101	1.07156	1	69	59	1.20305	1				
9	chr14	102808257	102808257	G	A	exonic	ZNF839		nonsynonymous SNV	ZNF839:NM_001267827:exon8:c.G2177A:	COSMIC	ID=COSM549559	212	179	0.981124	1	205	175	1.20198	1				
9	chr1	240071103	240071103	G	A	exonic	CHRM3		nonsynonymous SNV	CHRM3:NM_000740:exon5:c.G352A:p.G11	COSMIC	ID=COSM348591	423	184	0.952776	1	353	163	1.14037	1				
9	chr15	28360664	28360664	G	A	exonic	HERC2		stopgain	HERC2:NM_004667:exon89:c.C13633T:p.R	NOVEL		123	99	0.955718	1	176	121	1.06334	1				
9	chr9	134348956	134348956	T	G	exonic	PRRC2B		nonsynonymous SNV	PRRC2B:NM_013318:exon14:c.T2169G:p.	NOVEL		172	116	0.863203	1	270	184	1.0578	1				
9	chr5	43649374	43649374	C	T	exonic	NNT		nonsynonymous SNV	NNT:NM_001331026:exon10:c.C1177T:p.H	NOVEL		109	106	1.05661	1	137	89	1.02784	1				
9	chr7	99998830	99998830	G	A	exonic	ZCWPW1		stopgain	ZCWPW1:NM_001258008:exon16:c.C1543	DBSNP+COSMIC	ID=COSM372422	86	66	0.930568	1	62	40	1.02354	1				
9	chr1	84376975	84376975	A	G	exonic	TTLL7		synonymous SNV	TTLL7:NM_001350215:exon14:c.T1578C:p	DBSNP		91	72	0.946657	1	160	99	0.99765	1				
9	chr12	94613837	94613837	T	A	exonic	PLXNC1		nonsynonymous SNV	PLXNC1:NM_005761:exon6:c.T1600A:p.C5	NOVEL		75	90	1.16898	1	70	43	0.993192	1				
9	chr8	113657378	113657378	A	G	exonic	CSMD3		synonymous SNV	CSMD3:NM_001363185:exon18:c.T2880C:	NOVEL		158	164	1.09153	1	141	146	0.988015	1				
9	chr1	40363069	40363069	C	A	exonic	MYCL		nonsynonymous SNV	MYCL:NM_001033081:exon2:c.G1070T:p.R	COSMIC	ID=COSM166745	103	76	0.90993	1	68	40	0.966672	1				
9	chr11	3691046	3691046	G	A	exonic	CHRNA10		synonymous SNV	CHRNA10:NM_020402:exon2:c.C187T:p.L6	NOVEL		305	298	1.05912	1	402	230	0.949847	1				
9	chr9	27055726	27055726	A	G	exonic	IFT74		nonsynonymous SNV	IFT74:NM_001099222:exon17:c.A1453G:p	NOVEL		61	49	0.954665	1	77	43	0.935255	1				
9	chr1	47133926	47133926	G	A	exonic	ATPAF1		synonymous SNV	ATPAF1:NM_001042546:exon1:c.C138T:p.	NOVEL		18	0	0	1	18	10	0.932148	1				
9	chr2	162036243	162036243	G	A	exonic	TANK		nonsynonymous SNV	TANK:NM_001199135:exon2:c.G70A:p.D24	NOVEL		230	192	0.975071	1	329	180	0.922992	1				
9	chr4	654333	654333	C	G	exonic	PDE6B		nonsynonymous SNV	PDE6B:NM_001350155:exon8:c.C390G:p.D	NOVEL		64	48	0.918482	1	135	71	0.899568	1				
9	chr19	49362813	49362813	C	T	exonic	PLEKHA4		nonsynonymous SNV	PLEKHA4:NM_001161354:exon7:c.G605A:	NOVEL		96	88	1.02497	1	112	57	0.880301	1				
9	chr5	58021943	58021943	G	C	exonic	RAB3C		nonsynonymous SNV	RAB3C:NM_001317915:exon3:c.G361C:p.D	NOVEL		195	151	0.935295	1	292	147	0.873969	1				
9	chr12	23793781	23793781	C	T	exonic	SOX5		nonsynonymous SNV	SOX5:NM_001261415:exon8:c.G952A:p.A3	COSMIC	ID=COSM377236	114	118	1.09004	1	182	88	0.850672	1				
9	chr12	55714593	55714593	G	A	exonic	OR6C1		synonymous SNV	OR6C1:NM_001005182:exon1:c.G210A:p.S	DBSNP+COSMIC	ID=COSM339886	129	101	0.941112	1	160	76	0.840513	1				
9	chr17	40025765	40025765	C	G	exonic	ACLY		nonsynonymous SNV	ACLY:NM_001303275:exon25:c.G3145C:p.	NOVEL		37	28	0.923193	1	76	36	0.838933	1				
9	chr12	25398285	25398285	C	T	exonic	KRAS		nonsynonymous SNV	KRAS:NM_001369786:exon2:c.G34A:p.G1	DBSNP+COSMIC	ID=COSM115250	90	60	0.85725	1	195	87	0.805217	1				
9	chr12	21970194	21970194	C	A	exonic	ABCC9		nonsynonymous SNV	ABCC9:NM_005691:exon31:c.G3819T:p.E1	NOVEL		250	231	1.02923	1	295	124	0.772415	1				
9	chr13	52686409	52686409	C	T	exonic	NEK5		nonsynonymous SNV	NEK5:NM_001365552:exon5:c.G307A:p.D1	NOVEL		126	4	0.0351731	1	52	46	0.755721	1				
9	chr3	183904393	183904393	G	A	exonic	ABCF3		nonsynonymous SNV	ABCF3:NM_001351298:exon2:c.G172A:p.D	NOVEL		279	7	0.0524541	1	124	41	0.648549	1				
9	chr20	42315576	42315576	C	A	exonic	MYBL2		nonsynonymous SNV	MYBL2:NM_001278610:exon4:c.C292A:p.R	NOVEL		128	0	0	1	185	58	0.622967	1				
9	chr14	95030225	95030225	G	C	exonic	SERPINA4		nonsynonymous SNV	SERPINA4:NM_001289032:exon2:c.G517C	NOVEL		272	8	0.0612322	1	192	60	0.621432	1				
9	chr7	84685042	84685042	T	G	exonic	SEMA3D		nonsynonymous SNV	SEMA3D:NM_152754:exon7:c.A852C:p.R2	NOVEL		74	2	0.056398	1	52	16	0.614121	1				
9	chr10	70248329	70248329	G	A	exonic	SLC25A16		nonsynonymous SNV	SLC25A16:NM_001324317:exon6:c.C266T:	DBSNP		447	5	0.0237071	1	423	126	0.59902	1				
9	chr19	8618027	8618027	C	T	exonic	MYO1F		nonsynonymous SNV	MYO1F:NM_001348355:exon6:c.G500A:p.	DBSNP+COSMIC	ID=COSM438223	87	3	0.0714375	1	24	7	0.589358	1				
9	chr19	4446558	4446558	C	T	exonic	UBXN6		nonsynonymous SNV	UBXN6:NM_001171091:exon8:c.G700A:p.	DBSNP+COSMIC	ID=COSM328955	342	5	0.0308808	1	273	79	0.58577	1				
9	chr5	140235931	140235931	G	T	exonic	PCDHA10		stopgain	PCDHA10:NM_018901:exon1:c.G298T:p.E1	NOVEL		234	5	0.0448353	1	129	36	0.569458	1				
9	chr1	159804991	159804991	G	C	exonic	SLAMF8		nonsynonymous SNV	SLAMF8:NM_001330741:exon4:c.G472C:p	NOVEL		147	1	0.0212373	1	186	34	0.557911	1				
9	chr3	128664310	128664310	G	A	exonic	KIAA1257		nonsynonymous SNV	KIAA1257:NM_001348520:exon9:c.C989T:	NOVEL		316	2	0.0134788	1	205	55	0.552119	1				
9	chr4	166996124	166996124	G	T	exonic	TLL1		synonymous SNV	TLL1:NM_012464:exon17:c.G2283T:p.V76	NOVEL		407	6	0.031135	1	389	99	0.529491	1				
9	chr11	92535061	92535061	G	T	exonic	FAT3		nonsynonymous SNV	FAT3:NM_001008781:exon9:c.G8882T:p.S	NOVEL		419	3	0.0152355	1	404	86	0.458084	1				
9	chr19	16902193	16902193	G	A	exonic	NWD1		synonymous SNV	NWD1:NM_001347994:exon13:c.G2355A:	DBSNP+COSMIC	ID=COSM536560	85	0	0	1	106	21	0.431577	1				
9	chr5	121412637	121412637	T	A	exonic	LOX		startloss	LOX:NM_001178102:exon1:c.A1T:p.M1?,L	NOVEL		323	2	0.0131885	1	370	61	0.369399	1				
9	chr21	10914367	10914367	C	T	exonic	TPTE		nonsynonymous SNV	TPTE:NM_001290224:exon16:c.G938A:p.C	DBSNP		370	54	0.272945	1	547	74	0.311016	1				
9	chr3	176768315	176768315	C	T	exonic	TBL1XR1		nonsynonymous SNV	TBL1XR1:NM_001321195:exon5:c.G250A:	COSMIC	ID=COSM285606	309	0	0	1	321	26	0.195563	1				
9	chr2	186673070	186673070	A	T	exonic	FSIP2		nonsynonymous SNV	FSIP2:NM_173651:exon17:c.A19037T:p.K6	NOVEL		109	51	0.683121	1	104	8	0.18643	1				
9	chr1	196963323	196963323	G	T	exonic	CFHR5		nonsynonymous SNV	CFHR5:NM_030787:exon4:c.G544T:p.V182	NOVEL		204	0	0	1	175	9	0.176577	1				
9	chr17	4461797	4461797	G	A	exonic	GGT6		nonsynonymous SNV	GGT6:NM_153338:exon3:c.C899T:p.A300V	DBSNP		133	0	0	1	94	6	0.156601	1				
9	chr11	51411490	51411490	C	G	exonic	OR4A5		nonsynonymous SNV	OR4A5:NM_001005272:exon1:c.G906C:p.	NOVEL		233	0	0	1	204	13	0.15636	1				
9	chr19	48832732	48832732	T	G	exonic	EMP3		nonsynonymous SNV	EMP3:NM_001313905:exon4:c.T305G:p.L	NOVEL		256	31	0.231487	1	468	25	0.132354	1				
9	chr3	173322647	173322647	G	A	exonic	NLGN1		nonsynonymous SNV	NLGN1:NM_001365923:exon2:c.G259A:p.	NOVEL		475	0	0	1	327	17	0.128983	1				
9	chr5	115428336	115428336	G	C	exonic	COMMD10		nonsynonymous SNV	COMMD10:NM_001308080:exon4:c.G296	NOVEL		426	4	0.0199361	1	407	20	0.122249	1				
9	chr7	142562030	142562030	A	C	exonic	EPHB6		nonsynonymous SNV	EPHB6:NM_004445:exon7:c.A472C:p.I158	NOVEL		287	0	0	1	388	19	0.121843	1				
9	chr8	3216751	3216751	C	T	exonic	CSMD1		nonsynonymous SNV	CSMD1:NM_033225:exon21:c.G3227A:p.R	DBSNP+COSMIC	ID=COSM124919	149	0	0	1	247	11	0.11128	1				
9	chr22	34000453	34000453	G	A	exonic	LARGE1		nonsynonymous SNV	LARGE1:NM_001362951:exon5:c.C583T:p.	DBSNP+COSMIC	ID=COSM293935	281	1	0.00759974	1	329	14	0.106531	1				
9	chr1	109793944	109793944	G	C	exonic	CELSR2		nonsynonymous SNV	CELSR2:NM_001408:exon1:c.G1243C:p.G4	NOVEL		390	3	0.0163597	1	436	18	0.103481	1				
9	chr19	1008225	1008225	G	A	exonic	GRIN3B		nonsynonymous SNV	GRIN3B:NM_138690:exon6:c.G2401A:p.D	DBSNP		199	2	0.0213246	1	433	17	0.0986006	1				
9	chr19	51446952	51446952	C	G	exonic	KLK5		nonsynonymous SNV	KLK5:NM_001077492:exon6:c.G817C:p.G2	NOVEL		174	2	0.0243537	1	204	8	0.0984911	1				
9	chr17	7802428	7802428	T	A	exonic	CHD3		nonsynonymous SNV	CHD3:NM_001005271:exon14:c.T2428A:p.	NOVEL		223	12	0.109436	1	285	10	0.0884751	1				
9	chr3	52400552	52400552	C	T	exonic	DNAH1		synonymous SNV	DNAH1:NM_015512:exon35:c.C5598T:p.S	NOVEL		182	20	0.212191	1	305	10	0.0828576	1				
9	chr22	24106839	24106839	G	T	exonic	C22orf15		nonsynonymous SNV	C22orf15:NM_001331041:exon4:c.G269T:	NOVEL		483	0	0	1	467	15	0.0812245	1				
9	chr10	128193349	128193349	G	A	exonic	C10orf90		synonymous SNV	C10orf90:NM_001004298:exon3:c.C420T:p	NOVEL		359	0	0	1	317	10	0.079817	1				
9	chr10	128974415	128974415	G	A	exonic	INSYN2A		nonsynonymous SNV	INSYN2A:NM_001039762:exon4:c.C245T:p	NOVEL		240	25	0.202182	1	167	5	0.0758725	1				
9	chrX	30261183	30261183	G	A	exonic	MAGEB4		nonsynonymous SNV	MAGEB4:NM_002367:exon1:c.G931A:p.E3	NOVEL		131	94	0.447675	1	247	15	0.0747142	1				
9	chrX	71425117	71425117	G	A	exonic	ERCC6L		nonsynonymous SNV	ERCC6L:NM_017669:exon2:c.C3500T:p.A1	NOVEL		193	0	0	1	137	8	0.0720004	1				
9	chr14	89160722	89160722	T	A	exonic	EML5		nonsynonymous SNV	EML5:NM_183387:exon17:c.A2468T:p.K82	NOVEL		148	13	0.173047	1	143	4	0.0710208	1				
9	chr16	69377383	69377383	A	T	exonic	TMED6		nonsynonymous SNV	TMED6:NM_144676:exon4:c.T650A:p.I217	NOVEL		250	55	0.386465	1	324	9	0.0705409	1				
9	chr2	166756316	166756316	C	T	exonic	TTC21B		synonymous SNV	TTC21B:NM_024753:exon21:c.G2832A:p.L	NOVEL		259	14	0.109904	1	271	7	0.0657198	1				
9	chr3	14862895	14862895	G	A	exonic	FGD5		nonsynonymous SNV	FGD5:NM_001320276:exon1:c.G2317A:p.D	NOVEL		656	0	0	1	777	20	0.065496	1				
9	chr2	79350320	79350320	T	A	exonic	REG1A		nonsynonymous SNV	REG1A:NM_002909:exon6:c.T480A:p.F160	NOVEL		243	23	0.185308	1	368	9	0.062308	1				
9	chr3	107097221	107097221	C	T	exonic	CCDC54		nonsynonymous SNV	CCDC54:NM_032600:exon1:c.C787T:p.L263	NOVEL		272	79	0.482356	1	178	4	0.057363	1				
9	chr10	19641090	19641090	C	T	exonic	MALRD1		synonymous SNV	MALRD1:NM_001142308:exon26:c.C4305T	NOVEL		378	34	0.17686	1	413	9	0.0556638	1				
9	chr14	77715129	77715129	A	G	exonic	TMEM63C		nonsynonymous SNV	TMEM63C:NM_020431:exon20:c.A1784G:	NOVEL		121	15	0.236374	1	185	4	0.0552384	1				
9	chr6	116442867	116442867	G	A	exonic	COL10A1		nonsynonymous SNV	COL10A1:NM_000493:exon3:c.C412T:p.R1	DBSNP		461	29	0.126838	1	288	5	0.0445395	1				
9	chr4	113540662	113540662	A	G	exonic	ZGRF1		nonsynonymous SNV	ZGRF1:NM_001350397:exon6:c.T536C:p.I	DBSNP		468	27	0.116898	1	474	7	0.0379836	1				
9	chr7	100377353	100377353	G	A	exonic	ZAN		unknown	UNKNOWN	NOVEL		60	12	0.357188	1	72	1	0.0357536	1				
9	chr19	56952620	56952620	G	C	exonic	ZNF667		nonsynonymous SNV	ZNF667:NM_022103:exon5:c.C1744G:p.P5	NOVEL		354	32	0.177668	1	464	6	0.0333193	1				
9	chr5	76028500	76028500	G	C	exonic	F2R		synonymous SNV	F2R:NM_001992:exon2:c.G450C:p.L150L,F	NOVEL		367	25	0.136679	1	338	2	0.015353	1				
9	chr10	124768552	124768552	G	T	exonic	ACADSB		nonsynonymous SNV	ACADSB:NM_001609:exon1:c.G7T:p.G3C	NOVEL		62	7	0.217419	1	44	0	0	1				
9	chr1	120307008	120307008	G	A	exonic	HMGCS2		nonsynonymous SNV	HMGCS2:NM_001166107:exon2:c.C346T:p	DBSNP		187	7	0.0773293	1	194	0	0	1				
9	chr17	40025841	40025841	C	T	splicing	ACLY	NM_001303274:exon2			NOVEL		44	37	0.978959	1	102	62	0.986713	1				
10	chr1	149884987	149884987	G	A	exonic	SV2A		nonsynonymous SNV	SV2A:NM_001328674:exon2:c.C406T:p.R1	DBSNP		174	1086	1.33197	6	153	1155	1.35663	6	184	1170	1.30883	6
10	chr10	134218962	134218962	C	T	exonic	PWWP2B		nonsynonymous SNV	PWWP2B:NM_001098637:exon2:c.C958T:	COSMIC	ID=COSM401279	314	272	1.05472	1	239	258	1.15142	1	247	248	1.04609	1
10	chr3	142840388	142840388	C	G	exonic	CHST2		nonsynonymous SNV	CHST2:NM_004267:exon2:c.C730G:p.L244	NOVEL		133	108	1.01829	1	114	118	1.12814	1	123	90	0.882236	1
10	chr14	62242825	62242825	G	A	exonic	SNAPC1		nonsynonymous SNV	SNAPC1:NM_003082:exon5:c.G547A:p.V1	NOVEL		52	34	0.898351	1	18	22	1.11759	1	24	28	1.12428	1
10	chr21	41452198	41452198	C	T	exonic	DSCAM		nonsynonymous SNV	DSCAM:NM_001271534:exon25:c.G4301A	NOVEL		42	42	1.13615	1	34	34	1.10902	1	41	31	0.898982	1
10	chr10	108339132	108339132	A	C	exonic	SORCS1		nonsynonymous SNV	SORCS1:NM_001013031:exon25:c.T3366G	NOVEL		110	98	1.0706	1	107	107	1.10902	1	81	89	1.09311	1
10	chr5	98115676	98115676	A	G	exonic	RGMB		nonsynonymous SNV	RGMB:NM_001366508:exon2:c.A529G:p.N	NOVEL		157	108	0.926069	1	149	145	1.09393	1	124	110	0.981519	1
10	chr22	50960455	50960455	T	C	exonic	NCAPH2		synonymous SNV	NCAPH2:NM_001185011:exon13:c.T1149C	NOVEL		16	70	1.03559	1	8	68	1.08982	1	5	52	0.992523	1
10	chr4	619788	619788	C	T	exonic	PDE6B		nonsynonymous SNV	PDE6B:NM_000283:exon1:c.C373T:p.P125	NOVEL		87	79	1.0814	1	90	86	1.08381	1	78	69	0.980062	1
10	chr7	5338899	5338899	G	A	exonic	SLC29A4		synonymous SNV	SLC29A4:NM_001040661:exon9:c.G1050A	DBSNP		35	33	1.10273	1	46	43	1.07164	1	40	34	0.959332	1
10	chr6	166721173	166721173	T	G	exonic	PRR18		nonsynonymous SNV	PRR18:NM_175922:exon1:c.A458C:p.E153	NOVEL		106	103	1.11984	1	112	101	1.05175	1	104	121	1.12286	1
10	chr19	57175198	57175198	T	A	exonic	ZNF835		nonsynonymous SNV	ZNF835:NM_001005850:exon2:c.A1369T:p	NOVEL		196	151	0.98881	1	158	142	1.04987	1	159	163	1.05695	1
10	chr8	35606141	35606141	G	A	exonic	UNC5D		synonymous SNV	UNC5D:NM_001322818:exon12:c.G1848A	DBSNP+COSMIC	ID=COSM145678	179	147	1.02463	1	132	118	1.04691	1	110	124	1.10644	1
10	chr17	27209635	27209635	C	T	exonic	FLOT2		nonsynonymous SNV	FLOT2:NM_001330170:exon5:c.G421A:p.D	NOVEL		229	150	0.899327	1	193	170	1.03875	1	172	143	0.947867	1
10	chr5	31409426	31409426	C	T	exonic	DROSHA		synonymous SNV	DROSHA:NM_001100412:exon31:c.G3570	DBSNP		103	66	0.887407	1	74	65	1.03721	1	80	79	1.03741	1
10	chr4	89361323	89361323	C	T	exonic	HERC6		synonymous SNV	HERC6:NM_001165136:exon21:c.C2655T:p	DBSNP		234	198	1.04147	1	175	153	1.03463	1	186	142	0.903933	1
10	chr4	647752	647752	G	A	exonic	PDE6B		nonsynonymous SNV	PDE6B:NM_000283:exon4:c.G823A:p.V275	DBSNP+COSMIC	ID=COSM442455	172	141	1.02362	1	184	159	1.02819	1	164	154	1.01115	1
10	chr22	38229066	38229066	G	A	exonic	ANKRD54		stopgain	ANKRD54:NM_001349853:exon6:c.C424T:	DBSNP		29	117	1.01958	1	23	119	1.02075	1	7	116	1.02604	1
10	chr13	73349483	73349483	T	C	exonic	DIS3		nonsynonymous SNV	DIS3:NM_001322348:exon5:c.A484G:p.R1	NOVEL		50	150	0.954224	1	28	135	1.0088	1	9	125	1.01489	1
10	chr6	127836780	127836780	C	G	exonic	SOGA3		nonsynonymous SNV	SOGA3:NM_001012279:exon2:c.G980C:p.	NOVEL		87	68	0.99688	1	96	80	1.0082	1	81	97	1.13782	1
10	chr4	4864741	4864741	A	G	exonic	MSX1		synonymous SNV	MSX1:NM_002448:exon2:c.A783G:p.A261	NOVEL		139	99	0.9452	1	120	100	1.0082	1	119	85	0.869982	1
10	chr12	5155092	5155092	C	T	exonic	KCNA5		synonymous SNV	KCNA5:NM_002234:exon1:c.C1779T:p.N59	DBSNP+COSMIC	ID=COSM124333	238	176	0.966002	1	197	161	0.997497	1	181	186	1.0582	1
10	chr2	71797808	71797808	C	A	exonic	DYSF		nonsynonymous SNV	DYSF:NM_001130976:exon28:c.C3069A:p.	DBSNP		191	127	0.907491	1	147	120	0.99687	1	119	104	0.973756	1
10	chr16	8622195	8622195	C	T	exonic	TMEM114		unknown	UNKNOWN	DBSNP		99	87	1.06285	1	110	89	0.991986	1	78	93	1.13556	1
10	chr8	42563916	42563916	T	C	exonic	CHRNB3		nonsynonymous SNV	CHRNB3:NM_000749:exon2:c.T109C:p.F37	NOVEL		413	315	0.983206	1	329	264	0.987456	1	302	246	0.937295	1
10	chr2	33783326	33783326	C	G	exonic	RASGRP3		nonsynonymous SNV	RASGRP3:NM_015376:exon15:c.C1625G:p	NOVEL		90	67	0.969707	1	65	51	0.975171	1	71	58	0.938772	1
10	chr5	159851330	159851330	T	C	exonic	PTTG1		synonymous SNV	PTTG1:NM_001282382:exon3:c.T363C:p.N	NOVEL		149	123	1.02755	1	109	85	0.97182	1	80	64	0.927981	1
10	chr9	101804370	101804370	C	T	exonic	COL15A1		nonsynonymous SNV	COL15A1:NM_001855:exon24:c.C2555T:p.	NOVEL		134	75	0.815418	1	93	72	0.967871	1	104	74	0.868027	1
10	chrX	107437765	107437765	T	A	exonic	COL4A6		nonsynonymous SNV	COL4A6:NM_001287758:exon16:c.A1001T	NOVEL		21	91	0.923122	1	13	89	0.967673	1	5	93	0.990715	1
10	chr5	127728871	127728871	G	A	exonic	FBN2		synonymous SNV	FBN2:NM_001999:exon10:c.C1422T:p.A47	DBSNP+COSMIC	ID=COSM143289	217	156	0.950345	1	167	127	0.958131	1	142	132	1.00588	1
10	chr2	8870907	8870907	A	T	exonic	KIDINS220		nonsynonymous SNV	KIDINS220:NM_001348734:exon28:c.T509	NOVEL		252	192	0.982616	1	202	151	0.948792	1	185	152	0.941749	1
10	chr13	86370034	86370034	G	A	exonic	SLITRK6		nonsynonymous SNV	SLITRK6:NM_032229:exon2:c.C610T:p.L20	NOVEL		72	225	0.963863	1	55	192	0.946814	1	14	190	1.01329	1
10	chr12	88496713	88496713	C	G	exonic	CEP290		nonsynonymous SNV	CEP290:NM_025114:exon26:c.G2893C:p.A	NOVEL		89	74	1.0316	1	70	52	0.945393	1	53	34	0.815983	1
10	chr2	48063045	48063045	T	C	exonic	FBXO11		nonsynonymous SNV	FBXO11:NM_001190274:exon5:c.A683G:p	NOVEL		72	78	1.1816	1	77	55	0.924182	1	53	46	0.970162	1
10	chr2	32475639	32475639	C	G	exonic	NLRC4		nonsynonymous SNV	NLRC4:NM_001199138:exon4:c.G1294C:p	NOVEL		245	180	0.962386	1	227	161	0.920371	1	206	157	0.903056	1
10	chr4	156773411	156773411	T	C	exonic	ASIC5		nonsynonymous SNV	ASIC5:NM_017419:exon4:c.A643G:p.T215	NOVEL		297	230	0.991706	1	246	174	0.918901	1	221	195	0.97873	1
10	chr17	5356936	5356936	C	T	exonic	DHX33		nonsynonymous SNV	DHX33:NM_001199699:exon7:c.G841A:p.V	DBSNP		268	180	0.912977	1	193	135	0.912911	1	191	154	0.932016	1
10	chr4	126237973	126237973	C	A	exonic	FAT4		nonsynonymous SNV	FAT4:NM_001291285:exon1:c.C407A:p.P1	NOVEL		292	218	0.971297	1	292	203	0.909619	1	245	220	0.987851	1
10	chr11	106647213	106647213	C	T	exonic	GUCY1A2		nonsynonymous SNV	GUCY1A2:NM_000855:exon6:c.G1788A:p.	COSMIC	ID=COSM613110	82	88	1.17625	1	87	60	0.905321	1	67	54	0.931816	1
10	chr15	69007587	69007587	A	G	exonic	CORO2B		nonsynonymous SNV	CORO2B:NM_001190456:exon8:c.A889G:p	NOVEL		264	180	0.921202	1	245	166	0.895849	1	202	158	0.916381	1
10	chr12	109372354	109372354	C	G	exonic	SVOP		nonsynonymous SNV	SVOP:NM_018711:exon2:c.G123C:p.W41C	NOVEL		82	34	0.666019	1	57	36	0.858595	1	57	47	0.943596	1
10	chr7	88963488	88963488	A	G	exonic	ZNF804B		nonsynonymous SNV	ZNF804B:NM_181646:exon4:c.A1192G:p.T	NOVEL		263	165	0.876003	1	217	135	0.850667	1	165	136	0.943396	1
10	chr16	2578283	2578283	G	A	exonic	AMDHD2		nonsynonymous SNV	AMDHD2:NM_001145815:exon7:c.G764A:	DBSNP		232	29	0.252478	1	195	59	0.515213	1	183	52	0.462016	1
10	chr1	1233280	1233280	C	A	exonic	ACAP3		synonymous SNV	ACAP3:NM_030649:exon14:c.G1050T:p.L3	NOVEL		165	30	0.349584	1	143	40	0.484817	1	115	39	0.528769	1
10	chr13	53420220	53420220	C	T	exonic	PCDH8		synonymous SNV	PCDH8:NM_002590:exon1:c.G2352A:p.G7	NOVEL		159	60	0.348575	1	125	73	0.449074	1	83	75	0.516436	1
10	chr4	266394	266394	G	T	exonic	ZNF732		nonsynonymous SNV	ZNF732:NM_001137608:exon4:c.C252A:p.	NOVEL		214	34	0.311525	1	135	34	0.446232	1	115	44	0.5778	1
10	chr11	85396855	85396855	G	A	exonic	CCDC89		stopgain	CCDC89:NM_152723:exon1:c.C319T:p.Q10	NOVEL		299	54	0.347604	1	258	62	0.429745	1	251	86	0.532832	1
10	chr1	154987221	154987221	C	T	exonic	ZBTB7B		stopgain	ZBTB7B:NM_001256455:exon2:c.C85T:p.Q	NOVEL		761	26	0.207217	1	723	53	0.424685	1	698	84	0.653949	1
10	chr14	71435799	71435799	T	C	exonic	PCNX1		synonymous SNV	PCNX1:NM_001308160:exon5:c.T576C:p.S	NOVEL		274	45	0.320544	1	164	43	0.422102	1	139	41	0.47559	1
10	chr5	50091161	50091161	G	C	exonic	PARP8		nonsynonymous SNV	PARP8:NM_001178056:exon12:c.G1338C:	NOVEL		617	84	0.272287	1	560	129	0.415278	1	483	140	0.469204	1
10	chr1	155324302	155324302	C	A	exonic	ASH1L		nonsynonymous SNV	ASH1L:NM_001366177:exon16:c.G7190T:p	NOVEL		966	49	0.302801	1	788	56	0.412571	1	754	66	0.490006	1

10	chr4	91229874	91229874	G	A	exonic	CCSER1		nonsynonymous SNV	CCSER1:NM_001145065:exon2:c.G439A:p.	DBSNP		318	43	0.270662	1	260	58	0.404548	1	181	72	0.594201	1				
10	chr7	1786162	1786162	G	A	exonic	ELFN1		nonsynonymous SNV	ELFN1:NM_001128636:exon3:c.G1930A:p.	NOVEL		72	9	0.252478	1	46	10	0.396078	1	61	23	0.571703	1				
10	chr17	61607500	61607500	A	G	exonic	KCNH6		nonsynonymous SNV	KCNH6:NM_001278919:exon3:c.A356G:p.	COSMIC	ID=COSM536391	75	13	0.335681	1	68	14	0.378689	1	52	27	0.713606	1				
10	chr1	110086040	110086040	G	A	exonic	GPR61		synonymous SNV	GPR61:NM_031936:exon2:c.G396A:p.S132	DBSNP+COSMIC	ID=COSM339952	325	47	0.287092	1	300	61	0.374793	1	256	86	0.525042	1				
10	chr5	167956788	167956788	G	T	exonic	FBLL1		nonsynonymous SNV	FBLL1:NM_001355274:exon1:c.G279T:p.K	NOVEL		53	7	0.265102	1	75	15	0.369673	1	49	11	0.382792	1				
10	chr8	144943496	144943496	A	T	exonic	EPPK1		nonsynonymous SNV	EPPK1:NM_031308:exon2:c.T3926A:p.V13	NOVEL		250	39	0.306642	1	263	46	0.357799	1	210	77	0.560184	1				
10	chr11	119146795	119146795	C	T	exonic	CBL		nonsynonymous SNV	CBL:NM_005188:exon6:c.C958T:p.H320Y	NOVEL		178	22	0.249953	1	131	25	0.355455	1	107	41	0.578421	1				
10	chr2	120194573	120194573	T	C	exonic	TMEM37		nonsynonymous SNV	TMEM37:NM_183240:exon2:c.T130C:p.S4	NOVEL		191	28	0.290522	1	164	29	0.33328	1	168	43	0.425508	1				
10	chr15	35084392	35084392	G	A	exonic	ACTC1		nonsynonymous SNV	ACTC1:NM_005159:exon5:c.C707T:p.S236	NOVEL		305	0	0	1	231	28	0.239788	1	239	2	0.0173274	1				
10	chr11	75152703	75152703	C	T	exonic	GDPD5		nonsynonymous SNV	GDPD5:NM_001351167:exon9:c.G899A:p.	DBSNP		62	1	0.0360682	1	50	6	0.237647	1	71	0	0	1				
10	chr19	19017895	19017895	C	T	exonic	COPE		nonsynonymous SNV	COPE:NM_001330469:exon4:c.G317A:p.R1	DBSNP		182	0	0	1	130	15	0.229452	1	151	0	0	1				
10	chr12	21242843	21242843	A	G	exonic	SLCO1B3-SLCO1B		nonsynonymous SNV	SLCO1B7:NM_001009562:exon13:c.A1726	NOVEL		98	1	0.0229525	1	71	8	0.224611	1	63	1	0.0326243	1				
10	chr17	80708373	80708373	T	A	exonic	FN3K		synonymous SNV	FN3K:NM_022158:exon6:c.T672A:p.A224A	NOVEL		657	0	0	1	512	57	0.222193	1	541	0	0	1				
10	chr9	133884838	133884838	C	T	exonic	LAMC3		synonymous SNV	LAMC3:NM_006059:exon1:c.C237T:p.A79A	NOVEL		243	1	0.0093127	1	194	21	0.216645	1	228	0	0	1				
10	chr4	72994591	72994591	G	A	exonic	NPFFR2		nonsynonymous SNV	NPFFR2:NM_004885:exon2:c.G589A:p.G1	COSMIC	ID=COSM610101	212	0	0	1	156	16	0.206329	1	164	0	0	1				
10	chr11	67206363	67206363	C	T	exonic	CORO1B		nonsynonymous SNV	CORO1B:NM_020441:exon10:c.G1123A:p.	DBSNP		143	0	0	1	125	12	0.194281	1	118	0	0	1				
10	chr7	48450118	48450118	T	A	exonic	ABCA13		synonymous SNV	ABCA13:NM_152701:exon40:c.T12072A:p.	NOVEL		457	2	0.00990109	1	321	30	0.189576	1	385	0	0	1				
10	chr5	32091128	32091128	C	G	exonic	PDZD2		nonsynonymous SNV	PDZD2:NM_178140:exon20:c.C7574G:p.P2	NOVEL		237	1	0.00954748	1	209	19	0.184836	1	207	7	0.0682977	1				
10	chr10	18250764	18250764	G	A	exonic	SLC39A12		synonymous SNV	SLC39A12:NM_001282734:exon2:c.G114A	NOVEL		528	1	0.00429546	1	352	29	0.168827	1	357	2	0.0116321	1				
10	chrX	150348464	150348464	G	A	exonic	GPR50		nonsynonymous SNV	GPR50:NM_004224:exon2:c.G409A:p.E13	DBSNP+COSMIC	ID=COSM355906	242	0	0	1	185	31	0.159165	1	184	0	0	1				
10	chr5	122495212	122495212	A	T	exonic	PRDM6		nonsynonymous SNV	PRDM6:NM_001136239:exon5:c.A1033T:p	NOVEL		364	0	0	1	282	20	0.14689	1	257	1	0.00809286	1				
10	chr6	43139686	43139686	C	T	exonic	SRF		synonymous SNV	SRF:NM_003131:exon1:c.C292T:p.L98L	NOVEL		296	3	0.022799	1	216	15	0.144028	1	231	5	0.0442364	1				
10	chr9	72895806	72895806	G	A	exonic	SMC5		synonymous SNV	SMC5:NM_015110:exon6:c.G810A:p.R270	NOVEL		234	0	0	1	140	9	0.133975	1	181	0	0	1				
10	chr15	33855202	33855202	A	G	exonic	RYR3		synonymous SNV	RYR3:NM_001036:exon11:c.A1137G:p.L37	DBSNP		285	0	0	1	221	14	0.132138	1	223	0	0	1				
10	chr14	38061212	38061212	G	A	exonic	FOXA1		synonymous SNV	FOXA1:NM_004496:exon2:c.C777T:p.Y259	DBSNP		116	16	0.27543	1	130	8	0.117796	1	116	7	0.118827	1				
10	chr8	87076809	87076809	C	T	exonic	PSKH2		synonymous SNV	PSKH2:NM_033126:exon2:c.G237A:p.R79R	NOVEL		676	86	0.256454	1	660	35	0.1117	1	544	26	0.0952402	1				
10	chr16	69687177	69687177	G	T	exonic	NFAT5		nonsynonymous SNV	NFAT5:NM_001367709:exon4:c.G179T:p.S	NOVEL		343	34	0.204929	1	316	15	0.100515	1	296	10	0.0682339	1				
10	chr17	29657376	29657376	A	C	exonic	NF1		nonsynonymous SNV	NF1:NM_000267:exon38:c.A5609C:p.Q187	NOVEL		604	9	0.0333617	1	426	16	0.0802909	1	413	27	0.128125	1				
10	chr11	6559710	6559710	C	A	exonic	DNHD1		synonymous SNV	DNHD1:NM_144666:exon15:c.C3096A:p.I1	NOVEL		368	25	0.144548	1	350	13	0.0794338	1	318	14	0.0880464	1				
10	chr6	132966375	132966375	C	G	exonic	TAAR1		synonymous SNV	TAAR1:NM_138327:exon1:c.G768C:p.V256	NOVEL		680	0	0	1	523	19	0.0777541	1	532	0	0	1				
10	chr11	102564794	102564794	C	T	exonic	MMP27		nonsynonymous SNV	MMP27:NM_022122:exon8:c.G1036A:p.E3	COSMIC	ID=COSM170448	180	7	0.0493558	1	121	6	0.057545	1	94	19	0.182931	1				
10	chr19	11728077	11728077	C	T	exonic	ZNF627		synonymous SNV	ZNF627:NM_001290084:exon4:c.A429T:p.	NOVEL		195	11	0.121336	1	159	4	0.0544303	1	156	1	0.0132991	1				
10	chr13	58299008	58299008	A	G	exonic	PCDH17		synonymous SNV	PCDH17:NM_001040429:exon4:c.A3060G	NOVEL		414	0	0	1	353	14	0.0464646	1	332	0	0	1				
10	chr5	79732669	79732669	G	A	exonic	ZFYVE16		synonymous SNV	ZFYVE16:NM_001284237:exon3:c.G165A:	NOVEL		955	22	0.0511674	1	731	10	0.029933	1	698	14	0.0410553	1				
10	chr14	92470291	92470291	T	C	exonic	TRIP11		synonymous SNV	TRIP11:NM_001321851:exon11:c.A4026G	NOVEL		539	18	0.0734316	1	432	5	0.025378	1	384	5	0.0268375	1				
10	chr3	108813801	108813801	C	T	exonic	MORC1		nonsynonymous SNV	MORC1:NM_014429:exon7:c.G538A:p.E18	DBSNP		160	13	0.170751	1	114	1	0.0192873	1	129	3	0.0474536	1				
10	chr20	44163119	44163119	T	C	exonic	WFDC6		nonsynonymous SNV	WFDC6:NM_080827:exon3:c.A248G:p.E83	NOVEL		313	12	0.0839003	1	231	2	0.0190389	1	226	4	0.0363123	1				
10	chr4	70513055	70513055	G	T	exonic	UGT2A1		nonsynonymous SNV	UGT2A1:NM_001301239:exon1:c.C308A:p	NOVEL		672	0	0	1	523	1	0.00423289	1	523	18	0.0694699	1				
10	chr9	133769089	133769089	C	T	exonic	QRFP		nonsynonymous SNV	QRFP:NM_198180:exon3:c.G137A:p.G46E	NOVEL		322	0	0	1	284	0	0	1	312	16	0.101852	1				
10	chrX	43626733	43626733	C	A	exonic	MAOB		nonsynonymous SNV	MAOB:NM_000898:exon15:c.G1543T:p.G5	NOVEL		40	0	0	1	20	0	0	1	29	5	0.153526	1				
10	chr9	77280402	77280402	A	G	splicing	RORB	NM_006914:exon7:c.8			NOVEL		428	276	0.890845	1	284	226	0.982895	1	227	254	1.10258	1				
10	chr2	173850223	173850223	T	A	splicing	RAPGEF4	NM_007023:exon12:c			NOVEL		294	2	0.0153534	1	196	14	0.147869	1	214	0	0	1				
13	chrX	131803191	131803191	G	A	exonic	HS6ST2		synonymous SNV	HS6ST2:NM_001077188:exon5:c.C1017T:p	NOVEL		150	0	NA	1	115	34	NA	1								
13	chrX	148059894	148059894	A	G	exonic	AFF2		nonsynonymous SNV	AFF2:NM_001170628:exon15:c.A2402G:p	NOVEL		341	0	NA	1	319	28	NA	1								
13	chr9	94123982	94123982	T	G	exonic	AUH		nonsynonymous SNV	AUH:NM_001306190:exon1:c.A190C:p.K64	NOVEL		200	126	1.42778	1	192	134	1.35193	3								
13	chr14	47120464	47120464	A	T	exonic	RPL10L		nonsynonymous SNV	RPL10L:NM_080746:exon1:c.T476A:p.F159	NOVEL		64	85	1.30653	1	113	52	1.08209	2								
13	chr20	21306918	21306918	C	T	exonic	XRN2		nonsynonymous SNV	XRN2:NM_001317960:exon2:c.C311T:p.P1	NOVEL		33	41	1.26893	1	69	31	1.0644	2								
13	chr12	76424616	76424616	C	G	exonic	PHLDA1		synonymous SNV	PHLDA1:NM_007350:exon1:c.G906C:p.L30	NOVEL		59	73	1.26658	1	104	65	1.32059	2								
13	chr12	130926752	130926752	C	T	exonic	RIMBP2		nonsynonymous SNV	RIMBP2:NM_015347:exon9:c.G1094A:p.R	COSMIC	ID=COSM568304	153	151	1.254	1	239	2	0.0416742	1								
13	chr12	48368571	48368571	C	T	exonic	COL2A1		nonsynonymous SNV	COL2A1:NM_033150:exon51:c.G3754A:p.E	DBSNP		82	89	1.19201	1	177	68	0.952983	2								
13	chr10	81318726	81318726	G	A	exonic	SFTPA2		nonsynonymous SNV	SFTPA2:NM_001320814:exon3:c.C238T:p.	NOVEL		120	66	1.16751	1	159	23	0.972128	1								
13	chr1	2526729	2526729	C	T	exonic	MMEL1		nonsynonymous SNV	MMEL1:NM_033467:exon16:c.G1570A:p.E	DBSNP		89	209	1.16327	2	81	168	1.3085	4								
13	chr17	7792353	7792353	G	A	exonic	CHD3		nonsynonymous SNV	CHD3:NM_001005273:exon1:c.G35A:p.R12	NOVEL		130	71	1.16223	1	107	52	1.12292	2								
13	chr10	119305213	119305213	C	T	exonic	EMX2		synonymous SNV	EMX2:NM_004098:exon2:c.C477T:p.T159T	DBSNP		191	101	1.13807	1	177	90	1.29649	2								
13	chr1	27876299	27876299	C	T	exonic	AHDC1		stopgain	AHDC1:NM_001029882:exon6:c.G2328A:p	NOVEL		318	166	1.13774	1	430	47	0.764367	1								
13	chr8	54742092	54742092	C	T	exonic	ATP6V1H		nonsynonymous SNV	ATP6V1H:NM_015941:exon4:c.G218A:p.C	NOVEL		64	63	1.13611	1	129	43	1.10839	2								
13	chr16	27789936	27789936	C	T	exonic	KIAA0556		stopgain	KIAA0556:NM_015202:exon28:c.C4843T:p	DBSNP+COSMIC	ID=COSM669333	106	100	1.11178	1	148	112	1.10456	3								
13	chr6	7556031	7556031	G	A	exonic	DSP		nonsynonymous SNV	DSP:NM_001008844:exon2:c.G251A:p.R84	DBSNP		300	159	1.11152	1	355	98	0.850966	2								
13	chr2	27157489	27157489	G	A	exonic	DPYSL5		synonymous SNV	DPYSL5:NM_001253723:exon8:c.G834A:p.	NOVEL		55	31	1.10323	1	57	13	1.46103	1								
13	chr6	26031882	26031882	G	A	exonic	HIST1H3B		nonsynonymous SNV	HIST1H3B:NM_003537:exon1:c.C407T:p.A	DBSNP		104	226	1.09874	2	170	206	1.43672	3								
13	chr20	20278961	20278961	G	T	exonic	CFAP61		nonsynonymous SNV	CFAP61:NM_015585:exon25:c.G3353T:p.G	COSMIC	ID=COSM723444	100	92	1.09742	1	163	77	1.10159	2								
13	chr8	118817026	118817026	C	T	exonic	EXT1		nonsynonymous SNV	EXT1:NM_000127:exon10:c.G1990A:p.V66	NOVEL		102	93	1.09228	1	158	264	1.23455	5								
13	chr4	147561600	147561600	C	T	exonic	POU4F2		synonymous SNV	POU4F2:NM_004575:exon2:c.C870T:p.G29	NOVEL		292	143	1.0905	1	332	153	1.24089	2								
13	chr9	124976591	124976591	G	A	exonic	LHX6		nonsynonymous SNV	LHX6:NM_001242335:exon3:c.C106T:p.R3	NOVEL		206	86	1.08799	1	210	51	0.964025	2								
13	chr17	17696875	17696875	C	T	exonic	RAI1		stopgain	RAI1:NM_030665:exon3:c.C613T:p.Q205X	NOVEL		346	167	1.0711	1	466	0	0	1								
13	chr5	149459633	149459633	G	A	exonic	CSF1R		nonsynonymous SNV	CSF1R:NM_001288705:exon3:c.C574T:p.R	DBSNP+COSMIC	ID=COSM106427	95	50	1.05537	1	119	40	0.989572	2								
13	chr16	14698035	14698035	T	C	exonic	PARN		nonsynonymous SNV	PARN:NM_001242992:exon10:c.A613G:p.	COSMIC	ID=COSM728151	432	366	1.05042	1	642	418	1.01115	3								
13	chr1	2434266	2434266	C	T	exonic	PLCH2		nonsynonymous SNV	PLCH2:NM_001303012:exon21:c.C3296T:p	DBSNP		221	102	1.04756	1	257	34	0.906376	1								
13	chr2	197189873	197189873	T	A	exonic	HECW2		nonsynonymous SNV	HECW2:NM_001348768:exon6:c.A572T:p.	NOVEL		66	34	1.04059	1	84	39	1.24722	2								
13	chr1	115256529	115256529	T	C	exonic	NRAS		nonsynonymous SNV	NRAS:NM_002524:exon3:c.A182G:p.Q61R	DBSNP+COSMIC	ID=COSM584;OC	108	89	1.03469	1	144	68	1.10132	2								
13	chr21	45978017	45978017	G	A	exonic	KRTAP10-3		synonymous SNV	KRTAP10-3:NM_198696:exon1:c.C582T:p.	DBSNP		133	51	1.02391	1	168	44	1.02394	2								
13	chr6	26216758	26216758	G	T	exonic	HIST1H2BG		stopgain	HIST1H2BG:NM_003518:exon1:c.C114A:p.	NOVEL		83	146	1.02287	2	133	137	1.33061	3								
13	chr1	249142357	249142357	G	A	exonic	ZNF672		nonsynonymous SNV	ZNF672:NM_024836:exon4:c.G884A:p.R29	COSMIC	ID=COSM683228	166	83	1.01	1	351	79	0.998255	2								
13	chr9	113538102	113538102	G	A	exonic	MUSK		nonsynonymous SNV	MUSK:NM_001166281:exon8:c.G955A:p.A	DBSNP+COSMIC	ID=COSM110398	106	52	1.00727	1	152	39	0.803185	2								
13	chr19	35837519	35837519	T	C	exonic	CD22		synonymous SNV	CD22:NM_001185101:exon12:c.T1932C:p.	NOVEL		189	193	1.0069	2	117	148	1.46457	3								
13	chr1	29447550	29447550	C	T	exonic	TMEM200B		nonsynonymous SNV	TMEM200B:NM_001003682:exon2:c.G791	NOVEL		306	132	0.999725	1	332	137	1.13303	2								
13	chr2	40656479	40656479	C	G	exonic	SLC8A1		nonsynonymous SNV	SLC8A1:NM_001112800:exon1:c.G942C:p.	NOVEL		262	489	0.996415	2	401	380	1.27593	3								
13	chr18	32822714	32822714	G	C	exonic	ZNF397		nonsynonymous SNV	ZNF397:NM_001135178:exon2:c.G280C:p.	NOVEL		108	52	0.994683	1	136	46	0.994194	2								
13	chr2	85991193	85991193	C	T	exonic	ATOH8		nonsynonymous SNV	ATOH8:NM_032827:exon2:c.C848T:p.A283	NOVEL		114	192	0.960177	2	167	152	1.24953	3								
13	chr2	39440612	39440612	C	G	exonic	CDKL4		nonsynonymous SNV	CDKL4:NM_001009565:exon3:c.G292C:p.V	NOVEL		109	49	0.949162	1	121	47	1.10046	2								
13	chr2	96810582	96810582	C	T	exonic	DUSP2		nonsynonymous SNV	DUSP2:NM_004418:exon2:c.G428A:p.C143	DBSNP		105	170	0.945992	2	177	174	1.29998	3								
13	chr5	76932978	76932978	C	T	exonic	OTP		nonsynonymous SNV	OTP:NM_032109:exon2:c.G115A:p.G39R	DBSNP		110	49	0.943192	1	108	52	1.2784	2								
13	chr4	68424539	68424539	G	A	exonic	STAP1		synonymous SNV	STAP1:NM_001317769:exon1:c.G12A:p.K4	DBSNP		311	123	0.940148	1	273	101	1.06227	2								
13	chr2	21228920	21228920	G	A	exonic	APOB		nonsynonymous SNV	APOB:NM_000384:exon26:c.C10820T:p.P3	NOVEL		199	88	0.93843	1	246	70	0.871356	2								
13	chr12	113593202	113593202	C	G	exonic	CFAP73		nonsynonymous SNV	CFAP73:NM_001144872:exon6:c.C828G:p.	NOVEL		68	47	0.93602	1	117	56	1.11143	2								
13	chr1	170129773	170129773	T	A	exonic	METTL11B		nonsynonymous SNV	METTL11B:NM_001136107:exon2:c.T269A	NOVEL		311	134	0.921882	1	526	47	0.72732	1								
13	chr10	22605405	22605405	T	G	exonic	COMMD3;COMMD		nonsynonymous SNV	COMMD3-BMI1:NM_001204062:exon1:c.T	NOVEL		109	197	0.913001	2	158	173	1.15862	4								
13	chr7	30951568	30951568	T	C	exonic	AQP1		nonsynonymous SNV	AQP1:NM_001329872:exon1:c.T44C:p.V15	NOVEL		126	122	0.908628	2	179	105	1.21601	3								
13	chr15	34395795	34395795	C	A	exonic	PGBD4		nonsynonymous SNV	PGBD4:NM_152595:exon1:c.C1063A:p.R35	DBSNP		607	198	0.90861	1	782	203	1.01676	2								
13	chr22	18907238	18907238	C	T	exonic	LOC102724788;PR		nonsynonymous SNV	PRODH:NM_001195226:exon9:c.G761A:p.	DBSNP		131	85	0.901261	1	159	67	1.01791	2								
13	chr3	124209726	124209726	C	A	exonic	KALRN		nonsynonymous SNV	KALRN:NM_001322992:exon29:c.C4426A:	NOVEL		211	67	0.890304	1	217	39	0.751594	2								
13	chr7	106508506	106508506	A	G	exonic	PIK3CG		nonsynonymous SNV	PIK3CG:NM_001282426:exon2:c.A500G:p.	NOVEL		111	100	0.875378	2	157	109	1.34776	3								
13	chr17	2593927	2593927	G	T	exonic	CLUH		synonymous SNV	CLUH:NM_001366661:exon26:c.C4008A:p.	NOVEL		161	58	0.871394	1	129	54	1.01318	2								
13	chr22	31286739	31286739	A	G	exonic	OSBP2		nonsynonymous SNV	OSBP2:NM_001282742:exon6:c.A280G:p.I	NOVEL		111	68	0.870044	1	136	52	0.949703	2								
13	chr5	141358390	141358390	G	T	exonic	RNF14		nonsynonymous SNV	RNF14:NM_183398:exon4:c.G451T:p.G15	NOVEL		115	45	0.860783	1	108	36	0.983387	2								
13	chr2	159166060	159166060	A	C	exonic	CCDC148		nonsynonymous SNV	CCDC148:NM_001301684:exon7:c.T557G:	NOVEL		185	72	0.857434	1	248	60	0.766276	2								
13	chr19	1440232	1440232	T	C	exonic	RPS15		nonsynonymous SNV	RPS15:NM_001018:exon3:c.T304C:p.F102	NOVEL		253	208	0.833375	2	299	193	1.15945	3								
13	chr3	39230332	39230332	C	T	exonic	XIRP1		nonsynonymous SNV	XIRP1:NM_001198621:exon2:c.G605A:p.R	DBSNP		443	115	0.821462	1	347	135	1.10172	2								
13	chr7	45744149	45744149	G	T	exonic	ADCY1		nonsynonymous SNV	ADCY1:NM_021116:exon17:c.G2751T:p.E9	NOVEL		111	86	0.806325	2	136	92	1.32715	3								
13	chr19	35757601	35757601	T	C	exonic	LSR		synonymous SNV	LSR:NM_001260490:exon4:c.T792C:p.Y264	NOVEL		87	59	0.805366	2	88	22	0.78671	2								
13	chr18	11888719	11888719	C	T	exonic	MPPE1		nonsynonymous SNV	MPPE1:NM_001242904:exon5:c.G518A:p.	DBSNP		80	28	0.793479	1	92	22	0.759106	2								
13	chr9	137774406	137774406	G	T	exonic	FCN2		nonsynonymous SNV	FCN2:NM_004108:exon2:c.G135T:p.K45N	NOVEL		174	47	0.785622	1	180	31	1.44967	1								
13	chr10	26463047	26463047	T	C	exonic	MYO3A		nonsynonymous SNV	MYO3A:NM_017433:exon30:c.T3854C:p.L	NOVEL		480	17	0.0970173	1	620	1	0.0142788	1								
13	chr11	130078520	130078520	A	T	exonic	ST14		nonsynonymous SNV	ST14:NM_021978:exon17:c.A2210T:p.H73	NOVEL		300	1	0.0132421	1	165	49	0.900672	2								
13	chr10	50034774	50034774	T	A	exonic	WDFY4		stopgain	WDFY4:NM_020945:exon36:c.T6041A:p.L	NOVEL		355	1	0.00924231	1	177	69	1.07882	2								
13	chr5	1882085	1882085	G	A	exonic	IRX4		nonsynonymous SNV	IRX4:NM_016358:exon2:c.C134T:p.A45V,I	DBSNP		358	1	0.00852525	1	267	108	1.13286	2								
13	chr14	26917499	26917499	C	T	exonic	NOVA1		nonsynonymous SNV	NOVA1:NM_001366394:exon4:c.G1007A:	NOVEL		275	1	0.00829805	1	413	19	0.302024	1								
13	chr1	26085158	26085158	A	C	exonic	MAN1C1		nonsynonymous SNV	MAN1C1:NM_001289010:exon6:c.A1005C	NOVEL		918	1	0.00360965	1	566	235	1.13796	2								
13	chr1	3421775	3421775	G	T	exonic	MEGF6		nonsynonymous SNV	MEGF6:NM_001409:exon17:c.C2185A:p.Q	DBSNP		249	0	0	1	174	72	1.13525	2								
13	chr1	16259291	16259291	G	A	exonic	SPEN		nonsynonymous SNV	SPEN:NM_015001:exon11:c.G6556A:p.A21	COSMIC	ID=COSM692061	330	0	0	1	281	48	1.1318	1								
13	chr1	173776408	173776408	G	C	exonic	CENPL		synonymous SNV	CENPL:NM_001171182:exon3:c.C417G:p.V	NOVEL		175	0	0	1	185	15	0.665034	1								
13	chr1	201029843	201029843	G	A	exonic	CACNA1S		synonymous SNV	CACNA1S:NM_000069:exon26:c.C3357T:p.	NOVEL		197	0	0	1	213	9	0.359478	1								
13	chr10	61819757	61819757	A	C	exonic	ANK3		synonymous SNV	ANK3:NM_001149:exon17:c.T2166G:p.P72	NOVEL		118	0	0	1	82	40	1.26106	2								
13	chr10	102241681	102241681	G	C	exonic	WNT8B		nonsynonymous SNV	WNT8B:NM_003393:exon5:c.G380C:p.W1	NOVEL		120	0	0	1	64	22	0.983923	2								
13	chr10	134941945	134941945	C	T	exonic	ADGRA1		nonsynonymous SNV	ADGRA1:NM_001291085:exon4:c.C322T:p	NOVEL		125	0	0	1	63	31	1.26844	2								
13	chr13	32352682	32352682	C	G	exonic	RXFP2		synonymous SNV	RXFP2:NM_001166058:exon9:c.C747G:p.P	NOVEL		61	0	0	1	55	14	1.39332	1								
13	chr14	62244800	62244800	T	G	exonic	SNAPC1		stopgain	SNAPC1:NM_003082:exon6:c.T704G:p.L23	NOVEL		93	0	0	1	61	50	1.03109	3								
13	chr16	2818093	2818093	G	A	exonic	SRRM2		nonsynonymous SNV	SRRM2:NM_016333:exon11:c.G7564A:p.A	NOVEL		184	0	0	1	138	115	1.16553	3								
13	chr16	89644066	89644066	G	A	exonic	CPNE7		synonymous SNV	CPNE7:NM_014427:exon2:c.G294A:p.G98	DBSNP		83	0	0	1	72	48	1.04895	3								
13	chr17	12901770	12901770	C	T	exonic	ELAC2		synonymous SNV	ELAC2:NM_001165962:exon15:c.G1359A:	DBSNP		332	0	0	1	266	17	0.41251	1								
13	chr17	32965210	32965210	G	A	exonic	TMEM132E		nonsynonymous SNV	TMEM132E:NM_001304438:exon9:c.G318	NOVEL		153	0	0	1	134	22	1.10946	1								
13	chr17	78269503	78269503	G	A	exonic	RNF213		synonymous SNV	RNF213:NM_001256071:exon10:c.G1902A	NOVEL		597	0	0	1	671	26	0.293464	1								
13	chr18	32470361	32470361	T	[illegible]	exonic	DTNA		nonsynonymous SNV	DTNA:NM_001198938:exon20:c.T2132A:p	NOVEL		282	0	0	1	243	75	0.927724	2								
13	chr19	41726702	41726702	C	A	exonic	AXL		nonsynonymous SNV	AXL:NM_001699:exon2:c.C247A:p.Q83K,A	NOVEL		342	0	0	1	193	71	1.05789	2								
13	chr2	33487855	33487855	A	T	exonic	LTBP1		nonsynonymous SNV	LTBP1:NM_000627:exon10:c.A1507T:p.I50	NOVEL		534	0	0	1	623	35	0.418463	1								
13	chr2	65217224	65217224	G	A	exonic	SLC1A4		synonymous SNV	SLC1A4:NM_003038:exon1:c.G447A:p.Q14	NOVEL		265	0	0	1	193	63	0.968022	2								
13	chr20	982789	982789	G	T	exonic	RSPO4		nonsynonymous SNV	RSPO4:NM_001029871:exon1:c.C19A:p.L7	COSMIC	ID=COSM444148	231	0	0	1	171	91	1.19257	2								
13	chr22	21097019	21097019	C	G	exonic	PI4KA		nonsynonymous SNV	PI4KA:NM_001362862:exon30:c.G3397C:p	NOVEL		169	0	0	1	144	67	1.09027	2								
13	chr22	30064349	30064349	G	T	exonic	NF2		nonsynonymous SNV	NF2:NM_181830:exon8:c.G664T:p.D222Y,	NOVEL		98	0	0	1	71	28	0.971102	2								
13	chr22	30064368	30064368	G	T	exonic	NF2		nonsynonymous SNV	NF2:NM_181830:exon8:c.G683T:p.R228M	NOVEL		102	0	0	1	79	26	0.85021	2								
13	chr22	30064369	30064369	G	A	exonic	NF2		synonymous SNV	NF2:NM_181830:exon8:c.G684A:p.R228R,	NOVEL		103	0	0	1	79	26	0.85021	2								
13	chr3	42777285	42777285	G	T	exonic	CCDC13		nonsynonymous SNV	CCDC13:NM_144719:exon10:c.C1285A:p.L	NOVEL		231	0	0	1	125	45	1.04123	2								
13	chr3	121252020	121252020	C	A	exonic	POLQ		synonymous SNV	POLQ:NM_199420:exon6:c.G777T:p.V259	NOVEL		523	0	0	1	509	14	0.264128	1								
13	chr4	52938309	52938309	G	C	exonic	SPATA18		nonsynonymous SNV	SPATA18:NM_001346103:exon2:c.G70C:p	NOVEL		274	0	0	1	235	24	0.728996	1								
13	chr4	77662433	77662433	A	G	exonic	SHROOM3		nonsynonymous SNV	SHROOM3:NM_020859:exon5:c.A3107G:p	DBSNP		233	0	0	1	223	35	1.06724	1								
13	chr4	140307320	140307320	G	A	exonic	NAA15		nonsynonymous SNV	NAA15:NM_057175:exon19:c.G2336A:p.S	NOVEL		506	0	0	1	343	162	1.26185	2								
13	chr5	7626285	7626285	G	A	exonic	ADCY2		synonymous SNV	ADCY2:NM_020546:exon4:c.G576A:p.L192	NOVEL		528	0	0	1	436	147	0.991821	2								
13	chr5	35026546	35026546	G	A	exonic	AGXT2		nonsynonymous SNV	AGXT2:NM_001306173:exon8:c.C839T:p.S	NOVEL		464	0	0	1	329	128	1.10174	2								
13	chr5	76507086	76507086	G	T	exonic	PDE8B		synonymous SNV	PDE8B:NM_001029851:exon1:c.G336T:p.V	NOVEL		179	0	0	1	126	19	1.03086	1								
13	chr5	176666833	176666833	G	C	exonic	NSD1		nonsynonymous SNV	NSD1:NM_022455:exon8:c.G4269C:p.M14	NOVEL		202	0	0	1	172	55	0.953063	2								
13	chr5	180055973	180055973	G	C	exonic	FLT4		nonsynonymous SNV	FLT4:NM_001354989:exon8:c.C1012G:p.L	NOVEL		286	0	0	1	234	77	0.973901	2								
13	chr6	394983	394983	A	G	exonic	IRF4		nonsynonymous SNV	IRF4:NM_001195286:exon3:c.A379G:p.I12	NOVEL		310	0	0	1	231	74	0.954369	2								
13	chr6	26250471	26250471	C	T	exonic	HIST1H3F		nonsynonymous SNV	HIST1H3F:NM_021018:exon1:c.G363A:p.N	NOVEL		385	0	0	1	296	93	0.940411	2								
13	chr6	166580999	166580999	C	G	exonic	TBXT		nonsynonymous SNV	TBXT:NM_001366285:exon1:c.G81C:p.E27	NOVEL		246	0	0	1	175	111	1.3326	2								
13	chr7	70227888	70227888	C	A	exonic	AUTS2		nonsynonymous SNV	AUTS2:NM_001127231:exon7:c.C775A:p.H	NOVEL		138	0	0	1	119	15	0.880645	1								
13	chr8	37978473	37978473	G	A	exonic	ASH2L		nonsynonymous SNV	ASH2L:NM_001282272:exon9:c.G554A:p.R	COSMIC	ID=COSM145687	126	0	0	1	101	70	1.02784	2								
13	chr8	41166620	41166620	G	A	exonic	SFRP1		nonsynonymous SNV	SFRP1:NM_003012:exon1:c.C59T:p.A20V	COSMIC	ID=COSM599777	51	0	0	1	64	21	1.24067	1								
13	chr9	95947835	95947835	G	A	exonic	WNK2		synonymous SNV	WNK2:NM_001282394:exon1:c.G624A:p.T	COSMIC	ID=COSM390896	280	0	0	1	238	55	0.926091	2								
13	chr4	106194076	106194076	G	C	splicing	TET2	NM_001127208:exon1			COSMIC	ID=COSM690770	126	208	1.03292	2	124	173	1.14563	4								
13	chr5	32388781	32388781	C	T	splicing	ZFR	NM_016107:exon13:c			NOVEL		276	0	0	1	197	80	1.13604	2								
13	chr5	179042597	179042597	C	T	splicing	HNRNPH1	NM_001364250:exon1			NOVEL		73	0	0	1	51	18	1.02614	2								
14	chrX	54040828	54040828	G	A	exonic	PHF8		synonymous SNV	PHF8:NM_001184896:exon7:c.C873T:p.V2	NOVEL		119	61	NA	1	164	70	NA	1	126	65	NA	1	92	41	NA	1
14	chr2	242644101	242644101	A	T	exonic	ING5		nonsynonymous SNV	ING5:NM_001330162:exon2:c.A71T:p.N24	NOVEL		310	1	NA	1	310	0	NA	1	171	46	NA	1	65	56	NA	1
14	chr12	54676217	54676217	T	C	exonic	HNRNPA1		nonsynonymous SNV	HNRNPA1:NM_002136:exon5:c.T530C:p.V	NOVEL		66	47	0.955651	1	71	58	0.977255	1	41	25	0.902811	1	4	6	1.49473	1
14	chr9	106900378	106900378	G	A	exonic	SMC2		nonsynonymous SNV	SMC2:NM_001042550:exon24:c.G3349A:p	NOVEL		344	732	1.44713	2	345	838	1.4628	2	267	579	1	3	148	282	1.46148	2
14	chrX	153215850	153215850	G	C	exonic	HCFC1		nonsynonymous SNV	HCFC1:NM_005334:exon24:c.C5848G:p.R	NOVEL		94	212	1.41471	2	82	215	1.45561	2	108	235	1.43594	2	72	138	1.39499	2
14	chr16	88712555	88712555	C	T	exonic	CYBA		nonsynonymous SNV	CYBA:NM_000101:exon5:c.G338A:p.C113Y	NOVEL		77	0	0	1	77	0	0	1	45	21	0.758361	1	9	10	1.31117	1
14	chr16	29827853	29827853	C	T	exonic	PAGR1		nonsynonymous SNV	PAGR1:NM_024516:exon1:c.C7T:p.L3F	NOVEL		86	0	0	1	38	0	0	1	52	19	0.637817	1	23	25	1.29751	1
14	chr15	101853708	101853708	G	A	exonic	PCSK6		unknown	UNKNOWN	NOVEL		70	0	0	1	68	0	0	1	73	23	0.571028	1	26	28	1.29175	1
14	chr3	37774247	37774247	C	T	exonic	ITGA9		synonymous SNV	ITGA9:NM_002207:exon19:c.C2112T:p.L70	NOVEL		203	137	0.92581	1	173	168	1.07084	1	109	76	0.979135	1	49	52	1.28261	1
14	chr12	31135479	31135479	G	A	exonic	TSPAN11		nonsynonymous SNV	TSPAN11:NM_001370301:exon5:c.G439A:	DBSNP		75	52	0.940762	1	64	41	0.848719	1	55	33	0.893783	1	32	33	1.26477	1
14	chr4	95204447	95204447	C	G	exonic	SMARCAD1		nonsynonymous SNV	SMARCAD1:NM_001254949:exon14:c.C16	NOVEL		64	23	0.607419	1	31	29	1.05055	1	40	24	0.893783	1	14	14	1.24561	1
14	chr9	15744762	15744762	G	A	exonic	CCDC171		synonymous SNV	CCDC171:NM_001355547:exon16:c.G2565	NOVEL		373	94	0.856348	1	393	111	0.909598	1	227	77	1.11027	1	93	35	1.21871	1
14	chr5	157230723	157230723	G	A	exonic	CLINT1		nonsynonymous SNV	CLINT1:NM_001195555:exon8:c.C947T:p.S	NOVEL		142	43	0.766476	1	149	87	1.16991	1	207	70	0.855016	1	140	75	1.21787	1
14	chr8	30703368	30703368	T	A	exonic	TEX15		stopgain	TEX15:NM_001350162:exon8:c.A4315T:p.	NOVEL		145	138	1.1204	1	134	128	1.06189	1	139	84	0.897791	1	22	21	1.21664	1
14	chr4	17590520	17590520	C	T	exonic	LAP3		synonymous SNV	LAP3:NM_015907:exon7:c.C783T:p.F261F	NOVEL		377	60	0.315464	1	323	140	0.657229	1	167	133	1.05665	1	109	92	1.14026	1
14	chr13	45914890	45914890	A	T	exonic	TPT1		nonsynonymous SNV	TPT1:NM_001286272:exon2:c.T59A:p.I20N	NOVEL		44	150	1.00332	1	24	178	1.03412	1	20	79	1.11979	1	28	90	1.13737	1
14	chr2	96810562	96810562	C	T	exonic	DUSP2		nonsynonymous SNV	DUSP2:NM_004418:exon2:c.G448A:p.A150	NOVEL		150	112	0.982193	1	123	110	1.02614	1	109	100	1.14039	1	50	42	1.1373	1
14	chr9	14784501	14784501	G	A	exonic	FREM1		nonsynonymous SNV	FREM1:NM_144966:exon25:c.C4309T:p.P1	NOVEL		340	80	0.810363	1	305	113	1.1165	1	213	69	1.07254	1	135	46	1.13272	1
14	chr22	32111982	32111982	T	C	exonic	PRR14L		nonsynonymous SNV	PRR14L:NM_173566:exon4:c.A1843G:p.T6	NOVEL		434	230	0.795866	1	370	314	0.997799	1	277	230	1.08124	1	132	110	1.13237	1
14	chr3	58135907	58135907	C	T	exonic	FLNB		nonsynonymous SNV	FLNB:NM_001164319:exon37:c.C6248T:p.	NOVEL		158	137	1.06703	1	145	147	1.09422	1	157	106	0.960618	1	63	52	1.12647	1
14	chr19	620073	620073	G	A	exonic	POLRMT		nonsynonymous SNV	POLRMT:NM_005035:exon12:c.C2771T:p.S	NOVEL		246	108	1.00606	1	246	123	1.05785	1	291	106	0.903382	1	156	74	1.12326	1
14	chr3	50229198	50229198	G	A	exonic	GNAT1		nonsynonymous SNV	GNAT1:NM_000172:exon1:c.G40A:p.E14K	COSMIC	ID=COSM558354	113	95	1.0494	1	94	100	1.12039	1	94	70	1.01731	1	53	43	1.11586	1
14	chr17	8014797	8014797	C	T	exonic	ALOXE3		synonymous SNV	ALOXE3:NM_001369446:exon7:c.G834A:p	NOVEL		74	43	0.844428	1	70	59	0.994104	1	43	33	1.03491	1	47	38	1.11372	1
14	chr12	54741596	54741596	G	C	exonic	COPZ1		synonymous SNV	COPZ1:NM_001271734:exon5:c.G294C:p.L	NOVEL		151	128	1.05411	1	156	133	1.00028	1	131	103	1.04911	1	51	41	1.11022	1
14	chrX	85211350	85211350	T	C	exonic	CHM		nonsynonymous SNV	CHM:NM_000390:exon8:c.A974G:p.K325R	NOVEL		127	121	1.12102	1	150	109	0.914737	1	174	120	0.972825	1	25	20	1.10721	1
14	chr3	49694662	49694662	C	G	exonic	BSN		nonsynonymous SNV	BSN:NM_003458:exon5:c.C7673G:p.S2558	NOVEL		246	222	1.0899	1	242	233	1.06618	1	230	165	0.995606	1	119	95	1.10592	1
14	chr12	6748158	6748158	T	G	exonic	ACRBP		nonsynonymous SNV	ACRBP:NM_032489:exon9:c.A1471C:p.K49	NOVEL		64	58	1.09232	1	75	72	1.0646	1	59	30	0.8034	1	39	31	1.10326	1
14	chr11	65365777	65365777	C	T	exonic	MAP3K11		synonymous SNV	MAP3K11:NM_002419:exon10:c.G2529A:	DBSNP		66	42	0.893523	1	44	48	1.13403	1	36	28	1.04275	1	9	7	1.08991	1
14	chr16	83999559	83999559	G	A	exonic	OSGIN1		nonsynonymous SNV	OSGIN1:NM_182981:exon6:c.G1381A:p.A	DBSNP+COSMIC	ID=COSM699870	192	0	0	1	195	0	0	1	98	47	0.772557	1	45	35	1.08991	1
14	chr1	20072005	20072005	C	T	exonic	TMCO4		synonymous SNV	TMCO4:NM_001349115:exon10:c.G897A:	NOVEL		9	59	1.13332	1	6	50	1.04781	1	14	46	1.06062	1	14	38	1.08974	1
14	chr1	203768595	203768595	A	T	exonic	ZBED6		nonsynonymous SNV	ZBED6:NM_001174108:exon1:c.A1945T:p	NOVEL		683	220	0.803409	1	632	295	1.00992	1	369	166	1.04981	1	150	68	1.08901	1
14	chr17	48447893	48447893	G	A	exonic	MRPL27		nonsynonymous SNV	MRPL27:NM_016504:exon2:c.C95T:p.S32F	NOVEL		162	123	0.991609	1	142	167	1.1747	1	124	102	1.0757	1	93	71	1.07852	1
14	chr5	174156258	174156258	G	A	exonic	MSX2		nonsynonymous SNV	MSX2:NM_002449:exon2:c.G476A:p.R159	DBSNP		460	210	1.03359	1	465	205	0.971011	1	345	147	1.0109	1	262	117	1.07777	1
14	chr9	109690249	109690249	G	A	exonic	ZNF462		synonymous SNV	ZNF462:NM_021224:exon3:c.G4056A:p.G	NOVEL		324	260	0.947043	2	317	289	0.984809	2	217	135	0.840571	2	160	149	1.07458	2
14	chr16	81737650	81737650	A	C	exonic	CMIP		nonsynonymous SNV	CMIP:NM_030629:exon18:c.A1769C:p.K59	NOVEL		139	98	0.950075	1	146	138	1.05616	1	109	86	1.05115	1	148	112	1.07314	1
14	chrX	100746175	100746175	T	C	exonic	ARMCX4		nonsynonymous SNV	ARMCX4:NM_001256155:exon2:c.T2599C	NOVEL		362	271	0.983661	1	303	263	1.00997	1	259	169	0.941117	1	110	83	1.07135	1
14	chr9	712909	712909	C	T	exonic	KANK1		nonsynonymous SNV	KANK1:NM_001354335:exon2:c.C1669T:p.	DBSNP		900	0	0	1	967	0	0	1	601	121	0.734618	1	367	116	1.07042	1
14	chr7	117008744	117008744	G	T	exonic	ASZ1		synonymous SNV	ASZ1:NM_001301822:exon10:c.C459A:p.L	NOVEL		232	97	0.972249	1	289	139	1.03066	1	193	73	0.928533	1	99	43	1.0572	1
14	chr15	99672451	99672451	G	A	exonic	SYNM		unknown	UNKNOWN	NOVEL		161	124	0.99967	1	187	138	0.922922	1	132	104	1.05032	1	93	68	1.05219	1
14	chr5	32088502	32088502	G	A	exonic	PDZD2		nonsynonymous SNV	PDZD2:NM_178140:exon20:c.G4948A:p.A	DBSNP		626	252	0.946472	1	657	267	0.917032	1	478	184	0.940407	1	257	110	1.04642	1
14	chr2	179641415	179641415	G	T	exonic	TTN		nonsynonymous SNV	TTN:NM_003319:exon27:c.C5038A:p.L168	NOVEL		174	151	1.06751	1	204	186	1.03662	1	143	118	1.07756	1	72	52	1.04471	1
14	chr4	106157342	106157342	T	G	exonic	TET2		stopgain	TET2:NM_001127208:exon3:c.T2243G:p.L	NOVEL		828	0	0	1	835	0	0	1	318	112	0.620798	1	225	162	1.04284	1
14	chr3	164907941	164907941	C	T	exonic	SLITRK3		synonymous SNV	SLITRK3:NM_001318810:exon2:c.G678A:p	NOVEL		276	173	0.885278	1	246	210	1.00098	1	167	125	1.0203	1	64	46	1.04178	1
14	chr3	48608102	48608102	G	A	exonic	COL7A1		synonymous SNV	COL7A1:NM_000094:exon95:c.C7314T:p.P	NOVEL		97	34	0.596331	1	62	60	1.06896	1	64	45	0.983981	1	32	23	1.04178	1
14	chr19	52659595	52659595	G	A	exonic	ZNF836		synonymous SNV	ZNF836:NM_001102657:exon5:c.C1341T:	DBSNP+COSMIC	ID=COSM370734	305	142	1.04757	1	352	152	0.957102	1	397	145	0.905159	1	137	58	1.03842	1
14	chr12	122247775	122247775	C	A	exonic	SETD1B		nonsynonymous SNV	SETD1B:NM_001353345:exon15:c.C924A:p	NOVEL		517	1	0.00443558	1	535	1	0.00405513	1	238	87	0.638023	1	181	128	1.03196	1
14	chr10	71562292	71562292	G	A	exonic	COL13A1		nonsynonymous SNV	COL13A1:NM_001130103:exon1:c.G113A:	NOVEL		77	67	1.06904	1	91	61	0.87228	1	63	46	1.00585	1	17	12	1.03085	1
14	chr12	49176817	49176817	G	A	exonic	ADCY6		nonsynonymous SNV	ADCY6:NM_015270:exon2:c.C401T:p.A134	DBSNP		281	0	0	1	303	0	0	1	149	58	0.667818	1	111	77	1.02034	1
14	chr8	11606570	11606570	G	A	exonic	GATA4		synonymous SNV	GATA4:NM_001308093:exon2:c.G762A:p.	DBSNP		131	123	1.11263	1	112	124	1.14203	1	95	82	1.10418	1	50	34	1.00835	1
14	chr9	139360510	139360510	A	C	exonic	SEC16A		nonsynonymous SNV	SEC16A:NM_001276418:exon7:c.T4207G:	NOVEL		376	123	1.04868	1	396	106	0.872086	1	337	89	0.915785	1	165	48	1.00439	1
14	chrX	107334668	107334668	G	A	exonic	PSMD10		nonsynonymous SNV	PSMD10:NM_002814:exon1:c.C83T:p.A28V	NOVEL		294	1	0.00778857	1	247	0	0	1	130	59	0.744031	1	43	29	1.00341	1
14	chr8	70512918	70512918	T	C	exonic	SULF1		nonsynonymous SNV	SULF1:NM_001128204:exon8:c.T815C:p.M	NOVEL		195	133	0.931661	1	190	152	0.966022	1	191	159	1.08275	1	119	80	1.0015	1
14	chr19	37903573	37903573	G	A	exonic	ZNF569		nonsynonymous SNV	ZNF569:NM_001330482:exon5:c.C1510T:	NOVEL		255	119	1.04925	1	272	110	0.913849	1	251	123	1.11273	1	107	43	1.00082	1
14	chr1	24201293	24201293	G	A	exonic	CNR2		nonsynonymous SNV	CNR2:NM_001841:exon2:c.C815T:p.T272N	DBSNP+COSMIC	ID=COSM425765	203	0	0	1	224	0	0	1	93	103	0.727002	1	40	80	0.994149	1
14	chr7	75630286	75630286	G	C	exonic	STYXL1		nonsynonymous SNV	STYXL1:NM_001317787:exon6:c.C444G:p.	NOVEL		412	133	0.804743	1	414	183	0.972796	1	296	125	1.00458	1	144	57	0.990048	1
14	chr12	25380275	25380275	T	G	exonic	KRAS		nonsynonymous SNV	KRAS:NM_001369786:exon3:c.A183C:p.Q6	DBSNP+COSMIC	ID=COSM554,CO	89	100	1.21568	1	106	84	0.960937	1	82	53	0.935713	1	43	28	0.982454	1
14	chr11	82917147	82917147	A	G	exonic	ANKRD42		synonymous SNV	ANKRD42:NM_001300972:exon3:c.A270G:	NOVEL		285	191	0.921948	1	308	256	0.986575	1	279	186	0.953368	1	154	100	0.980796	1
14	chr12	21028325	21028325	C	T	exonic	SLCO1B3;SLCO1B3		nonsynonymous SNV	SLCO1B3:NM_001349920:exon7:c.C800T:	COSMIC	ID=COSM345911	171	98	0.837055	1	172	168	1.07399	1	87	77	1.11905	1	54	35	0.979694	1
14	chr4	41748027	41748027	C	T	exonic	PHOX2B		nonsynonymous SNV	PHOX2B:NM_003924:exon3:c.G742A:p.A2	COSMIC	ID=COSM548308	62	51	1.03698	1	46	42	1.03738	1	71	57	1.06137	1	17	11	0.978695	1
14	chr9	117848295	117848295	C	T	exonic	TNC		nonsynonymous SNV	TNC:NM_002160:exon3:c.G1715A:p.R572	DBSNP		485	154	1.02532	1	495	158	0.999311	1	381	102	0.925691	1	228	64	0.976876	1
14	chr11	63078489	63078489	C	T	exonic	SLC22A10		nonsynonymous SNV	SLC22A10:NM_001039752:exon10:c.C1609	NOVEL		54	36	0.919052	1	61	67	1.13772	1	62	37	0.890773	1	14	9	0.974826	1
14	chr3	57448467	57448467	G	C	exonic	DNAH12		unknown	UNKNOWN	NOVEL		168	106	0.888864	1	152	120	0.958919	1	104	93	1.12517	1	47	30	0.970606	1
14	chr9	125797530	125797530	C	T	exonic	GPR21		stopgain	GPR21:NM_005294:exon2:c.C685T:p.Q229	NOVEL		587	170	0.955415	1	612	181	0.942676	1	438	116	0.917828	1	184	51	0.967264	1
14	chr7	82451929	82451929	A	G	exonic	PCLO		synonymous SNV	PCLO:NM_014510:exon20:c.T14673C:p.R4	NOVEL		433	196	1.02756	1	482	224	1.00691	1	361	149	0.98849	1	197	75	0.962653	1
14	chr19	44793326	44793326	C	A	exonic	ZNF235		nonsynonymous SNV	ZNF235:NM_004234:exon5:c.G262T:p.A88	NOVEL		436	160	0.88527	1	482	176	0.848852	1	367	148	0.972323	1	134	51	0.962445	1
14	chr14	103395145	103395145	C	A	exonic	AMN		nonsynonymous SNV	AMN:NM_030943:exon5:c.C346A:p.H116N	NOVEL		180	107	0.856608	1	151	136	1.02997	1	109	99	1.13442	1	105	66	0.961525	1
14	chr1	183853914	183853914	G	T	exonic	RGL1		nonsynonymous SNV	RGL1:NM_001297670:exon7:c.G787T:p.D2	COSMIC	ID=COSM463547	246	112	1.03166	1	263	143	1.11778	1	159	48	0.784561	1	58	22	0.960086	1
14	chr14	24730980	24730980	G	A	exonic	TGM1		synonymous SNV	TGM1:NM_000359:exon3:c.C429T:p.R143	DBSNP+COSMIC	ID=COSM349542	222	191	1.06258	1	222	172	0.948859	1	131	119	1.13451	1	99	62	0.959353	1
14	chr14	103395144	103395144	G	T	exonic	AMN		synonymous SNV	AMN:NM_030943:exon5:c.G345T:p.P115P	NOVEL		181	105	0.843535	1	149	137	1.04118	1	109	100	1.14039	1	106	66	0.955934	1
14	chr1	164815839	164815839	G	A	exonic	PBX1		nonsynonymous SNV	PBX1:NM_001353130:exon8:c.G970A:p.D3	NOVEL		247	79	0.799119	1	215	112	1.08696	1	169	75	1.03999	1	122	46	0.95593	1
14	chr19	16687394	16687394	T	C	exonic	MED26		nonsynonymous SNV	MED26:NM_004831:exon3:c.A1247G:p.K4	NOVEL		281	396	0.964447	2	261	392	0.952551	2	174	264	1.01966	2	123	147	0.950388	2
14	chr1	240071662	240071662	A	G	exonic	CHRM3		nonsynonymous SNV	CHRM3:NM_000740:exon5:c.A911G:p.N30	NOVEL		635	0	0	1	653	0	0	1	266	62	0.639549	1	131	49	0.950388	1
14	chr7	23775284	23775284	T	C	exonic	STK31		nonsynonymous SNV	STK31:NM_001260504:exon7:c.T542C:p.L1	NOVEL		653	2	0.0100691	1	718	1	0.00441384	1	505	105	0.582392	1	209	78	0.948834	1
14	chr11	66330279	66330279	T	C	exonic	ACTN3		unknown	UNKNOWN	DBSNP		183	149	1.03117	1	197	155	0.957103	1	214	130	0.900711	1	94	57	0.940395	1
14	chr5	140753843	140753843	C	T	exonic	PCDHGA6		nonsynonymous SNV	PCDHGA6:NM_018919:exon1:c.C193T:p.R6	NOVEL		327	102	0.784052	1	299	116	0.887064	1	268	90	0.850581	1	114	42	0.939945	1
14	chr19	57640878	57640878	C	A	exonic	USP29		nonsynonymous SNV	USP29:NM_020903:exon4:c.C835A:p.H279	NOVEL		484	189	0.92608	1	433	228	1.09466	1	355	155	1.02829	1	134	48	0.920762	1
14	chr4	55968116	55968116	C	T	exonic	KDR		synonymous SNV	KDR:NM_002253:exon15:c.G2214A:p.Q73	NOVEL		181	112	0.878275	1	182	132	0.913721	1	111	63	0.862963	1	82	48	0.919836	1
14	chr8	92145377	92145377	A	T	exonic	LRRC69		nonsynonymous SNV	LRRC69:NM_001129890:exon4:c.A423T:p.	NOVEL		78	72	1.10286	1	96	62	0.852912	1	73	41	0.857195	1	19	11	0.913448	1
14	chr7	57188701	57188701	C	T	exonic	ZNF479		nonsynonymous SNV	ZNF479:NM_001370129:exon4:c.G421A:p	DBSNP+COSMIC	ID=COSM496593	463	176	NA	1	490	223	0.992569	1	523	192	0.908555	1	232	82	0.911721	1
14	chr19	49116462	49116462	C	T	exonic	FAM83E		stopgain	FAM83E:NM_017708:exon1:c.G168A:p.W	NOVEL		273	0	0	1	249	0	0	1	150	45	0.780789	1	119	42	0.910754	1
14	chr3	183873517	183873517	G	A	exonic	DVL3		nonsynonymous SNV	DVL3:NM_004423:exon1:c.G94A:p.D32N	DBSNP		197	149	0.989442	1	171	134	0.954936	1	158	127	1.06209	1	113	65	0.909716	1
14	chr11	66482736	66482736	C	T	exonic	SPTBN2		nonsynonymous SNV	SPTBN2:NM_006946:exon4:c.G440A:p.R14	NOVEL		121	85	0.948051	1	105	73	0.891399	1	132	80	0.899404	1	70	40	0.905899	1
14	chr17	61607501	61607501	C	T	exonic	KCNH6		synonymous SNV	KCNH6:NM_001278919:exon3:c.C357T:p.D	DBSNP+COSMIC	ID=COSM279513	45	42	1.1092	1	47	39	0.985679	1	44	27	0.906371	1	23	13	0.899608	1
14	chr6	116752142	116752142	G	T	exonic	DSE		nonsynonymous SNV	DSE:NM_001080976:exon4:c.G696T:p.W2	NOVEL		62	19	0.53895	1	41	45	1.13732	1	17	19	1.25792	1	16	9	0.89684	1
14	chr11	26463643	26463643	T	C	exonic	ANO3		synonymous SNV	ANO3:NM_031418:exon2:c.T225C:p.T75T,	NOVEL		111	90	1.02879	1	121	75	0.831715	1	78	46	0.884172	1	48	27	0.89684	1
14	chr1	37282785	37282785	T	G	exonic	GRIK3		nonsynonymous SNV	GRIK3:NM_000831:exon13:c.A1967C:p.N6	NOVEL		122	0	0	1	109	0	0	1	48	56	0.744919	1	32	48	0.894734	1
14	chr3	128694689	128694689	G	A	exonic	KIAA1257		synonymous SNV	KIAA1257:NM_001348520:exon6:c.C81T:p	NOVEL		100	62	0.87934	1	105	84	0.966022	1	69	50	1.00144	1	54	30	0.889722	1
14	chrX	69282423	69282423	C	T	exonic	OTUD6A		nonsynonymous SNV	OTUD6A:NM_207320:exon1:c.C49T:p.R17	DBSNP+COSMIC	ID=COSM544792	179	114	0.893958	1	160	152	1.05891	1	119	84	0.986243	1	72	40	0.889722	1
14	chr18	28660075	28660075	T	A	exonic	DSC2		nonsynonymous SNV	DSC2:NM_004949:exon10:c.A1507T:p.S50	NOVEL		417	302	0.965068	1	454	373	0.980331	1	305	197	0.935326	1	126	70	0.889722	1
14	chr8	6669358	6669358	G	A	exonic	XKR5		synonymous SNV	XKR5:NM_207411:exon8:c.C1422T:p.V474	NOVEL		235	182	1.0028	1	193	194	1.08958	1	156	104	0.953368	1	87	48	0.885768	1
14	chr5	40854460	40854460	C	T	exonic	CARD6		nonsynonymous SNV	CARD6:NM_032587:exon3:c.C3026T:p.P10	NOVEL		344	125	0.878899	1	354	128	0.842768	1	178	86	1.10217	1	165	56	0.884654	1
14	chr12	101316041	101316041	G	A	exonic	ANO4		nonsynonymous SNV	ANO4:NM_001286616:exon2:c.G93A:p.M	NOVEL		200	159	1.01761	1	189	174	1.04187	1	181	131	1.00073	1	137	75	0.881329	1
14	chr16	28914755	28914755	C	T	exonic	ATP2A1		synonymous SNV	ATP2A1:NM_001286075:exon19:c.C2599T	NOVEL		215	173	1.02446	1	191	167	1.01392	1	187	142	1.02871	1	174	95	0.8798	1
14	chr3	139258377	139258377	C	T	exonic	RBP1		nonsynonymous SNV	RBP1:NM_001130992:exon1:c.G184A:p.E6	NOVEL		143	102	0.956564	1	148	109	0.921855	1	95	56	0.883918	1	88	48	0.879255	1
14	chr20	1162213	1162213	T	A	exonic	TMEM74B		nonsynonymous SNV	TMEM74B:NM_018354:exon2:c.A50T:p.D1	NOVEL		187	0	0	1	200	0	0	1	140	52	0.64551	1	55	30	0.879255	1
14	chr12	88560150	88560150	C	G	exonic	TMTC3		nonsynonymous SNV	TMTC3:NM_001366583:exon5:c.C148G:p.	NOVEL		319	209	0.909478	1	418	299	0.906403	1	285	182	0.928871	1	158	86	0.878054	1
14	chr12	18891397	18891397	C	T	exonic	CAPZA3		synonymous SNV	CAPZA3:NM_033328:exon1:c.C195T:p.I65I	DBSNP+COSMIC	ID=COSM572496	581	274	0.736316	1	473	419	1.02098	1	385	257	0.954111	1	178	96	0.872837	1
14	chr7	90895592	90895592	G	A	exonic	FZD1		nonsynonymous SNV	FZD1:NM_003505:exon1:c.G1397A:p.G466	NOVEL		740	0	0	1	637	1	0.00497421	1	410	91	0.614553	1	199	66	0.869512	1
14	chr2	47357389	47357389	A	G	exonic	STPG4		nonsynonymous SNV	STPG4:NM_001163561:exon4:c.T410C:p.L	NOVEL		236	210	1.08184	1	325	240	0.923277	1	256	162	0.923718	1	206	110	0.867198	1
14	chr5	13911601	13911601	C	T	exonic	DNAH5		nonsynonymous SNV	DNAH5:NM_001369:exon12:c.G1538A:p.G	COSMIC	ID=COSM360922	301	125	0.967614	1	345	131	0.873393	1	210	81	0.941777	1	103	34	0.866435	1
14	chrX	122538637	122538637	C	G	exonic	GRIA3		nonsynonymous SNV	GRIA3:NM_000828:exon10:c.C1372G:p.L4	NOVEL		272	182	0.921076	1	301	230	0.941462	1	201	151	1.02243	1	111	59	0.864601	1
14	chr14	91952003	91952003	A	C	exonic	PPP4R3A		nonsynonymous SNV	PPP4R3A:NM_001284280:exon3:c.T270G:	NOVEL		76	57	0.984698	1	86	70	0.97531	1	76	44	0.873921	1	36	19	0.860604	1
14	chr5	140181225	140181225	C	G	exonic	PCDHA3		nonsynonymous SNV	PCDHA3:NM_018906:exon1:c.C443G:p.P14	NOVEL		312	110	0.859572	1	346	139	0.909533	1	191	80	0.998796	1	83	27	0.856937	1
14	chr2	96810082	96810082	A	T	exonic	DUSP2		nonsynonymous SNV	DUSP2:NM_004418:exon3:c.T541A:p.F181	NOVEL		110	93	1.05261	1	105	76	0.91265	1	116	68	0.880829	1	54	28	0.850662	1
14	chr4	106155307	106155307	C	T	exonic	TET2		stopgain	TET2:NM_001127208:exon3:c.C208T:p.Q7	NOVEL		294	236	1.0231	1	281	278	1.08094	1	207	150	1.00144	1	97	50	0.847355	1
14	chr16	65005886	65005886	G	A	exonic	CDH11		nonsynonymous SNV	CDH11:NM_001330576:exon9:c.C1094T:p.	NOVEL		134	99	0.976246	1	163	130	0.964373	1	143	90	0.920635	1	70	36	0.846076	1
14	chr1	240370959	240370959	C	T	exonic	FMN2		synonymous SNV	FMN2:NM_020066:exon5:c.C2847T:p.P949	DBSNP+COSMIC	ID=COSM505328	81	0	0	1	70	1	0.0446979	1	29	4	0.410112	1	19	6	0.837894	1
14	chr2	241872188	241872188	C	T	exonic	CROCC2		nonsynonymous SNV	CROCC2:NM_001351305:exon9:c.C1114T:	DBSNP		75	64	1.0579	1	92	57	0.831492	1	59	40	0.962998	1	75	38	0.837756	1
14	chr2	96810084	96810084	A	C	exonic	DUSP2		nonsynonymous SNV	DUSP2:NM_004418:exon3:c.T539G:p.L180	NOVEL		111	90	1.02879	1	106	74	0.89357	1	115	66	0.869093	1	56	28	0.830408	1
14	chr22	29195047	29195047	C	T	exonic	XBP1		nonsynonymous SNV	XBP1:NM_001079539:exon2:c.G322A:p.E1	NOVEL		231	140	0.86703	1	191	150	0.956107	1	155	102	0.945949	1	67	33	0.822104	1
14	chr14	57858434	57858434	G	A	exonic	NAA30		synonymous SNV	NAA30:NM_001011713:exon2:c.G759A:p.	NOVEL		87	65	0.982539	1	82	69	0.993211	1	53	47	1.12021	1	43	21	0.817433	1
14	chr10	101373668	101373668	C	T	exonic	SLC25A28		nonsynonymous SNV	SLC25A28:NM_031212:exon2:c.G305A:p.S	COSMIC	ID=COSM375295	141	0	0	1	143	0	0	1	66	14	0.417099	1	43	21	0.817433	1
14	chr1	33960424	33960424	C	A	exonic	ZSCAN20		nonsynonymous SNV	ZSCAN20:NM_145238:exon8:c.C2480A:p.	NOVEL		32	119	1.02939	1	12	134	1.07709	1	27	112	1.1147	1	20	23	0.797631	1
14	chr16	70818708	70818708	C	T	exonic	VAC14		nonsynonymous SNV	VAC14:NM_018052:exon4:c.G457A:p.G15	NOVEL		104	104	1.14881	1	159	124	0.952368	1	87	66	1.02814	1	32	15	0.795071	1
14	chr9	113213676	113213676	C	T	exonic	SVEP1		nonsynonymous SNV	SVEP1:NM_153366:exon23:c.G3794A:p.R	DBSNP		1009	250	0.844799	1	1137	331	0.931234	1	801	215	0.927594	1	410	88	0.787582	1
14	chr4	159051992	159051992	A	T	exonic	GASK1B		nonsynonymous SNV	GASK1B:NM_001128424:exon4:c.T1298A	NOVEL		34	35	1.16546	1	47	31	0.863846	1	58	32	0.847438	1	26	12	0.786702	1
14	chr2	71012586	71012586	C	A	exonic	FIGLA		synonymous SNV	FIGLA:NM_001004311:exon3:c.G570T:p.V	NOVEL		227	0	0	1	238	1	0.00909435	1	99	41	0.698002	1	33	15	0.778507	1
14	chr19	17628631	17628631	C	G	exonic	PGLS		nonsynonymous SNV	PGLS:NM_012088:exon4:c.C611G:p.T204S	NOVEL		628	0	0	1	686	0	0	1	403	101	0.678027	1	311	89	0.776797	1
14	chr12	102517706	102517706	A	T	exonic	PARPBP		stopgain	PARPBP:NM_001319988:exon2:c.A40T:p.K	NOVEL		320	0	0	1	356	0	0	1	213	85	0.679835	1	78	35	0.771618	1
14	chr3	167090684	167090684	G	C	exonic	ZBBX		nonsynonymous SNV	ZBBX:NM_001199201:exon4:c.C6G:p.N2K,	NOVEL		82	56	0.932371	1	108	91	0.993934	1	86	47	0.842261	1	30	13	0.75316	1

14	chr19	1615497	1615497	G	A	exonic	TCF3		nonsynonymous SNV	TCF3:NM_001351778:exon18:c.C1606T:p.L	NOVEL		347	80	0.617823	1	292	111	0.874104	1	298	92	0.79814	1	102	27	0.730721	1
14	chr2	163030270	163030270	C	A	exonic	FAP		nonsynonymous SNV	FAP:NM_001291807:exon22:c.G1922T:p.G	NOVEL		104	64	0.875287	1	117	86	0.920814	1	65	41	0.921889	1	51	20	0.701753	1
14	chr7	141445381	141445381	G	A	exonic	SSBP1		nonsynonymous SNV	SSBP1:NM_001256510:exon6:c.G400A:p.A	NOVEL		172	0	0	1	241	0	0	1	125	29	0.637138	1	64	16	0.698245	1
14	chr1	165324778	165324778	T	C	exonic	LMX1A		nonsynonymous SNV	LMX1A:NM_001174069:exon2:c.A19G:p.M	NOVEL		422	152	0.87324	1	366	160	0.965338	1	220	80	0.902246	1	146	36	0.690572	1
14	chr19	15224404	15224404	C	G	exonic	SYDE1		nonsynonymous SNV	SYDE1:NM_001300910:exon8:c.C1637G:p.	NOVEL		525	217	0.964401	1	488	209	0.951609	1	364	128	0.88024	1	299	72	0.677542	1
14	chr4	79359794	79359794	G	A	exonic	FRAS1		nonsynonymous SNV	FRAS1:NM_001166133:exon39:c.G5288A:	NOVEL		374	0	0	1	440	0	0	1	331	36	0.233796	1	149	5	0.0808839	1
14	chr17	40997667	40997667	A	C	exonic	AOC2		synonymous SNV	AOC2:NM_001158:exon1:c.A1024C:p.R342	NOVEL		365	0	0	1	320	0	0	1	262	25	0.207615	1	134	4	0.0722094	1
14	chr6	70639366	70639366	A	C	exonic	COL19A1		nonsynonymous SNV	COL19A1:NM_001858:exon6:c.A440C:p.Q1	NOVEL		220	0	0	1	228	0	0	1	216	17	0.173898	1	48	1	0.0508413	1
14	chr15	55930819	55930819	C	T	exonic	PRTG		nonsynonymous SNV	PRTG:NM_173814:exon14:c.G2380A:p.A7	DBSNP		455	0	0	1	559	0	0	1	316	30	0.206655	1	198	4	0.0493311	1
14	chr19	59061337	59061337	G	A	exonic	TRIM28		nonsynonymous SNV	TRIM28:NM_005762:exon15:c.G2128A:p.A	NOVEL		699	0	0	1	712	1	0.00445098	1	508	34	0.212244	1	352	5	0.0488967	1
14	chr7	99217342	99217342	C	T	exonic	ZSCAN25		nonsynonymous SNV	ZSCAN25:NM_001350979:exon2:c.C113T:p	COSMIC	ID=COSM364289	508	0	0	1	490	1	0.00646344	1	371	25	0.2136	1	297	4	0.046395	1
14	chr17	3721687	3721687	C	T	exonic	NCBP3		nonsynonymous SNV	NCBP3:NM_001114118:exon10:c.G1180A:	NOVEL		413	0	0	1	479	0	0	1	283	24	0.186326	1	173	3	0.042464	1
14	chr9	27548619	27548619	C	T	exonic	C9orf72		nonsynonymous SNV	C9orf72:NM_001256054:exon10:c.G1195A	NOVEL		788	0	0	1	908	0	0	1	533	20	0.158532	1	266	2	0.0332612	1
14	chr5	127522309	127522309	A	T	exonic	SLC12A2		nonsynonymous SNV	SLC12A2:NM_001256461:exon26:c.A3577	NOVEL		360	0	0	1	431	1	0.00734618	1	355	39	0.334907	1	108	1	0.0320296	1
14	chr3	48663765	48663765	C	T	exonic	SLC26A6		synonymous SNV	SLC26A6:NM_001281733:exon18:c.G1827	NOVEL		344	0	0	1	326	0	0	1	265	22	0.182701	1	161	2	0.0305672	1
14	chr12	12871192	12871192	G	A	exonic	CDKN1B		nonsynonymous SNV	CDKN1B:NM_004064:exon1:c.G419A:p.S1	NOVEL		370	0	0	1	387	2	0.0111751	1	198	18	0.198618	1	171	2	0.0288003	1
14	chr3	12645718	12645718	G	C	exonic	RAF1		nonsynonymous SNV	RAF1:NM_001354695:exon5:c.C409G:p.L1	NOVEL		202	0	0	1	222	0	0	1	115	20	0.353099	1	88	1	0.0279913	1
14	chr16	2002973	2002973	C	G	exonic	RPL3L		synonymous SNV	RPL3L:NM_005061:exon3:c.G267C:p.V89V	DBSNP		242	0	0	1	220	0	0	1	140	19	0.284811	1	99	1	0.0249122	1
14	chr16	75513359	75513359	C	G	exonic	CHST6		nonsynonymous SNV	CHST6:NM_021615:exon3:c.G368C:p.W12	NOVEL		511	0	0	1	500	0	0	1	412	56	0.285196	1	225	2	0.0219491	1
14	chr5	149630279	149630279	G	C	exonic	CAMK2A		nonsynonymous SNV	CAMK2A:NM_001369025:exon10:c.C788G	NOVEL		432	0	0	1	436	0	0	1	303	28	0.286211	1	172	1	0.0201805	1
14	chr7	56147344	56147344	C	T	exonic	SUMF2		nonsynonymous SNV	SUMF2:NM_001130069:exon8:c.C800T:p.S	NOVEL		614	0	0	1	600	0	0	1	398	14	0.114971	1	219	1	0.0158692	1
14	chr17	78112059	78112059	C	T	exonic	EIF4A3		nonsynonymous SNV	EIF4A3:NM_014740:exon8:c.G749A:p.G25	NOVEL		82	0	0	1	84	0	0	1	93	15	0.331031	1	48	0	0	1
14	chr8	39114731	39114731	G	C	exonic	ADAM32		synonymous SNV	ADAM32:NM_001313994:exon16:c.G1734	NOVEL		279	1	0.00820582	1	278	0	0	1	237	31	0.275694	1	93	0	0	1
14	chr7	127014527	127014527	C	A	exonic	ZNF800		nonsynonymous SNV	ZNF800:NM_176814:exon5:c.G863T:p.S28	NOVEL		636	0	0	1	722	0	0	1	382	33	0.269043	1	185	0	0	1
14	chr21	34635178	34635178	C	A	exonic	IFNAR2		synonymous SNV	IFNAR2:NM_001289125:exon9:c.C921A:p.	DBSNP		299	1	0.00432543	1	340	0	0	1	143	32	0.252968	1	88	0	0	1
14	chr5	139267075	139267075	T	G	exonic	NRG2		nonsynonymous SNV	NRG2:NM_001184935:exon2:c.A722C:p.K	NOVEL		375	0	0	1	394	0	0	1	194	8	0.133997	1	128	0	0	1
14	chr11	65340978	65340978	C	G	exonic	FAM89B		nonsynonymous SNV	FAM89B:NM_001098784:exon2:c.C446G:p	NOVEL		193	18	0.196006	1	201	15	0.150941	1	197	0	0	1	149	0	0	1
14	chr9	115811706	115811706	C	A	exonic	ZFP37		synonymous SNV	ZFP37:NM_001282515:exon3:c.G330T:p.G	NOVEL		1019	28	0.113776	1	1110	27	0.0980754	1	932	0	0	1	417	0	0	1
14	chrX	148059462	148059462	G	C	splicing	AFF2	NM_001169122:exon1			NOVEL		152	128	1.05034	1	169	139	0.98092	1	132	71	0.83361	1	74	55	1.06215	1
14	chr15	33893754	33893754	G	T	splicing	RYR3	NM_001243996:exon1			NOVEL		220	0	0	1	258	0	0	1	140	10	0.158895	1	164	2	0.0300147	1
14	chrX	48935301	48935301	C	T	splicing	WDR45	NM_007075:exon5:c.2			NOVEL		257	0	0	1	225	0	0	1	109	33	0.276947	1	88	2	0.0276803	1
15	chr1	63788817	63788817	G	A	exonic	FOXD3		nonsynonymous SNV	FOXD3:NM_012183:exon1:c.G88A:p.E30K	COSMIC	ID=COSM426565	117	65	NA	1	158	86	NA	1	190	77	NA	1				
15	chrX	3248115	3248115	G	A	exonic	MXRA5		nonsynonymous SNV	MXRA5:NM_015419:exon4:c.C653T:p.P218	COSMIC	ID=COSM671839	0	54	NA	1	5	68	NA	1	3	51	NA	1				
15	chrX	78427050	78427050	C	G	exonic	GPR174		synonymous SNV	GPR174:NM_032553:exon1:c.C546G:p.S18	NOVEL		7	277	NA	1	31	303	NA	1	8	263	NA	1				
15	chrX	135055821	135055821	A	T	exonic	MMGT1		nonsynonymous SNV	MMGT1:NM_173470:exon1:c.T14A:p.L5Q	NOVEL		172	123	NA	1	190	161	NA	1	170	129	NA	1				
15	chrX	141291066	141291066	C	T	exonic	MAGEC2		synonymous SNV	MAGEC2:NM_016249:exon3:c.G708A:p.V2	NOVEL		209	0	NA	1	278	0	NA	1	186	21	NA	1				
15	chr1	74937872	74937872	G	A	exonic	LRRC53		synonymous SNV	LRRC53:NM_001364665:exon4:c.C1434T:p	NOVEL		138	120	1.44468	1	185	168	0.762777	2	150	128	1.45813	1				
15	chr9	18680331	18680331	C	T	exonic	ADAMTSL1		synonymous SNV	ADAMTSL1:NM_001040272:exon11:c.C11	DBSNP		96	53	1.10484	1	132	76	1.17048	1	120	71	1.25878	1				
15	chr1	247886796	247886796	T	G	exonic	OR14A2		synonymous SNV	OR14A2:NM_001355292:exon5:c.A550C:p	NOVEL		116	107	1.01053	1	206	147	0.918429	1	165	166	1.24515	1				
15	chr6	116442162	116442162	G	A	exonic	COL10A1		nonsynonymous SNV	COL10A1:NM_000493:exon3:c.C1117T:p.P	NOVEL		106	131	1.16411	1	173	164	1.07329	1	114	133	1.16678	1				
15	chr9	138376757	138376757	A	G	exonic	PPP1R26		nonsynonymous SNV	PPP1R26:NM_014811:exon4:c.A401G:p.E1	NOVEL		30	11	0.833333	1	31	14	0.996623	1	28	16	1.15159	1				
15	chr12	49435719	49435719	G	C	exonic	KMT2D		nonsynonymous SNV	KMT2D:NM_003482:exon29:c.C6164G:p.A	NOVEL		117	115	1.04395	1	149	101	0.891014	1	120	132	1.13503	1				
15	chr9	133271644	133271644	C	T	exonic	HMCN2		unknown	UNKNOWN	NOVEL		182	84	0.980861	1	238	90	0.87899	1	169	94	1.13189	1				
15	chr5	163329	163329	G	A	exonic	PLEKHG4B		nonsynonymous SNV	PLEKHG4B:NM_052909:exon13:c.G3142A:	NOVEL		187	74	0.880645	1	219	112	1.08463	1	175	94	1.10664	1				
15	chr3	8590329	8590329	C	T	exonic	LMCD1		nonsynonymous SNV	LMCD1:NM_001278233:exon3:c.C244T:p.R	DBSNP+COSMIC	ID=COSM282788	258	120	0.986051	1	319	181	1.15964	1	351	183	1.08528	1				
15	chr2	223160284	223160284	G	C	exonic	PAX3		synonymous SNV	PAX3:NM_000438:exon3:c.C414G:p.L138L	NOVEL		92	84	1.00516	1	127	76	0.825697	1	99	98	1.07794	1				
15	chr2	241439879	241439879	C	G	exonic	ANKMY1		nonsynonymous SNV	ANKMY1:NM_001282780:exon10:c.G1700	NOVEL		139	145	1.07528	1	184	122	0.879309	1	165	163	1.07683	1				
15	chr11	76795980	76795980	G	A	exonic	CAPN5		synonymous SNV	CAPN5:NM_004055:exon2:c.G48A:p.L16L	COSMIC	ID=COSM718029	347	175	1.0413	1	420	220	1.10118	1	352	178	1.06359	1				
15	chr1	153740211	153740211	G	A	exonic	INTS3		nonsynonymous SNV	INTS3:NM_023015:exon21:c.G2152A:p.D7	COSMIC	ID=COSM668642	152	144	1.02457	1	184	155	1.00841	1	248	184	1.05749	1				
15	chr5	36035970	36035970	C	T	exonic	UGT3A2		nonsynonymous SNV	UGT3A2:NM_001168316:exon6:c.G1300A	DBSNP+COSMIC	ID=COSM76948;	214	104	1.01582	1	266	121	1.00159	1	304	152	1.05562	1				
15	chr8	19221709	19221709	G	T	exonic	SH2D4A		nonsynonymous SNV	SH2D4A:NM_001174160:exon6:c.G698T:p	NOVEL		81	68	0.961155	1	103	81	0.97089	1	73	69	1.05292	1				
15	chr3	169710616	169710616	A	G	exonic	SEC62		nonsynonymous SNV	SEC62:NM_003262:exon8:c.A965G:p.E322	NOVEL		373	174	0.988033	1	458	225	1.0553	1	444	220	1.04926	1				
15	chr18	3729258	3729258	G	A	exonic	DLGAP1		nonsynonymous SNV	DLGAP1:NM_001003809:exon4:c.C562T:p.	DBSNP		127	101	0.932947	1	176	135	0.957363	1	146	137	1.04898	1				
15	chr2	11053695	11053695	G	A	exonic	KCNF1		synonymous SNV	KCNF1:NM_002236:exon1:c.G1143A:p.T38	DBSNP+COSMIC	ID=COSM729050	146	140	1.03094	1	181	175	1.08415	1	183	169	1.04034	1				
15	chr2	141200085	141200085	C	T	exonic	LRP1B		nonsynonymous SNV	LRP1B:NM_018557:exon66:c.G10402A:p.E	NOVEL		161	147	1.00516	1	264	175	0.879178	1	127	117	1.03903	1				
15	chr3	186504986	186504986	A	T	exonic	EIF4A2		nonsynonymous SNV	EIF4A2:NM_001967:exon8:c.A842T:p.N28	NOVEL		41	65	0.952329	2	64	104	0.991538	2	64	122	1.0386	2				
15	chr6	33635658	33635658	C	T	exonic	ITPR3		synonymous SNV	ITPR3:NM_002224:exon16:c.C1803T:p.N6	NOVEL		148	125	0.964313	1	169	125	0.937704	1	181	163	1.02674	1				
15	chr19	18119536	18119536	G	A	exonic	ARRDC2		synonymous SNV	ARRDC2:NM_001025604:exon2:c.G276A:p	COSMIC	ID=COSM148078	286	108	0.851407	1	313	138	0.980207	1	242	115	1.02014	1				
15	chr6	110300453	110300453	C	T	exonic	GPR6		synonymous SNV	GPR6:NM_005284:exon2:c.C138T:p.A46A	NOVEL		91	85	1.01713	1	125	99	0.974743	1	96	85	1.01759	1				
15	chr12	1017135	1017135	A	T	exonic	WNK1		nonsynonymous SNV	WNK1:NM_014823:exon25:c.A6022T:p.N2	NOVEL		39	47	1.15099	1	76	57	0.945206	1	51	45	1.01572	1				
15	chr1	205814641	205814641	C	T	exonic	PM20D1		nonsynonymous SNV	PM20D1:NM_152491:exon3:c.G301A:p.V1	DBSNP+COSMIC	ID=COSM902844	110	121	1.10317	1	179	137	0.956173	1	136	94	1.01471	1				
15	chr3	73111590	73111590	C	T	exonic	EBLN2		nonsynonymous SNV	EBLN2:NM_018029:exon1:c.C358T:p.H120	NOVEL		634	290	0.974845	1	800	319	0.913221	1	713	336	1.01437	1				
15	chr11	5424353	5424353	G	A	exonic	OR51J1		nonsynonymous SNV	OR51J1:NM_001348224:exon1:c.G527A:p	NOVEL		495	248	1.03675	1	664	264	0.91132	1	572	268	1.01038	1				
15	chr12	6161871	6161871	G	A	exonic	VWF		nonsynonymous SNV	VWF:NM_000552:exon16:c.C2024T:p.P67	DBSNP		127	135	1.08518	1	212	150	0.913873	1	132	111	0.989806	1				
15	chr17	43227130	43227130	C	G	exonic	HEXIM1		synonymous SNV	HEXIM1:NM_006460:exon1:c.C573G:p.A19	NOVEL		280	295	1.0805	1	424	308	0.927989	1	325	272	0.987252	1				
15	chr6	99893928	99893928	A	G	exonic	USP45		nonsynonymous SNV	USP45:NM_001346024:exon12:c.T1546C:p	NOVEL		169	129	0.911683	1	242	165	0.894113	1	215	178	0.981433	1				
15	chr6	1611324	1611324	G	T	exonic	FOXC1		nonsynonymous SNV	FOXC1:NM_001453:exon1:c.G644T:p.G21	NOVEL		31	48	0.943613	2	58	57	0.793893	2	35	57	0.981042	2				
15	chr5	126993362	126993362	G	A	exonic	CTXN3		nonsynonymous SNV	CTXN3:NM_001048252:exon3:c.G149A:p.R	DBSNP+COSMIC	ID=COSM663796	450	187	0.911826	1	530	228	0.963565	1	319	143	0.980222	1				
15	chr6	28097311	28097311	T	C	exonic	ZSCAN16		synonymous SNV	ZSCAN16:NM_001320555:exon4:c.T630C:	NOVEL		808	243	1.17834	1	924	264	0.85929	1	767	235	0.97726	1				
15	chr20	62830248	62830248	A	G	exonic	MYT1		nonsynonymous SNV	MYT1:NM_004535:exon3:c.A34G:p.T12A	NOVEL		162	127	0.9255	1	215	162	0.947713	1	146	117	0.96397	1				
15	chr9	116859931	116859931	G	A	exonic	KIF12		nonsynonymous SNV	KIF12:NM_138424:exon1:c.C52T:p.P18S	DBSNP		147	83	1.12088	1	176	82	1.01814	1	242	105	0.958275	1				
15	chr16	22126775	22126775	T	A	exonic	VWA3A		nonsynonymous SNV	VWA3A:NM_173615:exon9:c.T797A:p.V26	DBSNP		49	41	0.959427	1	77	59	0.956789	1	143	67	0.955125	1				
15	chr3	77638049	77638049	G	A	exonic	ROBO2		nonsynonymous SNV	ROBO2:NM_002942:exon17:c.G2648A:p.R	COSMIC	ID=COSM282702	197	357	1.00078	2	283	398	0.936098	2	251	365	0.938237	2				
15	chr3	187442784	187442784	T	G	exonic	BCL6		nonsynonymous SNV	BCL6:NM_001134738:exon7:c.A1754C:p.H	NOVEL		65	124	1.01892	2	86	161	1.04403	2	98	138	0.925908	2				
15	chr10	21806675	21806675	A	T	exonic	SKIDA1		nonsynonymous SNV	SKIDA1:NM_207371:exon4:c.T77A:p.M26K	NOVEL		100	113	1.1173	1	185	113	0.836306	1	177	132	0.925654	1				
15	chr5	96503481	96503481	C	G	exonic	RIOK2		nonsynonymous SNV	RIOK2:NM_001159749:exon8:c.G1087C:p.	NOVEL		715	318	0.956173	1	888	390	0.977572	1	562	232	0.925333	1				
15	chr17	19684331	19684331	C	G	exonic	ULK2		nonsynonymous SNV	ULK2:NM_001142610:exon24:c.G2726C:p.	NOVEL		42	21	0.70202	1	47	33	0.90976	1	47	35	0.924885	1				
15	chr12	41966665	41966665	A	T	exonic	PDZRN4		nonsynonymous SNV	PDZRN4:NM_013377:exon8:c.A1310T:p.Q	NOVEL		117	136	1.13211	1	212	173	0.991034	1	175	124	0.898636	1				
15	chr5	31511162	31511162	G	A	exonic	DROSHA		nonsynonymous SNV	DROSHA:NM_001100412:exon8:c.C1301T:	DBSNP+COSMIC	ID=COSM280612	204	100	1.02173	1	270	134	1.06252	1	231	89	0.880786	1				
15	chr9	126777429	126777429	T	G	exonic	LHX2		nonsynonymous SNV	LHX2:NM_004789:exon3:c.T352G:p.C118G	NOVEL		336	147	0.945322	1	402	182	0.998329	1	348	134	0.880417	1				
15	chr11	377076	377076	C	T	exonic	B4GALNT4		synonymous SNV	B4GALNT4:NM_178537:exon14:c.C1953T:	NOVEL		98	0	0	1	107	0	0	1	142	46	0.774873	1				
15	chr3	58139348	58139348	G	A	exonic	FLNB		nonsynonymous SNV	FLNB:NM_001164319:exon38:c.G6542A:p	NOVEL		234	1	0.0132173	1	305	1	0.0104687	1	185	57	0.745916	1				
15	chr4	78527031	78527031	C	T	exonic	CXCL13		synonymous SNV	CXCL13:NM_006419:exon2:c.C12T:p.I4I	NOVEL		258	3	0.0242076	1	344	4	0.0253503	1	144	70	0.70879	1				
15	chr19	13248118	13248118	T	C	exonic	NACC1		nonsynonymous SNV	NACC1:NM_052876:exon4:c.T1154C:p.M3	NOVEL		347	2	0.0177998	1	438	5	0.0361561	1	278	80	0.707681	1				
15	chr14	96795011	96795011	C	T	exonic	ATG2B		synonymous SNV	ATG2B:NM_018036:exon13:c.G1974A:p.Q	NOVEL		222	4	0.0372754	1	355	8	0.0486056	1	177	85	0.702993	1				
15	chr19	43772025	43772025	C	T	exonic	PSG9		nonsynonymous SNV	PSG9:NM_001301707:exon2:c.G341A:p.R1	DBSNP+COSMIC	ID=COSM675758	414	2	0.014933	1	464	3	0.0205788	1	601	170	0.698273	1				
15	chr2	217279609	217279609	C	A	exonic	SMARCAL1		nonsynonymous SNV	SMARCAL1:NM_001127207:exon3:c.C182	NOVEL		412	9	0.0450226	1	526	11	0.0451774	1	268	125	0.689209	1				
15	chr12	25398281	25398281	C	T	exonic	KRAS		nonsynonymous SNV	KRAS:NM_001369786:exon2:c.G38A:p.G1	DBSNP+COSMIC	ID=COSM114013	138	1	0.0151515	1	196	0	0	1	177	79	0.668683	1				
15	chr21	47706933	47706933	G	T	exonic	YBEY		nonsynonymous SNV	YBEY:NM_001006114:exon2:c.G106T:p.D3	NOVEL		315	4	0.0264083	1	382	6	0.0341054	1	302	131	0.655566	1				
15	chr11	56230173	56230173	C	G	exonic	OR5M9		synonymous SNV	OR5M9:NM_001004743:exon1:c.G705C:p.	NOVEL		249	3	0.0369769	1	362	1	0.00882487	1	249	65	0.655563	1				
15	chr14	100141927	100141927	G	T	exonic	HHIPL1		synonymous SNV	HHIPL1:NM_001127258:exon9:c.G2313T:p	NOVEL		153	2	0.027175	1	228	3	0.0286426	1	116	49	0.643495	1				
15	chr17	35603836	35603836	G	A	exonic	ACACA		nonsynonymous SNV	ACACA:NM_198837:exon17:c.C2081T:p.S6	NOVEL		259	2	0.0161384	1	320	2	0.0136986	1	245	101	0.632526	1				
15	chr3	112251600	112251600	T	C	exonic	ATG3		synonymous SNV	ATG3:NM_022488:exon12:c.A891G:p.V29	NOVEL		236	1	0.0131057	1	299	0	0	1	135	33	0.622064	1				
15	chr14	64675565	64675565	T	C	exonic	SYNE2		synonymous SNV	SYNE2:NM_015180:exon101:c.T18291C:p.	DBSNP		195	3	0.03191	1	258	3	0.0253503	1	171	49	0.482622	1				
15	chr16	52473244	52473244	A	G	exonic	TOX3		nonsynonymous SNV	TOX3:NM_001080430:exon7:c.T1624C:p.S	NOVEL		140	109	0.92193	1	167	141	1.00965	1	246	66	0.458377	1				
15	chr15	28386718	28386718	G	A	exonic	HERC2		nonsynonymous SNV	HERC2:NM_004667:exon78:c.C11875T:p.H	NOVEL		445	196	0.949747	1	568	261	1.00856	1	580	74	0.358331	1				
15	chr4	153897552	153897552	C	T	exonic	FHDC1		nonsynonymous SNV	FHDC1:NM_033393:exon11:c.C3109T:p.R1	NOVEL		286	236	0.952165	1	363	298	0.994301	1	355	66	0.3397	1				
15	chr9	123924365	123924365	T	C	exonic	CNTRL		nonsynonymous SNV	CNTRL:NM_001330762:exon23:c.T3583C:	DBSNP		478	219	0.975935	1	663	242	0.856608	1	765	86	0.320036	1				
15	chr1	115256530	115256530	G	T	exonic	NRAS		nonsynonymous SNV	NRAS:NM_002524:exon3:c.C181A:p.Q61K	DBSNP+COSMIC	ID=COSM580;CO	12	108	0.995453	1	36	143	0.966764	1	125	42	0.293465	1				
15	chr17	72436773	72436773	C	A	exonic	GPRC5C		synonymous SNV	GPRC5C:NM_001366261:exon2:c.C858A:p.	NOVEL		141	111	0.927669	1	145	121	1.00324	1	236	36	0.286792	1				
15	chr17	76672239	76672239	G	A	exonic	CYTH1		synonymous SNV	CYTH1:NM_001292018:exon13:c.C954T:p.	NOVEL		84	67	0.934477	1	101	91	1.04531	1	176	26	0.278904	1				
15	chr14	58563571	58563571	G	A	exonic	ARMH4		nonsynonymous SNV	ARMH4:NM_001001872:exon5:c.C1960T:p	NOVEL		170	1	0.0123161	1	139	90	0.866782	1	187	27	0.27339	1				
15	chr10	91143193	91143193	A	T	exonic	IFIT1B		nonsynonymous SNV	IFIT1B:NM_001010987:exon2:c.A123T:p.E	NOVEL		328	0	0	1	248	203	0.99271	1	403	57	0.268504	1				
15	chr19	53572126	53572126	C	A	exonic	ZNF160		nonsynonymous SNV	ZNF160:NM_001322136:exon5:c.G1661T:	NOVEL		447	0	0	1	605	1	0.00525639	1	739	68	0.266849	1				
15	chr21	31587762	31587762	A	T	exonic	CLDN8		nonsynonymous SNV	CLDN8:NM_199328:exon1:c.T482A:p.L161	NOVEL		246	0	0	1	179	126	0.911116	1	252	32	0.244155	1				
15	chr7	123101633	123101633	A	G	exonic	IQUB		synonymous SNV	IQUB:NM_001282855:exon11:c.T1785C:p.	NOVEL		345	0	0	1	340	148	0.971532	1	283	30	0.236826	1				
15	chr7	114271689	114271689	G	A	exonic	FOXP2		nonsynonymous SNV	FOXP2:NM_148899:exon5:c.G704A:p.S235	COSMIC	ID=COSM281220	194	1	0.0157514	1	199	89	0.989949	1	171	18	0.235322	1				
15	chr4	122254169	122254169	A	G	exonic	QRFPR		synonymous SNV	QRFPR:NM_198179:exon4:c.T604C:p.L202	NOVEL		187	0	0	1	191	118	0.842222	1	222	26	0.227172	1				
15	chr8	95502308	95502308	T	A	exonic	VIRMA		nonsynonymous SNV	VIRMA:NM_015496:exon23:c.A5143T:p.N	NOVEL		225	0	0	1	170	140	0.996023	1	301	35	0.225716	1				
15	chr11	17436869	17436869	G	A	exonic	ABCC8		nonsynonymous SNV	ABCC8:NM_000352:exon18:c.C2273T:p.A7	DBSNP		123	51	0.910397	1	129	51	0.907638	1	249	18	0.213497	1				
15	chr10	126662954	126662954	G	A	exonic	ZRANB1		nonsynonymous SNV	ZRANB1:NM_017580:exon5:c.G1414A:p.D	DBSNP		206	0	0	1	135	116	1.01927	1	183	20	0.213485	1				
15	chr15	63084975	63084975	G	A	exonic	TLN2		nonsynonymous SNV	TLN2:NM_015059:exon43:c.G5872A:p.V19	DBSNP		157	0	0	1	187	0	0	1	159	11	0.204915	1				
15	chr2	55470671	55470671	C	T	exonic	MTIF2		nonsynonymous SNV	MTIF2:NM_001321004:exon11:c.G1445A:	NOVEL		449	0	0	1	323	222	0.898379	1	260	27	0.203852	1				
15	chr10	12214780	12214780	C	T	exonic	NUDT5		synonymous SNV	NUDT5:NM_001321647:exon7:c.G471A:p.	NOVEL		333	0	0	1	210	167	0.976963	1	319	32	0.19755	1				
15	chr22	29957532	29957532	C	T	exonic	NIPSNAP1		nonsynonymous SNV	NIPSNAP1:NM_001202502:exon6:c.G482A	DBSNP		141	0	0	1	95	84	1.03497	1	214	21	0.193635	1				
15	chr9	21350673	21350673	G	A	exonic	IFNA6		stopgain	IFNA6:NM_021002:exon1:c.C214T:p.Q72X	NOVEL		366	0	0	1	353	133	0.876659	1	470	28	0.190395	1				
15	chr17	40327691	40327691	C	T	exonic	KCNH4		nonsynonymous SNV	KCNH4:NM_012285:exon6:c.G893A:p.R29	DBSNP+COSMIC	ID=COSM386817	177	0	0	1	228	0	0	1	187	18	0.190262	1				
15	chr16	90029815	90029815	C	A	exonic	DEF8		synonymous SNV	DEF8:NM_001242817:exon9:c.C786A:p.T2	NOVEL		101	1	0.0206476	1	52	57	1.15332	1	118	11	0.184772	1				
15	chr4	62598641	62598641	G	A	exonic	ADGRL3		synonymous SNV	ADGRL3:NM_001371342:exon5:c.G549A:p	DBSNP		421	0	0	1	344	213	0.843388	1	365	34	0.184646	1				
15	chr17	37618601	37618601	C	T	exonic	CDK12		stopgain	CDK12:NM_015083:exon1:c.C277T:p.R93X	COSMIC	ID=COSM351677	254	0	0	1	215	143	0.88096	1	286	25	0.174186	1				
15	chr1	150390131	150390131	A	C	exonic	RPRD2		nonsynonymous SNV	RPRD2:NM_001297673:exon2:c.A265C:p.K	NOVEL		469	0	0	1	572	0	0	1	449	33	0.169984	1				
15	chr19	38572326	38572326	G	A	exonic	SIPA1L3		nonsynonymous SNV	SIPA1L3:NM_015073:exon3:c.G121A:p.A4	NOVEL		169	0	0	1	203	0	0	1	165	9	0.163804	1				
15	chr18	54281827	54281827	C	T	exonic	TXNL1		nonsynonymous SNV	TXNL1:NM_004786:exon6:c.G563A:p.G188	NOVEL		169	0	0	1	127	84	0.878011	1	182	14	0.154777	1				
15	chr19	58948499	58948499	T	G	exonic	ZNF132		nonsynonymous SNV	ZNF132:NM_003433:exon2:c.A147C:p.Q4	NOVEL		477	0	0	1	355	177	1.06648	1	396	19	0.144989	1				
15	chr14	78205212	78205212	T	C	exonic	SNW1		nonsynonymous SNV	SNW1:NM_001318844:exon5:c.A442G:p.R	NOVEL		276	0	0	1	345	0	0	1	199	14	0.142424	1				
15	chr7	94048824	94048824	T	C	exonic	COL1A2		synonymous SNV	COL1A2:NM_000089:exon34:c.T2040C:p.A	NOVEL		394	0	0	1	532	2	0.0119979	1	326	17	0.122463	1				
15	chr19	42824506	42824506	T	C	exonic	TMEM145		nonsynonymous SNV	TMEM145:NM_001366910:exon13:c.T115	NOVEL		327	0	0	1	374	0	0	1	326	13	0.121443	1				
15	chr14	51224865	51224865	C	T	exonic	NIN		synonymous SNV	NIN:NM_020921:exon18:c.G2883A:p.S961	DBSNP		222	0	0	1	286	1	0.0076846	1	238	14	0.120382	1				
15	chr2	112995991	112995991	C	A	exonic	ZC3H8		nonsynonymous SNV	ZC3H8:NM_032494:exon3:c.G271T:p.A91S	NOVEL		239	0	0	1	295	0	0	1	257	15	0.119497	1				
15	chr11	13380135	13380135	A	C	exonic	ARNTL		synonymous SNV	ARNTL:NM_001351805:exon6:c.A337C:p.R	NOVEL		537	0	0	1	656	0	0	1	480	17	0.108324	1				
15	chr19	10817981	10817981	C	T	exonic	QTRT1		nonsynonymous SNV	QTRT1:NM_031209:exon4:c.C455T:p.S152	NOVEL		505	0	0	1	636	1	0.00502893	1	819	27	0.10107	1				
15	chr15	41366763	41366763	C	T	exonic	INO80		nonsynonymous SNV	INO80:NM_017553:exon10:c.G1132A:p.A	NOVEL		378	0	0	1	433	1	0.00738117	1	461	14	0.0933394	1				
15	chr11	47505946	47505946	C	T	exonic	CELF1		nonsynonymous SNV	CELF1:NM_001025596:exon4:c.G440A:p.S	NOVEL		572	2	0.0108225	1	658	2	0.00970736	1	641	18	0.0865003	1				
15	chr4	56734645	56734645	G	A	exonic	EXOC1		nonsynonymous SNV	EXOC1:NM_001024924:exon5:c.G559A:p.A	COSMIC	ID=COSM105618	516	0	0	1	681	1	0.00323384	1	390	15	0.0802545	1				
15	chr22	24459520	24459520	G	A	exonic	CABIN1		nonsynonymous SNV	CABIN1:NM_001201429:exon13:c.G1645A	NOVEL		234	0	0	1	239	0	0	1	265	10	0.0787954	1				
15	chr1	236758908	236758908	T	A	exonic	HEATR1		nonsynonymous SNV	HEATR1:NM_018072:exon8:c.A1022T:p.Y3	NOVEL		355	0	0	1	479	0	0	1	277	9	0.0781301	1				
15	chr16	84205876	84205876	C	G	exonic	DNAAF1		synonymous SNV	DNAAF1:NM_001318756:exon5:c.C831G:p	NOVEL		257	0	0	1	303	0	0	1	302	11	0.0761521	1				
15	chr4	141870489	141870489	T	A	exonic	RNF150		nonsynonymous SNV	RNF150:NM_020724:exon3:c.A773T:p.K25	NOVEL		371	0	0	1	527	0	0	1	474	13	0.0578426	1				
15	chr13	114287535	114287535	G	A	exonic	TFDP1		nonsynonymous SNV	TFDP1:NM_007111:exon6:c.G409A:p.E137	COSMIC	ID=COSM945628	45	21	0.67011	1	95	0	0	1	111	1	0.0193471	1				
15	chr16	30005661	30005661	A	G	exonic	HIRIP3		nonsynonymous SNV	HIRIP3:NM_003609:exon4:c.T805C:p.S269	NOVEL		288	79	0.453348	1	464	0	0	1	565	2	0.0105597	1				
15	chr1	208383651	208383651	C	A	exonic	PLXNA2		nonsynonymous SNV	PLXNA2:NM_025179:exon3:c.G1345T:p.G	NOVEL		164	41	0.421212	1	259	0	0	1	408	1	0.00607042	1				
15	chr6	35213029	35213029	G	A	exonic	SCUBE3		nonsynonymous SNV	SCUBE3:NM_001303136:exon19:c.G2423A	NOVEL		265	73	0.454859	1	397	0	0	1	359	1	0.00601909	1				
15	chr2	84915653	84915653	A	G	exonic	DNAH6		nonsynonymous SNV	DNAH6:NM_001370:exon45:c.A7228G:p.N	NOVEL		103	48	0.669476	1	203	0	0	1	167	0	0	1				
15	chr22	18389476	18389476	G	A	exonic	MICAL3		nonsynonymous SNV	MICAL3:NM_001122731:exon1:c.C103T:p.	NOVEL		125	18	0.265098	1	159	0	0	1	164	0	0	1				
15	chr6	168265391	168265391	C	G	exonic	AFDN		nonsynonymous SNV	AFDN:NM_001040000:exon2:c.C266G:p.P	NOVEL		440	60	0.252727	1	687	0	0	1	547	0	0	1				
15	chr3	150387081	150387081	A	C	splicing	ERICH6	NM_001308234:exon1			NOVEL		199	74	0.841935	1	272	100	0.861137	1	170	73	0.951365	1				
15	chr3	160956616	160956616	T	A	splicing	NMD3	NM_015938:exon9:c.7			NOVEL		108	1	0.028496	1	144	0	0	1	99	32	0.773587	1				
15	chr1	37962339	37962339	T	C	splicing	MEAF6	NM_022756:exon6:c.5			NOVEL		121	0	0	1	76	56	0.935658	1	118	9	0.153558	1				
17	chrX	19449651	19449651	G	C	exonic	MAP3K15		synonymous SNV	MAP3K15:NM_001001671:exon7:c.C1071	NOVEL		107	10	NA	1	104	21	NA	1								
17	chr12	88477666	88477666	T	C	exonic	CEP290		synonymous SNV	CEP290:NM_025114:exon36:c.A4770G:p.L	NOVEL		123	96	1.18416	1	150	136	1.49716	1								
17	chr4	96140436	96140436	C	T	exonic	UNC5C		synonymous SNV	UNC5C:NM_003728:exon9:c.G1329A:p.T4	DBSNP		133	92	1.10456	1	139	126	1.497	1								
17	chr11	17531313	17531313	C	T	exonic	USH1C		nonsynonymous SNV	USH1C:NM_153676:exon18:c.G1603A:p.G	DBSNP+COSMIC	ID=COSM558696	116	108	1.30244	1	117	106	1.49657	1								
17	chr12	16035693	16035693	G	T	exonic	STRAP		nonsynonymous SNV	STRAP:NM_007178:exon1:c.G52T:p.V18F	NOVEL		250	213	1.24274	1	282	253	1.48889	1								
17	chr13	60413558	60413558	T	A	exonic	DIAPH3		nonsynonymous SNV	DIAPH3:NM_030932:exon17:c.A1973T:p.N	COSMIC	ID=COSM502778	73	162	1.26861	1	145	237	1.46097	1								
17	chr2	74709331	74709331	A	G	exonic	CCDC142		nonsynonymous SNV	CCDC142:NM_001365575:exon1:c.T634C:p	NOVEL		208	168	1.20699	1	237	198	1.43309	1								
17	chr16	4935839	4935839	C	G	exonic	PPL		nonsynonymous SNV	PPL:NM_002705:exon22:c.G2817C:p.K939	NOVEL		212	206	1.33129	1	243	201	1.42531	1								
17	chr8	35583900	35583900	C	T	exonic	UNC5D		nonsynonymous SNV	UNC5D:NM_001322818:exon10:c.C1519T:	NOVEL		250	195	1.18374	1	310	255	1.42098	1								
17	chr19	45991918	45991918	T	C	exonic	RTN2		nonsynonymous SNV	RTN2:NM_206901:exon4:c.A407G:p.N136	NOVEL		232	198	1.24388	1	259	213	1.4208	1								
17	chr5	35654708	35654708	T	A	exonic	SPEF2		nonsynonymous SNV	SPEF2:NM_024867:exon7:c.T858A:p.D286	NOVEL		323	300	1.30082	1	429	352	1.41902	1								
17	chr6	28402772	28402772	C	T	exonic	ZSCAN23		nonsynonymous SNV	ZSCAN23:NM_001012455:exon4:c.G640A:	DBSNP		149	106	1.12292	1	165	135	1.4168	1								
17	chr10	91066408	91066408	G	A	exonic	IFIT2		nonsynonymous SNV	IFIT2:NM_001547:exon2:c.G695A:p.G232	NOVEL		231	201	1.25688	1	290	236	1.41261	1								
17	chr14	60433388	60433388	A	C	exonic	LRRC9		synonymous SNV	LRRC9:NM_001355272:exon11:c.A1293C:p	NOVEL		75	54	1.1308	1	83	67	1.40631	1								
17	chr2	21233746	21233746	A	C	exonic	APOB		synonymous SNV	APOB:NM_000384:exon26:c.T5994G:p.A1	NOVEL		321	285	1.27044	1	414	332	1.40118	1								
17	chr20	36869665	36869665	G	A	exonic	KIAA1755		nonsynonymous SNV	KIAA1755:NM_001029864:exon3:c.C868T:	DBSNP+COSMIC	ID=COSM458178	253	189	1.15511	1	294	228	1.37518	1								
17	chr1	20141507	20141507	G	T	exonic	RNF186		nonsynonymous SNV	RNF186:NM_019062:exon1:c.C88A:p.H30	NOVEL		139	121	1.25717	1	182	139	1.36335	1								
17	chr10	135193636	135193636	G	C	exonic	PAOX		synonymous SNV	PAOX:NM_152911:exon2:c.G315C:p.G105	NOVEL		85	76	1.27518	1	102	77	1.35436	1								
17	chr19	45150609	45150609	C	T	exonic	PVR		nonsynonymous SNV	PVR:NM_001135768:exon2:c.C194T:p.T65	NOVEL		160	141	1.26542	1	184	137	1.34373	1								
17	chr12	92539197	92539197	T	C	exonic	BTG1		nonsynonymous SNV	BTG1:NM_001731:exon1:c.A115G:p.T39A	NOVEL		79	52	1.0723	1	93	69	1.341	1								
17	chr11	63078495	63078495	G	A	exonic	SLC22A10		nonsynonymous SNV	SLC22A10:NM_001039752:exon10:c.G161	DBSNP		75	44	0.998823	1	91	67	1.3351	1								
17	chr1	20141504	20141504	A	G	exonic	RNF186		nonsynonymous SNV	RNF186:NM_019062:exon1:c.T91C:p.S31P	NOVEL		140	123	1.26337	1	189	137	1.32312	1								
17	chr7	21677275	21677275	C	T	exonic	DNAH11		nonsynonymous SNV	DNAH11:NM_001277115:exon27:c.C4772T	DBSNP+COSMIC	ID=COSM302542	281	255	1.28516	1	404	283	1.29696	1								
17	chr16	4487436	4487436	G	T	exonic	DNAJA3		nonsynonymous SNV	DNAJA3:NM_001135110:exon3:c.G379T:p	NOVEL		235	177	1.16054	1	290	198	1.27744	1								
17	chr15	62292792	62292792	T	A	exonic	VPS13C		nonsynonymous SNV	VPS13C:NM_017684:exon14:c.A1195T:p.I	NOVEL		109	68	1.03781	1	138	94	1.27566	1								
17	chr7	154746060	154746060	G	C	exonic	PAXIP1		nonsynonymous SNV	PAXIP1:NM_007349:exon16:c.C2726G:p.T	NOVEL		213	170	1.19904	1	288	195	1.27111	1								
17	chr5	96232519	96232519	C	T	exonic	ERAP2		synonymous SNV	ERAP2:NM_001329229:exon8:c.C1320T:p.	NOVEL		51	44	1.25116	1	60	40	1.25938	1								
17	chr11	58035054	58035054	C	G	exonic	OR10W1		nonsynonymous SNV	OR10W1:NM_207374:exon1:c.G277C:p.A	NOVEL		204	166	1.21196	1	284	189	1.25805	1								
17	chr12	92539281	92539281	T	C	exonic	BTG1		nonsynonymous SNV	BTG1:NM_001731:exon1:c.A31G:p.M11V	NOVEL		86	52	1.0179	1	107	69	1.23433	1								
17	chr20	60847229	60847229	T	A	exonic	OSBPL2		nonsynonymous SNV	OSBPL2:NM_001278649:exon5:c.T31A:p.F	NOVEL		201	141	1.11372	1	253	162	1.22903	1								
17	chr10	3186522	3186522	C	G	exonic	PITRM1		nonsynonymous SNV	PITRM1:NM_001242309:exon19:c.G2200C	NOVEL		221	122	0.960834	1	256	160	1.21094	1								
17	chr10	105763004	105763004	A	G	exonic	SLK		nonsynonymous SNV	SLK:NM_001304743:exon9:c.A2068G:p.I69	DBSNP		271	154	0.978847	1	325	195	1.18067	1								
17	chr6	43034110	43034110	C	T	exonic	KLC4		nonsynonymous SNV	KLC4:NM_001289035:exon4:c.C407T:p.T13	DBSNP		139	89	1.05448	1	154	92	1.17747	1								
17	chr10	115537233	115537233	C	T	exonic	PLEKHS1		nonsynonymous SNV	PLEKHS1:NM_182601:exon12:c.C1232T:p.A	NOVEL		312	189	1.01908	1	393	229	1.15915	1								
17	chr5	140222962	140222962	G	A	exonic	PCDHA8		nonsynonymous SNV	PCDHA8:NM_018911:exon1:c.G2056A:p.V	DBSNP		144	107	1.15158	1	197	114	1.15409	1								
17	chr2	182387052	182387052	T	G	exonic	ITGA4		nonsynonymous SNV	ITGA4:NM_000885:exon18:c.T2057G:p.I68	NOVEL		254	167	1.07156	1	330	188	1.14268	1								
17	chr5	147889369	147889369	C	T	exonic	HTR4		synonymous SNV	HTR4:NM_001040169:exon5:c.G726A:p.S2	DBSNP+COSMIC	ID=COSM106404	278	165	1.00615	1	359	201	1.13007	1								
17	chr12	25398284	25398284	C	A	exonic	KRAS		nonsynonymous SNV	KRAS:NM_001369786:exon2:c.G35T:p.G12	DBSNP+COSMIC	ID=COSM114013	125	72	0.9873	1	160	84	1.08389	1								
17	chr9	72064530	72064530	C	A	exonic	APBA1		synonymous SNV	APBA1:NM_001163:exon10:c.G2151T:p.L7	NOVEL		62	28	0.840424	1	67	35	1.08035	1								
17	chr16	52107708	52107708	A	G	exonic	C16orf97		nonsynonymous SNV	C16orf97:NM_001242473:exon1:c.T89C:p.	NOVEL		142	118	1.226	1	229	117	1.06465	1								
17	chr11	4825231	4825231	A	G	exonic	OR52R1		nonsynonymous SNV	OR52R1:NM_001005177:exon1:c.T380C:p.	NOVEL		192	100	0.925124	1	221	112	1.05894	1								
17	chr10	55582205	55582205	C	T	exonic	PCDH15		nonsynonymous SNV	PCDH15:NM_001142767:exon31:c.G5161A	NOVEL		98	65	1.07723	1	100	50	1.04948	1								
17	chr1	154116020	154116020	A	G	exonic	NUP210L		nonsynonymous SNV	NUP210L:NM_001159484:exon3:c.T374C:	NOVEL		321	232	1.1333	1	496	241	1.02955	1								
17	chr15	65621765	65621765	G	A	exonic	IGDCC3		nonsynonymous SNV	IGDCC3:NM_004884:exon13:c.C2168T:p.P	DBSNP+COSMIC	ID=COSM225643	123	50	1.01731	1	137	40	0.937502	1								
17	chr19	42482122	42482122	G	A	exonic	ATP1A3		nonsynonymous SNV	ATP1A3:NM_001256213:exon14:c.C1942T	COSMIC	ID=COSM660740	243	100	0.787569	1	270	110	0.911392	1								
17	chr17	7578525	7578525	G	T	exonic	TP53		stopgain	TP53:NM_001126115:exon1:c.C9A:p.C3X,T	COSMIC	ID=COSM44319,	21	78	1.34047	2	25	93	0.846633	2								
17	chr2	136033297	136033297	A	C	exonic	ZRANB3		nonsynonymous SNV	ZRANB3:NM_001286568:exon9:c.T995G:p	NOVEL		79	44	0.966341	1	117	43	0.846145	1								
17	chr6	154481007	154481007	G	C	exonic	IPCEF1		nonsynonymous SNV	IPCEF1:NM_001130700:exon12:c.C1273G:	NOVEL		18	81	1.39202	1	22	70	0.817343	2								
17	chr12	14923855	14923855	G	T	exonic	HIST4H4		nonsynonymous SNV	HIST4H4:NM_175054:exon1:c.C164A:p.T5	NOVEL		211	180	1.24359	1	253	244	0.772858	2								
17	chr15	22929881	22929881	C	T	exonic	CYFIP1		synonymous SNV	CYFIP1:NM_001324125:exon3:c.C189T:p.H	NOVEL		96	35	0.721738	1	130	42	0.768806	1								
17	chr17	8354180	8354180	T	C	exonic	NDEL1		synonymous SNV	NDEL1:NM_001025579:exon6:c.T609C:p.C	NOVEL		31	128	1.36965	1	44	110	0.767302	2								
17	chr17	40970680	40970680	C	G	exonic	BECN1		nonsynonymous SNV	BECN1:NM_001313999:exon5:c.G370C:p.D	NOVEL		25	106	1.37667	1	49	121	0.764594	2								
17	chr14	37737998	37737998	G	C	exonic	MIPOL1		nonsynonymous SNV	MIPOL1:NM_138731:exon6:c.G387C:p.K12	NOVEL		215	6	0.0733402	1	213	29	0.377293	1								
17	chr12	124399545	124399545	G	A	exonic	DNAH10		nonsynonymous SNV	DNAH10:NM_207437:exon61:c.G10367A:	DBSNP		106	4	0.0618677	1	127	21	0.304847	1								
17	chr6	117679037	117679037	A	C	exonic	ROS1		nonsynonymous SNV	ROS1:NM_002944:exon24:c.T3784G:p.Y12	NOVEL		74	5	0.107681	1	119	11	0.181792	1								
17	chr20	35879631	35879631	C	T	exonic	GHRH		synonymous SNV	GHRH:NM_001184731:exon4:c.G309A:p.R	COSMIC	ID=COSM539199	320	5	0.0415594	1	383	18	0.141327	1								
17	chr9	104357090	104357090	C	G	exonic	PPP3R2		synonymous SNV	PPP3R2:NM_147180:exon1:c.G114C:p.L38	NOVEL		545	8	0.0390794	1	576	22	0.115829	1								
18	chr2	242430591	242430591	C	T	exonic	FARP2		nonsynonymous SNV	FARP2:NM_014808:exon23:c.C2620T:p.P8	NOVEL		95	48	NA	1	99	86	NA	1	5	58	NA	1				
18	chrX	12632964	12632964	G	A	exonic	FRMPD4		nonsynonymous SNV	FRMPD4:NM_001368400:exon3:c.G266A:	COSMIC	ID=COSM443959	8	77	NA	1	8	86	NA	1	1	49	NA	1				
18	chrX	19425322	19425322	C	T	exonic	MAP3K15		nonsynonymous SNV	MAP3K15:NM_001001671:exon13:c.G182	DBSNP+COSMIC	ID=COSM232759	12	110	NA	1	13	107	NA	1	1	93	NA	1				
18	chrX	76778837	76778837	T	A	exonic	ATRX		nonsynonymous SNV	ATRX:NM_138270:exon30:c.A6628T:p.I22	NOVEL		88	215	NA	1	38	277	NA	1	3	128	NA	1				
18	chr2	242274542	242274542	T	C	exonic	SEPTIN2		synonymous SNV	SEPTIN2:NM_001349304:exon3:c.T12C:p.	NOVEL		91	29	NA	1	82	76	NA	1	35	0	NA	1				
18	chrX	32486707	32486707	C	A	exonic	DMD		stopgain	DMD:NM_000109:exon23:c.G3046T:p.E10	COSMIC	ID=COSM492768	122	2	NA	1	148	8	NA	1	5	108	NA	1				
18	chrX	48418407	48418407	C	T	exonic	TBC1D25		nonsynonymous SNV	TBC1D25:NM_001348262:exon6:c.C1159T	DBSNP		183	0	NA	1	171	0	NA	1	132	10	NA	1				
18	chrY	15448080	15448080	T	G	exonic	UTY		nonsynonymous SNV	UTY:NM_001258265:exon12:c.A1657C:p.S	NOVEL		237	0	NA	1	230	0	NA	1	163	10	NA	1				
18	chr17	41960711	41960711	G	A	exonic	MPP2		synonymous SNV	MPP2:NM_001278371:exon3:c.C130T:p.L4	NOVEL		169	0	0	1	192	0	0	1	50	66	1.18487	1				
18	chr9	132565623	132565623	G	A	exonic	TOR1B		synonymous SNV	TOR1B:NM_001317893:exon1:c.G132A:p.	NOVEL		348	192	1.14136	1	349	346	1.01223	2	285	173	1.18216	1				
18	chr8	145726617	145726617	G	T	exonic	PPP1R16A		nonsynonymous SNV	PPP1R16A:NM_001329443:exon11:c.G114	NOVEL		205	180	1.03329	1	210	170	1.00542	1	212	125	1.14059	1				
18	chr12	110346736	110346736	G	A	exonic	TCHP		synonymous SNV	TCHP:NM_001143852:exon8:c.G873A:p.E2	DBSNP		235	0	0	1	231	0	0	1	114	131	1.1135	1				
18	chr14	23395354	23395354	G	A	exonic	PRMT5		synonymous SNV	PRMT5:NM_001282953:exon6:c.C582T:p.F	NOVEL		109	0	0	1	139	6	0.0929965	1	28	32	1.11067	1				
18	chr19	15272102	15272102	C	T	exonic	NOTCH3		nonsynonymous SNV	NOTCH3:NM_000435:exon33:c.G6337A:p.	NOVEL		210	0	0	1	212	0	0	1	106	59	1.09956	1				
18	chr8	144378612	144378612	G	A	exonic	ZNF696		synonymous SNV	ZNF696:NM_030895:exon3:c.G767A:p.R2	DBSNP		225	204	1.05095	1	230	228	1.1188	1	296	164	1.09632	1				
18	chr17	59445778	59445778	C	T	exonic	BCAS3		nonsynonymous SNV	BCAS3:NM_001320470:exon23:c.C2516T:	DBSNP+COSMIC	ID=COSM513537	152	145	1.079	1	128	149	1.2089	1	83	92	1.0948	1				
18	chr9	131223285	131223285	C	T	exonic	ODF2		nonsynonymous SNV	ODF2:NM_001351581:exon3:c.C320T:p.T1	NOVEL		482	245	1.0818	1	770	244	0.978524	1	326	175	1.09319	1				
18	chr9	140123495	140123495	G	T	exonic	RNF224		nonsynonymous SNV	RNF224:NM_001190228:exon3:c.G428T:p	NOVEL		252	0	0	1	351	3	0.0344618	1	101	55	1.08415	1				
18	chr16	85690249	85690249	G	A	exonic	GSE1		synonymous SNV	GSE1:NM_001134473:exon6:c.G978A:p.E3	NOVEL		3	45	1.13446	1	9	39	1.01352	1	0	32	1.0825	1				
18	chr2	61417662	61417662	G	A	exonic	USP34		synonymous SNV	USP34:NM_014709:exon77:c.C9720T:p.F3	NOVEL		270	0	0	1	281	0	0	1	84	90	1.07715	1				
18	chr1	234744660	234744660	C	T	exonic	IRF2BP2		nonsynonymous SNV	IRF2BP2:NM_001077397:exon1:c.G581A:p	DBSNP+COSMIC	ID=COSM570530	96	90	0.78256	2	71	112	0.993744	2	80	43	1.07501	1				
18	chr10	23292221	23292221	A	T	exonic	ARMC3		nonsynonymous SNV	ARMC3:NM_001282747:exon10:c.A820T:p	NOVEL		73	67	1.05768	1	82	67	1.01058	1	49	52	1.07218	1				
18	chr1	231337174	231337174	A	T	exonic	TRIM67		nonsynonymous SNV	TRIM67:NM_001004342:exon5:c.A1445T:p	NOVEL		378	85	0.593822	1	318	124	0.911039	1	154	82	1.06844	1				
18	chr9	12704615	12704615	A	C	exonic	TYRP1		nonsynonymous SNV	TYRP1:NM_000550:exon6:c.A1171C:p.T39	NOVEL		113	187	1.00048	2	121	187	0.983511	2	85	182	1.06666	2				
18	chr16	46729542	46729542	A	T	exonic	ORC6		nonsynonymous SNV	ORC6:NM_014321:exon5:c.A518T:p.D173V	NOVEL		124	2	0.0192077	1	106	4	0.0453605	1	1	54	1.06282	1				
18	chr11	64622288	64622288	C	A	exonic	EHD1		synonymous SNV	EHD1:NM_006795:exon5:c.G1122T:p.L374	NOVEL		372	0	0	1	388	0	0	1	246	126	1.06004	1				
18	chr1	27333302	27333302	C	T	exonic	TENT5B		synonymous SNV	TENT5B:NM_052943:exon2:c.G411A:p.E13	NOVEL		533	0	0	1	508	0	0	1	197	204	1.05942	1				
18	chr18	28991117	28991117	C	T	exonic	DSG4		synonymous SNV	DSG4:NM_001134453:exon14:c.C2118T:p.	DBSNP+COSMIC	ID=COSM280929	220	164	0.943892	1	218	177	1.00707	1	123	127	1.05791	1				
18	chr10	70992652	70992652	G	A	exonic	HKDC1		nonsynonymous SNV	HKDC1:NM_025130:exon3:c.G359A:p.R120	DBSNP		227	215	1.07504	1	231	204	1.05396	1	155	160	1.05778	1				
18	chr14	105409215	105409215	C	T	exonic	AHNAK2		synonymous SNV	AHNAK2:NM_001350929:exon7:c.G12273A	NOVEL		102	104	1.11577	1	86	99	1.20267	1	77	79	1.0546	1				
18	chr16	7645624	7645624	T	G	exonic	RBFOX1		nonsynonymous SNV	RBFOX1:NM_145891:exon5:c.T602G:p.V2	NOVEL		396	0	0	1	422	0	0	1	91	93	1.05257	1				
18	chr17	7577550	7577550	C	T	exonic	TP53		nonsynonymous SNV	TP53:NM_001126115:exon3:c.G335A:p.G1	DBSNP+COSMIC	ID=COSM164685	171	0	0	1	189	0	0	1	2	68	1.05157	1				
18	chr1	34684293	34684293	C	T	exonic	C1orf94		synonymous SNV	C1orf94:NM_001134734:exon7:c.C1728T:p	NOVEL		166	0	0	1	201	3	0.0330502	1	52	53	1.05117	1				
18	chr11	102826044	102826044	T	C	exonic	MMP13		nonsynonymous SNV	MMP13:NM_002427:exon2:c.A299G:p.D10	NOVEL		389	176	0.688452	1	337	253	0.963722	1	242	494	1.0503	2				
18	chr16	50815298	50815298	C	T	exonic	CYLD		stopgain	CYLD:NM_001042355:exon9:c.C1651T:p.Q	NOVEL		19	29	0.731095	1	9	40	1.0183	1	2	64	1.04969	1				
18	chr3	30713409	30713409	A	T	exonic	TGFBR2		nonsynonymous SNV	TGFBR2:NM_003242:exon4:c.A734T:p.E24	NOVEL		370	0	0	1	379	1	0.00591425	1	127	129	1.04938	1				
18	chr15	65108791	65108791	C	T	exonic	PIF1		synonymous SNV	PIF1:NM_001286496:exon12:c.G1848A:p.F	NOVEL		312	0	0	1	313	0	0	1	119	60	1.04904	1				
18	chr13	100634540	100634540	C	A	exonic	ZIC2		nonsynonymous SNV	ZIC2:NM_007129:exon1:c.C222A:p.S74R	NOVEL		33	60	0.780702	1	10	67	1.08541	1	3	86	1.04601	1				
18	chr2	113780305	113780305	C	A	exonic	IL36B		nonsynonymous SNV	IL36B:NM_014438:exon6:c.G441T:p.K147N	DBSNP		257	0	0	1	293	0	0	1	96	96	1.04125	1				
18	chr21	27347463	27347463	T	A	exonic	APP		stopgain	APP:NM_001136129:exon8:c.A985T:p.R32	NOVEL		267	3	0.0245565	1	296	5	0.0373325	1	79	78	1.03462	1				
18	chr8	56436431	56436431	C	T	exonic	XKR4		nonsynonymous SNV	XKR4:NM_052898:exon3:c.C1598T:p.A533	NOVEL		287	0	0	1	309	1	0.00724973	1	126	258	1.03302	2				
18	chr6	74477841	74477841	C	T	exonic	CD109		nonsynonymous SNV	CD109:NM_001159588:exon13:c.C1330T:p	NOVEL		111	4	0.0510308	1	100	10	0.113401	1	1	104	1.03133	2				
18	chr3	129225287	129225287	C	T	exonic	IFT122		stopgain	IFT122:NM_052990:exon19:c.C2353T:p.Q	NOVEL		61	48	0.97325	1	67	56	1.02321	1	33	32	1.02523	1				
18	chr8	25253168	25253168	C	T	exonic	DOCK5		nonsynonymous SNV	DOCK5:NM_024940:exon45:c.C4637T:p.P1	NOVEL		170	127	0.945054	1	144	113	0.988163	1	93	90	1.02418	1				
18	chr22	41536210	41536210	T	C	exonic	EP300		synonymous SNV	EP300:NM_001362843:exon9:c.T1827C:p.\	NOVEL		510	351	0.900977	1	483	356	0.953611	1	303	292	1.022	1				
18	chr19	57175104	57175104	G	A	exonic	ZNF835		nonsynonymous SNV	ZNF835:NM_001005850:exon2:c.C1463T:p	DBSNP+COSMIC	ID=COSM139685	340	148	0.973551	1	237	181	0.973163	1	347	167	1.01683	1				
18	chr14	76211901	76211901	A	G	exonic	TTLL5		synonymous SNV	TTLL5:NM_015072:exon17:c.A1464G:p.T4	NOVEL		272	218	0.983264	1	286	237	1.01843	1	130	124	1.01665	1				
18	chr22	46318854	46318854	C	T	exonic	WNT7B		nonsynonymous SNV	WNT7B:NM_058238:exon4:c.G932A:p.R31	NOVEL		210	189	1.04688	1	213	177	1.01998	1	149	141	1.01252	1				
18	chr10	124221519	124221519	G	T	exonic	HTRA1		synonymous SNV	HTRA1:NM_002775:exon1:c.G351T:p.P117	DBSNP		38	26	0.897848	1	44	27	0.854651	1	37	35	1.01232	1				
18	chr1	118008971	118008971	A	G	exonic	MAN1A2		nonsynonymous SNV	MAN1A2:NM_006699:exon8:c.A1090G:p.K	NOVEL		365	155	0.658776	1	247	194	0.988659	1	88	83	1.0108	1				
18	chr19	31768485	31768485	C	T	exonic	TSHZ3		synonymous SNV	TSHZ3:NM_020856:exon2:c.G2214A:p.K73	NOVEL		498	0	0	1	505	0	0	1	269	131	1.00707	1				
18	chr14	38218309	38218309	T	C	exonic	TTC6		synonymous SNV	TTC6:NM_001310135:exon13:c.T2577C:p.A	NOVEL		701	0	0	1	608	1	0.00369034	1	212	195	0.997757	1				
18	chr4	436024	436024	C	A	exonic	ZNF721		nonsynonymous SNV	ZNF721:NM_133474:exon3:c.G2232T:p.K7	NOVEL		230	155	0.889776	1	205	177	1.04134	1	173	158	0.994062	1				
18	chr9	131760861	131760861	G	A	exonic	NUP188		nonsynonymous SNV	NUP188:NM_015354:exon32:c.G3474A:p.	NOVEL		363	0	0	1	481	0	0	1	259	120	0.990916	1				
18	chr20	44530970	44530970	C	A	exonic	PLTP		nonsynonymous SNV	PLTP:NM_001242920:exon10:c.G826T:p.A	NOVEL		192	0	0	1	189	1	0.0118285	1	136	63	0.990791	1				
18	chr3	125286385	125286385	G	C	exonic	OSBPL11		nonsynonymous SNV	OSBPL11:NM_022776:exon6:c.C721G:p.L2	NOVEL		217	106	0.725292	1	161	137	1.03321	1	83	75	0.988527	1				
18	chr20	21328841	21328841	T	C	exonic	XRN2		nonsynonymous SNV	XRN2:NM_001317960:exon18:c.T1957C:p.	NOVEL		465	5	0.0235116	1	514	11	0.0470887	1	297	137	0.987929	1				
18	chr16	9057131	9057131	C	G	exonic	USP7		nonsynonymous SNV	USP7:NM_003470:exon1:c.G12C:p.Q4H	DBSNP+COSMIC	ID=COSM138063	25	17	0.894559	1	31	26	1.02514	1	20	18	0.986446	1				
18	chr3	196229831	196229831	C	T	exonic	RNF168		nonsynonymous SNV	RNF168:NM_152617:exon1:c.G214A:p.V7	NOVEL		528	0	0	1	610	1	0.00367826	1	167	150	0.985409	1				
18	chr15	78921678	78921678	C	T	exonic	CHRNB4		synonymous SNV	CHRNB4:NM_000750:exon5:c.G969A:p.S3	DBSNP		202	316	0.979139	2	204	365	1.03913	2	177	301	0.985379	2				
18	chr12	129559055	129559055	G	A	exonic	TMEM132D		nonsynonymous SNV	TMEM132D:NM_133448:exon9:c.C2665T:p	NOVEL		571	1	0.00386379	1	622	1	0.00360741	1	203	182	0.984453	1				
18	chr19	54496245	54496245	G	A	exonic	CACNG6		synonymous SNV	CACNG6:NM_031897:exon1:c.G114A:p.K3	NOVEL		289	1	0.0110693	1	219	3	0.0303705	1	146	67	0.984442	1				
18	chr1	204217974	204217974	C	T	exonic	PLEKHA6		nonsynonymous SNV	PLEKHA6:NM_014935:exon12:c.G1799A:p	DBSNP+COSMIC	ID=COSM338564	120	102	1.48616	1	117	159	0.935397	2	102	48	0.98401	1				
18	chr10	75561394	75561394	C	A	exonic	ZSWIM8		nonsynonymous SNV	ZSWIM8:NM_001242488:exon26:c.C5539A	NOVEL		455	0	0	1	418	4	0.0213025	1	172	153	0.980375	1				
18	chr22	41572880	41572880	C	A	exonic	EP300		nonsynonymous SNV	EP300:NM_001362843:exon30:c.C5087A:p	NOVEL		605	0	0	1	618	3	0.0108571	1	178	158	0.979269	1				
18	chr19	41518343	41518343	C	T	exonic	CYP2B6		nonsynonymous SNV	CYP2B6:NM_000767:exon7:c.C1105T:p.H3	DBSNP+COSMIC	ID=COSM585585	820	0	0	1	578	0	0	1	321	146	0.978432	1				

18	chr2	237289242	237289242	G	A	exonic	IQCA1		nonsynonymous SNV	IQCA1:NM_001270584:exon11:c.C1325T:p	NOVEL		618	4	0.0142128	1	651	17	0.0571947	1	13	122	0.978257	1
18	chr1	228476504	228476504	G	T	exonic	OBSCN		nonsynonymous SNV	OBSCN:NM_001098623:exon38:c.G10254T	NOVEL		469	1	0.00688209	1	509	1	0.00636748	1	281	131	0.97774	1
18	chr4	159642618	159642618	C	G	exonic	PPID		nonsynonymous SNV	PPID:NM_005038:exon2:c.G103C:p.E35Q	NOVEL		542	0	0	1	687	3	0.0140861	1	154	135	0.972793	1
18	chr15	29346174	29346174	C	A	exonic	APBA2		synonymous SNV	APBA2:NM_001130414:exon3:c.C87A:p.P2	NOVEL		176	1	0.0181361	1	194	1	0.0166144	1	107	48	0.96918	1
18	chr1	40366998	40366998	G	A	exonic	MYCL		nonsynonymous SNV	MYCL:NM_001033081:exon1:c.C109T:p.P3	NOVEL		320	3	0.0205271	1	298	6	0.0443569	1	100	87	0.968862	1
18	chr1	3754054	3754054	C	T	exonic	CEP104		synonymous SNV	CEP104:NM_014704:exon9:c.G921A:p.Q30	NOVEL		351	0	0	1	395	1	0.00567529	1	96	83	0.965627	1
18	chr4	126372579	126372579	C	T	exonic	FAT4		stopgain	FAT4:NM_001291285:exon9:c.C10414T:p.	COSMIC	ID=COSM360001	497	230	0.92142	1	518	240	1.02579	1	169	146	0.965221	1
18	chr5	37007469	37007469	C	T	exonic	NIPBL		nonsynonymous SNV	NIPBL:NM_015384:exon18:c.C4132T:p.H13	NOVEL		224	90	0.920089	1	209	103	1.06955	1	134	61	0.961932	1
18	chr3	49138008	49138008	C	T	exonic	QARS		nonsynonymous SNV	QARS:NM_001272073:exon11:c.G943A:p.	NOVEL		278	235	1.01242	1	290	258	1.05809	1	174	147	0.953667	1
18	chr1	200878379	200878379	G	A	exonic	INAVA		synonymous SNV	INAVA:NM_001142569:exon8:c.G813A:p.	NOVEL		195	0	0	1	193	0	0	1	58	26	0.951795	1
18	chr10	118434850	118434850	C	T	exonic	HSPA12A		synonymous SNV	HSPA12A:NM_025015:exon12:c.G1470A:p	NOVEL		162	91	0.794933	1	125	96	0.976253	1	82	69	0.951605	1
18	chr3	124017709	124017709	G	A	exonic	KALRN		synonymous SNV	KALRN:NM_001024660:exon6:c.G1035A:p	NOVEL		271	222	0.995212	1	281	249	1.05586	1	200	168	0.950705	1
18	chr3	129302413	129302413	G	A	exonic	PLXND1		synonymous SNV	PLXND1:NM_015103:exon8:c.C2238T:p.P7	DBSNP		118	93	0.974115	1	145	106	0.949107	1	72	60	0.94659	1
18	chr19	35616208	35616208	G	A	exonic	LGI4		synonymous SNV	LGI4:NM_139284:exon9:c.C1503T:p.A501A	NOVEL		344	154	0.992678	1	197	157	0.996735	1	310	137	0.942459	1
18	chr19	5221249	5221249	G	A	exonic	PTPRS		nonsynonymous SNV	PTPRS:NM_130853:exon12:c.C1924T:p.L64	DBSNP		440	0	0	1	499	0	0	1	213	94	0.94154	1
18	chr9	131515106	131515106	G	A	exonic	ZER1		nonsynonymous SNV	ZER1:NM_006336:exon5:c.C769T:p.R257C	DBSNP		301	0	0	1	384	2	0.0210699	1	150	64	0.935968	1
18	chr15	28424299	28424299	C	A	exonic	HERC2		nonsynonymous SNV	HERC2:NM_004667:exon58:c.G9008T:p.S3	NOVEL		253	132	1.1006	1	329	115	0.839138	1	202	86	0.934546	1
18	chr17	40342686	40342686	C	A	exonic	GHDC		nonsynonymous SNV	GHDC:NM_001142623:exon8:c.G1244T:p.	NOVEL		282	0	0	1	337	0	0	1	75	60	0.925554	1
18	chr5	43297155	43297155	C	G	exonic	HMGCS1		nonsynonymous SNV	HMGCS1:NM_001324223:exon3:c.G562C:p	NOVEL		594	1	0.00539511	1	605	0	0	1	309	133	0.925292	1
18	chr4	62936490	62936490	A	C	exonic	ADGRL3		nonsynonymous SNV	ADGRL3:NM_001371342:exon21:c.A4193C	NOVEL		217	189	1.02883	1	229	222	1.10627	1	134	107	0.924594	1
18	chr4	173150845	173150845	A	T	exonic	GALNTL6		synonymous SNV	GALNTL6:NM_001034845:exon3:c.A177T:p	NOVEL		279	346	0.806177	2	272	456	1.01466	2	142	113	0.922832	1
18	chr15	42276125	42276125	A	T	exonic	PLA2G4E		nonsynonymous SNV	PLA2G4E:NM_001206670:exon20:c.T2434A	NOVEL		106	53	1.07003	1	122	56	1.01926	1	72	30	0.920483	1
18	chr2	166011145	166011145	T	C	exonic	SCN3A		synonymous SNV	SCN3A:NM_001081676:exon11:c.A1197G:	NOVEL		348	305	1.03228	1	365	272	0.95965	1	187	148	0.920029	1
18	chr1	93996313	93996313	C	T	exonic	FNBP1L		nonsynonymous SNV	FNBP1L:NM_001024948:exon7:c.C512T:p.	NOVEL		86	46	0.770182	1	63	46	0.94845	1	38	30	0.918749	1
18	chr10	63810723	63810723	T	G	exonic	ARID5B		nonsynonymous SNV	ARID5B:NM_001244638:exon2:c.T81G:p.D	NOVEL		362	263	0.930005	1	347	284	1.01151	1	184	145	0.917818	1
18	chr12	124086715	124086715	G	A	exonic	DDX55		nonsynonymous SNV	DDX55:NM_020936:exon1:c.G20A:p.G7D	NOVEL		129	0	0	1	126	0	0	1	44	34	0.907755	1
18	chr5	158141161	158141161	C	A	exonic	EBF1		synonymous SNV	EBF1:NM_182708:exon11:c.G1062T:p.L35	NOVEL		282	120	0.958235	1	332	132	0.921668	1	236	98	0.902254	1
18	chr1	25944811	25944811	C	T	exonic	MAN1C1		nonsynonymous SNV	MAN1C1:NM_001289010:exon1:c.C523T:p	NOVEL		138	109	0.975302	1	131	124	1.09286	1	93	71	0.901569	1
18	chr3	14960288	14960288	C	T	exonic	FGD5		nonsynonymous SNV	FGD5:NM_001320276:exon13:c.C3517T:p.	NOVEL		108	71	0.876627	1	110	89	1.00513	1	65	49	0.895108	1
18	chr3	51746690	51746690	G	T	exonic	GRM2		stopgain	GRM2:NM_000839:exon3:c.G652T:p.E218	NOVEL		377	2	0.0116627	1	361	12	0.0723029	1	186	140	0.894324	1
18	chr22	30806623	30806623	C	A	exonic	SEC14L2		nonsynonymous SNV	SEC14L2:NM_001204204:exon6:c.C370A:p	NOVEL		108	87	0.986039	1	116	70	0.845801	1	68	51	0.892499	1
18	chr10	88418318	88418318	C	A	exonic	OPN4		nonsynonymous SNV	OPN4:NM_033282:exon4:c.C502A:p.R168S	NOVEL		608	6	0.021597	1	612	7	0.025415	1	220	164	0.8894	1
18	chr11	14520410	14520410	G	A	exonic	COPB1		nonsynonymous SNV	COPB1:NM_001144061:exon2:c.C65T:p.S2	NOVEL		58	22	0.607774	1	59	41	0.92144	1	127	50	0.88408	1
18	chr12	49982335	49982335	C	T	exonic	FAM186B		synonymous SNV	FAM186B:NM_032130:exon6:c.G2436A:p.	NOVEL		152	121	0.979563	1	161	126	0.98667	1	114	84	0.883484	1
18	chr16	56500169	56500169	A	G	exonic	OGFOD1		nonsynonymous SNV	OGFOD1:NM_001324357:exon5:c.A551G:	NOVEL		208	0	0	1	176	0	0	1	18	78	0.879529	1
18	chr2	220422622	220422622	G	A	exonic	OBSL1		nonsynonymous SNV	OBSL1:NM_001173431:exon11:c.C3713T:p	NOVEL		96	79	0.997697	1	83	73	1.05167	1	82	59	0.871399	1
18	chr12	50366960	50366960	G	A	exonic	AQP6		nonsynonymous SNV	AQP6:NM_001652:exon1:c.G4A:p.D2N	COSMIC	ID=COSM652024	203	0	0	1	181	2	0.0245619	1	81	58	0.868956	1
18	chr11	3838720	3838720	G	T	exonic	PGAP2		nonsynonymous SNV	PGAP2:NM_014489:exon3:c.G303T:p.W10	NOVEL		415	0	0	1	449	2	0.00996636	1	294	113	0.868918	1
18	chr7	24690240	24690240	C	T	exonic	MPP6		nonsynonymous SNV	MPP6:NM_001303037:exon5:c.C560T:p.P1	NOVEL		593	0	0	1	641	1	0.00504642	1	221	87	0.868596	1
18	chr16	14354873	14354873	C	A	exonic	MRTFB		nonsynonymous SNV	MRTFB:NM_001365411:exon15:c.C2839A	NOVEL		404	227	0.795071	1	369	279	0.967637	1	219	148	0.839808	1
18	chr11	44135744	44135744	G	A	exonic	EXT2		synonymous SNV	EXT2:NM_000401:exon4:c.G735A:p.L245L	NOVEL		155	0	0	1	205	2	0.0217142	1	101	37	0.839107	1
18	chr15	43500868	43500868	A	C	exonic	EPB42		nonsynonymous SNV	EPB42:NM_000119:exon7:c.T1028G:p.L34	NOVEL		410	0	0	1	428	0	0	1	183	67	0.838744	1
18	chr1	93142764	93142764	T	C	exonic	EVI5		nonsynonymous SNV	EVI5:NM_001308248:exon10:c.A1241G:p.	NOVEL		153	122	0.980475	1	176	146	1.01901	1	89	59	0.830185	1
18	chr18	28989548	28989548	G	A	exonic	DSG4		synonymous SNV	DSG4:NM_001134453:exon13:c.G2067A:p	NOVEL		81	80	1.09818	1	120	90	0.963178	1	77	51	0.829745	1
18	chr11	62648673	62648673	T	C	exonic	SLC3A2		nonsynonymous SNV	SLC3A2:NM_001013251:exon1:c.T178C:p.	NOVEL		83	65	0.970647	1	78	57	0.948908	1	120	43	0.825611	1
18	chr19	48846004	48846004	C	T	exonic	TMEM143		nonsynonymous SNV	TMEM143:NM_001303540:exon4:c.G458A	NOVEL		436	0	0	1	365	0	0	1	214	74	0.804144	1
18	chr7	70229938	70229938	T	A	exonic	AUTS2		nonsynonymous SNV	AUTS2:NM_001127231:exon8:c.T1415A:p.	NOVEL		209	63	0.743513	1	229	101	0.991576	1	114	40	0.798709	1
18	chr1	155254957	155254957	A	C	exonic	HCN3		nonsynonymous SNV	HCN3:NM_020897:exon5:c.A1091C:p.Y364	NOVEL		162	0	0	1	152	0	0	1	72	25	0.792534	1
18	chr1	29030739	29030739	G	A	exonic	GMEB1		nonsynonymous SNV	GMEB1:NM_001319674:exon8:c.G766A:p.	NOVEL		70	78	1.16478	1	100	83	1.01932	1	62	36	0.764999	1
18	chr20	61512428	61512428	G	C	exonic	DIDO1		nonsynonymous SNV	DIDO1:NM_001193369:exon16:c.C4880G:p	NOVEL		141	60	0.659728	1	124	76	0.854018	1	171	55	0.761639	1
18	chr4	25864329	25864329	A	G	exonic	SEL1L3		nonsynonymous SNV	SEL1L3:NM_015187:exon1:c.T130C:p.S44P	NOVEL		8	12	1.32605	1	21	13	0.859306	1	20	10	0.694166	1
18	chr5	178772261	178772261	C	T	exonic	ADAMTS2		synonymous SNV	ADAMTS2:NM_014244:exon1:c.G69A:p.L2	NOVEL		95	0	0	1	87	0	0	1	52	15	0.68844	1
18	chr7	123257685	123257685	G	A	exonic	ASB15		synonymous SNV	ASB15:NM_080928:exon5:c.G345A:p.L115	NOVEL		573	0	0	1	604	0	0	1	326	71	0.549942	1
18	chr19	50365106	50365106	G	A	exonic	PNKP		synonymous SNV	PNKP:NM_007254:exon14:c.C1221T:p.T40	NOVEL		407	0	0	1	335	0	0	1	222	44	0.517685	1
18	chr19	53518648	53518648	A	T	exonic	ERVV-1		nonsynonymous SNV	ERVV-1:NM_152473:exon1:c.A1305T:p.K4	NOVEL		672	158	0.611077	1	388	159	0.65327	1	437	85	0.509616	1
18	chr11	4510659	4510659	G	T	exonic	OR52K1		nonsynonymous SNV	OR52K1:NM_001005171:exon1:c.G529T:p.	DBSNP		398	0	0	1	407	0	0	1	472	48	0.28889	1
18	chr18	28711619	28711619	C	G	exonic	DSC1		nonsynonymous SNV	DSC1:NM_004948:exon15:c.G2425C:p.G80	NOVEL		409	0	0	1	414	0	0	1	221	34	0.277666	1
18	chr11	55595507	55595507	C	A	exonic	OR5L2		nonsynonymous SNV	OR5L2:NM_001004739:exon1:c.C813A:p.D	DBSNP+COSMIC	ID=COSM688811	362	0	0	1	394	0	0	1	441	33	0.217886	1
18	chr11	4621783	4621783	C	A	exonic	TRIM68		nonsynonymous SNV	TRIM68:NM_001304496:exon6:c.G512T:p.	NOVEL		312	1	0.00706098	1	338	0	0	1	214	15	0.204998	1
18	chr15	65499240	65499240	C	T	exonic	CILP		nonsynonymous SNV	CILP:NM_003613:exon4:c.G304A:p.A102T	NOVEL		228	1	0.0140179	1	261	0	0	1	173	9	0.154763	1
18	chr11	6585271	6585271	C	G	exonic	DNHD1		nonsynonymous SNV	DNHD1:NM_144666:exon31:c.C10201G:p.	NOVEL		295	0	0	1	286	1	0.00783071	1	293	14	0.14272	1
18	chr1	985066	985066	G	T	exonic	AGRN		synonymous SNV	AGRN:NM_198576:exon26:c.G4635T:p.V1	NOVEL		330	0	0	1	318	0	0	1	192	10	0.103094	1
18	chr4	77662190	77662190	G	C	exonic	SHROOM3		nonsynonymous SNV	SHROOM3:NM_020859:exon5:c.G2864C:p	NOVEL		249	2	0.0176103	1	276	0	0	1	228	10	0.0874999	1
18	chr9	111849582	111849582	C	G	exonic	TMEM245		synonymous SNV	TMEM245:NM_032012:exon6:c.G1191C:p.	DBSNP		631	0	0	1	855	0	0	1	505	14	0.084422	1
18	chr6	44376312	44376312	C	A	exonic	CDC5L		nonsynonymous SNV	CDC5L:NM_001253:exon8:c.C1035A:p.N34	DBSNP		855	2	0.00515773	1	819	0	0	1	761	21	0.0825775	1
18	chr1	38450380	38450380	T	A	exonic	SF3A3		nonsynonymous SNV	SF3A3:NM_001320830:exon3:c.A216T:p.Q	NOVEL		490	1	0.0045012	1	499	0	0	1	275	9	0.0659946	1
18	chr2	31483654	31483654	C	T	exonic	EHD3		nonsynonymous SNV	EHD3:NM_014600:exon4:c.C781T:p.P261S	NOVEL		606	0	0	1	557	0	0	1	512	16	0.063106	1
18	chr8	130863139	130863139	T	G	exonic	FAM49B		nonsynonymous SNV	FAM49B:NM_001353297:exon7:c.A217C:p	NOVEL		147	2	0.0296656	1	86	74	1.03943	1	146	1	0.0209186	1
18	chr5	167420067	167420067	A	C	exonic	TENM2		nonsynonymous SNV	TENM2:NM_001080428:exon3:c.A493C:p.	NOVEL		367	4	0.0346101	1	287	104	0.861738	1	306	2	0.0199677	1
18	chr19	56969558	56969558	C	T	exonic	ZNF667		stopgain	ZNF667:NM_022103:exon4:c.G219A:p.W7	NOVEL		228	1	0.0140179	1	157	23	0.28717	1	167	1	0.0186288	1
18	chr10	60272949	60272949	C	G	exonic	BICC1		nonsynonymous SNV	BICC1:NM_001080512:exon1:c.C46G:p.P16	NOVEL		228	3	0.0287024	1	134	107	0.997815	1	139	1	0.014875	1
18	chr3	142388319	142388319	G	A	exonic	PLS1		nonsynonymous SNV	PLS1:NM_001145319:exon3:c.G158A:p.R5	DBSNP+COSMIC	ID=COSM103942	418	0	0	1	343	41	0.239958	1	195	1	0.010625	1
18	chr11	64694308	64694308	C	A	exonic	PPP2R5B		nonsynonymous SNV	PPP2R5B:NM_006244:exon3:c.C324A:p.N1	NOVEL		271	8	0.0633717	1	149	97	0.886176	1	338	1	0.00923198	1
18	chr10	75276458	75276458	A	T	exonic	USP54		nonsynonymous SNV	USP54:NM_001320437:exon18:c.T3555A:p	NOVEL		340	11	0.069262	1	235	149	0.872044	1	245	1	0.00846543	1
18	chr15	50534616	50534616	G	A	exonic	HDC		synonymous SNV	HDC:NM_001306146:exon11:c.C1731T:p.S	NOVEL		454	22	0.148365	1	532	0	0	1	412	1	0.00757783	1
18	chr4	39303934	39303934	T	A	exonic	RFC1		nonsynonymous SNV	RFC1:NM_001363495:exon18:c.A2421T:p.	NOVEL		508	29	0.119353	1	645	1	0.00347897	1	285	1	0.00728146	1
18	chr2	27325271	27325271	T	G	exonic	CGREF1		synonymous SNV	CGREF1:NM_001166239:exon4:c.A174C:p.	NOVEL		438	5	0.0249446	1	256	139	0.790862	1	333	1	0.00623502	1
18	chr19	23159694	23159694	T	A	exonic	ZNF728		stopgain	ZNF728:NM_001267716:exon4:c.A445T:p.	NOVEL		752	9	0.0379642	1	547	200	0.867417	1	571	1	0.00608777	1
18	chr2	170115556	170115556	A	G	exonic	LRP2		nonsynonymous SNV	LRP2:NM_004525:exon17:c.T2492C:p.V83	NOVEL		751	19	0.0545346	1	714	0	0	1	388	1	0.00535346	1
18	chr2	202590177	202590177	C	A	exonic	ALS2		synonymous SNV	ALS2:NM_020919:exon20:c.G3249T:p.G10	NOVEL		865	23	0.0572433	1	947	0	0	1	473	1	0.00439345	1
18	chr20	62172651	62172651	G	C	exonic	SRMS		nonsynonymous SNV	SRMS:NM_080823:exon7:c.C1178G:p.T39	NOVEL		590	26	0.0932829	1	646	0	0	1	785	1	0.00398173	1
18	chr2	179612758	179612758	T	C	exonic	TTN		nonsynonymous SNV	TTN:NM_133379:exon46:c.A14369G:p.E47	NOVEL		946	21	0.0479957	1	606	406	0.901631	1	643	1	0.00323369	1
18	chr4	123184067	123184067	T	C	exonic	KIAA1109		nonsynonymous SNV	KIAA1109:NM_015312:exon41:c.T6911C:p	NOVEL		260	6	0.0656953	1	159	77	1.05705	1	106	0	0	1
18	chr16	84027988	84027988	G	A	exonic	NECAB2		synonymous SNV	NECAB2:NM_001329749:exon6:c.G429A:p	NOVEL		146	3	0.0243642	1	28	133	1.03047	1	94	0	0	1
18	chr3	133362092	133362092	C	G	exonic	TOPBP1		nonsynonymous SNV	TOPBP1:NM_001363889:exon12:c.G1958C	NOVEL		202	2	0.0216675	1	92	71	0.978935	1	214	0	0	1
18	chr12	43886375	43886375	G	A	exonic	ADAMTS20		stopgain	ADAMTS20:NM_025003:exon6:c.C1009T:p	NOVEL		291	4	0.0299673	1	165	116	0.927758	1	202	0	0	1
18	chr2	196851835	196851835	T	A	exonic	DNAH7		nonsynonymous SNV	DNAH7:NM_018897:exon14:c.A1709T:p.K5	DBSNP		430	8	0.0403669	1	259	176	0.909299	1	222	0	0	1
18	chr18	21891950	21891950	C	T	exonic	OSBPL1A		nonsynonymous SNV	OSBPL1A:NM_080597:exon13:c.G1090A:p	NOVEL		294	6	0.0442018	1	181	122	0.9049	1	194	0	0	1
18	chr1	25889602	25889602	G	A	exonic	LDLRAP1		nonsynonymous SNV	LDLRAP1:NM_015627:exon6:c.G574A:p.V1	DBSNP+COSMIC	ID=COSM584618	214	1	0.0102795	1	135	89	0.892946	1	177	0	0	1
18	chr1	38095242	38095242	C	T	exonic	RSPO1		nonsynonymous SNV	RSPO1:NM_001242908:exon3:c.G92A:p.R3	DBSNP+COSMIC	ID=COSM677011	322	4	0.0271176	1	225	148	0.891736	1	210	0	0	1
18	chr16	77859271	77859271	C	A	exonic	VAT1L		nonsynonymous SNV	VAT1L:NM_020927:exon3:c.C492A:p.N164	NOVEL		60	4	0.0756305	1	18	45	0.891011	1	30	0	0	1
18	chr18	44260410	44260410	C	T	exonic	ST8SIA5		synonymous SNV	ST8SIA5:NM_001307987:exon6:c.G633A:p	COSMIC	ID=COSM679127	549	11	0.0434124	1	317	200	0.869406	1	429	0	0	1
18	chr8	8185367	8185367	G	A	exonic	PRAG1		synonymous SNV	PRAG1:NM_001080826:exon6:c.C2925T:p.	DBSNP		252	11	0.0924371	1	185	116	0.866113	1	234	0	0	1
18	chr7	100655589	100655589	C	G	exonic	MUC12		nonsynonymous SNV	MUC12:NM_001164462:exon6:c.C15264G	DBSNP		544	5	0.0292358	1	410	149	0.863561	1	325	0	0	1
18	chr15	25924692	25924692	C	T	exonic	ATP10A		synonymous SNV	ATP10A:NM_024490:exon21:c.G4296A:p.L	NOVEL		389	7	0.056744	1	324	114	0.843236	1	263	0	0	1
18	chr2	215815756	215815756	C	T	exonic	ABCA12		synonymous SNV	ABCA12:NM_015657:exon37:c.G5745A:p.E	NOVEL		510	5	0.0214572	1	325	193	0.837357	1	313	0	0	1
18	chr1	190250767	190250767	C	T	exonic	BRINP3		nonsynonymous SNV	BRINP3:NM_199051:exon3:c.G350A:p.R11	DBSNP		512	8	0.0497628	1	401	139	0.835909	1	314	0	0	1
18	chr9	79932614	79932614	G	T	exonic	VPS13A		nonsynonymous SNV	VPS13A:NM_001018037:exon39:c.G4839T	NOVEL		107	2	0.0589007	1	97	27	0.705441	1	78	0	0	1
18	chr1	115258747	115258747	C	G	exonic	NRAS		nonsynonymous SNV	NRAS:NM_002524:exon2:c.G35C:p.G12A	DBSNP+COSMIC	ID=COSM565;OC	200	19	0.191743	1	174	72	0.65778	1	155	0	0	1
18	chr1	115258747	115258747	C	T	exonic	NRAS		nonsynonymous SNV	NRAS:NM_002524:exon2:c.G35A:p.G12D	DBSNP+COSMIC	ID=COSM564;OC	212	6	0.055	1	0	0	0	0	86	69	0.898	1
18	chr2	190718800	190718800	C	T	exonic	PMS1		stopgain	PMS1:NM_001289409:exon6:c.C430T:p.Q1	DBSNP		115	1	0.0190525	1	87	31	0.590423	1	73	0	0	1
18	chr1	233464451	233464451	C	G	exonic	MAP3K21		nonsynonymous SNV	MAP3K21:NM_032435:exon1:c.C677G:p.P	NOVEL		81	16	0.533539	1	103	19	0.505745	1	110	0	0	1
18	chr16	20331613	20331613	G	A	exonic	GP2		stopgain	GP2:NM_001007242:exon4:c.C388T:p.Q13	NOVEL		285	0	0	1	259	53	0.381772	1	166	0	0	1
18	chr7	80295797	80295797	A	G	exonic	CD36		nonsynonymous SNV	CD36:NM_001371080:exon5:c.A275G:p.N9	DBSNP		505	0	0	1	466	56	0.347565	1	424	0	0	1
18	chr1	236332007	236332007	T	A	exonic	GPR137B		nonsynonymous SNV	GPR137B:NM_003272:exon2:c.T416A:p.V1	NOVEL		576	1	0.00560586	1	557	56	0.296664	1	497	0	0	1
18	chr1	155261658	155261658	C	A	exonic	PKLR		nonsynonymous SNV	PKLR:NM_000298:exon10:c.G1507T:p.A50	NOVEL		159	0	0	1	162	8	0.15282	1	112	0	0	1
18	chr17	75478338	75478338	C	T	exonic	SEPTIN9		synonymous SNV	SEPTIN9:NM_001113495:exon2:c.C498T:p	DBSNP+COSMIC	ID=COSM274430	327	0	0	1	322	21	0.137597	1	218	0	0	1
18	chr5	13735328	13735328	G	A	exonic	DNAH5		synonymous SNV	DNAH5:NM_001369:exon68:c.C11673T:p.	NOVEL		413	0	0	1	382	15	0.122411	1	222	0	0	1
18	chr1	42921619	42921619	C	T	exonic	ZMYND12		nonsynonymous SNV	ZMYND12:NM_032257:exon1:c.G50A:p.C1	NOVEL		398	0	0	1	410	17	0.0894755	1	157	0	0	1
18	chr4	3424171	3424171	G	A	exonic	RGS12		synonymous SNV	RGS12:NM_198227:exon9:c.G963A:p.P321	DBSNP		413	0	0	1	333	13	0.0844404	1	275	0	0	1
18	chr11	111835360	111835360	C	A	exonic	DIXDC1		nonsynonymous SNV	DIXDC1:NM_001278542:exon3:c.C145A:p.	NOVEL		114	11	0.194488	1	127	2	0.0348436	1	97	0	0	1
18	chr3	196018240	196018240	A	G	exonic	TCTEX1D2		nonsynonymous SNV	TCTEX1D2:NM_001351628:exon5:c.T382C	NOVEL		223	27	0.238689	1	266	3	0.0250641	1	199	0	0	1
18	chr19	42930715	42930715	G	T	exonic	LIPE		nonsynonymous SNV	LIPE:NM_005357:exon1:c.C587A:p.S196Y	NOVEL		401	28	0.209516	1	356	4	0.0249713	1	301	0	0	1
18	chr11	85445743	85445743	G	T	exonic	SYTL2		nonsynonymous SNV	SYTL2:NM_001162951:exon6:c.C626A:p.T2	NOVEL		387	17	0.0929988	1	470	5	0.023657	1	358	0	0	1
18	chr15	26806143	26806143	T	C	exonic	GABRB3		nonsynonymous SNV	GABRB3:NM_001191320:exon6:c.A761G:p	NOVEL		276	18	0.196536	1	282	2	0.0228155	1	208	0	0	1
18	chr1	19244985	19244985	G	A	exonic	IFFO2		synonymous SNV	IFFO2:NM_001136265:exon4:c.C834T:p.D2	NOVEL		332	45	0.263804	1	405	3	0.0165251	1	326	0	0	1
18	chr1	170961456	170961456	T	A	exonic	MROH9		nonsynonymous SNV	MROH9:NM_001163629:exon12:c.T1180A	NOVEL		373	16	0.133042	1	399	2	0.0161966	1	218	0	0	1
18	chr2	119729107	119729107	C	A	exonic	MARCO		nonsynonymous SNV	MARCO:NM_006770:exon4:c.C457A:p.P15	NOVEL		482	55	0.226359	1	422	3	0.0158641	1	388	0	0	1
18	chr7	53104153	53104153	C	A	exonic	POM121L12		synonymous SNV	POM121L12:NM_182595:exon1:c.C789A:p	DBSNP		574	48	0.247724	1	696	3	0.0139047	1	475	0	0	1
18	chr3	78763636	78763636	T	A	exonic	ROBO1		nonsynonymous SNV	ROBO1:NM_001145845:exon6:c.A839T:p.	NOVEL		848	112	0.257844	1	993	6	0.013498	1	489	0	0	1
18	chr22	32110923	32110923	C	T	exonic	PRR14L		nonsynonymous SNV	PRR14L:NM_173566:exon4:c.G2902A:p.E9	NOVEL		531	27	0.10694	1	604	3	0.0111075	1	398	0	0	1
18	chr6	129618969	129618969	G	A	exonic	LAMA2		nonsynonymous SNV	LAMA2:NM_000426:exon21:c.G2996A:p.C	NOVEL		131	16	0.159688	1	134	1	0.00924011	1	68	0	0	1
18	chr2	95815673	95815673	C	G	exonic	ZNF514		nonsynonymous SNV	ZNF514:NM_001318005:exon5:c.G776C:p.	NOVEL		668	76	0.225762	1	763	3	0.00880189	1	461	0	0	1
18	chr3	147130393	147130393	G	A	exonic	ZIC1		synonymous SNV	ZIC1:NM_003412:exon2:c.G1071A:p.T357	COSMIC	ID=COSM103962	457	35	0.157222	1	531	2	0.00843308	1	318	0	0	1
18	chr1	46827428	46827428	C	G	exonic	NSUN4		nonsynonymous SNV	NSUN4:NM_001256127:exon6:c.C918G:p.	NOVEL		260	18	0.143099	1	330	1	0.00678977	1	180	0	0	1
18	chr2	102481432	102481432	G	A	exonic	MAP4K4		nonsynonymous SNV	MAP4K4:NM_004834:exon16:c.G1652A:p.	DBSNP		240	35	0.281284	1	335	1	0.00668873	1	193	0	0	1
18	chr11	113614574	113614574	C	T	exonic	ZW10		nonsynonymous SNV	ZW10:NM_004724:exon10:c.G1461A:p.M4	DBSNP		992	46	0.0979422	1	1030	3	0.00652686	1	832	0	0	1
18	chr19	3600297	3600297	C	T	exonic	TBXA2R		nonsynonymous SNV	TBXA2R:NM_001060:exon2:c.G336A:p.M1	NOVEL		536	20	0.115471	1	500	1	0.00646667	1	375	0	0	1
18	chr17	11795152	11795152	G	C	exonic	DNAH9		nonsynonymous SNV	DNAH9:NM_004662:exon4:c.G107C:p.R36	NOVEL		391	24	0.127812	1	405	1	0.0055355	1	125	0	0	1
18	chr7	99764176	99764176	G	C	exonic	GAL3ST4		synonymous SNV	GAL3ST4:NM_024637:exon3:c.C378G:p.T1	DBSNP		532	15	0.088028	1	591	1	0.00547264	1	346	0	0	1
18	chr8	2800056	2800056	G	T	exonic	CSMD1		synonymous SNV	CSMD1:NM_033225:exon69:c.C10473A:p.	NOVEL		420	15	0.0762099	1	433	1	0.00517838	1	299	0	0	1
18	chr1	60375577	60375577	A	G	exonic	CYP2J2		nonsynonymous SNV	CYP2J2:NM_000775:exon5:c.T740C:p.L247	NOVEL		381	47	0.242697	1	524	1	0.00428079	1	254	0	0	1
18	chr12	12870942	12870942	C	T	exonic	CDKN1B		stopgain	CDKN1B:NM_004064:exon1:c.C169T:p.Q5	COSMIC	ID=COSM542124	438	26	0.123841	1	531	1	0.00422446	1	306	0	0	1
18	chr15	40564444	40564444	C	T	exonic	BUB1B-PAK6;PAK6		nonsynonymous SNV	PAK6:NM_001276717:exon4:c.C878T:p.P2	DBSNP		735	59	0.238533	1	788	1	0.00410621	1	549	0	0	1
18	chr9	107266706	107266706	C	A	exonic	OR13F1		nonsynonymous SNV	OR13F1:NM_001004485:exon1:c.C163A:p.	NOVEL		882	25	0.0884809	1	1073	1	0.0037863	1	567	0	0	1
18	chr17	38905562	38905562	C	G	exonic	KRT25		synonymous SNV	KRT25:NM_181534:exon7:c.G1191C:p.G39	NOVEL		1009	77	0.156701	1	1069	1	0.00210039	1	789	0	0	1
18	chr12	130922968	130922968	G	C	exonic	RIMBP2		nonsynonymous SNV	RIMBP2:NM_015347:exon10:c.C1547G:p.T	NOVEL		83	10	0.237644	1	141	0	0	1	100	0	0	1
18	chr10	75139843	75139843	G	C	exonic	ANXA7		nonsynonymous SNV	ANXA7:NM_001320879:exon9:c.C915G:p.I	NOVEL		432	44	0.204294	1	507	0	0	1	248	0	0	1
18	chr4	99030375	99030375	C	A	exonic	STPG2		nonsynonymous SNV	STPG2:NM_174952:exon4:c.G469T:p.G157	COSMIC	ID=COSM298934	115	8	0.18943	1	162	0	0	1	59	0	0	1
18	chr11	14901822	14901822	C	T	exonic	CYP2R1		nonsynonymous SNV	CYP2R1:NM_024514:exon3:c.G860A:p.G28	DBSNP+COSMIC	ID=COSM690078	502	44	0.178102	1	584	0	0	1	534	0	0	1
18	chr1	6214876	6214876	C	T	exonic	CHD5		nonsynonymous SNV	CHD5:NM_015557:exon5:c.G589A:p.A197	DBSNP+COSMIC	ID=COSM467209	119	10	0.171325	1	109	0	0	1	96	0	0	1
18	chr16	8732953	8732953	G	A	exonic	METTL22		nonsynonymous SNV	METTL22:NM_024109:exon6:c.G706A:p.G	DBSNP		278	23	0.168877	1	331	0	0	1	209	0	0	1
18	chr1	237870356	237870356	C	G	exonic	RYR2		nonsynonymous SNV	RYR2:NM_001035:exon68:c.C9688G:p.Q32	NOVEL		747	39	0.160495	1	766	0	0	1	413	0	0	1
18	chr6	28212980	28212980	C	T	exonic	ZKSCAN4		nonsynonymous SNV	ZKSCAN4:NM_001304506:exon5:c.G1087A	NOVEL		649	45	0.143305	1	671	0	0	1	559	0	0	1
18	chr17	68128200	68128200	C	T	exonic	KCNJ16		nonsynonymous SNV	KCNJ16:NM_001291623:exon3:c.C77T:p.S2	DBSNP		539	37	0.141967	1	649	0	0	1	346	0	0	1
18	chr10	75258763	75258763	G	A	exonic	USP54		nonsynonymous SNV	USP54:NM_001320437:exon22:c.C4493T:p	NOVEL		209	14	0.13875	1	289	0	0	1	138	0	0	1
18	chr12	89745483	89745483	C	T	exonic	DUSP6		nonsynonymous SNV	DUSP6:NM_001946:exon1:c.G334A:p.E112	NOVEL		357	18	0.106084	1	356	0	0	1	235	0	0	1
18	chr19	49310330	49310330	A	G	exonic	BCAT2		nonsynonymous SNV	BCAT2:NM_001190:exon2:c.T26C:p.I9T	NOVEL		305	10	0.101908	1	285	0	0	1	210	0	0	1
18	chr12	56360854	56360854	C	T	exonic	CDK2		nonsynonymous SNV	CDK2:NM_001290230:exon1:c.C62T:p.A21	NOVEL		409	19	0.0981114	1	448	0	0	1	250	0	0	1
18	chr5	55110739	55110739	C	A	exonic	DDX4		nonsynonymous SNV	DDX4:NM_001166534:exon13:c.C1279A:p.	NOVEL		941	29	0.0959717	1	987	0	0	1	536	0	0	1
18	chr9	131339128	131339128	C	T	exonic	SPTAN1		synonymous SNV	SPTAN1:NM_001130438:exon6:c.C678T:p.	COSMIC	ID=COSM577637	561	16	0.0890146	1	711	0	0	1	333	0	0	1
18	chr19	46257881	46257881	C	T	exonic	BHMG1		nonsynonymous SNV	BHMG1:NM_001310124:exon7:c.C701T:p.	NOVEL		429	12	0.0873493	1	338	0	0	1	207	0	0	1
18	chr3	33586219	33586219	G	A	exonic	CLASP2		nonsynonymous SNV	CLASP2:NM_001207044:exon25:c.C2632T:	NOVEL		215	8	0.0792857	1	263	0	0	1	142	0	0	1
18	chr14	88477746	88477746	G	A	exonic	GPR65		synonymous SNV	GPR65:NM_003608:exon2:c.G555A:p.L185	NOVEL		1129	30	0.0572068	1	1108	0	0	1	675	0	0	1
18	chr11	94335111	94335111	G	A	exonic	PIWIL4		nonsynonymous SNV	PIWIL4:NM_152431:exon12:c.G1531A:p.G	COSMIC	ID=COSM719873	595	15	0.0543464	1	608	0	0	1	553	0	0	1
18	chr19	8186141	8186141	C	A	splicing	FBN3	NM_032447:exon25:c.			NOVEL		203	278	0.927655	2	215	275	0.909128	2	95	177	1.00052	2
18	chr11	115088712	115088712	C	A	splicing	CADM1	NM_001098517:exon6			NOVEL		450	19	0.0895345	1	480	17	0.0768733	1	134	229	0.987174	2
18	chr10	63810761	63810761	T	A	splicing	ARID5B	NM_032199:exon5:c.8			NOVEL		341	254	0.943466	1	312	263	1.02795	1	170	140	0.940483	1
18	chr17	42156034	42156034	C	G	splicing	HDAC5	NM_001015053:exon2			NOVEL		494	0	0	1	486	1	0.00461481	1	141	98	0.853911	1
18	chr7	128525150	128525150	C	T	splicing	KCP	NM_001366122:exon2			NOVEL		124	45	0.854757	1	143	68	1.04411	1	139	48	0.789313	1
18	chr18	2772246	2772246	A	T	splicing	SMCHD1	NM_015295:exon41:c.			NOVEL		259	0	0	1	306	0	0	1	120	54	0.646292	1
18	chr11	61643664	61643664	T	A	splicing	FADS3	NM_021727:exon10:c.			NOVEL		95	3	0.0676558	1	61	43	0.92922	1	99	0	0	1
18	chr3	63264352	63264352	G	T	splicing	SYNPR	NM_001130003:exon2			NOVEL		259	5	0.0418577	1	162	106	0.888903	1	283	0	0	1
18	chr7	16918270	16918270	C	A	splicing	AGR3	NM_176813:exon2:UT			NOVEL		409	17	0.128102	1	489	2	0.0131968	1	208	0	0	1
18	chr8	145139945	145139945	C	T	splicing	GPAA1	NM_003801:exon9:c.1			DBSNP+COSMIC	ID=COSM109717	222	9	0.0861073	1	276	0	0	1	194	0	0	1
19	chr16	1555550	1555550	G	A	exonic	TELO2		stopgain	TELO2:NM_001351846:exon16:c.G1982A:p	COSMIC	ID=COSM568748	115	0	0	1	45	68	1.23612	1				
19	chr7	114562577	114562577	G	C	exonic	MDFIC		nonsynonymous SNV	MDFIC:NM_001166346:exon1:c.G106C:p.V	NOVEL		68	0	0	1	21	30	1.20832	1				
19	chr6	70854106	70854106	T	C	exonic	COL19A1		nonsynonymous SNV	COL19A1:NM_001858:exon24:c.T1631C:p.	NOVEL		124	67	1.08778	1	111	69	1.17076	1				
19	chr3	119334905	119334905	C	T	exonic	PLA1A		synonymous SNV	PLA1A:NM_001206961:exon5:c.C192T:p.N	DBSNP		211	1	0.0146273	1	118	78	1.15989	1				
19	chr19	46025441	46025441	G	A	exonic	VASP		synonymous SNV	VASP:NM_003370:exon5:c.G444A:p.P148P	DBSNP+COSMIC	ID=COSM556163	117	37	0.745044	1	47	60	1.15186	1				
19	chr17	10542987	10542987	C	T	exonic	MYH3		nonsynonymous SNV	MYH3:NM_002470:exon23:c.G2815A:p.A9	NOVEL		180	0	0	1	82	100	1.12865	1				
19	chr18	12254914	12254914	G	A	exonic	CIDEA		stopgain	CIDEA:NM_001318383:exon1:c.G99A:p.W3	NOVEL		67	49	0.888595	1	44	51	1.10275	1				
19	chr19	15302350	15302350	T	A	exonic	NOTCH3		synonymous SNV	NOTCH3:NM_000435:exon6:c.A921T:p.T30	NOVEL		319	187	1.14602	1	167	193	1.10125	1				
19	chr11	18723296	18723296	G	A	exonic	TMEM86A		nonsynonymous SNV	TMEM86A:NM_153347:exon3:c.G463A:p.	NOVEL		464	0	0	1	301	168	1.0903	1				
19	chr3	183013205	183013205	G	A	exonic	MCF2L2		stopgain	MCF2L2:NM_015078:exon13:c.C1558T:p.Q	COSMIC	ID=COSM399288	244	0	0	1	153	91	1.087	1				
19	chr17	3119702	3119702	C	A	exonic	OR1A1		nonsynonymous SNV	OR1A1:NM_014565:exon1:c.C788A:p.T263	COSMIC	ID=COSM648604	347	0	0	1	154	173	1.08675	1				
19	chr14	75415227	75415227	G	A	exonic	PGF		synonymous SNV	PGF:NM_001207012:exon4:c.C375T:p.H12	DBSNP+COSMIC	ID=COSM726300	94	89	1.02307	1	93	104	1.08442	1				
19	chr20	34560713	34560713	T	A	exonic	CNBD2		nonsynonymous SNV	CNBD2:NM_001304367:exon2:c.T214A:p.Y	NOVEL		76	97	0.829734	2	52	126	1.07729	2				
19	chr5	102338812	102338812	T	A	exonic	PAM		nonsynonymous SNV	PAM:NM_138821:exon15:c.T1368A:p.D45	NOVEL		588	0	0	1	174	191	1.07491	1				
19	chr3	173997168	173997168	G	T	exonic	NLGN1		nonsynonymous SNV	NLGN1:NM_001365929:exon5:c.G1377T:p.	NOVEL		804	335	0.912057	1	759	441	1.07111	1				
19	chr3	51929278	51929278	G	C	exonic	IQCF1		synonymous SNV	IQCF1:NM_152397:exon4:c.C246G:p.T82T	NOVEL		482	0	0	1	181	197	1.07055	1				
19	chr22	45289390	45289390	G	A	exonic	PHF21B		nonsynonymous SNV	PHF21B:NM_001242450:exon7:c.C745T:p.	COSMIC	ID=COSM122037	187	157	0.960079	1	159	173	1.07038	1				
19	chr22	32233022	32233022	G	A	exonic	DEPDC5		synonymous SNV	DEPDC5:NM_001242897:exon24:c.G2001A	DBSNP		224	1	0.00934939	1	105	114	1.06928	1				
19	chr7	107580627	107580627	T	A	exonic	LAMB1		nonsynonymous SNV	LAMB1:NM_002291:exon25:c.A3568T:p.I1	NOVEL		488	207	0.923606	1	222	235	1.05629	1				
19	chr12	71978068	71978068	T	G	exonic	LGR5		nonsynonymous SNV	LGR5:NM_001277226:exon17:c.T2206G:p.	NOVEL		485	374	0.915892	1	466	490	1.05286	1				
19	chr9	113088464	113088464	T	C	exonic	TXNDC8		synonymous SNV	TXNDC8:NM_001286946:exon3:c.A186G:p	DBSNP		289	112	0.866845	1	121	127	1.05192	1				
19	chr21	39086687	39086687	G	A	exonic	KCNJ6		nonsynonymous SNV	KCNJ6:NM_002240:exon3:c.C773T:p.T258	COSMIC	ID=COSM121153	159	118	0.896124	1	170	178	1.05068	1				
19	chr8	102656372	102656372	G	C	exonic	GRHL2		nonsynonymous SNV	GRHL2:NM_001330593:exon13:c.G1483C:	NOVEL		154	121	0.92559	1	132	137	1.04616	1				
19	chr8	144922289	144922289	G	A	exonic	NRBP2		nonsynonymous SNV	NRBP2:NM_178564:exon2:c.C242T:p.A81V	COSMIC	ID=COSM488858	117	75	0.821724	1	84	86	1.03916	1				
19	chr13	24874608	24874608	C	T	exonic	SPATA13		nonsynonymous SNV	SPATA13:NM_001286793:exon8:c.C1358T	NOVEL		320	1	0.00655331	1	4	169	1.03636	1				
19	chr11	66638344	66638344	G	C	exonic	PC		nonsynonymous SNV	PC:NM_022172:exon6:c.C653G:p.T218S,PC	NOVEL		576	1	0.00537434	1	388	200	1.03529	1				
19	chr2	170402739	170402739	G	A	exonic	FASTKD1		stopgain	FASTKD1:NM_001322048:exon7:c.C1621T	NOVEL		136	0	0	1	63	64	1.03516	1				
19	chr17	79826287	79826287	C	T	exonic	ARHGDIA		nonsynonymous SNV	ARHGDIA:NM_001301240:exon7:c.G676A:	NOVEL		209	0	0	1	133	68	1.02973	1				
19	chr16	3640986	3640986	G	A	exonic	SLX4		nonsynonymous SNV	SLX4:NM_032444:exon12:c.C2653T:p.P885	NOVEL		238	222	1.01522	1	203	204	1.0296	1				
19	chr19	11150180	11150180	G	A	exonic	SMARCA4		nonsynonymous SNV	SMARCA4:NM_001128849:exon30:c.G421	DBSNP		89	165	1.00721	2	80	80	1.02707	1				
19	chr1	34329918	34329918	T	C	exonic	HMGB4		synonymous SNV	HMGB4:NM_145205:exon2:c.T126C:p.S42	NOVEL		513	0	0	1	243	243	1.02707	1				
19	chr1	228699494	228699494	G	A	exonic	BTNL10		synonymous SNV	BTNL10:NM_001287262:exon2:c.C165T:p.	NOVEL		487	269	0.748508	1	341	1076	1.02617	3				
19	chr19	39423091	39423091	G	A	exonic	MRPS12		synonymous SNV	MRPS12:NM_021107:exon2:c.G168A:p.P5	DBSNP		336	650	1.02213	2	343	341	1.02407	1				
19	chr13	110845205	110845205	G	T	exonic	COL4A1		synonymous SNV	COL4A1:NM_001303110:exon23:c.C1437A	COSMIC	ID=COSM709056	217	1	0.0096496	1	4	104	1.0216	1				
19	chr19	3827602	3827602	G	A	exonic	ZFR2		nonsynonymous SNV	ZFR2:NM_015174:exon6:c.C902T:p.A301V	NOVEL		178	0	0	1	80	79	1.02061	1				
19	chr5	14692990	14692990	G	A	exonic	OTULIN		nonsynonymous SNV	OTULIN:NM_138348:exon7:c.G892A:p.V29	DBSNP		884	227	0.812345	1	560	282	1.01941	1				
19	chr19	44469212	44469212	G	A	exonic	ZNF221		synonymous SNV	ZNF221:NM_001297588:exon3:c.G192A:p	DBSNP		552	213	0.863414	1	239	235	1.01841	1				
19	chr7	44179452	44179452	G	C	exonic	MYL7		nonsynonymous SNV	MYL7:NM_021223:exon5:c.C306G:p.D102	NOVEL		37	20	1.08807	1	48	24	1.01459	1				
19	chr9	130287393	130287393	T	C	exonic	NIBAN2		nonsynonymous SNV	NIBAN2:NM_001035534:exon4:c.A326G:p	DBSNP		549	0	0	1	162	158	1.01423	1				
19	chr13	41239838	41239838	T	C	exonic	FOXO1		nonsynonymous SNV	FOXO1:NM_002015:exon1:c.A512G:p.K17	NOVEL		162	139	0.971436	1	7	146	1.01235	1				
19	chr9	125143979	125143979	C	T	exonic	PTGS1		nonsynonymous SNV	PTGS1:NM_001271164:exon6:c.C571T:p.R	COSMIC	ID=COSM332330	270	104	0.863037	1	130	126	1.01102	1				
19	chr1	115256530	115256530	G	T	exonic	NRAS		nonsynonymous SNV	NRAS:NM_002524:exon3:c.C181A:p.Q61K	DBSNP+COSMIC	ID=COSM580;OC	186	99	0.730729	1	155	148	1.00334	1				
19	chr14	72165704	72165704	T	A	exonic	SIPA1L1		synonymous SNV	SIPA1L1:NM_001284246:exon11:c.T3381A	NOVEL		261	193	0.894267	1	239	227	1.00062	1				
19	chr3	42576538	42576538	C	T	exonic	VIPR1		nonsynonymous SNV	VIPR1:NM_001251883:exon8:c.C452T:p.P1	DBSNP+COSMIC	ID=COSM681997	345	88	0.620476	1	138	131	1.00035	1				
19	chr7	150761819	150761819	C	T	exonic	SLC4A2		stopgain	SLC4A2:NM_001199693:exon3:c.C397T:p.	NOVEL		177	1	0.0174213	1	56	53	0.998804	1				
19	chr3	121354619	121354619	C	T	exonic	HCLS1		synonymous SNV	HCLS1:NM_001292041:exon8:c.G543A:p.P	DBSNP		342	1	0.0090408	1	211	110	0.998769	1				
19	chr15	74703925	74703925	G	A	exonic	SEMA7A		nonsynonymous SNV	SEMA7A:NM_001146029:exon11:c.C1507T	DBSNP		79	39	1.02491	1	80	39	0.997537	1				
19	chr2	169020338	169020338	A	T	exonic	STK39		nonsynonymous SNV	STK39:NM_013233:exon4:c.T483A:p.N161	NOVEL		162	116	0.877767	1	157	147	0.993287	1				
19	chr12	26731717	26731717	T	C	exonic	ITPR2		nonsynonymous SNV	ITPR2:NM_002223:exon34:c.A4559G:p.Y1	NOVEL		215	138	0.822376	1	186	173	0.989881	1				
19	chr7	21778460	21778460	G	A	exonic	DNAH11		nonsynonymous SNV	DNAH11:NM_001277115:exon47:c.G7787	DBSNP+COSMIC	ID=COSM664621	353	150	0.92475	1	300	143	0.982525	1				
19	chr11	64622151	64622151	C	G	exonic	EHD1		nonsynonymous SNV	EHD1:NM_006795:exon5:c.G1259C:p.G42	NOVEL		754	0	0	1	538	256	0.981366	1				
19	chr5	90046460	90046460	C	T	exonic	ADGRV1		synonymous SNV	ADGRV1:NM_032119:exon53:c.C11067T:p	NOVEL		835	0	0	1	272	248	0.979669	1				
19	chr5	71495394	71495394	A	G	exonic	MAP1B		nonsynonymous SNV	MAP1B:NM_001324255:exon4:c.A5834G:	NOVEL		1243	22	0.0648565	1	367	333	0.977186	1				
19	chr12	130648815	130648815	G	C	exonic	FZD10		nonsynonymous SNV	FZD10:NM_007197:exon1:c.G1328C:p.R44	DBSNP		419	0	0	1	212	192	0.976227	1				
19	chr18	39647472	39647472	G	A	exonic	PIK3C3		nonsynonymous SNV	PIK3C3:NM_001308020:exon23:c.G2455A	NOVEL		142	0	0	1	74	67	0.976083	1				
19	chr5	137217684	137217684	G	A	exonic	MYOT		nonsynonymous SNV	MYOT:NM_001135940:exon6:c.G154A:p.D	DBSNP		1233	351	0.826368	1	507	457	0.973801	1				
19	chr1	169512016	169512016	A	C	exonic	F5		nonsynonymous SNV	F5:NM_000130:exon13:c.T2312G:p.I771R	NOVEL		791	1	0.00265608	1	1074	339	0.972651	1				
19	chr4	126370137	126370137	G	A	exonic	FAT4		nonsynonymous SNV	FAT4:NM_001291285:exon9:c.G7972A:p.D	NOVEL		330	249	0.904663	1	305	274	0.972082	1				
19	chr14	38724159	38724159	T	A	exonic	CLEC14A		nonsynonymous SNV	CLEC14A:NM_175060:exon1:c.A1069T:p.T	NOVEL		252	186	0.893315	1	244	219	0.971615	1				
19	chr6	109274386	109274386	C	T	exonic	ARMC2		nonsynonymous SNV	ARMC2:NM_001286609:exon12:c.C1252T:	NOVEL		362	186	1.05253	1	363	169	0.970208	1				
19	chr1	34090799	34090799	G	T	exonic	CSMD2		synonymous SNV	CSMD2:NM_001281956:exon34:c.C5352A:	NOVEL		216	131	0.794159	1	152	136	0.970013	1				
19	chr1	55119799	55119799	G	A	exonic	MROH7		synonymous SNV	MROH7:NM_001039464:exon3:c.G1200A:	NOVEL		130	0	0	1	75	67	0.969209	1				
19	chr1	115829289	115829289	G	A	exonic	NGF		nonsynonymous SNV	NGF:NM_002506:exon3:c.C128T:p.S43F	DBSNP+COSMIC	ID=COSM276334	531	1	0.00395416	1	281	248	0.963002	1				
19	chr3	192980977	192980977	G	A	exonic	PLAAT1		nonsynonymous SNV	PLAAT1:NM_020386:exon3:c.G358A:p.E12	NOVEL		479	197	0.903692	1	450	221	0.959947	1				
19	chr15	91017869	91017869	C	T	exonic	IQGAP1		nonsynonymous SNV	IQGAP1:NM_003870:exon23:c.C2728T:p.L	NOVEL		226	87	0.861938	1	208	95	0.954316	1				
19	chr15	55731757	55731757	G	A	exonic	DNAAF4		nonsynonymous SNV	DNAAF4:NM_001033559:exon7:c.C806T:p	NOVEL		495	1	0.00625201	1	344	157	0.953835	1				
19	chr3	48610764	48610764	T	A	exonic	COL7A1		synonymous SNV	COL7A1:NM_000094:exon83:c.A6636T:p.T	NOVEL		337	6	0.0534057	1	136	117	0.949941	1				
19	chr22	46096162	46096162	C	G	exonic	ATXN10		nonsynonymous SNV	ATXN10:NM_001167621:exon3:c.C200G:p	NOVEL		132	0	0	1	70	60	0.948067	1				
19	chr2	108863714	108863714	G	A	exonic	SULT1C3		nonsynonymous SNV	SULT1C3:NM_001008743:exon2:c.G64A:p	DBSNP		610	0	0	1	337	287	0.944775	1				
19	chr17	38936690	38936690	C	T	exonic	KRT27		synonymous SNV	KRT27:NM_181537:exon3:c.G546A:p.A182	DBSNP		342	131	0.858838	1	367	165	0.944025	1				
19	chr2	63832512	63832512	G	C	exonic	MDH1		nonsynonymous SNV	MDH1:NM_001199112:exon6:c.G507C:p.W	NOVEL		302	250	0.952723	1	322	273	0.94249	1				
19	chr8	110457745	110457745	C	T	exonic	PKHD1L1		nonsynonymous SNV	PKHD1L1:NM_177531:exon38:c.C5647T:p.	DBSNP+COSMIC	ID=COSM450038	259	226	0.98024	1	293	248	0.941641	1				
19	chr14	103969408	103969408	C	A	exonic	MARK3		nonsynonymous SNV	MARK3:NM_001128920:exon16:c.C1986A	NOVEL		397	0	0	1	234	197	0.938902	1				
19	chr5	26885856	26885856	T	C	exonic	CDH9		synonymous SNV	CDH9:NM_016279:exon11:c.A1749G:p.T58	DBSNP		297	339	1.0596	2	320	141	0.930957	1				
19	chr9	101470769	101470769	G	T	exonic	GABBR2		nonsynonymous SNV	GABBR2:NM_005458:exon1:c.C251A:p.A8	NOVEL		195	0	0	1	64	53	0.93051	1				
19	chr12	110399151	110399151	G	A	exonic	GIT2		synonymous SNV	GIT2:NM_001135214:exon11:c.C919T:p.L3	DBSNP		331	0	0	1	152	125	0.926961	1				
19	chr2	217347645	217347645	A	C	exonic	SMARCAL1		nonsynonymous SNV	SMARCAL1:NM_001127207:exon18:c.A28	NOVEL		369	8	0.044639	1	197	162	0.92694	1				
19	chr20	60988531	60988531	G	C	exonic	RBBP8NL		nonsynonymous SNV	RBBP8NL:NM_080833:exon11:c.C1601G:p	NOVEL		53	120	1.02648	2	72	111	0.92311	2				
19	chr1	248570180	248570180	G	A	exonic	OR2T1		nonsynonymous SNV	OR2T1:NM_030904:exon1:c.G885A:p.M29	DBSNP		231	0	0	1	309	90	0.914469	1				
19	chr7	6662388	6662388	G	C	exonic	ZNF853		nonsynonymous SNV	ZNF853:NM_001353546:exon3:c.G1721C:	NOVEL		495	1	0.00625201	1	335	143	0.910583	1				
19	chr19	30500147	30500147	G	A	exonic	URI1		nonsynonymous SNV	URI1:NM_001252641:exon8:c.G868A:p.D2	NOVEL		404	704	0.985154	2	466	371	0.910499	1				
19	chr19	9016694	9016694	C	T	exonic	MUC16		synonymous SNV	MUC16:NM_024690:exon28:c.G38043A:p.	NOVEL		432	0	0	1	159	124	0.900049	1				
19	chr9	79896801	79896801	G	A	exonic	VPS13A		nonsynonymous SNV	VPS13A:NM_001018037:exon28:c.G2923A	DBSNP+COSMIC	ID=COSM111024	283	94	0.773845	1	134	104	0.89761	1				
19	chr15	85643413	85643413	G	A	exonic	PDE8A		synonymous SNV	PDE8A:NM_173454:exon10:c.G882A:p.Q29	NOVEL		549	0	0	1	379	158	0.895559	1				
19	chr6	135754295	135754295	T	A	exonic	AHI1		synonymous SNV	AHI1:NM_001134830:exon14:c.A2136T:p.	NOVEL		764	0	0	1	514	210	0.885871	1				
19	chr3	77147210	77147210	A	T	exonic	ROBO2		nonsynonymous SNV	ROBO2:NM_001290039:exon2:c.A107T:p.	NOVEL		456	3	0.0199544	1	172	128	0.876435	1				
19	chr1	173010512	173010512	A	G	exonic	TNFSF18		nonsynonymous SNV	TNFSF18:NM_005092:exon3:c.T595C:p.S1	NOVEL		166	0	0	1	201	55	0.871008	1				
19	chr1	1334608	1334608	C	G	exonic	CCNL2		nonsynonymous SNV	CCNL2:NM_001039577:exon1:c.G79C:p.G2	NOVEL		278	1	0.00753983	1	154	113	0.869357	1				
19	chr15	28408419	28408419	G	A	exonic	HERC2		stopgain	HERC2:NM_004667:exon69:c.C10567T:p.Q	NOVEL		607	0	0	1	441	176	0.868238	1				
19	chr1	181686394	181686394	C	T	exonic	CACNA1E		nonsynonymous SNV	CACNA1E:NM_000721:exon11:c.C1481T:p	NOVEL		132	0	0	1	166	45	0.864628	1				
19	chr19	47524393	47524393	C	G	exonic	NPAS1		nonsynonymous SNV	NPAS1:NM_002517:exon2:c.C89G:p.P30R	NOVEL		248	1	0.0124538	1	111	80	0.860375	1				
19	chr12	5153677	5153677	G	C	exonic	KCNA5		nonsynonymous SNV	KCNA5:NM_002234:exon1:c.G364C:p.V122	NOVEL		169	89	0.725665	1	152	104	0.834496	1				
19	chr10	97388241	97388241	C	T	exonic	ALDH18A1		nonsynonymous SNV	ALDH18A1:NM_001323419:exon5:c.G181A	NOVEL		239	0	0	1	123	90	0.771807	1				
19	chr5	16704689	16704689	G	T	exonic	MYO10		synonymous SNV	MYO10:NM_012334:exon22:c.C2275A:p.R	NOVEL		741	1	0.00535828	1	378	128	0.769965	1				
19	chr10	50534114	50534114	C	A	exonic	C10orf71		nonsynonymous SNV	C10orf71:NM_001135196:exon3:c.C3524A	NOVEL		273	191	0.865927	1	214	154	0.764396	1				
19	chr10	61833293	61833293	T	A	exonic	ANK3		nonsynonymous SNV	ANK3:NM_020987:exon37:c.A7346T:p.H24	NOVEL		248	0	0	1	167	118	0.75628	1				
19	chr17	72926478	72926478	T	C	exonic	OTOP2		nonsynonymous SNV	OTOP2:NM_178160:exon6:c.T748C:p.Y250	NOVEL		464	0	0	1	358	116	0.744888	1				
19	chr3	195475883	195475883	G	A	exonic	MUC4		synonymous SNV	MUC4:NM_138297:exon22:c.C3063T:p.Y10	DBSNP		171	52	0.723102	1	152	46	0.677127	1				
19	chr7	100366242	100366242	A	G	exonic	ZAN		unknown	UNKNOWN	NOVEL		675	0	0	1	240	117	0.673207	1				
19	chr14	20344722	20344722	C	G	exonic	OR4K2		nonsynonymous SNV	OR4K2:NM_001005501:exon1:c.C296G:p.T	NOVEL		1678	2	0.0025043	1	1213	382	0.491965	1				
19	chr2	224856589	224856589	T	A	exonic	SERPINE2		nonsynonymous SNV	SERPINE2:NM_001136528:exon4:c.A616T:	NOVEL		220	1	0.00951861	1	198	27	0.246497	1				
19	chr6	168352399	168352399	C	T	exonic	AFDN		synonymous SNV	AFDN:NM_001207008:exon28:c.C4293T:p	DBSNP+COSMIC	ID=COSM671347	677	0	0	1	587	48	0.23008	1				
19	chr17	79519341	79519341	G	A	exonic	FAAP100		nonsynonymous SNV	FAAP100:NM_025161:exon1:c.C16T:p.P6S	NOVEL		276	0	0	1	249	18	0.205198	1				
19	chr16	69419309	69419309	C	G	exonic	TERF2		nonsynonymous SNV	TERF2:NM_005652:exon2:c.G460C:p.E154	NOVEL		777	0	0	1	652	70	0.199155	1				
19	chr16	57760823	57760823	C	T	exonic	DRC7		nonsynonymous SNV	DRC7:NM_001289163:exon13:c.C1859T:p.	NOVEL		336	1	0.00624218	1	309	31	0.18729	1				
19	chr4	123277092	123277092	A	T	exonic	KIAA1109		nonsynonymous SNV	KIAA1109:NM_015312:exon81:c.A14447T:	NOVEL		699	0	0	1	712	64	0.169414	1				

19	chr6	64423470	64423470	C	T	exonic	PHF3		nonsynonymous SNV	PHF3:NM_001370349:exon15:c.C5722T:p.	NOVEL		1174	1	0.00263914	1	951	55	0.166976	1				
19	chr1	186063442	186063442	T	C	exonic	HMCN1		nonsynonymous SNV	HMCN1:NM_031935:exon67:c.T10231C:p.	NOVEL		435	0	0	1	741	30	0.157749	1				
19	chr9	125852565	125852565	G	T	exonic	RABGAP1		nonsynonymous SNV	RABGAP1:NM_012197:exon21:c.G2529T:p	NOVEL		467	0	0	1	267	22	0.156371	1				
19	chr15	31218129	31218129	C	T	exonic	FAN1		synonymous SNV	FAN1:NM_014967:exon10:c.C2475T:p.S82	NOVEL		380	1	0.00813909	1	385	18	0.13595	1				
19	chr11	18741822	18741822	A	T	exonic	IGSF22		nonsynonymous SNV	IGSF22:NM_173588:exon5:c.T389A:p.L130	NOVEL		174	0	0	1	175	7	0.117068	1				
19	chr4	68606341	68606341	G	T	exonic	GNRHR		nonsynonymous SNV	GNRHR:NM_000406:exon3:c.C844A:p.P28	COSMIC	ID=COSM736967	716	1	0.00293391	1	638	32	0.0981084	1				
19	chr5	65470845	65470845	C	A	exonic	SREK1		nonsynonymous SNV	SREK1:NM_001077199:exon10:c.C1556A:p	COSMIC	ID=COSM106945	430	105	0.731909	1	302	4	0.0268516	1				
19	chr19	8842179	8842179	C	T	exonic	OR2Z1		synonymous SNV	OR2Z1:NM_001004699:exon1:c.C789T:p.C	DBSNP+COSMIC	ID=COSM139782	475	170	0.817317	1	450	3	0.0136036	1				
19	chr18	44266233	44266233	C	A	exonic	ST8SIA5		nonsynonymous SNV	ST8SIA5:NM_001307987:exon4:c.G380T:p	NOVEL		178	7	0.0795962	1	205	1	0.00997158	1				
19	chr4	6611707	6611707	A	G	exonic	MAN2B2		nonsynonymous SNV	MAN2B2:NM_001292038:exon13:c.A2036	NOVEL		377	26	0.135717	1	414	2	0.0098757	1				
19	chr11	5080475	5080475	A	T	exonic	OR52E2		nonsynonymous SNV	OR52E2:NM_001005164:exon1:c.T383A:p.	NOVEL		419	18	0.12773	1	414	1	0.00733438	1				
19	chr5	147830784	147830784	T	C	exonic	HTR4		nonsynonymous SNV	HTR4:NM_001040169:exon6:c.A1128G:p.I	NOVEL		461	28	0.213536	1	284	1	0.00720753	1				
19	chr11	114577278	114577278	G	A	exonic	NXPE2		nonsynonymous SNV	NXPE2:NM_182495:exon6:c.G1306A:p.D43	NOVEL		928	34	0.109599	1	1029	2	0.00590449	1				
19	chr3	33155764	33155764	C	A	exonic	CRTAP		synonymous SNV	CRTAP:NM_006371:exon1:c.C195A:p.G65	NOVEL		175	65	0.826861	1	166	0	0	1				
19	chr14	102498744	102498744	C	G	exonic	DYNC1H1		nonsynonymous SNV	DYNC1H1:NM_001376:exon52:c.C10019G:	NOVEL		193	42	0.375965	1	258	0	0	1				
19	chr7	120971936	120971936	A	T	exonic	WNT16		nonsynonymous SNV	WNT16:NM_016087:exon3:c.A521T:p.D17	NOVEL		604	54	0.254489	1	480	0	0	1				
19	chr3	88202546	88202546	A	T	exonic	C3orf38		nonsynonymous SNV	C3orf38:NM_173824:exon2:c.A300T:p.Q10	NOVEL		358	29	0.22878	1	294	0	0	1				
19	chr11	77884845	77884845	C	T	exonic	KCTD21		synonymous SNV	KCTD21:NM_001029859:exon2:c.G756A:p.	NOVEL		503	40	0.228434	1	521	0	0	1				
19	chr8	99019805	99019805	C	T	exonic	MATN2		nonsynonymous SNV	MATN2:NM_001317748:exon9:c.C1426T:p	DBSNP+COSMIC	ID=COSM279243	143	14	0.187583	1	161	0	0	1				
19	chr1	233136175	233136175	C	A	exonic	PCNX2		nonsynonymous SNV	PCNX2:NM_014801:exon30:c.G5204T:p.W	NOVEL		329	31	0.181144	1	669	0	0	1				
19	chr17	39767417	39767417	G	A	exonic	KRT16		synonymous SNV	KRT16:NM_005557:exon4:c.C837T:p.G279	DBSNP+COSMIC	ID=COSM406640	341	21	0.179892	1	383	0	0	1				
19	chr16	20638622	20638622	G	A	exonic	ACSM1		nonsynonymous SNV	ACSM1:NM_052956:exon10:c.C1316T:p.T4	DBSNP		150	14	0.179577	1	151	0	0	1				
19	chr19	39398094	39398094	A	G	exonic	NFKBIB		nonsynonymous SNV	NFKBIB:NM_001243116:exon5:c.A506G:p.	NOVEL		323	18	0.163689	1	255	0	0	1				
19	chr9	124975914	124975914	C	A	exonic	LHX6		nonsynonymous SNV	LHX6:NM_001242335:exon5:c.G377T:p.R1	NOVEL		237	12	0.149572	1	162	0	0	1				
19	chr19	4013254	4013254	C	T	exonic	PIAS4		nonsynonymous SNV	PIAS4:NM_015897:exon2:c.C361T:p.R121	NOVEL		473	23	0.143796	1	336	0	0	1				
19	chr19	56538535	56538535	G	A	exonic	NLRP5		nonsynonymous SNV	NLRP5:NM_153447:exon7:c.G936A:p.M31	DBSNP		1266	55	0.12911	1	899	0	0	1				
19	chr12	48723477	48723477	G	A	exonic	H1FNT		nonsynonymous SNV	H1FNT:NM_181788:exon1:c.G403A:p.A13	COSMIC	ID=COSM404222	123	8	0.128465	1	103	0	0	1				
19	chr3	52324457	52324457	C	T	exonic	GLYCTK		synonymous SNV	GLYCTK:NM_001144951:exon2:c.C99T:p.A	NOVEL		287	11	0.112696	1	178	0	0	1				
19	chr17	61466689	61466689	G	T	exonic	TANC2		nonsynonymous SNV	TANC2:NM_025185:exon16:c.G2613T:p.L8	NOVEL		324	12	0.11075	1	345	0	0	1				
19	chr22	17619173	17619173	G	A	exonic	HDHD5		nonsynonymous SNV	HDHD5:NM_017829:exon8:c.C920T:p.A307	DBSNP		229	11	0.0964156	1	264	0	0	1				
19	chr1	158906908	158906908	A	C	exonic	PYHIN1		nonsynonymous SNV	PYHIN1:NM_152501:exon2:c.A208C:p.K70	NOVEL		331	14	0.085364	1	638	0	0	1				
19	chr17	59763491	59763491	G	A	exonic	BRIP1		nonsynonymous SNV	BRIP1:NM_032043:exon19:c.C2611T:p.H87	DBSNP		869	23	0.0799584	1	1018	0	0	1				
19	chr12	128899431	128899431	G	A	exonic	TMEM132C		synonymous SNV	TMEM132C:NM_001136103:exon2:c.G240	NOVEL		227	8	0.0716124	1	198	0	0	1				
19	chr7	66453370	66453370	C	T	exonic	SBDS		synonymous SNV	SBDS:NM_016038:exon5:c.G741A:p.E247E	DBSNP		831	18	0.0657455	1	885	0	0	1				
20	chr17	74133670	74133670	C	A	exonic	FOXJ1		nonsynonymous SNV	FOXJ1:NM_001454:exon3:c.G1030T:p.V34	NOVEL		108	108	1.14215	1	92	109	1.1013	1	114	146	1.14013	1
20	chr17	73124953	73124953	G	A	exonic	ARMC7		synonymous SNV	ARMC7:NM_024585:exon3:c.G417A:p.T13	DBSNP		389	0	0	1	168	195	1.09094	1	235	249	1.04455	1
20	chr12	96180827	96180827	A	G	exonic	NTN4		nonsynonymous SNV	NTN4:NM_001329700:exon2:c.T475C:p.Y1	NOVEL		626	2	0.00727483	1	307	354	1.08762	1	370	358	0.998455	1
20	chr1	27939528	27939528	C	T	exonic	FGR		nonsynonymous SNV	FGR:NM_001042729:exon13:c.G1487A:p.R	DBSNP+COSMIC	ID=COSM134153	157	138	1.06859	1	149	170	1.08226	1	169	168	1.01218	1
20	chr19	50157916	50157916	G	A	exonic	SCAF1		nonsynonymous SNV	SCAF1:NM_021228:exon9:c.G3407A:p.S11	NOVEL		418	1	0.00545178	1	228	260	1.082	1	258	277	1.05124	1
20	chr16	88691044	88691044	G	A	exonic	ZC3H18		nonsynonymous SNV	ZC3H18:NM_144604:exon12:c.G1933A:p.A	NOVEL		196	1	0.0115954	1	93	106	1.08175	1	113	108	0.992221	1
20	chr11	93104330	93104330	T	C	exonic	DEUP1		nonsynonymous SNV	DEUP1:NM_181645:exon7:c.T673C:p.F225	DBSNP+COSMIC	ID=COSM495463	138	136	1.13381	1	135	153	1.07888	1	182	199	1.06049	1
20	chr7	100731749	100731749	A	C	exonic	TRIM56		nonsynonymous SNV	TRIM56:NM_030961:exon3:c.A1156C:p.T3	NOVEL		177	0	0	1	83	94	1.07852	1	94	128	1.17067	1
20	chr12	18552664	18552664	C	G	exonic	PIK3C2G		nonsynonymous SNV	PIK3C2G:NM_004570:exon15:c.C2075G:p.	COSMIC	ID=COSM565666	465	1	0.00490192	1	240	270	1.07515	1	277	311	1.07389	1
20	chr2	43452737	43452737	G	C	exonic	ZFP36L2		nonsynonymous SNV	ZFP36L2:NM_006887:exon2:c.C206G:p.P6	NOVEL		207	0	0	1	94	105	1.07155	1	121	128	1.04373	1
20	chr17	79914549	79914549	T	C	exonic	NOTUM		nonsynonymous SNV	NOTUM:NM_178493:exon8:c.A965G:p.Y3	NOVEL		502	1	0.00454134	1	201	224	1.07037	1	258	261	1.02106	1
20	chr5	178359125	178359125	G	A	exonic	ZFP2		nonsynonymous SNV	ZFP2:NM_030613:exon5:c.G811A:p.E271K	NOVEL		389	27	0.14826	1	181	201	1.06858	1	275	243	0.952474	1
20	chr19	1122143	1122143	C	G	exonic	SBNO2		nonsynonymous SNV	SBNO2:NM_001100122:exon8:c.G973C:p.	NOVEL		336	1	0.00677833	1	164	182	1.06824	1	217	191	0.950495	1
20	chr20	37591020	37591020	C	G	exonic	DHX35		nonsynonymous SNV	DHX35:NM_001190809:exon1:c.C10G:p.P4	DBSNP		272	231	1.04905	1	212	233	1.06334	1	316	260	0.91649	1
20	chr1	226924758	226924758	C	T	exonic	ITPKB		synonymous SNV	ITPKB:NM_002221:exon2:c.G402A:p.L134	COSMIC	ID=COSM414342	256	519	1.09971	2	237	548	1.0579	2	331	640	0.998682	2
20	chr19	47195006	47195006	C	A	exonic	PRKD2		nonsynonymous SNV	PRKD2:NM_001079882:exon11:c.G1217T:	NOVEL		20	26	1.29112	1	24	26	1.05603	1	33	24	0.854896	1
20	chr20	61512112	61512112	G	A	exonic	DIDO1		synonymous SNV	DIDO1:NM_001193369:exon16:c.C5196T:p	NOVEL		255	220	1.05799	1	231	250	1.05553	1	257	265	1.03075	1
20	chr1	197072186	197072186	G	C	exonic	ASPM		synonymous SNV	ASPM:NM_018136:exon18:c.C6195G:p.T20	NOVEL		391	180	1.03533	1	421	224	1.05257	1	544	232	0.905989	1
20	chr2	1906895	1906895	G	A	exonic	MYT1L		synonymous SNV	MYT1L:NM_001303052:exon14:c.C1989T:p	NOVEL		321	0	0	1	163	175	1.05147	1	197	185	0.983298	1
20	chr2	131674211	131674211	C	T	exonic	ARHGEF4		nonsynonymous SNV	ARHGEF4:NM_001367493:exon2:c.C2692T	NOVEL		369	0	0	1	187	199	1.04699	1	182	185	1.02349	1
20	chr3	169644818	169644818	G	A	exonic	SAMD7		synonymous SNV	SAMD7:NM_001304366:exon6:c.G768A:p.	DBSNP		637	0	0	1	328	349	1.04692	1	414	425	1.0285	1
20	chr22	50899993	50899993	G	A	exonic	SBF1		nonsynonymous SNV	SBF1:NM_001365819:exon22:c.C2801T:p.	COSMIC	ID=COSM573475	212	35	0.323686	1	130	138	1.04573	1	136	144	1.04419	1
20	chr11	93803610	93803610	A	G	exonic	HEPHL1		synonymous SNV	HEPHL1:NM_001098672:exon6:c.A1134G:	NOVEL		342	0	0	1	154	161	1.03798	1	190	202	1.04627	1
20	chr15	79237294	79237294	G	A	exonic	CTSH		synonymous SNV	CTSH:NM_004390:exon1:c.C30T:p.A10A	DBSNP		107	82	0.99107	1	94	98	1.03657	1	96	90	0.982441	1
20	chr16	3065397	3065397	C	G	exonic	CLDN6		nonsynonymous SNV	CLDN6:NM_021195:exon2:c.G626C:p.R209	NOVEL		250	0	0	1	131	136	1.03443	1	169	180	1.04719	1
20	chr6	46609963	46609963	C	G	exonic	CYP39A1		nonsynonymous SNV	CYP39A1:NM_001278738:exon2:c.G250C:	NOVEL		222	17	0.162481	1	119	123	1.0322	1	153	131	0.936547	1
20	chr17	42931994	42931994	C	T	exonic	EFTUD2		nonsynonymous SNV	EFTUD2:NM_001142605:exon21:c.G2084A	COSMIC	ID=COSM319157	276	34	0.250536	1	144	148	1.02933	1	190	171	0.961758	1
20	chr15	101528892	101528892	C	T	exonic	LRRK1		stopgain	LRRK1:NM_024652:exon5:c.C487T:p.R163	DBSNP+COSMIC	ID=COSM220041	175	193	1.19801	1	195	198	1.02317	1	236	191	0.908202	1
20	chr19	10254551	10254551	C	T	exonic	DNMT1		nonsynonymous SNV	DNMT1:NM_001318730:exon28:c.G2959A	NOVEL		311	0	0	1	126	127	1.01943	1	176	143	0.910169	1
20	chr4	140196466	140196466	T	C	exonic	MGARP		nonsynonymous SNV	MGARP:NM_032623:exon2:c.A163G:p.T55	DBSNP		450	0	0	1	258	258	1.01542	1	306	264	0.940385	1
20	chr3	10974899	10974899	C	T	exonic	SLC6A11		synonymous SNV	SLC6A11:NM_014229:exon11:c.C1434T:p.	DBSNP+COSMIC	ID=COSM103618	108	94	1.06299	1	96	95	1.0101	1	159	125	0.893652	1
20	chr6	394972	394972	A	G	exonic	IRF4		nonsynonymous SNV	IRF4:NM_001195286:exon3:c.A368G:p.K1	COSMIC	ID=COSM329431	381	1	0.00597983	1	215	210	1.00347	1	258	217	0.927562	1
20	chr18	61449709	61449709	C	T	exonic	SERPINB7		nonsynonymous SNV	SERPINB7:NM_001040147:exon2:c.C103T:	NOVEL		248	1	0.00917388	1	119	116	1.00246	1	148	156	1.0419	1
20	chr12	69759575	69759575	A	G	exonic	YEATS4		nonsynonymous SNV	YEATS4:NM_006530:exon4:c.A244G:p.T82	NOVEL		222	1	0.0102435	1	123	119	0.998634	1	113	130	1.08621	1
20	chr3	31712406	31712406	A	G	exonic	OSBPL10		nonsynonymous SNV	OSBPL10:NM_001174060:exon8:c.T1604C	NOVEL		216	158	0.965024	1	181	175	0.998304	1	220	224	1.02433	1
20	chr6	80721463	80721463	A	T	exonic	TTK		nonsynonymous SNV	TTK:NM_001166691:exon7:c.A746T:p.Q24	NOVEL		263	0	0	1	123	118	0.994351	1	184	157	0.934807	1
20	chr12	2997095	2997095	A	G	exonic	RHNO1		nonsynonymous SNV	RHNO1:NM_001252499:exon3:c.A187G:p.	NOVEL		88	102	1.22631	1	105	100	0.990652	1	117	122	1.03643	1
20	chr5	127626531	127626531	C	T	exonic	FBN2		nonsynonymous SNV	FBN2:NM_001999:exon50:c.G6338A:p.C2	NOVEL		343	320	1.10253	1	331	315	0.990268	1	424	359	0.930914	1
20	chr12	72288148	72288148	A	G	exonic	TBC1D15		nonsynonymous SNV	TBC1D15:NM_022771:exon7:c.A701G:p.H	DBSNP		328	216	0.907	1	278	263	0.987264	1	351	331	0.985418	1
20	chr1	19484433	19484433	C	G	exonic	UBR4		nonsynonymous SNV	UBR4:NM_020765:exon40:c.G5636C:p.G1	NOVEL		242	211	1.06399	1	198	187	0.986406	1	283	227	0.903717	1
20	chr1	152128689	152128689	C	T	exonic	RPTN		nonsynonymous SNV	RPTN:NM_001122965:exon3:c.G886A:p.G	DBSNP+COSMIC	ID=COSM149849	328	507	0.997089	2	338	629	0.985727	2	397	736	0.984271	2
20	chr20	61440951	61440951	G	C	exonic	OGFR		nonsynonymous SNV	OGFR:NM_007346:exon4:c.G334C:p.D112	NOVEL		469	0	0	1	236	222	0.984379	1	285	274	0.995212	1
20	chr2	179616655	179616655	C	G	exonic	TTN		nonsynonymous SNV	TTN:NM_133379:exon46:c.G10472C:p.R34	NOVEL		634	2	0.00718332	1	308	289	0.983101	1	358	314	0.948718	1
20	chr17	73566518	73566518	G	A	exonic	LLGL2		nonsynonymous SNV	LLGL2:NM_001031803:exon16:c.G1964A:p	NOVEL		162	1	0.0140141	1	83	77	0.97734	1	112	105	0.982441	1
20	chr17	76472667	76472667	T	C	exonic	DNAH17		nonsynonymous SNV	DNAH17:NM_173628:exon52:c.A8141G:p.	NOVEL		140	123	1.06832	1	148	137	0.976226	1	148	141	0.990599	1
20	chr11	46897436	46897436	G	A	exonic	LRP4		synonymous SNV	LRP4:NM_002334:exon26:c.C3618T:p.I120	NOVEL		470	0	0	1	247	227	0.972573	1	295	275	0.979568	1
20	chr1	74937388	74937388	A	C	exonic	LRRC53		nonsynonymous SNV	LRRC53:NM_001364665:exon4:c.T1918G:	NOVEL		230	20	0.182744	1	133	122	0.971616	1	155	128	0.918333	1
20	chr3	151056316	151056316	G	T	exonic	P2RY12		nonsynonymous SNV	P2RY12:NM_176876:exon2:c.C318A:p.F10	NOVEL		868	0	0	1	416	381	0.970826	1	551	488	0.953632	1
20	chr10	97373840	97373840	G	T	exonic	ALDH18A1		nonsynonymous SNV	ALDH18A1:NM_001323419:exon11:c.C104	NOVEL		365	1	0.00624125	1	189	173	0.970538	1	234	221	0.986183	1
20	chr7	87079401	87079401	G	A	exonic	ABCB4		nonsynonymous SNV	ABCB4:NM_000443:exon8:c.C716T:p.S239	DBSNP		275	248	1.08318	1	271	248	0.970419	1	308	320	1.03459	1
20	chr5	149425203	149425203	G	C	exonic	HMGXB3		nonsynonymous SNV	HMGXB3:NM_001366501:exon15:c.G2407	NOVEL		149	149	1.14215	1	157	143	0.968032	1	180	174	0.997982	1
20	chr5	180219149	180219149	G	A	exonic	MGAT1		nonsynonymous SNV	MGAT1:NM_001114619:exon2:c.C823T:p.	NOVEL		237	218	1.09445	1	236	214	0.965775	1	267	247	0.975687	1
20	chr22	37333522	37333522	G	C	exonic	CSF2RB		nonsynonymous SNV	CSF2RB:NM_000395:exon14:c.G1672C:p.A	NOVEL		361	1	0.00631021	1	178	161	0.964497	1	207	234	1.07734	1
20	chr21	47841912	47841912	G	A	exonic	PCNT		synonymous SNV	PCNT:NM_001315529:exon32:c.G6699A:p.	NOVEL		293	31	0.218559	1	176	159	0.963889	1	210	176	0.925768	1
20	chr7	150390156	150390156	G	A	exonic	GIMAP2		nonsynonymous SNV	GIMAP2:NM_015660:exon3:c.G782A:p.C2	COSMIC	ID=COSM667100	404	0	0	1	244	220	0.962896	1	243	222	0.969341	1
20	chr19	58565137	58565137	C	T	exonic	ZSCAN1		synonymous SNV	ZSCAN1:NM_182572:exon6:c.C945T:p.R31	DBSNP		291	217	0.975772	1	279	251	0.961773	1	288	304	1.04263	1
20	chr2	198511240	198511240	A	T	exonic	RFTN2		nonsynonymous SNV	RFTN2:NM_144629:exon2:c.T290A:p.L97	NOVEL		295	0	0	1	149	133	0.957806	1	184	167	0.96602	1
20	chr5	140187569	140187569	C	T	exonic	PCDHA4		nonsynonymous SNV	PCDHA4:NM_018907:exon1:c.C797T:p.A26	NOVEL		263	231	1.06816	1	301	268	0.956527	1	354	326	0.973387	1
20	chr4	73957192	73957192	C	T	exonic	ANKRD17		synonymous SNV	ANKRD17:NM_198889:exon28:c.G5400A:p	NOVEL		435	0	0	1	229	203	0.954305	1	281	286	1.02414	1
20	chr6	80721475	80721475	T	C	exonic	TTK		nonsynonymous SNV	TTK:NM_001166691:exon7:c.T758C:p.I253	DBSNP		279	0	0	1	132	117	0.954248	1	190	168	0.952803	1
20	chr9	127253418	127253418	C	T	exonic	NR5A1		synonymous SNV	NR5A1:NM_004959:exon6:c.G1080A:p.A3	DBSNP		76	56	0.969095	1	52	46	0.95325	1	60	53	0.952301	1
20	chr2	26029186	26029186	C	T	exonic	ASXL2		nonsynonymous SNV	ASXL2:NM_018263:exon3:c.G164A:p.C55Y	NOVEL		803	1	0.00284116	1	432	381	0.95172	1	508	445	0.948077	1
20	chr5	140209768	140209768	G	A	exonic	PCDHA6		nonsynonymous SNV	PCDHA6:NM_018909:exon1:c.G2092A:p.V	COSMIC	ID=COSM317822	113	94	1.03731	1	90	79	0.949326	1	111	100	0.962264	1
20	chr4	70799938	70799938	T	C	exonic	CSN1S1		synonymous SNV	CSN1S1:NM_001025104:exon3:c.T60C:p.P	NOVEL		187	151	1.0205	1	179	156	0.945703	1	191	210	1.06329	1
20	chr12	91572243	91572243	T	C	exonic	DCN		synonymous SNV	DCN:NM_133504:exon1:c.A87G:p.L29L,DC	NOVEL		297	238	1.01619	1	259	225	0.944087	1	296	299	1.02031	1
20	chr4	88533648	88533648	G	C	exonic	DSPP		nonsynonymous SNV	DSPP:NM_014208:exon4:c.G310C:p.G104	NOVEL		770	3	0.00886532	1	380	328	0.940839	1	470	398	0.930979	1
20	chrX	70756147	70756147	G	C	exonic	OGT		nonsynonymous SNV	OGT:NM_181672:exon2:c.G157C:p.D53H,C	NOVEL		197	141	0.952916	1	169	145	0.937806	1	184	198	1.05239	1
20	chr12	25378562	25378562	C	G	exonic	KRAS		nonsynonymous SNV	KRAS:NM_001369786:exon4:c.G436C:p.A1	DBSNP+COSMIC	ID=COSM19905,	108	66	0.866457	1	77	66	0.937309	1	115	102	0.954371	1
20	chrX	100808404	100808404	G	A	exonic	ARMCX1		nonsynonymous SNV	ARMCX1:NM_016608:exon4:c.G491A:p.R1	NOVEL		216	195	1.08379	1	235	201	0.936234	1	278	223	0.903741	1
20	chr6	34512073	34512073	C	T	exonic	SPDEF		nonsynonymous SNV	SPDEF:NM_001252294:exon2:c.G160A:p.G	COSMIC	ID=COSM335091	188	142	0.98294	1	171	145	0.931871	1	192	187	1.0018	1
20	chrX	100808403	100808403	A	T	exonic	ARMCX1		nonsynonymous SNV	ARMCX1:NM_016608:exon4:c.A490T:p.R1	NOVEL		219	191	1.06415	1	237	200	0.929444	1	279	228	0.913069	1
20	chr8	30969189	30969189	G	A	exonic	WRN		nonsynonymous SNV	WRN:NM_000553:exon19:c.G2147A:p.R71	DBSNP		191	174	1.08895	1	196	165	0.928221	1	249	223	0.959267	1
20	chr1	114254372	114254372	C	T	exonic	PHTF1		nonsynonymous SNV	PHTF1:NM_001323046:exon7:c.G185A:p.S	NOVEL		327	0	0	1	181	152	0.926988	1	192	212	1.06545	1
20	chr1	161696548	161696548	G	A	exonic	FCRLB		synonymous SNV	FCRLB:NM_001320241:exon6:c.G630A:p.A	DBSNP		209	13	0.192324	1	148	65	0.924903	1	174	92	1.0481	1
20	chr1	201926416	201926416	C	T	exonic	TIMM17A		stopgain	TIMM17A:NM_006335:exon2:c.C34T:p.R1	DBSNP+COSMIC	ID=COSM402697	1041	3	0.00943763	1	769	337	0.923501	1	917	403	0.925183	1
20	chr16	58044976	58044976	G	C	exonic	USB1		nonsynonymous SNV	USB1:NM_001204911:exon4:c.G504C:p.Q	NOVEL		146	0	0	1	84	69	0.915867	1	104	84	0.90719	1
20	chr11	72469614	72469614	G	C	exonic	STARD10		synonymous SNV	STARD10:NM_006645:exon4:c.C420G:p.G	NOVEL		689	2	0.00950592	1	433	186	0.91072	1	505	234	0.959551	1
20	chr1	68895609	68895609	A	G	exonic	RPE65		synonymous SNV	RPE65:NM_000329:exon14:c.T1452C:p.G4	NOVEL		473	3	0.0143968	1	248	200	0.906623	1	289	248	0.937679	1
20	chrX	88008443	88008443	G	C	exonic	CPXCR1		nonsynonymous SNV	CPXCR1:NM_001184771:exon3:c.G28C:p.D	NOVEL		155	132	1.05062	1	154	124	0.905841	1	183	179	1.00397	1
20	chr16	89263778	89263778	C	G	exonic	SLC22A31		nonsynonymous SNV	SLC22A31:NM_001366322:exon8:c.G908C	NOVEL		275	0	0	1	151	121	0.903423	1	156	157	1.01843	1
20	chr12	65224221	65224221	G	A	exonic	TBC1D30		nonsynonymous SNV	TBC1D30:NM_001330186:exon4:c.G347A:	NOVEL		650	0	0	1	358	285	0.900137	1	437	381	0.945689	1
20	chr11	7670085	7670085	G	A	exonic	PPFIBP2		nonsynonymous SNV	PPFIBP2:NM_001256569:exon15:c.G1423A	DBSNP		707	1	0.00322641	1	366	289	0.896048	1	420	361	0.938497	1
20	chrX	149638462	149638462	A	G	exonic	MAMLD1		nonsynonymous SNV	MAMLD1:NM_001177466:exon2:c.A542G:	NOVEL		385	0	0	1	204	160	0.892675	1	240	203	0.930399	1
20	chr2	179569638	179569638	A	G	exonic	TTN		nonsynonymous SNV	TTN:NM_133378:exon101:c.T25928C:p.V8	NOVEL		109	0	0	1	68	53	0.88954	1	93	71	0.879005	1
20	chr11	57004106	57004106	A	G	exonic	APLNR		nonsynonymous SNV	APLNR:NM_005161:exon1:c.T373C:p.F125	NOVEL		175	22	0.255099	1	116	89	0.88168	1	98	117	1.1049	1
20	chr19	4433253	4433253	G	C	exonic	CHAF1A		nonsynonymous SNV	CHAF1A:NM_005483:exon13:c.G2390C:p.R	NOVEL		439	0	0	1	235	177	0.872471	1	251	217	0.941436	1
20	chr6	168376900	168376900	G	T	exonic	HGC6.3		nonsynonymous SNV	HGC6.3:NM_001129895:exon1:c.C433A:p.	NOVEL		79	53	0.91718	1	72	54	0.870358	1	93	66	0.842798	1
20	chr11	35333840	35333840	C	G	exonic	SLC1A2		nonsynonymous SNV	SLC1A2:NM_004171:exon4:c.G466C:p.G15	NOVEL		283	0	0	1	165	121	0.8592	1	159	189	1.1027	1
20	chr17	76122392	76122392	G	C	exonic	TMC6		synonymous SNV	TMC6:NM_001127198:exon4:c.C237G:p.L7	NOVEL		213	0	0	1	103	75	0.855689	1	100	95	0.989158	1
20	chr18	19756936	19756936	G	A	exonic	GATA6		nonsynonymous SNV	GATA6:NM_005257:exon3:c.G1156A:p.E38	NOVEL		45	0	0	1	21	15	0.846182	1	19	20	1.04122	1
20	chr7	2627493	2627493	C	T	exonic	IQCE		synonymous SNV	IQCE:NM_001287501:exon11:c.C831T:p.R2	NOVEL		141	147	1.16594	1	144	102	0.842054	1	167	163	1.00288	1
20	chr8	128750683	128750683	C	G	exonic	MYC		nonsynonymous SNV	MYC:NM_001354870:exon2:c.C217G:p.P7	DBSNP+COSMIC	ID=COSM565630	498	1	0.00457775	1	309	213	0.828674	1	322	275	0.935266	1
20	chr5	140209558	140209558	A	T	exonic	PCDHA6		nonsynonymous SNV	PCDHA6:NM_018909:exon1:c.A1882T:p.T6	NOVEL		300	1	0.00758902	1	155	87	0.730094	1	177	121	0.824415	1
20	chr2	26405952	26405952	A	G	exonic	GAREM2		nonsynonymous SNV	GAREM2:NM_001191033:exon2:c.A139G:	NOVEL		149	2	0.0302556	1	152	51	0.51021	1	188	47	0.406075	1
20	chr10	68687832	68687832	G	A	exonic	LRRTM3		synonymous SNV	LRRTM3:NM_178011:exon2:c.G1158A:p.R	NOVEL		290	0	0	1	247	70	0.44845	1	344	71	0.347366	1
20	chr17	16004807	16004807	G	A	exonic	NCOR1		nonsynonymous SNV	NCOR1:NM_001190438:exon17:c.C2168T:	NOVEL		653	0	0	1	539	142	0.423464	1	654	133	0.343126	1
20	chr17	74075582	74075582	A	G	exonic	ZACN		nonsynonymous SNV	ZACN:NM_180990:exon2:c.A160G:p.N54D	NOVEL		703	3	0.00970664	1	484	126	0.419484	1	623	129	0.348296	1
20	chr19	1127702	1127702	C	A	exonic	SBNO2		synonymous SNV	SBNO2:NM_001100122:exon2:c.G171T:p.	NOVEL		219	0	0	1	168	43	0.413867	1	225	53	0.387086	1
20	chr2	209028031	209028031	T	A	exonic	CRYGA		nonsynonymous SNV	CRYGA:NM_014617:exon2:c.A149T:p.N50	NOVEL		279	0	0	1	243	62	0.412826	1	285	47	0.287433	1
20	chr10	461747	461747	C	G	exonic	DIP2C		nonsynonymous SNV	DIP2C:NM_014974:exon7:c.G821C:p.R274	NOVEL		733	0	0	1	580	143	0.401673	1	711	141	0.336013	1
20	chr19	52658805	52658805	T	A	exonic	ZNF836		stopgain	ZNF836:NM_001102657:exon5:c.A2131T:	NOVEL		511	0	0	1	475	117	0.401365	1	574	102	0.306359	1
20	chr2	179469827	179469827	G	A	exonic	TTN		nonsynonymous SNV	TTN:NM_003319:exon108:c.C26882T:p.A8	NOVEL		572	0	0	1	529	126	0.390665	1	650	128	0.334047	1
20	chr22	43623412	43623412	C	G	exonic	SCUBE1		nonsynonymous SNV	SCUBE1:NM_173050:exon10:c.G1175C:p.R	NOVEL		182	0	0	1	127	30	0.388058	1	185	34	0.315218	1
20	chr13	37446991	37446991	G	T	exonic	SMAD9		synonymous SNV	SMAD9:NM_001127217:exon3:c.C474A:p.	NOVEL		318	0	0	1	258	59	0.377979	1	333	45	0.241712	1
20	chr2	202136308	202136308	A	G	exonic	CASP8		synonymous SNV	CASP8:NM_001080125:exon3:c.A552G:p.	NOVEL		473	2	0.00961809	1	352	80	0.376081	1	516	90	0.301541	1
20	chr11	119003576	119003576	G	A	exonic	HINFP		nonsynonymous SNV	HINFP:NM_001351963:exon7:c.G260A:p.C	NOVEL		308	0	0	1	260	59	0.375609	1	316	66	0.350798	1
20	chr17	1628909	1628909	T	A	exonic	WDR81		stopgain	WDR81:NM_001163809:exon1:c.T656A:p.	NOVEL		447	0	0	1	390	85	0.363413	1	449	97	0.360708	1
20	chr2	11054006	11054006	A	T	exonic	KCNF1		nonsynonymous SNV	KCNF1:NM_002236:exon1:c.A1454T:p.H48	NOVEL		444	0	0	1	323	69	0.357469	1	443	77	0.300652	1
20	chr17	40475059	40475059	T	C	exonic	STAT3		synonymous SNV	STAT3:NM_001369512:exon20:c.A1851G:	NOVEL		140	0	0	1	129	27	0.351491	1	152	26	0.296572	1
20	chr9	139972984	139972984	G	T	exonic	UAP1L1		synonymous SNV	UAP1L1:NM_207309:exon3:c.G525T:p.G1	NOVEL		751	0	0	1	568	118	0.349327	1	669	141	0.353436	1
20	chr17	40475058	40475058	C	G	exonic	STAT3		nonsynonymous SNV	STAT3:NM_001369512:exon20:c.G1852C:	COSMIC	ID=COSM116677	137	0	0	1	129	26	0.340656	1	148	26	0.30339	1
20	chr6	54095575	54095575	C	A	exonic	MLIP		nonsynonymous SNV	MLIP:NM_138569:exon11:c.C1177A:p.P39	NOVEL		430	1	0.00529999	1	350	70	0.338473	1	461	70	0.267658	1
20	chr4	184233500	184233500	C	T	exonic	WWC2		stopgain	WWC2:NM_024949:exon22:c.C3391T:p.Q1	NOVEL		782	0	0	1	591	118	0.337995	1	777	143	0.315591	1
20	chr9	19119739	19119739	G	C	exonic	PLIN2		nonsynonymous SNV	PLIN2:NM_001122:exon6:c.C686G:p.S229	DBSNP		147	1	0.0154344	1	132	26	0.334188	1	127	24	0.322709	1
20	chr11	58275154	58275154	A	C	exonic	OR5B21		nonsynonymous SNV	OR5B21:NM_001005218:exon1:c.T425G:p	NOVEL		299	0	0	1	241	47	0.331421	1	299	44	0.260457	1
20	chrX	70351987	70351987	A	T	exonic	MED12		nonsynonymous SNV	MED12:NM_005120:exon30:c.A4184T:p.E	NOVEL		277	0	0	1	262	51	0.330903	1	341	53	0.273122	1
20	chr11	45671409	45671409	G	C	exonic	CHST1		synonymous SNV	CHST1:NM_003654:exon4:c.C1065G:p.A35	NOVEL		462	0	0	1	326	61	0.320106	1	421	65	0.271553	1
20	chr17	40257185	40257185	T	A	exonic	DHX58		nonsynonymous SNV	DHX58:NM_024119:exon10:c.A1252T:p.R4	NOVEL		271	0	0	1	197	36	0.313777	1	249	54	0.361849	1
20	chr15	25940114	25940114	G	C	exonic	ATP10A		synonymous SNV	ATP10A:NM_024490:exon14:c.C2940G:p.A	NOVEL		381	0	0	1	285	52	0.313363	1	385	79	0.345689	1
20	chr1	95492778	95492778	C	A	exonic	ALG14		stopgain	ALG14:NM_001305242:exon4:c.G364T:p.	NOVEL		216	0	0	1	176	32	0.312436	1	217	36	0.288907	1
20	chr14	104642957	104642957	G	C	exonic	KIF26A		nonsynonymous SNV	KIF26A:NM_015656:exon12:c.G3832C:p.A	NOVEL		345	0	0	1	308	35	0.309269	1	350	30	0.23924	1
20	chr5	169028626	169028626	C	T	exonic	SPDL1		nonsynonymous SNV	SPDL1:NM_001329639:exon11:c.C1667T:p	NOVEL		635	0	0	1	575	102	0.305975	1	649	113	0.301093	1
20	chr11	62491194	62491194	G	T	exonic	HNRNPUL2		synonymous SNV	HNRNPUL2:NM_001079559:exon4:c.C756	NOVEL		310	0	0	1	300	53	0.304913	1	341	65	0.32506	1
20	chr18	63525054	63525054	A	T	exonic	CDH7		nonsynonymous SNV	CDH7:NM_001317214:exon8:c.A1238T:p.Y	NOVEL		199	0	0	1	170	30	0.304625	1	207	24	0.210948	1
20	chr11	5536924	5536924	A	T	exonic	UBQLNL		nonsynonymous SNV	UBQLNL:NM_145053:exon1:c.T748A:p.Y2	NOVEL		428	0	0	1	399	70	0.30311	1	480	80	0.290054	1
20	chr17	7577535	7577535	C	G	exonic	TP53		nonsynonymous SNV	TP53:NM_001126115:exon3:c.G350C:p.R1	DBSNP+COSMIC	ID=COSM43665,	174	0	0	1	175	30	0.297196	1	201	37	0.315647	1
20	chr19	18047262	18047262	G	C	exonic	CCDC124		nonsynonymous SNV	CCDC124:NM_001136203:exon2:c.G33C:p.	NOVEL		323	0	0	1	264	45	0.295753	1	295	62	0.352615	1
20	chr4	159634324	159634324	C	T	exonic	PPID		nonsynonymous SNV	PPID:NM_005038:exon7:c.G841A:p.A281T	NOVEL		643	0	0	1	607	100	0.287247	1	766	110	0.254956	1
20	chr15	89383293	89383293	G	C	exonic	ACAN		nonsynonymous SNV	ACAN:NM_001135:exon4:c.G505C:p.D169	NOVEL		315	0	0	1	238	38	0.279608	1	266	38	0.253797	1
20	chr1	169821981	169821981	G	C	exonic	C1orf112		nonsynonymous SNV	C1orf112:NM_001366773:exon22:c.G1446	NOVEL		638	1	0.00513974	1	594	55	0.256851	1	732	60	0.229574	1
20	chr2	179501475	179501475	C	T	exonic	TTN		nonsynonymous SNV	TTN:NM_003319:exon53:c.G13784A:p.R4	NOVEL		514	0	0	1	473	60	0.228612	1	569	68	0.216744	1
20	chr20	51870164	51870164	C	T	exonic	TSHZ2		nonsynonymous SNV	TSHZ2:NM_001193421:exon2:c.C158T:p.T	DBSNP		221	0	0	1	203	23	0.206678	1	235	16	0.129426	1
20	chr19	57988651	57988651	C	G	exonic	ZNF772		synonymous SNV	ZNF772:NM_001024596:exon1:c.G27C:p.P	NOVEL		421	0	0	1	356	40	0.205135	1	423	40	0.175411	1
20	chr19	13227133	13227133	C	A	exonic	TRMT1		nonsynonymous SNV	TRMT1:NM_001142554:exon1:c.G81T:p.Q	NOVEL		369	0	0	1	328	36	0.200852	1	380	49	0.231908	1
20	chr17	73654422	73654422	C	G	exonic	RECQL5		nonsynonymous SNV	RECQL5:NM_001003715:exon7:c.G1105C:	NOVEL		301	0	0	1	262	28	0.196081	1	306	46	0.265333	1
20	chr17	7579359	7579359	G	A	exonic	TP53		nonsynonymous SNV	TP53:NM_001126118:exon3:c.C211T:p.R7	DBSNP+COSMIC	ID=COSM247949	39	0	0	1	42	4	0.176594	1	53	4	0.142483	1
20	chr5	140222978	140222978	C	A	exonic	PCDHA8		nonsynonymous SNV	PCDHA8:NM_018911:exon1:c.C2072A:p.A	DBSNP		306	0	0	1	301	27	0.167172	1	346	22	0.121381	1
20	chr1	186270776	186270776	T	G	exonic	PRG4		nonsynonymous SNV	PRG4:NM_001127708:exon3:c.T129G:p.C	NOVEL		248	1	0.0131899	1	223	13	0.166953	1	277	21	0.21355	1
20	chr3	74420451	74420451	T	C	exonic	CNTN3		nonsynonymous SNV	CNTN3:NM_020872:exon5:c.A554G:p.K18	NOVEL		257	1	0.00885386	1	273	24	0.164108	1	370	37	0.18458	1
20	chr9	139412660	139412660	T	A	exonic	NOTCH1		nonsynonymous SNV	NOTCH1:NM_017617:exon7:c.A1184T:p.K3	COSMIC	ID=COSM587852	178	0	0	1	161	14	0.162467	1	164	18	0.200807	1
20	chr16	27475761	27475761	C	A	exonic	GTF3C1		nonsynonymous SNV	GTF3C1:NM_001286242:exon34:c.G5752T	NOVEL		433	1	0.00526336	1	422	36	0.159629	1	465	47	0.186382	1
20	chr10	120824934	120824934	G	T	exonic	EIF3A		synonymous SNV	EIF3A:NM_003750:exon7:c.C1099A:p.R36	NOVEL		581	0	0	1	509	42	0.154801	1	596	81	0.242925	1
20	chr11	111626047	111626047	C	T	exonic	PPP2R1B		nonsynonymous SNV	PPP2R1B:NM_001177563:exon4:c.G434A:	DBSNP+COSMIC	ID=COSM373886	591	0	0	1	582	47	0.151748	1	653	65	0.183809	1
20	chr6	55933882	55933882	G	C	exonic	COL21A1		nonsynonymous SNV	COL21A1:NM_001318753:exon6:c.C247G:	NOVEL		563	0	0	1	517	41	0.149219	1	639	75	0.213275	1
20	chrX	21581476	21581476	G	A	exonic	CNKSR2		nonsynonymous SNV	CNKSR2:NM_001330771:exon11:c.G1277A	NOVEL		844	0	0	1	831	59	0.134628	1	900	71	0.148462	1
20	chr12	88974054	88974054	A	G	exonic	KITLG		startloss	KITLG:NM_000899:exon1:c.T2C:p.M1?,KIT	NOVEL		386	0	0	1	312	20	0.12234	1	388	24	0.118274	1
20	chr1	165322373	165322373	G	A	exonic	LMX1A		nonsynonymous SNV	LMX1A:NM_001174069:exon3:c.C203T:p.P	NOVEL		322	0	0	1	311	13	0.121608	1	367	18	0.14168	1
20	chr2	56098211	56098211	A	G	exonic	EFEMP1		nonsynonymous SNV	EFEMP1:NM_001039349:exon9:c.T1048C:	NOVEL		691	0	0	1	674	36	0.102972	1	752	35	0.0902963	1
20	chr1	217975104	217975104	C	A	exonic	SPATA17		nonsynonymous SNV	SPATA17:NM_138796:exon9:c.C917A:p.P3	NOVEL		289	0	0	1	267	9	0.0988316	1	367	8	0.064648	1
20	chr1	161047527	161047527	G	A	exonic	NECTIN4		nonsynonymous SNV	NECTIN4:NM_030916:exon3:c.C446T:p.P1	NOVEL		266	1	0.0123007	1	268	9	0.0984748	1	295	15	0.146631	1
20	chr3	155199100	155199100	G	C	exonic	PLCH1		nonsynonymous SNV	PLCH1:NM_014996:exon22:c.C4715G:p.S1	NOVEL		543	0	0	1	515	26	0.0976002	1	594	37	0.119055	1
20	chr2	166786187	166786187	T	A	exonic	TTC21B		nonsynonymous SNV	TTC21B:NM_024753:exon10:c.A1158T:p.E	NOVEL		369	0	0	1	395	19	0.0932026	1	430	29	0.128281	1
20	chr8	8235361	8235361	C	T	exonic	PRAG1		synonymous SNV	PRAG1:NM_001080826:exon3:c.G558A:p.	NOVEL		519	1	0.00439288	1	479	23	0.0930463	1	573	34	0.113728	1
20	chr4	55129951	55129951	A	T	exonic	PDGFRA		nonsynonymous SNV	PDGFRA:NM_001347827:exon4:c.A485T:p	NOVEL		403	1	0.0056542	1	405	19	0.0910044	1	526	19	0.0707838	1
20	chr19	9639090	9639090	G	T	exonic	ZNF426		nonsynonymous SNV	ZNF426:NM_001300883:exon6:c.C1517A:	NOVEL		571	0	0	1	590	27	0.0888696	1	742	12	0.0323137	1
20	chrX	154059061	154059061	A	G	exonic	SMIM9		nonsynonymous SNV	SMIM9:NM_001162936:exon3:c.T71C:p.V	NOVEL		676	1	0.00337415	1	696	29	0.0812334	1	795	15	0.0375996	1
20	chr14	73751052	73751052	G	A	exonic	NUMB		nonsynonymous SNV	NUMB:NM_001005745:exon9:c.C653T:p.S	NOVEL		256	0	0	1	218	9	0.0805177	1	287	15	0.100847	1
20	chr18	224161	224161	A	T	exonic	THOC1		synonymous SNV	THOC1:NM_005131:exon16:c.T1227A:p.P4	DBSNP		680	0	0	1	621	23	0.0725299	1	736	8	0.021832	1
20	chr6	34003563	34003563	C	T	exonic	GRM4		nonsynonymous SNV	GRM4:NM_001256809:exon7:c.G1976A:p.	DBSNP		506	0	0	1	447	16	0.0701801	1	541	8	0.0295866	1
20	chr16	23091476	23091476	A	C	exonic	USP31		nonsynonymous SNV	USP31:NM_020718:exon13:c.T1967G:p.M	NOVEL		556	0	0	1	543	18	0.0651605	1	658	8	0.0243889	1
20	chr5	128957927	128957927	G	A	exonic	ADAMTS19		synonymous SNV	ADAMTS19:NM_133638:exon10:c.G1656A	DBSNP+COSMIC	ID=COSM481856	485	0	0	1	487	16	0.0645992	1	584	14	0.0475339	1
20	chr17	7578403	7578403	C	A	exonic	TP53		nonsynonymous SNV	TP53:NM_001126115:exon1:c.G131T:p.C4	DBSNP+COSMIC	ID=COSM117399	330	2	0.0137608	1	314	10	0.0626801	1	352	4	0.0228132	1
20	chr10	116068259	116068259	C	T	exonic	AFAP1L2		synonymous SNV	AFAP1L2:NM_001001936:exon9:c.G900A:	NOVEL		417	0	0	1	385	12	0.0613855	1	465	15	0.0634493	1
20	chr3	196867030	196867030	C	A	exonic	DLG1		nonsynonymous SNV	DLG1:NM_001204387:exon4:c.G445T:p.A	NOVEL		668	0	0	1	613	18	0.0579319	1	798	32	0.0782796	1
20	chr5	158750205	158750205	G	A	exonic	IL12B		nonsynonymous SNV	IL12B:NM_002187:exon3:c.C221T:p.T74I	NOVEL		225	0	0	1	209	6	0.0566745	1	242	19	0.147805	1
20	chr6	46424497	46424497	C	T	exonic	RCAN2		nonsynonymous SNV	RCAN2:NM_001251973:exon2:c.G217A:p.	NOVEL		460	0	0	1	505	14	0.0547817	1	571	9	0.0315059	1
20	chr11	1857358	1857358	C	A	exonic	SYT8		synonymous SNV	SYT8:NM_001290332:exon5:c.C405A:p.I13	NOVEL		483	0	0	1	418	10	0.0474494	1	516	18	0.0684397	1
20	chr2	80530808	80530808	T	A	exonic	LRRTM1		nonsynonymous SNV	LRRTM1:NM_178839:exon2:c.A137T:p.E46	NOVEL		332	2	0.0136784	1	362	5	0.0276681	1	412	12	0.0574635	1
20	chr8	3019693	3019693	C	G	exonic	CSMD1		synonymous SNV	CSMD1:NM_033225:exon38:c.G5832C:p.G	NOVEL		306	15	0.106743	1	325	1	0.00622956	1	370	0	0	1
20	chr15	42280277	42280277	T	C	exonic	PLA2G4E		nonsynonymous SNV	PLA2G4E:NM_001206670:exon16:c.A1801	NOVEL		334	14	0.091897	1	346	1	0.00585255	1	370	0	0	1
20	chr8	42012409	42012409	C	G	exonic	AP3M2		synonymous SNV	AP3M2:NM_006803:exon2:c.C204G:p.A68	NOVEL		474	23	0.105712	1	419	1	0.00483532	1	549	0	0	1
20	chr8	55538150	55538150	A	G	exonic	RP1		nonsynonymous SNV	RP1:NM_006269:exon4:c.A1708G:p.I570V	NOVEL		261	142	0.804889	1	402	0	0	1	514	0	0	1
20	chr1	118166305	118166305	C	A	exonic	FAM46C		nonsynonymous SNV	FAM46C:NM_017709:exon2:c.C815A:p.S27	NOVEL		290	151	0.782151	1	513	0	0	1	534	0	0	1
20	chr18	20602154	20602154	A	G	exonic	RBBP8		nonsynonymous SNV	RBBP8:NM_203292:exon17:c.A2420G:p.D	NOVEL		580	273	0.731082	1	943	0	0	1	1143	4	0.00708065	1
20	chr15	45557781	45557781	G	T	exonic	SLC28A2		nonsynonymous SNV	SLC28A2:NM_004212:exon9:c.G781T:p.A2	NOVEL		202	78	0.63634	1	338	0	0	1	347	0	0	1
20	chr16	22926750	22926750	G	A	exonic	HS3ST2		nonsynonymous SNV	HS3ST2:NM_006043:exon2:c.G971A:p.R32	COSMIC	ID=COSM157986	505	185	0.612456	1	724	0	0	1	840	0	0	1
20	chr9	127920640	127920640	A	C	exonic	PPP6C		nonsynonymous SNV	PPP6C:NM_001123369:exon3:c.T193G:p.Y	NOVEL		179	13	0.154666	1	183	0	0	1	234	1	0.0086399	1
20	chr14	24776648	24776648	G	A	exonic	CIDEB		nonsynonymous SNV	CIDEB:NM_014430:exon4:c.C115T:p.R39C	DBSNP		205	11	0.11633	1	202	0	0	1	245	0	0	1
20	chr5	147024485	147024485	G	C	exonic	JAKMIP2		nonsynonymous SNV	JAKMIP2:NM_001282282:exon5:c.C885G:p	NOVEL		553	23	0.0912132	1	571	0	0	1	630	0	0	1
20	chr15	39880376	39880376	C	T	exonic	THBS1		synonymous SNV	THBS1:NM_003246:exon9:c.C1428T:p.G47	DBSNP		316	12	0.0835718	1	303	0	0	1	357	0	0	1
20	chr9	135977352	135977352	C	T	exonic	RALGDS		synonymous SNV	RALGDS:NM_001042368:exon15:c.G2019A	DBSNP		291	10	0.0758902	1	299	0	0	1	376	0	0	1
20	chr13	103339380	103339380	C	T	exonic	METTL21C		nonsynonymous SNV	METTL21C:NM_001010977:exon3:c.G310A	NOVEL		738	23	0.0690392	1	762	0	0	1	906	1	0.00223856	1
20	chr1	18809727	18809727	G	A	exonic	KLHDC7A		nonsynonymous SNV	KLHDC7A:NM_152375:exon1:c.G2252A:p.R	DBSNP		675	20	0.0657351	1	681	0	0	1	804	0	0	1
20	chr2	183822184	183822184	C	G	splicing	NCKAP1	NM_013436:exon19:c.			NOVEL		516	2	0.00881968	1	267	280	1.03955	1	349	301	0.940221	1
20	chr10	129213415	129213415	G	A	splicing	DOCK1	NM_001290223:exon4			NOVEL		495	0	0	1	241	215	0.957521	1	298	264	0.953771	1
20	chr17	78081598	78081598	G	A	splicing	GAA	NM_000152:exon5:c.8			NOVEL		140	0	0	1	84	72	0.937309	1	98	87	0.954826	1
20	chr12	25656670	25656670	A	G	splicing	LMNTD1	NM_001145728:exon9			DBSNP		78	0	0	1	77	15	0.331115	1	80	17	0.355839	1
23	chr5	9066732	9066732	C	T	exonic	SEMA5A		synonymous SNV	SEMA5A:NM_003966:exon17:c.G2100A:p.	DBSNP+COSMIC	ID=COSM314104	151	90	1.1956	1	171	139	0.840657	2	171	117	1.28756	1
23	chr12	7053308	7053308	G	A	exonic	C12orf57		synonymous SNV	C12orf57:NM_001301837:exon1:c.G24A:p.	DBSNP		101	76	1.04174	1	139	117	1.18069	1	121	121	1.15548	1
23	chr3	164741517	164741517	T	A	exonic	SI		nonsynonymous SNV	SI:NM_001041:exon26:c.T2940A:p.D980E	NOVEL		76	202	1.24476	2	144	241	0.991256	2	79	187	1.11405	2
23	chr1	82421645	82421645	A	G	exonic	ADGRL2		nonsynonymous SNV	ADGRL2:NM_001297705:exon9:c.A1867G:	DBSNP		14	169	1.12028	2	38	221	1.10218	2	9	194	1.10425	2
23	chr2	25799765	25799765	C	T	exonic	DTNB		nonsynonymous SNV	DTNB:NM_001320935:exon6:c.G206A:p.R	DBSNP		73	48	0.962444	1	84	70	1.17426	1	76	68	1.09128	1
23	chr3	130674983	130674983	C	T	exonic	ATP2C1		synonymous SNV	ATP2C1:NM_001199182:exon9:c.C777T:p.	NOVEL		330	38	0.353788	1	290	125	0.953941	1	232	115	1.05037	1
23	chr3	109050858	109050858	C	T	exonic	DPPA4		nonsynonymous SNV	DPPA4:NM_001348928:exon3:c.G43A:p.A1	NOVEL		144	19	0.399368	1	133	34	0.644796	1	103	51	1.0496	1
23	chr1	7890026	7890026	A	G	exonic	PER3		nonsynonymous SNV	PER3:NM_001289862:exon19:c.A3019G:p.	DBSNP+COSMIC	ID=COSM215737	93	6	0.14704	1	121	12	0.233087	1	101	84	1.0493	1
23	chr10	55570420	55570420	C	A	exonic	PCDH15		stopgain	PCDH15:NM_001354420:exon33:c.G4372T	NOVEL		116	112	1.28759	1	125	212	1.12712	2	77	146	1.04023	2
23	chr3	113063427	113063427	T	G	exonic	CFAP44		nonsynonymous SNV	CFAP44:NM_001164496:exon23:c.A3198C	NOVEL		67	146	1.17422	2	92	134	0.938914	2	78	146	1.03288	2
23	chr1	7913459	7913459	G	A	exonic	UTS2		synonymous SNV	UTS2:NM_021995:exon1:c.C33T:p.C11C	DBSNP		159	31	0.395847	1	126	77	0.979903	1	116	88	0.996882	1
23	chr9	21334551	21334551	A	G	exonic	KLHL9		nonsynonymous SNV	KLHL9:NM_018847:exon1:c.T308C:p.L103P	NOVEL		115	186	0.934157	2	172	230	0.906007	2	54	115	0.982457	2
23	chr9	118974159	118974159	T	G	exonic	PAPPA		nonsynonymous SNV	PAPPA:NM_002581:exon4:c.T1866G:p.C62	NOVEL		175	18	0.28198	1	118	67	1.147	1	140	72	0.980684	1
23	chr1	17548923	17548923	C	T	exonic	PADI1		synonymous SNV	PADI1:NM_013358:exon2:c.C231T:p.V77V	DBSNP		75	54	1.0156	1	82	60	1.09157	1	106	78	0.979644	1
23	chr1	7890024	7890024	T	G	exonic	PER3		nonsynonymous SNV	PER3:NM_001289862:exon19:c.T3017G:p.	DBSNP+COSMIC	ID=COSM215736	98	4	0.0951435	1	127	8	0.153089	1	108	68	0.892869	1
23	chr6	168443328	168443328	T	C	exonic	KIF25		nonsynonymous SNV	KIF25:NM_030615:exon8:c.T917C:p.L306S	NOVEL		97	17	0.361796	1	81	10	0.195351	1	132	50	0.87071	1
23	chr5	170883784	170883784	G	A	exonic	FGF18		nonsynonymous SNV	FGF18:NM_003862:exon5:c.G599A:p.R200	DBSNP+COSMIC	ID=COSM535694	65	22	0.764551	1	76	36	1.01799	1	77	33	0.85085	1
23	chr1	152192163	152192163	G	T	exonic	HRNR		nonsynonymous SNV	HRNR:NM_001009931:exon3:c.C1942A:p.	DBSNP+COSMIC	ID=COSM619195	149	3	0.0596734	1	164	2	0.0382998	1	61	25	0.824467	1
23	chr4	79321971	79321971	A	T	exonic	FRAS1		nonsynonymous SNV	FRAS1:NM_001166133:exon30:c.A4059T:	NOVEL		205	21	0.28094	1	199	76	0.878528	1	113	57	0.821157	1
23	chr1	158621162	158621162	G	A	exonic	SPTA1		nonsynonymous SNV	SPTA1:NM_003126:exon24:c.C3472T:p.R1	DBSNP+COSMIC	ID=COSM897816	60	4	0.188966	1	84	28	0.794721	1	53	21	0.804858	1
23	chr8	116616131	116616131	G	A	exonic	TRPS1		stopgain	TRPS1:NM_001282902:exon3:c.C2038T:p.	NOVEL		83	91	1.49953	1	100	93	1.24484	1	72	38	0.79833	1
23	chr9	101585600	101585600	A	G	exonic	GALNT12		nonsynonymous SNV	GALNT12:NM_024642:exon2:c.A434G:p.Y	NOVEL		127	65	1.02356	1	161	82	1.06873	1	122	46	0.790644	1
23	chr8	20112683	20112683	C	T	exonic	LZTS1		nonsynonymous SNV	LZTS1:NM_001362884:exon2:c.G10A:p.V4	DBSNP		74	35	0.920669	1	51	35	1.05138	1	31	16	0.786708	1
23	chr1	17313087	17313087	C	T	exonic	ATP13A2		synonymous SNV	ATP13A2:NM_001141973:exon28:c.G3261	NOVEL		54	0	0	1	37	5	0.307545	1	45	19	0.686065	1
23	chr1	17273321	17273321	G	A	exonic	CROCC		synonymous SNV	CROCC:NM_014675:exon17:c.G2349A:p.A	DBSNP		108	14	0.278412	1	86	25	0.581843	1	164	69	0.68436	1
23	chr1	7890016	7890016	G	A	exonic	PER3		synonymous SNV	PER3:NM_001289862:exon19:c.G3009A:p.	DBSNP+COSMIC	ID=COSM224271	116	0	0	1	151	1	0.0169959	1	120	50	0.679692	1
23	chr4	22390236	22390236	A	G	exonic	ADGRA3		nonsynonymous SNV	ADGRA3:NM_145290:exon19:c.T3058C:p.	NOVEL		225	114	1.01674	1	243	115	1.02115	1	231	86	0.664415	1
23	chr14	32257073	32257073	A	G	exonic	NUBPL		nonsynonymous SNV	NUBPL:NM_001201574:exon2:c.A52G:p.I1	NOVEL		57	31	1.09093	1	75	28	0.860955	1	47	14	0.662721	1
23	chr22	29708365	29708365	G	A	exonic	GAS2L1		nonsynonymous SNV	GAS2L1:NM_001278730:exon6:c.G1244A:	DBSNP		167	0	0	1	182	8	0.108774	1	270	104	0.642618	1
23	chr4	22390238	22390238	A	G	exonic	ADGRA3		nonsynonymous SNV	ADGRA3:NM_145290:exon19:c.T3056C:p.	DBSNP		220	115	1.0379	1	247	115	1.00987	1	238	83	0.633247	1
23	chr22	19165735	19165735	A	G	exonic	SLC25A1		nonsynonymous SNV	SLC25A1:NM_001256534:exon1:c.T134C:p	NOVEL		87	0	0	1	89	2	0.0567776	1	89	31	0.596997	1
23	chr17	71189228	71189228	C	T	exonic	COG1		nonsynonymous SNV	COG1:NM_018714:exon1:c.C20T:p.S7L	DBSNP		51	0	0	1	63	6	0.224642	1	47	16	0.586909	1
23	chr7	21757433	21757433	G	T	exonic	DNAH11		stopgain	DNAH11:NM_001277115:exon43:c.G7024T	NOVEL		192	0	0	1	279	9	0.104544	1	135	35	0.583917	1
23	chr21	45534070	45534070	G	C	exonic	LOC102724159;PV		synonymous SNV	LOC102724159:NM_001368238:exon4:c.G	NOVEL		74	0	0	1	87	7	0.19238	1	86	29	0.582762	1
23	chr8	48866919	48866919	T	C	exonic	PRKDC		unknown	UNKNOWN	NOVEL		91	0	0	1	61	4	0.158977	1	15	5	0.577739	1
23	chr12	25380275	25380275	T	G	exonic	KRAS		nonsynonymous SNV	KRAS:NM_001369786:exon3:c.A183C:p.Q6	DBSNP+COSMIC	ID=COSM554,CO	87	0	0	1	104	9	0.205756	1	53	16	0.535873	1
23	chr11	6579230	6579230	C	T	exonic	DNHD1		nonsynonymous SNV	DNHD1:NM_144666:exon25:c.C8705T:p.T2	NOVEL		349	0	0	1	336	7	0.0646344	1	364	67	0.461903	1
23	chr5	121495925	121495925	A	G	exonic	LOC100505841		startloss	LOC100505841:NM_001195535:exon1:c.A	NOVEL		275	0	0	1	318	13	0.124387	1	225	43	0.455056	1
23	chr6	7581236	7581236	C	T	exonic	DSP		synonymous SNV	DSP:NM_004415:exon23:c.C4813T:p.L1605	NOVEL		129	0	0	1	182	5	0.0846814	1	122	21	0.4165	1
23	chr7	149558506	149558506	G	A	exonic	ZNF862		nonsynonymous SNV	ZNF862:NM_001099220:exon7:c.G2257A:	DBSNP+COSMIC	ID=COSM476247	180	0	0	1	190	10	0.158354	1	153	26	0.411957	1
23	chr17	39623497	39623497	G	A	exonic	KRT32		synonymous SNV	KRT32:NM_002278:exon1:c.C81T:p.S27S	DBSNP		74	11	0.313974	1	63	32	0.870192	1	81	16	0.381188	1
23	chr16	30990620	30990620	T	A	exonic	SETD1A		synonymous SNV	SETD1A:NM_014712:exon14:c.T3513A:p.S	NOVEL		75	0	0	1	78	2	0.0794721	1	254	37	0.367148	1
23	chr2	131810545	131810545	G	A	exonic	FAM168B		synonymous SNV	FAM168B:NM_001009993:exon6:c.C519T:	NOVEL		31	0	0	1	48	1	0.0527221	1	35	6	0.338188	1
23	chr1	110038809	110038809	C	T	exonic	CYB561D1		synonymous SNV	CYB561D1:NM_001134402:exon2:c.C447T	DBSNP		155	0	0	1	165	0	0	1	117	18	0.308127	1
23	chr14	31084636	31084636	T	C	exonic	G2E3		synonymous SNV	G2E3:NM_001308097:exon13:c.T1617C:p.	NOVEL		113	0	0	1	141	0	0	1	42	5	0.307188	1
23	chr2	11767130	11767130	G	A	exonic	GREB1		nonsynonymous SNV	GREB1:NM_014668:exon25:c.G4349A:p.R	DBSNP+COSMIC	ID=COSM440967	119	15	0.271585	1	123	59	0.83747	1	141	21	0.299568	1
23	chr3	135721397	135721397	T	C	exonic	PPP2R3A		synonymous SNV	PPP2R3A:NM_002718:exon2:c.T1057C:p.L	NOVEL		509	0	0	1	560	34	0.181281	1	385	40	0.298295	1
23	chr9	8331587	8331587	A	G	exonic	PTPRD		synonymous SNV	PTPRD:NM_001171025:exon27:c.T4308C:p	DBSNP		189	11	0.16629	1	187	46	0.625261	1	168	19	0.293389	1
23	chr17	39296675	39296675	C	T	exonic	KRTAP4-6		nonsynonymous SNV	KRTAP4-6:NM_030976:exon1:c.G65A:p.R2	DBSNP+COSMIC	ID=COSM459753	138	0	0	1	192	1	0.0133854	1	138	20	0.292526	1
23	chr15	48744862	48744862	G	A	exonic	FBN1		synonymous SNV	FBN1:NM_000138:exon45:c.C5442T:p.N18	DBSNP		249	0	0	1	257	7	0.0960082	1	238	22	0.284639	1

23	chr9	138903784	138903784	G	A	exonic	NACC2		nonsynonymous SNV	NACC2:NM_144653:exon6:c.C1342T:p.R44	NOVEL		169	18	0.291028	1	179	40	0.578463	1	202	22	0.2836	1
23	chr22	51169432	51169432	G	A	exonic	SHANK3		nonsynonymous SNV	SHANK3:NM_033517:exon22:c.G4846A:p.'	NOVEL		29	0	0	1	30	15	0.861127	1	131	16	0.251532	1
23	chr10	118030397	118030397	G	A	exonic	GFRA1		nonsynonymous SNV	GFRA1:NM_145793:exon2:c.C271T:p.R91V	NOVEL		158	0	0	1	214	5	0.0818124	1	244	20	0.240735	1
23	chr19	35801012	35801012	C	T	exonic	MAG		synonymous SNV	MAG:NM_001199216:exon8:c.C1392T:p.T	DBSNP+COSMIC	ID=COSM548603	210	0	0	1	238	5	0.06197	1	172	15	0.221488	1
23	chr16	70834754	70834754	G	T	exonic	VAC14		nonsynonymous SNV	VAC14:NM_018052:exon1:c.C50A:p.A17D	NOVEL		91	0	0	1	123	0	0	1	87	7	0.215032	1
23	chr5	140208179	140208179	T	A	exonic	PCDHA6		stopgain	PCDHA6:NM_018909:exon1:c.T503A:p.L16	NOVEL		307	0	0	1	366	0	0	1	259	21	0.212712	1
23	chr6	13206144	13206144	A	T	exonic	PHACTR1		synonymous SNV	PHACTR1:NM_001322311:exon5:c.A486T:p	COSMIC	ID=COSM654709	208	3	0.0429875	1	166	40	0.614968	1	311	25	0.211024	1
23	chr19	7796190	7796190	G	A	exonic	CLEC4G		nonsynonymous SNV	CLEC4G:NM_001244856:exon3:c.C146T:p.A	NOVEL		88	1	0.0424373	1	113	0	0	1	144	11	0.209228	1
23	chr9	18929011	18929011	C	T	exonic	SAXO1		nonsynonymous SNV	SAXO1:NM_001287049:exon4:c.G269A:p.F	DBSNP+COSMIC	ID=COSM468262	252	7	0.081715	1	248	77	0.750355	1	155	12	0.20749	1
23	chr8	6605330	6605330	A	G	exonic	AGPAT5		synonymous SNV	AGPAT5:NM_018361:exon6:c.A726G:p.R2	NOVEL		232	0	0	1	184	0	0	1	155	15	0.203908	1
23	chr12	112888210	112888210	G	A	exonic	PTPN11		nonsynonymous SNV	PTPN11:NM_001330437:exon3:c.G226A:p	DBSNP+COSMIC	ID=COSM13000;	167	5	0.0705279	1	140	81	0.94685	1	117	11	0.198598	1
23	chr11	55595241	55595241	C	A	exonic	OR5L2		nonsynonymous SNV	OR5L2:NM_001004739:exon1:c.C547A:p.P	NOVEL		340	0	0	1	400	0	0	1	212	15	0.196344	1
23	chr12	53645522	53645522	G	A	exonic	MFSD5		synonymous SNV	MFSD5:NM_001170790:exon1:c.G6A:p.E2	NOVEL		131	0	0	1	126	0	0	1	120	11	0.19405	1
23	chr3	40231924	40231924	C	T	exonic	MYRIP		synonymous SNV	MYRIP:NM_001284426:exon8:c.C1074T:p.	DBSNP		143	1	0.0237928	1	123	30	0.620997	1	154	10	0.193255	1
23	chr2	69330020	69330020	C	A	exonic	ANTXR1		synonymous SNV	ANTXR1:NM_018153:exon10:c.C750A:p.R	NOVEL		63	0	0	1	85	0	0	1	55	5	0.19258	1
23	chr19	33699859	33699859	C	A	exonic	SLC7A10		stopgain	SLC7A10:NM_019849:exon11:c.G1510T:p.	NOVEL		157	1	0.0216846	1	172	1	0.0174089	1	140	10	0.184081	1
23	chr3	52255631	52255631	G	A	exonic	TLR9		nonsynonymous SNV	TLR9:NM_017442:exon2:c.C2701T:p.R901	DBSNP+COSMIC	ID=COSM122941	358	0	0	1	397	8	0.0625597	1	505	31	0.183304	1
23	chr7	100410525	100410525	C	T	exonic	EPHB4		synonymous SNV	EPHB4:NM_004444:exon12:c.G1962A:p.T	DBSNP		327	0	0	1	335	0	0	1	349	24	0.182488	1
23	chr7	130002741	130002741	G	A	exonic	CPA5		nonsynonymous SNV	CPA5:NM_001127442:exon8:c.G653A:p.G	NOVEL		133	0	0	1	186	0	0	1	207	14	0.179667	1
23	chr7	143573404	143573404	G	A	exonic	TCAF1		nonsynonymous SNV	TCAF1:NM_001206938:exon2:c.C298T:p.P	DBSNP		229	0	0	1	239	1	0.0131962	1	212	14	0.175692	1
23	chr9	90502746	90502746	G	A	exonic	SPATA31E1		nonsynonymous SNV	SPATA31E1:NM_178828:exon4:c.G3344A:	DBSNP		113	0	0	1	135	2	0.0462348	1	134	8	0.16268	1
23	chr19	15472993	15472993	T	C	exonic	AKAP8		synonymous SNV	AKAP8:NM_005858:exon10:c.A1230G:p.Q	NOVEL		356	0	0	1	366	0	0	1	260	15	0.160812	1
23	chr19	15472992	15472992	T	G	exonic	AKAP8		nonsynonymous SNV	AKAP8:NM_005858:exon10:c.A1231C:p.S4	NOVEL		356	0	0	1	361	0	0	1	262	15	0.159651	1
23	chr3	65425654	65425654	C	G	exonic	MAGI1		synonymous SNV	MAGI1:NM_001033057:exon9:c.G1170C:p	NOVEL		228	4	0.0590717	1	220	53	0.614855	1	267	14	0.157905	1
23	chr1	2451784	2451784	C	T	exonic	PANK4		nonsynonymous SNV	PANK4:NM_018216:exon5:c.G676A:p.G22	NOVEL		110	0	0	1	97	0	0	1	179	13	0.156471	1
23	chr16	2122341	2122341	C	G	exonic	TSC2		nonsynonymous SNV	TSC2:NM_001318831:exon17:c.C1597G:p.	DBSNP		105	0	0	1	131	7	0.161248	1	131	7	0.146471	1
23	chr6	26199157	26199157	C	G	exonic	HIST1H2AD		nonsynonymous SNV	HIST1H2AD:NM_021065:exon1:c.G315C:p.	NOVEL		184	1	0.016343	1	216	4	0.0575833	1	169	9	0.143402	1
23	chr19	19329749	19329749	C	T	exonic	NCAN		synonymous SNV	NCAN:NM_004386:exon3:c.C99T:p.S33S	DBSNP+COSMIC	ID=COSM310095	251	1	0.0149878	1	276	24	0.299975	1	298	15	0.141288	1
23	chr5	79442008	79442008	G	A	exonic	SERINC5		synonymous SNV	SERINC5:NM_001174071:exon11:c.C1143T	DBSNP		139	1	0.0215961	1	166	0	0	1	154	8	0.140058	1
23	chr20	47887931	47887931	G	T	exonic	ZNFX1		nonsynonymous SNV	ZNFX1:NM_021035:exon3:c.C418A:p.Q140	NOVEL		327	0	0	1	329	0	0	1	148	7	0.130406	1
23	chr15	63111803	63111803	C	T	exonic	TLN2		nonsynonymous SNV	TLN2:NM_015059:exon50:c.C6860T:p.A22	DBSNP		377	1	0.00926091	1	449	28	0.212546	1	301	12	0.128968	1
23	chr15	53889452	53889452	G	T	exonic	WDR72		nonsynonymous SNV	WDR72:NM_182758:exon18:c.C2972A:p.A	NOVEL		383	0	0	1	431	0	0	1	403	16	0.128455	1
23	chr19	39090601	39090601	C	G	exonic	MAP4K1		nonsynonymous SNV	MAP4K1:NM_001042600:exon22:c.G1633	COSMIC	ID=COSM996091	392	0	0	1	315	0	0	1	555	25	0.119018	1
23	chr11	55110968	55110968	C	T	exonic	OR4A16		stopgain	OR4A16:NM_001005274:exon1:c.C292T:p.	NOVEL		478	7	0.0505245	1	500	87	0.536655	1	558	21	0.107769	1
23	chr11	63486853	63486853	G	T	exonic	RTN3		nonsynonymous SNV	RTN3:NM_001265590:exon2:c.G543T:p.K1	NOVEL		567	8	0.0487043	1	570	101	0.520436	1	484	17	0.100824	1
23	chr8	48874954	48874954	A	T	exonic	MCM4		nonsynonymous SNV	MCM4:NM_005914:exon4:c.A489T:p.K163	NOVEL		315	5	0.0448004	1	306	3	0.0250814	1	202	8	0.0880364	1
23	chr11	499971	499971	C	T	exonic	RNH1		nonsynonymous SNV	RNH1:NM_203384:exon4:c.G301A:p.G101	DBSNP		51	0	0	1	46	9	0.51825	1	65	1	0.0450204	1
23	chr20	3644040	3644040	G	A	exonic	GFRA4		nonsynonymous SNV	GFRA4:NM_022139:exon1:c.C7T:p.R3C,GF	DBSNP		62	20	0.737428	1	102	0	0	1	134	2	0.0424642	1
23	chr4	3444490	3444490	C	A	exonic	HGFAC		nonsynonymous SNV	HGFAC:NM_001297439:exon2:c.C149A:p.T	NOVEL		154	1	0.0195062	1	151	15	0.287249	1	135	1	0.0180078	1
23	chr14	51561167	51561167	G	T	exonic	TRIM9		nonsynonymous SNV	TRIM9:NM_015163:exon1:c.C491A:p.A164	NOVEL		266	1	0.0115986	1	266	30	0.320988	1	264	1	0.0108965	1
23	chr9	140174343	140174343	A	G	exonic	TOR4A		nonsynonymous SNV	TOR4A:NM_017723:exon2:c.A1202G:p.Y4	NOVEL		148	2	0.0403127	1	174	17	0.281887	1	129	0	0	1
23	chr5	65084048	65084048	G	A	exonic	NLN		synonymous SNV	NLN:NM_020726:exon8:c.G1062A:p.G354	NOVEL		215	1	0.0139975	1	241	22	0.264927	1	133	0	0	1
23	chr12	305357	305357	C	T	exonic	SLC6A12		nonsynonymous SNV	SLC6A12:NM_001206931:exon11:c.G1259	DBSNP		95	0	0	1	85	9	0.247345	1	61	0	0	1
23	chr11	11924821	11924821	C	T	exonic	USP47		nonsynonymous SNV	USP47:NM_017944:exon6:c.C547T:p.H183	DBSNP		357	0	0	1	334	17	0.153392	1	296	0	0	1
23	chr19	52825018	52825018	C	T	exonic	ZNF480		nonsynonymous SNV	ZNF480:NM_001297624:exon4:c.C386T:p.	NOVEL		247	78	0.822278	1	290	15	0.148118	1	98	0	0	1
23	chr1	248058913	248058913	C	A	exonic	OR2W3		nonsynonymous SNV	OR2W3:NM_001001957:exon1:c.C25A:p.Q	COSMIC	ID=COSM380299	318	0	0	1	354	15	0.129223	1	293	0	0	1
23	chr17	12666491	12666491	G	A	exonic	MYOCD		nonsynonymous SNV	MYOCD:NM_153604:exon13:c.G2347A:p.A	NOVEL		191	0	0	1	219	10	0.112811	1	166	0	0	1
23	chr2	219824876	219824876	C	T	exonic	CDK5R2		nonsynonymous SNV	CDK5R2:NM_003936:exon1:c.C334T:p.P11	NOVEL		65	23	0.63411	1	112	5	0.110401	1	152	0	0	1
23	chr19	4548284	4548284	C	G	exonic	SEMA6B		nonsynonymous SNV	SEMA6B:NM_032108:exon13:c.G1445C:p.	NOVEL		526	1	0.00716683	1	506	15	0.107957	1	608	0	0	1
23	chr3	122631118	122631118	G	A	exonic	SEMA5B		nonsynonymous SNV	SEMA5B:NM_001031702:exon19:c.C2797T	COSMIC	ID=COSM203033	143	14	0.305517	1	155	5	0.0989714	1	239	0	0	1
23	chr7	140453155	140453155	C	T	exonic	BRAF		nonsynonymous SNV	BRAF:NM_001354609:exon15:c.G1780A:p	DBSNP+COSMIC	ID=COSM27639;	98	20	0.51245	1	127	4	0.0967048	1	96	0	0	1
23	chr5	150029420	150029420	C	T	exonic	SYNPO		nonsynonymous SNV	SYNPO:NM_001109974:exon2:c.C1583T:p.	NOVEL		110	32	0.681342	1	176	5	0.0874885	1	337	0	0	1
23	chr17	10317332	10317332	G	C	exonic	MYH8		nonsynonymous SNV	MYH8:NM_002472:exon12:c.C1034G:p.T3	DBSNP		151	49	0.594409	1	237	8	0.0843553	1	205	0	0	1
23	chr9	104449348	104449348	G	A	exonic	GRIN3A		synonymous SNV	GRIN3A:NM_133445:exon2:c.C834T:p.D27	NOVEL		254	63	0.600876	1	340	9	0.0816726	1	280	0	0	1
23	chr17	47295239	47295239	A	T	exonic	ABI3		nonsynonymous SNV	ABI3:NM_001135186:exon3:c.A406T:p.N1	NOVEL		126	10	0.178394	1	168	5	0.0746642	1	189	0	0	1
23	chr8	27634611	27634611	G	C	exonic	ESCO2		nonsynonymous SNV	ESCO2:NM_001017420:exon3:c.G786C:p.K	NOVEL		201	19	0.247624	1	188	3	0.0405767	1	85	0	0	1
23	chr19	8152732	8152732	C	T	exonic	FBN3		nonsynonymous SNV	FBN3:NM_001321431:exon53:c.G6596A:p	DBSNP+COSMIC	ID=COSM148168	177	26	0.483743	1	226	2	0.032892	1	178	0	0	1
23	chr13	32914533	32914533	T	C	exonic	BRCA2		nonsynonymous SNV	BRCA2:NM_000059:exon11:c.T6041C:p.V2	NOVEL		106	30	0.535182	1	181	2	0.0282337	1	74	0	0	1
23	chr5	74645899	74645899	G	A	exonic	HMGCR		nonsynonymous SNV	HMGCR:NM_000859:exon7:c.G589A:p.G1	NOVEL		518	15	0.0850878	1	615	5	0.025541	1	441	0	0	1
23	chr10	50374957	50374957	G	A	exonic	TMEM273		synonymous SNV	TMEM273:NM_001010863:exon3:c.C195T	DBSNP+COSMIC	ID=COSM687331	91	17	0.412592	1	161	1	0.0221196	1	216	0	0	1
23	chr2	95954729	95954729	C	A	exonic	PROM2		synonymous SNV	PROM2:NM_001165977:exon23:c.C2490A	NOVEL		114	20	0.362113	1	161	1	0.0159468	1	224	0	0	1
23	chr12	55714427	55714427	T	G	exonic	OR6C1		stopgain	OR6C1:NM_001005182:exon1:c.T44G:p.L1	NOVEL		97	28	0.54346	1	144	0	0	1	180	0	0	1
23	chr3	3081889	3081889	G	T	exonic	CNTN4		nonsynonymous SNV	CNTN4:NM_001206956:exon11:c.G1345T:	DBSNP		147	25	0.497988	1	198	0	0	1	168	0	0	1
23	chr10	123843558	123843558	C	T	exonic	TACC2		nonsynonymous SNV	TACC2:NM_001291876:exon4:c.C1543T:p.	NOVEL		157	18	0.269606	1	221	0	0	1	490	0	0	1
23	chr2	226446937	226446937	C	T	exonic	NYAP2		synonymous SNV	NYAP2:NM_020864:exon4:c.C804T:p.Y268	DBSNP+COSMIC	ID=COSM245255	194	20	0.226744	1	293	0	0	1	300	0	0	1
23	chr1	9098885	9098885	A	T	splicing	SLC2A5	NM_003039:exon9:c.1			NOVEL		49	0	0	1	50	0	0	1	53	7	0.269611	1
24	chr10	115805165	115805165	G	A	exonic	ADRB1		nonsynonymous SNV	ADRB1:NM_000684:exon1:c.G1274A:p.G4	NOVEL		40	42	1.11487	1	48	45	1.42923	1				
24	chr12	52908739	52908739	C	A	exonic	KRT5		nonsynonymous SNV	KRT5:NM_000424:exon9:c.G1760T:p.S587	NOVEL		16	19	1.18161	1	32	25	1.2955	1				
24	chr8	101225478	101225478	A	G	exonic	SPAG1		synonymous SNV	SPAG1:NM_003114:exon11:c.A1257G:p.P	NOVEL		29	34	1.1747	1	57	41	1.23575	1				
24	chr6	150238116	150238116	G	T	exonic	RAET1G		nonsynonymous SNV	RAET1G:NM_001001788:exon5:c.C937A:p	NOVEL		24	19	0.961776	1	32	22	1.20338	1				
24	chr17	72366733	72366733	G	A	exonic	GPR142		stopgain	GPR142:NM_001331076:exon3:c.G168A:p	NOVEL		45	38	0.996539	1	83	57	1.20259	1				
24	chr3	193160304	193160304	C	G	exonic	ATP13A4		nonsynonymous SNV	ATP13A4:NM_032279:exon19:c.G2194C:p.	NOVEL		39	40	1.1021	1	62	38	1.12242	1				
24	chr15	40583227	40583227	C	T	exonic	PLCB2		nonsynonymous SNV	PLCB2:NM_001284298:exon26:c.G2983A:p	NOVEL		10	14	1.26971	1	18	11	1.12038	1				
24	chr2	102486807	102486807	T	C	exonic	MAP4K4		nonsynonymous SNV	MAP4K4:NM_001242559:exon21:c.T2447C	NOVEL		89	68	0.942754	1	113	69	1.11982	1				
24	chr4	85708849	85708849	A	C	exonic	WDFY3		nonsynonymous SNV	WDFY3:NM_014991:exon23:c.T3687G:p.S	NOVEL		156	3	0.0410689	1	93	55	1.09767	1				
24	chr15	64967876	64967876	C	T	exonic	ZNF609		synonymous SNV	ZNF609:NM_015042:exon4:c.C2823T:p.N9	DBSNP		84	75	1.02672	1	112	66	1.09521	1				
24	chr19	16664651	16664651	T	C	exonic	SLC35E1		nonsynonymous SNV	SLC35E1:NM_024881:exon6:c.A1072G:p.K	NOVEL		54	90	0.992704	2	80	99	1.09335	2				
24	chr9	13136723	13136723	A	C	exonic	MPDZ		nonsynonymous SNV	MPDZ:NM_001261406:exon29:c.T4181G:p	NOVEL		29	87	1.19124	2	75	90	1.07829	2				
24	chr4	187454906	187454906	C	T	exonic	MTNR1A		synonymous SNV	MTNR1A:NM_005958:exon2:c.G990A:p.R3	NOVEL		49	43	1.01735	1	90	50	1.05491	1				
24	chr1	1534749	1534749	G	C	exonic	FNDC10		nonsynonymous SNV	FNDC10:NM_001242659:exon1:c.C647G:p	DBSNP		24	30	1.20925	1	58	32	1.05022	1				
24	chr16	11979099	11979099	A	C	exonic	GSPT1		nonsynonymous SNV	GSPT1:NM_001130006:exon10:c.T1283G:p	COSMIC	ID=COSM381719	70	48	0.885418	1	98	54	1.04935	1				
24	chr15	51692574	51692574	C	T	exonic	GLDN		synonymous SNV	GLDN:NM_001330297:exon8:c.C534T:p.S1	DBSNP		143	129	1.03231	1	227	123	1.03803	1				
24	chr12	22680823	22680823	C	T	exonic	C2CD5		nonsynonymous SNV	C2CD5:NM_001286174:exon3:c.G181A:p.D	NOVEL		96	6	0.128038	1	72	38	1.02038	1				
24	chr19	9067698	9067698	G	C	exonic	MUC16		nonsynonymous SNV	MUC16:NM_024690:exon3:c.C19748G:p.T	DBSNP		165	59	0.836707	1	211	73	1.01628	1				
24	chr19	42840352	42840352	C	T	exonic	MEGF8		synonymous SNV	MEGF8:NM_001271938:exon6:c.C1098T:p	NOVEL		146	67	0.999229	1	171	59	1.01422	1				
24	chr1	200567477	200567477	A	G	exonic	KIF14		nonsynonymous SNV	KIF14:NM_001305792:exon12:c.T964C:p.S	NOVEL		50	41	0.980689	1	54	28	1.00859	1				
24	chr4	7985001	7985001	G	A	exonic	ABLIM2		nonsynonymous SNV	ABLIM2:NM_001286688:exon14:c.C917T:p	DBSNP		98	84	1.00461	1	149	77	1.00636	1				
24	chr14	23777334	23777334	G	T	exonic	BCL2L2;BCL2L2-PA		stopgain	BCL2L2:NM_001199839:exon3:c.G358T:p.	NOVEL		52	54	1.10886	1	94	48	0.998447	1				
24	chr18	74153594	74153594	C	T	exonic	ZNF516		nonsynonymous SNV	ZNF516:NM_014643:exon3:c.G1417A:p.A	DBSNP		46	59	1.22307	1	92	46	0.98458	1				
24	chr1	208390955	208390955	C	T	exonic	PLXNA2		nonsynonymous SNV	PLXNA2:NM_025179:exon2:c.G313A:p.E10	DBSNP		86	81	1.05574	1	146	73	0.98458	1				
24	chr17	73569700	73569700	C	T	exonic	LLGL2		nonsynonymous SNV	LLGL2:NM_001031803:exon21:c.C2864T:p	DBSNP		58	54	1.04946	1	99	49	0.977927	1				
24	chr11	1156629	1156629	G	C	exonic	MUC5AC		unknown	UNKNOWN	NOVEL		102	56	1.1259	1	137	45	0.977573	1				
24	chrX	153631658	153631658	G	A	exonic	DNASE1L1		synonymous SNV	DNASE1L1:NM_001009934:exon6:c.C477T	DBSNP+COSMIC	ID=COSM544791	120	107	1.026	1	158	77	0.967821	1				
24	chr12	21791327	21791327	T	A	exonic	LDHB		nonsynonymous SNV	LDHB:NM_001174097:exon6:c.A673T:p.S2	NOVEL		33	39	1.17902	1	64	31	0.963852	1				
24	chrX	107224714	107224714	C	T	exonic	TEX13B		nonsynonymous SNV	TEX13B:NM_031273:exon3:c.G535A:p.E17	NOVEL		150	4	0.0565364	1	112	53	0.948777	1				
24	chr11	64875828	64875828	T	C	exonic	VPS51		synonymous SNV	VPS51:NM_013265:exon5:c.T885C:p.P295	NOVEL		45	63	0.926523	2	77	69	0.934274	2				
24	chr16	69369071	69369071	G	A	exonic	COG8		nonsynonymous SNV	COG8:NM_032382:exon3:c.C766T:p.R256V	DBSNP		90	71	0.95989	1	126	58	0.93107	1				
24	chr12	47471531	47471531	G	A	exonic	AMIGO2		synonymous SNV	AMIGO2:NM_181847:exon2:c.C1255T:p.L	COSMIC	ID=COSM659924	290	11	0.0795454	1	283	128	0.919899	1				
24	chr18	44109152	44109152	G	A	exonic	LOXHD1		synonymous SNV	LOXHD1:NM_001308013:exon9:c.C897T:p.	NOVEL		113	2	0.0378548	1	108	46	0.882286	1				
24	chr13	109792965	109792965	G	A	exonic	MYO16		nonsynonymous SNV	MYO16:NM_001198950:exon32:c.G4405A	NOVEL		88	3	0.0717578	1	92	39	0.879358	1				
24	chr16	81293316	81293316	G	A	exonic	BCO1		nonsynonymous SNV	BCO1:NM_017429:exon3:c.G229A:p.D77N	DBSNP+COSMIC	ID=COSM269324	245	11	0.093528	1	197	83	0.875573	1				
24	chr9	127572227	127572227	T	G	exonic	OLFML2A		nonsynonymous SNV	OLFML2A:NM_001282715:exon5:c.T853G:	NOVEL		446	13	0.0899705	1	413	117	0.872807	1				
24	chrX	22291809	22291809	A	T	exonic	CBLL2		nonsynonymous SNV	CBLL2:NM_152577:exon1:c.A701T:p.K234I	NOVEL		476	16	0.0707854	1	362	148	0.857164	1				
24	chr5	176827487	176827487	C	T	exonic	PFN3		nonsynonymous SNV	PFN3:NM_001029886:exon1:c.G91A:p.V31	NOVEL		77	1	0.0279058	1	81	33	0.85503	1				
24	chr6	74111703	74111703	A	G	exonic	DDX43		synonymous SNV	DDX43:NM_018665:exon4:c.A558G:p.T186	NOVEL		285	5	0.0375285	1	268	107	0.8428	1				
24	chrX	152771517	152771517	G	A	exonic	BGN		nonsynonymous SNV	BGN:NM_001711:exon4:c.G548A:p.R183Q	DBSNP		141	2	0.0304427	1	113	45	0.841255	1				
24	chr19	44351810	44351810	G	A	exonic	ZNF283		nonsynonymous SNV	ZNF283:NM_001297752:exon6:c.G640A:p	NOVEL		216	3	0.0435158	1	215	57	0.828541	1				
24	chr9	133942352	133942352	C	T	exonic	LAMC3		nonsynonymous SNV	LAMC3:NM_006059:exon14:c.C2353T:p.R7	DBSNP+COSMIC	ID=COSM469737	208	4	0.0599368	1	180	47	0.818616	1				
24	chr12	25398282	25398282	C	A	exonic	KRAS		nonsynonymous SNV	KRAS:NM_001369786:exon2:c.G37T:p.G13	DBSNP+COSMIC	ID=COSM115250	172	5	0.0614873	1	147	56	0.814825	1				
24	chr11	71948549	71948549	C	T	exonic	INPPL1		synonymous SNV	INPPL1:NM_001567:exon26:c.C3261T:p.G	NOVEL		139	2	0.0450589	1	133	34	0.804953	1				
24	chr2	27360320	27360320	C	T	exonic	PRR30		nonsynonymous SNV	PRR30:NM_178553:exon3:c.G878A:p.R29	DBSNP		100	1	0.021551	1	99	37	0.803591	1				
24	chr3	97605590	97605590	C	T	exonic	CRYBG3		nonsynonymous SNV	CRYBG3:NM_153605:exon8:c.C7268T:p.S2	NOVEL		144	3	0.0444215	1	143	53	0.798715	1				
24	chr6	84798861	84798861	C	T	exonic	MRAP2		synonymous SNV	MRAP2:NM_001346541:exon3:c.C21T:p.D	NOVEL		140	5	0.075057	1	114	42	0.795238	1				
24	chr20	47710703	47710703	T	C	exonic	CSE1L		nonsynonymous SNV	CSE1L:NM_001256135:exon22:c.T2306C:p	NOVEL		73	2	0.0580441	1	66	24	0.787664	1				
24	chr14	103596129	103596129	G	A	exonic	TNFAIP2		nonsynonymous SNV	TNFAIP2:NM_006291:exon3:c.G913A:p.G3	DBSNP		78	6	0.155475	1	95	34	0.778505	1				
24	chr3	127396576	127396576	G	T	exonic	ABTB1		stopgain	ABTB1:NM_032548:exon10:c.G493T:p.E16	NOVEL		120	2	0.0356828	1	94	33	0.767507	1				
24	chr19	36357115	36357115	C	T	exonic	KIRREL2		synonymous SNV	KIRREL2:NM_001329530:exon14:c.C1698T	DBSNP+COSMIC	ID=COSM685968	27	21	1.38979	1	42	10	0.760335	1				
24	chr20	61959779	61959779	C	T	exonic	COL20A1		nonsynonymous SNV	COL20A1:NM_020882:exon34:c.C3710T:p.	DBSNP		108	3	0.0588284	1	105	36	0.754146	1				
24	chr2	95954006	95954006	C	A	exonic	PROM2		nonsynonymous SNV	PROM2:NM_001165977:exon21:c.C2292A	NOVEL		179	7	0.081917	1	154	49	0.712972	1				
24	chr18	77624080	77624080	C	T	exonic	KCNG2		nonsynonymous SNV	KCNG2:NM_012283:exon1:c.C413T:p.T138	NOVEL		11	0	0	1	22	6	0.632944	1				
24	chr13	73337656	73337656	A	T	exonic	DIS3		nonsynonymous SNV	DIS3:NM_001322348:exon15:c.T1691A:p.L	NOVEL		152	3	0.0421287	1	89	33	0.569284	1				
24	chrX	21667016	21667016	T	C	exonic	CNKSR2		synonymous SNV	CNKSR2:NM_001330772:exon19:c.T2523C	NOVEL		106	0	0	1	96	22	0.550697	1				
24	chr8	27779338	27779338	G	A	exonic	SCARA5		synonymous SNV	SCARA5:NM_173833:exon4:c.C666T:p.D22	DBSNP		44	3	0.138935	1	55	12	0.529028	1				
24	chr6	130399711	130399711	C	A	exonic	L3MBTL3		nonsynonymous SNV	L3MBTL3:NM_001007102:exon13:c.C1178	NOVEL		174	3	0.0368924	1	186	38	0.501081	1				
24	chr21	43702465	43702465	C	T	exonic	ABCG1		nonsynonymous SNV	ABCG1:NM_004915:exon6:c.C670T:p.R224	DBSNP		92	0	0	1	95	17	0.448335	1				
24	chrX	47072456	47072456	T	G	exonic	UBA1		nonsynonymous SNV	UBA1:NM_003334:exon23:c.T2714G:p.V9	NOVEL		136	0	0	1	145	25	0.434373	1				
24	chr3	172061943	172061943	C	T	exonic	FNDC3B		synonymous SNV	FNDC3B:NM_001135095:exon19:c.C2145T	NOVEL		182	0	0	1	176	25	0.367381	1				
24	chr11	3249902	3249902	C	A	exonic	MRGPRE		nonsynonymous SNV	MRGPRE:NM_001039165:exon2:c.G128T:p	COSMIC	ID=COSM361537	230	0	0	1	256	21	0.299742	1				
24	chrX	26212258	26212258	G	A	exonic	MAGEB6		nonsynonymous SNV	MAGEB6:NM_173523:exon2:c.G295A:p.D9	NOVEL		92	81	1.01913	1	173	18	0.278363	1				
24	chr18	67727209	67727209	C	T	exonic	RTTN		nonsynonymous SNV	RTTN:NM_001318520:exon35:c.G2081A:p	NOVEL		156	0	0	1	178	18	0.271262	1				
24	chr20	23016491	23016491	G	T	exonic	SSTR4		nonsynonymous SNV	SSTR4:NM_001052:exon1:c.G371T:p.S124	NOVEL		310	0	0	1	318	31	0.262367	1				
24	chr16	83065693	83065693	G	T	exonic	CDH13		nonsynonymous SNV	CDH13:NM_001220489:exon3:c.G236T:p.G	NOVEL		118	0	0	1	113	11	0.262025	1				
24	chrX	118694303	118694303	C	T	exonic	CXorf56		nonsynonymous SNV	CXorf56:NM_001170569:exon2:c.G23A:p.R	DBSNP		99	107	1.13059	1	200	19	0.256261	1				
24	chr3	159997145	159997145	T	C	exonic	IFT80		nonsynonymous SNV	IFT80:NM_001190242:exon15:c.A1261G:p	NOVEL		86	65	0.936969	1	137	12	0.237885	1				
24	chr2	163374503	163374503	G	T	exonic	KCNH7		nonsynonymous SNV	KCNH7:NM_033272:exon4:c.C629A:p.P210	NOVEL		124	97	0.955363	1	230	20	0.236299	1				
24	chr4	128651770	128651770	C	G	exonic	SLC25A31		nonsynonymous SNV	SLC25A31:NM_001318467:exon1:c.C70G:p	DBSNP		127	88	0.890909	1	252	21	0.227211	1				
24	chr7	100686679	100686679	C	T	exonic	MUC17		synonymous SNV	MUC17:NM_001040105:exon3:c.C11982T:	DBSNP+COSMIC	ID=COSM671772	158	101	0.84881	1	313	26	0.226541	1				
24	chr15	78921643	78921643	C	T	exonic	CHRNB4		nonsynonymous SNV	CHRNB4:NM_000750:exon5:c.G1004A:p.R	DBSNP		88	58	0.864697	1	182	15	0.224904	1				
24	chr6	160492966	160492966	G	T	exonic	IGF2R		nonsynonymous SNV	IGF2R:NM_000876:exon32:c.G4468T:p.A1	NOVEL		76	71	1.05131	1	166	12	0.199129	1				
24	chr3	160968685	160968685	C	T	exonic	NMD3		nonsynonymous SNV	NMD3:NM_015938:exon16:c.C1499T:p.S5	NOVEL		159	146	1.04194	1	310	22	0.19573	1				
24	chr5	167689572	167689572	C	A	exonic	TENM2		synonymous SNV	TENM2:NM_001080428:exon25:c.C7365A:	NOVEL		60	73	1.1947	1	118	8	0.187539	1				
24	chr5	33989551	33989551	C	T	exonic	AMACR		nonsynonymous SNV	AMACR:NM_001167595:exon5:c.G796A:p.	NOVEL		258	0	0	1	268	18	0.1859	1				
24	chr12	25380275	25380275	T	G	exonic	KRAS		nonsynonymous SNV	KRAS:NM_001369786:exon3:c.A183C:p.Q6	DBSNP+COSMIC	ID=COSM554,CO	50	41	0.980689	1	140	9	0.178414	1				
24	chr4	6606978	6606978	A	G	exonic	MAN2B2		nonsynonymous SNV	MAN2B2:NM_001292038:exon11:c.A1583	NOVEL		60	53	1.02091	1	150	9	0.167193	1				
24	chr10	119003710	119003710	G	T	exonic	SLC18A2		nonsynonymous SNV	SLC18A2:NM_003054:exon3:c.G350T:p.C1	NOVEL		113	86	0.940664	1	234	14	0.166743	1				
24	chr11	433877	433877	G	A	exonic	ANO9		nonsynonymous SNV	ANO9:NM_001012302:exon3:c.C142T:p.P4	NOVEL		131	51	0.890161	1	219	9	0.156069	1				
24	chr4	77662879	77662879	C	T	exonic	SHROOM3		nonsynonymous SNV	SHROOM3:NM_020859:exon5:c.C3553T:p.	DBSNP		78	59	0.93739	1	111	6	0.151474	1				
24	chr19	21299686	21299686	G	A	exonic	ZNF714		synonymous SNV	ZNF714:NM_182515:exon5:c.G216A:p.L72	COSMIC	ID=COSM710905	444	79	0.479838	1	597	22	0.140521	1				
24	chr2	198266711	198266711	T	C	exonic	SF3B1		nonsynonymous SNV	SF3B1:NM_012433:exon15:c.A2221G:p.K7	COSMIC	ID=COSM541958	122	85	0.893794	1	248	12	0.136326	1				
24	chr12	121701700	121701700	C	T	exonic	CAMKK2		nonsynonymous SNV	CAMKK2:NM_001270486:exon5:c.G668A:p	DBSNP		98	0	0	1	128	6	0.132257	1				
24	chr19	4328716	4328716	G	A	exonic	STAP2		synonymous SNV	STAP2:NM_001013841:exon6:c.C546T:p.D	NOVEL		43	20	1.00846	1	58	2	0.131791	1				
24	chr11	45265717	45265717	C	T	exonic	SYT13		synonymous SNV	SYT13:NM_020826:exon6:c.G1167A:p.A38	DBSNP		72	29	0.912108	1	117	4	0.130702	1				
24	chr16	72822652	72822652	A	G	exonic	ZFHX3		nonsynonymous SNV	ZFHX3:NM_001164766:exon9:c.T6781C:p.S	NOVEL		54	52	1.06779	1	118	5	0.120071	1				
24	chr11	50003983	50003983	G	T	exonic	OR4C12		nonsynonymous SNV	OR4C12:NM_001005270:exon1:c.C55A:p.P	COSMIC	ID=COSM419308	228	76	0.794163	1	340	10	0.112964	1				
24	chr8	42294726	42294726	C	A	exonic	SLC20A2		nonsynonymous SNV	SLC20A2:NM_001257180:exon8:c.G1304T	NOVEL		78	47	0.818421	1	165	6	0.10364	1				
24	chr2	220505683	220505683	C	T	exonic	SLC4A3		nonsynonymous SNV	SLC4A3:NM_001326559:exon22:c.C3701T:	DBSNP		49	27	0.773284	1	84	3	0.101853	1				
24	chr5	146889078	146889078	C	G	exonic	DPYSL3		nonsynonymous SNV	DPYSL3:NM_001197294:exon1:c.G344C:p.	NOVEL		29	18	0.833611	1	46	0	0	1				
24	chr9	133266842	133266842	A	C	exonic	HMCN2		unknown	UNKNOWN	NOVEL		81	14	0.468138	1	105	0	0	1				
24	chr8	103269859	103269859	C	A	splicing	UBR5	NM_015902:exon58:c.			COSMIC	ID=COSM417018	74	56	0.937635	1	151	12	0.217453	1				
25	chrX	9863916	9863916	G	A	exonic	SHROOM2		synonymous SNV	SHROOM2:NM_001649:exon4:c.G1968A:p	NOVEL		54	66	NA	1	10	121	NA	1	18	87	NA	1
25	chrX	47039873	47039873	G	C	exonic	RBM10		nonsynonymous SNV	RBM10:NM_001204466:exon11:c.G985C:p	NOVEL		33	0	NA	1	24	1	NA	1	38	17	NA	1
25	chr2	131801885	131801885	G	A	exonic	ARHGEF4		nonsynonymous SNV	ARHGEF4:NM_001367493:exon12:c.G517	DBSNP+COSMIC	ID=COSM430352	80	62	0.998325	1	61	63	1.22694	1	70	61	1.32886	1
25	chr2	229890461	229890461	C	T	exonic	PID1		nonsynonymous SNV	PID1:NM_001330157:exon2:c.G394A:p.A1	DBSNP+COSMIC	ID=COSM442441	195	72	0.616581	1	171	145	1.10811	1	137	117	1.31454	1
25	chr1	151384211	151384211	C	T	exonic	POGZ		nonsynonymous SNV	POGZ:NM_145796:exon10:c.G1531A:p.D5	NOVEL		85	61	0.955313	1	74	63	1.11051	1	76	57	1.22305	1
25	chr16	1033863	1033863	C	T	exonic	SOX8		synonymous SNV	SOX8:NM_014587:exon2:c.C558T:p.S186S	DBSNP+COSMIC	ID=COSM547367	154	99	0.894712	1	134	125	1.1655	1	208	155	1.21856	1
25	chr10	101421276	101421276	C	T	exonic	ENTPD7		nonsynonymous SNV	ENTPD7:NM_001349963:exon3:c.C82T:p.R	DBSNP		68	80	1.23594	1	94	89	1.17447	1	75	53	1.18165	1
25	chr3	10379965	10379965	C	T	exonic	ATP2B2		synonymous SNV	ATP2B2:NM_001330611:exon19:c.G3180A	DBSNP+COSMIC	ID=COSM391442	190	86	1.02405	1	156	95	1.2925	1	238	105	1.17973	1
25	chr14	75230798	75230798	G	A	exonic	YLPM1		synonymous SNV	YLPM1:NM_019589:exon1:c.G606A:p.A20	NOVEL		133	107	1.01939	1	144	114	1.06706	1	235	165	1.17719	1
25	chr19	55870062	55870062	G	T	exonic	FAM71E2		nonsynonymous SNV	FAM71E2:NM_001145402:exon9:c.C2174	NOVEL		330	206	0.827785	2	294	149	1.14249	1	233	102	1.17339	1
25	chr11	6977749	6977749	G	A	exonic	ZNF215		nonsynonymous SNV	ZNF215:NM_001354858:exon5:c.G827A:p	DBSNP		205	52	0.66497	1	205	104	1.14936	1	143	60	1.13905	1
25	chr11	78775932	78775932	G	C	exonic	TENM4		nonsynonymous SNV	TENM4:NM_001098816:exon6:c.C344G:p.	NOVEL		67	99	0.980007	2	82	103	0.950641	2	70	95	1.10942	2
25	chr5	9063094	9063094	T	G	exonic	SEMA5A		nonsynonymous SNV	SEMA5A:NM_003966:exon18:c.A2423C:p.	NOVEL		153	242	1.00675	2	157	272	1.08259	2	160	213	1.10035	2
25	chr2	112705129	112705129	G	A	exonic	MERTK		nonsynonymous SNV	MERTK:NM_006343:exon4:c.G742A:p.V24	DBSNP+COSMIC	ID=COSM139879	62	46	0.973874	1	54	48	1.13643	1	45	28	1.09461	1
25	chr1	115256529	115256529	T	C	exonic	NRAS		nonsynonymous SNV	NRAS:NM_002524:exon3:c.A182G:p.Q61R	DBSNP+COSMIC	ID=COSM584;OC	98	89	1.08822	1	129	89	0.985909	1	113	70	1.09161	1
25	chr5	45645369	45645369	A	C	exonic	HCN1		nonsynonymous SNV	HCN1:NM_021072:exon2:c.T767G:p.I256S	NOVEL		123	227	1.06576	2	143	200	0.995604	2	119	155	1.09003	2
25	chr12	111758297	111758297	C	T	exonic	CUX2		synonymous SNV	CUX2:NM_001370598:exon17:c.C2298T:p.	COSMIC	ID=COSM467565	85	84	1.13648	1	97	63	0.950876	1	80	49	1.084	1
25	chr14	38296447	38296447	A	G	exonic	TTC6		nonsynonymous SNV	TTC6:NM_001368142:exon11:c.A1076G:p.	NOVEL		251	185	0.970184	1	301	233	1.0537	1	235	142	1.0749	1
25	chr3	5024774	5024774	G	A	exonic	BHLHE40		synonymous SNV	BHLHE40:NM_003670:exon5:c.G636A:p.S2	DBSNP		541	136	0.66021	1	513	251	1.12192	1	501	193	1.07173	1
25	chr16	31228267	31228267	C	T	exonic	PYDC1		nonsynonymous SNV	PYDC1:NM_152901:exon1:c.G83A:p.G28E	NOVEL		174	126	0.960325	1	178	116	0.952827	1	243	145	1.06649	1
25	chr3	154864987	154864987	A	T	exonic	MME		nonsynonymous SNV	MME:NM_000902:exon15:c.A1471T:p.N49	NOVEL		206	97	1.05211	1	216	103	1.10262	1	159	59	1.043	1
25	chr15	75042314	75042314	C	T	exonic	CYP1A2		nonsynonymous SNV	CYP1A2:NM_000761:exon2:c.C235T:p.R79	DBSNP+COSMIC	ID=COSM173726	395	134	1.08418	1	357	84	0.837836	1	319	92	1.0367	1
25	chr20	44572047	44572047	G	A	exonic	PCIF1		nonsynonymous SNV	PCIF1:NM_022104:exon9:c.G890A:p.R297	COSMIC	ID=COSM674141	50	46	1.09561	1	74	44	0.90048	1	43	24	1.02225	1
25	chr19	10395086	10395086	G	A	exonic	ICAM1		synonymous SNV	ICAM1:NM_000201:exon5:c.G933A:p.P311	DBSNP		388	387	1.06864	2	404	371	1.05284	2	412	316	1.00515	2
25	chr5	149546443	149546443	T	C	exonic	CDX1		nonsynonymous SNV	CDX1:NM_001804:exon1:c.T4C:p.Y2H	NOVEL		55	107	1.08535	2	47	88	1.11301	2	106	113	0.994243	2
25	chr15	50884776	50884776	T	A	exonic	TRPM7		nonsynonymous SNV	TRPM7:NM_001301212:exon26:c.A3656T:	NOVEL		248	231	1.03205	2	234	240	1.11358	2	121	90	0.987727	2
25	chr5	54945126	54945126	A	C	exonic	SLC38A9		synonymous SNV	SLC38A9:NM_001258286:exon9:c.T780G:p	NOVEL		158	57	0.871301	1	156	63	0.982375	1	137	47	0.984392	1
25	chr5	134074399	134074399	A	C	exonic	CAMLG		nonsynonymous SNV	CAMLG:NM_001745:exon1:c.A89C:p.E30A	NOVEL		111	57	1.11506	1	114	47	0.996903	1	140	46	0.953088	1
25	chr9	140358614	140358614	C	T	exonic	PNPLA7		nonsynonymous SNV	PNPLA7:NM_152286:exon27:c.G3115A:p.D	DBSNP		208	40	0.690339	1	192	69	1.16286	1	140	36	0.94732	1
25	chr3	69169001	69169001	T	G	exonic	LMOD3		nonsynonymous SNV	LMOD3:NM_198271:exon2:c.A505C:p.T16	NOVEL		303	75	0.652081	1	264	94	0.896656	1	205	66	0.938562	1
25	chr3	135870047	135870047	G	A	exonic	MSL2		nonsynonymous SNV	MSL2:NM_001145417:exon2:c.C1454T:p.A	NOVEL		409	147	0.868909	1	401	170	1.0167	1	331	105	0.928092	1
25	chr7	92979318	92979318	C	G	exonic	VPS50		nonsynonymous SNV	VPS50:NM_017667:exon25:c.C2436G:p.H8	NOVEL		241	120	1.09246	1	280	113	0.981899	1	231	70	0.896231	1
25	chr12	63182013	63182013	C	T	exonic	PPM1H		nonsynonymous SNV	PPM1H:NM_020700:exon4:c.G862A:p.D28	NOVEL		84	63	0.979923	1	73	79	1.25512	1	83	37	0.879919	1
25	chr9	134003012	134003012	T	G	exonic	NUP214		synonymous SNV	NUP214:NM_001318324:exon2:c.T147G:p	NOVEL		495	160	1.04552	1	490	151	1.03619	1	313	72	0.866121	1
25	chr9	79974272	79974272	A	G	exonic	VPS13A		nonsynonymous SNV	VPS13A:NM_001018037:exon57:c.A7942G	NOVEL		267	74	0.92882	1	269	69	0.897947	1	219	48	0.832601	1
25	chr8	131200393	131200393	C	T	exonic	ASAP1		nonsynonymous SNV	ASAP1:NM_001362924:exon6:c.G442A:p.D	DBSNP		111	76	0.52285	1	92	169	NA	1	109	75	0.755622	1
25	chr8	131200418	131200418	C	G	exonic	ASAP1		nonsynonymous SNV	ASAP1:NM_001362924:exon6:c.G417C:p.L	NOVEL		112	84	0.551352	1	104	196	NA	1	137	93	0.749577	1
25	chr19	32949299	32949299	C	T	exonic	DPY19L3		synonymous SNV	DPY19L3:NM_001172774:exon12:c.C1206T	DBSNP		416	95	0.696169	1	334	137	0.9933	1	218	52	0.679309	1
25	chr8	145773263	145773263	G	A	exonic	ARHGAP39		stopgain	ARHGAP39:NM_025251:exon4:c.C1207T:p	NOVEL		218	0	0	1	226	0	0	1	206	42	0.584615	1
25	chr6	161508750	161508750	A	C	exonic	MAP3K4		nonsynonymous SNV	MAP3K4:NM_001301072:exon10:c.A2587C	NOVEL		149	0	0	1	161	5	0.0727386	1	69	15	0.509606	1
25	chr16	89831393	89831393	G	A	exonic	FANCA		nonsynonymous SNV	FANCA:NM_000135:exon28:c.C2683T:p.P8	DBSNP		274	0	0	1	302	9	0.0698852	1	227	44	0.463346	1
25	chr19	4234705	4234705	G	A	exonic	EBI3		nonsynonymous SNV	EBI3:NM_005755:exon4:c.G421A:p.A141T	DBSNP		395	0	0	1	391	4	0.0445432	1	438	46	0.440169	1
25	chr11	56510357	56510357	C	A	exonic	OR9G4		nonsynonymous SNV	OR9G4:NM_001005284:exon1:c.G931T:p.	COSMIC	ID=COSM928472	222	0	0	1	234	5	0.0714419	1	150	19	0.433267	1
25	chr15	88690581	88690581	A	G	exonic	NTRK3		nonsynonymous SNV	NTRK3:NM_001320134:exon4:c.T449C:p.L	NOVEL		542	2	0.0157357	1	508	4	0.0343644	1	291	30	0.432836	1
25	chr18	52946863	52946863	C	G	exonic	TCF4		nonsynonymous SNV	TCF4:NM_001243234:exon2:c.G94C:p.A32	NOVEL		272	0	0	1	305	0	0	1	224	28	0.428199	1
25	chr5	132015497	132015497	C	G	exonic	IL4		nonsynonymous SNV	IL4:NM_172348:exon2:c.C227G:p.A76G,IL4	NOVEL		298	0	0	1	299	0	0	1	201	23	0.395702	1
25	chr20	44596552	44596552	C	T	exonic	ZNF335		nonsynonymous SNV	ZNF335:NM_022095:exon5:c.G635A:p.G2	NOVEL		155	0	0	1	137	1	0.0174994	1	127	20	0.388271	1
25	chr19	44418985	44418985	G	A	exonic	ZNF45		synonymous SNV	ZNF45:NM_003425:exon10:c.C603T:p.F20	COSMIC	ID=COSM554223	885	75	0.334383	1	1006	16	0.0651195	1	525	55	0.384845	1
25	chr19	44419279	44419279	G	A	exonic	ZNF45		synonymous SNV	ZNF45:NM_003425:exon10:c.C309T:p.F10	NOVEL		440	50	0.436745	1	433	8	0.075456	1	191	19	0.367186	1
25	chr11	134229018	134229018	A	C	exonic	GLB1L2		nonsynonymous SNV	GLB1L2:NM_001370460:exon7:c.A716C:p.	NOVEL		300	0	0	1	308	0	0	1	303	31	0.357687	1
25	chr19	4217918	4217918	C	G	exonic	ANKRD24		nonsynonymous SNV	ANKRD24:NM_133475:exon18:c.C2761G:p	NOVEL		84	0	0	1	89	10	0.444307	1	25	2	0.343062	1
25	chr19	16688137	16688137	A	T	exonic	MED26		synonymous SNV	MED26:NM_004831:exon3:c.T504A:p.A168	NOVEL		310	0	0	1	278	0	0	1	229	18	0.337507	1
25	chr19	44419273	44419273	G	A	exonic	ZNF45		synonymous SNV	ZNF45:NM_003425:exon10:c.C315T:p.S10	NOVEL		463	47	0.394441	1	447	8	0.0731343	1	210	19	0.33672	1
25	chr15	73660504	73660504	C	A	exonic	HCN4		synonymous SNV	HCN4:NM_005477:exon1:c.G108T:p.G36G	NOVEL		227	0	0	1	205	0	0	1	269	21	0.335373	1
25	chr4	83839934	83839934	A	C	exonic	THAP9		nonsynonymous SNV	THAP9:NM_024672:exon5:c.A2569C:p.K85	NOVEL		99	0	0	1	111	0	0	1	53	7	0.332942	1
25	chr19	44419272	44419272	G	A	exonic	ZNF45		stopgain	ZNF45:NM_003425:exon10:c.C316T:p.Q10	NOVEL		466	49	0.407233	1	447	8	0.0731343	1	213	19	0.332366	1
25	chr14	20943349	20943349	G	A	exonic	PNP		nonsynonymous SNV	PNP:NM_000270:exon5:c.G590A:p.G197D	NOVEL		151	0	0	1	207	1	0.0116102	1	143	18	0.319057	1
25	chr17	3638156	3638156	C	G	exonic	ITGAE		nonsynonymous SNV	ITGAE:NM_002208:exon21:c.G2610C:p.M	NOVEL		221	0	0	1	215	2	0.0222574	1	167	21	0.318775	1
25	chr17	7727276	7727276	G	A	exonic	DNAH2		synonymous SNV	DNAH2:NM_020877:exon75:c.G11454A:p.	NOVEL		221	0	0	1	254	0	0	1	161	20	0.315336	1
25	chr4	122828644	122828644	G	C	exonic	TRPC3		nonsynonymous SNV	TRPC3:NM_003305:exon6:c.C1652G:p.S55	NOVEL		147	0	0	1	155	0	0	1	89	11	0.313917	1
25	chr11	118130814	118130814	T	C	exonic	MPZL2		nonsynonymous SNV	MPZL2:NM_005797:exon4:c.A539G:p.K18	NOVEL		525	0	0	1	653	0	0	1	399	35	0.31079	1
25	chr19	45561146	45561146	C	A	exonic	CLASRP		synonymous SNV	CLASRP:NM_001278439:exon6:c.C417A:p.	NOVEL		176	0	0	1	152	18	0.465738	1	154	11	0.308756	1
25	chr9	116857500	116857500	G	A	exonic	KIF12		synonymous SNV	KIF12:NM_138424:exon7:c.C594T:p.T198T	NOVEL		422	1	0.0101184	1	367	0	0	1	645	46	0.308309	1
25	chr17	35880709	35880709	G	A	exonic	SYNRG		nonsynonymous SNV	SYNRG:NM_001163547:exon18:c.C3091T:	NOVEL		170	0	0	1	179	0	0	1	241	29	0.306518	1
25	chr11	107207285	107207285	T	G	exonic	CWF19L2		nonsynonymous SNV	CWF19L2:NM_152434:exon15:c.A2357C:p	NOVEL		154	0	0	1	163	0	0	1	106	9	0.301601	1
25	chr1	185270145	185270145	C	T	exonic	IVNS1ABP		nonsynonymous SNV	IVNS1ABP:NM_006469:exon10:c.G1079A:	NOVEL		260	0	0	1	268	0	0	1	222	26	0.299188	1
25	chr17	71238490	71238490	G	T	exonic	C17orf80		nonsynonymous SNV	C17orf80:NM_001100622:exon4:c.G1621T	NOVEL		265	0	0	1	290	0	0	1	178	20	0.288262	1
25	chr3	52292649	52292649	A	G	exonic	WDR82		nonsynonymous SNV	WDR82:NM_025222:exon8:c.T815C:p.I272	NOVEL		325	0	0	1	366	0	0	1	339	27	0.284296	1
25	chr19	44417773	44417773	G	A	exonic	ZNF45		synonymous SNV	ZNF45:NM_003425:exon10:c.C1815T:p.V6	NOVEL		427	39	0.358206	1	430	3	0.0288188	1	410	30	0.276707	1
25	chr19	44417697	44417697	G	A	exonic	ZNF45		stopgain	ZNF45:NM_003425:exon10:c.C1891T:p.Q6	DBSNP		440	47	0.413069	1	419	3	0.02957	1	254	18	0.268568	1
25	chr3	122990436	122990436	G	T	exonic	SEC22A		nonsynonymous SNV	SEC22A:NM_012430:exon7:c.G791T:p.C26	NOVEL		620	0	0	1	729	2	0.00934315	1	593	44	0.266196	1
25	chr1	19181428	19181428	G	A	exonic	TAS1R2		nonsynonymous SNV	TAS1R2:NM_152232:exon3:c.C536T:p.A17	NOVEL		258	0	0	1	262	0	0	1	238	24	0.261416	1
25	chr19	35842663	35842663	G	C	exonic	FFAR1		nonsynonymous SNV	FFAR1:NM_005303:exon1:c.G209C:p.G70	NOVEL		459	0	0	1	395	0	0	1	495	38	0.251469	1
25	chr6	7884595	7884595	A	T	exonic	TXNDC5		stopgain	TXNDC5:NM_001145549:exon9:c.T849A:p.	NOVEL		91	0	0	1	107	17	0.331078	1	64	6	0.244611	1
25	chr7	11676061	11676061	C	A	exonic	THSD7A		nonsynonymous SNV	THSD7A:NM_015204:exon2:c.G718T:p.V24	NOVEL		518	0	0	1	564	0	0	1	430	29	0.243486	1
25	chr5	140790149	140790149	G	C	exonic	PCDHGB6		nonsynonymous SNV	PCDHGB6:NM_018926:exon1:c.G2380C:p.	NOVEL		595	0	0	1	603	0	0	1	386	25	0.234416	1
25	chr19	44418369	44418369	G	A	exonic	ZNF45		stopgain	ZNF45:NM_003425:exon10:c.C1219T:p.Q4	COSMIC	ID=COSM360850	349	46	0.498442	1	375	5	0.0547304	1	238	14	0.225465	1
25	chr3	157082113	157082113	C	T	exonic	VEPH1		nonsynonymous SNV	VEPH1:NM_001167911:exon8:c.G1316A:p	NOVEL		183	0	0	1	174	0	0	1	82	5	0.221482	1
25	chr2	179584886	179584886	C	G	exonic	TTN		nonsynonymous SNV	TTN:NM_133378:exon78:c.G19751C:p.W6	NOVEL		487	0	0	1	574	0	0	1	507	40	0.208687	1
25	chr7	128481343	128481343	G	T	exonic	FLNC		nonsynonymous SNV	FLNC:NM_001127487:exon12:c.G1933T:p.	NOVEL		305	1	0.0107402	1	277	1	0.0122839	1	382	21	0.200818	1
25	chr10	116417780	116417780	C	G	exonic	ABLIM1		nonsynonymous SNV	ABLIM1:NM_002313:exon1:c.G180C:p.L60	NOVEL		361	0	0	1	367	1	0.00656229	1	355	26	0.194747	1
25	chr11	67265040	67265040	G	C	exonic	PITPNM1		synonymous SNV	PITPNM1:NM_001130848:exon13:c.C1893	NOVEL		339	0	0	1	313	0	0	1	367	19	0.189694	1
25	chr4	155241880	155241880	C	A	exonic	DCHS2		synonymous SNV	DCHS2:NM_001358235:exon9:c.G4671T:p.	DBSNP		404	0	0	1	483	0	0	1	205	14	0.182434	1
25	chr19	52000679	52000679	C	G	exonic	SIGLEC12		nonsynonymous SNV	SIGLEC12:NM_033329:exon5:c.G1072C:p.	NOVEL		234	0	0	1	243	0	0	1	209	10	0.175972	1
25	chr13	41239821	41239821	C	T	exonic	FOXO1		nonsynonymous SNV	FOXO1:NM_002015:exon1:c.G529A:p.A17	NOVEL		213	1	0.0106845	1	240	0	0	1	179	11	0.16522	1
25	chr5	140794128	140794128	T	C	exonic	PCDHGA10		synonymous SNV	PCDHGA10:NM_018913:exon1:c.T1386C:p	NOVEL		569	0	0	1	513	1	0.00664382	1	484	21	0.160257	1
25	chr14	104618409	104618409	G	T	exonic	KIF26A		nonsynonymous SNV	KIF26A:NM_015656:exon3:c.G346T:p.A11	NOVEL		118	0	0	1	103	0	0	1	247	14	0.153077	1
25	chr2	210806091	210806091	C	A	exonic	UNC80		nonsynonymous SNV	UNC80:NM_032504:exon43:c.C6595A:p.L	NOVEL		186	0	0	1	247	0	0	1	163	9	0.149326	1
25	chr19	19656567	19656567	G	C	exonic	CILP2		nonsynonymous SNV	CILP2:NM_153221:exon8:c.G3213C:p.L107	NOVEL		466	0	0	1	443	0	0	1	372	12	0.144729	1
25	chr3	57542980	57542980	G	T	exonic	PDE12		nonsynonymous SNV	PDE12:NM_001322176:exon1:c.G874T:p.D	NOVEL		522	0	0	1	556	0	0	1	506	19	0.139471	1
25	chr11	56128323	56128323	A	G	exonic	OR8J1		nonsynonymous SNV	OR8J1:NM_001005205:exon1:c.A601G:p.I	NOVEL		433	0	0	1	480	0	0	1	251	9	0.1334	1
25	chr6	35199540	35199540	C	T	exonic	SCUBE3		stopgain	SCUBE3:NM_001303136:exon4:c.C373T:p.	NOVEL		383	0	0	1	390	0	0	1	492	24	0.132735	1
25	chr1	233498032	233498032	A	G	exonic	MAP3K21		synonymous SNV	MAP3K21:NM_032435:exon5:c.A1545G:p.	NOVEL		200	0	0	1	191	0	0	1	164	7	0.116822	1
25	chr16	82033594	82033594	C	G	exonic	SDR42E1		nonsynonymous SNV	SDR42E1:NM_145168:exon3:c.G304C:p.G	NOVEL		146	0	0	1	197	0	0	1	189	8	0.11589	1
25	chr14	100793589	100793589	C	A	exonic	SLC25A47		nonsynonymous SNV	SLC25A47:NM_207117:exon4:c.C209A:p.S	NOVEL		233	0	0	1	248	0	0	1	364	15	0.112947	1
25	chr1	43818196	43818196	C	T	exonic	MPL		nonsynonymous SNV	MPL:NM_005373:exon12:c.C1661T:p.A554	DBSNP		170	0	0	1	170	0	0	1	234	9	0.105696	1
25	chr3	46009502	46009502	G	A	exonic	FYCO1		nonsynonymous SNV	FYCO1:NM_024513:exon8:c.C1324T:p.R44	DBSNP+COSMIC	ID=COSM120769	628	4	0.0208006	1	579	45	0.246268	1	540	15	0.104157	1
25	chr12	132325348	132325348	C	T	exonic	MMP17		nonsynonymous SNV	MMP17:NM_016155:exon4:c.C653T:p.T21	NOVEL		319	0	0	1	299	0	0	1	509	19	0.102693	1
25	chr20	44004019	44004019	G	C	exonic	TP53TG5		nonsynonymous SNV	TP53TG5:NM_014477:exon4:c.C428G:p.T1	NOVEL		307	0	0	1	323	0	0	1	298	11	0.101591	1
25	chr3	145913043	145913043	G	T	exonic	PLSCR4		nonsynonymous SNV	PLSCR4:NM_001177304:exon5:c.C498A:p.	NOVEL		502	0	0	1	549	25	0.148734	1	526	14	0.0999131	1
25	chr1	6536001	6536001	C	G	exonic	PLEKHG5		nonsynonymous SNV	PLEKHG5:NM_001042664:exon3:c.G139C:p	NOVEL		150	0	0	1	150	0	0	1	276	10	0.0997829	1
25	chr17	56382790	56382790	G	A	exonic	TSPOAP1		synonymous SNV	TSPOAP1:NM_024418:exon28:c.C5211T:p.	NOVEL		408	0	0	1	408	0	0	1	461	16	0.0957247	1
25	chr4	85750235	85750235	G	T	exonic	WDFY3		nonsynonymous SNV	WDFY3:NM_014991:exon9:c.C878A:p.S29	NOVEL		456	0	0	1	453	5	0.0263638	1	405	13	0.0887543	1
25	chr10	119043501	119043501	C	G	exonic	PDZD8		nonsynonymous SNV	PDZD8:NM_173791:exon5:c.G2743C:p.G9	NOVEL		518	0	0	1	564	0	0	1	416	13	0.0864785	1
25	chr19	3808964	3808964	C	T	exonic	ZFR2		synonymous SNV	ZFR2:NM_015174:exon17:c.G2451A:p.V81	NOVEL		139	2	0.0607107	1	122	19	0.592725	1	136	2	0.0671209	1
25	chr2	218762631	218762631	C	A	exonic	TNS1		stopgain	TNS1:NM_001308022:exon5:c.G58T:p.E20	NOVEL		284	1	0.00802276	1	279	21	0.169045	1	367	8	0.0608809	1
25	chr18	74980796	74980796	G	T	exonic	GALR1		nonsynonymous SNV	GALR1:NM_001480:exon3:c.G988T:p.D33	NOVEL		532	0	0	1	501	26	0.168478	1	447	6	0.0510436	1
25	chr8	103287986	103287986	C	T	exonic	UBR5		nonsynonymous SNV	UBR5:NM_001282873:exon46:c.G6580A:p	COSMIC	ID=COSM173944	350	0	0	1	329	31	0.207952	1	416	7	0.0472259	1
25	chr3	69351535	69351535	C	T	exonic	FRMD4B		synonymous SNV	FRMD4B:NM_015123:exon4:c.G375A:p.L1	NOVEL		467	0	0	1	465	21	0.147558	1	402	4	0.0379684	1
25	chr22	30494914	30494914	C	T	exonic	HORMAD2		nonsynonymous SNV	HORMAD2:NM_152510:exon3:c.C125T:p.S	COSMIC	ID=COSM384245	241	0	0	1	214	13	0.1383	1	236	2	0.0239814	1
25	chr15	34533489	34533489	C	A	exonic	SLC12A6		nonsynonymous SNV	SLC12A6:NM_001042497:exon16:c.G2167	NOVEL		565	0	0	1	474	15	0.134928	1	393	2	0.0234498	1
25	chr6	151151827	151151827	G	A	exonic	PLEKHG1		nonsynonymous SNV	PLEKHG1:NM_001329803:exon13:c.G1463	NOVEL		410	1	0.00556323	1	433	20	0.106619	1	417	3	0.0203842	1

25	chr19	46893309	46893309	G	C	exonic	PPP5C		nonsynonymous SNV	PPP5C:NM_001204284:exon11:c.G1291C:p	NOVEL		264	0	0	1	232	10	0.181762	1	501	2	0.0184149	1
25	chr7	100457655	100457655	G	C	exonic	SLC12A9		nonsynonymous SNV	SLC12A9:NM_001363494:exon5:c.G697C:p	NOVEL		407	0	0	1	408	18	0.144293	1	421	2	0.0182212	1
25	chr3	100976421	100976421	C	T	exonic	IMPG2		nonsynonymous SNV	IMPG2:NM_016247:exon10:c.G1105A:p.G	COSMIC	ID=COSM452131	390	1	0.00840534	1	436	16	0.120882	1	218	1	0.0175972	1
25	chr18	21199557	21199557	C	T	exonic	ANKRD29		nonsynonymous SNV	ANKRD29:NM_001308238:exon7:c.G565A	NOVEL		158	0	0	1	165	10	0.195138	1	229	1	0.0167556	1
25	chr7	124404936	124404936	G	A	exonic	GPR37		nonsynonymous SNV	GPR37:NM_005302:exon1:c.C95T:p.P32L	NOVEL		419	0	0	1	478	24	0.163263	1	484	2	0.0158592	1
25	chr19	55853724	55853724	G	T	exonic	KMT5C		synonymous SNV	KMT5C:NM_032701:exon3:c.G252T:p.R84	NOVEL		71	0	0	1	55	5	0.283068	1	48	0	0	1
25	chr22	28290624	28290624	T	A	exonic	PITPNB		synonymous SNV	PITPNB:NM_001284277:exon7:c.A375T:p.	NOVEL		105	0	0	1	82	7	0.189938	1	60	0	0	1
25	chr19	47231881	47231881	C	G	exonic	STRN4		nonsynonymous SNV	STRN4:NM_001039877:exon7:c.G1033C:p	NOVEL		258	0	0	1	223	10	0.188783	1	236	0	0	1
25	chr4	169204733	169204733	A	T	exonic	DDX60		nonsynonymous SNV	DDX60:NM_017631:exon13:c.T1586A:p.L5	NOVEL		151	0	0	1	172	14	0.181768	1	98	0	0	1
25	chr5	140773629	140773629	A	T	exonic	PCDHGA8		nonsynonymous SNV	PCDHGA8:NM_014004:exon1:c.A1249T:p.	NOVEL		579	0	0	1	565	22	0.127987	1	439	0	0	1
25	chr1	53250676	53250676	T	A	exonic	ZYG11B		nonsynonymous SNV	ZYG11B:NM_024646:exon5:c.T1220A:p.V4	NOVEL		223	0	0	1	241	11	0.105413	1	111	0	0	1
25	chr11	60501112	60501112	G	T	exonic	MS4A18		stopgain	MS4A18:NM_001354471:exon3:c.G577T:p	NOVEL		271	33	0.356757	1	358	1	0.00951232	1	302	0	0	1
25	chr9	107379866	107379866	G	T	exonic	OR13C9		nonsynonymous SNV	OR13C9:NM_001001956:exon1:c.C620A:p.	NOVEL		875	75	0.337903	1	935	0	0	1	488	0	0	1
25	chr3	111693376	111693376	C	T	exonic	PHLDB2		nonsynonymous SNV	PHLDB2:NM_145753:exon17:c.C3599T:p.T	NOVEL		210	23	0.324417	1	271	0	0	1	192	0	0	1
25	chr8	143999103	143999103	G	A	exonic	CYP11B2		synonymous SNV	CYP11B2:NM_000498:exon1:c.C154T:p.L52	NOVEL		208	32	0.304865	1	219	0	0	1	454	0	0	1
25	chr8	42048910	42048910	C	A	exonic	PLAT		nonsynonymous SNV	PLAT:NM_000930:exon3:c.G95T:p.R32I,PL	NOVEL		81	21	0.264865	1	80	0	0	1	105	0	0	1
25	chr8	42038083	42038083	C	G	exonic	PLAT		nonsynonymous SNV	PLAT:NM_001319189:exon8:c.G743C:p.G2	NOVEL		91	23	0.259554	1	97	0	0	1	99	0	0	1
25	chr8	42038151	42038151	C	A	exonic	PLAT		synonymous SNV	PLAT:NM_001319189:exon8:c.G675T:p.G2	NOVEL		81	20	0.25475	1	80	0	0	1	92	0	0	1
25	chr8	42048901	42048901	C	A	exonic	PLAT		nonsynonymous SNV	PLAT:NM_000930:exon3:c.G104T:p.R35I,P	NOVEL		75	18	0.248997	1	70	0	0	1	93	0	0	1
25	chr5	16680081	16680081	G	T	exonic	MYO10		nonsynonymous SNV	MYO10:NM_012334:exon33:c.C4517A:p.T	NOVEL		237	19	0.243919	1	233	0	0	1	214	0	0	1
25	chr8	42037833	42037833	C	T	exonic	PLAT		synonymous SNV	PLAT:NM_001319189:exon9:c.G882A:p.E2	DBSNP		140	14	0.116953	1	133	0	0	1	137	0	0	1
25	chr8	42037781	42037781	C	A	exonic	PLAT		nonsynonymous SNV	PLAT:NM_001319189:exon9:c.G934T:p.D3	NOVEL		115	11	0.112312	1	106	0	0	1	134	0	0	1
25	chr8	42037787	42037787	C	T	exonic	PLAT		nonsynonymous SNV	PLAT:NM_001319189:exon9:c.G928A:p.D3	NOVEL		119	11	0.108857	1	106	0	0	1	137	0	0	1
25	chr1	54344314	54344314	C	T	splicing	YIPF1	NM_018982:exon5:c.2			DBSNP		181	0	0	1	162	0	0	1	162	8	0.134296	1
26	chrX	99661719	99661719	G	A	exonic	PCDH19		nonsynonymous SNV	PCDH19:NM_001105243:exon1:c.C1877T:p	DBSNP		5	165	NA	1	4	215	NA	1	0	147	NA	1
26	chrX	103268012	103268012	G	C	exonic	H2BFWT		nonsynonymous SNV	H2BFWT:NM_001002916:exon1:c.C221G:p	NOVEL		43	39	NA	1	0	90	NA	1	0	60	NA	1
26	chrX	140984785	140984785	G	T	exonic	MAGEC3		nonsynonymous SNV	MAGEC3:NM_177456:exon5:c.G347T:p.S1	NOVEL		42	32	NA	1	1	70	NA	1	1	29	NA	1
26	chrX	79286504	79286504	T	C	exonic	TBX22		nonsynonymous SNV	TBX22:NM_001303475:exon7:c.T1097C:p.	NOVEL		195	0	NA	1	119	85	NA	1	135	0	NA	1
26	chrX	139586439	139586439	G	A	exonic	SOX3		nonsynonymous SNV	SOX3:NM_005634:exon1:c.C787T:p.H263Y	NOVEL		61	0	NA	1	0	83	NA	1	14	4	NA	1
26	chr5	26881451	26881451	C	G	exonic	CDH9		nonsynonymous SNV	CDH9:NM_016279:exon12:c.G2164C:p.E72	NOVEL		172	163	1.20024	1	221	203	0.989729	1	101	89	NA	1
26	chr12	96408758	96408758	G	C	exonic	LTA4H		nonsynonymous SNV	LTA4H:NM_000895:exon12:c.C1079G:p.T3	NOVEL		142	135	1.2022	1	158	158	1.03361	1	125	176	1.44968	1
26	chr7	157926396	157926396	G	A	exonic	PTPRN2		nonsynonymous SNV	PTPRN2:NM_001308267:exon8:c.C1415T:p	DBSNP+COSMIC	ID=COSM700368	78	41	1.19442	1	78	45	1.08353	1	76	36	1.43977	1
26	chr1	2418808	2418808	C	T	exonic	PLCH2		synonymous SNV	PLCH2:NM_001303012:exon7:c.C1026T:p.	DBSNP+COSMIC	ID=COSM906464	45	58	1.38904	1	59	39	0.822668	1	41	55	1.42042	1
26	chr1	207076332	207076332	A	C	exonic	IL24		nonsynonymous SNV	IL24:NM_001185157:exon6:c.A393C:p.E13	NOVEL		249	69	0.63176	1	129	183	0.868561	2	86	380	1.41859	2
26	chr11	34120856	34120856	G	A	exonic	CAPRIN1		nonsynonymous SNV	CAPRIN1:NM_005898:exon19:c.G2072A:p.	NOVEL		149	45	0.572182	1	94	104	1.08581	1	37	49	1.41262	1
26	chr1	86173916	86173916	C	T	exonic	ZNHIT6		nonsynonymous SNV	ZNHIT6:NM_001170670:exon1:c.G52A:p.V	NOVEL		194	37	0.395106	1	133	127	1.00976	1	154	199	1.39767	1
26	chr5	77684723	77684723	C	T	exonic	SCAMP1		unknown	UNKNOWN	DBSNP		60	53	1.15697	1	59	58	1.02477	1	21	27	1.3946	1
26	chr20	56138674	56138674	T	C	exonic	PCK1		synonymous SNV	PCK1:NM_002591:exon6:c.T852C:p.F284F	NOVEL		105	7	0.154171	1	119	1	0.0172268	1	81	53	1.37614	1
26	chr12	27916261	27916261	T	C	exonic	MANSC4		nonsynonymous SNV	MANSC4:NM_001146221:exon3:c.A433G:	NOVEL		93	78	1.12518	1	84	79	1.0019	1	29	36	1.37314	1
26	chr11	102980490	102980490	T	A	exonic	DYNC2H1		nonsynonymous SNV	DYNC2H1:NM_001080463:exon1:c.T187A:	NOVEL		43	33	1.07108	1	59	35	0.769709	1	13	16	1.36788	1
26	chr2	74275408	74275408	G	A	exonic	TET3		synonymous SNV	TET3:NM_001366022:exon3:c.G2085A:p.E	NOVEL		120	157	1.39812	1	152	161	1.06333	1	204	246	1.35534	1
26	chr5	19544018	19544018	T	G	exonic	CDH18		nonsynonymous SNV	CDH18:NM_001167667:exon9:c.A1350C:p.	NOVEL		78	76	1.21735	1	74	78	1.06081	1	102	65	1.35421	1
26	chr16	88499828	88499828	G	C	exonic	ZNF469		nonsynonymous SNV	ZNF469:NM_001367624:exon1:c.G5950C:	NOVEL		178	36	0.414966	1	104	99	1.00815	1	78	93	1.34838	1
26	chr9	104133419	104133419	T	A	exonic	BAAT		stopgain	BAAT:NM_001127610:exon2:c.A268T:p.R9	NOVEL		253	110	1.05053	1	286	134	0.944908	1	118	74	1.34097	1
26	chr2	186664624	186664624	C	A	exonic	FSIP2		nonsynonymous SNV	FSIP2:NM_173651:exon17:c.C10591A:p.Q	NOVEL		150	43	0.549585	1	115	89	0.901875	1	47	55	1.33687	1
26	chr1	21605796	21605796	C	T	exonic	ECE1		synonymous SNV	ECE1:NM_001113347:exon1:c.G132A:p.Q4	NOVEL		110	113	1.24996	1	99	110	1.08801	1	175	202	1.32842	1
26	chr4	7435602	7435602	C	A	exonic	PSAPL1		nonsynonymous SNV	PSAPL1:NM_001085382:exon1:c.G1005T:	NOVEL		147	152	1.254	1	190	158	0.938565	1	154	177	1.32578	1
26	chr1	203138165	203138165	C	T	exonic	MYBPH		nonsynonymous SNV	MYBPH:NM_004997:exon9:c.G1286A:p.R4	DBSNP		100	138	0.965757	2	167	73	0.900836	1	136	83	1.31863	1
26	chr7	6098634	6098634	G	A	exonic	EIF2AK1		synonymous SNV	EIF2AK1:NM_001134335:exon1:c.C81T:p.	NOVEL		139	74	1.20441	1	144	85	1.0993	1	138	84	1.31649	1
26	chr6	151917701	151917701	A	T	exonic	CCDC170		nonsynonymous SNV	CCDC170:NM_025059:exon9:c.A1699T:p.N	NOVEL		116	88	1.06408	1	109	121	1.08754	1	65	73	1.31151	1
26	chr22	19511789	19511789	G	C	exonic	CLDN5		nonsynonymous SNV	CLDN5:NM_001130861:exon1:c.C245G:p.S	NOVEL		129	34	0.514535	1	78	69	0.970327	1	134	150	1.30948	1
26	chr17	73497984	73497984	G	A	exonic	CASKIN2		synonymous SNV	CASKIN2:NM_001142643:exon17:c.C2925T	DBSNP		66	64	1.2144	1	66	64	1.01771	1	61	68	1.30691	1
26	chr19	47774787	47774787	C	T	exonic	CCDC9		nonsynonymous SNV	CCDC9:NM_015603:exon12:c.C1448T:p.P4	NOVEL		43	19	1.06239	1	26	16	0.787512	1	32	34	1.27721	1
26	chr1	36859448	36859448	C	T	exonic	LSM10		nonsynonymous SNV	LSM10:NM_032881:exon2:c.G283A:p.E95	NOVEL		152	41	0.524023	1	132	121	0.98867	1	92	96	1.26602	1
26	chr5	177019341	177019341	C	T	exonic	TMED9		synonymous SNV	TMED9:NM_017510:exon1:c.C126T:p.I42I	NOVEL		103	76	1.04733	1	91	90	1.0279	1	72	75	1.26494	1
26	chr1	232942885	232942885	G	A	exonic	MAP10		nonsynonymous SNV	MAP10:NM_019090:exon1:c.G2116A:p.D7	NOVEL		277	155	1.23219	1	314	168	1.03228	1	184	105	1.2641	1
26	chr10	88702741	88702741	C	T	exonic	MMRN2		synonymous SNV	MMRN2:NM_024756:exon6:c.G1800A:p.L	NOVEL		101	25	0.489433	1	57	76	1.18127	1	109	112	1.25647	1
26	chr1	109952579	109952579	C	T	exonic	PSMA5		nonsynonymous SNV	PSMA5:NM_001199772:exon7:c.G445A:p.	NOVEL		174	44	0.672345	1	96	69	0.864473	1	39	40	1.25533	1
26	chr3	74418472	74418472	T	C	exonic	CNTN3		nonsynonymous SNV	CNTN3:NM_020872:exon7:c.A814G:p.K27	NOVEL		292	138	1.11258	1	314	189	1.11283	1	153	86	1.25196	1
26	chr14	44976128	44976128	T	G	exonic	FSCB		nonsynonymous SNV	FSCB:NM_032135:exon1:c.A63C:p.K21N	NOVEL		127	108	1.13365	1	151	147	1.01974	1	66	168	1.24897	2
26	chr21	37833538	37833538	C	T	exonic	CLDN14		synonymous SNV	CLDN14:NM_001146079:exon2:c.G456A:p	NOVEL		75	69	1.18198	1	68	77	1.09776	1	81	45	1.2426	1
26	chr11	9164297	9164297	G	C	exonic	DENND5A		nonsynonymous SNV	DENND5A:NM_001348749:exon20:c.C341	NOVEL		87	93	1.27448	1	99	103	1.05408	1	77	77	1.23964	1
26	chr2	74007200	74007200	G	C	exonic	DUSP11		nonsynonymous SNV	DUSP11:NM_003584:exon1:c.C43G:p.R15	DBSNP		91	94	1.25337	1	119	90	0.89019	1	121	121	1.23964	1
26	chr20	1161950	1161950	G	A	exonic	TMEM74B		nonsynonymous SNV	TMEM74B:NM_001304749:exon2:c.C241T	NOVEL		273	83	0.575111	1	177	168	1.00665	1	144	143	1.23532	1
26	chr16	67709428	67709428	C	T	exonic	GFOD2		nonsynonymous SNV	GFOD2:NM_030819:exon3:c.G788A:p.R26	DBSNP		12	328	1.18984	2	6	328	1.01504	2	1	268	1.23503	2
26	chr1	11898624	11898624	C	T	exonic	CLCN6		synonymous SNV	CLCN6:NM_001256959:exon21:c.C2370T:p	DBSNP		275	241	1.1521	1	298	301	1.03879	1	318	315	1.23377	1
26	chr16	65005840	65005840	G	T	exonic	CDH11		synonymous SNV	CDH11:NM_001330576:exon9:c.C1140A:p.	NOVEL		6	226	1.20147	2	6	236	1.00798	2	1	141	1.23091	2
26	chr16	65005841	65005841	G	T	exonic	CDH11		nonsynonymous SNV	CDH11:NM_001330576:exon9:c.C1139A:p.	NOVEL		6	224	1.2012	2	6	234	1.00777	2	1	140	1.23085	2
26	chr7	139636026	139636026	G	A	exonic	TBXAS1		nonsynonymous SNV	TBXAS1:NM_001366537:exon4:c.G187A:p	DBSNP+COSMIC	ID=COSM473258	160	73	1.08615	1	166	75	0.921676	1	125	47	1.22399	1
26	chr1	3800181	3800181	A	G	exonic	DFFB		nonsynonymous SNV	DFFB:NM_001282669:exon7:c.A965G:p.N	NOVEL		88	105	1.34201	1	99	117	1.11974	1	104	101	1.2215	1
26	chr11	66136845	66136845	G	A	exonic	SLC29A2		synonymous SNV	SLC29A2:NM_001532:exon3:c.C270T:p.Y9	NOVEL		91	68	1.05496	1	76	93	1.13758	1	109	105	1.21647	1
26	chr19	57328156	57328156	C	T	exonic	PEG3		nonsynonymous SNV	PEG3:NM_001369735:exon8:c.G1189A:p.E	NOVEL		498	84	0.500354	1	267	234	0.965527	1	150	143	1.21003	1
26	chr1	112525029	112525029	C	T	exonic	KCND3		nonsynonymous SNV	KCND3:NM_004980:exon2:c.G320A:p.R10	COSMIC	ID=COSM669073	414	222	1.16276	1	241	220	0.986525	1	204	193	1.2053	1
26	chr22	18304835	18304835	C	T	exonic	MICAL3		nonsynonymous SNV	MICAL3:NM_015241:exon24:c.G3409A:p.E	COSMIC	ID=COSM569280	137	51	0.669169	1	122	87	0.860517	1	157	146	1.19464	1
26	chr10	73545398	73545398	T	C	exonic	CDH23		nonsynonymous SNV	CDH23:NM_022124:exon42:c.T5723C:p.V1	NOVEL		162	141	1.14789	1	143	114	0.916976	1	174	161	1.19154	1
26	chr7	107748225	107748225	C	T	exonic	LAMB4		nonsynonymous SNV	LAMB4:NM_001318046:exon6:c.G442A:p.	NOVEL		402	82	0.58734	1	374	161	0.891263	1	345	122	1.17018	1
26	chr19	36940144	36940144	C	T	exonic	ZNF566		nonsynonymous SNV	ZNF566:NM_001300970:exon4:c.G680A:p	NOVEL		235	35	0.539191	1	190	69	0.789011	1	86	46	1.16904	1
26	chr19	57088311	57088311	G	T	exonic	ZNF470		nonsynonymous SNV	ZNF470:NM_001001668:exon6:c.G514T:p	NOVEL		185	27	0.441519	1	188	0	0	1	36	32	1.16672	1
26	chr3	48802441	48802441	G	A	exonic	PRKAR2A		nonsynonymous SNV	PRKAR2A:NM_001321989:exon7:c.C785T:	NOVEL		112	67	1.29761	1	131	49	0.806227	1	125	63	1.16593	1
26	chr17	73214354	73214354	G	A	exonic	NUP85		nonsynonymous SNV	NUP85:NM_001303276:exon6:c.G412A:p.	NOVEL		68	72	1.26861	1	69	78	1.09689	1	61	54	1.16419	1
26	chr11	119043692	119043692	G	C	exonic	NLRX1		nonsynonymous SNV	NLRX1:NM_001282143:exon4:c.G223C:p.A	NOVEL		36	47	1.39683	1	47	39	0.937459	1	52	46	1.16375	1
26	chr18	77137338	77137338	G	A	exonic	ATP9B		synonymous SNV	ATP9B:NM_001306085:exon29:c.G3366A:	DBSNP		85	84	1.22607	1	98	95	1.01754	1	111	98	1.16254	1
26	chr3	49162823	49162823	A	T	exonic	LAMB2		stopgain	LAMB2:NM_002292:exon19:c.T2583A:p.C	COSMIC	ID=COSM284963	386	92	0.667239	1	368	163	0.909132	1	373	187	1.16183	1
26	chr20	34810293	34810293	G	T	exonic	EPB41L1		stopgain	EPB41L1:NM_001258331:exon19:c.G2308	NOVEL		51	38	1.05322	1	45	39	0.95978	1	96	48	1.15976	1
26	chr5	74998617	74998617	G	A	exonic	POC5		nonsynonymous SNV	POC5:NM_152408:exon4:c.C251T:p.S84L,P	DBSNP+COSMIC	ID=COSM592246	96	87	1.17271	1	104	92	0.970327	1	62	54	1.15415	1
26	chr17	11840756	11840756	G	T	exonic	DNAH9		stopgain	DNAH9:NM_004662:exon12:c.G1513T:p.E	NOVEL		94	87	1.18567	1	91	92	1.03926	1	111	96	1.14981	1
26	chr9	113496570	113496570	T	C	exonic	MUSK		nonsynonymous SNV	MUSK:NM_001166281:exon6:c.T668C:p.V	COSMIC	ID=COSM462880	144	69	1.12303	1	163	104	1.1536	1	144	71	1.14897	1
26	chr4	3317994	3317994	G	A	exonic	RGS12		nonsynonymous SNV	RGS12:NM_002926:exon2:c.G97A:p.G33R	DBSNP+COSMIC	ID=COSM676583	114	111	1.21693	1	115	141	1.13859	1	88	76	1.14894	1
26	chr2	27306422	27306422	G	A	exonic	EMILIN1		synonymous SNV	EMILIN1:NM_007046:exon4:c.G1983A:p.L	NOVEL		168	55	0.608389	1	98	82	0.941733	1	107	91	1.13947	1
26	chr17	15968268	15968268	G	A	exonic	NCOR1		nonsynonymous SNV	NCOR1:NM_001190440:exon33:c.C5065T:	NOVEL		151	144	1.2041	1	136	168	1.14241	1	98	83	1.13691	1
26	chr1	112524471	112524471	C	T	exonic	KCND3		nonsynonymous SNV	KCND3:NM_004980:exon2:c.G878A:p.R29	COSMIC	ID=COSM893890	162	215	0.949867	2	176	125	0.858479	1	118	99	1.1311	1
26	chr19	38976592	38976592	G	A	exonic	RYR1		nonsynonymous SNV	RYR1:NM_000540:exon34:c.G5297A:p.G1	NOVEL		391	42	0.40346	1	237	109	0.966262	1	384	195	1.1298	1
26	chr11	122666890	122666890	C	T	exonic	UBASH3B		synonymous SNV	UBASH3B:NM_001363365:exon8:c.C1035	DBSNP		115	118	1.24925	1	127	133	1.05746	1	127	106	1.12792	1
26	chr7	150644080	150644080	G	A	exonic	KCNH2		nonsynonymous SNV	KCNH2:NM_172057:exon10:c.C2195T:p.T7	DBSNP		80	34	1.03394	1	72	41	1.07458	1	107	36	1.12765	1
26	chr4	164246788	164246788	G	C	exonic	NPY1R		synonymous SNV	NPY1R:NM_000909:exon3:c.C822G:p.V274	NOVEL		130	116	1.16318	1	137	128	0.998505	1	70	58	1.12343	1
26	chr15	101432825	101432825	G	A	exonic	ALDH1A3		synonymous SNV	ALDH1A3:NM_000693:exon4:c.G456A:p.Q	NOVEL		114	0	0	1	96	0	0	1	29	24	1.1227	1
26	chr7	121755210	121755210	T	G	exonic	AASS		nonsynonymous SNV	AASS:NM_005763:exon9:c.A961C:p.T321P	NOVEL		87	135	1.05408	2	85	164	0.975323	2	39	118	1.1222	3
26	chr19	52869643	52869643	C	T	exonic	ZNF610		nonsynonymous SNV	ZNF610:NM_001161427:exon5:c.C883T:p.	NOVEL		156	65	1.01963	1	204	0	0	1	57	47	1.12045	1
26	chr7	142569711	142569711	G	A	exonic	TRPV6		nonsynonymous SNV	TRPV6:NM_018646:exon15:c.C2047T:p.R6	DBSNP+COSMIC	ID=COSM473675	72	33	1.08955	1	88	35	0.842746	1	81	27	1.11982	1
26	chr8	77767670	77767670	C	T	exonic	ZFHX4		nonsynonymous SNV	ZFHX4:NM_024721:exon10:c.C8513T:p.S2	NOVEL		247	132	0.977783	1	220	373	0.931447	2	137	246	1.11737	2
26	chr17	41832790	41832790	G	A	exonic	SOST		nonsynonymous SNV	SOST:NM_025237:exon2:c.C562T:p.R188W	NOVEL		177	285	1.06929	2	162	309	0.971497	2	120	215	1.11649	2
26	chr6	151161271	151161271	C	T	exonic	PLEKHG1		stopgain	PLEKHG1:NM_001329803:exon14:c.C3280	NOVEL		132	147	1.29968	1	138	143	1.052	1	121	99	1.11568	1
26	chr9	132757186	132757186	C	G	exonic	FNBP1		nonsynonymous SNV	FNBP1:NM_001363755:exon2:c.G76C:p.E2	NOVEL		142	65	1.08859	1	159	86	1.0396	1	89	42	1.1155	1
26	chr10	68138931	68138931	C	T	exonic	CTNNA3		nonsynonymous SNV	CTNNA3:NM_001127384:exon12:c.G1711	DBSNP		79	73	1.18468	1	117	89	0.893119	1	54	44	1.11315	1
26	chr1	231487195	231487195	C	T	exonic	SPRTN		nonsynonymous SNV	SPRTN:NM_001261462:exon3:c.C467T:p.A	NOVEL		224	111	1.13791	1	142	202	0.869555	2	129	223	1.1021	2
26	chr10	90492032	90492032	G	T	exonic	LIPK		nonsynonymous SNV	LIPK:NM_001080518:exon4:c.G520T:p.G1	NOVEL		221	59	0.519778	1	137	137	1.03361	1	90	72	1.1019	1
26	chr11	107207349	107207349	G	A	exonic	CWF19L2		nonsynonymous SNV	CWF19L2:NM_152434:exon15:c.C2293T:p	NOVEL		28	33	1.33447	1	59	40	0.83524	1	25	20	1.1019	1
26	chr11	108200976	108200976	A	G	exonic	ATM		nonsynonymous SNV	ATM:NM_000051:exon50:c.A7343G:p.D24	COSMIC	ID=COSM690741	107	80	1.05529	1	114	111	1.01983	1	48	38	1.0955	1
26	chr9	84605912	84605912	C	G	exonic	SPATA31D1		nonsynonymous SNV	SPATA31D1:NM_001001670:exon4:c.C527	DBSNP		444	208	1.10595	1	524	261	0.984702	1	579	266	1.09525	1
26	chr4	114274541	114274541	A	T	exonic	ANK2		nonsynonymous SNV	ANK2:NM_001148:exon38:c.A4767T:p.E15	NOVEL		256	0	0	1	331	0	0	1	62	49	1.09446	1
26	chr7	48450209	48450209	T	G	exonic	ABCA13		nonsynonymous SNV	ABCA13:NM_152701:exon40:c.T12163G:p	NOVEL		332	81	0.679918	1	307	128	0.871474	1	301	138	1.09372	1
26	chr2	179447069	179447069	T	G	exonic	TTN		synonymous SNV	TTN:NM_003319:exon142:c.A38919C:p.V1	NOVEL		236	170	1.03287	1	255	209	0.931139	1	138	108	1.08847	1
26	chr1	236917340	236917340	C	A	exonic	ACTN2		nonsynonymous SNV	ACTN2:NM_001103:exon16:c.C1933A:p.Q	DBSNP		141	83	1.2725	1	156	76	0.970196	1	134	61	1.08839	1
26	chr7	117175374	117175374	T	A	exonic	CFTR		nonsynonymous SNV	CFTR:NM_000492:exon6:c.T652A:p.L218M	NOVEL		152	88	1.27114	1	188	109	1.08694	1	153	49	1.08656	1
26	chr9	4604205	4604205	G	A	exonic	SPATA6L		nonsynonymous SNV	SPATA6L:NM_001353488:exon9:c.C881T:	DBSNP		74	23	0.584897	1	41	47	1.10408	1	27	21	1.08469	1
26	chr9	101980494	101980494	C	T	exonic	ALG2		nonsynonymous SNV	ALG2:NM_033087:exon2:c.G973A:p.E325	NOVEL		154	63	1.00647	1	144	80	1.05773	1	82	37	1.08179	1
26	chr1	158436233	158436233	T	A	exonic	OR10K1		nonsynonymous SNV	OR10K1:NM_001004473:exon1:c.T882A:p.	NOVEL		616	1	0.0056187	1	665	0	0	1	445	141	1.07778	1
26	chr2	116539939	116539939	C	T	exonic	DPP10		nonsynonymous SNV	DPP10:NM_001321913:exon15:c.C733T:p.	NOVEL		109	97	1.16152	1	116	113	1.02007	1	95	73	1.07731	1
26	chr1	217955536	217955536	C	T	exonic	SPATA17		synonymous SNV	SPATA17:NM_138796:exon8:c.C744T:p.T2	COSMIC	ID=COSM678810	238	123	1.13499	1	166	278	0.960233	2	101	162	1.07157	2
26	chr3	140277689	140277689	G	A	exonic	CLSTN2		synonymous SNV	CLSTN2:NM_022131:exon12:c.G2031A:p.V	NOVEL		118	22	0.544774	1	72	44	1.12338	1	63	28	1.07055	1
26	chr19	39882263	39882263	G	A	exonic	MED29		synonymous SNV	MED29:NM_001317770:exon1:c.G201A:p.	NOVEL		226	66	0.940156	1	155	91	1.13462	1	193	90	1.06685	1
26	chr7	154862809	154862809	G	A	exonic	HTR5A		nonsynonymous SNV	HTR5A:NM_024012:exon1:c.G200A:p.R67	NOVEL		223	118	1.19963	1	288	117	0.855588	1	320	100	1.0665	1
26	chr17	8449876	8449876	G	C	exonic	MYH10		nonsynonymous SNV	MYH10:NM_005964:exon11:c.C1234G:p.Q	NOVEL		140	117	1.12299	1	176	150	0.951174	1	121	91	1.06422	1
26	chr2	210896246	210896246	C	T	exonic	KANSL1L		nonsynonymous SNV	KANSL1L:NM_152519:exon9:c.G2054A:p.R	NOVEL		120	39	0.60505	1	81	94	1.11039	1	52	39	1.06255	1
26	chr12	130831129	130831129	G	T	exonic	PIWIL1		nonsynonymous SNV	PIWIL1:NM_001190971:exon5:c.G531T:p.	COSMIC	ID=COSM936977	61	51	1.12325	1	69	78	1.09689	1	24	18	1.06255	1
26	chr3	195516055	195516055	G	A	exonic	MUC4		nonsynonymous SNV	MUC4:NM_018406:exon2:c.C2396T:p.S799	NOVEL		225	40	0.523282	1	185	82	0.909571	1	91	40	1.06238	1
26	chr3	184297523	184297523	G	A	exonic	EPHB3		nonsynonymous SNV	EPHB3:NM_004443:exon11:c.G1973A:p.R	DBSNP		276	131	1.11583	1	304	146	0.960891	1	239	105	1.06199	1
26	chr1	185894287	185894287	G	T	exonic	HMCN1		nonsynonymous SNV	HMCN1:NM_031935:exon9:c.G1394T:p.R4	NOVEL		201	116	1.21897	1	105	211	0.988779	2	82	128	1.06035	2
26	chr1	77620224	77620224	A	G	exonic	PIGK		nonsynonymous SNV	PIGK:NM_005482:exon9:c.T896C:p.L299P	NOVEL		83	0	0	1	92	0	0	1	35	26	1.05674	1
26	chr2	219920576	219920576	C	T	exonic	IHH		nonsynonymous SNV	IHH:NM_002181:exon3:c.G589A:p.A197T	DBSNP		43	46	1.27494	1	38	42	1.08529	1	58	43	1.05554	1
26	chr2	109292452	109292452	G	T	exonic	LIMS1		nonsynonymous SNV	LIMS1:NM_001193482:exon6:c.G625T:p.V	NOVEL		84	16	0.394679	1	82	50	0.783037	1	46	34	1.0537	1
26	chr12	50348077	50348077	C	T	exonic	AQP2		nonsynonymous SNV	AQP2:NM_000486:exon2:c.C500T:p.S167F	NOVEL		153	46	0.570201	1	89	86	1.01589	1	126	93	1.05285	1
26	chr1	92707788	92707788	C	T	exonic	C1orf146		nonsynonymous SNV	C1orf146:NM_001012425:exon3:c.C86T:p.	DBSNP		198	0	0	1	220	0	0	1	57	42	1.05182	1
26	chr10	25464995	25464995	G	T	exonic	GPR158		nonsynonymous SNV	GPR158:NM_020752:exon1:c.G646T:p.A2	NOVEL		94	0	0	1	111	0	0	1	106	78	1.051	1
26	chr11	47599636	47599636	A	G	exonic	KBTBD4		synonymous SNV	KBTBD4:NM_001318717:exon2:c.T33C:p.D	NOVEL		40	38	1.20175	1	39	42	1.07189	1	26	19	1.04681	1
26	chr7	140373568	140373568	C	T	exonic	ADCK2		synonymous SNV	ADCK2:NM_052853:exon1:c.C438T:p.I146	NOVEL		314	137	1.05309	1	321	138	0.890431	1	505	154	1.04675	1
26	chr6	112460396	112460396	C	G	exonic	LAMA4		nonsynonymous SNV	LAMA4:NM_001105206:exon24:c.G3208C	NOVEL		183	0	0	1	201	0	0	1	84	61	1.04301	1
26	chr1	207110621	207110621	C	A	exonic	PIGR		nonsynonymous SNV	PIGR:NM_002644:exon4:c.G864T:p.K288N	NOVEL		402	90	0.532608	1	156	307	0.981886	2	178	266	1.04222	2
26	chr5	112175159	112175159	A	C	exonic	APC		nonsynonymous SNV	APC:NM_001127511:exon14:c.A3814C:p.N	NOVEL		150	143	1.2039	1	178	156	0.965527	1	101	73	1.04016	1
26	chr19	42879928	42879928	C	G	exonic	MEGF8		synonymous SNV	MEGF8:NM_001410:exon41:c.C7338G:p.V	NOVEL		289	0	0	1	210	0	0	1	220	98	1.03382	1
26	chr5	43487124	43487124	C	T	exonic	C5orf34		nonsynonymous SNV	C5orf34:NM_198566:exon13:c.G1810A:p.E	NOVEL		75	61	1.10641	1	67	56	0.941173	1	35	25	1.03304	1
26	chr17	46675366	46675366	C	G	exonic	HOXB6		nonsynonymous SNV	HOXB6:NM_001369397:exon2:c.G147C:p.Q	NOVEL		324	10	0.133735	1	314	1	0.0117083	1	202	60	1.02579	1
26	chr5	138860765	138860765	C	T	exonic	TMEM173		synonymous SNV	TMEM173:NM_001367258:exon3:c.G33A:	NOVEL		40	22	0.875295	1	32	21	0.819086	1	44	31	1.02477	1
26	chr19	44681138	44681138	C	T	exonic	ZNF226		stopgain	ZNF226:NM_001032372:exon6:c.C1723T:	NOVEL		213	18	0.324115	1	242	0	0	1	66	29	1.02405	1
26	chr6	168708823	168708823	G	T	exonic	DACT2		nonsynonymous SNV	DACT2:NM_001286350:exon3:c.C1104A:p.	NOVEL		91	72	1.0896	1	86	73	0.9491	1	74	52	1.0232	1
26	chr19	56538545	56538545	G	A	exonic	NLRP5		nonsynonymous SNV	NLRP5:NM_153447:exon7:c.G946A:p.V31	DBSNP+COSMIC	ID=COSM297008	488	217	1.06707	1	634	0	0	1	258	181	1.02221	1
26	chr15	90348372	90348372	G	A	exonic	ANPEP		synonymous SNV	ANPEP:NM_001150:exon4:c.C834T:p.Y278	NOVEL		254	0	0	1	207	0	0	1	147	103	1.02147	1
26	chr12	22040796	22040796	C	T	exonic	ABCC9		synonymous SNV	ABCC9:NM_005691:exon13:c.G1875A:p.S	DBSNP		235	24	0.228578	1	309	3	0.0198771	1	169	118	1.01936	1
26	chr21	47571511	47571511	G	A	exonic	FTCD;FTCD-AS1		synonymous SNV	FTCD-AS1:NM_001350598:exon1:c.G63A:	DBSNP		100	99	1.22717	1	109	79	0.868671	1	99	140	1.01904	2
26	chr11	62911122	62911122	T	A	exonic	SLC22A24		nonsynonymous SNV	SLC22A24:NM_001136506:exon1:c.A130T	NOVEL		161	126	1.08296	1	142	157	1.08546	1	115	80	1.01714	1
26	chr17	40475064	40475064	C	G	exonic	STAT3		nonsynonymous SNV	STAT3:NM_001369512:exon20:c.G1846C:	DBSNP+COSMIC	ID=COSM698670	85	7	0.187687	1	81	0	0	1	42	29	1.01267	1
26	chr12	85638568	85638568	C	T	exonic	LRRIQ1		nonsynonymous SNV	LRRIQ1:NM_001079910:exon27:c.C5018T	NOVEL		152	0	0	1	177	0	0	1	51	35	1.00901	1
26	chr15	43821936	43821936	C	T	exonic	MAP1A		synonymous SNV	MAP1A:NM_002373:exon5:c.C8124T:p.D2	NOVEL		163	58	0.909823	1	99	84	0.948887	1	84	57	1.00226	1
26	chr2	232196526	232196526	G	A	exonic	ARMC9		nonsynonymous SNV	ARMC9:NM_001352757:exon19:c.G1696A	NOVEL		177	0	0	1	177	0	0	1	87	59	1.0019	1
26	chr8	65509231	65509231	C	T	exonic	CYP7B1		nonsynonymous SNV	CYP7B1:NM_004820:exon6:c.G1489A:p.D	NOVEL		74	72	1.38448	1	86	154	0.950196	2	34	46	1.00029	2
26	chr8	114290919	114290919	T	G	exonic	CSMD3		nonsynonymous SNV	CSMD3:NM_001363185:exon3:c.A416C:p.	NOVEL		122	70	1.02354	1	250	130	1.0132	1	194	78	0.997736	1
26	chr12	49427369	49427369	G	A	exonic	KMT2D		stopgain	KMT2D:NM_003482:exon39:c.C11119T:p.	COSMIC	ID=COSM940014	157	122	1.07865	1	151	107	0.857335	1	148	99	0.993722	1
26	chr4	73414375	73414375	C	T	exonic	ADAMTS3		synonymous SNV	ADAMTS3:NM_014243:exon3:c.G324A:p.V	NOVEL		242	0	0	1	271	0	0	1	95	63	0.988576	1
26	chr15	45427446	45427446	G	A	exonic	DUOX1		stopgain	DUOX1:NM_175940:exon5:c.G452A:p.W1	NOVEL		165	76	1.09325	1	156	1	0.013167	1	78	51	0.980183	1
26	chr15	75248640	75248640	C	G	exonic	RPP25		synonymous SNV	RPP25:NM_017793:exon1:c.G285C:p.T95T	NOVEL		160	96	1.30003	1	223	1	0.00922865	1	104	68	0.980183	1
26	chr5	34923331	34923331	A	G	exonic	BRIX1		nonsynonymous SNV	BRIX1:NM_018321:exon8:c.A655G:p.N219	NOVEL		160	0	0	1	175	0	0	1	40	26	0.976688	1
26	chr19	10670353	10670353	G	A	exonic	KRI1		nonsynonymous SNV	KRI1:NM_023008:exon11:c.C959T:p.S320F	COSMIC	ID=COSM397067	392	147	1.1344	1	291	157	1.0749	1	449	139	0.972417	1
26	chr12	82147771	82147771	A	G	exonic	PPFIA2		nonsynonymous SNV	PPFIA2:NM_001220473:exon2:c.T230C:p.L	NOVEL		192	7	0.0867698	1	208	0	0	1	98	63	0.970155	1
26	chr19	9059068	9059068	G	T	exonic	MUC16		nonsynonymous SNV	MUC16:NM_024690:exon3:c.C28378A:p.P	NOVEL		489	41	0.321771	1	288	162	1.1042	1	315	97	0.968477	1
26	chr8	120581539	120581539	G	A	exonic	ENPP2		synonymous SNV	ENPP2:NM_001040092:exon21:c.C1989T:	NOVEL		263	12	0.122506	1	372	1	0.00794008	1	161	62	0.967335	1
26	chr7	99015188	99015188	C	T	exonic	BUD31		synonymous SNV	BUD31:NM_001370407:exon5:c.C354T:p.	NOVEL		266	66	0.689171	1	233	138	1.10164	1	189	143	0.964665	2
26	chr19	2408405	2408405	C	T	exonic	TMPRSS9		synonymous SNV	TMPRSS9:NM_182973:exon7:c.C792T:p.S	NOVEL		193	63	1.02362	1	161	62	0.852769	1	232	70	0.953468	1
26	chr3	48226174	48226174	C	G	exonic	CDC25A		nonsynonymous SNV	CDC25A:NM_001789:exon3:c.G259C:p.D8	NOVEL		77	33	1.04002	1	88	63	1.23566	1	24	9	0.948896	1
26	chr19	1031103	1031103	G	C	exonic	CNN2		nonsynonymous SNV	CNN2:NM_001303499:exon2:c.G97C:p.E3	NOVEL		167	6	0.144259	1	140	0	0	1	87	26	0.946476	1
26	chr8	143623655	143623655	A	G	exonic	ADGRB1		nonsynonymous SNV	ADGRB1:NM_001702:exon28:c.A4060G:p.	NOVEL		230	0	0	1	243	1	0.0121379	1	236	88	0.944991	1
26	chr3	194373477	194373477	C	T	exonic	LSG1		nonsynonymous SNV	LSG1:NM_018385:exon8:c.G1154A:p.G38	NOVEL		255	78	0.812029	1	251	96	0.819362	1	172	64	0.943535	1
26	chr10	37488686	37488686	G	A	exonic	ANKRD30A		synonymous SNV	ANKRD30A:NM_052997:exon30:c.G2580A	COSMIC	ID=COSM632800	165	45	0.528587	1	139	113	0.926967	1	70	43	0.943445	1
26	chr1	65278486	65278486	T	A	exonic	RAVER2		nonsynonymous SNV	RAVER2:NM_001366165:exon10:c.T1746A	NOVEL		337	0	0	1	374	0	0	1	129	79	0.941652	1
26	chr3	49698369	49698369	C	A	exonic	BSN		nonsynonymous SNV	BSN:NM_003458:exon6:c.C9091A:p.Q303	NOVEL		240	33	0.419057	1	194	96	0.980409	1	286	106	0.940827	1
26	chr21	22658632	22658632	A	G	exonic	NCAM2		synonymous SNV	NCAM2:NM_001352591:exon4:c.A381G:p.	NOVEL		310	0	0	1	377	0	0	1	243	90	0.940348	1
26	chr5	54674255	54674255	G	A	exonic	MTREX		nonsynonymous SNV	MTREX:NM_015360:exon17:c.G1924A:p.E	NOVEL		157	0	0	1	131	0	0	1	41	25	0.939123	1
26	chr4	79328926	79328926	C	T	exonic	FRAS1		synonymous SNV	FRAS1:NM_001166133:exon31:c.C4239T:	NOVEL		151	0	0	1	150	0	0	1	105	64	0.938901	1
26	chr20	33514000	33514000	A	T	exonic	ACSS2		stopgain	ACSS2:NM_001242393:exon16:c.A1534T:	NOVEL		228	1	0.0107718	1	240	0	0	1	253	93	0.935184	1
26	chr10	135215092	135215092	C	T	exonic	MTG1		stopgain	MTG1:NM_138384:exon7:c.C571T:p.Q191	NOVEL		228	0	0	1	241	0	0	1	124	75	0.934404	1
26	chr19	872057	872057	C	T	exonic	MED16		nonsynonymous SNV	MED16:NM_005481:exon12:c.G1967A:p.R	DBSNP		143	117	0.935882	2	161	84	1.05162	1	241	198	0.927653	2
26	chr19	41727828	41727828	C	T	exonic	AXL		synonymous SNV	AXL:NM_001699:exon4:c.C453T:p.A151A,	DBSNP		146	39	0.876863	1	100	48	0.994773	1	212	81	0.927394	1
26	chr3	184922495	184922495	G	A	exonic	EHHADH		nonsynonymous SNV	EHHADH:NM_001166415:exon6:c.C331T:p	NOVEL		100	70	1.42748	1	140	70	0.987217	1	94	34	0.924185	1
26	chr7	100174549	100174549	C	T	exonic	LRCH4		nonsynonymous SNV	LRCH4:NM_001289934:exon13:c.G1444A:	NOVEL		172	41	0.667307	1	162	64	0.838698	1	146	102	0.921143	2
26	chr6	43308644	43308644	A	C	exonic	ZNF318		nonsynonymous SNV	ZNF318:NM_014345:exon9:c.T3379G:p.S	NOVEL		262	0	0	1	277	0	0	1	128	75	0.915992	1
26	chr11	78380575	78380575	T	A	exonic	TENM4		nonsynonymous SNV	TENM4:NM_001098816:exon32:c.A6815T	NOVEL		269	0	0	1	295	0	0	1	198	116	0.915915	1
26	chr19	12187637	12187637	A	G	exonic	ZNF844		nonsynonymous SNV	ZNF844:NM_001136501:exon4:c.A1702G:	NOVEL		427	0	0	1	335	0	0	1	193	55	0.912275	1
26	chr19	38746880	38746880	G	T	exonic	PPP1R14A		nonsynonymous SNV	PPP1R14A:NM_001243947:exon1:c.C100A	NOVEL		97	0	0	1	89	0	0	1	105	39	0.90855	1
26	chr20	40161874	40161874	T	A	exonic	CHD6		nonsynonymous SNV	CHD6:NM_032221:exon3:c.A369T:p.E123D	NOVEL		78	0	0	1	85	0	0	1	17	6	0.90764	1
26	chr5	82838019	82838019	C	T	exonic	VCAN		nonsynonymous SNV	VCAN:NM_001164097:exon7:c.C6236T:p.S	NOVEL		341	22	0.149499	1	443	4	0.0184986	1	166	95	0.902422	1
26	chr19	55451254	55451254	G	A	exonic	NLRP7		synonymous SNV	NLRP7:NM_001127255:exon4:c.C933T:p.L	DBSNP		167	81	1.13228	1	208	0	0	1	158	89	0.893346	1
26	chr19	36038032	36038032	C	T	exonic	TMEM147		synonymous SNV	TMEM147:NM_001242598:exon4:c.C219T	DBSNP		325	33	0.383415	1	326	0	0	1	247	89	0.888582	1
26	chr3	49136370	49136370	T	A	exonic	QARS		nonsynonymous SNV	QARS:NM_001272073:exon19:c.A1778T:p	NOVEL		130	67	1.17904	1	143	66	0.935258	1	151	51	0.878434	1
26	chr19	8038749	8038749	C	A	exonic	ELAVL1		nonsynonymous SNV	ELAVL1:NM_001419:exon4:c.G290T:p.R97	COSMIC	ID=COSM320097	645	1	0.00643882	1	523	1	0.00585347	1	479	130	0.878094	1
26	chr1	180886999	180886999	G	T	exonic	KIAA1614		nonsynonymous SNV	KIAA1614:NM_020950:exon3:c.G1010T:p.	NOVEL		193	0	0	1	186	0	0	1	169	57	0.877519	1
26	chr17	9395166	9395166	T	A	exonic	STX8		nonsynonymous SNV	STX8:NM_004853:exon6:c.A521T:p.N174I	NOVEL		76	0	0	1	84	0	0	1	22	12	0.875042	1
26	chr19	7605066	7605066	C	T	exonic	PNPLA6		synonymous SNV	PNPLA6:NM_001166114:exon5:c.C586T:p.	NOVEL		235	0	0	1	193	0	0	1	234	63	0.872567	1
26	chr20	62384166	62384166	C	T	exonic	ZBTB46		nonsynonymous SNV	ZBTB46:NM_001369741:exon4:c.G1271A:	NOVEL		235	0	0	1	245	0	0	1	145	145	0.869821	2
26	chr15	73624602	73624602	G	A	exonic	HCN4		nonsynonymous SNV	HCN4:NM_005477:exon3:c.C1241T:p.A414	NOVEL		157	72	1.08998	1	174	4	0.0464543	1	93	50	0.866883	1
26	chr7	127251209	127251209	G	T	exonic	PAX4		nonsynonymous SNV	PAX4:NM_001366110:exon12:c.C965A:p.P	NOVEL		63	0	0	1	69	0	0	1	46	29	0.865995	2
26	chr15	41043761	41043761	G	C	exonic	RMDN3		nonsynonymous SNV	RMDN3:NM_001304802:exon4:c.C387G:p.	NOVEL		144	61	1.03157	1	183	0	0	1	105	56	0.86236	1
26	chr1	154543851	154543851	C	A	exonic	CHRNB2		nonsynonymous SNV	CHRNB2:NM_000748:exon5:c.C552A:p.D1	NOVEL		541	1	0.0063962	1	559	0	0	1	368	226	0.85212	2
26	chr7	141872515	141872515	A	C	exonic	MGAM2		nonsynonymous SNV	MGAM2:NM_001293626:exon30:c.A3512	NOVEL		324	0	0	1	404	0	0	1	211	129	0.849747	2
26	chr13	70314560	70314560	G	A	exonic	KLHL1		nonsynonymous SNV	KLHL1:NM_001286725:exon7:c.C1585T:p.R	DBSNP+COSMIC	ID=COSM669643	118	0	0	1	158	0	0	1	50	26	0.848177	1
26	chr8	87242191	87242191	C	T	exonic	SLC7A13		nonsynonymous SNV	SLC7A13:NM_138817:exon1:c.G316A:p.G1	NOVEL		294	0	0	1	402	0	0	1	146	47	0.847287	1
26	chr3	62142819	62142819	G	T	exonic	PTPRG		nonsynonymous SNV	PTPRG:NM_002841:exon7:c.G761T:p.G25	NOVEL		214	0	0	1	239	0	0	1	140	45	0.846313	1
26	chr15	83826295	83826295	C	T	exonic	HDGFL3		nonsynonymous SNV	HDGFL3:NM_016073:exon4:c.G331A:p.E1	NOVEL		236	38	0.480789	1	250	1	0.00823593	1	91	47	0.844394	1
26	chr7	122809355	122809355	A	C	exonic	SLC13A1		nonsynonymous SNV	SLC13A1:NM_022444:exon4:c.T400G:p.L1	NOVEL		121	0	0	1	124	0	0	1	48	11	0.835121	1
26	chr9	103102649	103102649	C	T	exonic	TEX10		synonymous SNV	TEX10:NM_001161584:exon5:c.G1149A:p.	NOVEL		211	0	0	1	208	0	0	1	70	22	0.832003	1
26	chr12	21790040	21790040	G	A	exonic	LDHB		synonymous SNV	LDHB:NM_001174097:exon7:c.C802T:p.L2	NOVEL		41	32	1.08131	1	34	32	1.00229	1	18	9	0.826429	1
26	chr15	51975299	51975299	G	T	exonic	SCG3		nonsynonymous SNV	SCG3:NM_013243:exon3:c.G159T:p.K53N	COSMIC	ID=COSM962751	76	30	0.981153	1	55	55	1.03361	1	15	28	0.807209	2
26	chr21	41710039	41710039	A	C	exonic	DSCAM		nonsynonymous SNV	DSCAM:NM_001271534:exon8:c.T1772G:	NOVEL		32	49	1.49223	1	54	48	0.972809	1	22	19	0.806176	2
26	chr19	15198734	15198734	C	A	exonic	OR1I1		nonsynonymous SNV	OR1I1:NM_001004713:exon1:c.C858A:p.N	COSMIC	ID=COSM126008	286	5	0.0714687	1	282	0	0	1	272	66	0.803234	1
26	chr19	18245485	18245485	C	A	exonic	MAST3		nonsynonymous SNV	MAST3:NM_015016:exon15:c.C1581A:p.H	NOVEL		259	93	1.09895	1	212	80	0.840334	1	370	89	0.797613	1
26	chr19	7935968	7935968	G	A	exonic	PRR36		unknown	UNKNOWN	DBSNP		149	38	0.845241	1	147	0	0	1	51	12	0.78353	1
26	chr5	140362110	140362110	C	T	exonic	PCDHA1;PCDHA10		nonsynonymous SNV	PCDHAC1:NM_018898:exon3:c.C2543T:p.P	COSMIC	ID=COSM361034	82	0	0	1	102	0	0	1	51	33	0.778866	1
26	chr1	60456475	60456475	A	G	exonic	C1orf87		nonsynonymous SNV	C1orf87:NM_152377:exon12:c.T1511C:p.L	NOVEL		46	36	1.08296	1	57	34	0.772367	1	12	20	0.774777	2
26	chr17	73627631	73627631	G	A	exonic	RECQL5		synonymous SNV	RECQL5:NM_004259:exon9:c.C1347T:p.R4	NOVEL		53	52	1.22162	1	34	47	1.1995	1	38	63	0.773243	2
26	chr19	39912881	39912881	G	A	exonic	PLEKHG2		nonsynonymous SNV	PLEKHG2:NM_001351693:exon17:c.G1453	NOVEL		359	35	0.369497	1	348	0	0	1	277	82	0.766242	1
26	chr19	9640310	9640310	T	G	exonic	ZNF426		nonsynonymous SNV	ZNF426:NM_001300883:exon6:c.A297C:p.	NOVEL		695	0	0	1	556	0	0	1	392	87	0.747134	1
26	chr17	41001386	41001386	G	T	exonic	AOC2		synonymous SNV	AOC2:NM_009590:exon2:c.G1872T:p.G62	DBSNP		202	0	0	1	209	0	0	1	209	57	0.745561	1
26	chr5	137216517	137216517	G	C	exonic	MYOT		nonsynonymous SNV	MYOT:NM_001135940:exon5:c.G94C:p.E3	DBSNP		138	10	0.166672	1	162	3	0.0375858	1	54	23	0.740566	1
26	chr8	101609015	101609015	C	A	exonic	SNX31		nonsynonymous SNV	SNX31:NM_001363720:exon7:c.G533T:p.C	NOVEL		61	17	0.611874	1	75	36	0.960535	1	105	27	0.711672	1
26	chr20	55052138	55052138	T	C	exonic	RTF2		nonsynonymous SNV	RTF2:NM_001283036:exon4:c.T356C:p.F1	NOVEL		316	0	0	1	325	0	0	1	289	71	0.686193	1
26	chr4	128689970	128689970	A	G	exonic	SLC25A31		nonsynonymous SNV	SLC25A31:NM_001318467:exon5:c.A697G	NOVEL		159	0	0	1	201	0	0	1	84	31	0.668329	1
26	chr16	2103363	2103363	G	A	exonic	TSC2		stopgain	TSC2:NM_001318829:exon3:c.G99A:p.W3	COSMIC	ID=COSM734907	51	0	0	1	56	0	0	1	17	6	0.64677	1
26	chr5	6749688	6749688	C	T	exonic	TENT4A		synonymous SNV	TENT4A:NM_001171805:exon9:c.C1605T:	NOVEL		70	0	0	1	113	0	0	1	106	24	0.64233	1
26	chr15	43258371	43258371	G	A	exonic	UBR1		nonsynonymous SNV	UBR1:NM_174916:exon41:c.C4571T:p.P15	DBSNP		314	0	0	1	228	0	0	1	161	55	0.6313	1
26	chr9	123476106	123476106	G	A	exonic	MEGF9		synonymous SNV	MEGF9:NM_001080497:exon1:c.C531T:p.L	NOVEL		55	0	0	1	74	0	0	1	37	8	0.61854	1
26	chr8	133595986	133595986	G	C	exonic	LRRC6		nonsynonymous SNV	LRRC6:NM_001321962:exon9:c.C935G:p.S	NOVEL		266	0	0	1	364	0	0	1	161	33	0.591837	1
26	chr4	57797283	57797283	G	A	exonic	REST		nonsynonymous SNV	REST:NM_001193508:exon4:c.G2259A:p.N	DBSNP+COSMIC	ID=COSM157942	68	0	0	1	80	1	0.0255212	1	32	9	0.544233	1
26	chr12	25378643	25378643	C	G	exonic	KRAS		nonsynonymous SNV	KRAS:NM_001369786:exon4:c.G355C:p.D1	NOVEL		90	0	0	1	96	0	0	1	58	14	0.482083	1
26	chr12	111748379	111748379	G	A	exonic	CUX2		stopgain	CUX2:NM_001370598:exon15:c.G1607A:p	NOVEL		139	0	0	1	69	63	0.986627	1	132	19	0.311963	1
26	chr20	57429719	57429719	G	T	exonic	GNAS		nonsynonymous SNV	GNAS:NM_001077490:exon1:c.G1212T:p.	DBSNP		90	0	0	1	102	0	0	1	103	10	0.307901	1
26	chr15	81224317	81224317	G	A	exonic	CEMIP		synonymous SNV	CEMIP:NM_018689:exon21:c.G2730A:p.L9	COSMIC	ID=COSM174934	390	0	0	1	190	169	0.973147	1	229	30	0.287176	1
26	chr3	56807829	56807829	G	A	exonic	ARHGEF3		nonsynonymous SNV	ARHGEF3:NM_001128616:exon2:c.C130T:	DBSNP		179	0	0	1	112	58	1.01045	1	89	8	0.286951	1
26	chr20	24939603	24939603	G	A	exonic	CST7		nonsynonymous SNV	CST7:NM_003650:exon3:c.G283A:p.G95S	NOVEL		107	0	0	1	108	0	0	1	135	17	0.277289	1
26	chr12	129178499	129178499	G	A	exonic	TMEM132C		synonymous SNV	TMEM132C:NM_001136103:exon6:c.G157	NOVEL		135	0	0	1	156	0	0	1	123	13	0.236991	1
26	chr17	77808835	77808835	C	T	exonic	CBX4		synonymous SNV	CBX4:NM_003655:exon5:c.G606A:p.A202	NOVEL		205	0	0	1	178	0	0	1	88	9	0.230037	1
26	chr8	55540381	55540381	G	T	exonic	RP1		nonsynonymous SNV	RP1:NM_006269:exon4:c.G3939T:p.K1313	NOVEL		545	3	0.0153691	1	606	234	0.825031	1	659	46	0.227017	1
26	chr2	220173946	220173946	C	T	exonic	PTPRN		nonsynonymous SNV	PTPRN:NM_001199763:exon1:c.G109A:p.	NOVEL		101	0	0	1	54	51	1.00408	1	151	15	0.224032	1
26	chr19	10103701	10103701	G	A	exonic	COL5A3		nonsynonymous SNV	COL5A3:NM_015719:exon20:c.C1792T:p.P	NOVEL		425	0	0	1	261	114	0.932434	1	350	20	0.222353	1
26	chr14	105418520	105418520	T	C	exonic	AHNAK2		nonsynonymous SNV	AHNAK2:NM_001350929:exon7:c.A2968G	NOVEL		148	0	0	1	120	6	0.098439	1	99	8	0.2215	1
26	chr15	77907123	77907123	G	A	exonic	LINGO1		nonsynonymous SNV	LINGO1:NM_032808:exon2:c.C1126T:p.R3	NOVEL		185	15	0.260006	1	105	90	0.954101	1	154	15	0.220055	1
26	chr19	47424945	47424945	C	T	exonic	ARHGAP35		synonymous SNV	ARHGAP35:NM_004491:exon2:c.C3013T:p	DBSNP		466	152	1.02304	1	399	174	0.931407	1	377	26	0.216429	1
26	chr15	52074983	52074983	G	A	exonic	TMOD2		synonymous SNV	TMOD2:NM_014548:exon7:c.G690A:p.K2	NOVEL		230	20	0.277339	1	132	117	0.971344	1	149	14	0.212945	1
26	chr1	48771547	48771547	C	T	exonic	SPATA6		nonsynonymous SNV	SPATA6:NM_001286239:exon11:c.G1156A	NOVEL		160	0	0	1	115	85	0.878568	1	87	8	0.208782	1
26	chr8	16032718	16032718	G	A	exonic	MSR1		synonymous SNV	MSR1:NM_001363744:exon3:c.C249T:p.I8	DBSNP+COSMIC	ID=COSM364681	338	0	0	1	275	0	0	1	118	19	0.205156	1
26	chr11	123848098	123848098	G	T	exonic	OR10S1		nonsynonymous SNV	OR10S1:NM_001004474:exon1:c.C301A:p.	NOVEL		168	0	0	1	180	0	0	1	128	11	0.196202	1
26	chr8	22012934	22012934	C	T	exonic	LGI3		synonymous SNV	LGI3:NM_139278:exon2:c.G249A:p.A83A	DBSNP+COSMIC	ID=COSM109804	84	0	0	1	58	7	0.114931	1	59	9	0.195788	1
26	chr20	40733264	40733264	T	A	exonic	PTPRT		nonsynonymous SNV	PTPRT:NM_007050:exon25:c.A3485T:p.N1	NOVEL		226	0	0	1	193	7	0.0723526	1	204	12	0.193294	1
26	chr19	45663406	45663406	C	T	exonic	NKPD1		startloss	NKPD1:NM_198478:exon1:c.G3A:p.M1?	NOVEL		389	106	0.890716	1	282	122	0.926239	1	429	26	0.191694	1
26	chr12	7637708	7637708	G	T	exonic	CD163		synonymous SNV	CD163:NM_001370145:exon11:c.C2763A:	NOVEL		95	0	0	1	56	47	0.943294	1	109	9	0.189098	1
26	chr10	135372426	135372426	C	G	exonic	SYCE1		nonsynonymous SNV	SYCE1:NM_001143763:exon4:c.G226C:p.E	NOVEL		250	0	0	1	296	0	0	1	187	15	0.184105	1
26	chr11	64000002	64000002	G	A	exonic	DNAJC4		nonsynonymous SNV	DNAJC4:NM_001307980:exon3:c.G281A:p	DBSNP		220	0	0	1	193	0	0	1	256	20	0.179658	1
26	chr10	135372423	135372423	C	G	exonic	SYCE1		nonsynonymous SNV	SYCE1:NM_001143763:exon4:c.G229C:p.A	DBSNP		242	0	0	1	289	0	0	1	182	14	0.177092	1
26	chr1	110050559	110050559	G	C	exonic	AMIGO1		nonsynonymous SNV	AMIGO1:NM_020703:exon2:c.C976G:p.L3	NOVEL		437	1	0.00760539	1	186	167	0.977976	1	368	28	0.175303	1
26	chr6	13588690	13588690	A	T	exonic	SIRT5		nonsynonymous SNV	SIRT5:NM_001193267:exon4:c.A243T:p.Q	NOVEL		178	0	0	1	182	13	0.137815	1	156	11	0.163306	1
26	chr15	90903897	90903897	C	G	exonic	ZNF774		nonsynonymous SNV	ZNF774:NM_001004309:exon4:c.C834G:p	NOVEL		190	86	1.08022	1	106	103	1.01877	1	143	9	0.1468	1
26	chr5	76249920	76249920	G	T	exonic	CRHBP		nonsynonymous SNV	CRHBP:NM_001882:exon3:c.G242T:p.C81F	COSMIC	ID=COSM166127	221	0	0	1	113	114	1.03816	1	317	19	0.140198	1
26	chr1	52205860	52205860	G	A	exonic	OSBPL9		nonsynonymous SNV	OSBPL9:NM_148909:exon5:c.G364A:p.V1	NOVEL		161	0	0	1	162	8	0.0972809	1	84	5	0.139286	1
26	chr10	122665391	122665391	C	A	exonic	WDR11		nonsynonymous SNV	WDR11:NM_018117:exon27:c.C3295A:p.R	NOVEL		186	0	0	1	234	0	0	1	101	6	0.139025	1
26	chr5	76249921	76249921	C	T	exonic	CRHBP		synonymous SNV	CRHBP:NM_001882:exon3:c.C243T:p.C81C	COSMIC	ID=COSM564687	221	0	0	1	113	116	1.04715	1	320	19	0.138957	1
26	chr7	143056064	143056064	C	T	exonic	FAM131B		nonsynonymous SNV	FAM131B:NM_001278297:exon4:c.G40A:	COSMIC	ID=COSM387894	320	0	0	1	297	11	0.105773	1	502	15	0.12996	1
26	chr7	42005747	42005747	G	T	exonic	GLI3		nonsynonymous SNV	GLI3:NM_000168:exon15:c.C2924A:p.A97	NOVEL		139	0	0	1	156	14	0.243901	1	105	4	0.12768	1
26	chr16	4245721	4245721	C	T	exonic	SRL		nonsynonymous SNV	SRL:NM_001098814:exon5:c.G443A:p.G14	NOVEL		200	0	0	1	185	18	0.1833	1	94	5	0.125216	1
26	chr7	21805073	21805073	C	A	exonic	DNAH11		nonsynonymous SNV	DNAH11:NM_001277115:exon55:c.C8968	NOVEL		314	0	0	1	367	18	0.138467	1	351	13	0.12426	1
26	chr14	38276606	38276606	A	C	exonic	TTC6		nonsynonymous SNV	TTC6:NM_001310135:exon25:c.A4444C:p.	NOVEL		225	0	0	1	282	0	0	1	296	10	0.113702	1
26	chr6	83938585	83938585	T	C	exonic	ME1		synonymous SNV	ME1:NM_002395:exon10:c.A1092G:p.L364	NOVEL		254	0	0	1	257	15	0.114001	1	213	10	0.111179	1
26	chr12	9994946	9994946	A	G	exonic	KLRF1		synonymous SNV	KLRF1:NM_001291822:exon4:c.A354G:p.Q	NOVEL		315	1	0.00780614	1	344	16	0.0918764	1	277	12	0.102946	1
26	chr10	102732712	102732712	G	A	exonic	SEMA4G		nonsynonymous SNV	SEMA4G:NM_001203244:exon1:c.G43A:p.	DBSNP		208	1	0.0118026	1	200	1	0.0102847	1	238	10	0.0999712	1
26	chr16	88805100	88805100	C	G	exonic	PIEZO1		synonymous SNV	PIEZO1:NM_001142864:exon6:c.G510C:p.	NOVEL		157	0	0	1	143	31	0.368298	1	239	10	0.0995697	1

26	chr4	155241559	155241559	G	A	exonic	DCHS2		synonymous SNV	DCHS2:NM_001358235:exon9:c.C4992T:p.	NOVEL		347	0	0	1	397	0	0	1	242	10	0.0983844	1
26	chr3	48620997	48620997	C	A	exonic	COL7A1		nonsynonymous SNV	COL7A1:NM_000094:exon40:c.G4393T:p.C	NOVEL		289	0	0	1	196	68	0.762849	1	212	6	0.0957602	1
26	chr3	48620996	48620996	C	A	exonic	COL7A1		nonsynonymous SNV	COL7A1:NM_000094:exon40:c.G4394T:p.C	DBSNP		288	1	0.0119956	1	196	67	0.754489	1	214	6	0.0948896	1
26	chr11	67164740	67164740	G	T	exonic	RAD9A		synonymous SNV	RAD9A:NM_001243224:exon7:c.G735T:p.\	DBSNP		240	0	0	1	137	98	0.862074	1	414	14	0.0810981	1
26	chr16	67328548	67328548	T	A	exonic	KCTD19		nonsynonymous SNV	KCTD19:NM_001100915:exon11:c.A1525T	NOVEL		164	0	0	1	100	30	0.47705	1	183	6	0.0787075	1
26	chr11	61503789	61503789	G	C	exonic	DAGLA		nonsynonymous SNV	DAGLA:NM_006133:exon13:c.G1338C:p.E	NOVEL		127	0	0	1	106	22	0.355303	1	154	5	0.077965	1
26	chr12	8203187	8203187	C	T	exonic	FOXJ2		nonsynonymous SNV	FOXJ2:NM_018416:exon10:c.C1607T:p.T5	NOVEL		120	0	0	1	98	59	0.776853	1	185	5	0.0652444	1
26	chr11	113780132	113780132	C	T	exonic	HTR3B		synonymous SNV	HTR3B:NM_001363563:exon1:c.C135T:p.A	NOVEL		264	0	0	1	258	58	0.379426	1	244	6	0.0595029	1
26	chr1	78392222	78392222	G	T	exonic	NEXN		stopgain	NEXN:NM_001172309:exon6:c.G421T:p.E	NOVEL		167	0	0	1	172	12	0.134819	1	82	2	0.0590306	1
26	chr18	46287950	46287950	G	A	exonic	CTIF		nonsynonymous SNV	CTIF:NM_014772:exon9:c.G1261A:p.A421	NOVEL		292	0	0	1	190	126	0.824271	1	291	7	0.0582383	1
26	chr7	97822838	97822838	C	T	exonic	LMTK2		synonymous SNV	LMTK2:NM_014916:exon11:c.C3061T:p.L1	NOVEL		730	0	0	1	671	101	0.38747	1	1049	12	0.0506611	1
26	chr7	155534783	155534783	G	A	exonic	RBM33		nonsynonymous SNV	RBM33:NM_053043:exon13:c.G2320A:p.A	NOVEL		294	0	0	1	249	38	0.392135	1	216	2	0.0410944	1
26	chr3	48642159	48642159	C	T	exonic	UQCRC1		nonsynonymous SNV	UQCRC1:NM_003365:exon4:c.G352A:p.A1	DBSNP		212	2	0.0323994	1	192	26	0.353224	1	268	2	0.0257725	1
26	chr20	16488639	16488639	G	A	exonic	KIF16B		synonymous SNV	KIF16B:NM_001199865:exon7:c.C663T:p.S	DBSNP		152	0	0	1	155	39	0.415575	1	134	1	0.0183651	1
26	chr1	115256530	115256530	G	T	exonic	NRAS		nonsynonymous SNV	NRAS:NM_002524:exon3:c.C181A:p.Q61K	DBSNP+COSMIC	ID=COSM580;OC	226	43	0.532491	1	240	0	0	1	142	1	0.0173377	1
26	chr19	36508798	36508798	G	T	exonic	CLIP3		nonsynonymous SNV	CLIP3:NM_001199570:exon9:c.C1279A:p.C	NOVEL		309	0	0	1	227	40	0.443693	1	289	1	0.0115677	1
26	chr1	99772470	99772470	C	A	exonic	PLPPR4		synonymous SNV	PLPPR4:NM_001166252:exon6:c.C2022A:p	NOVEL		277	21	0.173831	1	344	0	0	1	254	1	0.00972269	1
26	chr1	151162513	151162513	A	C	exonic	VPS72		nonsynonymous SNV	VPS72:NM_001271087:exon1:c.T85G:p.F2	NOVEL		376	118	0.828088	1	489	0	0	1	738	1	0.00606128	1
26	chr9	35826060	35826060	G	T	exonic	FAM221B		nonsynonymous SNV	FAM221B:NM_001012446:exon2:c.C99A:p	NOVEL		119	0	0	1	60	23	0.572844	1	78	0	0	1
26	chr3	151166809	151166809	C	T	exonic	IGSF10		nonsynonymous SNV	IGSF10:NM_178822:exon4:c.G960A:p.M32	NOVEL		686	0	0	1	648	115	0.446383	1	496	0	0	1
26	chr10	115537197	115537197	T	A	exonic	PLEKHS1		nonsynonymous SNV	PLEKHS1:NM_182601:exon12:c.T1196A:p.\	NOVEL		347	0	0	1	314	84	0.436297	1	288	0	0	1
26	chr11	3380824	3380824	C	T	exonic	ZNF195		nonsynonymous SNV	ZNF195:NM_007152:exon4:c.G1198A:p.G4	NOVEL		211	1	0.0116356	1	191	48	0.415174	1	174	0	0	1
26	chr8	38876392	38876392	A	T	exonic	ADAM9		synonymous SNV	ADAM9:NM_003816:exon7:c.A627T:p.P20	NOVEL		304	0	0	1	124	74	0.398859	1	156	0	0	1
26	chr8	30695509	30695509	T	C	exonic	TEX15		nonsynonymous SNV	TEX15:NM_001350162:exon10:c.A8291G:p	NOVEL		227	0	0	1	105	61	0.392171	1	102	0	0	1
26	chr1	247419444	247419444	C	A	exonic	VN1R5		unknown	UNKNOWN	NOVEL		266	1	0.0128623	1	234	34	0.375732	1	86	0	0	1
26	chr14	21831073	21831073	T	A	exonic	SUPT16H		synonymous SNV	SUPT16H:NM_007192:exon14:c.A1545T:p	NOVEL		261	0	0	1	253	55	0.369146	1	259	0	0	1
26	chr19	47425220	47425220	G	T	exonic	ARHGAP35		nonsynonymous SNV	ARHGAP35:NM_004491:exon2:c.G3288T:p	NOVEL		534	2	0.0155204	1	405	54	0.360849	1	325	0	0	1
26	chr3	38355227	38355227	C	A	exonic	SLC22A14		synonymous SNV	SLC22A14:NM_004803:exon7:c.C1173A:p.\	NOVEL		189	0	0	1	162	21	0.339862	1	186	0	0	1
26	chr7	42088202	42088202	G	T	exonic	GLI3		synonymous SNV	GLI3:NM_000168:exon5:c.C567A:p.P189P	NOVEL		115	0	0	1	153	16	0.280393	1	91	0	0	1
26	chr3	121417650	121417650	C	T	exonic	GOLGB1		nonsynonymous SNV	GOLGB1:NM_001256488:exon12:c.G1480	NOVEL		380	1	0.00909906	1	360	36	0.269241	1	180	0	0	1
26	chr5	68728448	68728448	G	T	exonic	MARVELD2		nonsynonymous SNV	MARVELD2:NM_001244734:exon3:c.G124	DBSNP		277	0	0	1	307	21	0.132352	1	244	0	0	1
26	chr5	41042290	41042290	C	T	exonic	MROH2B		synonymous SNV	MROH2B:NM_173489:exon19:c.G1857A:p	NOVEL		153	0	0	1	183	9	0.0969009	1	52	0	0	1
26	chr17	80274718	80274718	G	A	exonic	CD7		synonymous SNV	CD7:NM_006137:exon2:c.C222T:p.D74D	DBSNP		168	9	0.125428	1	160	1	0.0128399	1	243	0	0	1
26	chr4	84227376	84227376	C	T	exonic	HPSE		nonsynonymous SNV	HPSE:NM_001199830:exon8:c.G1012A:p.E	NOVEL		240	77	0.599177	1	304	1	0.00677776	1	203	0	0	1
26	chr19	56104563	56104563	C	T	exonic	FIZ1		synonymous SNV	FIZ1:NM_032836:exon3:c.G744A:p.L248L	DBSNP		52	20	0.962984	1	75	0	0	1	61	0	0	1
26	chr1	109815325	109815325	C	T	exonic	CELSR2		synonymous SNV	CELSR2:NM_001408:exon30:c.C8118T:p.F2	NOVEL		170	62	0.890225	1	169	0	0	1	176	0	0	1
26	chr1	109815458	109815458	C	T	exonic	CELSR2		nonsynonymous SNV	CELSR2:NM_001408:exon31:c.C8147T:p.P2	NOVEL		230	81	0.867602	1	208	0	0	1	296	0	0	1
26	chr1	203152802	203152802	C	T	exonic	CHI3L1		synonymous SNV	CHI3L1:NM_001276:exon5:c.G432A:p.R14	COSMIC	ID=COSM571945	109	37	0.844199	1	143	0	0	1	104	0	0	1
26	chr9	88694209	88694209	G	A	exonic	GOLM1		synonymous SNV	GOLM1:NM_016548:exon2:c.C27T:p.R9R,(	NOVEL		113	32	0.765074	1	156	0	0	1	168	0	0	1
26	chr15	42110429	42110429	G	A	exonic	MAPKBP1		nonsynonymous SNV	MAPKBP1:NM_001265611:exon18:c.G201	NOVEL		158	41	0.714253	1	142	0	0	1	145	0	0	1
26	chr16	66942382	66942382	G	A	exonic	CDH16		synonymous SNV	CDH16:NM_001204746:exon17:c.C2112T:p	NOVEL		101	40	0.699785	1	147	0	0	1	151	0	0	1
26	chr13	25744033	25744033	G	A	exonic	AMER2		synonymous SNV	AMER2:NM_152704:exon1:c.C1725T:p.N5	DBSNP		181	68	0.673648	1	267	0	0	1	222	0	0	1
26	chr17	41367909	41367909	C	T	exonic	TMEM106A		synonymous SNV	TMEM106A:NM_001291588:exon4:c.C378	NOVEL		135	32	0.664286	1	197	0	0	1	296	0	0	1
26	chr10	112838897	112838897	G	A	exonic	ADRA2A		synonymous SNV	ADRA2A:NM_000681:exon1:c.G1143A:p.Q	COSMIC	ID=COSM150452	146	52	0.647831	1	214	0	0	1	140	0	0	1
26	chr17	72999981	72999981	G	T	exonic	CDR2L		nonsynonymous SNV	CDR2L:NM_014603:exon5:c.G1210T:p.G4	NOVEL		89	27	0.574155	1	109	0	0	1	82	0	0	1
26	chr10	124457863	124457863	G	A	exonic	C10orf120		nonsynonymous SNV	C10orf120:NM_001010912:exon3:c.C394T	COSMIC	ID=COSM134649	287	86	0.568739	1	447	0	0	1	233	0	0	1
26	chr13	99947863	99947863	A	T	exonic	GPR183		synonymous SNV	GPR183:NM_004951:exon2:c.T537A:p.I17	NOVEL		270	78	0.55289	1	375	0	0	1	229	0	0	1
26	chr5	52160886	52160886	C	T	exonic	ITGA1		stopgain	ITGA1:NM_181501:exon5:c.C466T:p.Q156	NOVEL		422	120	0.546142	1	652	0	0	1	449	0	0	1
26	chr15	59752316	59752316	G	A	exonic	FAM81A		nonsynonymous SNV	FAM81A:NM_152450:exon3:c.G205A:p.G	NOVEL		307	57	0.542869	1	318	0	0	1	214	0	0	1
26	chr8	139164786	139164786	T	C	exonic	FAM135B		nonsynonymous SNV	FAM135B:NM_001362965:exon13:c.A193	NOVEL		268	64	0.54119	1	503	0	0	1	248	0	0	1
26	chr8	77616446	77616446	C	G	exonic	ZFHX4		nonsynonymous SNV	ZFHX4:NM_024721:exon2:c.C123G:p.D41E	NOVEL		183	43	0.534155	1	329	0	0	1	202	0	0	1
26	chr5	7866886	7866886	G	A	exonic	FASTKD3		synonymous SNV	FASTKD3:NM_024091:exon2:c.C1311T:p.F	NOVEL		164	45	0.531116	1	224	0	0	1	132	0	0	1
26	chr20	36869562	36869562	G	A	exonic	KIAA1755		nonsynonymous SNV	KIAA1755:NM_001029864:exon3:c.C971T:	DBSNP		167	45	0.523601	1	250	0	0	1	229	0	0	1
26	chr3	42740363	42740363	G	T	exonic	HHATL		nonsynonymous SNV	HHATL:NM_020707:exon5:c.C320A:p.A107	NOVEL		197	35	0.523	1	237	0	0	1	263	0	0	1
26	chr3	4738919	4738919	G	A	exonic	ITPR1		stopgain	ITPR1:NM_001168272:exon33:c.G4325A:p	NOVEL		290	46	0.474613	1	392	0	0	1	360	0	0	1
26	chr7	5104994	5104994	C	T	exonic	RBAK		nonsynonymous SNV	RBAK:NM_021163:exon5:c.C1907T:p.S636	NOVEL		114	25	0.443658	1	168	0	0	1	72	0	0	1
26	chr9	131447459	131447459	G	C	exonic	SET		nonsynonymous SNV	SET:NM_001248000:exon1:c.G13C:p.D5H	NOVEL		178	8	0.149107	1	210	0	0	1	141	0	0	1
26	chr6	41318505	41318505	C	T	exonic	NCR2		nonsynonymous SNV	NCR2:NM_004828:exon5:c.C734T:p.P245L	COSMIC	ID=COSM362721	141	8	0.132442	1	175	0	0	1	126	0	0	1
26	chr6	108029118	108029118	G	A	exonic	SCML4		synonymous SNV	SCML4:NM_001286409:exon3:c.C345T:p.D	DBSNP+COSMIC	ID=COSM506131	128	7	0.0760532	1	156	0	0	1	193	0	0	1
26	chr7	138763849	138763849	C	T	splicing	ZC3HAV1	NM_001363491:exon4			NOVEL		75	35	1.10305	1	75	34	0.923818	1	66	27	1.30044	1
26	chr11	121466482	121466482	G	A	splicing	SORL1	NM_003105:exon32:c.			NOVEL		237	0	0	1	267	0	0	1	97	84	1.15061	1
26	chr6	34937785	34937785	A	T	splicing	ANKS1A	NM_015245:exon3:c.2			NOVEL		85	0	0	1	87	0	0	1	51	44	1.1483	1
26	chr11	10015546	10015546	C	T	splicing	SBF2	NM_030962:exon10:c			NOVEL		61	0	0	1	46	31	0.832257	1	31	2	0.15026	1
26	chr18	24442562	24442562	T	C	splicing	AQP4	NM_001364287:exon2			NOVEL		173	1	0.0141767	1	179	0	0	1	116	6	0.121932	1
26	chr3	175165161	175165161	G	T	splicing	NAALADL2	NM_207015:exon6:c.1			NOVEL		316	0	0	1	276	90	0.728275	1	239	5	0.0712968	1
26	chr9	111659567	111659567	T	C	splicing	ELP1	NM_003640:exon23:c.			NOVEL		152	0	0	1	163	20	0.323678	1	154	3	0.0664832	1
27	chrX	117035861	117035861	C	T	exonic	KLHL13		nonsynonymous SNV	KLHL13:NM_001168300:exon6:c.C1397A:p	NOVEL		158	7	NA	1	143	0	NA	1	141	0	NA	1
27	chrX	142717391	142717391	G	T	exonic	SLITRK4		nonsynonymous SNV	SLITRK4:NM_001184749:exon2:c.C1534A:	NOVEL		7	395	NA	1	0	369	NA	1	0	283	NA	1
27	chr3	151056456	151056456	T	A	exonic	P2RY12		nonsynonymous SNV	P2RY12:NM_176876:exon2:c.A178T:p.I60F	NOVEL		194	90	0.694724	1	136	152	1.09868	1	80	102	1.1423	1
27	chr4	135121223	135121223	A	T	exonic	PABPC4L		nonsynonymous SNV	PABPC4L:NM_001114734:exon2:c.T952A:p	NOVEL		190	140	0.930042	1	168	156	1.0023	1	142	177	1.13093	1
27	chr4	135121224	135121224	T	A	exonic	PABPC4L		synonymous SNV	PABPC4L:NM_001114734:exon2:c.A951T:p	NOVEL		192	141	0.928246	1	172	157	0.993395	1	143	178	1.13023	1
27	chr11	47369426	47369426	A	G	exonic	MYBPC3		nonsynonymous SNV	MYBPC3:NM_000256:exon7:c.T803C:p.L26	NOVEL		61	0	0	1	39	38	1.02733	1	35	43	1.12364	1
27	chr16	1498726	1498726	G	A	exonic	CLCN7		synonymous SNV	CLCN7:NM_001114331:exon19:c.C1767T:p	NOVEL		51	40	0.963623	1	66	68	1.05639	1	120	147	1.12217	1
27	chr22	28693821	28693821	A	G	exonic	TTC28		synonymous SNV	TTC28:NM_001145418:exon4:c.T549C:p.Y	DBSNP+COSMIC	ID=COSM330400	42	24	0.797179	1	38	24	0.80582	1	29	35	1.11465	1
27	chr18	8376191	8376191	C	A	exonic	PTPRM		nonsynonymous SNV	PTPRM:NM_002845:exon23:c.C3280A:p.H	NOVEL		169	0	0	1	113	99	0.972115	1	75	90	1.11176	1
27	chr5	145641358	145641358	G	A	exonic	RBM27		nonsynonymous SNV	RBM27:NM_018989:exon13:c.G2179A:p.G	NOVEL		62	38	0.833052	1	50	41	0.937909	1	26	31	1.10851	1
27	chr18	31324235	31324235	G	T	exonic	ASXL3		nonsynonymous SNV	ASXL3:NM_030632:exon12:c.G4423T:p.V1	NOVEL		102	110	1.13748	1	90	121	1.19377	1	156	181	1.09471	1
27	chr2	166740499	166740499	C	T	exonic	TTC21B		nonsynonymous SNV	TTC21B:NM_024753:exon26:c.G3489A:p.N	NOVEL		149	91	0.831225	1	109	102	1.00632	1	104	118	1.08338	1
27	chr8	145583861	145583861	G	A	exonic	SLC52A2		nonsynonymous SNV	SLC52A2:NM_001253815:exon3:c.G709A:p	NOVEL		87	113	1.23862	1	132	149	1.10382	1	214	242	1.08169	1
27	chr8	41525901	41525901	G	A	exonic	ANK1		nonsynonymous SNV	ANK1:NM_000037:exon39:c.C5278T:p.H17	NOVEL		252	0	0	1	217	242	1.09754	1	282	310	1.06731	1
27	chr1	175066662	175066662	C	A	exonic	TNN		synonymous SNV	TNN:NM_022093:exon8:c.C1698A:p.T566T	DBSNP		313	0	0	1	310	86	0.88643	1	245	87	1.05821	1
27	chr20	62839754	62839754	C	A	exonic	MYT1		nonsynonymous SNV	MYT1:NM_004535:exon7:c.C1205A:p.P402	NOVEL		174	0	0	1	241	109	0.95973	1	160	85	1.05408	1
27	chr9	100092719	100092719	G	A	exonic	CCDC180		synonymous SNV	CCDC180:NM_020893:exon18:c.G2076A:p.	NOVEL		193	87	0.681161	1	187	175	1.00635	1	91	96	1.04636	1
27	chr16	21145698	21145698	C	G	exonic	DNAH3		nonsynonymous SNV	DNAH3:NM_017539:exon7:c.G964C:p.V32	NOVEL		129	121	1.06104	1	152	141	1.00177	1	170	179	1.04539	1
27	chr22	46782327	46782327	C	T	exonic	CELSR1		synonymous SNV	CELSR1:NM_014246:exon19:c.G6711A:p.R	DBSNP		85	0	0	1	43	60	1.21264	1	98	103	1.04446	1
27	chr11	120200987	120200987	G	A	exonic	TLCD5		synonymous SNV	TLCD5:NM_001198675:exon2:c.G198A:p.L	NOVEL		156	0	0	1	73	72	1.03367	1	86	90	1.04227	1
27	chr2	25043645	25043645	G	A	exonic	ADCY3		nonsynonymous SNV	ADCY3:NM_001320613:exon21:c.C3203T:p	COSMIC	ID=COSM242851	180	182	1.10218	1	263	220	0.948187	1	312	326	1.04147	1
27	chr1	56977724	56977724	C	T	exonic	PLPP3		nonsynonymous SNV	PLPP3:NM_003713:exon5:c.G734A:p.R245	COSMIC	ID=COSM727980	136	8	0.121791	1	0	111	1.0817	1	1	105	1.02843	1
27	chr22	17449262	17449262	T	G	exonic	GAB4		nonsynonymous SNV	GAB4:NM_001366857:exon3:c.A490C:p.K1	NOVEL		164	1	0.0132863	1	102	99	1.02532	1	149	148	1.01568	1
27	chr5	137593617	137593617	C	A	exonic	GFRA3		nonsynonymous SNV	GFRA3:NM_001496:exon4:c.G496T:p.A16	NOVEL		132	108	0.986509	1	138	131	1.01377	1	187	183	1.00809	1
27	chr11	609091	609091	G	A	exonic	PHRF1		nonsynonymous SNV	PHRF1:NM_001286581:exon14:c.G3635A:	NOVEL		138	0	0	1	95	86	0.989096	1	79	77	1.00605	1
27	chr17	80615867	80615867	G	T	exonic	RAB40B		nonsynonymous SNV	RAB40B:NM_006822:exon6:c.C709A:p.H2	NOVEL		96	0	0	1	0	130	1.0817	1	4	125	1.00603	1
27	chr12	25398284	25398284	C	G	exonic	KRAS		nonsynonymous SNV	KRAS:NM_001369786:exon2:c.G35C:p.G12	DBSNP+COSMIC	ID=COSM114013	107	107	1.09612	1	1	157	1.03426	2	3	215	1.00509	2
27	chr19	2733433	2733433	G	A	exonic	SLC39A3		synonymous SNV	SLC39A3:NM_144564:exon3:c.C261T:p.A87	DBSNP+COSMIC	ID=COSM400064	79	68	1.0141	1	89	86	1.02301	1	92	89	1.00222	1
27	chr16	23690330	23690330	C	A	exonic	PLK1		nonsynonymous SNV	PLK1:NM_005030:exon1:c.C77A:p.A26E	NOVEL		35	44	1.221	1	55	57	1.05944	1	23	22	0.996465	1
27	chr8	39495067	39495067	G	C	exonic	ADAM18		nonsynonymous SNV	ADAM18:NM_001320313:exon8:c.G600C:p	NOVEL		222	0	0	1	109	107	1.03121	1	92	88	0.996465	1
27	chr6	51890330	51890330	C	T	exonic	PKHD1		synonymous SNV	PKHD1:NM_138694:exon32:c.G4278A:p.S1	DBSNP		205	7	0.0723853	1	108	110	1.0504	1	120	114	0.99298	1
27	chr3	183273272	183273272	A	G	exonic	KLHL6		nonsynonymous SNV	KLHL6:NM_130446:exon1:c.T170C:p.I57T	DBSNP		110	116	1.12522	1	108	99	0.995596	1	161	151	0.986448	1
27	chr7	150095108	150095108	G	C	exonic	ZNF775		nonsynonymous SNV	ZNF775:NM_173680:exon3:c.G1539C:p.Q5	NOVEL		199	16	0.163144	1	260	135	1.05324	1	405	193	0.980564	1
27	chr7	136938294	136938294	C	A	exonic	PTN		nonsynonymous SNV	PTN:NM_001321386:exon3:c.G206T:p.G69	COSMIC	ID=COSM123790	61	50	0.987496	1	62	144	1.0771	2	64	116	0.978983	2
27	chr3	52548821	52548821	G	T	exonic	STAB1		synonymous SNV	STAB1:NM_015136:exon35:c.G3783T:p.L1	NOVEL		69	6	0.175379	1	58	55	1.01322	1	79	73	0.978883	1
27	chr5	124080100	124080100	T	A	exonic	ZNF608		nonsynonymous SNV	ZNF608:NM_020747:exon1:c.A583T:p.S19	NOVEL		365	0	0	1	190	187	1.03257	1	197	182	0.978777	1
27	chr1	205628562	205628562	T	A	exonic	SLC45A3		nonsynonymous SNV	SLC45A3:NM_033102:exon5:c.A1462T:p.I4	NOVEL		272	0	0	1	282	83	0.928168	1	320	102	0.976063	1
27	chr11	5664562	5664562	G	T	exonic	TRIM34;TRIM6-TR		nonsynonymous SNV	TRIM34:NM_001003827:exon8:c.G1090T:	NOVEL		254	0	0	1	135	123	0.992439	1	111	102	0.97605	1
27	chr22	24898128	24898128	T	C	exonic	UPB1		nonsynonymous SNV	UPB1:NM_016327:exon3:c.T311C:p.V104A	NOVEL		436	2	0.0100102	1	246	228	1.00132	1	197	181	0.975975	1
27	chr4	111553563	111553563	A	G	exonic	PITX2		synonymous SNV	PITX2:NM_001204398:exon3:c.T120C:p.S4	NOVEL		135	8	0.122643	1	85	74	0.968842	1	110	101	0.975642	1
27	chr15	59808870	59808870	G	A	exonic	FAM81A		synonymous SNV	FAM81A:NM_152450:exon8:c.G813A:p.K2	NOVEL		103	85	0.991173	1	86	103	1.13447	1	61	56	0.97556	1
27	chr19	35941589	35941589	T	G	exonic	FFAR2		nonsynonymous SNV	FFAR2:NM_001370087:exon2:c.T973G:p.S	NOVEL		99	51	0.745362	1	81	72	0.979624	1	125	114	0.972207	1
27	chr8	59059054	59059054	G	A	exonic	FAM110B		nonsynonymous SNV	FAM110B:NM_147189:exon5:c.G265A:p.A	NOVEL		186	19	0.203183	1	134	220	0.957591	2	187	328	0.967512	2
27	chr2	237172953	237172953	G	T	exonic	ASB18		synonymous SNV	ASB18:NM_212556:exon1:c.C36A:p.L12L	NOVEL		143	0	0	1	72	84	1.12092	1	81	73	0.966171	1
27	chr19	45207415	45207415	C	T	exonic	CEACAM16		synonymous SNV	CEACAM16:NM_001039213:exon4:c.C510T	NOVEL		25	35	1.27881	1	45	34	0.895922	1	50	45	0.965474	1
27	chr10	25140366	25140366	A	G	exonic	PRTFDC1		nonsynonymous SNV	PRTFDC1:NM_020200:exon8:c.T581C:p.F1	DBSNP+COSMIC	ID=COSM534122	177	80	0.68241	1	123	132	1.07759	1	69	62	0.964655	1
27	chr6	162864477	162864477	A	T	exonic	PRKN		synonymous SNV	PRKN:NM_004562:exon2:c.T36A:p.G12G,F	NOVEL		35	17	0.642226	1	46	39	0.955134	1	59	53	0.964516	1
27	chr19	14675054	14675054	G	T	exonic	TECR		synonymous SNV	TECR:NM_138501:exon7:c.G444T:p.V148V	NOVEL		59	56	1.06753	1	106	96	0.989323	1	116	104	0.963524	1
27	chr1	11210282	11210282	C	T	exonic	MTOR		nonsynonymous SNV	MTOR:NM_004958:exon31:c.G4471A:p.G1	NOVEL		87	64	0.929162	1	88	70	0.922273	1	78	69	0.956717	1
27	chr15	65345339	65345339	C	T	exonic	SLC51B		nonsynonymous SNV	SLC51B:NM_178859:exon4:c.C224T:p.P75L	DBSNP		121	119	1.08699	1	112	123	1.08957	1	144	126	0.951171	1
27	chr7	44721422	44721422	C	T	exonic	OGDH		nonsynonymous SNV	OGDH:NM_001165036:exon10:c.C1298T:p	NOVEL		285	1	0.00766518	1	341	138	0.887839	1	297	135	0.949445	1
27	chr5	434588	434588	C	G	exonic	AHRR		nonsynonymous SNV	AHRR:NM_001242412:exon11:c.C1745G:p	NOVEL		54	41	0.946125	1	87	80	0.997222	1	108	94	0.94848	1
27	chr6	393328	393328	A	G	exonic	IRF4		nonsynonymous SNV	IRF4:NM_001195286:exon2:c.A176G:p.K5	COSMIC	ID=COSM571010	160	0	0	1	81	67	0.942392	1	133	115	0.945144	1
27	chr16	11000786	11000786	G	A	exonic	CIITA		synonymous SNV	CIITA:NM_000246:exon11:c.G1437A:p.A47	DBSNP+COSMIC	ID=COSM395710	391	0	0	1	249	206	0.942485	1	276	237	0.941635	1
27	chr8	95444645	95444645	G	A	exonic	FSBP		nonsynonymous SNV	FSBP:NM_001256141:exon2:c.C614T:p.S2	DBSNP		257	0	0	1	272	129	0.99137	1	270	121	0.940217	1
27	chr1	155920135	155920135	C	T	exonic	ARHGEF2		nonsynonymous SNV	ARHGEF2:NM_001162383:exon21:c.G284	NOVEL		115	22	0.673207	1	126	45	1.07413	1	101	30	0.924784	1
27	chr16	4924409	4924409	C	A	exonic	UBN1		synonymous SNV	UBN1:NM_001079514:exon15:c.C1998A:p	NOVEL		206	0	0	1	131	153	1.12148	1	99	82	0.923394	1
27	chr7	158716300	158716300	A	T	exonic	WDR60		nonsynonymous SNV	WDR60:NM_001350915:exon16:c.A1560T	NOVEL		267	1	0.00818	1	311	164	1.064	1	335	146	0.922205	1
27	chr5	39134983	39134983	C	T	exonic	FYB1		nonsynonymous SNV	FYB1:NM_001243093:exon8:c.G1679C:p.V	NOVEL		415	34	0.166005	1	200	183	0.994651	1	269	222	0.921559	1
27	chr1	2450620	2450620	T	A	exonic	PANK4		nonsynonymous SNV	PANK4:NM_018216:exon7:c.A997T:p.M33	NOVEL		126	8	0.13088	1	87	77	0.977384	1	118	97	0.91957	1
27	chr5	10461422	10461422	G	A	exonic	ROPN1L		nonsynonymous SNV	ROPN1L:NM_031916:exon4:c.G544A:p.V1	NOVEL		228	208	1.04584	1	232	208	0.984077	1	329	267	0.913097	1
27	chr19	41128426	41128426	G	A	exonic	LTBP4		unknown	UNKNOWN	DBSNP+COSMIC	ID=COSM638164	94	0	0	1	64	55	0.962131	1	104	84	0.910695	1
27	chr8	139890026	139890026	T	G	exonic	COL22A1		nonsynonymous SNV	COL22A1:NM_152888:exon3:c.A625C:p.I20	NOVEL		47	36	0.950852	1	63	54	0.960785	1	67	54	0.90962	1
27	chr2	26688572	26688572	G	A	exonic	OTOF		synonymous SNV	OTOF:NM_194322:exon20:c.C2697T:p.R89	DBSNP+COSMIC	ID=COSM118015	166	14	0.170508	1	115	96	0.947125	1	120	95	0.90061	1
27	chr11	100849779	100849779	C	T	exonic	ARHGAP42		nonsynonymous SNV	ARHGAP42:NM_152432:exon22:c.C2455T:	DBSNP+COSMIC	ID=COSM735626	260	205	0.966472	1	246	183	0.887998	1	193	152	0.898	1
27	chr7	18066688	18066688	C	T	exonic	PRPS1L1		nonsynonymous SNV	PRPS1L1:NM_175886:exon1:c.G718A:p.G2	NOVEL		285	132	0.693947	1	374	170	0.963032	1	291	119	0.881826	1
27	chr2	1271181	1271181	G	C	exonic	SNTG2		synonymous SNV	SNTG2:NM_018968:exon14:c.G1122C:p.L3	NOVEL		65	7	0.213135	1	42	48	1.11024	1	63	48	0.881394	1
27	chr9	137706686	137706686	C	A	exonic	COL5A1		stopgain	COL5A1:NM_000093:exon50:c.C3950A:p.S	NOVEL		107	13	0.237493	1	66	84	1.16575	1	101	76	0.875169	1
27	chr9	77353448	77353448	C	G	exonic	TRPM6		nonsynonymous SNV	TRPM6:NM_001177310:exon36:c.G5636C	NOVEL		79	28	0.573671	1	66	40	0.785548	1	81	58	0.850482	1
27	chr9	120053664	120053664	G	T	exonic	ASTN2		nonsynonymous SNV	ASTN2:NM_001365068:exon2:c.C571A:p.L	NOVEL		79	5	0.130491	1	56	65	1.11827	1	63	45	0.84926	1
27	chr6	105572441	105572441	T	C	exonic	BVES		nonsynonymous SNV	BVES:NM_001199563:exon5:c.A629G:p.H2	NOVEL		123	0	0	1	70	49	0.857171	1	54	38	0.841875	1
27	chr1	178875972	178875972	G	C	exonic	RALGPS2		nonsynonymous SNV	RALGPS2:NM_001286247:exon18:c.G1614	NOVEL		311	0	0	1	232	68	0.925186	1	200	52	0.833284	1
27	chr7	44444044	44444044	C	T	exonic	NUDCD3		nonsynonymous SNV	NUDCD3:NM_015332:exon4:c.G781A:p.V2	DBSNP+COSMIC	ID=COSM326271	226	0	0	1	238	109	0.968027	1	307	116	0.833177	1
27	chr1	149871828	149871828	G	A	exonic	BOLA1		synonymous SNV	BOLA1:NM_001321025:exon2:c.G216A:p.\	NOVEL		168	52	0.990893	1	201	63	0.974042	1	189	47	0.804222	1
27	chr7	14378297	14378297	C	T	exonic	DGKB		synonymous SNV	DGKB:NM_001350720:exon21:c.G1911A:p	NOVEL		200	96	0.710997	1	215	119	1.09797	1	198	71	0.80191	1
27	chr6	36106749	36106749	G	A	exonic	MAPK13		nonsynonymous SNV	MAPK13:NM_002754:exon11:c.G935A:p.R	DBSNP		179	0	0	1	175	48	0.448079	1	168	74	0.623258	1
27	chr10	75520478	75520478	C	T	exonic	SEC24C		synonymous SNV	SEC24C:NM_198597:exon6:c.C858T:p.F286	DBSNP		86	0	0	1	64	18	0.456959	1	68	29	0.609366	1
27	chr15	43720284	43720284	G	A	exonic	TP53BP1		nonsynonymous SNV	TP53BP1:NM_001141979:exon18:c.C3758	NOVEL		306	0	0	1	305	48	0.283064	1	261	84	0.496263	1
27	chr5	122425723	122425723	G	T	exonic	PRDM6		nonsynonymous SNV	PRDM6:NM_001136239:exon2:c.G14T:p.G	NOVEL		106	0	0	1	80	23	0.464846	1	181	39	0.361321	1
27	chr5	122425722	122425722	G	T	exonic	PRDM6		stopgain	PRDM6:NM_001136239:exon2:c.G13T:p.G	NOVEL		106	0	0	1	79	22	0.45344	1	181	39	0.361321	1
27	chr19	44008063	44008063	C	G	exonic	PHLDB3		nonsynonymous SNV	PHLDB3:NM_198850:exon2:c.G208C:p.D70	NOVEL		74	0	0	1	82	2	0.0495643	1	134	10	0.141543	1
27	chr2	21229734	21229734	T	G	exonic	APOB		nonsynonymous SNV	APOB:NM_000384:exon26:c.A10006C:p.N	NOVEL		408	0	0	1	379	27	0.138438	1	260	8	0.0608425	1
27	chr8	144999924	144999924	G	T	exonic	PLEC		synonymous SNV	PLEC:NM_201378:exon31:c.G4131A:p.K13	NOVEL		69	0	0	1	75	11	0.266264	1	166	5	0.0595972	1
27	chr5	82815376	82815376	T	C	exonic	VCAN		synonymous SNV	VCAN:NM_001164098:exon7:c.T1251C:p.D	DBSNP		383	50	0.253146	1	403	4	0.020459	1	430	1	0.00472906	1
27	chr4	148887939	148887939	C	A	exonic	ARHGAP10		nonsynonymous SNV	ARHGAP10:NM_024605:exon18:c.C1665A	NOVEL		229	35	0.290638	1	266	0	0	1	239	0	0	1
27	chr18	28662379	28662379	G	A	exonic	DSC2		nonsynonymous SNV	DSC2:NM_004949:exon9:c.C1088T:p.S363	NOVEL		107	12	0.221066	1	106	0	0	1	94	0	0	1
27	chr9	139909151	139909151	T	A	exonic	ABCA2		nonsynonymous SNV	ABCA2:NM_001606:exon26:c.A3994T:p.S1	NOVEL		98	10	0.202985	1	134	0	0	1	348	0	0	1
27	chr5	156479411	156479411	C	A	exonic	HAVCR1		nonsynonymous SNV	HAVCR1:NM_012206:exon3:c.G634T:p.V2	NOVEL		336	31	0.185176	1	359	0	0	1	343	0	0	1
27	chr2	73184204	73184204	C	T	exonic	SFXN5		nonsynonymous SNV	SFXN5:NM_001330400:exon14:c.G974A:p	NOVEL		248	22	0.178627	1	325	0	0	1	451	0	0	1
27	chr1	196711025	196711025	C	T	exonic	CFH		nonsynonymous SNV	CFH:NM_000186:exon19:c.C2977T:p.P993	NOVEL		502	22	0.17601	1	411	0	0	1	407	0	0	1
27	chr16	4247824	4247824	T	A	exonic	SRL		nonsynonymous SNV	SRL:NM_001098814:exon4:c.A352T:p.N11	NOVEL		120	10	0.168634	1	150	0	0	1	144	0	0	1
27	chr17	26883865	26883865	G	A	exonic	PIGS		nonsynonymous SNV	PIGS:NM_033198:exon9:c.C1060T:p.P354	DBSNP		52	4	0.156589	1	91	0	0	1	76	0	0	1
27	chr11	71726934	71726934	G	A	exonic	NUMA1		nonsynonymous SNV	NUMA1:NM_006185:exon15:c.C1615T:p.L	NOVEL		213	8	0.0793571	1	275	0	0	1	272	0	0	1
28	chrX	16850816	16850816	C	A	exonic	TXLNG		nonsynonymous SNV	TXLNG:NM_001168683:exon4:c.C539A:p.T	NOVEL		7	105	NA	1	10	73	NA	1				
28	chrX	30260363	30260363	G	A	exonic	MAGEB4		synonymous SNV	MAGEB4:NM_002367:exon1:c.G111A:p.E3	NOVEL		153	8	NA	1	142	0	NA	1				
28	chrX	108673608	108673608	C	T	exonic	GUCY2F		synonymous SNV	GUCY2F:NM_001522:exon8:c.G1719A:p.L5	NOVEL		12	209	NA	1	24	135	NA	1				
28	chrX	138644071	138644071	A	G	exonic	F9		synonymous SNV	F9:NM_001313913:exon7:c.A1113G:p.G37	NOVEL		5	123	NA	1	18	67	NA	1				
28	chrX	16657341	16657341	G	C	exonic	CTPS2		nonsynonymous SNV	CTPS2:NM_001144002:exon14:c.C1322G:p	NOVEL		165	0	NA	1	115	11	NA	1				
28	chrY	14837072	14837072	G	C	exonic	USP9Y		nonsynonymous SNV	USP9Y:NM_004654:exon6:c.C352G:p.Q118	NOVEL		72	0	NA	1	11	34	NA	1				
28	chr7	1518500	1518500	C	T	exonic	INTS1		nonsynonymous SNV	INTS1:NM_001080453:exon32:c.G4351A:p	DBSNP		128	111	0.934129	2	150	57	1.48763	1				
28	chr3	48698127	48698127	G	A	exonic	CELSR3		synonymous SNV	CELSR3:NM_001407:exon1:c.C1941T:p.H64	NOVEL		428	0	0	1	115	172	1.43979	1				
28	chr1	150936122	150936122	C	A	exonic	SETDB1		nonsynonymous SNV	SETDB1:NM_001145415:exon20:c.C3574A	DBSNP		241	0	0	1	172	59	1.41621	1				
28	chr21	43256739	43256739	T	C	exonic	PRDM15		nonsynonymous SNV	PRDM15:NM_001040424:exon9:c.A1132G	NOVEL		98	99	0.927077	2	120	71	1.37875	1				
28	chr3	38287649	38287649	G	T	exonic	OXSR1		nonsynonymous SNV	OXSR1:NM_005109:exon13:c.G1194T:p.Q	NOVEL		309	0	0	1	101	126	1.33351	1				
28	chr3	52824782	52824782	A	G	exonic	ITIH1		nonsynonymous SNV	ITIH1:NM_001166435:exon16:c.A1475G:p	NOVEL		390	0	0	1	102	120	1.29862	1				
28	chr3	130712874	130712874	G	T	exonic	ATP2C1		nonsynonymous SNV	ATP2C1:NM_001199182:exon20:c.G1974T	NOVEL		142	0	0	1	73	45	1.29754	1				
28	chr17	33353396	33353396	C	A	exonic	RFFL		nonsynonymous SNV	RFFL:NM_001017368:exon2:c.G177T:p.R5	NOVEL		58	0	0	1	28	16	1.23725	1				
28	chr13	73366607	73366607	T	G	exonic	PIBF1		nonsynonymous SNV	PIBF1:NM_001349655:exon3:c.T275G:p.L9	NOVEL		61	59	1.04765	1	44	46	1.22791	1				
28	chr7	22196418	22196418	C	A	exonic	RAPGEF5		nonsynonymous SNV	RAPGEF5:NM_001367603:exon6:c.G737T:	NOVEL		200	0	0	1	158	44	1.17677	1				
28	chr2	25967221	25967221	G	T	exonic	ASXL2		nonsynonymous SNV	ASXL2:NM_001369347:exon10:c.C1205A:p	NOVEL		502	0	0	1	269	162	1.17419	1				
28	chr7	113518709	113518709	C	A	exonic	PPP1R3A		nonsynonymous SNV	PPP1R3A:NM_002711:exon4:c.G2438T:p.R	NOVEL		888	2	0.00903965	1	474	172	1.17217	1				
28	chr8	28331302	28331302	A	C	exonic	FBXO16		nonsynonymous SNV	FBXO16:NM_172366:exon3:c.T122G:p.L41	NOVEL		52	0	0	1	44	41	1.15882	1				
28	chr21	43256603	43256603	T	C	exonic	PRDM15		nonsynonymous SNV	PRDM15:NM_001040424:exon9:c.A1268G	NOVEL		42	38	0.876275	2	40	18	1.15108	1				
28	chr6	88853848	88853848	G	A	exonic	CNR1		synonymous SNV	CNR1:NM_001160259:exon2:c.C1146T:p.C	NOVEL		341	0	0	1	251	128	1.14911	1				
28	chr7	8790761	8790761	T	C	exonic	NXPH1		nonsynonymous SNV	NXPH1:NM_152745:exon3:c.T178C:p.S60P	NOVEL		780	0	0	1	616	165	1.14136	1				
28	chr1	109740249	109740249	C	T	exonic	KIAA1324		nonsynonymous SNV	KIAA1324:NM_001284353:exon9:c.C1261T	NOVEL		55	1	0.020193	1	21	81	1.1137	1				
28	chr19	36351271	36351271	A	G	exonic	KIRREL2		nonsynonymous SNV	KIRREL2:NM_001329530:exon5:c.A596G:p	NOVEL		196	0	0	1	133	64	1.10536	1				
28	chr16	66527125	66527125	C	A	exonic	BEAN1		nonsynonymous SNV	BEAN1:NM_001197225:exon5:c.C408A:p.H	NOVEL		260	0	0	1	107	91	1.10415	1				
28	chr5	176958298	176958298	G	T	exonic	FAM193B		nonsynonymous SNV	FAM193B:NM_001366500:exon6:c.C1161A	NOVEL		267	0	0	1	114	96	1.09826	1				
28	chr4	146823450	146823450	C	T	exonic	ZNF827		nonsynonymous SNV	ZNF827:NM_001306215:exon2:c.G961A:p	NOVEL		44	39	1.00122	1	30	25	1.09202	1				
28	chr17	40117310	40117310	G	T	exonic	TTC25		unknown	UNKNOWN	DBSNP		524	0	0	1	240	113	1.08917	1				
28	chr15	73045163	73045163	C	A	exonic	ADPGK		nonsynonymous SNV	ADPGK:NM_001365227:exon4:c.G596T:p.(	NOVEL		248	80	0.981133	1	218	76	1.08633	1				
28	chr8	69002816	69002816	A	C	exonic	PREX2		nonsynonymous SNV	PREX2:NM_024870:exon20:c.A2116C:p.T7	NOVEL		150	0	0	1	67	55	1.08307	1				
28	chr6	165703535	165703535	A	C	exonic	C6orf118		nonsynonymous SNV	C6orf118:NM_144980:exon7:c.T1142G:p.I	NOVEL		46	140	1.17826	2	99	48	1.08281	1				
28	chr3	39307579	39307579	C	T	exonic	CX3CR1		nonsynonymous SNV	CX3CR1:NM_001171171:exon2:c.G422A:p.	DBSNP		79	183	1.09302	2	17	152	1.08039	2				
28	chr17	38510653	38510653	G	A	exonic	RARA		nonsynonymous SNV	RARA:NM_001145302:exon5:c.G616A:p.G	COSMIC	ID=COSM406617	148	70	1.00496	1	83	142	1.07366	2				
28	chr13	28239924	28239924	G	A	exonic	POLR1D		nonsynonymous SNV	POLR1D:NM_001206559:exon3:c.G119A:p.	NOVEL		333	0	0	1	140	110	1.05707	1				
28	chr11	124310174	124310174	G	T	exonic	OR8B8		nonsynonymous SNV	OR8B8:NM_012378:exon1:c.C808A:p.Q27	NOVEL		468	0	0	1	274	123	1.05416	1				
28	chr18	72775336	72775336	G	A	exonic	ZNF407		nonsynonymous SNV	ZNF407:NM_017757:exon8:c.G5659A:p.A	DBSNP		64	130	1.04862	2	58	94	1.05207	2				
28	chr3	184290878	184290878	A	C	exonic	EPHB3		nonsynonymous SNV	EPHB3:NM_004443:exon3:c.A770C:p.N257	NOVEL		192	0	0	1	123	55	1.05132	1				
28	chr4	108622376	108622376	C	G	exonic	PAPSS1		nonsynonymous SNV	PAPSS1:NM_005443:exon2:c.G126C:p.Q42	NOVEL		318	0	0	1	159	123	1.04787	1				
28	chr19	46733649	46733649	C	T	exonic	IGFL1		synonymous SNV	IGFL1:NM_198541:exon3:c.C198T:p.T66T	NOVEL		345	128	0.846946	1	156	249	1.04594	2				
28	chr19	58384615	58384615	G	A	exonic	ZNF814		nonsynonymous SNV	ZNF814:NM_001144989:exon3:c.C2143T:p	DBSNP+COSMIC	ID=COSM100251	509	0	0	1	320	142	1.04577	1				
28	chr12	54925071	54925071	G	T	exonic	NCKAP1L		nonsynonymous SNV	NCKAP1L:NM_001184976:exon23:c.G2384	NOVEL		196	0	0	1	122	54	1.04393	1				
28	chr18	64239414	64239414	T	A	exonic	CDH19		nonsynonymous SNV	CDH19:NM_001271028:exon2:c.A28T:p.M	NOVEL		227	118	1.07046	1	231	102	1.04219	1				
28	chr5	90149331	90149331	T	A	exonic	ADGRV1		nonsynonymous SNV	ADGRV1:NM_032119:exon80:c.T17435A:p	NOVEL		183	0	0	1	98	75	1.04152	1				
28	chr1	53727746	53727746	C	T	exonic	LRP8		synonymous SNV	LRP8:NM_033300:exon10:c.G1398A:p.V46	NOVEL		102	0	0	1	68	52	1.04106	1				
28	chr9	135985057	135985057	G	C	exonic	RALGDS		synonymous SNV	RALGDS:NM_001042368:exon4:c.C375G:p	NOVEL		212	0	0	1	102	78	1.04106	1				
28	chr5	180651203	180651203	A	T	exonic	TRIM41		synonymous SNV	TRIM41:NM_033549:exon1:c.A204T:p.G68	NOVEL		137	0	0	1	55	42	1.04023	1				
28	chr4	186573873	186573873	G	T	exonic	SORBS2		synonymous SNV	SORBS2:NM_001145674:exon2:c.C34A:p.R	NOVEL		191	135	0.882391	1	142	108	1.03785	1				
28	chr16	4242416	4242416	T	C	exonic	SRL		nonsynonymous SNV	SRL:NM_001098814:exon6:c.A1160G:p.K3	NOVEL		298	0	0	1	206	90	1.03453	1				
28	chr14	95670707	95670707	C	T	exonic	CLMN		nonsynonymous SNV	CLMN:NM_024734:exon9:c.G979A:p.V327	DBSNP		166	122	0.902635	1	143	108	1.03372	1				
28	chr15	42979940	42979940	T	A	exonic	STARD9		nonsynonymous SNV	STARD9:NM_020759:exon23:c.T6164A:p.N	NOVEL		360	333	0.966479	2	270	261	1.03279	2				
28	chr16	2854494	2854494	T	C	exonic	PRSS41		nonsynonymous SNV	PRSS41:NM_001354586:exon6:c.T733C:p.\	DBSNP+COSMIC	ID=COSN267316	81	0	0	1	60	26	1.02864	1				
28	chr3	183957490	183957490	C	T	exonic	VWA5B2		synonymous SNV	VWA5B2:NM_001320373:exon13:c.C1752	NOVEL		216	0	0	1	80	122	1.02747	2				
28	chr3	49755391	49755391	G	A	exonic	AMIGO3		nonsynonymous SNV	AMIGO3:NM_198722:exon1:c.C1508T:p.T5	NOVEL		60	101	0.981685	2	13	76	1.02576	2				
28	chr19	38077569	38077569	G	A	exonic	ZNF571		synonymous SNV	ZNF571:NM_001321272:exon2:c.C9T:p.H3	DBSNP		444	0	0	1	227	97	1.01863	1				
28	chr10	35928681	35928681	C	T	exonic	FZD8		synonymous SNV	FZD8:NM_031866:exon1:c.G1677A:p.P559	COSMIC	ID=COSM666820	162	0	0	1	99	72	1.01155	1				
28	chr3	16419414	16419414	C	T	exonic	RFTN1		nonsynonymous SNV	RFTN1:NM_015150:exon5:c.G637A:p.A21	DBSNP		170	288	0.98402	2	45	238	1.01021	2				
28	chr2	191895777	191895777	G	A	exonic	STAT4		nonsynonymous SNV	STAT4:NM_001243835:exon23:c.C2141T:p	NOVEL		565	259	0.983738	1	456	192	1.00813	1				
28	chr10	32101648	32101648	T	G	exonic	ARHGAP12		nonsynonymous SNV	ARHGAP12:NM_001270699:exon11:c.A17	NOVEL		110	90	0.958865	1	90	65	1.00747	1				
28	chr19	1360475	1360475	C	A	exonic	PWWP3A		nonsynonymous SNV	PWWP3A:NM_001369792:exon4:c.C351A:	NOVEL		395	1	0.00790336	1	237	199	1.00469	2				
28	chr11	121008473	121008473	C	T	exonic	TECTA		synonymous SNV	TECTA:NM_005422:exon10:c.C3285T:p.R1	NOVEL		362	0	0	1	213	89	1.00271	1				
28	chr2	196636453	196636453	C	T	exonic	DNAH7		synonymous SNV	DNAH7:NM_018897:exon61:c.G11364A:p.	NOVEL		284	445	0.955233	2	180	258	1.00209	2				
28	chr6	39196856	39196856	G	A	exonic	KCNK5		nonsynonymous SNV	KCNK5:NM_003740:exon1:c.C32T:p.A11V	NOVEL		104	44	0.93046	1	89	37	0.999129	1				
28	chr22	24437598	24437598	C	T	exonic	CABIN1		synonymous SNV	CABIN1:NM_001199281:exon5:c.C222T:p.	DBSNP+COSMIC	ID=COSM458225	102	0	0	1	90	64	0.998416	1				
28	chr16	84229247	84229247	C	T	exonic	ADAD2		synonymous SNV	ADAD2:NM_001145400:exon6:c.C996T:p.R	DBSNP+COSMIC	ID=COSM412943	109	139	1.19428	1	37	180	0.996403	2				
28	chr9	5456120	5456120	A	G	exonic	CD274		nonsynonymous SNV	CD274:NM_001267706:exon2:c.A7G:p.I3V	NOVEL		107	101	1.03467	1	92	65	0.994641	1				
28	chr19	49001750	49001750	G	A	exonic	LMTK3		nonsynonymous SNV	LMTK3:NM_001080434:exon12:c.C2663T:p	NOVEL		517	0	0	1	356	147	0.994351	1				
28	chr11	67187050	67187050	G	T	exonic	CARNS1		synonymous SNV	CARNS1:NM_020811:exon5:c.G576T:p.V19	NOVEL		162	0	0	1	102	42	0.992378	1				
28	chr13	114784320	114784320	C	G	exonic	RASA3		synonymous SNV	RASA3:NM_001320822:exon10:c.G765C:p.	NOVEL		48	0	0	1	53	37	0.98767	1				
28	chr7	6063249	6063249	C	G	exonic	AIMP2		nonsynonymous SNV	AIMP2:NM_001326607:exon3:c.C683G:p.A	DBSNP		297	0	0	1	218	125	0.98441	2				
28	chr19	35617371	35617371	C	T	exonic	LGI4		nonsynonymous SNV	LGI4:NM_139284:exon8:c.G1102A:p.E368	NOVEL		159	0	0	1	91	37	0.983518	1				
28	chr12	78570927	78570927	A	T	exonic	NAV3		nonsynonymous SNV	NAV3:NM_001024383:exon27:c.A5131T:p	NOVEL		214	99	0.989914	1	185	75	0.981473	1				
28	chr5	52201697	52201697	G	A	exonic	ITGA1		nonsynonymous SNV	ITGA1:NM_181501:exon12:c.G1414A:p.D4	NOVEL		133	101	0.919709	1	184	151	0.97733	2				
28	chr19	44254785	44254785	G	A	exonic	SMG9		nonsynonymous SNV	SMG9:NM_019108:exon2:c.C109T:p.R37W	DBSNP		260	0	0	1	164	66	0.976352	1				
28	chr10	27324295	27324295	T	A	exonic	ANKRD26		nonsynonymous SNV	ANKRD26:NM_001256053:exon24:c.A3081	NOVEL		453	0	0	1	231	158	0.975798	1				
28	chr19	46351212	46351212	C	T	exonic	SYMPK		stopgain	SYMPK:NM_004819:exon7:c.G474A:p.W15	NOVEL		67	101	0.940782	2	117	47	0.975089	1				
28	chr12	48920268	48920268	C	A	exonic	OR8S1		nonsynonymous SNV	OR8S1:NM_001005203:exon1:c.C854A:p.S	DBSNP		297	0	0	1	152	61	0.974408	1				
28	chr19	9089631	9089631	T	A	exonic	MUC16		synonymous SNV	MUC16:NM_024690:exon1:c.A2184T:p.S7	DBSNP		297	0	0	1	97	66	0.972767	1				
28	chr15	30058628	30058628	T	C	exonic	TJP1		nonsynonymous SNV	TJP1:NM_001330239:exon5:c.A430G:p.S14	NOVEL		795	257	0.982718	1	559	167	0.966662	1				
28	chr4	54440039	54440039	C	A	exonic	LNX1		nonsynonymous SNV	LNX1:NM_001126328:exon2:c.G131T:p.C4	NOVEL		138	0	0	1	64	43	0.965466	1				
28	chr5	130828353	130828353	C	T	exonic	RAPGEF6		synonymous SNV	RAPGEF6:NM_001164386:exon14:c.G165	NOVEL		234	0	0	1	155	104	0.964686	1				
28	chr4	25821504	25821504	C	G	exonic	SEL1L3		nonsynonymous SNV	SEL1L3:NM_001297592:exon8:c.G1244C:p	NOVEL		390	0	0	1	188	126	0.964036	1				
28	chr15	85401019	85401019	G	A	exonic	ALPK3		nonsynonymous SNV	ALPK3:NM_020778:exon6:c.G3656A:p.R12	DBSNP+COSMIC	ID=COSM659880	236	182	0.875743	2	217	182	0.958436	2				
28	chr17	36104844	36104844	T	A	exonic	HNF1B		nonsynonymous SNV	HNF1B:NM_000458:exon1:c.A32T:p.E11V,	NOVEL		211	0	0	1	158	61	0.947711	1				
28	chr3	111312561	111312561	C	A	exonic	ZBED2		nonsynonymous SNV	ZBED2:NM_024508:exon2:c.G488T:p.R163	NOVEL		486	0	0	1	302	116	0.944218	1				
28	chr12	25398284	25398284	C	T	exonic	KRAS		nonsynonymous SNV	KRAS:NM_001369786:exon2:c.G35A:p.G1	DBSNP+COSMIC	ID=COSM113536	135	198	0.93046	2	119	148	0.942998	2				
28	chr19	464301	464301	C	T	exonic	ODF3L2		nonsynonymous SNV	ODF3L2:NM_182577:exon4:c.G413A:p.R13	NOVEL		58	0	0	1	43	32	0.939187	2				
28	chr8	110492291	110492291	G	A	exonic	PKHD1L1		nonsynonymous SNV	PKHD1L1:NM_177531:exon55:c.G9250A:p	NOVEL		132	0	0	1	166	45	0.938909	1				
28	chr15	42454625	42454625	C	T	exonic	VPS39		nonsynonymous SNV	VPS39:NM_015289:exon22:c.G2230A:p.G	DBSNP		495	0	0	1	307	87	0.927936	1				
28	chr6	168708179	168708179	G	A	exonic	DACT2		nonsynonymous SNV	DACT2:NM_001286350:exon3:c.C1748T:p.	NOVEL		202	0	0	1	145	56	0.923886	1				
28	chr3	100508327	100508327	C	A	exonic	ABI3BP		nonsynonymous SNV	ABI3BP:NM_001349332:exon24:c.G2000T	NOVEL		443	0	0	1	324	120	0.919578	1				
28	chr12	46315876	46315876	C	A	exonic	SCAF11		nonsynonymous SNV	SCAF11:NM_004719:exon15:c.G4347T:p.K	COSMIC	ID=COSM136170	768	0	0	1	417	154	0.917646	1				
28	chr3	157131688	157131688	A	G	exonic	VEPH1		synonymous SNV	VEPH1:NM_001167911:exon6:c.T888C:p.A	NOVEL		529	0	0	1	306	113	0.917603	1				
28	chr7	8095177	8095177	C	A	exonic	GLCCI1		nonsynonymous SNV	GLCCI1:NM_138426:exon4:c.C811A:p.Q27	NOVEL		392	0	0	1	352	72	0.917395	1				
28	chr7	138946293	138946293	G	C	exonic	UBN2		nonsynonymous SNV	UBN2:NM_173569:exon6:c.G1201C:p.E40	NOVEL		683	0	0	1	503	132	0.915153	1				
28	chr11	78369145	78369145	G	A	exonic	TENM4		synonymous SNV	TENM4:NM_001098816:exon34:c.C8268T:	NOVEL		237	373	0.956876	2	400	146	0.90981	1				
28	chr11	62455831	62455831	G	A	exonic	LRRN4CL		synonymous SNV	LRRN4CL:NM_203422:exon2:c.C150T:p.Y5	DBSNP		252	0	0	1	165	60	0.907317	1				
28	chr18	7231448	7231448	C	A	exonic	LRRC30		nonsynonymous SNV	LRRC30:NM_001105581:exon1:c.C312A:p.	NOVEL		462	0	0	1	270	98	0.906084	1				
28	chr10	118969119	118969119	T	C	exonic	KCNK18		nonsynonymous SNV	KCNK18:NM_181840:exon3:c.T464C:p.L15	DBSNP		328	0	0	1	284	103	0.905559	1				
28	chr20	56140545	56140545	G	A	exonic	PCK1		synonymous SNV	PCK1:NM_002591:exon10:c.G1554A:p.R51	NOVEL		351	0	0	1	202	73	0.903193	1				
28	chr7	24874245	24874245	T	C	exonic	OSBPL3		nonsynonymous SNV	OSBPL3:NM_145322:exon12:c.A1405G:p.I	NOVEL		577	0	0	1	489	98	0.90194	1				
28	chr11	94277788	94277788	T	C	exonic	FUT4		synonymous SNV	FUT4:NM_002033:exon1:c.T489C:p.C163C	NOVEL		129	0	0	1	64	23	0.899496	1				
28	chr1	41578989	41578989	C	T	exonic	SCMH1		synonymous SNV	SCMH1:NM_001172220:exon7:c.G498A:p.	NOVEL		57	41	0.891462	1	59	35	0.894525	1				
28	chr12	131471671	131471671	G	A	exonic	ADGRD1		stopgain	ADGRD1:NM_198827:exon6:c.G522A:p.W	COSMIC	ID=COSM455122	228	0	0	1	99	50	0.891741	1				
28	chr8	11058157	11058157	T	A	exonic	XKR6		nonsynonymous SNV	XKR6:NM_173683:exon1:c.A692T:p.Q231L	NOVEL		87	0	0	1	77	45	0.886146	1				
28	chr3	132153456	132153456	G	T	exonic	DNAJC13		nonsynonymous SNV	DNAJC13:NM_001329126:exon2:c.G62T:p.	NOVEL		276	0	0	1	172	60	0.879941	1				
28	chr15	48733922	48733922	G	T	exonic	FBN1		stopgain	FBN1:NM_000138:exon50:c.C6159A:p.C20	DBSNP		494	135	0.863366	1	414	109	0.87583	1				
28	chr11	33790430	33790430	G	C	exonic	FBXO3		nonsynonymous SNV	FBXO3:NM_012175:exon3:c.C325G:p.P109	NOVEL		309	0	0	1	203	70	0.87242	1				
28	chr1	22852784	22852784	G	A	exonic	ZBTB40		synonymous SNV	ZBTB40:NM_001330398:exon16:c.G3279A	DBSNP		130	0	0	1	93	53	0.872118	1				
28	chr6	26056464	26056464	C	T	exonic	HIST1H1C		nonsynonymous SNV	HIST1H1C:NM_005319:exon1:c.G193A:p.A	DBSNP+COSMIC	ID=COSM565059	142	251	0.999443	2	213	83	0.870153	1				
28	chr19	51830425	51830425	G	A	exonic	IGLON5		synonymous SNV	IGLON5:NM_001101372:exon6:c.G738A:p	COSMIC	ID=COSM469199	447	0	0	1	294	103	0.860346	1				
28	chr9	100894519	100894519	A	T	exonic	CORO2A		nonsynonymous SNV	CORO2A:NM_003389:exon6:c.T713A:p.M2	NOVEL		85	67	0.939239	1	97	54	0.859151	1				
28	chr19	44376887	44376887	T	G	exonic	ZNF404		nonsynonymous SNV	ZNF404:NM_001033719:exon2:c.A1470C:p	NOVEL		94	154	0.97173	2	167	56	0.854424	1				

28	chr10	74103184	74103184	G	A	exonic	DNAJB12		synonymous SNV	DNAJB12:NM_001002762:exon3:c.C397T:p	NOVEL		71	61	0.984693	1	69	38	0.853203	1				
28	chr17	76472782	76472782	T	C	exonic	DNAH17		nonsynonymous SNV	DNAH17:NM_173628:exon52:c.A8026G:p.	NOVEL		276	0	0	1	168	56	0.85061	1				
28	chr18	21453130	21453130	C	A	exonic	LAMA3		nonsynonymous SNV	LAMA3:NM_000227:exon1:c.C122A:p.P41H	NOVEL		671	0	0	1	368	122	0.847138	1				
28	chr14	86087951	86087951	C	T	exonic	FLRT2		synonymous SNV	FLRT2:NM_001346143:exon2:c.C93T:p.S31	NOVEL		225	0	0	1	94	50	0.83418	1				
28	chr8	63948278	63948278	T	C	exonic	GGH		nonsynonymous SNV	GGH:NM_003878:exon2:c.A161G:p.Y54C	NOVEL		213	0	0	1	122	63	0.818128	1				
28	chr4	187629589	187629589	G	C	exonic	FAT1		nonsynonymous SNV	FAT1:NM_005245:exon2:c.C1393G:p.Q465	NOVEL		455	1	0.00467283	1	216	111	0.815507	1				
28	chr19	35231565	35231565	T	G	exonic	ZNF181		nonsynonymous SNV	ZNF181:NM_001029997:exon4:c.T279G:p.	NOVEL		307	1	0.0101615	1	214	67	0.811258	1				
28	chr9	107549221	107549221	G	A	exonic	ABCA1		nonsynonymous SNV	ABCA1:NM_005502:exon47:c.C6241T:p.R2	DBSNP		258	1	0.00822707	1	157	80	0.81095	1				
28	chr15	48062722	48062722	G	T	exonic	SEMA6D		nonsynonymous SNV	SEMA6D:NM_020858:exon17:c.G1776T:p.	NOVEL		285	66	0.756395	1	235	56	0.808704	1				
28	chr2	72359725	72359725	C	G	exonic	CYP26B1		nonsynonymous SNV	CYP26B1:NM_001277742:exon5:c.G945C:p	NOVEL		516	0	0	1	255	88	0.801476	1				
28	chr5	66410046	66410046	A	C	exonic	MAST4		nonsynonymous SNV	MAST4:NM_001297651:exon9:c.A1008C:p	NOVEL		280	0	0	1	312	69	0.797292	1				
28	chr1	118165846	118165846	T	A	exonic	FAM46C		nonsynonymous SNV	FAM46C:NM_017709:exon2:c.T356A:p.F11	NOVEL		110	0	0	1	41	53	0.790737	1				
28	chr7	64452063	64452063	G	A	exonic	ERV3-1		synonymous SNV	ERV3-1:NM_001007253:exon2:c.C1342T:p	NOVEL		296	0	0	1	194	42	0.783485	1				
28	chr1	223971959	223971959	T	A	exonic	TP53BP2		nonsynonymous SNV	TP53BP2:NM_001031685:exon17:c.A3221	DBSNP		465	0	0	1	287	72	0.77052	1				
28	chr5	41927245	41927245	T	C	exonic	FBXO4		nonsynonymous SNV	FBXO4:NM_001297437:exon2:c.T320C:p.L	NOVEL		229	0	0	1	274	59	0.768329	1				
28	chr7	148801808	148801808	C	T	exonic	ZNF425		synonymous SNV	ZNF425:NM_001001661:exon4:c.G1155A:	NOVEL		311	275	0.94388	2	442	93	0.765284	1				
28	chr4	27009178	27009178	C	T	exonic	STIM2		synonymous SNV	STIM2:NM_001169117:exon8:c.C1005T:p.	DBSNP		136	0	0	1	83	38	0.754485	1				
28	chr20	8714018	8714018	G	A	exonic	PLCB1		synonymous SNV	PLCB1:NM_015192:exon19:c.G2022A:p.V6	NOVEL		46	96	1.03796	2	104	33	0.747485	1				
28	chr7	45010550	45010550	G	A	exonic	MYO1G		nonsynonymous SNV	MYO1G:NM_033054:exon8:c.C955T:p.R31	NOVEL		163	0	0	1	181	29	0.746051	1				
28	chr2	230272008	230272008	C	T	exonic	DNER		nonsynonymous SNV	DNER:NM_139072:exon10:c.G1663A:p.G5	COSMIC	ID=COSM576662	141	69	1.02834	1	118	32	0.725854	1				
28	chr16	89264572	89264572	C	A	exonic	SLC22A31		nonsynonymous SNV	SLC22A31:NM_001366322:exon7:c.G826T	NOVEL		45	0	0	1	28	12	0.720732	1				
28	chr19	10742762	10742762	C	T	exonic	SLC44A2		synonymous SNV	SLC44A2:NM_001145056:exon10:c.C747T:	NOVEL		209	0	0	1	150	40	0.716303	1				
28	chr13	43180700	43180700	T	A	exonic	TNFSF11		synonymous SNV	TNFSF11:NM_003701:exon5:c.T600A:p.T2	DBSNP		424	0	0	1	238	100	0.710781	1				
28	chr2	102793056	102793056	C	T	exonic	IL1R1		nonsynonymous SNV	IL1R1:NM_001320983:exon10:c.C1115T:p.	NOVEL		576	0	0	1	296	77	0.70238	1				
28	chr8	95161090	95161090	C	A	exonic	CDH17		nonsynonymous SNV	CDH17:NM_001144663:exon14:c.G1809T:p	NOVEL		16	275	1.00683	2	168	69	0.699444	1				
28	chr7	98792870	98792870	G	T	exonic	KPNA7		nonsynonymous SNV	KPNA7:NM_001145715:exon4:c.C376A:p.L	NOVEL		192	0	0	1	143	27	0.699211	1				
28	chr20	42213523	42213523	C	A	exonic	SGK2		nonsynonymous SNV	SGK2:NM_016276:exon12:c.C1151A:p.P38	NOVEL		283	0	0	1	167	42	0.683744	1				
28	chr14	102675651	102675651	G	C	exonic	WDR20		nonsynonymous SNV	WDR20:NM_001242416:exon2:c.G961C:p.	NOVEL		373	0	0	1	215	83	0.669136	1				
28	chr2	168100958	168100958	G	A	exonic	XIRP2		stopgain	XIRP2:NM_001199144:exon7:c.G2390A:p.W	NOVEL		368	152	0.914844	1	254	61	0.658885	1				
28	chr8	6614708	6614708	A	G	exonic	AGPAT5		synonymous SNV	AGPAT5:NM_018361:exon8:c.A894G:p.S29	NOVEL		43	0	0	1	56	20	0.632221	1				
28	chr19	59056289	59056289	C	T	exonic	TRIM28		synonymous SNV	TRIM28:NM_005762:exon1:c.C165T:p.G55	NOVEL		68	0	NA	1	45	10	0.618625	1				
28	chr18	19019513	19019513	G	A	exonic	GREB1L		synonymous SNV	GREB1L:NM_001142966:exon8:c.G864A:p	NOVEL		300	0	0	1	199	44	0.61608	1				
28	chr5	89781488	89781488	C	T	exonic	POLR3G		nonsynonymous SNV	POLR3G:NM_001370351:exon2:c.C104T:p.	NOVEL		15	107	0.934413	2	102	35	0.613762	1				
28	chr6	56338772	56338772	T	A	exonic	DST		nonsynonymous SNV	DST:NM_015548:exon74:c.A14024T:p.Q46	NOVEL		344	0	0	1	224	46	0.579675	1				
28	chr6	87795539	87795539	C	A	exonic	CGA		nonsynonymous SNV	CGA:NM_000735:exon4:c.G286T:p.G96W,	NOVEL		381	0	0	1	228	46	0.571212	1				
28	chr21	45511920	45511920	A	G	exonic	TRAPPC10		nonsynonymous SNV	TRAPPC10:NM_001351709:exon12:c.A158	NOVEL		261	0	0	1	106	33	0.570363	1				
28	chr8	110471837	110471837	G	C	exonic	PKHD1L1		nonsynonymous SNV	PKHD1L1:NM_177531:exon47:c.G7018C:p.	DBSNP		253	0	0	1	296	43	0.558422	1				
28	chr1	71532477	71532477	C	T	exonic	ZRANB2		nonsynonymous SNV	ZRANB2:NM_005455:exon9:c.G911A:p.R3	NOVEL		117	0	0	1	103	65	0.542611	1				
28	chr1	71531422	71531422	C	A	exonic	ZRANB2		stopgain	ZRANB2:NM_005455:exon10:c.G943T:p.E3	NOVEL		168	1	0.00669119	1	139	86	0.536044	1				
28	chr1	71531418	71531418	T	A	exonic	ZRANB2		nonsynonymous SNV	ZRANB2:NM_005455:exon10:c.A947T:p.N	NOVEL		164	1	0.0068534	1	140	80	0.509978	1				
28	chr15	85230864	85230864	A	T	exonic	SEC11A		synonymous SNV	SEC11A:NM_001271919:exon3:c.T303A:p.	NOVEL		133	0	0	1	82	11	0.497055	1				
28	chr3	51989876	51989876	C	T	exonic	GPR62		synonymous SNV	GPR62:NM_080865:exon1:c.C208T:p.L70L	NOVEL		73	0	0	1	40	10	0.480488	1				
28	chr14	20849189	20849189	G	A	exonic	TEP1		nonsynonymous SNV	TEP1:NM_001319035:exon31:c.C4339T:p.R	DBSNP		112	1	0.0188567	1	90	19	0.418774	1				
28	chr2	27454371	27454371	G	A	exonic	CAD		nonsynonymous SNV	CAD:NM_001306079:exon15:c.G2134A:p.A	NOVEL		187	0	0	1	130	19	0.398354	1				
28	chr2	9017209	9017209	T	G	exonic	MBOAT2		nonsynonymous SNV	MBOAT2:NM_001321266:exon5:c.A242C:p	NOVEL		265	0	0	1	161	22	0.375555	1				
28	chr1	3550029	3550029	T	G	exonic	WRAP73		synonymous SNV	WRAP73:NM_017818:exon9:c.A855C:p.G2	NOVEL		194	1	0.0109272	1	128	23	0.365934	1				
28	chr8	87588054	87588054	A	T	exonic	CNGB3		nonsynonymous SNV	CNGB3:NM_019098:exon18:c.T2408A:p.V8	NOVEL		237	0	0	1	166	22	0.281137	1				
28	chr7	149420012	149420012	G	T	exonic	KRBA1		unknown	UNKNOWN	NOVEL		445	0	0	1	391	26	0.274493	1				
28	chr5	71482496	71482496	A	T	exonic	MAP1B		nonsynonymous SNV	MAP1B:NM_001324255:exon3:c.A47T:p.Q	NOVEL		103	0	0	1	158	10	0.26205	1				
28	chr3	38645257	38645257	T	C	exonic	SCN5A		synonymous SNV	SCN5A:NM_000335:exon12:c.A1836G:p.T6	NOVEL		197	0	0	1	125	15	0.257404	1				
28	chr8	120581482	120581482	A	G	exonic	ENPP2		synonymous SNV	ENPP2:NM_001040092:exon21:c.T2046C:p	NOVEL		298	0	0	1	469	28	0.248025	1				
28	chr9	14120446	14120446	G	A	exonic	NFIB		nonsynonymous SNV	NFIB:NM_001282787:exon7:c.C482T:p.T16	NOVEL		74	0	0	1	45	5	0.240244	1				
28	chr7	83636736	83636736	T	G	exonic	SEMA3A		nonsynonymous SNV	SEMA3A:NM_006080:exon10:c.A1073C:p.	NOVEL		310	0	0	1	247	14	0.236146	1				
28	chr12	56426454	56426454	G	T	exonic	IKZF4		nonsynonymous SNV	IKZF4:NM_001351090:exon5:c.G690T:p.Q	NOVEL		191	0	0	1	175	13	0.235275	1				
28	chr6	41129230	41129230	C	A	exonic	TREM2		synonymous SNV	TREM2:NM_001271821:exon2:c.G162T:p.	DBSNP		167	0	0	1	165	12	0.230674	1				
28	chr22	19195725	19195725	G	A	exonic	CLTCL1		nonsynonymous SNV	CLTCL1:NM_001835:exon22:c.C3539T:p.T1	NOVEL		80	0	0	1	143	15	0.22808	1				
28	chr7	82763659	82763659	A	G	exonic	PCLO		synonymous SNV	PCLO:NM_014510:exon3:c.T3207C:p.G106	NOVEL		490	0	0	1	332	18	0.226411	1				
28	chr5	14477012	14477012	A	T	exonic	TRIO		nonsynonymous SNV	TRIO:NM_007118:exon41:c.A6093T:p.L203	DBSNP		166	0	0	1	218	12	0.226252	1				
28	chr14	21992355	21992355	G	C	exonic	SALL2		nonsynonymous SNV	SALL2:NM_001364564:exon2:c.C1501G:p.	NOVEL		83	0	0	1	81	8	0.21595	1				
28	chr15	69560600	69560600	G	T	exonic	GLCE		nonsynonymous SNV	GLCE:NM_001324092:exon4:c.G100T:p.D3	NOVEL		340	0	0	1	267	14	0.209371	1				
28	chr10	104119996	104119996	C	A	exonic	GBF1		synonymous SNV	GBF1:NM_001199378:exon12:c.C1236A:p.	NOVEL		324	0	0	1	389	25	0.205461	1				
28	chr10	29770564	29770564	C	A	exonic	SVIL		synonymous SNV	SVIL:NM_003174:exon26:c.G3771T:p.T125	NOVEL		313	1	0.00678602	1	248	23	0.203897	1				
28	chr2	128397867	128397867	G	T	exonic	LIMS2		stopgain	LIMS2:NM_001256542:exon5:c.C324A:p.C	NOVEL		109	0	0	1	95	6	0.202125	1				
28	chr7	111846861	111846861	G	C	exonic	ZNF277		synonymous SNV	ZNF277:NM_021994:exon1:c.G90C:p.G30	NOVEL		349	0	0	1	272	13	0.200813	1				
28	chr18	3215010	3215010	T	G	exonic	MYOM1		nonsynonymous SNV	MYOM1:NM_003803:exon2:c.A212C:p.Q7	DBSNP		106	0	0	1	82	5	0.195543	1				
28	chr3	63542306	63542306	A	T	exonic	SYNPR		synonymous SNV	SYNPR:NM_144642:exon3:c.A237T:p.A79A	COSMIC	ID=COSM378168	228	0	0	1	149	13	0.192788	1				
28	chr6	10930695	10930695	C	T	exonic	SYCP2L		synonymous SNV	SYCP2L:NM_001040274:exon19:c.C1581T:	NOVEL		200	0	0	1	150	9	0.192591	1				
28	chr7	38783002	38783002	G	C	exonic	VPS41		nonsynonymous SNV	VPS41:NM_080631:exon23:c.C2047G:p.P6	NOVEL		481	0	0	1	464	17	0.190939	1				
28	chr18	10979647	10979647	G	A	exonic	PIEZO2		nonsynonymous SNV	PIEZO2:NM_022068:exon3:c.C172T:p.R58	DBSNP		469	1	0.006659	1	360	21	0.187536	1				
28	chr12	112608975	112608975	G	A	exonic	HECTD4		nonsynonymous SNV	HECTD4:NM_001109662:exon68:c.C11908	NOVEL		159	0	0	1	135	10	0.183268	1				
28	chr12	2558145	2558145	C	A	exonic	CACNA1C		synonymous SNV	CACNA1C:NM_000719:exon4:c.C481A:p.R1	NOVEL		570	0	0	1	427	24	0.181061	1				
28	chr3	52431774	52431774	T	C	exonic	DNAH1		nonsynonymous SNV	DNAH1:NM_015512:exon74:c.T11839C:p.F	NOVEL		358	0	0	1	248	20	0.179287	1				
28	chr19	45204735	45204735	G	C	exonic	CEACAM16		synonymous SNV	CEACAM16:NM_001039213:exon2:c.G9C:p	NOVEL		137	0	0	1	128	7	0.176423	1				
28	chr1	26648540	26648540	C	A	exonic	CRYBG2		synonymous SNV	CRYBG2:NM_001039775:exon20:c.G4905T	NOVEL		297	0	0	1	266	21	0.175788	1				
28	chr2	62450314	62450314	A	T	exonic	B3GNT2		nonsynonymous SNV	B3GNT2:NM_001319075:exon2:c.A959T:p	NOVEL		484	1	0.00645305	1	343	19	0.163963	1				
28	chr11	20070555	20070555	G	C	exonic	NAV2		nonsynonymous SNV	NAV2:NM_001111019:exon6:c.G1442C:p.S	NOVEL		294	0	0	1	258	13	0.163217	1				
28	chr1	100381011	100381011	G	T	exonic	AGL		nonsynonymous SNV	AGL:NM_000028:exon31:c.G4228T:p.G141	NOVEL		122	0	0	1	202	14	0.155714	1				
28	chr9	116931522	116931522	G	T	exonic	COL27A1		nonsynonymous SNV	COL27A1:NM_032888:exon3:c.G1687T:p.A	NOVEL		253	0	0	1	218	15	0.154663	1				
28	chr6	49494239	49494239	C	G	exonic	GLYATL3		nonsynonymous SNV	GLYATL3:NM_001010904:exon6:c.C479G:p	NOVEL		554	0	0	1	410	19	0.150691	1				
28	chr7	18066660	18066660	G	T	exonic	PRPS1L1		nonsynonymous SNV	PRPS1L1:NM_175886:exon1:c.C746A:p.T24	NOVEL		688	0	0	1	633	18	0.149376	1				
28	chr12	120586071	120586071	C	T	exonic	GCN1		synonymous SNV	GCN1:NM_006836:exon37:c.G4626A:p.K15	NOVEL		281	0	0	1	185	11	0.149139	1				
28	chr10	73122141	73122141	G	T	exonic	SLC29A3		nonsynonymous SNV	SLC29A3:NM_001363518:exon6:c.G970T:p	NOVEL		336	0	0	1	295	17	0.130902	1				
28	chr8	122626543	122626543	G	A	exonic	HAS2		synonymous SNV	HAS2:NM_005328:exon4:c.C1465T:p.L489	DBSNP		654	0	0	1	851	26	0.130517	1				
28	chr17	7760363	7760363	C	G	exonic	NAA38		nonsynonymous SNV	NAA38:NM_032356:exon1:c.G379C:p.G12	NOVEL		193	0	0	1	119	6	0.115317	1				
28	chr19	3574511	3574511	C	A	exonic	HMG20B		nonsynonymous SNV	HMG20B:NM_006339:exon4:c.C278A:p.P9	NOVEL		442	0	0	1	496	13	0.11244	1				
28	chr15	43440986	43440986	G	T	exonic	TMEM62		stopgain	TMEM62:NM_001347019:exon3:c.G37T:p.	NOVEL		858	0	0	1	680	18	0.108371	1				
28	chr18	31324527	31324527	C	T	exonic	ASXL3		nonsynonymous SNV	ASXL3:NM_030632:exon12:c.C4715T:p.P15	DBSNP		477	0	0	1	358	11	0.101428	1				
28	chr4	87662833	87662833	A	G	exonic	PTPN13		nonsynonymous SNV	PTPN13:NM_006264:exon16:c.A2351G:p.H	NOVEL		311	1	0.00682952	1	262	11	0.0968016	1				
28	chr19	20044895	20044895	C	T	exonic	ZNF93		synonymous SNV	ZNF93:NM_031218:exon4:c.C1131T:p.A37	DBSNP+COSMIC	ID=COSM310198	149	9	0.174909	1	206	4	0.0648084	1				
28	chr1	34643703	34643703	C	A	exonic	C1orf94		nonsynonymous SNV	C1orf94:NM_001134734:exon1:c.C313A:p.	NOVEL		135	134	1.06144	1	221	1	0.0108218	1				
28	chr3	37034496	37034496	G	C	exonic	EPM2AIP1		nonsynonymous SNV	EPM2AIP1:NM_014805:exon1:c.C73G:p.R2	NOVEL		227	101	0.963728	1	248	1	0.00964835	1				
28	chr8	133920582	133920582	C	A	exonic	TG		synonymous SNV	TG:NM_003235:exon18:c.C3999A:p.I1333I	NOVEL		168	150	NA	1	245	0	0	1				
28	chr1	228467782	228467782	G	A	exonic	OBSCN		nonsynonymous SNV	OBSCN:NM_001098623:exon28:c.G7657A	NOVEL		183	71	1.15468	1	179	0	0	1				
28	chr3	98251101	98251101	T	G	exonic	GPR15		nonsynonymous SNV	GPR15:NM_005290:exon1:c.T224G:p.L75R	NOVEL		152	88	1.14757	1	214	0	0	1				
28	chr1	234367463	234367463	A	T	exonic	SLC35F3		nonsynonymous SNV	SLC35F3:NM_001300845:exon2:c.A377T:p	NOVEL		249	88	1.07867	1	288	0	0	1				
28	chr1	6533361	6533361	G	A	exonic	PLEKHG5		nonsynonymous SNV	PLEKHG5:NM_001042664:exon8:c.C745T:p	NOVEL		47	48	1.07662	1	84	0	0	1				
28	chr12	48723543	48723543	C	T	exonic	H1FNT		nonsynonymous SNV	H1FNT:NM_181788:exon1:c.C469T:p.R157	NOVEL		54	27	1.04324	1	79	0	0	1				
28	chr17	16203203	16203203	G	A	exonic	PIGL		nonsynonymous SNV	PIGL:NM_004278:exon3:c.G337A:p.D113N	NOVEL		110	55	1.04324	1	107	0	0	1				
28	chr18	74091535	74091535	C	A	exonic	ZNF516		synonymous SNV	ZNF516:NM_014643:exon4:c.G2535T:p.P8	NOVEL		411	202	1.03133	1	468	0	0	1				
28	chr3	178917649	178917649	C	G	exonic	PIK3CA		nonsynonymous SNV	PIK3CA:NM_006218:exon3:c.C524G:p.P175	NOVEL		273	134	1.03043	1	336	0	0	1				
28	chr9	88959987	88959987	T	C	exonic	TUT7		nonsynonymous SNV	TUT7:NM_001185059:exon5:c.A902G:p.K3	DBSNP+COSMIC	ID=COSM713542	162	144	1.00273	1	278	0	0	1				
28	chr6	10894396	10894396	G	T	exonic	SYCP2L		stopgain	SYCP2L:NM_001040274:exon4:c.G295T:p.	NOVEL		426	197	0.989658	1	472	0	0	1				
28	chr9	38413246	38413246	C	A	exonic	IGFBPL1		synonymous SNV	IGFBPL1:NM_001007563:exon3:c.G675T:p	NOVEL		106	90	0.978433	1	145	0	0	1				
28	chr12	85695206	85695206	C	T	exonic	ALX1		stopgain	ALX1:NM_006982:exon4:c.C934T:p.R312X	COSMIC	ID=COSM118265	270	447	0.975585	2	610	0	0	1				
28	chr7	143027978	143027978	A	G	exonic	CLCN1		nonsynonymous SNV	CLCN1:NM_000083:exon8:c.A967G:p.N323	DBSNP		160	51	0.972298	1	211	0	0	1				
28	chr1	233161052	233161052	T	A	exonic	PCNX2		nonsynonymous SNV	PCNX2:NM_014801:exon26:c.A4445T:p.H1	NOVEL		218	66	0.959977	1	228	0	0	1				
28	chr3	191087813	191087813	T	A	exonic	CCDC50		nonsynonymous SNV	CCDC50:NM_174908:exon5:c.T436A:p.Y14	NOVEL		188	83	0.958552	1	241	0	0	1				
28	chr7	142880940	142880940	C	T	exonic	TAS2R39		synonymous SNV	TAS2R39:NM_176881:exon1:c.C429T:p.S14	DBSNP		641	188	0.912252	1	732	0	0	1				
28	chr1	57415410	57415410	C	T	exonic	C8B		nonsynonymous SNV	C8B:NM_000066:exon6:c.G682A:p.E228K,	DBSNP+COSMIC	ID=COSM134365	110	82	0.910034	1	142	0	0	1				
28	chr10	124336202	124336202	T	C	exonic	DMBT1		nonsynonymous SNV	DMBT1:NM_001320644:exon7:c.T571C:p.(	NOVEL		96	59	0.811083	1	204	0	0	1				
28	chr5	140799830	140799830	A	G	exonic	PCDHGB7		nonsynonymous SNV	PCDHGB7:NM_018927:exon1:c.A2404G:p.	NOVEL		109	64	0.788277	1	157	0	0	1				
28	chr2	61711179	61711179	G	C	exonic	XPO1		nonsynonymous SNV	XPO1:NM_003400:exon21:c.C2570G:p.S85	NOVEL		405	43	0.300398	1	302	0	0	1				
28	chr8	77766721	77766721	C	G	exonic	ZFHX4		nonsynonymous SNV	ZFHX4:NM_024721:exon10:c.C7564G:p.Q2	NOVEL		297	42	0.263994	1	294	0	0	1				
28	chr2	143712419	143712419	T	C	exonic	KYNU		synonymous SNV	KYNU:NM_001032998:exon5:c.T414C:p.T1	DBSNP		171	13	0.221122	1	108	0	0	1				
28	chr19	44840833	44840833	C	A	exonic	ZNF112		stopgain	ZNF112:NM_001348282:exon3:c.G163T:p.	NOVEL		246	15	0.179869	1	247	0	0	1				
28	chr10	33481243	33481243	C	A	exonic	NRP1		nonsynonymous SNV	NRP1:NM_001244972:exon13:c.G2007T:p	NOVEL		116	8	0.137472	1	105	0	0	1				
28	chr4	187207571	187207571	T	C	exonic	F11		nonsynonymous SNV	F11:NM_000128:exon13:c.T1483C:p.S495	NOVEL		189	12	0.127213	1	153	0	0	1				
28	chr19	36210393	36210393	A	T	exonic	KMT2B		nonsynonymous SNV	KMT2B:NM_014727:exon2:c.A386T:p.D129	NOVEL		381	16	0.126135	1	290	0	0	1				
28	chr19	36210392	36210392	G	C	exonic	KMT2B		nonsynonymous SNV	KMT2B:NM_014727:exon2:c.G385C:p.D12	NOVEL		381	13	0.103265	1	290	0	0	1				
28	chrX	15267059	15267059	T	A	splicing	ASB9	NM_024087:exon6:c.5			NOVEL		80	0	NA	1	21	53	NA	1				
28	chr19	6916380	6916380	G	T	splicing	ADGRE1	NM_001256252:exon1			NOVEL		376	0	0	1	326	87	0.92739	1				
28	chr7	81620500	81620500	C	T	splicing	CACNA2D1	NM_000722:exon21:c			NOVEL		147	121	0.908097	2	206	43	0.760261	1				
28	chr14	53187700	53187700	G	C	splicing	PSMC6	NM_001366414:exon1			NOVEL		357	0	0	1	270	27	0.218404	1				
29	chr4	17841449	17841449	G	A	exonic	NCAPG		nonsynonymous SNV	NCAPG:NM_022346:exon17:c.G2617A:p.E	NOVEL		45	36	0.949443	1	42	55	1.2509	1				
29	chr10	98740989	98740989	A	C	exonic	LCOR		nonsynonymous SNV	LCOR:NM_001346516:exon8:c.A772C:p.I25	NOVEL		136	134	1.06021	1	144	160	1.16112	1				
29	chr18	33795723	33795723	G	A	exonic	MOCOS		nonsynonymous SNV	MOCOS:NM_017947:exon8:c.G1580A:p.R5	DBSNP		120	83	0.873441	1	133	138	1.12342	1				
29	chr16	22330170	22330170	T	C	exonic	POLR3E		synonymous SNV	POLR3E:NM_001258034:exon12:c.T781C:p	NOVEL		55	44	0.949443	1	73	71	1.08774	1				
29	chr4	151935740	151935740	C	T	exonic	LRBA		nonsynonymous SNV	LRBA:NM_001199282:exon2:c.G55A:p.G19	NOVEL		54	58	1.10627	1	67	65	1.08635	1				
29	chr3	195487957	195487957	C	T	exonic	MUC4		synonymous SNV	MUC4:NM_138297:exon14:c.G1785A:p.K5	NOVEL		60	93	0.953173	2	72	141	1.06118	2				
29	chr16	22330210	22330210	T	G	exonic	POLR3E		nonsynonymous SNV	POLR3E:NM_001258034:exon12:c.T821G:p	NOVEL		90	71	0.942072	1	109	100	1.05556	1				
29	chr10	112653891	112653891	T	C	exonic	PDCD4		nonsynonymous SNV	PDCD4:NM_001199492:exon9:c.T991C:p.S	NOVEL		65	61	1.03421	1	92	84	1.05293	1				
29	chr21	38128878	38128878	G	A	exonic	HLCS		synonymous SNV	HLCS:NM_001352514:exon10:c.C2415T:p.S	DBSNP		171	232	0.902741	2	183	336	1.03782	2				
29	chr1	228602690	228602690	C	T	exonic	TRIM17		nonsynonymous SNV	TRIM17:NM_001024940:exon2:c.G84A:p.M	NOVEL		81	62	0.926205	1	107	95	1.03754	1				
29	chr11	85606408	85606408	A	T	exonic	CCDC83		nonsynonymous SNV	CCDC83:NM_001286159:exon6:c.A584T:p.	NOVEL		248	100	0.90122	1	259	123	1.03234	1				
29	chr17	7727439	7727439	G	A	exonic	DNAH2		nonsynonymous SNV	DNAH2:NM_020877:exon76:c.G11479A:p.	NOVEL		119	91	0.925707	1	126	108	1.01821	1				
29	chr3	7503320	7503320	C	T	exonic	GRM7		nonsynonymous SNV	GRM7:NM_000844:exon7:c.C1426T:p.R47	COSMIC	ID=COSM432724	85	139	0.973077	2	106	181	1.01099	2				
29	chr2	139316729	139316729	G	A	exonic	SPOPL		synonymous SNV	SPOPL:NM_001001664:exon6:c.G618A:p.V	DBSNP		103	71	0.871687	1	86	72	1.00532	1				
29	chr12	124846816	124846816	G	A	exonic	NCOR2		nonsynonymous SNV	NCOR2:NM_001077261:exon23:c.C2902T:	DBSNP		43	27	0.823981	1	36	30	1.00279	1				
29	chr11	28112171	28112171	A	G	exonic	KIF18A		nonsynonymous SNV	KIF18A:NM_031217:exon5:c.T692C:p.V231	NOVEL		87	148	0.987584	2	110	178	0.990783	2				
29	chr19	41086518	41086518	T	G	exonic	SHKBP1		nonsynonymous SNV	SHKBP1:NM_138392:exon8:c.T609G:p.H20	NOVEL		122	219	1.00709	2	153	255	0.988298	2				
29	chr11	959447	959447	A	G	exonic	AP2A2		synonymous SNV	AP2A2:NM_001242837:exon2:c.A78G:p.K2	NOVEL		108	133	0.865396	2	106	174	0.982651	2				
29	chr14	23778069	23778069	G	A	exonic	BCL2L2		synonymous SNV	BCL2L2:NM_001199839:exon4:c.G477A:p.	DBSNP+COSMIC	ID=COSM203135	80	69	0.989269	1	102	81	0.976483	1				
29	chr9	135447794	135447794	C	A	exonic	CFAP77		nonsynonymous SNV	CFAP77:NM_001282957:exon6:c.C752A:p.	NOVEL		184	45	0.764872	1	199	64	0.963419	1				
29	chr11	88924425	88924425	C	T	exonic	TYR		nonsynonymous SNV	TYR:NM_000372:exon2:c.C875T:p.T292M	DBSNP+COSMIC	ID=COSM121052	145	243	0.982098	2	167	249	0.959527	2				
29	chr15	58971387	58971387	T	C	exonic	ADAM10		synonymous SNV	ADAM10:NM_001110:exon4:c.A420G:p.A1	NOVEL		113	212	1.0229	2	139	205	0.955315	2				
29	chr12	13716700	13716700	G	A	exonic	GRIN2B		nonsynonymous SNV	GRIN2B:NM_000834:exon13:c.C3472T:p.R	COSMIC	ID=COSM125379	97	80	0.965535	1	137	102	0.941528	1				
29	chr2	102803510	102803510	C	A	exonic	IL1RL2		synonymous SNV	IL1RL2:NM_001351447:exon1:c.C36A:p.L1	NOVEL		75	43	0.778463	1	78	58	0.940849	1				
29	chr2	128941335	128941335	A	G	exonic	UGGT1		nonsynonymous SNV	UGGT1:NM_020120:exon38:c.A4331G:p.D	NOVEL		97	78	0.952156	1	128	93	0.928371	1				
29	chr17	45822631	45822631	T	C	exonic	TBX21		nonsynonymous SNV	TBX21:NM_013351:exon6:c.T1507C:p.S50	NOVEL		223	111	1.04229	1	312	124	0.899442	1				
29	chr12	25398281	25398281	C	T	exonic	KRAS		nonsynonymous SNV	KRAS:NM_001369786:exon2:c.G38A:p.G1	DBSNP+COSMIC	ID=COSM114013	121	56	0.675875	1	140	95	0.891839	1				
29	chr11	82642943	82642943	T	C	exonic	DDIAS		nonsynonymous SNV	DDIAS:NM_001363481:exon5:c.T563C:p.F	NOVEL		590	211	0.826153	1	666	254	0.88517	1				
29	chr7	138268671	138268671	C	T	exonic	TRIM24		nonsynonymous SNV	TRIM24:NM_003852:exon18:c.C2768T:p.S	NOVEL		126	74	1.16041	1	181	67	0.866172	1				
29	chr11	101857704	101857704	T	C	exonic	CEP126		nonsynonymous SNV	CEP126:NM_020802:exon9:c.T3176C:p.I10	NOVEL		106	36	0.795105	1	117	43	0.861647	1				
29	chr6	7880559	7880559	G	A	exonic	BMP6		nonsynonymous SNV	BMP6:NM_001718:exon7:c.G1525A:p.A50	NOVEL		53	55	1.0879	1	79	49	0.844534	1				
29	chr12	78594324	78594324	T	C	exonic	NAV3		nonsynonymous SNV	NAV3:NM_014903:exon37:c.T6721C:p.Y22	NOVEL		25	25	1.06812	1	39	24	0.84043	1				
29	chr3	50147050	50147050	A	C	exonic	RBM5		nonsynonymous SNV	RBM5:NM_005778:exon15:c.A1207C:p.T4	NOVEL		134	43	0.761913	1	171	60	0.821443	1				
29	chr5	32774927	32774927	G	A	exonic	NPR3		synonymous SNV	NPR3:NM_001364460:exon3:c.G402A:p.Q	NOVEL		363	133	0.84097	1	480	162	0.809023	1				
29	chr1	8923389	8923389	G	C	exonic	ENO1		nonsynonymous SNV	ENO1:NM_001201483:exon9:c.C802G:p.Q	NOVEL		64	50	0.936951	1	86	47	0.77961	1				
29	chr8	107719241	107719241	G	T	exonic	OXR1		stopgain	OXR1:NM_181354:exon7:c.G1471T:p.E491	NOVEL		220	113	0.724913	1	278	145	0.756238	1				
29	chr3	47614239	47614239	G	A	exonic	CSPG5		nonsynonymous SNV	CSPG5:NM_001206942:exon3:c.C905T:p.T	DBSNP		258	68	0.654187	1	360	107	0.724611	1				
29	chr19	501748	501748	C	T	exonic	MADCAM1		synonymous SNV	MADCAM1:NM_130760:exon4:c.C747T:p.P	DBSNP		49	5	0.290393	1	62	14	0.582576	1				
29	chr9	117086341	117086341	C	T	exonic	ORM1		nonsynonymous SNV	ORM1:NM_000607:exon3:c.C301T:p.R101	DBSNP+COSMIC	ID=COSM493636	1527	166	0.405562	1	1923	221	0.432107	1				
29	chr19	49335967	49335967	C	T	exonic	HSD17B14		nonsynonymous SNV	HSD17B14:NM_016246:exon4:c.G233A:p.R	DBSNP+COSMIC	ID=COSM722967	229	21	0.263445	1	256	35	0.380376	1				
29	chr2	114685016	114685016	A	T	exonic	ACTR3		nonsynonymous SNV	ACTR3:NM_001277140:exon4:c.A167T:p.D	NOVEL		108	0	0	1	100	6	0.124875	1				
29	chr1	8924105	8924105	C	T	exonic	ENO1		stopgain	ENO1:NM_001201483:exon8:c.G633A:p.W	NOVEL		131	5	0.0785385	1	161	7	0.0919221	1				
29	chr17	76422678	76422678	C	T	exonic	DNAH17		nonsynonymous SNV	DNAH17:NM_173628:exon79:c.G12775A:p	COSMIC	ID=COSM452451	146	7	0.143488	1	189	4	0.0655452	1				
29	chr14	94830884	94830884	C	T	exonic	SERPINA2		unknown	UNKNOWN	NOVEL		251	10	0.0818486	1	326	5	0.0333252	1				
30	chr19	51503950	51503950	G	A	exonic	KLK8		nonsynonymous SNV	KLK8:NM_144505:exon3:c.C95T:p.T32I	DBSNP		117	60	1.05148	1	108	43	1.28499	1				
30	chr1	28315164	28315164	C	T	exonic	EYA3		synonymous SNV	EYA3:NM_001282560:exon15:c.G1284A:p.	NOVEL		75	82	1.09801	1	143	74	1.19778	1				
30	chr20	61958164	61958164	G	A	exonic	COL20A1		nonsynonymous SNV	COL20A1:NM_020882:exon31:c.G3517A:p	DBSNP+COSMIC	ID=COSM410032	75	92	1.15814	1	134	68	1.18239	1				
30	chr4	8108266	8108266	G	A	exonic	ABLIM2		nonsynonymous SNV	ABLIM2:NM_001130083:exon2:c.C109T:p.	DBSNP		133	124	1.01433	1	211	104	1.15965	1				
30	chr4	7640121	7640121	G	A	exonic	SORCS2		nonsynonymous SNV	SORCS2:NM_020777:exon4:c.G715A:p.A2	DBSNP+COSMIC	ID=COSM472905	191	192	1.05389	1	246	116	1.12552	1				
30	chr19	44079610	44079610	T	C	exonic	XRCC1		startloss	XRCC1:NM_006297:exon1:c.A1G:p.M1?	DBSNP		128	54	0.920338	1	137	44	1.09694	1				
30	chr14	60194346	60194346	G	A	exonic	RTN1		synonymous SNV	RTN1:NM_021136:exon3:c.C1056T:p.S352	DBSNP+COSMIC	ID=COSM677037	132	116	0.983327	1	201	91	1.09462	1				
30	chr7	107635354	107635354	G	A	exonic	LAMB1		nonsynonymous SNV	LAMB1:NM_002291:exon5:c.C401T:p.T134	NOVEL		54	72	0.886251	2	81	73	1.06938	2				
30	chr3	133524808	133524808	C	T	exonic	SRPRB		nonsynonymous SNV	SRPRB:NM_021203:exon2:c.C116T:p.S39L	NOVEL		197	121	1.18027	1	249	76	1.05509	1				
30	chr15	45398792	45398792	G	T	exonic	DUOX2		nonsynonymous SNV	DUOX2:NM_001363711:exon16:c.C1879A:	NOVEL		123	58	0.993972	1	132	40	1.04928	1				
30	chr22	18300089	18300089	C	G	exonic	MICAL3		nonsynonymous SNV	MICAL3:NM_015241:exon26:c.G5338C:p.V	NOVEL		50	52	1.07175	1	69	29	1.03938	1				
30	chr9	138649220	138649220	A	T	exonic	KCNT1		nonsynonymous SNV	KCNT1:NM_001272003:exon8:c.A608T:p.N	DBSNP		138	66	1.00355	1	138	41	1.03357	1				
30	chr10	15701035	15701035	G	A	exonic	ITGA8		nonsynonymous SNV	ITGA8:NM_001291494:exon9:c.C866T:p.T2	DBSNP+COSMIC	ID=COSM916733	74	91	1.15944	1	144	60	1.03306	1				
30	chr19	42224969	42224969	G	A	exonic	CEACAM5		synonymous SNV	CEACAM5:NM_001291484:exon8:c.G1899	DBSNP		319	157	1.0231	1	420	124	1.02856	1				
30	chr9	112898644	112898644	G	A	exonic	PALM2-AKAP2		nonsynonymous SNV	PALM2-AKAP2:NM_001004065:exon2:c.G	DBSNP+COSMIC	ID=COSM673711	297	153	1.05464	1	450	124	0.974694	1				
30	chr7	148801034	148801034	G	A	exonic	ZNF425		synonymous SNV	ZNF425:NM_001001661:exon4:c.C1929T:	COSMIC	ID=COSM108710	345	165	1.00355	1	440	121	0.973153	1				
30	chr3	140785209	140785209	G	A	exonic	SPSB4		nonsynonymous SNV	SPSB4:NM_080862:exon2:c.G263A:p.R88H	NOVEL		135	73	1.08864	1	175	48	0.971169	1				
30	chr5	156479590	156479590	G	A	exonic	HAVCR1		nonsynonymous SNV	HAVCR1:NM_012206:exon3:c.C455T:p.T15	DBSNP+COSMIC	ID=COSM616983	301	153	1.04535	1	396	108	0.966834	1				
30	chr13	99908124	99908124	C	A	exonic	GPR18		startloss	GPR18:NM_001098200:exon2:c.G3T:p.M1	NOVEL		155	116	0.899871	1	305	113	0.949523	1				
30	chr11	13408152	13408152	C	T	exonic	ARNTL		nonsynonymous SNV	ARNTL:NM_001351817:exon16:c.C1376T:	NOVEL		80	39	1.01658	1	94	25	0.947876	1				
30	chr2	125405393	125405393	C	T	exonic	CNTNAP5		synonymous SNV	CNTNAP5:NM_001367498:exon13:c.C1935	DBSNP+COSMIC	ID=COSM100610	76	49	0.824096	1	120	44	0.94235	1				
30	chr19	56511125	56511125	G	A	exonic	NLRP5		nonsynonymous SNV	NLRP5:NM_153447:exon1:c.G34A:p.A12T	DBSNP		80	62	1.35434	1	141	37	0.93797	1				
30	chr14	20502642	20502642	G	A	exonic	OR4K13		synonymous SNV	OR4K13:NM_001004714:exon1:c.C276T:p.	NOVEL		153	116	0.906561	1	288	104	0.93186	1				
30	chr19	2250362	2250362	A	G	exonic	AMH		nonsynonymous SNV	AMH:NM_000479:exon2:c.A439G:p.R147G	NOVEL		24	49	1.04104	2	43	30	0.927205	2				
30	chr2	197081795	197081795	T	G	exonic	HECW2		synonymous SNV	HECW2:NM_001304840:exon25:c.A3363C	NOVEL		172	140	0.943333	1	330	118	0.92514	1				
30	chr11	8752173	8752173	A	T	exonic	ST5		nonsynonymous SNV	ST5:NM_213618:exon3:c.T664A:p.F222I,S	NOVEL		85	155	1.00165	2	139	94	0.910124	2				
30	chr1	119964624	119964624	C	T	exonic	HSD3B2		nonsynonymous SNV	HSD3B2:NM_000198:exon4:c.C500T:p.A16	DBSNP+COSMIC	ID=COSM502977	113	107	1.02247	1	214	74	0.902491	1				
30	chr1	198725176	198725176	A	G	exonic	PTPRC		nonsynonymous SNV	PTPRC:NM_080921:exon30:c.A3304G:p.N1	NOVEL		115	122	1.08219	1	275	94	0.894757	1				
30	chr9	116045710	116045710	T	G	exonic	PRPF4		nonsynonymous SNV	PRPF4:NM_001244926:exon6:c.T602G:p.I2	NOVEL		106	49	0.980594	1	164	39	0.866816	1				
30	chr11	45986992	45986992	C	T	exonic	PHF21A		synonymous SNV	PHF21A:NM_001101802:exon9:c.G867A:p	NOVEL		210	71	0.783749	1	250	59	0.861494	1				
30	chr12	63544485	63544485	G	A	exonic	AVPR1A		synonymous SNV	AVPR1A:NM_000706:exon1:c.C132T:p.D44	DBSNP+COSMIC	ID=COSM466479	129	154	1.144	1	266	86	0.858142	1				
30	chr7	83689815	83689815	A	T	exonic	SEMA3A		stopgain	SEMA3A:NM_006080:exon5:c.T513A:p.Y1	NOVEL		222	115	1.0585	1	365	85	0.852246	1				
30	chr1	46085996	46085996	T	C	exonic	CCDC17		nonsynonymous SNV	CCDC17:NM_001114938:exon13:c.A1820G	DBSNP		81	64	0.927905	1	162	51	0.840996	1				
30	chr9	32480338	32480338	G	A	exonic	DDX58		nonsynonymous SNV	DDX58:NM_014314:exon12:c.C1653T:p.A5	NOVEL		102	188	1.00544	2	212	124	0.832551	2				
30	chr2	121736112	121736112	G	A	exonic	GLI2		nonsynonymous SNV	GLI2:NM_001371271:exon10:c.G1471A:p.	NOVEL		96	79	0.949031	1	168	51	0.817955	1				
30	chr3	193201777	193201777	G	T	exonic	ATP13A4		nonsynonymous SNV	ATP13A4:NM_032279:exon8:c.C756A:p.H2	NOVEL		155	78	1.0384	1	268	59	0.814072	1				
30	chr19	33792274	33792274	G	A	exonic	CEBPA		synonymous SNV	CEBPA:NM_001285829:exon1:c.C690T:p.S	NOVEL		149	63	0.921785	1	174	38	0.808826	1				
30	chr16	90130080	90130080	T	C	exonic	PRDM7		nonsynonymous SNV	PRDM7:NM_001098173:exon5:c.A448G:p.	NOVEL		55	45	0.946028	1	46	38	0.79447	2				
30	chr1	21031004	21031004	G	A	exonic	KIF17		synonymous SNV	KIF17:NM_001122819:exon5:c.C1059T:p.Y	NOVEL		125	94	0.902351	1	171	49	0.782306	1				
30	chr17	36700142	36700142	C	T	exonic	SRCIN1		synonymous SNV	SRCIN1:NM_025248:exon18:c.G3333A:p.A	DBSNP		58	72	1.16434	1	59	46	0.769382	2				
30	chr16	56487228	56487228	A	T	exonic	OGFOD1		nonsynonymous SNV	OGFOD1:NM_001324357:exon2:c.A205T:p	NOVEL		140	109	0.920277	1	276	76	0.758358	1				
30	chr1	115258745	115258745	C	G	exonic	NRAS		nonsynonymous SNV	NRAS:NM_002524:exon2:c.G37C:p.G13R	DBSNP+COSMIC	ID=COSM569;OC	62	52	0.958937	1	102	28	0.756516	1				
30	chr16	84126846	84126846	C	G	exonic	MBTPS1		nonsynonymous SNV	MBTPS1:NM_003791:exon6:c.G793C:p.G2	NOVEL		75	57	0.907805	1	119	32	0.744349	1				
30	chr6	3077187	3077187	A	T	exonic	RIPK1		nonsynonymous SNV	RIPK1:NM_001354930:exon2:c.A130T:p.M	NOVEL		20	26	1.18825	1	44	11	0.702479	1				
30	chr16	56487195	56487195	G	T	exonic	OGFOD1		nonsynonymous SNV	OGFOD1:NM_001324357:exon2:c.G172T:p	NOVEL		117	98	0.958251	1	237	59	0.700106	1				
30	chr5	121187900	121187900	A	G	exonic	FTMT		nonsynonymous SNV	FTMT:NM_177478:exon1:c.A242G:p.N81S	DBSNP		392	6	0.046762	1	464	23	0.213087	1				
30	chr16	50820775	50820775	T	G	exonic	CYLD		stopgain	CYLD:NM_001042355:exon12:c.T1950G:p.	NOVEL		36	9	0.220457	1	91	8	0.203022	1				
30	chr19	16594840	16594840	G	A	exonic	CALR3		synonymous SNV	CALR3:NM_145046:exon5:c.C579T:p.S193	DBSNP+COSMIC	ID=COSM407515	325	24	0.21331	1	360	12	0.145561	1				
30	chr11	130284433	130284433	C	T	exonic	ADAMTS8		nonsynonymous SNV	ADAMTS8:NM_007037:exon5:c.G1559A:p.	NOVEL		234	31	0.362861	1	280	0	0	1				
30	chr3	183754273	183754273	T	G	exonic	HTR3D		nonsynonymous SNV	HTR3D:NM_182537:exon3:c.T86G:p.I29S,H	NOVEL		136	7	0.15184	1	178	0	0	1				
30	chr17	18023206	18023206	C	T	exonic	MYO15A		synonymous SNV	MYO15A:NM_016239:exon2:c.C1092T:p.Y	NOVEL		545	20	0.0744172	1	611	0	0	1				
30	chr16	50827577	50827577	T	C	splicing	CYLD	NM_015247:exon18:c			NOVEL		127	4	0.0336576	1	243	9	0.0897284	1				
31	chrX	47044965	47044965	G	C	exonic	RBM10		nonsynonymous SNV	RBM10:NM_001204466:exon19:c.G2060C	NOVEL		2	54	NA	1	14	53	NA	1	76	73	NA	1
31	chrX	71352024	71352024	C	T	exonic	NHSL2		nonsynonymous SNV	NHSL2:NM_001013627:exon2:c.C376T:p.P	COSMIC	ID=COSM465090	44	0	NA	1	35	0	NA	1	76	5	NA	1
31	chr1	233297059	233297059	G	C	exonic	PCNX2		synonymous SNV	PCNX2:NM_014801:exon18:c.C3324G:p.L1	NOVEL		70	7	0.288354	1	40	66	1.36548	2	84	83	1.4898	2
31	chr17	31415905	31415905	G	A	exonic	ASIC2		synonymous SNV	ASIC2:NM_001094:exon3:c.C810T:p.T270T	DBSNP		35	11	0.681915	1	25	23	1.14333	1	55	30	1.41004	1
31	chr14	21991106	21991106	G	T	exonic	SALL2		nonsynonymous SNV	SALL2:NM_001364564:exon2:c.C2750A:p.A	DBSNP		44	34	0.946724	1	49	39	1.05747	1	123	65	1.38129	1
31	chr8	95680390	95680390	A	T	exonic	ESRP1		nonsynonymous SNV	ESRP1:NM_001034915:exon10:c.A1145T:p	NOVEL		28	26	1.04573	1	64	60	1.15456	1	115	58	1.3394	1
31	chr1	32713068	32713068	G	A	exonic	FAM167B		nonsynonymous SNV	FAM167B:NM_032648:exon1:c.G46A:p.E1	DBSNP		44	50	1.15526	1	48	35	1.00618	1	98	47	1.29497	1
31	chr17	36485473	36485473	C	T	exonic	GPR179		nonsynonymous SNV	GPR179:NM_001004334:exon11:c.G3979A	DBSNP		176	90	0.964842	1	167	86	0.811079	1	280	129	1.26007	1
31	chr11	122955269	122955269	A	T	exonic	CLMP		synonymous SNV	CLMP:NM_024769:exon3:c.T339A:p.V113V	NOVEL		189	2	0.0227424	1	105	76	1.00189	1	220	95	1.20488	1
31	chr19	55539071	55539071	C	T	exonic	GP6		nonsynonymous SNV	GP6:NM_001083899:exon4:c.G485A:p.R16	NOVEL		210	0	0	1	86	59	0.970889	1	142	61	1.2005	1
31	chr17	80373338	80373338	G	A	exonic	OGFOD3		synonymous SNV	OGFOD3:NM_024648:exon2:c.C240T:p.V8	COSMIC	ID=COSM352385	288	0	0	1	163	63	0.94357	1	245	77	1.19448	1
31	chr12	124416208	124416208	C	T	exonic	DNAH10		synonymous SNV	DNAH10:NM_207437:exon74:c.C12588T:p	DBSNP		124	0	0	1	113	67	0.888153	1	213	89	1.17737	1
31	chr2	175324665	175324665	T	C	exonic	GPR155		nonsynonymous SNV	GPR155:NM_001267051:exon9:c.A1628G:	NOVEL		3	51	1.10679	1	17	75	1.12996	1	115	73	1.17214	1
31	chr14	31775974	31775974	G	A	exonic	HEATR5A		stopgain	HEATR5A:NM_015473:exon31:c.C4930T:p	NOVEL		165	62	0.593205	1	157	116	1.01387	1	350	144	1.16457	1
31	chr19	4511019	4511019	C	A	exonic	PLIN4		nonsynonymous SNV	PLIN4:NM_001367868:exon5:c.G2953T:p.A	NOVEL		443	205	1.00345	1	310	109	0.880551	1	521	158	1.16234	1
31	chr15	77406927	77406927	G	T	exonic	PEAK1		nonsynonymous SNV	PEAK1:NM_024776:exon8:c.C4812A:p.H16	NOVEL		108	34	0.759468	1	175	76	1.0249	1	252	78	1.15675	1
31	chr3	9068659	9068659	C	T	exonic	SRGAP3		synonymous SNV	SRGAP3:NM_001033117:exon13:c.G1488A	DBSNP+COSMIC	ID=COSM481624	273	0	0	1	121	82	0.963836	1	253	102	1.14789	1
31	chr12	21981911	21981911	C	T	exonic	ABCC9		nonsynonymous SNV	ABCC9:NM_005691:exon29:c.G3650A:p.R1	DBSNP		95	104	1.13506	1	108	72	0.954433	1	235	94	1.14146	1
31	chr19	49797228	49797228	G	A	exonic	SLC6A16		nonsynonymous SNV	SLC6A16:NM_014037:exon9:c.C1474T:p.P4	NOVEL		47	51	0.82534	2	82	70	1.09885	1	165	65	1.12905	1
31	chr5	140812636	140812636	C	T	exonic	PCDHGA12		synonymous SNV	PCDHGA12:NM_003735:exon1:c.C2310T:p	DBSNP		86	82	0.894588	1	108	112	1.21473	1	250	98	1.12506	1
31	chr2	27722292	27722292	T	C	exonic	GCKR		nonsynonymous SNV	GCKR:NM_001486:exon7:c.T524C:p.V175A	NOVEL		75	57	1.07258	1	89	68	1.03346	1	195	76	1.1204	1
31	chr11	8148264	8148264	A	C	exonic	RIC3		nonsynonymous SNV	RIC3:NM_001206671:exon5:c.T612G:p.I20	NOVEL		248	0	0	1	225	123	0.843357	1	419	161	1.10899	1
31	chr11	57322043	57322043	C	T	exonic	UBE2L6		synonymous SNV	UBE2L6:NM_004223:exon3:c.G177A:p.P59	DBSNP		51	50	1.0752	1	54	43	1.05775	1	112	40	1.05135	1
31	chr1	173823017	173823017	C	T	exonic	DARS2		nonsynonymous SNV	DARS2:NM_001365212:exon14:c.C1495T:p	COSMIC	ID=COSM272567	29	393	1.47696	2	44	121	1.01646	1	191	102	1.05087	1
31	chr11	17608120	17608120	C	T	exonic	OTOG		synonymous SNV	OTOG:NM_001277269:exon23:c.C2895T:p	DBSNP		40	31	0.948293	1	48	32	0.954433	1	115	41	1.05	1
31	chr1	19423708	19423708	C	T	exonic	UBR4		nonsynonymous SNV	UBR4:NM_020765:exon91:c.G13369A:p.E	DBSNP		27	24	1.02207	1	37	30	1.06839	1	90	32	1.0479	1
31	chr19	47181701	47181701	C	T	exonic	PRKD2		nonsynonymous SNV	PRKD2:NM_001079882:exon15:c.G1819A:	NOVEL		213	87	0.91985	1	103	52	0.800492	1	198	70	1.0435	1
31	chr9	35074953	35074953	T	G	exonic	FANCG		nonsynonymous SNV	FANCG:NM_004629:exon12:c.A1607C:p.D	NOVEL		71	49	0.886858	1	43	33	1.03606	1	104	36	1.02731	1
31	chr2	113513783	113513783	C	T	exonic	CKAP2L		nonsynonymous SNV	CKAP2L:NM_001304361:exon4:c.G670A:p.	NOVEL		220	0	0	1	193	159	1.0778	1	453	156	1.02338	1
31	chr18	76756935	76756935	C	T	exonic	SALL3		synonymous SNV	SALL3:NM_171999:exon3:c.C3516T:p.R117	DBSNP+COSMIC	ID=COSM472351	25	25	1.08595	1	54	34	0.921895	1	114	39	1.01836	1
31	chr11	22387189	22387189	T	C	exonic	SLC17A6		nonsynonymous SNV	SLC17A6:NM_020346:exon7:c.T845C:p.I28	NOVEL		93	63	0.877112	1	99	77	1.04391	1	230	76	0.99225	1
31	chr1	201982310	201982310	A	G	exonic	ELF3		nonsynonymous SNV	ELF3:NM_001114309:exon7:c.A689G:p.D2	NOVEL		341	0	0	1	201	60	0.778131	1	337	85	0.985743	1
31	chr20	39990129	39990129	C	T	exonic	EMILIN3		nonsynonymous SNV	EMILIN3:NM_052846:exon4:c.G2080A:p.A	NOVEL		45	36	0.965287	1	45	24	0.829942	1	89	29	0.98185	1
31	chr9	137623973	137623973	G	A	exonic	COL5A1		synonymous SNV	COL5A1:NM_000093:exon9:c.G1389A:p.P4	DBSNP+COSMIC	ID=COSM274620	51	31	0.821083	1	73	42	0.871439	1	126	41	0.980836	1
31	chr1	202992002	202992002	C	T	exonic	TMEM183A		nonsynonymous SNV	TMEM183A:NM_001322958:exon7:c.C875	DBSNP		32	298	1.43216	2	331	119	0.89511	1	516	129	0.978785	1
31	chr1	33960630	33960630	A	G	exonic	ZSCAN20		nonsynonymous SNV	ZSCAN20:NM_145238:exon8:c.A2686G:p.I	NOVEL		95	70	0.921411	1	130	91	0.982504	1	233	75	0.972836	1
31	chr9	71661369	71661369	G	A	exonic	FXN		synonymous SNV	FXN:NM_000144:exon2:c.G234A:p.L78L,FX	NOVEL		43	35	0.974569	1	54	36	0.954433	1	135	43	0.965111	1
31	chr2	48809219	48809219	C	A	exonic	STON1;STON1-GT		nonsynonymous SNV	STON1-GTF2A1L:NM_001198594:exon1:c	COSMIC	ID=COSM679221	77	50	0.977899	1	178	111	0.916454	1	353	111	0.955727	1
31	chr17	33466298	33466298	C	T	exonic	NLE1		nonsynonymous SNV	NLE1:NM_018096:exon4:c.G416A:p.R139H	DBSNP		58	34	1.05387	1	57	42	1.01228	1	115	36	0.952477	1
31	chr7	87782590	87782590	A	G	exonic	ADAM22		nonsynonymous SNV	ADAM22:NM_001324417:exon21:c.A1733	NOVEL		132	114	1.00649	1	196	110	0.857742	1	376	117	0.94813	1
31	chr10	108923737	108923737	T	C	exonic	SORCS1		nonsynonymous SNV	SORCS1:NM_001013031:exon1:c.A548G:p	NOVEL		342	0	0	1	185	108	0.879511	1	405	126	0.947993	1
31	chr18	44774980	44774980	C	T	exonic	SKOR2		nonsynonymous SNV	SKOR2:NM_001037802:exon2:c.G575A:p.	NOVEL		273	0	0	1	258	173	0.957755	1	534	166	0.947412	1
31	chr3	151105696	151105696	C	T	exonic	MED12L		synonymous SNV	MED12L:NM_053002:exon35:c.C5082T:p.V	NOVEL		70	54	0.945826	1	53	55	1.21513	1	132	41	0.946819	1
31	chr12	21981983	21981983	C	T	exonic	ABCC9		nonsynonymous SNV	ABCC9:NM_005691:exon29:c.G3578A:p.R1	NOVEL		34	46	1.24884	1	62	39	0.921358	1	132	41	0.946819	1
31	chr1	103548363	103548363	G	A	exonic	COL11A1		nonsynonymous SNV	COL11A1:NM_001190709:exon2:c.C272T:p	NOVEL		26	25	1.06466	1	98	64	0.94265	1	150	46	0.937628	1
31	chr12	91396228	91396228	C	A	exonic	EPYC		nonsynonymous SNV	EPYC:NM_004950:exon2:c.G115T:p.D39Y	NOVEL		186	1	0.0116144	1	120	95	1.05432	1	235	71	0.926971	1
31	chr14	73980912	73980912	G	A	exonic	HEATR4		nonsynonymous SNV	HEATR4:NM_203309:exon5:c.C1237T:p.R4	DBSNP		56	0	0	1	53	26	0.785293	1	98	29	0.91227	1
31	chr10	52005186	52005186	G	C	exonic	ASAH2		synonymous SNV	ASAH2:NM_001143974:exon2:c.C156G:p.T	NOVEL		39	0	0	1	38	33	1.10902	1	72	21	0.902122	1
31	chr1	115258747	115258747	C	T	exonic	NRAS		nonsynonymous SNV	NRAS:NM_002524:exon2:c.G35A:p.G12D	DBSNP+COSMIC	ID=COSM564;OC	78	24	0.511034	1	44	38	1.10575	1	151	44	0.901461	1
31	chr5	176079874	176079874	A	G	exonic	TSPAN17		nonsynonymous SNV	TSPAN17:NM_001366492:exon2:c.A218G:	NOVEL		133	82	0.828351	1	90	45	0.795361	1	206	60	0.901153	1
31	chr21	31587850	31587850	C	G	exonic	CLDN8		nonsynonymous SNV	CLDN8:NM_199328:exon1:c.G394C:p.V132	NOVEL		151	0	0	1	73	55	1.02527	1	196	57	0.900085	1
31	chr19	11150144	11150144	G	A	exonic	SMARCA4		nonsynonymous SNV	SMARCA4:NM_001128849:exon30:c.G418	NOVEL		168	0	0	1	115	48	0.997128	1	174	38	0.89535	1

31	chr4	164247228	164247228	G	T	exonic	NPY1R		nonsynonymous SNV	NPY1R:NM_000909:exon2:c.C479A:p.A160	NOVEL		204	153	0.930813	1	255	196	1.03697	1	558	161	0.894594	1
31	chr2	74668874	74668874	G	A	exonic	RTKN		nonsynonymous SNV	RTKN:NM_001015055:exon1:c.C70T:p.R24	NOVEL		105	0	0	1	56	0	0	1	73	21	0.892525	1
31	chr7	94228152	94228152	T	G	exonic	SGCE		nonsynonymous SNV	SGCE:NM_001362809:exon7:c.A1038C:p.E	NOVEL		101	96	1.05839	1	125	82	0.945211	1	279	79	0.881603	1
31	chr19	19760642	19760642	C	A	exonic	ATP13A1		nonsynonymous SNV	ATP13A1:NM_020410:exon18:c.G2443T:p.	NOVEL		103	52	1.06412	1	111	35	0.811442	1	202	43	0.876693	1
31	chr1	215777490	215777490	G	A	exonic	KCTD3		stopgain	KCTD3:NM_001319294:exon13:c.G1155A:	NOVEL		16	122	0.960041	2	28	81	1.03002	1	159	65	0.869117	1
31	chr2	85011885	85011885	C	T	exonic	DNAH6		nonsynonymous SNV	DNAH6:NM_001370:exon66:c.C10904T:p.S	COSMIC	ID=COSM445858	79	0	0	1	95	62	0.942274	1	206	57	0.865861	1
31	chr16	88598602	88598602	C	A	exonic	ZFPM1		nonsynonymous SNV	ZFPM1:NM_153813:exon7:c.C905A:p.P302	NOVEL		165	0	0	1	106	0	0	1	117	32	0.858011	1
31	chr3	121414039	121414039	G	A	exonic	GOLGB1		synonymous SNV	GOLGB1:NM_001256488:exon12:c.C5091T	NOVEL		74	71	1.06348	1	160	97	0.900584	1	336	91	0.851417	1
31	chr16	89351359	89351359	C	T	exonic	ANKRD11		nonsynonymous SNV	ANKRD11:NM_001256183:exon9:c.G1591A	DBSNP+COSMIC	ID=COSM660028	23	21	1.03659	1	28	8	0.53024	1	71	19	0.843413	1
31	chr16	67201815	67201815	G	A	exonic	HSF4		synonymous SNV	HSF4:NM_001040667:exon11:c.G1047A:p.	NOVEL		135	0	0	1	107	0	0	1	135	36	0.841076	1
31	chr7	141740634	141740634	C	A	exonic	MGAM		nonsynonymous SNV	MGAM:NM_001365693:exon21:c.C2486A:	NOVEL		81	0	0	1	63	43	0.967939	1	176	44	0.799022	1
31	chr16	56667709	56667709	C	T	exonic	MT1M		synonymous SNV	MT1M:NM_176870:exon3:c.C141T:p.G47G	NOVEL		88	0	0	1	126	0	0	1	167	41	0.787498	1
31	chr11	69588878	69588878	G	A	exonic	FGF4		nonsynonymous SNV	FGF4:NM_002007:exon2:c.C358T:p.P120S	NOVEL		30	40	1.24108	1	38	24	0.923645	1	75	18	0.773248	1
31	chr7	150417150	150417150	G	A	exonic	GIMAP1;GIMAP1-		nonsynonymous SNV	GIMAP1-GIMAP5:NM_001199577:exon3:c	DBSNP		85	0	0	1	99	0	0	1	144	34	0.763111	1
31	chr2	37455965	37455965	T	C	exonic	CEBPZ		nonsynonymous SNV	CEBPZ:NM_005760:exon2:c.A371G:p.N124	DBSNP		65	0	0	1	176	0	0	1	233	49	0.694186	1
31	chr5	80639668	80639668	G	T	exonic	ACOT12		nonsynonymous SNV	ACOT12:NM_130767:exon10:c.C1013A:p.P	NOVEL		78	0	0	1	115	0	0	1	169	35	0.685436	1
31	chr15	41193192	41193192	G	A	exonic	VPS18		nonsynonymous SNV	VPS18:NM_020857:exon4:c.G2176A:p.A72	NOVEL		220	0	0	1	176	0	0	1	202	32	0.669255	1
31	chr6	114270360	114270360	T	A	exonic	HDAC2		nonsynonymous SNV	HDAC2:NM_001527:exon7:c.A704T:p.D235	NOVEL		107	0	0	1	125	0	0	1	184	36	0.653746	1
31	chr4	39448729	39448729	C	T	exonic	KLB		stopgain	KLB:NM_175737:exon4:c.C2383T:p.R795X	NOVEL		154	0	0	1	223	0	0	1	408	65	0.653095	1
31	chr12	113618816	113618816	G	C	exonic	DDX54		synonymous SNV	DDX54:NM_001111322:exon2:c.C222G:p.T	NOVEL		110	0	0	1	92	0	0	1	130	24	0.622615	1
31	chr19	4283126	4283126	C	T	exonic	SHD		nonsynonymous SNV	SHD:NM_020209:exon3:c.C479T:p.P160L	NOVEL		264	0	0	1	206	0	0	1	307	41	0.588505	1
31	chr20	24973248	24973248	G	T	exonic	APMAP		nonsynonymous SNV	APMAP:NM_020531:exon1:c.C77A:p.P26Q	NOVEL		92	0	0	1	53	0	0	1	81	11	0.477676	1
31	chr9	137776589	137776589	C	G	exonic	FCN2		nonsynonymous SNV	FCN2:NM_015837:exon3:c.C158G:p.A53G,	NOVEL		132	0	0	1	113	0	0	1	166	22	0.467513	1
31	chr2	1805500	1805500	T	A	exonic	MYT1L		nonsynonymous SNV	MYT1L:NM_001303052:exon23:c.A3244T:	NOVEL		133	0	0	1	195	0	0	1	265	14	0.200472	1
31	chr10	97443293	97443293	C	T	exonic	TCTN3		nonsynonymous SNV	TCTN3:NM_001143973:exon6:c.G745A:p.D	NOVEL		150	0	0	1	190	0	0	1	234	10	0.163734	1
31	chr1	231488770	231488770	C	T	exonic	SPRTN		nonsynonymous SNV	SPRTN:NM_032018:exon5:c.C1133T:p.S37	NOVEL		262	0	0	1	500	0	0	1	726	18	0.145043	1
31	chr10	7788587	7788587	C	T	exonic	ITIH2		synonymous SNV	ITIH2:NM_002216:exon20:c.C2613T:p.I871	COSMIC	ID=COSM457383	132	0	0	1	186	0	0	1	275	10	0.140179	1
31	chr10	70509379	70509379	C	T	exonic	CCAR1		nonsynonymous SNV	CCAR1:NM_001282959:exon9:c.C1010T:p.	NOVEL		192	0	0	1	254	0	0	1	393	13	0.127922	1
31	chr1	109650648	109650648	A	T	exonic	C1orf194		stopgain	C1orf194:NM_001122961:exon2:c.T57A:p.	DBSNP+COSMIC	ID=COSM380116	32	5	0.293499	1	48	3	0.140358	1	70	1	0.0562692	1
31	chr2	71825857	71825857	G	C	exonic	DYSF		synonymous SNV	DYSF:NM_001130976:exon32:c.G3642C:p.	NOVEL		137	44	0.603812	1	156	0	0	1	248	1	0.0160446	1
31	chr5	41143046	41143046	A	G	exonic	C6		nonsynonymous SNV	C6:NM_000065:exon18:c.T2686C:p.Y896H,	NOVEL		106	37	0.561959	1	199	0	0	1	346	1	0.0115133	1
31	chr19	897462	897462	G	A	exonic	R3HDM4		nonsynonymous SNV	R3HDM4:NM_138774:exon8:c.C782T:p.S26	NOVEL		78	0	0	1	66	18	0.72533	1	130	0	0	1
31	chr10	31809540	31809540	G	A	exonic	ZEB1		nonsynonymous SNV	ZEB1:NM_001323674:exon5:c.G1055A:p.R	NOVEL		188	0	0	1	199	86	0.720011	1	476	0	0	1
31	chr11	58949512	58949512	C	T	exonic	DTX4		nonsynonymous SNV	DTX4:NM_001300727:exon2:c.C194T:p.A6	DBSNP		201	0	0	1	105	43	0.693254	1	241	0	0	1
31	chr1	120277376	120277376	G	A	exonic	PHGDH		synonymous SNV	PHGDH:NM_006623:exon6:c.G630A:p.L210	NOVEL		199	0	NA	1	154	58	0.652796	1	347	0	0	1
31	chr10	134622236	134622236	C	T	exonic	CFAP46		nonsynonymous SNV	CFAP46:NM_001200049:exon58:c.G7837A	NOVEL		63	0	0	1	59	22	0.648072	1	134	0	0	1
31	chr4	40146297	40146297	G	A	exonic	N4BP2		nonsynonymous SNV	N4BP2:NM_018177:exon16:c.G5020A:p.A	NOVEL		240	0	0	1	211	78	0.643994	1	577	0	0	1
31	chr14	65199146	65199146	C	T	exonic	PLEKHG3		nonsynonymous SNV	PLEKHG3:NM_001308147:exon11:c.C1214	NOVEL		169	0	0	1	144	49	0.605793	1	313	0	0	1
31	chr14	55203972	55203972	A	G	exonic	SAMD4A		nonsynonymous SNV	SAMD4A:NM_015589:exon3:c.A946G:p.N	NOVEL		118	0	0	1	104	35	0.600812	1	204	0	0	1
31	chr9	137630345	137630345	G	T	exonic	COL5A1		nonsynonymous SNV	COL5A1:NM_000093:exon10:c.G1415T:p.G	COSMIC	ID=COSM618259	223	0	0	1	159	53	0.596521	1	345	0	0	1
31	chr17	46050952	46050952	G	C	exonic	CDK5RAP3		nonsynonymous SNV	CDK5RAP3:NM_001278197:exon3:c.G195C	DBSNP+COSMIC	ID=COSM712292	205	0	0	1	163	22	0.402525	1	311	0	0	1
31	chr6	108677894	108677894	G	A	exonic	AFG1L		nonsynonymous SNV	AFG1L:NM_001323005:exon5:c.G518A:p.R	NOVEL		199	0	0	1	224	24	0.230911	1	387	0	0	1
31	chr2	165586510	165586510	T	G	exonic	COBLL1		nonsynonymous SNV	COBLL1:NM_001278461:exon3:c.A346C:p.	NOVEL		36	0	0	1	51	8	0.187943	1	149	0	0	1
31	chr3	53269090	53269090	C	T	exonic	TKT		nonsynonymous SNV	TKT:NM_001064:exon5:c.G538A:p.A180T,	NOVEL		192	0	0	1	127	10	0.174167	1	240	0	0	1
31	chr1	205589475	205589475	C	T	exonic	ELK4		synonymous SNV	ELK4:NM_001973:exon3:c.G699A:p.L233L,	DBSNP		154	0	0	1	247	13	0.169244	1	395	0	0	1
31	chr17	71084898	71084898	G	A	exonic	SLC39A11		synonymous SNV	SLC39A11:NM_001159770:exon2:c.C6T:p.	DBSNP+COSMIC	ID=COSM536424	112	0	0	1	153	8	0.168192	1	244	0	0	1
31	chr19	10402350	10402350	G	C	exonic	ICAM5		nonsynonymous SNV	ICAM5:NM_003259:exon3:c.G538C:p.V180	NOVEL		125	0	0	1	173	9	0.167444	1	297	0	0	1
31	chr17	48265291	48265291	T	G	exonic	COL1A1		nonsynonymous SNV	COL1A1:NM_000088:exon45:c.A3315C:p.R	NOVEL		279	0	0	1	239	11	0.148934	1	382	0	0	1
31	chr4	89034516	89034516	C	T	exonic	ABCG2		nonsynonymous SNV	ABCG2:NM_001257386:exon9:c.G1133A:p	NOVEL		299	0	0	1	341	20	0.132193	1	600	0	0	1
31	chr6	137322708	137322708	T	C	exonic	IL20RA		nonsynonymous SNV	IL20RA:NM_001278723:exon6:c.A1316G:p	NOVEL		89	0	0	1	142	8	0.127258	1	312	0	0	1
31	chr2	139316758	139316758	C	G	exonic	SPOPL		nonsynonymous SNV	SPOPL:NM_001001664:exon6:c.C647G:p.S	DBSNP		82	0	0	1	131	7	0.121033	1	243	0	0	1
31	chr1	43770688	43770688	G	T	exonic	TIE1		synonymous SNV	TIE1:NM_001253357:exon2:c.G90T:p.G30	NOVEL		112	0	0	1	95	5	0.119304	1	185	0	0	1
31	chr20	62839371	62839371	T	A	exonic	MYT1		nonsynonymous SNV	MYT1:NM_004535:exon7:c.T822A:p.D274E	COSMIC	ID=COSM429449	107	8	0.151088	1	83	1	0.0284057	1	122	0	0	1
31	chr13	72063139	72063139	G	T	exonic	DACH1		nonsynonymous SNV	DACH1:NM_004392:exon4:c.C1106A:p.T36	COSMIC	ID=COSM498942	47	27	0.792449	1	41	0	0	1	126	0	0	1
31	chr3	180686002	180686002	T	C	exonic	FXR1		synonymous SNV	FXR1:NM_001013438:exon14:c.T1362C:p.	NOVEL		57	24	0.643525	1	112	0	0	1	205	0	0	1
31	chr17	169268	169268	G	T	exonic	RPH3AL		stopgain	RPH3AL:NM_001190411:exon4:c.C294A:p.	NOVEL		175	50	0.633698	1	77	0	0	1	148	0	0	1
31	chr3	180686001	180686001	G	T	exonic	FXR1		nonsynonymous SNV	FXR1:NM_001013438:exon14:c.G1361T:p.	NOVEL		59	24	0.628018	1	111	0	0	1	206	0	0	1
31	chr1	53442386	53442386	G	A	exonic	SCP2		nonsynonymous SNV	SCP2:NM_001007098:exon7:c.G487A:p.D1	NOVEL		39	15	0.603305	1	135	0	0	1	192	0	0	1
31	chr1	113212641	113212641	A	G	exonic	CAPZA1		nonsynonymous SNV	CAPZA1:NM_006135:exon10:c.A748G:p.M	NOVEL		163	61	0.591454	1	288	0	0	1	490	0	0	1
31	chr19	51051931	51051931	G	A	exonic	LRRC4B		synonymous SNV	LRRC4B:NM_001080457:exon2:c.C165T:p.	NOVEL		60	13	0.564858	1	56	0	0	1	104	0	0	1
31	chr2	113405003	113405003	G	A	exonic	SLC20A1		nonsynonymous SNV	SLC20A1:NM_005415:exon3:c.G437A:p.G1	NOVEL		124	43	0.559231	1	260	0	0	1	408	0	0	1
31	chr7	47917193	47917193	T	G	exonic	PKD1L1		nonsynonymous SNV	PKD1L1:NM_138295:exon22:c.A3557C:p.Y	NOVEL		86	24	0.473868	1	131	0	0	1	213	0	0	1
31	chr5	101593000	101593000	G	A	exonic	SLCO4C1		nonsynonymous SNV	SLCO4C1:NM_180991:exon6:c.C1288T:p.P	COSMIC	ID=COSM391840	119	23	0.351786	1	158	0	0	1	294	0	0	1
31	chr17	78933889	78933889	G	C	exonic	RPTOR		synonymous SNV	RPTOR:NM_001163034:exon26:c.G3015C:	DBSNP		64	9	0.351572	1	128	0	0	1	175	0	0	1
31	chr6	147830197	147830197	G	A	exonic	SAMD5		nonsynonymous SNV	SAMD5:NM_001030060:exon1:c.G133A:p.	COSMIC	ID=COSM362135	91	21	0.346296	1	194	0	0	1	334	0	0	1
31	chr1	67518474	67518474	C	T	exonic	SLC35D1		nonsynonymous SNV	SLC35D1:NM_015139:exon3:c.G304A:p.D1	NOVEL		209	36	0.319136	1	246	0	0	1	464	0	0	1
31	chr2	73449029	73449029	G	A	exonic	SMYD5		nonsynonymous SNV	SMYD5:NM_006062:exon6:c.G613A:p.V20	NOVEL		262	38	0.314623	1	239	0	0	1	377	0	0	1
31	chr3	50339745	50339745	G	A	exonic	HYAL1		nonsynonymous SNV	HYAL1:NM_033159:exon2:c.C643T:p.P215	NOVEL		110	18	0.305423	1	139	0	0	1	229	0	0	1
31	chr2	179598041	179598041	C	T	exonic	TTN		nonsynonymous SNV	TTN:NM_133378:exon51:c.G12247A:p.D40	DBSNP+COSMIC	ID=COSM270714	41	13	0.282123	1	120	0	0	1	272	0	0	1
31	chr19	50093210	50093210	C	T	exonic	PRRG2		nonsynonymous SNV	PRRG2:NM_000951:exon6:c.C491T:p.P164	NOVEL		162	15	0.268805	1	48	0	0	1	75	0	0	1
31	chr5	13721239	13721239	G	A	exonic	DNAH5		nonsynonymous SNV	DNAH5:NM_001369:exon71:c.C12149T:p.S	DBSNP		93	12	0.248217	1	192	0	0	1	315	0	0	1
31	chr11	123900914	123900914	G	A	exonic	OR10G8		synonymous SNV	OR10G8:NM_001004464:exon1:c.G585A:p	DBSNP		320	41	0.24667	1	292	0	0	1	558	0	0	1
31	chr19	9061637	9061637	G	A	exonic	MUC16		nonsynonymous SNV	MUC16:NM_024690:exon3:c.C25809T:p.D	NOVEL		172	14	0.238745	1	286	0	0	1	412	0	0	1
31	chr2	152222587	152222587	G	A	exonic	TNFAIP6		nonsynonymous SNV	TNFAIP6:NM_007115:exon3:c.G250A:p.A8	NOVEL		152	17	0.218475	1	186	0	0	1	301	0	0	1
31	chr22	50879304	50879304	C	G	exonic	PPP6R2		nonsynonymous SNV	PPP6R2:NM_001242899:exon21:c.C2350G	NOVEL		39	4	0.202037	1	78	0	0	1	132	0	0	1
31	chr19	39860624	39860624	G	A	exonic	SAMD4B		nonsynonymous SNV	SAMD4B:NM_001303614:exon3:c.G526A:	NOVEL		239	16	0.199021	1	205	0	0	1	314	0	0	1
31	chr7	149461873	149461873	G	A	exonic	ZNF467		nonsynonymous SNV	ZNF467:NM_207336:exon5:c.C1718T:p.P5	DBSNP		61	5	0.164538	1	138	0	0	1	219	0	0	1
31	chr1	7880652	7880652	C	T	exonic	PER3		stopgain	PER3:NM_001289861:exon16:c.C1909T:p.	NOVEL		137	8	0.119829	1	122	0	0	1	196	0	0	1
31	chr2	209150516	209150516	A	G	exonic	PIKFYVE		nonsynonymous SNV	PIKFYVE:NM_152671:exon4:c.A389G:p.N1	NOVEL		154	8	0.107254	1	252	0	0	1	485	0	0	1
31	chr1	29481310	29481310	T	G	exonic	SRSF4		nonsynonymous SNV	SRSF4:NM_005626:exon4:c.A476C:p.K159	NOVEL		199	10	0.0724629	1	354	0	0	1	585	0	0	1
31	chr6	160197289	160197289	C	A	exonic	ACAT2		nonsynonymous SNV	ACAT2:NM_001303253:exon6:c.C829A:p.P	NOVEL		209	8	0.0680889	1	273	0	0	1	478	0	0	1
31	chr13	48934151	48934151	A	T	splicing	RB1	NM_000321:exon7:c.6			COSMIC	ID=COSM395723	23	0	0	1	18	36	0.924055	1	88	44	0.998371	1
31	chr2	40402399	40402399	A	T	splicing	SLC8A1	NM_021097:exon3:c.1			NOVEL		80	0	0	1	150	0	0	1	163	38	0.755295	1
31	chr6	100896021	100896021	C	T	splicing	SIM1	NM_005068:exon7:c.8			NOVEL		55	0	0	1	38	26	0.969346	1	107	19	0.602438	1
31	chr5	38960498	38960498	A	T	splicing	RICTOR	NM_152756:exon20:c.			NOVEL		234	33	0.268437	1	350	0	0	1	619	1	0.00644373	1
31	chr4	1107142	1107142	A	T	splicing	RNF212	NM_001366918:exon1			NOVEL		150	0	0	1	71	8	0.241629	1	105	0	0	1
32	chr5	140565	140565	C	T	exonic	PLEKHG4B		nonsynonymous SNV	PLEKHG4B:NM_052909:exon3:c.C1211T:p.	NOVEL		192	85	0.938857	1	198	68	NA	1				
32	chr7	140453136	140453136	A	T	exonic	BRAF		nonsynonymous SNV	BRAF:NM_001354609:exon15:c.T1799A:p.	DBSNP+COSMIC	ID=COSM476;OC	91	43	0.981314	1	31	49	1.25956	1				
32	chr9	130635040	130635040	C	T	exonic	AK1		nonsynonymous SNV	AK1:NM_001318122:exon3:c.G184A:p.E62	DBSNP+COSMIC	ID=COSM587558	136	64	0.979062	1	133	81	1.1237	1				
32	chr2	171573774	171573774	G	A	exonic	SP5		nonsynonymous SNV	SP5:NM_001003845:exon2:c.G1057A:p.E3	NOVEL		110	101	1.01281	1	106	123	1.10454	1				
32	chr17	7246726	7246726	C	T	exonic	ACAP1		nonsynonymous SNV	ACAP1:NM_014716:exon6:c.C373T:p.R125	DBSNP+COSMIC	ID=COSM406934	29	25	0.979574	1	33	38	1.10062	1				
32	chr1	982300	982300	G	T	exonic	AGRN		nonsynonymous SNV	AGRN:NM_198576:exon19:c.G3351T:p.K1	NOVEL		55	30	0.746781	1	54	62	1.09912	1				
32	chr16	2578360	2578360	G	C	exonic	AMDHD2		nonsynonymous SNV	AMDHD2:NM_001145815:exon7:c.G841C:	NOVEL		57	55	1.03905	1	53	59	1.0833	1				
32	chr14	50101482	50101482	C	T	exonic	DNAAF2		nonsynonymous SNV	DNAAF2:NM_001083908:exon1:c.G386A:p	NOVEL		103	80	0.924975	1	78	86	1.07837	1				
32	chr12	7926424	7926424	T	C	exonic	NANOGNB		nonsynonymous SNV	NANOGNB:NM_001145465:exon4:c.T544	NOVEL		33	33	1.05794	1	37	40	1.06827	1				
32	chr1	1262695	1262695	G	A	exonic	CPTP		nonsynonymous SNV	CPTP:NM_001029885:exon3:c.G197A:p.R6	DBSNP		96	111	1.1346	1	119	127	1.06165	1				
32	chr19	49934391	49934391	C	T	exonic	SLC17A7		nonsynonymous SNV	SLC17A7:NM_020309:exon11:c.G1270A:p.	NOVEL		168	110	0.999839	1	207	113	1.04836	1				
32	chr5	22078769	22078769	C	G	exonic	CDH12		nonsynonymous SNV	CDH12:NM_001317227:exon2:c.G17C:p.C6	COSMIC	ID=COSM337384	177	43	0.598007	1	110	60	1.04781	1				
32	chr3	130150336	130150336	T	G	exonic	COL6A5		nonsynonymous SNV	COL6A5:NM_001278298:exon33:c.T5276G	NOVEL		217	173	0.938582	1	210	217	1.04507	1				
32	chr14	39644502	39644502	A	G	exonic	PNN		nonsynonymous SNV	PNN:NM_002687:exon1:c.A19G:p.T7A	NOVEL		37	17	0.66611	1	30	31	1.04507	1				
32	chr2	51254915	51254915	G	A	exonic	NRXN1		nonsynonymous SNV	NRXN1:NM_001135659:exon2:c.C497T:p.A	DBSNP+COSMIC	ID=COSM470828	150	160	1.09207	1	151	156	1.04496	1				
32	chr10	62648445	62648445	G	A	exonic	RHOBTB1		synonymous SNV	RHOBTB1:NM_001350910:exon5:c.C621T:	DBSNP		229	210	1.01215	1	197	201	1.03855	1				
32	chr2	120831687	120831687	T	C	exonic	EPB41L5		nonsynonymous SNV	EPB41L5:NM_001184937:exon5:c.T340C:p	NOVEL		83	79	1.03182	1	88	88	1.02821	1				
32	chr6	76022116	76022116	T	A	exonic	FILIP1		synonymous SNV	FILIP1:NM_001300866:exon5:c.A3432T:p.	NOVEL		174	145	0.961763	1	147	147	1.02821	1				
32	chrX	44120484	44120484	G	A	exonic	EFHC2		nonsynonymous SNV	EFHC2:NM_025184:exon4:c.C443T:p.T148	NOVEL		54	68	0.589671	1	0	110	1.02821	1				
32	chr20	5921975	5921975	G	A	exonic	TRMT6		nonsynonymous SNV	TRMT6:NM_001281467:exon8:c.C619T:p.R	DBSNP		213	55	0.434229	1	130	130	1.02821	1				
32	chr8	17417945	17417945	G	T	exonic	SLC7A2		synonymous SNV	SLC7A2:NM_001008539:exon9:c.G1407T:	DBSNP		33	72	1.04315	1	3	78	1.0173	1				
32	chrX	119387981	119387981	G	A	exonic	ZBTB33		synonymous SNV	ZBTB33:NM_006777:exon2:c.G711A:p.S23	DBSNP+COSMIC	ID=COSM722192	22	221	0.962159	1	3	248	1.01592	1				
32	chr16	68289271	68289271	C	T	exonic	PLA2G15		nonsynonymous SNV	PLA2G15:NM_001363551:exon4:c.C490T:p	NOVEL		30	36	0.608662	1	2	49	1.015	1				
32	chr1	45293543	45293543	G	A	exonic	PTCH2		nonsynonymous SNV	PTCH2:NM_001166292:exon14:c.C2030T:p	COSMIC	ID=COSM40513;	100	76	0.913675	1	109	106	1.01387	1				
32	chr3	4669453	4669453	T	C	exonic	ITPR1		nonsynonymous SNV	ITPR1:NM_001099952:exon5:c.T170C:p.L5	NOVEL		438	0	0	1	208	202	1.01317	1				
32	chr16	683977	683977	G	C	exonic	WFIKKN1		nonsynonymous SNV	WFIKKN1:NM_053284:exon2:c.G1567C:p.	NOVEL		67	51	0.91449	1	48	46	1.00634	1				
32	chr7	103557557	103557557	G	T	exonic	RELN		stopgain	RELN:NM_005045:exon2:c.C302A:p.S101X	NOVEL		259	436	0.959215	2	13	468	1.00042	2				
32	chr12	53702243	53702243	A	G	exonic	AAAS		nonsynonymous SNV	AAAS:NM_001173466:exon11:c.T1058C:p.	NOVEL		252	0	0	1	119	112	0.997054	1				
32	chr7	150878438	150878438	G	T	exonic	ASB10		nonsynonymous SNV	ASB10:NM_001142459:exon3:c.C692A:p.A	NOVEL		106	207	1.01121	2	9	237	0.990595	2				
32	chr11	34909855	34909855	G	A	exonic	APIP		nonsynonymous SNV	APIP:NM_015957:exon5:c.C446T:p.S149F	DBSNP		70	131	0.996528	2	65	128	0.984472	2				
32	chr9	96031020	96031020	C	G	exonic	WNK2		nonsynonymous SNV	WNK2:NM_001282394:exon18:c.C4025G:	DBSNP		68	118	0.970508	2	64	125	0.981745	2				
32	chr18	32920434	32920434	G	T	exonic	ZNF24		stopgain	ZNF24:NM_001308123:exon2:c.C181T:p.Q	NOVEL		96	86	0.999811	1	104	95	0.98171	1				
32	chrX	153628150	153628150	A	G	exonic	RPL10		nonsynonymous SNV	RPL10:NM_001256580:exon4:c.A89G:p.E3	NOVEL		23	4	0.156732	1	2	25	0.952049	1				
32	chr11	113934171	113934171	C	T	exonic	ZBTB16		nonsynonymous SNV	ZBTB16:NM_001354752:exon1:c.C149T:p.	NOVEL		338	146	0.922928	1	340	160	0.950015	1				
32	chr21	43274768	43274768	G	T	exonic	PRDM15		nonsynonymous SNV	PRDM15:NM_001040424:exon5:c.C556A:p	DBSNP		265	49	0.477449	1	242	113	0.944998	1				
32	chr4	70513339	70513339	G	A	exonic	UGT2A1		synonymous SNV	UGT2A1:NM_001301239:exon1:c.C24T:p.F	NOVEL		295	0	0	1	152	129	0.944053	1				
32	chr1	222803271	222803271	G	A	exonic	MIA3		synonymous SNV	MIA3:NM_001324062:exon4:c.G2709A:p.S	DBSNP		157	114	1.06296	1	245	114	0.942738	1				
32	chr4	106580295	106580295	C	T	exonic	ARHGEF38		nonsynonymous SNV	ARHGEF38:NM_001242729:exon10:c.C131	DBSNP		105	91	0.982373	1	100	84	0.938803	1				
32	chr7	48319435	48319435	C	T	exonic	ABCA13		nonsynonymous SNV	ABCA13:NM_152701:exon18:c.C8644T:p.P	DBSNP		367	7	0.0572362	1	256	117	0.931231	1				
32	chr5	1219133	1219133	C	T	exonic	SLC6A19		nonsynonymous SNV	SLC6A19:NM_001003841:exon9:c.C1289T:	COSMIC	ID=COSM560493	116	196	0.961018	2	109	180	0.924539	2				
32	chr1	176563812	176563812	C	T	exonic	PAPPA2		nonsynonymous SNV	PAPPA2:NM_020318:exon3:c.C1072T:p.R3	DBSNP+COSMIC	ID=COSM565292	233	59	0.510566	1	260	116	0.915906	1				
32	chr17	75190770	75190770	C	T	exonic	SEC14L1		synonymous SNV	SEC14L1:NM_001144001:exon5:c.C384T:p	DBSNP		50	62	1.17129	1	88	67	0.888906	1				
32	chr3	179069741	179069741	G	A	exonic	MFN1		nonsynonymous SNV	MFN1:NM_033540:exon3:c.G166A:p.E56K	DBSNP		219	2	0.0327507	1	191	57	0.88618	1				
32	chr1	228333761	228333761	C	T	exonic	GUK1		synonymous SNV	GUK1:NM_001159390:exon3:c.C210T:p.N7	DBSNP+COSMIC	ID=COSM667717	104	68	0.998994	1	173	72	0.872463	1				
32	chr3	119334945	119334945	G	A	exonic	PLA1A		nonsynonymous SNV	PLA1A:NM_001206961:exon5:c.G232A:p.A	DBSNP+COSMIC	ID=COSM312008	144	14	0.187483	1	79	58	0.870603	1				
32	chr7	141778765	141778765	G	A	exonic	MGAM		nonsynonymous SNV	MGAM:NM_001365693:exon49:c.G5804A	DBSNP		490	223	0.956444	1	320	230	0.859959	1				
32	chr15	77474124	77474124	G	A	exonic	PEAK1		nonsynonymous SNV	PEAK1:NM_024776:exon5:c.C145T:p.H49Y	NOVEL		222	41	0.476967	1	209	85	0.858326	1				
32	chr7	137271862	137271862	C	A	exonic	DGKI		nonsynonymous SNV	DGKI:NM_001321708:exon13:c.G1406T:p.	NOVEL		447	7	0.0471505	1	208	149	0.858284	1				
32	chr12	52214674	52214674	G	T	exonic	FIGNL2		synonymous SNV	FIGNL2:NM_001013690:exon2:c.C1524A:p	NOVEL		34	0	0	1	24	16	0.82257	1				
32	chr11	63991806	63991806	C	T	exonic	TRPT1		synonymous SNV	TRPT1:NM_001160393:exon5:c.G510A:p.R	NOVEL		163	0	0	1	145	34	0.58055	1				
32	chr1	67721529	67721529	A	G	exonic	IL23R		synonymous SNV	IL23R:NM_144701:exon10:c.A1158G:p.R3	NOVEL		94	0	0	1	84	25	0.471657	1				
32	chr7	39379476	39379476	G	C	exonic	POU6F2		nonsynonymous SNV	POU6F2:NM_001370959:exon5:c.G834C:p	NOVEL		161	0	0	1	132	22	0.424114	1				
32	chr19	38828248	38828248	C	A	exonic	CATSPERG		nonsynonymous SNV	CATSPERG:NM_001330496:exon3:c.C286A	NOVEL		263	1	0.00957147	1	276	38	0.359281	1				
32	chr3	130318630	130318630	G	T	exonic	COL6A6		synonymous SNV	COL6A6:NM_001102608:exon19:c.G4629T	NOVEL		188	0	0	1	137	29	0.359255	1				
32	chr19	38828249	38828249	C	T	exonic	CATSPERG		nonsynonymous SNV	CATSPERG:NM_001330496:exon3:c.C287T	NOVEL		266	0	0	1	277	38	0.358141	1				
32	chr5	140744081	140744081	C	T	exonic	PCDHGA5		nonsynonymous SNV	PCDHGA5:NM_018918:exon1:c.C184T:p.R6	NOVEL		158	66	0.90148	1	140	19	0.271565	1				
32	chr3	98299552	98299552	C	T	exonic	CPOX		nonsynonymous SNV	CPOX:NM_000097:exon7:c.G1340A:p.R447	DBSNP+COSMIC	ID=COSM142600	175	0	0	1	181	24	0.240752	1				
32	chr5	170236731	170236731	C	G	exonic	GABRP		nonsynonymous SNV	GABRP:NM_014211:exon9:c.C992G:p.S331	NOVEL		125	55	0.934868	1	145	17	0.23848	1				
32	chr21	27284244	27284244	G	T	exonic	APP		stopgain	APP:NM_001136129:exon11:c.C1325A:p.S	NOVEL		422	0	0	1	369	30	0.223218	1				
32	chr19	50826844	50826844	C	A	exonic	KCNC3		nonsynonymous SNV	KCNC3:NM_004977:exon2:c.G1366T:p.A45	NOVEL		437	0	0	1	491	37	0.208041	1				
32	chr1	210560905	210560905	G	T	exonic	HHAT		nonsynonymous SNV	HHAT:NM_001170587:exon3:c.G256T:p.A8	NOVEL		189	0	0	1	191	12	0.175495	1				
32	chr17	32964978	32964978	C	T	exonic	TMEM132E		synonymous SNV	TMEM132E:NM_001304438:exon9:c.C295	NOVEL		57	0	0	1	58	5	0.163208	1				
32	chr22	50719897	50719897	C	T	exonic	PLXNB2		nonsynonymous SNV	PLXNB2:NM_012401:exon22:c.G3554A:p.G	NOVEL		196	0	0	1	189	16	0.160501	1				
32	chr4	70505302	70505302	A	G	exonic	UGT2A2		nonsynonymous SNV	UGT2A2:NM_001105677:exon1:c.T57C:p.F	NOVEL		214	0	0	1	182	15	0.156581	1				
32	chr1	192544985	192544985	T	A	exonic	RGS1		synonymous SNV	RGS1:NM_002922:exon1:c.T63A:p.A21A	DBSNP		315	0	0	1	434	24	0.15557	1				
32	chr1	17567248	17567248	C	T	exonic	PADI1		nonsynonymous SNV	PADI1:NM_013358:exon15:c.C1751T:p.P58	NOVEL		332	0	0	1	310	24	0.147767	1				
32	chr6	57498973	57498973	G	T	exonic	PRIM2		unknown	UNKNOWN	NOVEL		393	0	0	1	395	30	0.145159	1				
32	chr19	55752865	55752865	G	C	exonic	PPP6R1		nonsynonymous SNV	PPP6R1:NM_014931:exon8:c.C988G:p.L33	NOVEL		139	0	0	1	183	9	0.139162	1				
32	chr6	52285237	52285237	C	G	exonic	EFHC1		nonsynonymous SNV	EFHC1:NM_018100:exon1:c.C29G:p.P10R	NOVEL		180	0	0	1	154	11	0.137095	1				
32	chr12	96912788	96912788	G	T	exonic	CFAP54		nonsynonymous SNV	CFAP54:NM_001306084:exon6:c.C881T:p.	NOVEL		62	0	0	1	108	7	0.125174	1				
32	chr14	73731015	73731015	C	T	exonic	PAPLN		synonymous SNV	PAPLN:NM_001365906:exon20:c.C2958T:	NOVEL		204	0	0	1	190	12	0.122164	1				
32	chr19	20308217	20308217	C	T	exonic	ZNF486		nonsynonymous SNV	ZNF486:NM_052852:exon4:c.C698T:p.T23	NOVEL		254	0	0	1	347	14	0.118054	1				
32	chr7	98782558	98782558	T	A	exonic	KPNA7		synonymous SNV	KPNA7:NM_001145715:exon7:c.A1128T:p.	NOVEL		136	0	0	1	111	6	0.105458	1				
32	chr1	171605704	171605704	G	A	exonic	MYOC		synonymous SNV	MYOC:NM_000261:exon3:c.C876T:p.G292	NOVEL		396	0	0	1	458	16	0.100213	1				
32	chr17	57812769	57812769	G	A	exonic	VMP1		synonymous SNV	VMP1:NM_001329394:exon3:c.G147A:p.Q	NOVEL		221	0	0	1	335	17	0.099316	1				
32	chr5	140480339	140480339	G	C	exonic	PCDHB3		nonsynonymous SNV	PCDHB3:NM_018937:exon1:c.G106C:p.A36	NOVEL		232	0	0	1	159	7	0.0958313	1				
32	chr7	150417246	150417246	C	T	exonic	GIMAP1;GIMAP1-		nonsynonymous SNV	GIMAP1-GIMAP5:NM_001199577:exon3:c	DBSNP+COSMIC	ID=COSM535823	133	63	0.982944	1	145	6	0.0817122	1				
32	chr12	109717680	109717680	G	A	exonic	FOXN4		synonymous SNV	FOXN4:NM_213596:exon10:c.C1350T:p.A	NOVEL		164	0	0	1	227	9	0.078423	1				
32	chrX	128621082	128621082	C	T	exonic	SMARCA1		nonsynonymous SNV	SMARCA1:NM_001282875:exon16:c.G209	NOVEL		88	8	0.0881617	1	88	2	0.0228492	1				
32	chr12	110240813	110240813	C	T	exonic	TRPV4		nonsynonymous SNV	TRPV4:NM_001177428:exon3:c.G695A:p.R	DBSNP+COSMIC	ID=COSM568044	67	25	0.471428	1	125	1	0.0163208	1				
32	chr5	109181658	109181658	T	G	exonic	MAN2A1		synonymous SNV	MAN2A1:NM_002372:exon18:c.T2793G:p.	NOVEL		643	33	0.149358	1	579	4	0.0155923	1				
32	chr9	100678501	100678501	T	A	exonic	TRMO		nonsynonymous SNV	TRMO:NM_016481:exon2:c.A196T:p.I66F	NOVEL		346	44	0.345182	1	413	1	0.00717101	1				
32	chr8	53568680	53568680	C	A	exonic	RB1CC1		stopgain	RB1CC1:NM_001083617:exon15:c.G3709T	COSMIC	ID=COSM442189	224	9	0.0587613	1	165	1	0.006364	1				
32	chr6	1313243	1313243	G	A	exonic	FOXQ1		nonsynonymous SNV	FOXQ1:NM_033260:exon1:c.G304A:p.G10	NOVEL		98	29	0.483154	1	133	0	0	1				
32	chr3	9988306	9988306	G	A	exonic	PRRT3		nonsynonymous SNV	PRRT3:NM_207351:exon4:c.C2551T:p.R85	NOVEL		88	26	0.482569	1	152	0	0	1				
32	chr8	59059308	59059308	C	A	exonic	FAM110B		nonsynonymous SNV	FAM110B:NM_147189:exon5:c.C519A:p.S	NOVEL		361	61	0.464378	1	386	0	0	1				
32	chr8	22472459	22472459	G	A	exonic	CCAR2		nonsynonymous SNV	CCAR2:NM_001363068:exon11:c.G1150A:	DBSNP+COSMIC	ID=COSM109813	40	17	0.453711	1	44	0	0	1				
32	chr2	187519405	187519405	G	C	exonic	ITGAV		nonsynonymous SNV	ITGAV:NM_001145000:exon14:c.G1426C:	NOVEL		95	25	0.440808	1	88	0	0	1				
32	chr17	38151528	38151528	G	A	exonic	PSMD3		nonsynonymous SNV	PSMD3:NM_002809:exon8:c.G1196A:p.R3	DBSNP		275	67	0.414514	1	394	0	0	1				
32	chr7	143657476	143657476	G	A	exonic	OR2F1		nonsynonymous SNV	OR2F1:NM_012369:exon1:c.G413A:p.G13	NOVEL		250	39	0.412678	1	227	0	0	1				
32	chr19	49442937	49442937	G	A	exonic	DHDH		nonsynonymous SNV	DHDH:NM_014475:exon4:c.G598A:p.V200	DBSNP+COSMIC	ID=COSM255837	220	40	0.388749	1	332	0	0	1				
32	chr21	28338100	28338100	G	A	exonic	ADAMTS5		nonsynonymous SNV	ADAMTS5:NM_007038:exon1:c.C611T:p.P	NOVEL		256	36	0.377207	1	291	0	0	1				
32	chr9	8518089	8518089	A	T	exonic	PTPRD		synonymous SNV	PTPRD:NM_001171025:exon8:c.T1272A:p.	NOVEL		322	45	0.375152	1	406	0	0	1				
32	chr11	72527835	72527835	C	A	exonic	ATG16L2		nonsynonymous SNV	ATG16L2:NM_033388:exon2:c.C181A:p.P6	NOVEL		128	17	0.358708	1	149	0	0	1				
32	chr11	6261617	6261617	G	A	exonic	CNGA4		nonsynonymous SNV	CNGA4:NM_001037329:exon4:c.G593A:p.	DBSNP+COSMIC	ID=COSM339800	387	50	0.349891	1	463	0	0	1				
32	chr15	73615673	73615673	T	A	exonic	HCN4		nonsynonymous SNV	HCN4:NM_005477:exon8:c.T2761A:p.S921	NOVEL		138	17	0.335566	1	157	0	0	1				
32	chr7	30491380	30491380	C	T	exonic	NOD1		synonymous SNV	NOD1:NM_001354849:exon6:c.G1653A:p.	DBSNP		374	45	0.32843	1	361	0	0	1				
32	chr9	125141147	125141147	G	C	exonic	PTGS1		nonsynonymous SNV	PTGS1:NM_001271165:exon4:c.G119C:p.R	DBSNP		177	18	0.282422	1	259	0	0	1				
32	chr1	240979657	240979657	G	T	exonic	RGS7		nonsynonymous SNV	RGS7:NM_001282773:exon9:c.C584A:p.P1	NOVEL		348	36	0.236894	1	496	0	0	1				
32	chr21	48080759	48080759	A	C	exonic	PRMT2		nonsynonymous SNV	PRMT2:NM_001535:exon8:c.A845C:p.K282	DBSNP		619	49	0.224429	1	701	0	0	1				
32	chr16	81187666	81187666	G	A	exonic	PKD1L2		unknown	UNKNOWN	DBSNP		48	12	0.223176	1	54	0	0	1				
32	chr19	45316697	45316697	G	T	exonic	BCAM		nonsynonymous SNV	BCAM:NM_001013257:exon6:c.G604T:p.G	NOVEL		261	22	0.196435	1	325	0	0	1				
32	chr7	82997033	82997033	A	G	exonic	SEMA3E		nonsynonymous SNV	SEMA3E:NM_001178129:exon17:c.T2017C	NOVEL		589	23	0.114927	1	451	0	0	1				
32	chr12	10224014	10224014	C	T	exonic	CLEC1A		nonsynonymous SNV	CLEC1A:NM_001297749:exon4:c.G485A:p.	DBSNP+COSMIC	ID=COSM135820	234	12	0.103214	1	250	0	0	1				
32	chr6	15627629	15627629	C	G	exonic	DTNBP1		nonsynonymous SNV	DTNBP1:NM_001271669:exon3:c.G195C:p	NOVEL		140	7	0.100756	1	163	0	0	1				
32	chr1	220986752	220986752	G	C	exonic	Mar-01		nonsynonymous SNV	MARC1:NM_022746:exon7:c.G1006C:p.G3	NOVEL		362	12	0.0810759	1	434	0	0	1				
32	chr4	48140747	48140747	G	A	exonic	TEC		stopgain	TEC:NM_003215:exon17:c.C1747T:p.R583	COSMIC	ID=COSM95672;	297	11	0.0755671	1	352	0	0	1				
32	chr4	126408663	126408663	A	T	exonic	FAT4		nonsynonymous SNV	FAT4:NM_001291285:exon16:c.A12986T:p	NOVEL		470	16	0.0696586	1	460	0	0	1				
32	chrX	136649519	136649519	C	A	exonic	ZIC3		nonsynonymous SNV	ZIC3:NM_001330661:exon1:c.C669A:p.N22	NOVEL		160	9	0.05634	1	164	0	0	1				
32	chrX	72667466	72667466	G	A	exonic	CDX4		nonsynonymous SNV	CDX4:NM_005193:exon1:c.G377A:p.S126N	NOVEL		184	8	0.0440808	1	198	0	0	1				
32	chr13	48881543	48881543	G	T	splicing	RB1	NM_000321:exon2:c.2			NOVEL		71	0	0	1	1	57	1.03821	1				
32	chr21	47414145	47414145	T	G	splicing	COL6A1	NM_001848:exon20:c			NOVEL		186	2	0.0325486	1	133	59	0.912287	1				
32	chr11	32417801	32417801	A	C	splicing	WT1	NM_001198551:exon7			NOVEL		161	0	0	1	157	30	0.476278	1				
33	chr3	86028324	86028324	C	A	exonic	CADM2		nonsynonymous SNV	CADM2:NM_001167674:exon8:c.C954A:p.I	NOVEL		379	55	0.264881	1	496	13	NA	1				
33	chr1	182026466	182026466	G	A	exonic	ZNF648		nonsynonymous SNV	ZNF648:NM_001009992:exon2:c.C680T:p.	NOVEL		129	60	0.962804	1	185	122	1.26044	1				
33	chr11	16071340	16071340	T	G	exonic	SOX6		nonsynonymous SNV	SOX6:NM_001145811:exon10:c.A1273C:p.	NOVEL		30	28	1.00904	1	29	32	1.17321	1				
33	chr17	7365655	7365655	G	T	exonic	ZBTB4		synonymous SNV	ZBTB4:NM_001128833:exon4:c.C2646T:p.	DBSNP		99	91	1.00107	1	107	118	1.17288	1				
33	chr2	125284881	125284881	C	T	exonic	CNTNAP5		synonymous SNV	CNTNAP5:NM_001367498:exon10:c.C1497	DBSNP+COSMIC	ID=COSM100609	31	38	1.1511	1	55	58	1.1479	1				
33	chr2	46588225	46588225	G	A	exonic	EPAS1		nonsynonymous SNV	EPAS1:NM_001430:exon6:c.G775A:p.D259	NOVEL		49	54	1.09581	1	94	98	1.14151	1				
33	chr3	170884989	170884989	G	T	exonic	TNIK		nonsynonymous SNV	TNIK:NM_001161560:exon10:c.C844A:p.Q	NOVEL		66	46	0.858455	1	114	95	1.12867	1				
33	chr11	123493258	123493258	C	T	exonic	GRAMD1B		synonymous SNV	GRAMD1B:NM_001286564:exon20:c.C205	COSMIC	ID=COSM556674	96	79	0.943554	1	111	112	1.12323	1				
33	chr20	58494577	58494577	T	C	exonic	SYCP2		nonsynonymous SNV	SYCP2:NM_014258:exon6:c.A373G:p.M125	DBSNP		26	30	1.11972	1	44	43	1.10536	1				
33	chr17	5012806	5012806	G	C	exonic	ZNF232		nonsynonymous SNV	ZNF232:NM_001320952:exon2:c.C381G:p	NOVEL		97	92	1.01743	1	154	135	1.0447	1				
33	chr15	55912265	55912265	G	A	exonic	PRTG		nonsynonymous SNV	PRTG:NM_173814:exon20:c.C3398T:p.S11	COSMIC	ID=COSM393678	55	48	0.974051	1	79	69	1.04266	1				
33	chr4	104640737	104640737	C	A	exonic	TACR3		synonymous SNV	TACR3:NM_001059:exon1:c.G96T:p.A32A	NOVEL		147	158	1.08277	1	223	191	1.03178	1				
33	chr9	133948221	133948221	T	A	exonic	LAMC3		nonsynonymous SNV	LAMC3:NM_006059:exon19:c.T3416A:p.L1	NOVEL		77	72	1.01001	1	121	102	1.02294	1				
33	chr3	4699878	4699878	T	A	exonic	ITPR1		nonsynonymous SNV	ITPR1:NM_001168272:exon12:c.T1022A:p	NOVEL		9	23	0.814863	1	19	49	1.00882	1				
33	chr19	8209844	8209844	C	T	exonic	FBN3		nonsynonymous SNV	FBN3:NM_001321431:exon6:c.G458A:p.R	DBSNP+COSMIC	ID=COSM273012	33	77	1.06149	2	67	119	1.00659	2				
33	chr11	123624744	123624744	G	T	exonic	OR6X1		synonymous SNV	OR6X1:NM_001005188:exon1:c.C483A:p.L	NOVEL		44	42	1.02077	1	69	56	1.00192	1				
33	chr7	134722242	134722242	T	G	exonic	AGBL3		nonsynonymous SNV	AGBL3:NM_178563:exon8:c.T1411G:p.Y47	NOVEL		43	32	0.891798	1	53	43	1.00173	1				
33	chr18	61324582	61324582	G	A	exonic	SERPINB3		synonymous SNV	SERPINB3:NM_006919:exon6:c.C534T:p.N	DBSNP+COSMIC	ID=COSM721635	120	106	0.980337	1	166	132	0.990634	1				
33	chr2	186660300	186660300	A	C	exonic	FSIP2		nonsynonymous SNV	FSIP2:NM_173651:exon16:c.A8437C:p.K28	NOVEL		202	169	0.952118	1	323	251	0.977951	1				
33	chr1	8073848	8073848	T	C	exonic	ERRFI1		nonsynonymous SNV	ERRFI1:NM_018948:exon4:c.A811G:p.R27	NOVEL		53	33	0.802035	1	79	61	0.974445	1				
33	chr15	74467556	74467556	C	A	exonic	ISLR		nonsynonymous SNV	ISLR:NM_005545:exon2:c.C357A:p.H119Q	NOVEL		218	188	0.967854	1	349	264	0.963161	1				
33	chr1	205033504	205033504	G	A	exonic	CNTN2		nonsynonymous SNV	CNTN2:NM_001346083:exon11:c.G1295A:	DBSNP+COSMIC	ID=COSM422985	135	66	0.995855	1	226	97	0.952508	1				
33	chr1	236987458	236987458	A	C	exonic	MTR		nonsynonymous SNV	MTR:NM_000254:exon9:c.A804C:p.R268S	NOVEL		205	76	0.820268	1	292	125	0.950765	1				
33	chr3	119177013	119177013	T	C	exonic	TMEM39A		nonsynonymous SNV	TMEM39A:NM_018266:exon3:c.A188G:p.	NOVEL		105	89	0.958884	1	140	103	0.947952	1				
33	chr17	65918959	65918959	C	T	exonic	BPTF		nonsynonymous SNV	BPTF:NM_182641:exon14:c.C5561T:p.T18	DBSNP		54	44	0.938436	1	102	75	0.94764	1				
33	chr18	44015271	44015271	T	A	exonic	RNF165		nonsynonymous SNV	RNF165:NM_152470:exon3:c.T397A:p.S13	NOVEL		145	100	0.853123	1	198	145	0.94543	1				
33	chr2	219530765	219530765	G	T	exonic	RNF25		synonymous SNV	RNF25:NM_022453:exon7:c.C447A:p.T149	NOVEL		68	58	0.962133	1	94	68	0.938748	1				
33	chr2	241683366	241683366	C	T	exonic	KIF1A		nonsynonymous SNV	KIF1A:NM_004321:exon31:c.G3274A:p.V1	DBSNP		75	66	0.978369	1	125	90	0.93618	1				
33	chr6	46867766	46867766	C	T	exonic	ADGRF5		nonsynonymous SNV	ADGRF5:NM_001098518:exon3:c.G157A:	DBSNP		110	86	0.917108	1	157	113	0.935987	1				
33	chr12	102142906	102142906	A	T	exonic	GNPTAB		nonsynonymous SNV	GNPTAB:NM_024312:exon20:c.T3666A:p.	NOVEL		49	45	1.0006	1	85	61	0.934399	1				
33	chr21	41033379	41033379	C	T	exonic	B3GALT5		nonsynonymous SNV	B3GALT5:NM_001278650:exon3:c.C893T:	NOVEL		22	29	1.18852	1	55	39	0.927881	1				
33	chr19	38708207	38708207	C	T	exonic	DPF1		nonsynonymous SNV	DPF1:NM_001135155:exon7:c.G701A:p.R2	NOVEL		83	67	0.933601	1	116	81	0.919547	1				
33	chr15	90897971	90897971	C	G	exonic	ZNF774		nonsynonymous SNV	ZNF774:NM_001004309:exon2:c.C79G:p.C	NOVEL		37	31	0.952863	1	54	37	0.909318	1				
33	chr3	63968020	63968020	G	T	exonic	ATXN7		nonsynonymous SNV	ATXN7:NM_001128149:exon3:c.G476T:p.G	DBSNP		39	35	0.860047	1	49	50	0.881222	1				
33	chr5	14482857	14482857	G	A	exonic	TRIO		nonsynonymous SNV	TRIO:NM_007118:exon46:c.G6632A:p.G22	NOVEL		25	20	0.928956	1	37	24	0.879907	1				
33	chr3	38738905	38738905	T	C	exonic	SCN10A		nonsynonymous SNV	SCN10A:NM_001293307:exon26:c.A5512G	NOVEL		23	48	0.76646	1	58	83	0.824114	1				
33	chr19	1488232	1488232	A	G	exonic	PCSK4		synonymous SNV	PCSK4:NM_017573:exon3:c.T342C:p.S114S	NOVEL		140	51	0.809814	1	214	72	0.792163	1				
33	chr19	1488231	1488231	C	G	exonic	PCSK4		nonsynonymous SNV	PCSK4:NM_017573:exon3:c.G343C:p.V115	NOVEL		141	51	0.805596	1	216	72	0.786661	1				
33	chr16	84358030	84358030	C	T	exonic	WFDC1		stopgain	WFDC1:NM_001282466:exon5:c.C568T:p.R	DBSNP		31	26	0.953403	1	72	39	0.785773	1				
33	chr8	143784724	143784724	G	C	exonic	LY6K		nonsynonymous SNV	LY6K:NM_017527:exon3:c.G433C:p.G145R	NOVEL		23	22	1.02185	1	51	25	0.735668	1				
33	chr12	25380275	25380275	T	G	exonic	KRAS		nonsynonymous SNV	KRAS:NM_001369786:exon3:c.A183C:p.Q6	DBSNP+COSMIC	ID=COSM554,CO	68	2	0.0597186	1	85	29	0.568916	1				
33	chr17	12656267	12656267	G	A	exonic	MYOCD		synonymous SNV	MYOCD:NM_001146312:exon10:c.G1662A	DBSNP+COSMIC	ID=COSM558839	72	1	0.0286322	1	82	24	0.506362	1				
33	chr15	84286866	84286866	C	T	exonic	SH3GL3		nonsynonymous SNV	SH3GL3:NM_001301108:exon9:c.C664T:p.	DBSNP		111	4	0.0727009	1	109	31	0.49521	1				
33	chrX	120009256	120009256	G	A	exonic	CT47B1		nonsynonymous SNV	CT47B1:NM_001145718:exon1:c.C269T:p.	DBSNP		25	1	0.0786568	1	28	7	0.423643	1				
33	chr19	19735078	19735078	T	C	exonic	LPAR2		nonsynonymous SNV	LPAR2:NM_004720:exon3:c.A1043G:p.D34	NOVEL		75	0	0	1	113	26	0.418325	1				
33	chr11	8132599	8132599	G	A	exonic	RIC3		synonymous SNV	RIC3:NM_001135109:exon2:c.C210T:p.D70	DBSNP		221	52	0.52128	1	263	51	0.403301	1				
33	chr1	108307708	108307708	A	T	exonic	VAV3		nonsynonymous SNV	VAV3:NM_006113:exon9:c.T911A:p.L304Q	NOVEL		101	15	0.270278	1	118	29	0.374672	1				
33	chr15	48818406	48818406	T	A	exonic	FBN1		nonsynonymous SNV	FBN1:NM_000138:exon9:c.A909T:p.E303D	NOVEL		138	64	0.662226	1	261	46	0.3351	1				
33	chr12	31605131	31605131	G	A	exonic	DENND5B		stopgain	DENND5B:NM_001366891:exon5:c.C841T:	NOVEL		197	1	0.0105563	1	229	40	0.332555	1				
33	chr12	4446271	4446271	T	G	exonic	TIGAR		nonsynonymous SNV	TIGAR:NM_020375:exon3:c.T137G:p.L46R	NOVEL		175	67	0.578678	1	265	46	0.33079	1				
33	chr14	105409220	105409220	G	C	exonic	AHNAK2		nonsynonymous SNV	AHNAK2:NM_001350929:exon7:c.C12268	NOVEL		52	30	0.76469	1	110	18	0.314498	1				
33	chr9	34972492	34972492	G	T	exonic	PHF24		nonsynonymous SNV	PHF24:NM_001304333:exon3:c.G528T:p.N	NOVEL		35	0	0	1	31	5	0.310615	1				
33	chr15	63046007	63046007	C	T	exonic	TLN2		synonymous SNV	TLN2:NM_015059:exon33:c.C4368T:p.S14	NOVEL		67	29	0.6314	1	162	24	0.288572	1				
33	chr1	115256530	115256530	G	T	exonic	NRAS		nonsynonymous SNV	NRAS:NM_002524:exon3:c.C181A:p.Q61K	DBSNP+COSMIC	ID=COSM580;OC	78	47	0.785897	1	147	26	0.285429	1				
33	chr2	185802023	185802023	C	T	exonic	ZNF804A		synonymous SNV	ZNF804A:NM_194250:exon4:c.C1900T:p.L	NOVEL		95	27	0.462575	1	132	19	0.281405	1				
33	chr1	200017564	200017564	C	A	exonic	NR5A2		nonsynonymous SNV	NR5A2:NM_001276464:exon4:c.C512A:p.S	NOVEL		358	85	0.58192	1	574	53	0.268107	1				
33	chr9	130570921	130570921	C	T	exonic	FPGS		nonsynonymous SNV	FPGS:NM_001288803:exon9:c.C829T:p.R2	DBSNP		66	0	0	1	75	10	0.263109	1				
33	chr1	10715788	10715788	C	T	exonic	CASZ1		nonsynonymous SNV	CASZ1:NM_001079843:exon9:c.G1583A:p.	NOVEL		130	41	0.501148	1	181	24	0.261826	1				
33	chr12	109841825	109841825	T	G	exonic	MYO1H		nonsynonymous SNV	MYO1H:NM_001101421:exon6:c.T741G:p.	NOVEL		234	0	0	1	292	38	0.257528	1				
33	chr7	146829358	146829358	G	A	exonic	CNTNAP2		unknown	UNKNOWN	DBSNP		111	0	0	1	150	19	0.251433	1				
33	chr15	66618665	66618665	G	T	exonic	DIS3L		stopgain	DIS3L:NM_001323937:exon11:c.G1786T:p.	NOVEL		49	0	0	1	64	8	0.248492	1				
33	chr10	25885651	25885651	G	C	exonic	GPR158		nonsynonymous SNV	GPR158:NM_020752:exon10:c.G2078C:p.	COSMIC	ID=COSM213379	112	0	0	1	160	38	0.237295	1				
33	chr6	39874282	39874282	C	T	exonic	MOCS1		nonsynonymous SNV	MOCS1:NM_001358529:exon10:c.G1714A	DBSNP		30	12	0.597186	1	71	8	0.226474	1				
33	chr7	93519620	93519620	C	T	exonic	TFPI2		nonsynonymous SNV	TFPI2:NM_001271004:exon2:c.G100A:p.E3	NOVEL		135	0	0	1	181	20	0.22253	1				
33	chr11	124412728	124412728	G	T	exonic	OR8B12		nonsynonymous SNV	OR8B12:NM_001005195:exon1:c.C823A:p	NOVEL		195	54	0.453286	1	334	35	0.212128	1				
33	chr17	2290529	2290529	G	A	exonic	MNT		nonsynonymous SNV	MNT:NM_020310:exon6:c.C1415T:p.P472L	NOVEL		56	26	0.662731	1	105	10	0.194472	1				
33	chr12	25380276	25380276	T	A	exonic	KRAS		nonsynonymous SNV	KRAS:NM_001369786:exon3:c.A182T:p.Q6	DBSNP+COSMIC	ID=COSM114013	69	1	0.0298593	1	105	9	0.17656	1				
33	chr6	10556676	10556676	A	T	exonic	GCNT2		nonsynonymous SNV	GCNT2:NM_001491:exon1:c.A20T:p.Y7F	NOVEL		45	0	0	1	62	5	0.166898	1				
33	chr1	99161196	99161196	T	A	exonic	SNX7		nonsynonymous SNV	SNX7:NM_152238:exon4:c.T597A:p.N199K	NOVEL		64	0	0	1	52	6	0.155748	1				
33	chr11	124765392	124765392	G	T	exonic	ROBO4		nonsynonymous SNV	ROBO4:NM_001301088:exon6:c.C562A:p.I	NOVEL		174	2	0.0237517	1	244	17	0.145668	1				
33	chr6	145956474	145956474	G	A	exonic	EPM2A		nonsynonymous SNV	EPM2A:NM_001018041:exon3:c.C625T:p.F	NOVEL		192	0	0	1	199	15	0.134987	1				
33	chr10	118423745	118423745	A	G	exonic	C10orf82		nonsynonymous SNV	C10orf82:NM_001330142:exon5:c.T499C:p	NOVEL		142	6	0.0847359	1	217	13	0.126407	1				

33	chr19	48626500	48626500	C	T	exonic	LIG1		nonsynonymous SNV	LIG1:NM_001289064:exon20:c.G1876A:p.V	NOVEL		137	0	
33	chr1	43898443	43898443	C	T	exonic	SZT2		stopgain	SZT2:NM_015284:exon38:c.C5404T:p.Q18	NOVEL		111	0	
33	chr1	155912476	155912476	G	T	exonic	RXFP4		nonsynonymous SNV	RXFP4:NM_181885:exon1:c.G976T:p.D326	NOVEL		274	0	
33	chr7	21468915	21468915	G	A	exonic	SP4		synonymous SNV	SP4:NM_001326542:exon3:c.G81A:p.Q27	COSMIC	ID=COSM399549	98	0	
33	chr2	168102877	168102877	G	T	exonic	XIRP2		nonsynonymous SNV	XIRP2:NM_001199144:exon7:c.G4309T:p.V	NOVEL		119	0	
33	chr7	2611934	2611934	C	T	exonic	IQCE		nonsynonymous SNV	IQCE:NM_001287501:exon3:c.C173T:p.P58	NOVEL		181	1	
33	chr8	12950267	12950267	C	T	exonic	DLC1		synonymous SNV	DLC1:NM_001348084:exon8:c.G2061A:p.R	NOVEL		54	0	
33	chr6	90642487	90642487	C	A	exonic	BACH2		nonsynonymous SNV	BACH2:NM_001170794:exon7:c.G2166T:p	NOVEL		173	0	
33	chr8	134296530	134296530	C	G	exonic	NDRG1		nonsynonymous SNV	NDRG1:NM_001135242:exon2:c.G25C:p.D	NOVEL		319	0	
33	chr14	20839384	20839384	G	C	exonic	TEP1		nonsynonymous SNV	TEP1:NM_001319035:exon49:c.C6986G:p.	NOVEL		298	1	
33	chr18	6824822	6824822	C	A	exonic	ARHGAP28		nonsynonymous SNV	ARHGAP28:NM_001366230:exon2:c.C184	NOVEL		186	25	
33	chr11	1272480	1272480	C	T	exonic	MUC5B		synonymous SNV	MUC5B:NM_002458:exon31:c.C14370T:p.S	NOVEL		522	49	
33	chr3	107447727	107447727	C	A	exonic	BBX		nonsynonymous SNV	BBX:NM_001142568:exon6:c.C521A:p.P17	NOVEL		257	25	
33	chr5	93856353	93856353	T	G	exonic	KIAA0825		nonsynonymous SNV	KIAA0825:NM_001145678:exon5:c.A570C:	NOVEL		38	6	
33	chr22	19494973	19494973	G	A	exonic	CDC45		nonsynonymous SNV	CDC45:NM_001178011:exon10:c.G751A:p.	DBSNP		215	25	
33	chr14	74500865	74500865	G	T	exonic	BBOF1		nonsynonymous SNV	BBOF1:NM_025057:exon4:c.G486T:p.E162	NOVEL		109	10	
33	chr2	152553197	152553197	G	T	exonic	NEB		nonsynonymous SNV	NEB:NM_001164507:exon17:c.C1523A:p.S	DBSNP+COSMIC	ID=COSM159066	266	23	
33	chr15	63966700	63966700	G	C	exonic	HERC1		nonsynonymous SNV	HERC1:NM_003922:exon38:c.C7687G:p.L2	NOVEL		160	10	
33	chr19	57176069	57176069	G	A	exonic	ZNF835		synonymous SNV	ZNF835:NM_001005850:exon2:c.C498T:p.	COSMIC	ID=COSM683354	171	8	
33	chr6	119136347	119136347	G	A	exonic	MCM9		synonymous SNV	MCM9:NM_017696:exon12:c.C3072T:p.N1	DBSNP		177	8	
33	chr11	64031224	64031224	C	T	exonic	PLCB3		synonymous SNV	PLCB3:NM_001184883:exon19:c.C2316T:p	NOVEL		337	15	
33	chr17	7106870	7106870	G	A	exonic	DLG4		synonymous SNV	DLG4:NM_001321076:exon5:c.C198T:p.R6	DBSNP+COSMIC	ID=COSM158913	272	9	
34	chrX	49062242	49062242	C	G	exonic	CACNA1F		nonsynonymous SNV	CACNA1F:NM_001256789:exon47:c.G5504	NOVEL		10	47	NA
34	chrX	100750029	100750029	G	A	exonic	ARMCX4		synonymous SNV	ARMCX4:NM_001256155:exon2:c.G6453A	NOVEL		107	89	NA
34	chrX	73524355	73524355	G	A	exonic	ZCCHC13		nonsynonymous SNV	ZCCHC13:NM_203303:exon1:c.G254A:p.R8	DBSNP+COSMIC	ID=COSM146933	156	0	NA
34	chrX	101909663	101909663	G	A	exonic	GPRASP1		synonymous SNV	GPRASP1:NM_001099411:exon3:c.G822A:	NOVEL		134	0	NA
34	chrX	107334734	107334734	G	A	exonic	PSMD10		nonsynonymous SNV	PSMD10:NM_002814:exon1:c.C17T:p.S6F,	NOVEL		393	0	NA
34	chrX	47918554	47918554	G	A	exonic	ZNF630		nonsynonymous SNV	ZNF630:NM_001037735:exon5:c.C1277T:p	NOVEL		67	0	NA
34	chr8	82626260	82626260	C	A	exonic	ZFAND1		stopgain	ZFAND1:NM_001170796:exon6:c.G373T:p	NOVEL		64	30	
34	chr8	68044300	68044300	G	A	exonic	CSPP1		synonymous SNV	CSPP1:NM_001291339:exon12:c.G915A:p.	NOVEL		87	81	
34	chr17	73096181	73096181	C	T	exonic	SLC16A5		synonymous SNV	SLC16A5:NM_001369668:exon4:c.C423T:p	DBSNP		170	76	
34	chr6	112402394	112402394	A	G	exonic	TUBE1		nonsynonymous SNV	TUBE1:NM_016262:exon5:c.T227C:p.M76	NOVEL		25	17	
34	chr4	44680665	44680665	G	T	exonic	GUF1		nonsynonymous SNV	GUF1:NM_001345868:exon1:c.G26T:p.W9	NOVEL		27	14	
34	chr11	45891733	45891733	G	C	exonic	CRY2		nonsynonymous SNV	CRY2:NM_001127457:exon8:c.G1141C:p.D	NOVEL		161	7	
34	chr7	128478662	128478662	G	A	exonic	FLNC		nonsynonymous SNV	FLNC:NM_001127487:exon8:c.G1216A:p.G	COSMIC	ID=COSM364079	195	0	
34	chr5	1087141	1087141	C	T	exonic	SLC12A7		synonymous SNV	SLC12A7:NM_006598:exon6:c.G552A:p.G1	NOVEL		163	96	
34	chr15	55722902	55722902	C	T	exonic	DNAAF4		nonsynonymous SNV	DNAAF4:NM_001033559:exon9:c.G1123A	DBSNP+COSMIC	ID=COSM125087	58	29	
34	chr17	46919176	46919176	G	A	exonic	CALCOCO2		nonsynonymous SNV	CALCOCO2:NM_001261390:exon2:c.G107A	NOVEL		186	71	
34	chr17	80038620	80038620	G	A	exonic	FASN		synonymous SNV	FASN:NM_004104:exon39:c.C6774T:p.H22	NOVEL		108	38	
34	chr5	178413488	178413488	C	T	exonic	GRM6		synonymous SNV	GRM6:NM_000843:exon9:c.G1767A:p.P58	DBSNP+COSMIC	ID=COSM385415	95	37	
34	chr17	73838555	73838555	G	A	exonic	UNC13D		synonymous SNV	UNC13D:NM_199242:exon6:c.C528T:p.I17	NOVEL		169	3	
34	chr3	141497168	141497168	C	T	exonic	GRK7		synonymous SNV	GRK7:NM_139209:exon1:c.C42T:p.T14T	DBSNP+COSMIC	ID=COSM722666	205	78	
34	chr1	118165650	118165650	A	T	exonic	FAM46C		nonsynonymous SNV	FAM46C:NM_017709:exon2:c.A160T:p.T54	NOVEL		139	103	
34	chr7	36194559	36194559	G	A	exonic	EEPD1		nonsynonymous SNV	EEPD1:NM_030636:exon2:c.G626A:p.G209	NOVEL		262	67	
34	chr17	78313485	78313485	G	A	exonic	RNF213		nonsynonymous SNV	RNF213:NM_001256071:exon26:c.G5318A	NOVEL		199	89	
34	chr5	140222850	140222850	G	C	exonic	PCDHA8		synonymous SNV	PCDHA8:NM_018911:exon1:c.G1944C:p.L6	NOVEL		247	81	
34	chr1	8390493	8390493	C	A	exonic	SLC45A1		nonsynonymous SNV	SLC45A1:NM_001080397:exon5:c.C1042A:	NOVEL		165	0	
34	chr17	10354742	10354742	G	T	exonic	MYH4		nonsynonymous SNV	MYH4:NM_017533:exon28:c.C3766A:p.L12	NOVEL		121	66	
34	chr4	46305584	46305584	A	T	exonic	GABRA2		nonsynonymous SNV	GABRA2:NM_001114175:exon7:c.T749A:p	NOVEL		156	105	
34	chr5	54639283	54639283	G	T	exonic	MTREX		nonsynonymous SNV	MTREX:NM_015360:exon8:c.G906T:p.Q30	NOVEL		234	95	
34	chr9	131830555	131830555	G	A	exonic	MIGA2		nonsynonymous SNV	MIGA2:NM_001329990:exon13:c.G1348A:	NOVEL		317	165	
34	chr11	33631338	33631338	G	A	exonic	KIAA1549L		nonsynonymous SNV	KIAA1549L:NM_012194:exon15:c.G5105A:	DBSNP		5	29	
34	chr1	155706792	155706792	C	G	exonic	DAP3		nonsynonymous SNV	DAP3:NM_001199850:exon11:c.C947G:p.P	NOVEL		103	52	
34	chr9	140446625	140446625	G	A	exonic	MRPL41		nonsynonymous SNV	MRPL41:NM_032477:exon2:c.G92A:p.R31	NOVEL		137	54	
34	chr20	44985259	44985259	C	G	exonic	SLC35C2		nonsynonymous SNV	SLC35C2:NM_015945:exon4:c.G289C:p.D9	NOVEL		107	91	
34	chr12	117968754	117968754	C	G	exonic	KSR2		synonymous SNV	KSR2:NM_173598:exon12:c.G1794C:p.S59	NOVEL		98	71	
34	chr5	161576227	161576227	G	T	exonic	GABRG2		nonsynonymous SNV	GABRG2:NM_000816:exon8:c.G1036T:p.V	NOVEL		293	140	
34	chr9	5988465	5988465	G	A	exonic	KIAA2026		nonsynonymous SNV	KIAA2026:NM_001017969:exon2:c.C674T:	NOVEL		358	118	
34	chr3	135721920	135721920	T	G	exonic	PPP2R3A		nonsynonymous SNV	PPP2R3A:NM_002718:exon2:c.T1580G:p.L	NOVEL		265	93	
34	chr3	133969388	133969388	C	A	exonic	RYK		unknown	UNKNOWN	NOVEL		16	6	
34	chr2	32526522	32526522	T	A	exonic	YIPF4		synonymous SNV	YIPF4:NM_032312:exon5:c.T555A:p.L185L	NOVEL		192	126	
34	chr14	21542693	21542693	C	T	exonic	ARHGEF40		synonymous SNV	ARHGEF40:NM_018071:exon3:c.C804T:p.V	DBSNP		45	39	
34	chr6	138611018	138611018	C	A	exonic	ARFGEF3		nonsynonymous SNV	ARFGEF3:NM_020340:exon18:c.C2960A:p	NOVEL		80	53	
34	chr16	81187858	81187858	G	A	exonic	PKD1L2		unknown	UNKNOWN	NOVEL		25	88	
34	chr9	36376021	36376021	A	T	exonic	RNF38		nonsynonymous SNV	RNF38:NM_194329:exon2:c.T116A:p.M39	NOVEL		177	76	
34	chr12	29509317	29509317	G	A	exonic	ERGIC2		synonymous SNV	ERGIC2:NM_016570:exon8:c.C570T:p.G19	NOVEL		42	139	
34	chr19	13923949	13923949	G	T	exonic	ZSWIM4		nonsynonymous SNV	ZSWIM4:NM_001367834:exon6:c.G1151T	NOVEL		203	71	
34	chr1	240072045	240072045	G	C	exonic	CHRM3		nonsynonymous SNV	CHRM3:NM_000740:exon5:c.G1294C:p.E4	NOVEL		120	42	
34	chr20	3730848	3730848	C	T	exonic	HSPA12B		synonymous SNV	HSPA12B:NM_001318322:exon10:c.C1017	DBSNP		78	61	
34	chr19	8987309	8987309	G	A	exonic	MUC16		synonymous SNV	MUC16:NM_024690:exon68:c.C41778T:p.F	NOVEL		78	35	
34	chr5	145719690	145719690	C	T	exonic	POU4F3		nonsynonymous SNV	POU4F3:NM_002700:exon2:c.C700T:p.L23	NOVEL		408	156	
34	chr7	97842055	97842055	C	T	exonic	BHLHA15		nonsynonymous SNV	BHLHA15:NM_177455:exon1:c.C434T:p.P1	NOVEL		258	170	
34	chr6	74516617	74516617	A	T	exonic	CD109		nonsynonymous SNV	CD109:NM_001159588:exon24:c.A2780T:p	NOVEL		164	126	
34	chr9	34709662	34709662	C	T	exonic	CCL21		nonsynonymous SNV	CCL21:NM_002989:exon3:c.G206A:p.R69H	DBSNP+COSMIC	ID=COSM124759	157	76	
34	chr19	4229552	4229552	C	A	exonic	EBI3		nonsynonymous SNV	EBI3:NM_005755:exon1:c.C5A:p.T2N	NOVEL		280	101	
34	chr5	35691350	35691350	T	C	exonic	SPEF2		nonsynonymous SNV	SPEF2:NM_024867:exon11:c.T1736C:p.L57	NOVEL		175	39	
34	chr15	101555562	101555562	G	A	exonic	LRRK1		nonsynonymous SNV	LRRK1:NM_024652:exon12:c.G1564A:p.A5	NOVEL		699	172	
34	chr2	105984171	105984171	G	T	exonic	FHL2		synonymous SNV	FHL2:NM_001318897:exon3:c.C15A:p.G5G	NOVEL		43	40	
34	chr15	23812446	23812446	T	G	exonic	MKRN3		nonsynonymous SNV	MKRN3:NM_005664:exon1:c.T1517G:p.I50	NOVEL		372	134	
34	chr5	140745833	140745833	G	A	exonic	PCDHGA5		nonsynonymous SNV	PCDHGA5:NM_018918:exon1:c.G1936A:p.	DBSNP+COSMIC	ID=COSM471249	184	0	
34	chr2	36737280	36737280	G	T	exonic	CRIM1		nonsynonymous SNV	CRIM1:NM_016441:exon9:c.G1656T:p.L55	NOVEL		179	0	
34	chr14	64954414	64954414	C	G	exonic	ZBTB25		nonsynonymous SNV	ZBTB25:NM_001354683:exon3:c.G535C:p.	NOVEL		74	67	
34	chr13	27241676	27241676	G	A	exonic	WASF3		nonsynonymous SNV	WASF3:NM_001291965:exon5:c.G291A:p.	COSMIC	ID=COSM346799	33	92	
34	chr17	1268176	1268176	T	C	exonic	YWHAE		nonsynonymous SNV	YWHAE:NM_006761:exon2:c.A241G:p.M8	NOVEL		104	83	
34	chr13	103514576	103514576	G	T	exonic	BIVM-ERCC5;ERCC		nonsynonymous SNV	ERCC5:NM_000123:exon8:c.G1077T:p.E35	NOVEL		21	52	
34	chr4	77305384	77305384	C	T	exonic	CCDC158		nonsynonymous SNV	CCDC158:NM_001042784:exon5:c.G583A:	NOVEL		231	135	
34	chr5	140755023	140755023	C	G	exonic	PCDHGA6		stopgain	PCDHGA6:NM_018919:exon1:c.C1373G:p.	NOVEL		519	205	
34	chr2	168114709	168114709	C	A	exonic	XIRP2		synonymous SNV	XIRP2:NM_001199145:exon8:c.C987A:p.P3	NOVEL		365	0	
34	chr1	212151685	212151685	T	C	exonic	INTS7		nonsynonymous SNV	INTS7:NM_001199809:exon10:c.A1252G:p	NOVEL		106	87	
34	chr11	65414509	65414509	C	T	exonic	SIPA1		synonymous SNV	SIPA1:NM_006747:exon8:c.C2004T:p.A668	NOVEL		66	47	
34	chr9	14808009	14808009	G	A	exonic	FREM1		nonsynonymous SNV	FREM1:NM_144966:exon18:c.C3017T:p.A1	DBSNP		115	27	
34	chr7	129152195	129152195	C	A	exonic	SMKR1		stopgain	SMKR1:NM_001195243:exon2:c.C111A:p.Y	NOVEL		955	188	
34	chr3	159711249	159711249	C	G	exonic	IL12A		nonsynonymous SNV	IL12A:NM_001354583:exon3:c.C276G:p.C9	NOVEL		50	13	
34	chr19	42341244	42341244	T	A	exonic	LYPD4		nonsynonymous SNV	LYPD4:NM_001291419:exon5:c.A609T:p.L2	NOVEL		53	2	
34	chr3	120315348	120315348	C	T	exonic	NDUFB4		synonymous SNV	NDUFB4:NM_001168331:exon1:c.C142T:p	NOVEL		118	154	
34	chr3	120315347	120315347	C	A	exonic	NDUFB4		stopgain	NDUFB4:NM_001168331:exon1:c.C141A:p	NOVEL		119	154	
34	chr18	44118239	44118239	G	T	exonic	LOXHD1		nonsynonymous SNV	LOXHD1:NM_001308013:exon6:c.C354A:p.	NOVEL		134	59	
34	chr1	245018733	245018733	T	C	exonic	HNRNPU		nonsynonymous SNV	HNRNPU:NM_004501:exon12:c.A2288G:p	NOVEL		13	10	
34	chr2	219204609	219204609	C	T	exonic	PNKD		nonsynonymous SNV	PNKD:NM_022572:exon2:c.C268T:p.R90C,	DBSNP		192	101	
34	chr3	164907659	164907659	A	G	exonic	SLITRK3		synonymous SNV	SLITRK3:NM_001318810:exon2:c.T960C:p.	NOVEL		169	63	
34	chr17	10398530	10398530	C	A	exonic	MYH1		nonsynonymous SNV	MYH1:NM_005963:exon36:c.G5274T:p.K17	NOVEL		190	115	
34	chr16	72923679	72923679	C	T	exonic	ZFHX3		synonymous SNV	ZFHX3:NM_001164766:exon3:c.G657A:p.E	DBSNP		15	44	
34	chr3	118623558	118623558	G	A	exonic	IGSF11		nonsynonymous SNV	IGSF11:NM_001015887:exon6:c.C791T:p.A	DBSNP		176	63	
34	chr1	205132981	205132981	A	T	exonic	DSTYK		nonsynonymous SNV	DSTYK:NM_015375:exon4:c.T1427A:p.V47	NOVEL		186	96	
34	chr6	129635936	129635936	G	A	exonic	LAMA2		nonsynonymous SNV	LAMA2:NM_000426:exon24:c.G3548A:p.R	NOVEL		41	24	
34	chr5	38885562	38885562	C	T	exonic	OSMR		nonsynonymous SNV	OSMR:NM_001168355:exon6:c.C815T:p.S	COSMIC	ID=COSM169568	207	106	
34	chr12	111078865	111078865	G	A	exonic	TCTN1		nonsynonymous SNV	TCTN1:NM_001082537:exon9:c.G1015A:p	NOVEL		217	147	
34	chr7	24789345	24789345	C	T	exonic	GSDME		nonsynonymous SNV	GSDME:NM_001127453:exon2:c.G49A:p.G	NOVEL		157	3	
34	chr7	1786692	1786692	G	A	exonic	ELFN1		synonymous SNV	ELFN1:NM_001128636:exon3:c.G2460A:p.	NOVEL		300	86	
34	chr11	68582876	68582876	G	A	exonic	CPT1A		synonymous SNV	CPT1A:NM_001031847:exon2:c.C67T:p.L2	NOVEL		97	4	
34	chr5	45262042	45262042	C	T	exonic	HCN1		nonsynonymous SNV	HCN1:NM_021072:exon8:c.G2654A:p.R88	DBSNP+COSMIC	ID=COSM361676	503	123	
34	chr7	80435015	80435015	G	A	exonic	SEMA3C		stopgain	SEMA3C:NM_001350120:exon7:c.C652T:p	DBSNP		289	80	
34	chr6	160496887	160496887	C	G	exonic	IGF2R		synonymous SNV	IGF2R:NM_000876:exon36:c.C5175G:p.G1	NOVEL		153	95	
34	chr11	102647033	102647033	C	G	exonic	MMP10		nonsynonymous SNV	MMP10:NM_002425:exon6:c.G910C:p.E30	NOVEL		144	66	
34	chr2	133971293	133971293	C	A	exonic	NCKAP5		nonsynonymous SNV	NCKAP5:NM_207363:exon5:c.G202T:p.A6	NOVEL		69	53	
34	chr3	141632612	141632612	T	C	exonic	ATP1B3		synonymous SNV	ATP1B3:NM_001679:exon4:c.T465C:p.S15	DBSNP		111	33	
34	chr7	149191143	149191143	C	T	exonic	ZNF746		nonsynonymous SNV	ZNF746:NM_001163474:exon3:c.G343A:p	NOVEL		399	226	
34	chr5	159399477	159399477	C	T	exonic	ADRA1B		nonsynonymous SNV	ADRA1B:NM_000679:exon2:c.C1541T:p.P5	NOVEL		29	21	
34	chr12	56981358	56981358	G	A	exonic	RBMS2		synonymous SNV	RBMS2:NM_002898:exon11:c.G972A:p.G3	NOVEL		29	29	
34	chr8	118849347	118849347	C	A	exonic	EXT1		nonsynonymous SNV	EXT1:NM_000127:exon2:c.G1056T:p.Q352	NOVEL		60	0	
34	chr11	27360368	27360368	C	G	exonic	CCDC34		stoploss	CCDC34:NM_030771:exon6:c.G1122C:p.X3	NOVEL		32	22	
34	chr3	44879821	44879821	A	T	exonic	KIF15		nonsynonymous SNV	KIF15:NM_020242:exon27:c.A3226T:p.T10	NOVEL		82	38	
34	chr15	65471319	65471319	C	G	exonic	CLPX		nonsynonymous SNV	CLPX:NM_006660:exon3:c.G311C:p.R104P	NOVEL		381	137	
34	chr9	90501338	90501338	A	T	exonic	SPATA31E1		stopgain	SPATA31E1:NM_178828:exon4:c.A1936T:p	NOVEL		140	4	
34	chr1	20668990	20668990	G	T	exonic	VWA5B1		nonsynonymous SNV	VWA5B1:NM_001039500:exon15:c.G2199	NOVEL		61	28	
34	chr1	228466544	228466544	C	T	exonic	OBSCN		synonymous SNV	OBSCN:NM_001098623:exon26:c.C7014T:	NOVEL		241	1	
34	chr10	23482192	23482192	G	T	exonic	PTF1A		nonsynonymous SNV	PTF1A:NM_178161:exon1:c.G733T:p.D245	NOVEL		18	16	
34	chr5	140039725	140039725	C	T	exonic	IK		stopgain	IK:NM_006083:exon15:c.C1300T:p.Q434X	NOVEL		110	34	
34	chr1	240072107	240072107	C	T	exonic	CHRM3		synonymous SNV	CHRM3:NM_000740:exon5:c.C1356T:p.A4	NOVEL		70	92	
34	chr2	70315483	70315483	A	G	exonic	PCBP1		nonsynonymous SNV	PCBP1:NM_006196:exon1:c.A608G:p.D203	NOVEL		46	44	
34	chr4	20543180	20543180	C	G	exonic	SLIT2		nonsynonymous SNV	SLIT2:NM_001289135:exon19:c.C2069G:p.	NOVEL		139	78	
34	chr3	156710889	156710889	A	T	exonic	LEKR1		nonsynonymous SNV	LEKR1:NM_001004316:exon9:c.A932T:p.E	NOVEL		54	18	
34	chr19	9074884	9074884	G	T	exonic	MUC16		nonsynonymous SNV	MUC16:NM_024690:exon3:c.C12562A:p.P	COSMIC	ID=COSM684073	263	0	
34	chr5	74931684	74931684	C	T	exonic	ANKDD1B		stopgain	ANKDD1B:NM_001276713:exon7:c.C775T:	NOVEL		223	99	
34	chr19	51628896	51628896	A	G	exonic	SIGLEC9		nonsynonymous SNV	SIGLEC9:NM_001198558:exon2:c.A464G:p	NOVEL		173	122	
34	chr7	27805592	27805592	A	G	exonic	TAX1BP1		synonymous SNV	TAX1BP1:NM_001079864:exon4:c.A405G:	NOVEL		463	261	
34	chr19	36883926	36883926	T	A	exonic	ZFP82		nonsynonymous SNV	ZFP82:NM_001321917:exon5:c.A1319T:p.	NOVEL		205	77	
34	chr7	96653912	96653912	C	G	exonic	DLX5		nonsynonymous SNV	DLX5:NM_005221:exon1:c.G24C:p.R8S	NOVEL		189	37	
34	chr1	223933083	223933083	G	T	exonic	CAPN2		nonsynonymous SNV	CAPN2:NM_001146068:exon4:c.G268T:p.V	NOVEL		104	76	
34	chr8	113933882	113933882	C	G	exonic	CSMD3		nonsynonymous SNV	CSMD3:NM_001363185:exon8:c.G1217C:p	NOVEL		187	97	
34	chr1	16255044	16255044	C	T	exonic	SPEN		nonsynonymous SNV	SPEN:NM_015001:exon11:c.C2309T:p.S77	COSMIC	ID=COSM136095	128	78	
34	chr20	49209652	49209652	G	T	exonic	RIPOR3		nonsynonymous SNV	RIPOR3:NM_001290268:exon18:c.C2294A	NOVEL		73	49	
34	chr18	28966691	28966691	A	C	exonic	DSG4		nonsynonymous SNV	DSG4:NM_001134453:exon3:c.A125C:p.Q	NOVEL		349	0	
34	chr12	113443012	113443012	G	T	exonic	OAS2		nonsynonymous SNV	OAS2:NM_002535:exon7:c.G1453T:p.A48	NOVEL		69	40	
34	chr18	9257736	9257736	C	T	exonic	ANKRD12		nonsynonymous SNV	ANKRD12:NM_001083625:exon8:c.C4402T	NOVEL		162	77	
34	chr18	44030689	44030689	G	T	exonic	RNF165		nonsynonymous SNV	RNF165:NM_001256758:exon4:c.G167T:p	NOVEL		50	30	
34	chr17	16346946	16346946	C	A	exonic	LRRC75A		nonsynonymous SNV	LRRC75A:NM_001113567:exon4:c.G991T:	NOVEL		60	37	
34	chr3	11070512	11070512	G	C	exonic	SLC6A1		nonsynonymous SNV	SLC6A1:NM_001348252:exon10:c.G636C:p	NOVEL		86	23	
34	chr11	108123623	108123623	C	G	exonic	ATM		nonsynonymous SNV	ATM:NM_000051:exon12:c.C1882G:p.Q62	NOVEL		83	34	
34	chr14	47120814	47120814	T	C	exonic	RPL10L		synonymous SNV	RPL10L:NM_080746:exon1:c.A126G:p.K42	DBSNP		316	1	
34	chr1	152192163	152192163	G	T	exonic	HRNR		nonsynonymous SNV	HRNR:NM_001009931:exon3:c.C1942A:p.	DBSNP+COSMIC	ID=COSM619195	114	22	
34	chr2	197184194	197184194	G	T	exonic	HECW2		nonsynonymous SNV	HECW2:NM_001304840:exon7:c.C352A:p.	NOVEL		186	1	
34	chr6	146350874	146350874	A	G	exonic	GRM1		nonsynonymous SNV	GRM1:NM_001278064:exon1:c.A221G:p.Y	NOVEL		130	0	
34	chr7	150884802	150884802	C	T	exonic	ASB10		stopgain	ASB10:NM_080871:exon1:c.G8A:p.W3X	NOVEL		110	21	
34	chr10	46970790	46970790	T	G	exonic	LOC102724488		nonsynonymous SNV	LOC102724488:NM_001370182:exon1:c.A	DBSNP		26	5	
34	chr9	37503078	37503078	C	G	exonic	POLR1E		nonsynonymous SNV	POLR1E:NM_022490:exon12:c.C1139G:p.S	NOVEL		92	0	
34	chr20	57288997	57288997	G	A	exonic	NPEPL1		nonsynonymous SNV	NPEPL1:NM_024663:exon10:c.G1150A:p.A	NOVEL		63	0	
34	chr20	44573541	44573541	C	T	exonic	PCIF1		nonsynonymous SNV	PCIF1:NM_022104:exon11:c.C1040T:p.P34	COSMIC	ID=COSM168172	108	0	
34	chr11	12225897	12225897	C	T	exonic	MICAL2		nonsynonymous SNV	MICAL2:NM_001282666:exon2:c.C365T:p.S	NOVEL		314	0	
34	chr3	130293041	130293041	C	A	exonic	COL6A6		synonymous SNV	COL6A6:NM_001102608:exon7:c.C3219A:p	COSMIC	ID=COSM260249	259	0	
34	chr14	60933684	60933684	C	A	exonic	C14orf39		nonsynonymous SNV	C14orf39:NM_174978:exon10:c.G846T:p.C	COSMIC	ID=COSM119809	96	0	
34	chr15	31776257	31776257	C	T	exonic	OTUD7A		nonsynonymous SNV	OTUD7A:NM_130901:exon14:c.G2021A:p.	NOVEL		321	0	
34	chr8	125579406	125579406	G	C	exonic	MTSS1		stopgain	MTSS1:NM_001282974:exon8:c.C632G:p.S	NOVEL		120	0	
34	chr3	43591249	43591249	G	C	exonic	ANO10		stopgain	ANO10:NM_001204833:exon9:c.C1427G:p	DBSNP		397	0	
34	chr10	90572964	90572964	G	A	exonic	LIPM		nonsynonymous SNV	LIPM:NM_001128215:exon3:c.G376A:p.D1	NOVEL		144	0	
34	chr3	37034368	37034368	C	T	exonic	EPM2AIP1		synonymous SNV	EPM2AIP1:NM_014805:exon1:c.G201A:p.R	NOVEL		355	0	
34	chr12	52201135	52201135	G	A	exonic	SCN8A		synonymous SNV	SCN8A:NM_001177984:exon26:c.G5742A:	NOVEL		131	0	
34	chr4	46930550	46930550	A	G	exonic	GABRA4		nonsynonymous SNV	GABRA4:NM_001204267:exon8:c.T1147C:	NOVEL		150	0	
34	chr2	201724890	201724890	C	T	exonic	CLK1		nonsynonymous SNV	CLK1:NM_001162407:exon4:c.G565A:p.G1	NOVEL		237	0	
34	chr15	43820355	43820355	C	A	exonic	MAP1A		synonymous SNV	MAP1A:NM_002373:exon4:c.C6684A:p.S2	NOVEL		666	0	
34	chr18	56182304	56182304	C	T	exonic	ALPK2		nonsynonymous SNV	ALPK2:NM_052947:exon10:c.G5950A:p.V1	NOVEL		322	0	
34	chr1	44595458	44595458	C	T	exonic	KLF17		nonsynonymous SNV	KLF17:NM_173484:exon2:c.C515T:p.T172I	DBSNP		252	0	
34	chr2	209188948	209188948	G	A	exonic	PIKFYVE		nonsynonymous SNV	PIKFYVE:NM_015040:exon18:c.G2273A:p.	DBSNP+COSMIC	ID=COSM674664	429	0	
34	chr22	50018204	50018204	G	T	exonic	C22orf34		nonsynonymous SNV	C22orf34:NM_001289922:exon4:c.C257A:p	NOVEL		306	0	
34	chr16	84520586	84520586	G	T	exonic	MEAK7		synonymous SNV	MEAK7:NM_020947:exon5:c.C609A:p.V20	NOVEL		80	5	
34	chr11	107299966	107299966	T	G	exonic	CWF19L2		nonsynonymous SNV	CWF19L2:NM_152434:exon8:c.A992C:p.K3	DBSNP+COSMIC	ID=COSM256711	119	0	
34	chr6	90642487	90642487	C	A	exonic	BACH2		nonsynonymous SNV	BACH2:NM_001170794:exon7:c.G2166T:p	NOVEL		209	8	
34	chr19	42753239	42753239	G	C	exonic	ERF		nonsynonymous SNV	ERF:NM_001301035:exon4:c.C800G:p.P26	NOVEL		120	0	
34	chr19	33616091	33616091	A	T	exonic	GPATCH1		nonsynonymous SNV	GPATCH1:NM_018025:exon18:c.A2602T:p	NOVEL		435	0	
34	chr6	75899067	75899067	G	A	exonic	COL12A1		nonsynonymous SNV	COL12A1:NM_004370:exon7:c.C689T:p.T2	NOVEL		221	0	
34	chr11	49075679	49075679	C	T	exonic	TRIM64C		nonsynonymous SNV	TRIM64C:NM_001206631:exon6:c.G940A:	NOVEL		220	0	
34	chr5	170235748	170235748	C	A	exonic	GABRP		nonsynonymous SNV	GABRP:NM_001291985:exon8:c.C824A:p.T	NOVEL		335	0	
34	chr7	72891397	72891397	C	A	exonic	BAZ1B		synonymous SNV	BAZ1B:NM_001370402:exon7:c.G2394T:p	NOVEL		612	0	
34	chr8	123964824	123964824	G	A	exonic	ZHX2		synonymous SNV	ZHX2:NM_014943:exon3:c.G1074A:p.T358	DBSNP+COSMIC	ID=COSM316599	514	0	
34	chr7	71177024	71177024	G	A	exonic	GALNT17		nonsynonymous SNV	GALNT17:NM_022479:exon11:c.G1690A:p	NOVEL		465	0	
34	chr8	144103125	144103125	C	G	exonic	LY6E		synonymous SNV	LY6E:NM_001127213:exon4:c.C315G:p.G1	NOVEL		223	0	
34	chr4	15989338	15989338	C	A	exonic	PROM1		nonsynonymous SNV	PROM1:NM_001145851:exon18:c.G2051T	NOVEL		485	0	
34	chr7	20418862	20418862	G	C	exonic	ITGB8		nonsynonymous SNV	ITGB8:NM_002214:exon4:c.G577C:p.D193	NOVEL		704	0	
34	chr2	168103870	168103870	T	G	exonic	XIRP2		nonsynonymous SNV	XIRP2:NM_001199144:exon7:c.T5302G:p.V	NOVEL		178	0	
34	chr17	67286038	67286038	T	C	exonic	ABCA5		nonsynonymous SNV	ABCA5:NM_018672:exon12:c.A1747G:p.I5	NOVEL		104	0	
34	chr12	51138413	51138413	C	G	exonic	DIP2B		nonsynonymous SNV	DIP2B:NM_173602:exon38:c.C4522G:p.L1	NOVEL		217	0	
34	chr5	127668650	127668650	C	A	exonic	FBN2		nonsynonymous SNV	FBN2:NM_001999:exon32:c.G4176T:p.K13	NOVEL		603	1	
34	chr21	43711719	43711719	G	A	exonic	ABCG1		nonsynonymous SNV	ABCG1:NM_004915:exon13:c.G1642A:p.A	NOVEL		283	0	
34	chr14	20666170	20666170	A	T	exonic	OR11G2		nonsynonymous SNV	OR11G2:NM_001005503:exon1:c.A676T:p	NOVEL		493	0	
34	chr14	64194042	64194042	G	A	exonic	SGPP1		synonymous SNV	SGPP1:NM_030791:exon1:c.C621T:p.T207	DBSNP		229	0	
34	chr2	169759036	169759036	G	A	exonic	G6PC2		synonymous SNV	G6PC2:NM_001081686:exon2:c.G300A:p.C	NOVEL		492	0	
34	chr7	112412901	112412901	C	G	exonic	TMEM168		nonsynonymous SNV	TMEM168:NM_022484:exon4:c.G1481C:p.	NOVEL		638	0	
34	chr21	42622762	42622762	T	C	exonic	BACE2		synonymous SNV	BACE2:NM_012105:exon7:c.T1068C:p.P35	NOVEL		309	0	
34	chr9	2645075	2645075	G	A	exonic	VLDLR		synonymous SNV	VLDLR:NM_001322225:exon8:c.G1182A:p.	NOVEL		351	0	
34	chr16	29998978	29998978	G	A	exonic	TAOK2		nonsynonymous SNV	TAOK2:NM_016151:exon16:c.G3385A:p.V	DBSNP		547	11	
34	chr1	222700376	222700376	C	A	exonic	HHIPL2		synonymous SNV	HHIPL2:NM_024746:exon7:c.G1740T:p.L58	NOVEL		268	0	
34	chr2	101646141	101646141	G	A	exonic	TBC1D8		synonymous SNV	TBC1D8:NM_001102426:exon12:c.C1989T	NOVEL		273	0	
34	chr19	54822826	54822826	G	T	exonic	LILRA5		synonymous SNV	LILRA5:NM_181985:exon4:c.C534A:p.T178	NOVEL		260	0	
34	chr11	62343262	62343262	C	T	exonic	TUT1		synonymous SNV	TUT1:NM_022830:exon9:c.G1929A:p.R64	NOVEL		323	0	
34	chr12	52284672	52284672	C	T	exonic	ANKRD33		synonymous SNV	ANKRD33:NM_001304460:exon4:c.C360T:	NOVEL		447	0	
34	chr16	50827469	50827469	G	A	exonic	CYLD		nonsynonymous SNV	CYLD:NM_001042355:exon16:c.G2354A:p.	NOVEL		211	0	
34	chr17	46846376	46846376	C	T	exonic	TTLL6		nonsynonymous SNV	TTLL6:NM_173623:exon8:c.G1730A:p.G57	NOVEL		112	7	
34	chr7	148875753	148875753	C	T	exonic	ZNF398		synonymous SNV	ZNF398:NM_170686:exon6:c.C789T:p.V26	NOVEL		246	0	
34	chr3	49060391	49060391	T	C	exonic	NDUFAF3		synonymous SNV	NDUFAF3:NM_199069:exon4:c.T420C:p.A	NOVEL		208	14	
34	chr19	44130310	44130310	G	C	exonic	CADM4		nonsynonymous SNV	CADM4:NM_145296:exon5:c.C630G:p.H21	NOVEL		353	23	
34	chr4	5333020	5333020	C	T	exonic	STK32B		synonymous SNV	STK32B:NM_001345969:exon4:c.C334T:p.	NOVEL		195	0	
34	chr4	11401516	11401516	C	T	exonic	HS3ST1		synonymous SNV	HS3ST1:NM_005114:exon2:c.G114A:p.Q38	NOVEL		162	0	
34	chr3	89448536	89448536	G	A	exonic	EPHA3		synonymous SNV	EPHA3:NM_005233:exon7:c.G1500A:p.K50	DBSNP		257	0	
34	chr21	46313365	46313365	C	T	exonic	ITGB2		nonsynonymous SNV	ITGB2:NM_000211:exon10:c.G1178A:p.R3	NOVEL		152	0	
34	chr12	48189036	48189036	G	A	exonic	HDAC7		synonymous SNV	HDAC7:NM_001098416:exon10:c.C1104T:p	DBSNP+COSMIC	ID=COSM468970	147	0	
34	chr1	169845154	169845154	C	T	exonic	SCYL3		nonsynonymous SNV	SCYL3:NM_020423:exon4:c.G430A:p.E144	NOVEL		472	1	
34	chr10	73767717	73767717	C	T	exonic	CHST3		nonsynonymous SNV	CHST3:NM_004273:exon3:c.C928T:p.R310	COSMIC	ID=COSM156124	40	5	
34	chr3	32188135	32188135	G	A	exonic	GPD1L		nonsynonymous SNV	GPD1L:NM_015141:exon5:c.G527A:p.G17	NOVEL		429	15	
34	chr1	145530952	145530952	G	A	exonic	ITGA10		synonymous SNV	ITGA10:NM_001303041:exon3:c.G255A:p.	NOVEL		118	17	
34	chr1	204218388	204218388	A	G	exonic	PLEKHA6		nonsynonymous SNV	PLEKHA6:NM_014935:exon11:c.T1598C:p.	NOVEL		560	16	
34	chr4	73184314	73184314	C	A	exonic	ADAMTS3		nonsynonymous SNV	ADAMTS3:NM_014243:exon10:c.G1460T:p	NOVEL		315	17	
34	chr6	41767588	41767588	C	T	exonic	USP49		synonymous SNV	USP49:NM_001286554:exon4:c.G1650A:p	COSMIC	ID=COSM378206	199	11	
34	chr5	13766141	13766141	A	C	exonic	DNAH5		nonsynonymous SNV	DNAH5:NM_001369:exon59:c.T10045G:p.	NOVEL		596	57	
34	chr5	5463584	5463584	G	A	exonic	ICE1		synonymous SNV	ICE1:NM_015325:exon13:c.G4137A:p.E137	NOVEL		707	75	
34	chr11	55541309	55541309	G	T	exonic	OR5D13		nonsynonymous SNV	OR5D13:NM_001001967:exon1:c.G396T:p	COSMIC	ID=COSM457444	373	26	
34	chr19	19379445	19379445	G	A	exonic	TM6SF2		synonymous SNV	TM6SF2:NM_001001524:exon6:c.C603T:p.	NOVEL		125	27	
34	chr9	107583782	107583782	A	G	exonic	ABCA1		nonsynonymous SNV	ABCA1:NM_005502:exon20:c.T2834C:p.I9	NOVEL		65	19	
34	chr1	182872264	182872264	T	A	exonic	SHCBP1L		synonymous SNV	SHCBP1L:NM_030933:exon9:c.A1620T:p.I5	NOVEL		93	20	
34	chr7	144095425	144095425	G	T	exonic	NOBOX		nonsynonymous SNV	NOBOX:NM_001080413:exon9:c.C1724A:p	NOVEL		236	30	
34	chr12	107245362	107245362	C	G	exonic	RIC8B		nonsynonymous SNV	RIC8B:NM_001330146:exon6:c.C1248G:p.	NOVEL		31	10	
34	chr12	107245360	107245360	T	A	exonic	RIC8B		nonsynonymous SNV	RIC8B:NM_001330146:exon6:c.T1246A:p.	NOVEL		33	10	
34	chr12	112623067	112623067	C	T	exonic	HECTD4		nonsynonymous SNV	HECTD4:NM_001109662:exon61:c.G9733A	DBSNP		192	56	
34	chr4	660349	660349	G	C	exonic	PDE6B		nonsynonymous SNV	PDE6B:NM_001350155:exon16:c.G1143C:	NOVEL		236	67	
34	chr12	41966595	41966595	G	T	exonic	PDZRN4		stopgain	PDZRN4:NM_013377:exon8:c.G1240T:p.E	DBSNP		181	51	
34	chr7	13950893	13950893	C	A	exonic	ETV1		nonsynonymous SNV	ETV1:NM_001163151:exon5:c.G668T:p.G	DBSNP+COSMIC	ID=COSM727355	461	48	
34	chr15	75643707	75643707	C	G	exonic	NEIL1		synonymous SNV	NEIL1:NM_001352519:exon3:c.C165G:p.L5	NOVEL		155	19	
34	chr17	8296567	8296567	G	C	exonic	RNF222		nonsynonymous SNV	RNF222:NM_001146684:exon3:c.C213G:p	NOVEL		152	38	
34	chr6	76712668	76712668	C	G	exonic	IMPG1		nonsynonymous SNV	IMPG1:NM_001282368:exon11:c.G1024C:	NOVEL		181	41	
34	chr14	31774330	31774330	C	T	exonic	HEATR5A		nonsynonymous SNV	HEATR5A:NM_015473:exon32:c.G5020A:p	NOVEL		208	47	
34	chr11	62602989	62602989	G	A	exonic	WDR74		synonymous SNV	WDR74:NM_001307977:exon6:c.C532T:p.	NOVEL		155	35	
34	chr6	101246703	101246703	T	C	exonic	ASCC3		synonymous SNV	ASCC3:NM_001284271:exon8:c.A1281G:p.	NOVEL		89	20	
34	chr16	239271	239271	C	G	exonic	LUC7L		nonsynonymous SNV	LUC7L:NM_201412:exon10:c.G1042C:p.D3	NOVEL		220	49	
34	chr4	36122921	36122921	G	T	exonic	ARAP2		nonsynonymous SNV	ARAP2:NM_015230:exon23:c.C3774A:p.H1	NOVEL		224	49	
34	chr6	43305161	43305161	G	T	exonic	ZNF318		nonsynonymous SNV	ZNF318:NM_014345:exon10:c.C6575A:p.S	NOVEL		103	22	
34	chr12	120576140	120576140	T	C	exonic	GCN1		nonsynonymous SNV	GCN1:NM_006836:exon47:c.A6236G:p.Y2	NOVEL		187	39	
34	chr5	176825158	176825158	C	T	exonic	SLC34A1		synonymous SNV	SLC34A1:NM_003052:exon13:c.C1791T:p.T	NOVEL		427	57	
34	chr2	70071265	70071265	G	A	exonic	GMCL1		nonsynonymous SNV	GMCL1:NM_178439:exon6:c.G695A:p.C23	NOVEL		33	4	
34	chr19	1775362	1775362	G	A	exonic	ONECUT3		nonsynonymous SNV	ONECUT3:NM_001080488:exon2:c.G1403	NOVEL		200	10	
34	chr5	108516561	108516561	A	C	exonic	FER		nonsynonymous SNV	FER:NM_001308031:exon9:c.A1055C:p.Q3	NOVEL		281	14	
34	chr2	171243649	171243649	C	G	exonic	MYO3B		nonsynonymous SNV	MYO3B:NM_001083615:exon14:c.C1408G	NOVEL		222	14	
34	chr12	123106492	123106492	G	A	exonic	KNTC1		nonsynonymous SNV	KNTC1:NM_014708:exon61:c.G6344A:p.G	NOVEL		268	16	
34	chr6	35840345	35840345	G	T	exonic	SRPK1		nonsynonymous SNV	SRPK1:NM_003137:exon8:c.C746A:p.S249	COSMIC	ID=COSM107844	124	7	
34	chrX	47044605	47044605	T	A	splicing	RBM10	NM_001204468:exon1			NOVEL		11	74	NA
34	chr18	72515919	72515919	G	T	splicing	ZNF407	NM_001146190:exon4			NOVEL		488	0	NA
34	chr7	23293933	23293933	T	G	splicing	GPNMB	NM_002510:exon3:c.3			NOVEL		306	57	
34	chr9	133260946	133260946	G	C	splicing	HMCN2	NM_001291815:exon4			NOVEL		112	52	
34	chr7	37283997	37283997	C	T	splicing	ELMO1	NM_001206480:exon7			NOVEL		280	52	
34	chr2	25468121	25468121	C	T	splicing	DNMT3A	NM_001320893:exon8			DBSNP+COSMIC	ID=COSM477512	157	50	
35	chr2	242396291	242396291	G	A	exonic	FARP2		nonsynonymous SNV	FARP2:NM_001282983:exon14:c.G1541A:	NOVEL		100	0	NA
35	chr10	134647613	134647613	G	A	exonic	CFAP46		nonsynonymous SNV	CFAP46:NM_001200049:exon49:c.C6881T:	DBSNP+COSMIC	ID=COSM473720	49	55	
35	chr16	4606514	4606514	C	T	exonic	C16orf96		synonymous SNV	C16orf96:NM_001145011:exon1:c.C24T:p.	DBSNP		18	16	
35	chr7	33063710	33063710	T	C	exonic	NT5C3A		nonsynonymous SNV	NT5C3A:NM_001002010:exon3:c.A248G:p	NOVEL		36	31	
35	chr1	39322557	39322557	G	A	exonic	RRAGC		synonymous SNV	RRAGC:NM_001271851:exon2:c.C333T:p.D	DBSNP		62	50	
35	chr11	75152828	75152828	C	G	exonic	GDPD5		nonsynonymous SNV	GDPD5:NM_001351167:exon9:c.G774C:p.R	NOVEL		94	64	
35	chr11	75152829	75152829	C	T	exonic	GDPD5		nonsynonymous SNV	GDPD5:NM_001351167:exon9:c.G773A:p.R	NOVEL		94	64	
35	chr1	42048831	42048831	G	A	exonic	HIVEP3		synonymous SNV	HIVEP3:NM_001127714:exon3:c.C1638T:p	DBSNP+COSMIC	ID=COSM399413	43	33	
35	chr22	28193059	28193059	G	A	exonic	MN1		nonsynonymous SNV	MN1:NM_002430:exon1:c.C3473T:p.A115	NOVEL		71	54	
35	chr16	16180730	16180730	C	T	exonic	ABCC1		nonsynonymous SNV	ABCC1:NM_004996:exon18:c.C2342T:p.A7	NOVEL		25	19	
35	chr15	54919140	54919140	C	T	exonic	UNC13C		synonymous SNV	UNC13C:NM_001080534:exon31:c.C6474T	NOVEL		342	152	
35	chr11	78381093	78381093	G	A	exonic	TENM4		synonymous SNV	TENM4:NM_001098816:exon32:c.C6297T:	DBSNP+COSMIC	ID=COSM135722	140	88	
35	chr19	8122713	8122713	T	C	exonic	CCL25		synonymous SNV	CCL25:NM_001201359:exon5:c.T351C:p.S1	NOVEL		187	81	

35	chr17	1303355	1303355	G	A	exonic	YWHAE		nonsynonymous SNV	YWHAE:NM_006761:exon1:c.C50T:p.A17V	NOVEL		136	96	1.03572	1	264	174	1.10716	1
35	chr20	10394024	10394024	G	A	exonic	MKKS		nonsynonymous SNV	MKKS:NM_018848:exon3:c.C139T:p.H47Y,	NOVEL		69	48	1.02687	1	134	165	1.0053	2
35	chr13	23913082	23913082	G	A	exonic	SACS		stopgain	SACS:NM_001278055:exon8:c.C4492T:p.R	DBSNP+COSMIC	ID=COSM404656	44	86	0.994286	1	115	305	1.01194	2
35	chr18	55024546	55024546	A	T	exonic	ST8SIA3		nonsynonymous SNV	ST8SIA3:NM_015879:exon3:c.A705T:p.L23	NOVEL		179	115	0.979061	1	376	191	0.938825	1
35	chr3	40529708	40529708	G	A	exonic	ZNF619		synonymous SNV	ZNF619:NM_001145083:exon4:c.G1575A:	DBSNP		96	61	0.972499	1	183	106	1.02221	1
35	chr19	2041859	2041859	C	T	exonic	MKNK2		nonsynonymous SNV	MKNK2:NM_017572:exon11:c.G925A:p.E3	NOVEL		27	33	0.963322	2	72	105	1.12326	2
35	chr19	4510709	4510709	G	T	exonic	PLIN4		nonsynonymous SNV	PLIN4:NM_001367868:exon5:c.C3263A:p.F	NOVEL		125	149	0.952455	2	237	259	0.988738	2
35	chr4	187539803	187539803	A	C	exonic	FAT1		nonsynonymous SNV	FAT1:NM_005245:exon10:c.T7937G:p.L26	NOVEL		87	53	0.94756	1	154	94	1.05636	1
35	chr5	43616023	43616023	T	A	exonic	NNT		nonsynonymous SNV	NNT:NM_001331026:exon3:c.T62A:p.I21N	NOVEL		205	76	0.947428	1	356	146	1.10139	1
35	chr5	43616035	43616035	A	G	exonic	NNT		nonsynonymous SNV	NNT:NM_001331026:exon3:c.A74G:p.Y25	NOVEL		215	79	0.94128	1	371	146	1.06944	1
35	chr2	84912579	84912579	T	C	exonic	DNAH6		nonsynonymous SNV	DNAH6:NM_001370:exon43:c.T7046C:p.I2	NOVEL		35	21	0.938621	1	59	37	1.07415	1
35	chr4	5170142	5170142	G	A	exonic	STK32B		nonsynonymous SNV	STK32B:NM_001345969:exon3:c.G225A:p.	NOVEL		40	23	0.91379	1	68	41	1.04831	1
35	chr4	62849318	62849318	A	C	exonic	ADGRL3		nonsynonymous SNV	ADGRL3:NM_001371342:exon15:c.A2975C	NOVEL		54	31	0.912855	1	77	47	1.05636	1
35	chr4	6303645	6303645	G	A	exonic	WFS1		nonsynonymous SNV	WFS1:NM_001145853:exon8:c.G2123A:p.	DBSNP		135	77	0.909105	1	258	163	1.07905	1
35	chr6	112437118	112437118	T	C	exonic	LAMA4		nonsynonymous SNV	LAMA4:NM_001105206:exon36:c.A5060G	NOVEL		131	74	0.903518	1	246	134	0.982778	1
35	chr1	207297584	207297584	C	T	exonic	C4BPA		synonymous SNV	C4BPA:NM_000715:exon6:c.C579T:p.Y193	DBSNP		55	31	0.902241	1	109	70	1.08988	1
35	chr16	3017054	3017054	C	T	exonic	KREMEN2		nonsynonymous SNV	KREMEN2:NM_001253725:exon6:c.C667T:	NOVEL		70	39	0.895565	1	112	70	1.07192	1
35	chr9	139230555	139230555	C	A	exonic	GPSM1		nonsynonymous SNV	GPSM1:NM_001145638:exon3:c.C423A:p.	NOVEL		124	31	0.891506	1	189	45	0.887399	1
35	chr7	86688667	86688667	T	C	exonic	KIAA1324L		nonsynonymous SNV	KIAA1324L:NM_001142749:exon1:c.A163C	NOVEL		29	15	0.853292	1	46	32	1.14338	1
35	chr3	146177862	146177862	C	T	exonic	PLSCR2		nonsynonymous SNV	PLSCR2:NM_001199979:exon2:c.G37A:p.G	DBSNP		64	33	0.851533	1	124	59	0.898535	1
35	chr1	233150516	233150516	G	A	exonic	PCNX2		nonsynonymous SNV	PCNX2:NM_014801:exon28:c.C4847T:p.T1	DBSNP		32	16	0.83433	1	42	45	1.44154	1
35	chr20	57466865	57466865	G	A	exonic	GNAS		synonymous SNV	GNAS:NM_000516:exon1:c.G84A:p.K28K,G	DBSNP		74	37	0.83433	1	190	64	0.918034	1
35	chr15	40661736	40661736	G	T	exonic	DISP2		synonymous SNV	DISP2:NM_033510:exon8:c.G3423T:p.G11	NOVEL		98	30	0.821013	1	262	61	0.90167	1
35	chr6	129959580	129959580	C	A	exonic	ARHGAP18		nonsynonymous SNV	ARHGAP18:NM_033515:exon3:c.G511T:p.	NOVEL		90	42	0.796406	1	150	91	1.05235	1
35	chr2	183582886	183582886	T	C	exonic	DNAJC10		nonsynonymous SNV	DNAJC10:NM_001271581:exon3:c.T73C:p.	NOVEL		24	11	0.786654	1	47	25	0.967702	1
35	chr1	237791219	237791219	C	T	exonic	RYR2		synonymous SNV	RYR2:NM_001035:exon41:c.C6279T:p.D20	COSMIC	ID=COSM340051	112	42	0.682634	1	310	11	0.095504	1
35	chr11	20622931	20622931	C	T	exonic	SLC6A5		nonsynonymous SNV	SLC6A5:NM_004211:exon2:c.C260T:p.A87	COSMIC	ID=COSM925912	54	20	0.676484	1	153	4	0.0924192	1
35	chr2	158142601	158142601	C	T	exonic	GALNT5		nonsynonymous SNV	GALNT5:NM_014568:exon3:c.C1696T:p.H5	NOVEL		137	48	0.649424	1	331	8	0.0657695	1
35	chr3	53220659	53220659	G	C	exonic	PRKCD		nonsynonymous SNV	PRKCD:NM_001354676:exon13:c.G1357C:	NOVEL		246	70	0.55446	1	570	20	0.094474	1
35	chr10	61823947	61823947	T	C	exonic	ANK3		nonsynonymous SNV	ANK3:NM_001149:exon15:c.A1982G:p.K6	NOVEL		34	8	0.47676	1	86	2	0.0633405	1
35	chr10	91143502	91143502	G	T	exonic	IFIT1B		nonsynonymous SNV	IFIT1B:NM_001010987:exon2:c.G432T:p.E	NOVEL		100	17	0.363682	1	249	0	0	1
35	chrX	48547221	48547221	A	G	exonic	WAS		synonymous SNV	WAS:NM_000377:exon10:c.A1104G:p.P36	NOVEL		10	4	0.35757	1	17	24	0.815702	1
35	chr9	135153524	135153524	T	C	exonic	SETX		nonsynonymous SNV	SETX:NM_001351527:exon21:c.A6775G:p.	NOVEL		158	12	0.314649	1	292	0	0	1
35	chr9	35825891	35825891	A	C	exonic	FAM221B		nonsynonymous SNV	FAM221B:NM_001012446:exon2:c.T268G	NOVEL		194	14	0.300026	1	357	0	0	1
35	chr1	115256529	115256529	T	C	exonic	NRAS		nonsynonymous SNV	NRAS:NM_002524:exon3:c.A182G:p.Q61R	DBSNP+COSMIC	ID=COSM584;OC	125	16	0.284027	1	171	82	0.903291	1
35	chr22	38122043	38122043	C	G	exonic	TRIOBP		nonsynonymous SNV	TRIOBP:NM_001039141:exon7:c.C3480G:	NOVEL		290	36	0.276404	1	494	0	0	1
35	chr5	68805092	68805092	A	G	exonic	OCLN		nonsynonymous SNV	OCLN:NM_001205254:exon3:c.A175G:p.T	NOVEL		252	19	0.245597	1	358	136	1.04257	1
35	chr21	40186202	40186202	G	A	exonic	ETS2		nonsynonymous SNV	ETS2:NM_005239:exon4:c.G190A:p.A64T,	DBSNP		186	14	0.245209	1	378	0	0	1
35	chr19	58718558	58718558	T	G	exonic	ZNF274		unknown	UNKNOWN	NOVEL		94	7	0.242781	1	142	2	0.052597	1
35	chr5	40681138	40681138	G	T	exonic	PTGER4		nonsynonymous SNV	PTGER4:NM_000958:exon2:c.G43T:p.D15	NOVEL		415	30	0.236157	1	631	227	1.00192	1
35	chr1	237013711	237013711	G	C	exonic	MTR		nonsynonymous SNV	MTR:NM_001291940:exon15:c.G362C:p.G	NOVEL		371	38	0.232552	1	435	218	0.930417	1
35	chr11	55703257	55703257	G	T	exonic	OR5I1		stopgain	OR5I1:NM_006637:exon1:c.C620A:p.S207	NOVEL		157	16	0.23149	1	411	13	NA	1
35	chr9	112017941	112017941	G	A	exonic	EPB41L4B		nonsynonymous SNV	EPB41L4B:NM_018424:exon11:c.C1019T:p	DBSNP+COSMIC	ID=COSM316909	139	6	0.184449	1	195	55	1.01518	1
35	chr3	39140350	39140350	G	A	exonic	GORASP1		synonymous SNV	GORASP1:NM_001278790:exon5:c.C486T:	NOVEL		216	17	0.182622	1	338	10	0.0800857	1
35	chr4	186283143	186283143	T	C	exonic	SNX25		nonsynonymous SNV	SNX25:NM_001317781:exon17:c.T2225C:	NOVEL		286	20	0.163594	1	578	13	0.0613042	1
35	chr10	13040491	13040491	G	C	exonic	CCDC3		synonymous SNV	CCDC3:NM_031455:exon2:c.C396G:p.L132	NOVEL		121	8	0.155224	1	238	2	0.0232248	1
35	chr16	89261306	89261306	C	T	exonic	CDH15		nonsynonymous SNV	CDH15:NM_004933:exon14:c.C2188T:p.P7	NOVEL		94	6	0.150179	1	117	72	1.06171	1
35	chr9	112017826	112017826	G	A	exonic	EPB41L4B		synonymous SNV	EPB41L4B:NM_018424:exon11:c.C1134T:p	DBSNP		179	6	0.144568	1	277	69	0.920228	1
35	chr8	24771884	24771884	C	T	exonic	NEFM		nonsynonymous SNV	NEFM:NM_005382:exon1:c.C578T:p.A193	NOVEL		113	6	0.126201	1	239	0	0	1
35	chr20	61835104	61835104	G	T	exonic	YTHDF1		nonsynonymous SNV	YTHDF1:NM_017798:exon4:c.C188A:p.S63	DBSNP		81	4	0.117788	1	189	9	0.165611	1
35	chr4	38916686	38916686	G	A	exonic	FAM114A1		nonsynonymous SNV	FAM114A1:NM_001350631:exon6:c.G316	DBSNP+COSMIC	ID=COSM275045	194	9	0.11097	1	341	1	0.00814907	1
35	chr3	32933054	32933054	G	A	exonic	TRIM71		synonymous SNV	TRIM71:NM_001039111:exon4:c.G2358A:	DBSNP+COSMIC	ID=COSM164223	175	8	0.10942	1	325	2	0.0170458	1
35	chr4	54231866	54231866	C	A	exonic	SCFD2		synonymous SNV	SCFD2:NM_152540:exon1:c.G243T:p.L81L	NOVEL		220	10	0.108826	1	421	0	0	1
35	chr5	19571854	19571854	G	T	exonic	CDH18		nonsynonymous SNV	CDH18:NM_001167667:exon8:c.C1087A:p.	NOVEL		439	14	0.10826	1	703	18	0.0945432	1
35	chr22	43614451	43614451	C	T	exonic	SCUBE1		synonymous SNV	SCUBE1:NM_173050:exon15:c.G1701A:p.	DBSNP		91	2	0.0538277	1	165	10	0.159256	1
35	chrX	132440110	132440110	A	G	exonic	GPC4		nonsynonymous SNV	GPC4:NM_001448:exon5:c.T950C:p.V317A	NOVEL		212	8	0.0455089	1	379	0	0	1
35	chr5	7867847	7867847	C	T	exonic	FASTKD3		nonsynonymous SNV	FASTKD3:NM_024091:exon2:c.G350A:p.S1	DBSNP		269	1	0.012974	1	398	112	0.831651	1
35	chr1	31819498	31819498	G	C	exonic	ZCCHC17		nonsynonymous SNV	ZCCHC17:NM_001282566:exon5:c.G395C:	NOVEL		56	0	0	1	63	30	0.899026	1
35	chr1	32671802	32671802	A	C	exonic	IQCC		synonymous SNV	IQCC:NM_001160042:exon2:c.A330C:p.R1	NOVEL		226	0	0	1	288	133	0.880448	1
35	chr1	40131762	40131762	C	T	exonic	NT5C1A		synonymous SNV	NT5C1A:NM_032526:exon2:c.G282A:p.G9	NOVEL		84	0	0	1	121	63	0.954238	1
35	chr1	151789232	151789232	C	T	exonic	RORC		nonsynonymous SNV	RORC:NM_001001523:exon3:c.G143A:p.R	DBSNP		122	0	0	1	144	43	0.640857	1
35	chr1	154988849	154988849	C	A	exonic	ZBTB7B		nonsynonymous SNV	ZBTB7B:NM_001256455:exon3:c.C1308A:	NOVEL		317	0	0	1	421	175	0.818325	1
35	chr1	200943891	200943891	G	C	exonic	KIF21B		nonsynonymous SNV	KIF21B:NM_001252103:exon33:c.C4726G:	NOVEL		271	0	0	1	292	125	0.835426	1
35	chr10	21106600	21106600	G	A	exonic	NEBL		stopgain	NEBL:NM_006393:exon21:c.C2077T:p.Q69	DBSNP		346	0	0	1	383	173	0.867172	1
35	chr11	1158067	1158067	C	A	exonic	MUC5AC		unknown	UNKNOWN	NOVEL		219	0	0	1	366	107	0.820587	1
35	chr11	4144479	4144479	G	T	exonic	RRM1		nonsynonymous SNV	RRM1:NM_001318065:exon6:c.G171T:p.E	NOVEL		204	0	0	1	368	87	0.693601	1
35	chr11	45949529	45949529	G	A	exonic	LARGE2		nonsynonymous SNV	LARGE2:NM_001300721:exon12:c.G1663A	NOVEL		142	0	0	1	240	76	0.872426	1
35	chr11	57982685	57982685	C	T	exonic	OR1S1		nonsynonymous SNV	OR1S1:NM_001004458:exon1:c.C469T:p.L	DBSNP		229	0	0	1	485	96	0.762461	1
35	chr11	65124622	65124622	A	G	exonic	TIGD3		nonsynonymous SNV	TIGD3:NM_145719:exon2:c.A1343G:p.N44	DBSNP		202	0	0	1	449	106	0.881323	1
35	chr11	126283978	126283978	C	G	exonic	ST3GAL4		nonsynonymous SNV	ST3GAL4:NM_001348398:exon11:c.C944G	NOVEL		159	0	0	1	341	74	0.822822	1
35	chr12	13220130	13220130	G	A	exonic	FAM234B		nonsynonymous SNV	FAM234B:NM_020853:exon7:c.G1042A:p.	NOVEL		97	0	0	1	228	9	0.105835	1
35	chr14	74999235	74999235	C	A	exonic	LTBP2		nonsynonymous SNV	LTBP2:NM_000428:exon10:c.G1881T:p.L6	NOVEL		179	0	0	1	188	92	0.915723	1
35	chr15	67786689	67786689	C	T	exonic	IQCH		synonymous SNV	IQCH:NM_001322471:exon13:c.C1632T:p.	NOVEL		190	0	0	1	334	65	0.777787	1
35	chr16	28877595	28877595	C	T	exonic	SH2B1		synonymous SNV	SH2B1:NM_015503:exon1:c.C180T:p.A60A	DBSNP		209	0	0	1	238	95	0.795085	1
35	chr16	72831915	72831915	G	T	exonic	ZFHX3		nonsynonymous SNV	ZFHX3:NM_001164766:exon8:c.C1924A:p.	NOVEL		177	0	0	1	302	17	0.148523	1
35	chr17	20108722	20108722	G	A	exonic	SPECC1		nonsynonymous SNV	SPECC1:NM_001033554:exon2:c.G1117A:	NOVEL		233	0	0	1	281	116	0.814332	1
35	chr19	9922031	9922031	G	C	exonic	FBXL12		synonymous SNV	FBXL12:NM_001316936:exon2:c.C423G:p.	NOVEL		366	0	0	1	424	130	0.888642	1
35	chr2	54839352	54839352	G	A	exonic	SPTBN1		nonsynonymous SNV	SPTBN1:NM_178313:exon3:c.G316A:p.A1	NOVEL		158	0	0	1	220	100	0.870932	1
35	chr2	112786168	112786168	C	G	exonic	MERTK		synonymous SNV	MERTK:NM_006343:exon19:c.C2727G:p.R	NOVEL		287	0	0	1	458	15	0.0883821	1
35	chr3	46450597	46450597	G	C	exonic	CCRL2		nonsynonymous SNV	CCRL2:NM_001130910:exon2:c.G1063C:p.	NOVEL		232	0	0	1	276	122	0.854301	1
35	chr5	140589439	140589439	A	T	exonic	PCDHB12		nonsynonymous SNV	PCDHB12:NM_018932:exon1:c.A960T:p.Q	NOVEL		218	0	0	1	314	82	0.784173	1
35	chr6	42130909	42130909	C	G	exonic	LOC114841037		stopgain	LOC114841037:NM_001370581:exon2:c.C	NOVEL		480	0	0	1	551	205	0.755729	1
35	chr6	149919422	149919422	C	G	exonic	KATNA1		nonsynonymous SNV	KATNA1:NM_001204076:exon6:c.G725C:p	NOVEL		210	0	0	1	224	74	0.692069	1
35	chr7	150493526	150493526	C	A	exonic	TMEM176B		nonsynonymous SNV	TMEM176B:NM_001101311:exon2:c.G132	NOVEL		103	0	0	1	112	41	0.746838	1
35	chr9	107547910	107547910	C	G	exonic	ABCA1		nonsynonymous SNV	ABCA1:NM_005502:exon49:c.G6412C:p.G	NOVEL		177	0	0	1	289	37	0.523729	1
35	chr19	17443909	17443909	C	T	splicing	ANO8	NM_020959:exon4:c.4			NOVEL		172	67	0.98201	1	287	108	1.03543	1
36	chr2	242814911	242814911	C	T	exonic	RTP5		nonsynonymous SNV	RTP5:NM_173821:exon2:c.C1204T:p.H402	NOVEL		106	10	NA	1	185	6	NA	1
36	chrX	37665763	37665763	C	T	exonic	CYBB		nonsynonymous SNV	CYBB:NM_000397:exon11:c.C1438T:p.L48	NOVEL		44	6	NA	1	77	0	NA	1
36	chrX	42637037	42637037	G	C	exonic	PPP1R2C		synonymous SNV	PPP1R2C:NM_025210:exon1:c.C294G:p.V9	NOVEL		90	19	NA	1	178	1	NA	1
36	chrX	48759532	48759532	G	C	exonic	PQBP1		synonymous SNV	PQBP1:NM_005710:exon4:c.G315C:p.R10	NOVEL		154	10	NA	1	183	0	NA	1
36	chrX	50351170	50351170	G	A	exonic	SHROOM4		nonsynonymous SNV	SHROOM4:NM_020717:exon6:c.C2972T:p.	NOVEL		91	7	NA	1	191	0	NA	1
36	chrX	53458511	53458511	G	C	exonic	HSD17B10		synonymous SNV	HSD17B10:NM_001037811:exon6:c.C600G	NOVEL		91	7	NA	1	109	0	NA	1
36	chrX	69722055	69722055	T	C	exonic	DLG3		nonsynonymous SNV	DLG3:NM_001166278:exon12:c.T1037C:p.	NOVEL		53	7	NA	1	99	1	NA	1
36	chrX	37706747	37706747	C	T	exonic	DYNLT3		nonsynonymous SNV	DYNLT3:NM_006520:exon1:c.G17A:p.R6H	COSMIC	ID=COSM551378	58	2	NA	1	57	8	NA	1
36	chrX	153051693	153051693	C	G	exonic	IDH3G		nonsynonymous SNV	IDH3G:NM_004135:exon12:c.G1054C:p.V3	NOVEL		67	0	NA	1	46	37	NA	1
36	chr12	4716510	4716510	A	G	exonic	DYRK4		synonymous SNV	DYRK4:NM_001282285:exon2:c.A114G:p.F	NOVEL		155	5	0.0788618	1	146	72	NA	1
36	chr11	73076774	73076774	C	T	exonic	ARHGEF17		nonsynonymous SNV	ARHGEF17:NM_014786:exon20:c.C5777T:	COSMIC	ID=COSM732814	31	17	0.893767	1	29	32	1.35189	1
36	chr9	113547156	113547156	C	A	exonic	MUSK		synonymous SNV	MUSK:NM_001369398:exon6:c.C186A:p.S	NOVEL		492	176	1.19184	1	698	290	1.34347	1
36	chr7	5568997	5568997	T	C	exonic	ACTB		nonsynonymous SNV	ACTB:NM_001101:exon3:c.A158G:p.Y53C	NOVEL		120	96	1.12159	1	144	136	1.25171	1
36	chr19	39663721	39663721	C	G	exonic	PAK4		nonsynonymous SNV	PAK4:NM_001014832:exon3:c.C368G:p.T1	NOVEL		37	16	1.0612	1	39	18	1.12959	1
36	chr4	129012622	129012622	A	C	exonic	LARP1B		nonsynonymous SNV	LARP1B:NM_001278604:exon7:c.A623C:p.	NOVEL		34	20	0.934658	1	58	45	1.1259	1
36	chr6	75893282	75893282	C	A	exonic	COL12A1		nonsynonymous SNV	COL12A1:NM_004370:exon10:c.G1375T:p.	NOVEL		68	45	1.00496	1	111	82	1.09491	1
36	chr2	235950951	235950951	C	T	exonic	SH3BP4		nonsynonymous SNV	SH3BP4:NM_001371306:exon3:c.C1538T:	NOVEL		100	75	1.08153	1	162	117	1.0807	1
36	chr16	53404162	53404162	G	A	exonic	LOC643802		nonsynonymous SNV	LOC643802:NM_001347693:exon2:c.C316	NOVEL		41	25	0.955901	1	94	67	1.07244	1
36	chr5	140754521	140754521	A	T	exonic	PCDHGA6		nonsynonymous SNV	PCDHGA6:NM_018919:exon1:c.A871T:p.I2	NOVEL		128	48	0.960976	1	232	98	1.06228	1
36	chr1	75009635	75009635	G	A	exonic	FPGT-TNNI3K;TNN		nonsynonymous SNV	TNNI3K:NM_015978:exon25:c.G2477A:p.R	DBSNP+COSMIC	ID=COSM349223	83	70	1.15458	1	147	103	1.06174	1
36	chr12	125834134	125834134	C	A	exonic	TMEM132B		nonsynonymous SNV	TMEM132B:NM_001366854:exon2:c.C204	NOVEL		142	78	0.894723	1	185	129	1.05872	1
36	chr16	1256251	1256251	C	T	exonic	CACNA1H		synonymous SNV	CACNA1H:NM_001005407:exon12:c.C2751	DBSNP		50	1	0.0494819	1	23	16	1.05725	1
36	chr1	44468005	44468005	C	T	exonic	SLC6A9		synonymous SNV	SLC6A9:NM_001328628:exon5:c.G747A:p.	NOVEL		151	94	0.96823	1	165	114	1.05299	1
36	chr1	181732570	181732570	G	A	exonic	CACNA1E		nonsynonymous SNV	CACNA1E:NM_001205294:exon33:c.G4661	NOVEL		20	34	1.10927	2	38	53	1.04167	2
36	chr15	102359198	102359198	A	C	exonic	OR4F15		nonsynonymous SNV	OR4F15:NM_001001674:exon1:c.A809C:p.	NOVEL		208	90	1.06417	1	317	127	1.02316	1
36	chr11	3789845	3789845	G	A	exonic	NUP98		nonsynonymous SNV	NUP98:NM_001365125:exon8:c.C914T:p.T	NOVEL		63	34	0.884553	1	67	44	1.02153	1
36	chr21	45217840	45217840	C	T	exonic	RRP1		stopgain	RRP1:NM_003683:exon8:c.C670T:p.Q224X	NOVEL		176	192	0.919194	2	173	227	1.01499	2
36	chr4	62758579	62758579	C	G	exonic	ADGRL3		synonymous SNV	ADGRL3:NM_001371342:exon7:c.C1467G:	NOVEL		61	32	0.868328	1	108	69	1.00461	1
36	chr6	152749417	152749417	C	T	exonic	SYNE1		synonymous SNV	SYNE1:NM_033071:exon37:c.G4920A:p.Q	NOVEL		54	30	0.901278	1	83	53	1.00429	1
36	chr22	41833033	41833033	T	G	exonic	TOB2		nonsynonymous SNV	TOB2:NM_016272:exon2:c.A317C:p.K106T	NOVEL		250	166	1.007	1	334	210	0.994817	1
36	chr3	11301234	11301234	C	A	exonic	HRH1		nonsynonymous SNV	HRH1:NM_001098211:exon2:c.C511A:p.Q	NOVEL		109	69	0.978241	1	140	87	0.98768	1
36	chr17	17034950	17034950	G	T	exonic	MPRIP		nonsynonymous SNV	MPRIP:NM_001364716:exon5:c.G535T:p.V	NOVEL		63	50	1.11663	1	76	47	0.984726	1
36	chr5	89990397	89990397	G	C	exonic	ADGRV1		synonymous SNV	ADGRV1:NM_032119:exon33:c.G7824C:p.	NOVEL		179	60	0.88458	1	356	129	0.951421	1
36	chr11	123301617	123301617	C	A	exonic	GRAMD1B		synonymous SNV	GRAMD1B:NM_001354837:exon1:c.C117A	NOVEL		149	79	0.874398	1	159	92	0.944576	1
36	chr13	60737857	60737857	G	A	exonic	DIAPH3		nonsynonymous SNV	DIAPH3:NM_001042517:exon1:c.C44T:p.S	NOVEL		53	0	0	1	45	26	0.943708	1
36	chr20	20269355	20269355	T	G	exonic	CFAP61		nonsynonymous SNV	CFAP61:NM_015585:exon23:c.T2899G:p.F	NOVEL		235	166	1.04467	1	366	211	0.942387	1
36	chr2	241889886	241889886	C	T	exonic	CROCC2		nonsynonymous SNV	CROCC2:NM_001351305:exon18:c.C2788T	DBSNP		75	46	0.959377	1	69	39	0.930601	1
36	chr5	99897879	99897879	A	T	exonic	FAM174A		nonsynonymous SNV	FAM174A:NM_198507:exon2:c.A556T:p.N	NOVEL		124	49	0.998008	1	227	78	0.914786	1
36	chr19	45186748	45186748	G	T	exonic	CEACAM19		nonsynonymous SNV	CEACAM19:NM_001127893:exon8:c.G886	NOVEL		77	1	0.0450669	1	44	15	0.909419	1
36	chr3	195515946	195515946	G	T	exonic	MUC4		synonymous SNV	MUC4:NM_018406:exon2:c.C2505A:p.S83	NOVEL		133	85	0.983964	1	152	82	0.903069	1
36	chr1	115256530	115256530	G	T	exonic	NRAS		nonsynonymous SNV	NRAS:NM_002524:exon3:c.C181A:p.Q61K	DBSNP+COSMIC	ID=COSM580;OC	94	71	1.0859	1	142	75	0.890685	1
36	chr2	233272631	233272631	C	G	exonic	ALPG		nonsynonymous SNV	ALPG:NM_031313:exon5:c.C552G:p.N184	NOVEL		65	2	0.0753307	1	76	40	0.888638	1
36	chr1	215752474	215752474	A	G	exonic	KCTD3		nonsynonymous SNV	KCTD3:NM_001319294:exon7:c.A529G:p.R	NOVEL		187	61	0.866686	1	338	110	0.878293	1
36	chr16	88964546	88964546	G	T	exonic	CBFA2T3		nonsynonymous SNV	CBFA2T3:NM_005187:exon3:c.C319A:p.P1	NOVEL		87	0	0	1	70	36	0.875224	1
36	chr17	48050558	48050558	T	C	exonic	DLX4		synonymous SNV	DLX4:NM_001934:exon1:c.T189C:p.R63R,D	NOVEL		149	74	0.83742	1	195	100	0.873576	1
36	chr1	82456176	82456176	G	T	exonic	ADGRL2		nonsynonymous SNV	ADGRL2:NM_012302:exon20:c.G3559T:p.D	NOVEL		143	67	0.805141	1	211	108	0.872481	1
36	chr17	7916510	7916510	A	T	exonic	GUCY2D		nonsynonymous SNV	GUCY2D:NM_000180:exon11:c.A2203T:p.I	NOVEL		120	70	0.929739	1	118	59	0.859016	1
36	chr12	66773198	66773198	G	A	exonic	GRIP1		nonsynonymous SNV	GRIP1:NM_001178074:exon19:c.C2327T:p	NOVEL		100	67	1.01245	1	144	70	0.84296	1
36	chr22	39122421	39122421	G	C	exonic	GTPBP1		nonsynonymous SNV	GTPBP1:NM_004286:exon8:c.G1377C:p.Q	NOVEL		58	4	0.162811	1	67	30	0.797026	1
36	chr10	17024633	17024633	C	T	exonic	CUBN		synonymous SNV	CUBN:NM_001081:exon31:c.G4545A:p.Q1	NOVEL		59	33	0.905196	1	112	49	0.784319	1
36	chr6	40360058	40360058	A	G	exonic	LRFN2		nonsynonymous SNV	LRFN2:NM_020737:exon3:c.T1994C:p.L66	NOVEL		106	2	0.0467329	1	76	33	0.780208	1
36	chr2	113943798	113943798	G	T	exonic	PSD4		nonsynonymous SNV	PSD4:NM_012455:exon5:c.G1594T:p.V532	NOVEL		45	0	0	1	49	21	0.773115	1
36	chr19	18272787	18272787	G	A	exonic	PIK3R2		nonsynonymous SNV	PIK3R2:NM_005027:exon7:c.G827A:p.S27	DBSNP		86	1	0.0405009	1	106	29	0.768403	1
36	chr6	90406203	90406203	C	T	exonic	MDN1		nonsynonymous SNV	MDN1:NM_014611:exon60:c.G9259A:p.G	NOVEL		59	1	0.0420596	1	89	36	0.74219	1
36	chr16	1827856	1827856	C	T	exonic	SPSB3		nonsynonymous SNV	SPSB3:NM_001324081:exon6:c.G613A:p.G	NOVEL		65	0	0	1	68	27	0.732424	1
36	chr1	180235642	180235642	C	T	exonic	LHX4		nonsynonymous SNV	LHX4:NM_033343:exon3:c.C364T:p.R122W	DBSNP		266	10	0.127666	1	271	69	0.725931	1
36	chr16	4432750	4432750	G	A	exonic	VASN		synonymous SNV	VASN:NM_138440:exon2:c.G1872A:p.L62	NOVEL		73	0	0	1	57	22	0.717659	1
36	chr3	10302037	10302037	A	C	exonic	TATDN2		nonsynonymous SNV	TATDN2:NM_014760:exon3:c.A631C:p.K21	NOVEL		244	4	0.0407029	1	218	84	0.716795	1
36	chr7	128517304	128517304	G	C	exonic	KCP		unknown	UNKNOWN	NOVEL		57	4	0.165481	1	40	15	0.702832	1
36	chr16	677559	677559	G	A	exonic	RAB40C		synonymous SNV	RAB40C:NM_021168:exon6:c.G783A:p.K2	NOVEL		320	9	0.069034	1	239	88	0.693518	1
36	chr5	41195983	41195983	G	T	exonic	C6		nonsynonymous SNV	C6:NM_000065:exon5:c.C498A:p.D166E,C6	DBSNP		444	11	0.0851854	1	542	130	0.691989	1
36	chr9	139995586	139995586	G	A	exonic	MAN1B1		nonsynonymous SNV	MAN1B1:NM_016219:exon7:c.G1046A:p.S	NOVEL		43	0	0	1	62	11	0.689692	1
36	chr7	1131397	1131397	C	A	exonic	GPER1		synonymous SNV	GPER1:NM_001098201:exon2:c.C33A:p.G1	NOVEL		297	6	0.0499718	1	223	81	0.686648	1
36	chr1	156214507	156214507	C	T	exonic	PAQR6		nonsynonymous SNV	PAQR6:NM_001272106:exon5:c.G283A:p.	NOVEL		194	0	0	1	232	53	0.665206	1
36	chr3	13611909	13611909	G	C	exonic	FBLN2		synonymous SNV	FBLN2:NM_001004019:exon2:c.G54C:p.L1	NOVEL		52	0	0	1	69	24	0.665045	1
36	chr17	27448152	27448152	C	A	exonic	MYO18A		nonsynonymous SNV	MYO18A:NM_001346767:exon6:c.G1449T	NOVEL		218	0	0	1	156	54	0.66267	1
36	chr1	34070914	34070914	C	T	exonic	CSMD2		nonsynonymous SNV	CSMD2:NM_001281956:exon42:c.G6500A	DBSNP		76	2	0.0647071	1	84	29	0.661367	1
36	chr15	74572373	74572373	G	A	exonic	CCDC33		nonsynonymous SNV	CCDC33:NM_025055:exon8:c.G829A:p.G2	DBSNP		233	5	0.0740247	1	230	52	0.659598	1
36	chr1	151137658	151137658	C	T	exonic	LYSMD1		nonsynonymous SNV	LYSMD1:NM_212551:exon1:c.G77A:p.S26	NOVEL		240	1	0.0146207	1	254	57	0.655601	1
36	chr19	41105147	41105147	G	A	exonic	LTBP4		unknown	UNKNOWN	NOVEL		109	0	0	1	103	23	0.652953	1
36	chr20	62374282	62374282	G	A	exonic	SLC2A4RG		nonsynonymous SNV	SLC2A4RG:NM_020062:exon8:c.G1103A:p	DBSNP+COSMIC	ID=COSM293277	242	1	0.0103851	1	218	73	0.646476	1
36	chr1	154841808	154841808	G	C	exonic	KCNN3		nonsynonymous SNV	KCNN3:NM_001204087:exon1:c.C633G:p.F	NOVEL		81	1	0.0429705	1	83	18	0.637494	1
36	chr14	35245654	35245654	C	A	exonic	BAZ1A		nonsynonymous SNV	BAZ1A:NM_182648:exon17:c.G2208T:p.E7	NOVEL		135	1	0.0185557	1	185	60	0.631114	1
36	chr8	142204138	142204138	G	A	exonic	DENND3		nonsynonymous SNV	DENND3:NM_001352890:exon23:c.G3643	NOVEL		58	0	0	1	79	25	0.619483	1
36	chr2	33622242	33622242	G	A	exonic	LTBP1		nonsynonymous SNV	LTBP1:NM_001166264:exon28:c.G3773A:	NOVEL		48	0	0	1	64	20	0.613583	1
36	chr15	55664182	55664182	C	T	exonic	CCPG1		nonsynonymous SNV	CCPG1:NM_001204450:exon6:c.G515A:p.S	NOVEL		164	2	0.0424527	1	229	47	0.609135	1
36	chr2	109381778	109381778	C	T	exonic	RANBP2		nonsynonymous SNV	RANBP2:NM_006267:exon20:c.C4783T:p.R	NOVEL		168	0	0	1	279	86	0.607195	1
36	chr20	51870822	51870822	G	A	exonic	TSHZ2		synonymous SNV	TSHZ2:NM_001193421:exon2:c.G816A:p.L	NOVEL		186	1	0.0134951	1	238	72	0.59854	1
36	chr11	65629747	65629747	C	T	exonic	MUS81		synonymous SNV	MUS81:NM_001350283:exon5:c.C516T:p.F	DBSNP		45	0	0	1	53	16	0.597577	1
36	chr19	55751469	55751469	G	A	exonic	PPP6R1		nonsynonymous SNV	PPP6R1:NM_014931:exon12:c.C1375T:p.P	NOVEL		219	4	0.0616463	1	169	51	0.597407	1
36	chr3	119133480	119133480	C	A	exonic	ARHGAP31		nonsynonymous SNV	ARHGAP31:NM_020754:exon12:c.C2704A:	NOVEL		250	0	0	1	235	70	0.591454	1
36	chr11	33370198	33370198	A	C	exonic	HIPK3		synonymous SNV	HIPK3:NM_001048200:exon12:c.A2437C:p	NOVEL		224	7	0.0764721	1	262	78	0.591205	1
36	chr10	49998921	49998921	G	A	exonic	WDFY4		nonsynonymous SNV	WDFY4:NM_001370153:exon23:c.G4216A	NOVEL		485	9	0.0459761	1	523	152	0.580313	1
36	chr3	4725314	4725314	T	C	exonic	ITPR1		synonymous SNV	ITPR1:NM_001168272:exon27:c.T3285C:p.	NOVEL		321	8	0.0613636	1	367	105	0.573284	1
36	chr9	37735721	37735721	A	C	exonic	FRMPD1		nonsynonymous SNV	FRMPD1:NM_001371223:exon13:c.A1391	NOVEL		130	1	0.0345311	1	190	32	0.569279	1
36	chr6	34824162	34824162	G	C	exonic	UHRF1BP1		nonsynonymous SNV	UHRF1BP1:NM_017754:exon10:c.G1267C:	NOVEL		57	0	0	1	71	20	0.566384	1
36	chr20	31383308	31383308	G	A	exonic	DNMT3B		nonsynonymous SNV	DNMT3B:NM_001207056:exon8:c.G932A:	NOVEL		152	2	0.0327737	1	167	47	0.565987	1
36	chr5	162890894	162890894	A	C	exonic	HMMR		nonsynonymous SNV	HMMR:NM_001142556:exon2:c.A79C:p.K2	NOVEL		38	0	0	1	48	9	0.564797	1
36	chr2	44174447	44174447	T	G	exonic	LRPPRC		nonsynonymous SNV	LRPPRC:NM_133259:exon20:c.A2028C:p.Q	NOVEL		84	1	0.0296892	1	107	30	0.564317	1
36	chr1	61869946	61869946	T	G	exonic	NFIA		nonsynonymous SNV	NFIA:NM_001134673:exon8:c.T1246G:p.F	NOVEL		87	1	0.028677	1	122	34	0.561665	1
36	chr11	5248214	5248214	G	T	exonic	HBB		nonsynonymous SNV	HBB:NM_000518:exon1:c.C38A:p.T13N	NOVEL		141	0	0	1	191	53	0.559769	1
36	chr8	145726662	145726662	G	A	exonic	PPP1R16A		synonymous SNV	PPP1R16A:NM_001329443:exon11:c.G118	NOVEL		90	0	0	1	170	47	0.558163	1
36	chr7	14758228	14758228	C	T	exonic	DGKB		synonymous SNV	DGKB:NM_001350711:exon5:c.G384A:p.L1	COSMIC	ID=COSM715786	167	1	0.0150213	1	160	44	0.555834	1
36	chr15	49574219	49574219	T	C	exonic	GALK2		synonymous SNV	GALK2:NM_001001556:exon6:c.T507C:p.R	NOVEL		158	0	0	1	165	30	0.550315	1
36	chr9	116224425	116224425	G	C	exonic	RGS3		nonsynonymous SNV	RGS3:NM_144488:exon4:c.G359C:p.W120	NOVEL		182	1	0.024719	1	198	27	0.549246	1
36	chr18	76755215	76755215	G	C	exonic	SALL3		nonsynonymous SNV	SALL3:NM_171999:exon2:c.G3224C:p.G10	NOVEL		116	0	0	1	163	44	0.547779	1
36	chr1	202777268	202777268	C	T	exonic	KDM5B		nonsynonymous SNV	KDM5B:NM_001314042:exon1:c.G166A:p.	NOVEL		220	2	0.0317439	1	194	35	0.546711	1
36	chr9	35957529	35957529	G	T	exonic	OR2S2		synonymous SNV	OR2S2:NM_019897:exon1:c.C567A:p.A189	NOVEL		188	0	0	1	270	43	0.542565	1
36	chr15	100269848	100269848	C	T	exonic	LYSMD4		nonsynonymous SNV	LYSMD4:NM_001284420:exon2:c.G188A:p	NOVEL		162	1	0.021617	1	224	40	0.541977	1
36	chr4	7714561	7714561	G	C	exonic	SORCS2		nonsynonymous SNV	SORCS2:NM_020777:exon15:c.G1970C:p.V	NOVEL		94	2	0.0525745	1	79	21	0.54118	1
36	chr5	132150469	132150469	T	C	exonic	SOWAHA		nonsynonymous SNV	SOWAHA:NM_175873:exon1:c.T1156C:p.Y	NOVEL		112	0	0	1	96	17	0.53814	1
36	chr19	11328042	11328042	C	G	exonic	DOCK6		synonymous SNV	DOCK6:NM_020812:exon29:c.G3576C:p.L1	NOVEL		261	5	0.0662327	1	278	49	0.53601	1
36	chr14	97000834	97000834	G	A	exonic	PAPOLA		stopgain	PAPOLA:NM_001293632:exon8:c.G93A:p.V	DBSNP		247	6	0.0598477	1	367	96	0.534334	1
36	chr2	186672560	186672560	A	T	exonic	FSIP2		nonsynonymous SNV	FSIP2:NM_173651:exon17:c.A18527T:p.Y6	NOVEL		102	3	0.0721022	1	122	31	0.522147	1
36	chr4	99956568	99956568	G	A	exonic	METAP1		synonymous SNV	METAP1:NM_015143:exon4:c.G297A:p.V9	NOVEL		72	0	0	1	107	27	0.519256	1
36	chr19	42213775	42213775	G	C	exonic	CEACAM5		nonsynonymous SNV	CEACAM5:NM_001291484:exon2:c.G241C	NOVEL		515	2	0.0135985	1	537	90	0.513452	1
36	chr1	192779500	192779500	G	A	exonic	RGS2		synonymous SNV	RGS2:NM_002923:exon3:c.G213A:p.K71K	NOVEL		152	0	0	1	198	33	0.511007	1
36	chr12	57619030	57619030	A	C	exonic	NXPH4		nonsynonymous SNV	NXPH4:NM_007224:exon2:c.A427C:p.N14	NOVEL		477	4	0.0209861	1	437	108	0.510681	1
36	chr2	211158475	211158475	T	C	exonic	MYL1		synonymous SNV	MYL1:NM_079420:exon5:c.A528G:p.E176	NOVEL		271	4	0.0367066	1	332	82	0.51043	1
36	chr2	196722393	196722393	T	A	exonic	DNAH7		nonsynonymous SNV	DNAH7:NM_018897:exon44:c.A8122T:p.M	NOVEL		171	1	0.014672	1	175	43	0.508317	1
36	chr5	149784725	149784725	C	A	exonic	CD74		nonsynonymous SNV	CD74:NM_001364083:exon4:c.G397T:p.V1	NOVEL		242	2	0.0288818	1	249	41	0.505721	1
36	chr14	24880385	24880385	C	T	exonic	NYNRIN		stopgain	NYNRIN:NM_025081:exon5:c.C2518T:p.Q8	NOVEL		319	1	0.00788618	1	221	53	0.49848	1
36	chr2	97279332	97279332	G	T	exonic	KANSL3		nonsynonymous SNV	KANSL3:NM_001349262:exon4:c.C4A:p.L2	NOVEL		99	0	0	1	134	32	0.496781	1
36	chr1	120166142	120166142	T	G	exonic	ZNF697		nonsynonymous SNV	ZNF697:NM_001080470:exon3:c.A824C:p.	NOVEL		135	2	0.0368406	1	129	30	0.486236	1
36	chr15	65687451	65687451	C	G	exonic	IGDCC4		synonymous SNV	IGDCC4:NM_020962:exon8:c.G1557C:p.L5	NOVEL		27	0	0	1	70	11	0.485772	1
36	chr15	42162071	42162071	G	A	exonic	SPTBN5		nonsynonymous SNV	SPTBN5:NM_016642:exon32:c.C5821T:p.R	DBSNP		20	7	0.91352	1	32	5	0.483385	1
36	chr9	101904952	101904952	G	C	exonic	TGFBR1		nonsynonymous SNV	TGFBR1:NM_001130916:exon4:c.G709C:p	NOVEL		395	0	0	1	469	55	0.480415	1
36	chr12	46318843	46318843	C	G	exonic	SCAF11		nonsynonymous SNV	SCAF11:NM_004719:exon12:c.G3574C:p.D	NOVEL		81	0	0	1	159	36	0.475763	1
36	chr17	10300128	10300128	A	T	exonic	MYH8		nonsynonymous SNV	MYH8:NM_002472:exon31:c.T4354A:p.F14	NOVEL		166	0	0	1	227	51	0.472768	1
36	chr2	43968179	43968179	C	A	exonic	PLEKHH2		nonsynonymous SNV	PLEKHH2:NM_172069:exon21:c.C3218A:p.	NOVEL		254	2	0.0197155	1	272	60	0.465732	1
36	chr19	42187833	42187833	C	A	exonic	CEACAM7		nonsynonymous SNV	CEACAM7:NM_001291485:exon3:c.G589T	NOVEL		311	1	0.0112667	1	337	50	0.462151	1
36	chr11	62370674	62370674	C	A	exonic	EML3		nonsynonymous SNV	EML3:NM_001300793:exon20:c.G2306T:p	NOVEL		245	1	0.0102584	1	235	50	0.452114	1
36	chr9	117555815	117555815	G	T	exonic	TNFSF15		nonsynonymous SNV	TNFSF15:NM_005118:exon2:c.C244A:p.Q8	NOVEL		499	0	0	1	551	60	0.449465	1
36	chr15	77473660	77473660	T	G	exonic	PEAK1		nonsynonymous SNV	PEAK1:NM_024776:exon5:c.A609C:p.E203	NOVEL		474	0	0	1	550	78	0.444283	1
36	chr12	46318849	46318849	G	A	exonic	SCAF11		synonymous SNV	SCAF11:NM_004719:exon12:c.C3568T:p.L	DBSNP		71	0	0	1	150	31	0.441373	1
36	chr14	63174756	63174756	G	A	exonic	KCNH5		stopgain	KCNH5:NM_139318:exon11:c.C2437T:p.R8	NOVEL		122	77	0.97646	1	189	51	0.426451	1
36	chr11	103027398	103027398	A	C	exonic	DYNC2H1		nonsynonymous SNV	DYNC2H1:NM_001080463:exon26:c.A4026	NOVEL		77	0	0	1	155	30	0.4179	1
36	chr9	35699985	35699985	C	G	exonic	TLN1		nonsynonymous SNV	TLN1:NM_006289:exon50:c.G6754C:p.D22	NOVEL		133	0	0	1	173	20	0.409261	1
36	chr13	21373411	21373411	G	C	exonic	XPO4		nonsynonymous SNV	XPO4:NM_022459:exon16:c.C2215G:p.P73	NOVEL		204	1	0.0123101	1	249	47	0.409194	1
36	chr19	58879267	58879267	C	T	exonic	ZNF837		nonsynonymous SNV	ZNF837:NM_138466:exon3:c.G1433A:p.R4	NOVEL		83	0	0	1	84	15	0.390462	1
36	chr3	15115844	15115844	C	T	exonic	RBSN		stopgain	RBSN:NM_001302378:exon13:c.G1800A:p	NOVEL		127	3	0.0582364	1	107	19	0.388603	1
36	chr16	23766423	23766423	G	T	exonic	CHP2		nonsynonymous SNV	CHP2:NM_022097:exon1:c.G53T:p.R18L	NOVEL		51	0	0	1	36	6	0.36815	1
36	chr15	41105571	41105571	G	C	exonic	ZFYVE19		nonsynonymous SNV	ZFYVE19:NM_001258420:exon7:c.G862C:	NOVEL		197	0	0	1	168	18	0.346166	1
36	chr19	14269917	14269917	G	T	exonic	ADGRL1		nonsynonymous SNV	ADGRL1:NM_014921:exon11:c.C2134A:p.C	NOVEL		90	5	0.185451	1	120	12	0.325186	1
36	chr1	186413590	186413590	T	A	exonic	PDC		nonsynonymous SNV	PDC:NM_022576:exon3:c.A106T:p.N36Y,P	NOVEL		292	46	0.47954	1	495	49	0.322197	1
36	chr17	39597145	39597145	C	T	exonic	KRT38		nonsynonymous SNV	KRT38:NM_006771:exon1:c.G29A:p.C10Y	NOVEL		19	3	0.344124	1	29	4	0.31237	1
36	chr7	150655537	150655537	G	A	exonic	KCNH2		nonsynonymous SNV	KCNH2:NM_000238:exon4:c.C526T:p.R176	DBSNP		27	4	0.325623	1	37	5	0.306792	1
36	chr3	113376214	113376214	G	A	exonic	USF3		stopgain	USF3:NM_001009899:exon7:c.C4315T:p.Q	NOVEL		60	3	0.12017	1	67	9	0.305177	1
36	chr15	81271719	81271719	A	T	exonic	MESD		nonsynonymous SNV	MESD:NM_015154:exon3:c.T546A:p.D182	NOVEL		304	0	0	1	335	31	0.302974	1
36	chr9	35611824	35611824	C	A	exonic	CD72		nonsynonymous SNV	CD72:NM_001782:exon7:c.G927T:p.L309F	NOVEL		223	13	0.24918	1	232	19	0.298956	1
36	chr12	113515280	113515280	G	C	exonic	DTX1		nonsynonymous SNV	DTX1:NM_004416:exon2:c.G311C:p.G104	NOVEL		107	2	0.0463042	1	119	15	0.288476	1
36	chr16	74946252	74946252	C	A	exonic	WDR59		synonymous SNV	WDR59:NM_001324171:exon14:c.G1233T	NOVEL		10	0	0	1	24	3	0.286339	1
36	chr15	72104825	72104825	T	A	exonic	NR2E3		nonsynonymous SNV	NR2E3:NM_014249:exon5:c.T721A:p.F241	NOVEL		97	2	0.0711834	1	164	14	0.281341	1
36	chr19	54783502	54783502	C	T	exonic	LILRB2		nonsynonymous SNV	LILRB2:NM_001278404:exon4:c.G8A:p.G3	COSMIC	ID=COSM353868	141	4	0.0948078	1	172	21	0.280404	1
36	chr6	56392416	56392416	T	A	exonic	DST		nonsynonymous SNV	DST:NM_015548:exon49:c.A9928T:p.R331	NOVEL		84	7	0.194121	1	158	19	0.276632	1
36	chr1	168066296	168066296	G	A	exonic	GPR161		synonymous SNV	GPR161:NM_001267613:exon2:c.C315T:p.	NOVEL		379	10	0.0905804	1	425	35	0.272167	1
36	chr1	228698264	228698264	G	A	exonic	BTNL10		synonymous SNV	BTNL10:NM_001287262:exon3:c.C423T:p.	NOVEL		161	7	0.146816	1	198	16	0.267443	1
36	chr1	155288022	155288022	G	C	exonic	FDPS		synonymous SNV	FDPS:NM_001242825:exon4:c.G111C:p.L3	NOVEL		110	4	0.123634	1	169	13	0.255504	1
36	chr15	74883921	74883921	A	C	exonic	ARID3B		nonsynonymous SNV	ARID3B:NM_001307939:exon7:c.A1186C:p	NOVEL		38	1	0.0903481	1	66	5	0.251905	1
36	chr12	72148954	72148954	C	G	exonic	RAB21		synonymous SNV	RAB21:NM_014999:exon1:c.C45G:p.G15G	DBSNP		46	2	0.105149	1	111	12	0.251419	1
36	chr15	82450061	82450061	T	G	exonic	EFL1		nonsynonymous SNV	EFL1:NM_001040610:exon15:c.A1870C:p.K	NOVEL		224	1	0.0156603	1	271	20	0.245845	1
36	chr17	37054713	37054713	G	A	exonic	LASP1		nonsynonymous SNV	LASP1:NM_001271608:exon3:c.G130A:p.V	NOVEL		178	7	0.0954867	1	228	24	0.245433	1
36	chr21	47556854	47556854	G	A	exonic	FTCD		synonymous SNV	FTCD:NM_001320412:exon14:c.C1653T:p.	DBSNP		144	14	0.312216	1	156	11	0.235614	1
36	chr7	45216955	45216955	A	G	exonic	RAMP3		nonsynonymous SNV	RAMP3:NM_005856:exon2:c.A106G:p.R36	NOVEL		164	9	0.131284	1	209	21	0.235296	1
36	chr14	92126209	92126209	T	G	exonic	CATSPERB		nonsynonymous SNV	CATSPERB:NM_024764:exon15:c.A1404C:	NOVEL		19	0	0	1	54	5	0.218394	1
36	chr11	6806794	6806794	C	T	exonic	OR2AG1		nonsynonymous SNV	OR2AG1:NM_001004489:exon1:c.C526T:p	NOVEL		237	15	0.150213	1	374	34	0.214754	1
36	chr5	2749143	2749143	G	C	exonic	IRX2		nonsynonymous SNV	IRX2:NM_001134222:exon3:c.C679G:p.L22	NOVEL		268	11	0.138922	1	316	20	0.21292	1
36	chr8	27277518	27277518	G	C	exonic	PTK2B		nonsynonymous SNV	PTK2B:NM_173175:exon3:c.G311C:p.W10	NOVEL		143	16	0.253945	1	156	14	0.212228	1
36	chr5	68677731	68677731	G	C	exonic	RAD17		nonsynonymous SNV	RAD17:NM_133339:exon4:c.G388C:p.G13	NOVEL		93	5	0.179774	1	207	13	0.211371	1
36	chr15	74328123	74328123	C	T	exonic	PML		nonsynonymous SNV	PML:NM_033250:exon7:c.C2177T:p.A726V	NOVEL		292	4	0.0476159	1	433	27	0.209957	1
36	chr17	43366662	43366662	C	G	exonic	MAP3K14		unknown	UNKNOWN	NOVEL		147	11	0.175692	1	183	16	0.2072	1
36	chr15	31793982	31793982	C	T	exonic	OTUD7A		nonsynonymous SNV	OTUD7A:NM_001329907:exon11:c.G1082	NOVEL		189	6	0.108418	1	235	14	0.201119	1
36	chr21	34927603	34927603	A	G	exonic	SON		synonymous SNV	SON:NM_001291411:exon3:c.A6066G:p.T	NOVEL		227	3	0.0459597	1	353	21	0.20085	1
36	chr15	42967108	42967108	G	A	exonic	STARD9		nonsynonymous SNV	STARD9:NM_020759:exon18:c.G1633A:p.A	NOVEL		89	6	0.222542	1	136	8	0.198725	1
36	chr12	117693830	117693830	G	A	exonic	NOS1		synonymous SNV	NOS1:NM_001204218:exon17:c.C2544T:p.	NOVEL		77	2	0.063888	1	72	6	0.198235	1
36	chr2	26703783	26703783	G	C	exonic	OTOF		synonymous SNV	OTOF:NM_001287489:exon15:c.C1674G:p	DBSNP		327	9	0.0675958	1	286	23	0.191819	1
36	chr11	64662538	64662538	C	G	exonic	ATG2A		synonymous SNV	ATG2A:NM_001367971:exon41:c.G5700C:	NOVEL		131	7	0.128008	1	112	9	0.191681	1
36	chr16	1818375	1818375	G	C	exonic	MAPK8IP3		synonymous SNV	MAPK8IP3:NM_001040439:exon29:c.G371	NOVEL		178	3	0.0418273	1	230	18	0.187044	1
36	chr4	46307615	46307615	T	C	exonic	GABRA2		nonsynonymous SNV	GABRA2:NM_001114175:exon6:c.A673G:p	DBSNP		357	9	0.0620552	1	462	36	0.186293	1
36	chr5	114860816	114860816	T	A	exonic	FEM1C		nonsynonymous SNV	FEM1C:NM_020177:exon3:c.A1043T:p.Y34	NOVEL		287	12	0.141414	1	421	23	0.185298	1
36	chr19	45911565	45911565	C	T	exonic	CD3EAP		synonymous SNV	CD3EAP:NM_001297590:exon3:c.C345T:p.	DBSNP		174	13	0.244374	1	239	13	0.18453	1
36	chr5	147505142	147505142	G	T	exonic	SPINK5		nonsynonymous SNV	SPINK5:NM_001127698:exon29:c.G2795T:	NOVEL		190	4	0.0726511	1	277	15	0.183753	1
36	chr14	20897110	20897110	C	G	exonic	KLHL33		synonymous SNV	KLHL33:NM_001109997:exon4:c.G1500C:p	NOVEL		107	2	0.0463042	1	157	12	0.182986	1
36	chr7	142566820	142566820	C	A	exonic	EPHB6		nonsynonymous SNV	EPHB6:NM_001280795:exon12:c.C1501A:	NOVEL		205	2	0.0243824	1	211	16	0.181642	1
36	chr14	92264136	92264136	C	A	exonic	TC2N		nonsynonymous SNV	TC2N:NM_001128595:exon8:c.G848T:p.G2	DBSNP		101	11	0.247851	1	160	12	0.179794	1
36	chr16	81819597	81819597	G	T	exonic	PLCG2		startloss	PLCG2:NM_002661:exon2:c.G3T:p.M1?	COSMIC	ID=COSM371210	177	13	0.172666	1	254	19	0.179355	1
36	chr16	58538104	58538104	G	A	exonic	NDRG4		synonymous SNV	NDRG4:NM_001242834:exon3:c.G228A:p.	NOVEL		162	0	0	1	161	12	0.178755	1
36	chr6	18197864	18197864	C	T	exonic	KDM1B		nonsynonymous SNV	KDM1B:NM_153042:exon10:c.C797T:p.A2	NOVEL		153	14	0.211557	1	220	16	0.174715	1
36	chr5	72868316	72868316	G	A	exonic	UTP15		nonsynonymous SNV	UTP15:NM_001284431:exon6:c.G106A:p.G	NOVEL		157	2	0.0443217	1	234	12	0.17449	1
36	chr6	52858999	52858999	G	A	exonic	GSTA4		nonsynonymous SNV	GSTA4:NM_001512:exon2:c.C43T:p.R15W	DBSNP		84	0	0	1	124	9	0.174387	1
36	chr17	36483342	36483342	C	G	exonic	GPR179		nonsynonymous SNV	GPR179:NM_001004334:exon11:c.G6110	NOVEL		223	6	0.0661199	1	326	23	0.169834	1
36	chr1	207643119	207643119	C	A	exonic	CR2		nonsynonymous SNV	CR2:NM_001006658:exon6:c.C897A:p.S29	NOVEL		346	16	0.155738	1	589	29	0.167855	1
36	chr3	141162956	141162956	G	A	exonic	ZBTB38		nonsynonymous SNV	ZBTB38:NM_001350100:exon4:c.G1726A:	NOVEL		339	11	0.0793124	1	558	38	0.164309	1
36	chr17	5392630	5392630	G	A	exonic	MIS12		nonsynonymous SNV	MIS12:NM_001258218:exon2:c.G448A:p.A	NOVEL		276	20	0.170512	1	416	28	0.162517	1
36	chr7	143573568	143573568	C	T	exonic	TCAF1		nonsynonymous SNV	TCAF1:NM_001206938:exon2:c.G134A:p.G	NOVEL		128	5	0.0948713	1	166	11	0.160156	1

36	chr15	43817381	43817381	T	C	exonic	MAP1A		nonsynonymous SNV	MAP1A:NM_002373:exon4:c.T3710C:p.M1	NOVEL		285	7	0.0844693	1	365	17	0.159188	1
36	chr1	11561604	11561604	T	G	exonic	DISP3		synonymous SNV	DISP3:NM_020780:exon2:c.T555G:p.P185P	NOVEL		145	7	0.116217	1	200	13	0.157285	1
36	chr14	93649954	93649954	C	A	exonic	MOAP1		nonsynonymous SNV	MOAP1:NM_022151:exon3:c.G634T:p.G21	NOVEL		156	8	0.123101	1	285	18	0.153092	1
36	chr9	119567936	119567936	C	A	exonic	ASTN2		nonsynonymous SNV	ASTN2:NM_014010:exon12:c.G2218T:p.G7	NOVEL		249	9	0.157799	1	318	11	0.153032	1
36	chr17	59477862	59477862	C	T	exonic	TBX2		synonymous SNV	TBX2:NM_005994:exon1:c.C325T:p.L109L	NOVEL		196	14	0.168239	1	175	11	0.152406	1
36	chr20	47836047	47836047	C	T	exonic	DDX27		nonsynonymous SNV	DDX27:NM_001348187:exon1:c.C155T:p.P	NOVEL		183	3	0.0407029	1	224	14	0.151591	1
36	chr2	37471071	37471071	G	A	exonic	NDUFAF7		synonymous SNV	NDUFAF7:NM_001083946:exon5:c.G453A	DBSNP		173	1	0.0145033	1	241	15	0.150999	1
36	chr12	55026982	55026982	C	G	exonic	LACRT		nonsynonymous SNV	LACRT:NM_033277:exon2:c.G94C:p.A32P	NOVEL		157	14	0.206609	1	194	12	0.150119	1
36	chr4	125591859	125591859	C	T	exonic	ANKRD50		nonsynonymous SNV	ANKRD50:NM_001167882:exon3:c.G2036	NOVEL		347	14	0.0978673	1	607	37	0.14806	1
36	chr5	131308989	131308989	G	C	exonic	ACSL6		synonymous SNV	ACSL6:NM_001205251:exon11:c.C873G:p.	NOVEL		145	0	0	1	186	8	0.147507	1
36	chr11	10823669	10823669	C	T	exonic	EIF4G2		nonsynonymous SNV	EIF4G2:NM_001042559:exon13:c.G1226A	NOVEL		198	16	0.188679	1	282	17	0.146521	1
36	chr14	23898441	23898441	C	G	exonic	MYH7		nonsynonymous SNV	MYH7:NM_000257:exon13:c.G1254C:p.Q4	NOVEL		373	10	0.0658898	1	387	23	0.144566	1
36	chr8	68189665	68189665	G	C	exonic	ARFGEF1		nonsynonymous SNV	ARFGEF1:NM_006421:exon8:c.C1055G:p.	NOVEL		100	8	0.186932	1	170	10	0.143169	1
36	chr7	71488749	71488749	C	G	exonic	CALN1		nonsynonymous SNV	CALN1:NM_001017440:exon4:c.G268C:p.G	NOVEL		281	3	0.0266575	1	411	24	0.142182	1
36	chr9	139412286	139412286	G	A	exonic	NOTCH1		synonymous SNV	NOTCH1:NM_017617:exon8:c.C1359T:p.V4	NOVEL		358	1	0.0126005	1	385	12	0.138349	1
36	chr17	6673972	6673972	G	T	exonic	XAF1		nonsynonymous SNV	XAF1:NM_001353140:exon4:c.G308T:p.C1	NOVEL		141	2	0.0352948	1	198	11	0.135634	1
36	chr10	69644805	69644805	G	T	exonic	SIRT1		nonsynonymous SNV	SIRT1:NM_012238:exon1:c.G326T:p.G109	NOVEL		117	1	0.0213863	1	135	7	0.127038	1
36	chr17	7402423	7402423	G	A	exonic	POLR2A		nonsynonymous SNV	POLR2A:NM_000937:exon9:c.G1401A:p.M	NOVEL		183	3	0.0407029	1	234	12	0.12571	1
36	chr11	14991573	14991573	G	C	exonic	CALCA		nonsynonymous SNV	CALCA:NM_001033952:exon3:c.C135G:p.D	DBSNP		93	0	0	1	117	6	0.12571	1
36	chr1	86523614	86523614	T	C	exonic	COL24A1		synonymous SNV	COL24A1:NM_152890:exon10:c.A1851G:p	NOVEL		223	3	0.0334988	1	304	15	0.121178	1
36	chr19	14512249	14512249	G	A	exonic	ADGRE5		nonsynonymous SNV	ADGRE5:NM_001784:exon8:c.G670A:p.A2	NOVEL		163	5	0.104868	1	268	9	0.116222	1
36	chr4	24534563	24534563	C	A	exonic	DHX15		nonsynonymous SNV	DHX15:NM_001358:exon12:c.G2024T:p.R6	NOVEL		132	8	0.144204	1	277	13	0.115523	1
36	chr12	40441946	40441946	A	T	exonic	SLC2A13		stopgain	SLC2A13:NM_052885:exon2:c.T623A:p.L20	NOVEL		227	29	0.285874	1	377	17	0.111192	1
36	chr17	18218027	18218027	G	A	exonic	TOP3A		synonymous SNV	TOP3A:NM_004618:exon1:c.C66T:p.S22S	NOVEL		140	7	0.12017	1	191	8	0.1036	1
36	chr2	73315586	73315586	C	G	exonic	RAB11FIP5		nonsynonymous SNV	RAB11FIP5:NM_001371272:exon3:c.G116	NOVEL		208	5	0.0592389	1	243	10	0.10186	1
36	chr19	6534844	6534844	G	A	exonic	TNFSF9		nonsynonymous SNV	TNFSF9:NM_003811:exon3:c.G532A:p.A17	DBSNP		350	24	0.226112	1	379	10	0.091955	1
36	chr19	8468385	8468385	G	A	exonic	RAB11B		synonymous SNV	RAB11B:NM_004218:exon5:c.G600A:p.V2	NOVEL		339	14	0.139745	1	501	13	0.0904701	1
36	chr15	75651510	75651510	C	T	exonic	MAN2C1		synonymous SNV	MAN2C1:NM_001256494:exon18:c.G2082	NOVEL		378	15	0.134488	1	480	12	0.0872451	1
36	chr7	7278255	7278255	G	A	exonic	C1GALT1		nonsynonymous SNV	C1GALT1:NM_020156:exon3:c.G590A:p.R1	NOVEL		222	7	0.0771399	1	324	11	0.0846195	1
36	chr15	54305888	54305888	T	G	exonic	UNC13C		nonsynonymous SNV	UNC13C:NM_001080534:exon1:c.T788G:p	NOVEL		374	14	0.127139	1	539	13	0.0842421	1
36	chr1	10207100	10207100	G	A	exonic	UBE4B		nonsynonymous SNV	UBE4B:NM_006048:exon18:c.G2156A:p.G	NOVEL		101	6	0.141509	1	148	5	0.0842173	1
36	chr6	41877090	41877090	C	G	exonic	MED20		nonsynonymous SNV	MED20:NM_001305455:exon2:c.G154C:p.	NOVEL		226	7	0.0758156	1	268	9	0.0837308	1
36	chr1	116577833	116577833	G	T	exonic	SLC22A15		nonsynonymous SNV	SLC22A15:NM_018420:exon7:c.G970T:p.G	NOVEL		220	3	0.0339495	1	311	10	0.0802819	1
36	chr1	39340335	39340335	A	T	exonic	GJA9		nonsynonymous SNV	GJA9:NM_030772:exon2:c.T1436A:p.F479	NOVEL		177	10	0.134951	1	264	8	0.0757956	1
36	chr8	13356841	13356841	C	T	exonic	DLC1		nonsynonymous SNV	DLC1:NM_001348081:exon2:c.G740A:p.R2	DBSNP		207	10	0.116294	1	379	10	0.0662481	1
36	chr16	78064629	78064629	G	C	exonic	CLEC3A		nonsynonymous SNV	CLEC3A:NM_005752:exon3:c.G485C:p.G16	NOVEL		174	11	0.150051	1	234	6	0.0644262	1
36	chr19	45206719	45206719	G	C	exonic	CEACAM16		synonymous SNV	CEACAM16:NM_001039213:exon3:c.G138	NOVEL		147	8	0.181431	1	172	3	0.0613208	1
36	chr2	120125056	120125056	G	C	exonic	DBI		synonymous SNV	DBI:NM_001178017:exon1:c.G144C:p.G48	NOVEL		65	8	0.276556	1	134	3	0.0564317	1
36	chr11	103075617	103075617	G	C	exonic	DYNC2H1		nonsynonymous SNV	DYNC2H1:NM_001080463:exon52:c.G8378	NOVEL		97	7	0.169856	1	137	3	0.0552225	1
36	chr13	28623866	28623866	T	G	exonic	FLT3		nonsynonymous SNV	FLT3:NM_004119:exon7:c.A788C:p.K263T	NOVEL		130	9	0.163397	1	191	4	0.0528625	1
36	chr20	8770882	8770882	C	T	exonic	PLCB1		stopgain	PLCB1:NM_015192:exon31:c.C3397T:p.Q1	NOVEL		242	14	0.138008	1	391	8	0.0516702	1
36	chr16	27473777	27473777	C	T	exonic	GTF3C1		synonymous SNV	GTF3C1:NM_001286242:exon36:c.G5880A	DBSNP		128	9	0.165782	1	174	3	0.0436788	1
36	chr8	55540763	55540763	G	T	exonic	RP1		stopgain	RP1:NM_006269:exon4:c.G4321T:p.E1441	NOVEL		100	15	0.329162	1	175	3	0.0434334	1
36	chr15	41687143	41687143	C	T	exonic	NDUFAF1		nonsynonymous SNV	NDUFAF1:NM_016013:exon3:c.G673A:p.E	NOVEL		199	13	0.216068	1	331	4	0.042711	1
36	chr8	21931288	21931288	G	A	exonic	DMTN		synonymous SNV	DMTN:NM_001323400:exon7:c.G657A:p.K	NOVEL		217	14	0.152944	1	329	5	0.0385786	1
36	chr4	20985535	20985535	T	A	exonic	KCNIP4		nonsynonymous SNV	KCNIP4:NM_001363504:exon1:c.A14T:p.E5	NOVEL		168	11	0.15508	1	273	4	0.0372137	1
36	chr11	290534	290534	G	C	exonic	PGGHG		nonsynonymous SNV	PGGHG:NM_025092:exon3:c.G404C:p.R13	NOVEL		161	23	0.315447	1	209	3	0.0364677	1
36	chr19	9028300	9028300	C	G	exonic	MUC16		nonsynonymous SNV	MUC16:NM_024690:exon11:c.G36492C:p.	NOVEL		188	8	0.14382	1	293	3	0.0362539	1
36	chr22	42032542	42032542	T	A	exonic	XRCC6		synonymous SNV	XRCC6:NM_001288978:exon4:c.T207A:p.L	NOVEL		124	7	0.134848	1	215	3	0.035464	1
36	chr2	136691501	136691501	C	T	exonic	DARS		synonymous SNV	DARS:NM_001293312:exon5:c.G183A:p.R	NOVEL		132	16	0.272819	1	224	3	0.0340579	1
36	chr5	133509701	133509701	T	G	exonic	SKP1		nonsynonymous SNV	SKP1:NM_006930:exon2:c.A13C:p.K5Q,SKP	NOVEL		73	8	0.348008	1	105	1	0.0337457	1
36	chr3	27427445	27427445	C	G	exonic	SLC4A7		nonsynonymous SNV	SLC4A7:NM_001258379:exon22:c.G3046C	NOVEL		171	17	0.228196	1	305	4	0.0333599	1
36	chr11	428490	428490	G	T	exonic	ANO9		synonymous SNV	ANO9:NM_001347882:exon12:c.C738A:p.I	NOVEL		225	22	0.224772	1	309	4	0.0329335	1
36	chr19	7964345	7964345	C	T	exonic	LRRC8E		nonsynonymous SNV	LRRC8E:NM_001268285:exon2:c.C551T:p.	NOVEL		309	10	0.110457	1	433	4	0.0327419	1
36	chr3	169550794	169550794	G	T	exonic	LRRIQ4		synonymous SNV	LRRIQ4:NM_001080460:exon4:c.G1353T:p	NOVEL		111	13	0.264569	1	237	3	0.0322131	1
36	chr2	26533657	26533657	G	C	exonic	ADGRF3		nonsynonymous SNV	ADGRF3:NM_153835:exon8:c.C2342G:p.P	NOVEL		124	7	0.134848	1	160	2	0.0318154	1
36	chr8	89180174	89180174	C	G	exonic	MMP16		nonsynonymous SNV	MMP16:NM_005941:exon4:c.G433C:p.D14	NOVEL		127	7	0.131829	1	248	3	0.0308014	1
36	chr8	122627193	122627193	G	T	exonic	HAS2		nonsynonymous SNV	HAS2:NM_005328:exon4:c.C815A:p.S272Y	NOVEL		392	41	0.238953	1	683	8	0.0298356	1
36	chr4	71555466	71555466	G	C	exonic	UTP3		nonsynonymous SNV	UTP3:NM_020368:exon1:c.G1072C:p.A358	NOVEL		224	11	0.118125	1	349	4	0.0292017	1
36	chr2	233408033	233408033	C	A	exonic	CHRNG		nonsynonymous SNV	CHRNG:NM_005199:exon8:c.C854A:p.T28	NOVEL		136	8	0.140199	1	176	2	0.0289556	1
36	chr13	88329707	88329707	C	T	exonic	SLITRK5		synonymous SNV	SLITRK5:NM_015567:exon2:c.C2064T:p.V6	COSMIC	ID=COSM733094	322	33	0.234586	1	449	5	0.0283816	1
36	chr12	2613633	2613633	G	T	exonic	CACNA1C		nonsynonymous SNV	CACNA1C:NM_001129840:exon8:c.G1145T	COSMIC	ID=COSM689992	271	9	0.081115	1	287	3	0.0266591	1
36	chr17	27381593	27381593	G	A	exonic	PIPOX		nonsynonymous SNV	PIPOX:NM_016518:exon5:c.G691A:p.E231	NOVEL		288	20	0.163869	1	316	3	0.0242356	1
36	chr13	61084812	61084812	T	C	exonic	TDRD3		nonsynonymous SNV	TDRD3:NM_001146070:exon10:c.T1064C:p	NOVEL		233	22	0.21772	1	323	3	0.0237152	1
36	chr1	44451265	44451265	G	A	exonic	B4GALT2		nonsynonymous SNV	B4GALT2:NM_001005417:exon6:c.G940A:	COSMIC	ID=COSM217140	424	42	0.227447	1	563	5	0.0226853	1
36	chr8	8865516	8865516	A	G	exonic	ERI1		nonsynonymous SNV	ERI1:NM_001354638:exon2:c.A145G:p.K4	NOVEL		144	15	0.238073	1	227	2	0.022507	1
36	chr11	48266662	48266662	G	A	exonic	OR4X2		nonsynonymous SNV	OR4X2:NM_001004727:exon1:c.G7A:p.E3K	COSMIC	ID=COSM441292	159	19	0.269371	1	239	2	0.0213863	1
36	chr7	134717686	134717686	G	A	exonic	AGBL3		nonsynonymous SNV	AGBL3:NM_178563:exon6:c.G510A:p.M17	NOVEL		176	15	0.198187	1	386	3	0.0198744	1
36	chr17	73738446	73738446	A	T	exonic	ITGB4		synonymous SNV	ITGB4:NM_001005619:exon23:c.A2658T:p	NOVEL		318	10	0.0769383	1	428	3	0.0179377	1
36	chr11	32417881	32417881	A	C	exonic	WT1		nonsynonymous SNV	WT1:NM_000378:exon6:c.T1135G:p.F379	NOVEL		97	6	0.147005	1	145	1	0.017651	1
36	chr19	2763725	2763725	G	T	exonic	SGTA		stopgain	SGTA:NM_003021:exon6:c.C423A:p.Y141X	NOVEL		161	19	0.371933	1	203	1	0.0175346	1
36	chr16	50745632	50745632	A	G	exonic	NOD2		nonsynonymous SNV	NOD2:NM_001293557:exon3:c.A1729G:p.	DBSNP		118	11	0.215189	1	147	1	0.0174125	1
36	chr2	1926854	1926854	G	C	exonic	MYT1L		synonymous SNV	MYT1L:NM_001303052:exon10:c.C687G:p	NOVEL		269	31	0.26077	1	460	3	0.0166979	1
36	chr2	136872782	136872782	T	G	exonic	CXCR4		nonsynonymous SNV	CXCR4:NM_001008540:exon1:c.A728C:p.K	NOVEL		97	11	0.257031	1	167	1	0.0153396	1
36	chr1	28209411	28209411	G	C	exonic	THEMIS2		nonsynonymous SNV	THEMIS2:NM_001105556:exon4:c.G1576C	NOVEL		154	14	0.210298	1	181	1	0.0141596	1
36	chr11	18735441	18735441	C	G	exonic	IGSF22		nonsynonymous SNV	IGSF22:NM_173588:exon14:c.G2053C:p.D	NOVEL		129	15	0.262873	1	183	1	0.0140057	1
36	chr9	35650377	35650377	C	G	exonic	SIT1		nonsynonymous SNV	SIT1:NM_014450:exon3:c.G272C:p.G91A	NOVEL		250	19	0.319509	1	307	1	0.0128226	1
36	chr2	152397256	152397256	G	C	exonic	NEB		synonymous SNV	NEB:NM_004543:exon110:c.C15639G:p.T5	NOVEL		148	8	0.129414	1	242	1	0.0106051	1
36	chr3	123301170	123301170	C	G	exonic	HACD2		synonymous SNV	HACD2:NM_001329783:exon2:c.G162C:p.L	NOVEL		320	36	0.255193	1	556	2	0.00923674	1
36	chr20	31024159	31024159	C	G	exonic	ASXL1		nonsynonymous SNV	ASXL1:NM_001363734:exon11:c.C3461G:p	NOVEL		208	14	0.159145	1	307	1	0.00836704	1
36	chr1	214542895	214542895	T	G	exonic	PTPN14		nonsynonymous SNV	PTPN14:NM_005401:exon17:c.A3176C:p.K	NOVEL		275	9	0.111663	1	442	1	0.0080746	1
36	chr15	45707837	45707837	T	A	exonic	SPATA5L1		nonsynonymous SNV	SPATA5L1:NM_001323640:exon5:c.T1697	NOVEL		269	22	0.266387	1	456	1	0.00782724	1
36	chr9	32632927	32632927	T	A	exonic	TAF1L		nonsynonymous SNV	TAF1L:NM_153809:exon1:c.A2651T:p.K88	NOVEL		420	17	0.175974	1	615	1	0.00641131	1
36	chr1	10067707	10067707	C	T	exonic	RBP7		synonymous SNV	RBP7:NM_052960:exon2:c.C153T:p.T51T	NOVEL		490	26	0.127157	1	685	1	0.00375663	1
36	chr12	123351827	123351827	C	G	exonic	VPS37B		nonsynonymous SNV	VPS37B:NM_024667:exon4:c.G694C:p.A2	NOVEL		38	8	0.438883	1	58	0	0	1
36	chr2	131883425	131883425	A	T	exonic	PLEKHB2		nonsynonymous SNV	PLEKHB2:NM_001100623:exon3:c.A137T:p	NOVEL		34	7	0.430855	1	75	0	0	1
36	chr4	76442161	76442161	C	T	exonic	THAP6		nonsynonymous SNV	THAP6:NM_001317791:exon3:c.C260T:p.S	NOVEL		66	8	0.272819	1	143	0	0	1
36	chr7	135047737	135047737	G	C	exonic	CNOT4		nonsynonymous SNV	CNOT4:NM_001190847:exon11:c.C1829G:	NOVEL		34	4	0.26564	1	69	0	0	1
36	chr18	51820827	51820827	G	T	exonic	POLI		nonsynonymous SNV	POLI:NM_001351616:exon8:c.G1850T:p.G	NOVEL		27	3	0.252358	1	94	0	0	1
36	chr15	40659622	40659622	C	A	exonic	DISP2		nonsynonymous SNV	DISP2:NM_033510:exon8:c.C1309A:p.L437	NOVEL		282	20	0.23335	1	422	0	0	1
36	chr10	69908221	69908221	G	A	exonic	MYPN		synonymous SNV	MYPN:NM_032578:exon5:c.G1242A:p.Q41	NOVEL		137	12	0.203241	1	240	0	0	1
36	chr11	65546069	65546069	C	T	exonic	AP5B1		nonsynonymous SNV	AP5B1:NM_138368:exon2:c.G1895A:p.G6	NOVEL		60	5	0.194121	1	106	0	0	1
36	chr20	22563064	22563064	G	C	exonic	FOXA2		synonymous SNV	FOXA2:NM_021784:exon2:c.C816G:p.A272	NOVEL		51	4	0.183533	1	72	0	0	1
36	chr5	13901618	13901618	C	A	exonic	DNAH5		nonsynonymous SNV	DNAH5:NM_001369:exon14:c.G1795T:p.D	NOVEL		245	13	0.177545	1	335	0	0	1
36	chr2	20870388	20870388	C	T	exonic	GDF7		nonsynonymous SNV	GDF7:NM_182828:exon2:c.C556T:p.P186S	NOVEL		82	6	0.172062	1	98	0	0	1
36	chr3	141161406	141161406	A	G	exonic	ZBTB38		nonsynonymous SNV	ZBTB38:NM_001350100:exon4:c.A176G:p	NOVEL		146	10	0.161768	1	265	0	0	1
36	chr2	234296895	234296895	A	C	exonic	DGKD		nonsynonymous SNV	DGKD:NM_003648:exon1:c.A17C:p.Y6S	NOVEL		262	17	0.153766	1	360	0	0	1
36	chr15	45968394	45968394	C	T	exonic	SQOR		synonymous SNV	SQOR:NM_021199:exon6:c.C750T:p.I250I,	NOVEL		178	8	0.151552	1	243	0	0	1
36	chr1	201970787	201970787	G	A	exonic	RNPEP		nonsynonymous SNV	RNPEP:NM_001319183:exon7:c.G448A:p.	NOVEL		158	7	0.149485	1	257	0	0	1
36	chr19	45381826	45381826	G	T	exonic	NECTIN2		nonsynonymous SNV	NECTIN2:NM_002856:exon6:c.G1389T:p.M	NOVEL		710	31	0.147061	1	748	0	0	1
36	chr11	8734122	8734122	G	A	exonic	ST5		synonymous SNV	ST5:NM_139157:exon8:c.C888T:p.P296P,S	DBSNP		126	7	0.13282	1	173	0	0	1
36	chr1	17708551	17708551	G	T	exonic	PADI6		stopgain	PADI6:NM_207421:exon6:c.G643T:p.E215	NOVEL		238	13	0.130703	1	340	0	0	1
36	chr22	50898773	50898773	T	G	exonic	SBF1		nonsynonymous SNV	SBF1:NM_001365819:exon25:c.A3214C:p.	NOVEL		189	10	0.126813	1	326	0	0	1
36	chr1	78430794	78430794	A	T	exonic	FUBP1		nonsynonymous SNV	FUBP1:NM_003902:exon8:c.T595A:p.L199	NOVEL		395	20	0.121618	1	603	0	0	1
36	chr20	3147361	3147361	T	G	exonic	LZTS3		nonsynonymous SNV	LZTS3:NM_001282533:exon3:c.A449C:p.K	NOVEL		122	6	0.118293	1	146	0	0	1
36	chr11	64135702	64135702	G	A	exonic	RPS6KA4		synonymous SNV	RPS6KA4:NM_001006944:exon10:c.G1170	NOVEL		144	7	0.116987	1	233	0	0	1
36	chr11	118984616	118984616	G	A	exonic	C2CD2L		nonsynonymous SNV	C2CD2L:NM_001290474:exon12:c.G1541A	DBSNP+COSMIC	ID=COSM210644	189	9	0.114708	1	290	0	0	1
36	chr16	23635389	23635389	C	T	exonic	PALB2		synonymous SNV	PALB2:NM_024675:exon8:c.G2775A:p.V92	NOVEL		276	13	0.113517	1	444	0	0	1
36	chr10	105365618	105365618	G	C	exonic	SH3PXD2A		synonymous SNV	SH3PXD2A:NM_001365079:exon8:c.C1008	NOVEL		131	6	0.110522	1	167	0	0	1
36	chr2	128408535	128408535	G	C	exonic	GPR17		nonsynonymous SNV	GPR17:NM_001161417:exon2:c.G226C:p.	NOVEL		221	10	0.109246	1	336	0	0	1
36	chr11	111156719	111156719	T	G	exonic	C11orf53		synonymous SNV	C11orf53:NM_198498:exon5:c.T807G:p.P2	DBSNP		181	8	0.106818	1	299	0	0	1
36	chr5	167689759	167689759	G	C	exonic	TENM2		nonsynonymous SNV	TENM2:NM_001080428:exon25:c.G7552C	NOVEL		320	10	0.106775	1	371	0	0	1
36	chr12	52448389	52448389	G	T	exonic	NR4A1		nonsynonymous SNV	NR4A1:NM_173157:exon2:c.G277T:p.A93	NOVEL		260	11	0.102433	1	354	0	0	1
36	chr11	5700387	5700387	C	T	exonic	TRIM5		nonsynonymous SNV	TRIM5:NM_033034:exon3:c.G430A:p.A14	NOVEL		193	8	0.100441	1	275	0	0	1
36	chr2	32640614	32640614	G	A	exonic	BIRC6		nonsynonymous SNV	BIRC6:NM_016252:exon10:c.G2255A:p.C7	DBSNP+COSMIC	ID=COSM683553	290	12	0.100275	1	436	0	0	1
36	chr7	21640548	21640548	G	A	exonic	DNAH11		synonymous SNV	DNAH11:NM_001277115:exon16:c.G3255	NOVEL		347	14	0.0978673	1	524	0	0	1
36	chr22	24583281	24583281	C	A	exonic	SUSD2		nonsynonymous SNV	SUSD2:NM_019601:exon11:c.C1754A:p.P	NOVEL		233	9	0.0938521	1	249	0	0	1
36	chr11	56085973	56085973	G	C	exonic	OR8K3		nonsynonymous SNV	OR8K3:NM_001005202:exon1:c.G191C:p.	NOVEL		234	9	0.0934658	1	400	0	0	1
36	chr2	217559200	217559200	C	G	exonic	IGFBP5		nonsynonymous SNV	IGFBP5:NM_000599:exon1:c.G299C:p.C10	NOVEL		290	11	0.0922238	1	278	0	0	1
36	chr12	6700676	6700676	C	T	exonic	CHD4		nonsynonymous SNV	CHD4:NM_001297553:exon21:c.G3275A:p	NOVEL		211	8	0.0921855	1	297	0	0	1
36	chr1	66102079	66102079	A	G	exonic	LEPR		nonsynonymous SNV	LEPR:NM_002303:exon20:c.A2879G:p.N96	NOVEL		279	10	0.087321	1	428	0	0	1
36	chr3	128707603	128707603	C	T	exonic	KIAA1257		nonsynonymous SNV	KIAA1257:NM_001348523:exon2:c.G85A:p	NOVEL		396	14	0.0861709	1	593	0	0	1
36	chr20	56082831	56082831	G	C	exonic	CTCFL		nonsynonymous SNV	CTCFL:NM_001269048:exon6:c.C1260G:p.	NOVEL		286	10	0.085256	1	399	0	0	1
36	chr7	5569295	5569295	C	A	splicing	ACTB	NM_001101:exon2:UT			DBSNP		7	6	1.16473	1	26	25	1.26326	1
36	chr9	95236904	95236904	A	G	splicing	ASPN	NM_017680:exon3:c.2			NOVEL		198	4	0.0895758	1	277	37	0.539334	1
36	chr15	43170884	43170884	T	G	splicing	TTBK2	NM_173500:exon2:UT			NOVEL		52	0	0	1	102	12	0.376531	1
36	chr20	35852280	35852280	G	T	splicing	RPN2	NM_001324302:exon1			NOVEL		337	13	0.0937329	1	503	0	0	1
39	chrX	73965423	73965423	A	T	exonic	NEXMIF		nonsynonymous SNV	NEXMIF:NM_001008537:exon2:c.T63A:p.N	NOVEL		19	56	NA	1	43	38	NA	1
39	chrX	77112310	77112310	C	T	exonic	MAGT1		nonsynonymous SNV	MAGT1:NM_001367916:exon5:c.G592A:p.	COSMIC	ID=COSM455634	41	91	NA	1	53	46	NA	1
39	chr3	169847060	169847060	C	T	exonic	PHC3		synonymous SNV	PHC3:NM_001308116:exon8:c.G1164A:p.Q	NOVEL		221	0	NA	1	90	26	1.03414	1
39	chr11	64893300	64893300	T	C	exonic	MRPL49		nonsynonymous SNV	MRPL49:NM_004927:exon4:c.T457C:p.F15	NOVEL		15	8	1.25036	1	13	2	0.615183	1
39	chr4	125590986	125590986	G	C	exonic	ANKRD50		nonsynonymous SNV	ANKRD50:NM_001167882:exon3:c.C2909G	NOVEL		158	123	1.1358	1	148	58	1.0175	1
39	chr9	17330640	17330640	G	A	exonic	CNTLN		nonsynonymous SNV	CNTLN:NM_001365029:exon9:c.G1352A:p	DBSNP+COSMIC	ID=COSM110752	27	21	1.13522	1	17	5	0.821335	1
39	chr2	179429065	179429065	G	T	exonic	TTN		nonsynonymous SNV	TTN:NM_003319:exon154:c.C54599A:p.T1	NOVEL		141	105	1.10753	1	94	40	1.07877	1
39	chr14	91791243	91791243	T	C	exonic	CCDC88C		nonsynonymous SNV	CCDC88C:NM_001080414:exon12:c.A1222	NOVEL		112	83	1.10445	1	88	33	0.985601	1
39	chr5	64848357	64848357	A	C	exonic	CENPK		nonsynonymous SNV	CENPK:NM_001267038:exon4:c.T131G:p.L	NOVEL		76	32	1.06512	1	74	12	0.643796	1
39	chr19	41950108	41950108	T	A	exonic	ERICH4		synonymous SNV	ERICH4:NM_001130514:exon2:c.T372A:p.	NOVEL		106	47	1.0516	1	55	25	1.12933	1
39	chr12	25378561	25378561	G	A	exonic	KRAS		nonsynonymous SNV	KRAS:NM_001369786:exon4:c.C437T:p.A1	DBSNP+COSMIC	ID=COSM19900,	18	23	1.04862	1	38	28	0.766579	2
39	chr2	120362802	120362802	A	C	exonic	CFAP221		nonsynonymous SNV	CFAP221:NM_001271049:exon11:c.A1070	NOVEL		178	120	1.04488	1	164	67	1.04818	1
39	chr1	202245558	202245558	C	T	exonic	LGR6		nonsynonymous SNV	LGR6:NM_001017403:exon5:c.C553T:p.L1	NOVEL		274	180	1.02877	1	349	77	0.96786	1
39	chr11	8667354	8667354	C	T	exonic	TRIM66		synonymous SNV	TRIM66:NM_014818:exon7:c.G603A:p.V20	DBSNP		71	95	1.02863	2	72	44	0.875045	2
39	chr5	16465471	16465471	C	T	exonic	ZNF622		nonsynonymous SNV	ZNF622:NM_033414:exon1:c.G304A:p.V10	NOVEL		313	121	1.00223	1	226	54	0.889818	1
39	chr2	131522046	131522046	G	T	exonic	AMER3		nonsynonymous SNV	AMER3:NM_001105193:exon2:c.G2401T:p	NOVEL		43	27	1.00085	1	26	8	0.850323	1
39	chr15	90335434	90335434	G	A	exonic	ANPEP		nonsynonymous SNV	ANPEP:NM_001150:exon18:c.C2485T:p.R8	DBSNP		167	101	0.977887	1	115	50	1.09511	1
39	chr17	65720207	65720207	A	T	exonic	NOL11		nonsynonymous SNV	NOL11:NM_001303272:exon5:c.A16T:p.I6	NOVEL		43	16	0.974858	1	35	9	0.943747	1
39	chr19	36636935	36636935	C	G	exonic	CAPNS1		nonsynonymous SNV	CAPNS1:NM_001302633:exon7:c.C247G:p	NOVEL		240	94	0.963438	1	104	42	1.03961	1
39	chr1	154317837	154317837	T	G	exonic	ATP8B2		nonsynonymous SNV	ATP8B2:NM_001367934:exon22:c.T2567G	NOVEL		39	22	0.935826	1	48	10	0.962105	1
39	chr11	118240176	118240176	C	T	exonic	UBE4A		nonsynonymous SNV	UBE4A:NM_001204077:exon4:c.C334T:p.R	DBSNP		193	209	0.934467	2	149	77	0.785991	2
39	chr11	64064654	64064654	T	C	exonic	KCNK4		nonsynonymous SNV	KCNK4:NM_001317090:exon4:c.T377C:p.V	NOVEL		198	68	0.918969	1	130	32	0.911382	1
39	chr19	44605298	44605298	T	G	exonic	ZNF224		nonsynonymous SNV	ZNF224:NM_001321645:exon5:c.T155G:p.	NOVEL		90	33	0.918441	1	47	15	0.874324	1
39	chr5	26906906	26906906	C	A	exonic	CDH9		nonsynonymous SNV	CDH9:NM_016279:exon4:c.G565T:p.A189	NOVEL		347	119	0.917983	1	290	67	0.865909	1
39	chr2	28764628	28764628	A	G	exonic	PLB1		nonsynonymous SNV	PLB1:NM_001170585:exon13:c.A862G:p.T	NOVEL		274	149	0.914004	1	192	56	0.816036	1
39	chr22	29692342	29692342	C	T	exonic	EWSR1		synonymous SNV	EWSR1:NM_001163286:exon11:c.C1110T:	DBSNP		56	30	0.905159	1	51	21	1.05405	1
39	chr1	28148762	28148762	G	T	exonic	STX12		nonsynonymous SNV	STX12:NM_177424:exon9:c.G753T:p.M25	NOVEL		145	76	0.892326	1	123	47	0.999129	1
39	chr19	49254006	49254006	C	T	exonic	FUT1		nonsynonymous SNV	FUT1:NM_000148:exon4:c.G533A:p.R178	NOVEL		177	191	0.888378	2	127	41	0.881957	1
39	chr17	65720246	65720246	A	G	exonic	NOL11		nonsynonymous SNV	NOL11:NM_001303272:exon5:c.A55G:p.N	NOVEL		64	21	0.888125	1	48	12	0.922774	1
39	chr19	3534797	3534797	C	G	exonic	FZR1		nonsynonymous SNV	FZR1:NM_001136197:exon11:c.C1178G:p.	NOVEL		331	112	0.865479	1	207	0	0	1
39	chr1	160252834	160252834	T	C	exonic	PEX19		synonymous SNV	PEX19:NM_001193644:exon3:c.A246G:p.Q	NOVEL		37	18	0.849204	1	42	17	0.803929	2
39	chr5	72151737	72151737	T	A	exonic	TNPO1		synonymous SNV	TNPO1:NM_001364292:exon4:c.T318A:p.I	NOVEL		52	46	0.843675	2	42	25	0.860797	2
39	chr5	72151738	72151738	A	T	exonic	TNPO1		stopgain	TNPO1:NM_001364292:exon4:c.A319T:p.R	NOVEL		52	46	0.843675	2	43	25	0.848138	2
39	chr5	140229950	140229950	G	A	exonic	PCDHA9		nonsynonymous SNV	PCDHA9:NM_014005:exon1:c.G1870A:p.V	DBSNP+COSMIC	ID=COSM385108	169	50	0.820728	1	128	0	0	1
39	chr2	120718469	120718469	G	A	exonic	PTPN4		nonsynonymous SNV	PTPN4:NM_002830:exon23:c.G2220A:p.M	NOVEL		66	29	0.792094	1	64	0	0	1
39	chr16	31226188	31226188	G	T	exonic	TRIM72		synonymous SNV	TRIM72:NM_001008274:exon2:c.G129T:p.	NOVEL		35	15	0.778437	1	30	0	0	1
39	chr17	48941308	48941308	C	G	exonic	TOB1		nonsynonymous SNV	TOB1:NM_005749:exon2:c.G71C:p.R24P,T	NOVEL		162	44	0.767819	1	150	0	0	1
39	chr19	44008065	44008065	C	T	exonic	PHLDB3		nonsynonymous SNV	PHLDB3:NM_198850:exon2:c.G206A:p.R6	DBSNP		100	28	0.748842	1	64	0	0	1
39	chr2	179575542	179575542	C	G	exonic	TTN		nonsynonymous SNV	TTN:NM_133378:exon95:c.G24550C:p.V8	NOVEL		93	37	0.738517	1	82	0	0	1
39	chr19	8155130	8155130	G	T	exonic	FBN3		synonymous SNV	FBN3:NM_001321431:exon49:c.C6037A:p.	DBSNP		241	69	0.710974	1	266	1	0.0208997	1
39	chr4	1730378	1730378	C	A	exonic	TACC3		nonsynonymous SNV	TACC3:NM_006342:exon4:c.C1249A:p.P41	NOVEL		160	58	0.690357	1	169	1	0.0212581	1
39	chr8	101936504	101936504	T	C	exonic	YWHAZ		synonymous SNV	YWHAZ:NM_001135699:exon4:c.A441G:p.	NOVEL		253	89	0.675252	1	234	0	0	1
39	chr4	24801363	24801363	G	A	exonic	SOD3		nonsynonymous SNV	SOD3:NM_003102:exon2:c.G220A:p.A74T	NOVEL		79	27	0.660937	1	86	0	0	1
39	chr6	54001603	54001603	G	A	exonic	MLIP		nonsynonymous SNV	MLIP:NM_001281746:exon4:c.G703A:p.D2	NOVEL		164	55	0.65166	1	183	0	0	1
39	chr20	18395111	18395111	T	C	exonic	DZANK1		nonsynonymous SNV	DZANK1:NM_018152:exon11:c.A604G:p.S	NOVEL		115	34	0.5921	1	128	0	0	1
39	chr17	53499052	53499052	C	G	exonic	MMD		nonsynonymous SNV	MMD:NM_012329:exon1:c.G5C:p.R2P	NOVEL		194	35	0.549422	1	137	15	0.455316	1
39	chr5	76785383	76785383	C	A	exonic	WDR41		nonsynonymous SNV	WDR41:NM_018268:exon2:c.G66T:p.Q22H	NOVEL		177	30	0.520984	1	129	1	0.0354913	1
39	chr10	120354350	120354350	A	T	exonic	PRLHR		nonsynonymous SNV	PRLHR:NM_004248:exon2:c.T407A:p.L136	NOVEL		150	37	0.513408	1	126	0	0	1
39	chr6	168434686	168434686	C	T	exonic	KIF25		nonsynonymous SNV	KIF25:NM_005355:exon4:c.C292T:p.P98S,	DBSNP		43	11	0.38674	1	40	14	0.93693	1
39	chr15	83933151	83933151	G	A	exonic	BNC1		synonymous SNV	BNC1:NM_001301206:exon4:c.C831T:p.F2	NOVEL		87	15	0.381587	1	63	16	0.731923	1
39	chr7	48494754	48494754	A	G	exonic	ABCA13		nonsynonymous SNV	ABCA13:NM_152701:exon43:c.A12686G:p	NOVEL		204	27	0.303287	1	122	40	0.892314	1
39	chr3	53857592	53857592	C	T	exonic	CHDH		synonymous SNV	CHDH:NM_018397:exon3:c.G444A:p.E148	NOVEL		80	7	0.289236	1	44	12	0.988687	1
39	chr22	29907268	29907268	G	T	exonic	THOC5		synonymous SNV	THOC5:NM_001002879:exon19:c.C1815A:	NOVEL		40	5	0.28831	1	40	0	0	1
39	chr19	56549507	56549507	T	C	exonic	NLRP5		nonsynonymous SNV	NLRP5:NM_153447:exon10:c.T2732C:p.V9	NOVEL		210	19	0.284028	1	109	0	0	1
39	chr19	5844722	5844722	T	A	exonic	FUT3		synonymous SNV	FUT3:NM_001097641:exon2:c.A129T:p.G4	NOVEL		399	26	0.281093	1	303	31	0.706677	1
39	chr19	5844733	5844733	C	A	exonic	FUT3		nonsynonymous SNV	FUT3:NM_001097641:exon2:c.G118T:p.D4	NOVEL		428	27	0.272658	1	319	36	0.772111	1
39	chr22	39812840	39812840	C	T	exonic	TAB1		synonymous SNV	TAB1:NM_006116:exon4:c.C369T:p.D123D	DBSNP		111	13	0.272034	1	91	0	0	1
39	chr19	5844586	5844586	C	A	exonic	FUT3		nonsynonymous SNV	FUT3:NM_001097641:exon2:c.G265T:p.A8	DBSNP		448	28	0.270282	1	324	44	0.910354	1
39	chr4	62897256	62897256	C	T	exonic	ADGRL3		synonymous SNV	ADGRL3:NM_001371342:exon18:c.C3234T	NOVEL		152	17	0.261014	1	170	0	0	1
39	chr19	5844604	5844604	C	G	exonic	FUT3		nonsynonymous SNV	FUT3:NM_001097641:exon2:c.G247C:p.E8	NOVEL		425	25	0.255266	1	297	41	0.923576	1
39	chr5	21817067	21817067	A	C	exonic	CDH12		nonsynonymous SNV	CDH12:NM_001364109:exon4:c.T317G:p.	NOVEL		374	26	0.233661	1	244	56	0.861256	1
39	chr12	56740617	56740617	T	C	exonic	STAT2		nonsynonymous SNV	STAT2:NM_005419:exon20:c.A1847G:p.E6	NOVEL		186	18	0.228952	1	206	0	0	1
39	chr4	62897290	62897290	A	T	exonic	ADGRL3		nonsynonymous SNV	ADGRL3:NM_001371342:exon18:c.A3268T	NOVEL		117	11	0.22299	1	123	0	0	1
39	chr2	169758023	169758023	C	T	exonic	G6PC2		nonsynonymous SNV	G6PC2:NM_001081686:exon1:c.C182T:p.A	DBSNP		66	6	0.216233	1	60	0	0	1
39	chr8	28828020	28828020	G	A	exonic	HMBOX1		nonsynonymous SNV	HMBOX1:NM_001135726:exon3:c.G484A:	NOVEL		72	6	0.199599	1	63	0	0	1
39	chr17	7258658	7258658	T	C	exonic	TMEM95		synonymous SNV	TMEM95:NM_001320435:exon1:c.T135C:p	NOVEL		240	14	0.198138	1	148	0	0	1
39	chr14	45398280	45398280	G	A	exonic	KLHL28		nonsynonymous SNV	KLHL28:NM_001308112:exon5:c.C1709T:p	NOVEL		399	32	0.192653	1	319	0	0	1
39	chr19	5844517	5844517	C	G	exonic	FUT3		nonsynonymous SNV	FUT3:NM_001097641:exon2:c.G334C:p.D1	NOVEL		480	21	0.192596	1	359	46	0.864785	1
39	chr19	5844456	5844456	C	G	exonic	FUT3		nonsynonymous SNV	FUT3:NM_001097641:exon2:c.G395C:p.W	NOVEL		383	16	0.184252	1	267	29	0.745954	1
39	chr19	5844467	5844467	C	T	exonic	FUT3		synonymous SNV	FUT3:NM_001097641:exon2:c.G384A:p.Q	NOVEL		413	17	0.181654	1	283	36	0.859246	1
39	chr11	1093676	1093676	G	T	exonic	MUC2		nonsynonymous SNV	MUC2:NM_002457:exon31:c.G5495T:p.S1	NOVEL		604	30	0.1701	1	380	0	0	1
39	chr4	52943162	52943162	G	A	exonic	SPATA18		nonsynonymous SNV	SPATA18:NM_001346103:exon3:c.G301A:	DBSNP+COSMIC	ID=COSM105593	174	12	0.167406	1	137	0	0	1
39	chr11	55371404	55371404	C	G	exonic	OR4C11		nonsynonymous SNV	OR4C11:NM_001004700:exon1:c.G446C:p	NOVEL		312	15	0.164899	1	256	0	0	1
39	chr1	118165902	118165902	T	C	exonic	FAM46C		nonsynonymous SNV	FAM46C:NM_017709:exon2:c.T412C:p.Y13	NOVEL		137	9	0.159953	1	128	0	0	1
39	chr19	5844439	5844439	C	T	exonic	FUT3		nonsynonymous SNV	FUT3:NM_001097641:exon2:c.G412A:p.E1	NOVEL		354	12	0.150649	1	240	29	0.820826	1
39	chr12	73015469	73015469	C	A	exonic	TRHDE		nonsynonymous SNV	TRHDE:NM_013381:exon15:c.C2478A:p.N	NOVEL		135	8	0.145163	1	152	0	0	1
39	chr1	118165901	118165901	A	G	exonic	FAM46C		synonymous SNV	FAM46C:NM_017709:exon2:c.A411G:p.A1	DBSNP		137	8	0.143161	1	128	0	0	1
39	chr19	38028084	38028084	G	A	exonic	ZNF793		nonsynonymous SNV	ZNF793:NM_001013659:exon8:c.G524A:p	DBSNP+COSMIC	ID=COSM320092	462	20	0.142045	1	234	0	0	1
39	chr1	34037250	34037250	C	T	exonic	CSMD2		synonymous SNV	CSMD2:NM_001281956:exon51:c.G7839A	NOVEL		75	4	0.131382	1	39	23	1.34063	1
39	chr22	29904458	29904458	G	A	exonic	THOC5		synonymous SNV	THOC5:NM_001002879:exon20:c.C2040T:p	NOVEL		146	7	0.118716	1	97	0	0	1
39	chr6	43106948	43106948	C	T	exonic	PTK7		synonymous SNV	PTK7:NM_001270398:exon9:c.C1461T:p.Y4	NOVEL		355	17	0.118579	1	286	0	0	1
39	chr4	15689016	15689016	C	T	exonic	FAM200B		nonsynonymous SNV	FAM200B:NM_001145191:exon2:c.C416T:	NOVEL		117	5	0.106344	1	78	15	0.582883	1
39	chr19	40902819	40902819	C	A	exonic	PRX		nonsynonymous SNV	PRX:NM_181882:exon7:c.G1440T:p.K480N	NOVEL		363	9	0.0828213	1	136	56	1.05405	1
39	chr18	21441744	21441744	C	T	exonic	LAMA3		synonymous SNV	LAMA3:NM_001127717:exon35:c.C4557T:	DBSNP		97	3	0.0778437	1	44	19	1.39149	1
39	chr1	220936274	220936274	C	T	exonic	Mar-02		nonsynonymous SNV	MARC2:NM_001317338:exon4:c.C632T:p.	DBSNP		85	2	0.0596503	1	63	18	1.0253	1
39	chr13	31724317	31724317	G	T	exonic	HSPH1		stopgain	HSPH1:NM_001286505:exon6:c.C683A:p.S	NOVEL		129	1	0.0199599	1	53	21	1.02556	1
39	chr2	197987480	197987480	C	T	exonic	ANKRD44		nonsynonymous SNV	ANKRD44:NM_001195144:exon7:c.G587A	NOVEL		134	1	0.0192207	1	55	19	0.927886	1
39	chr1	85506767	85506767	C	A	exonic	MCOLN3		nonsynonymous SNV	MCOLN3:NM_018298:exon3:c.G322T:p.D1	COSMIC	ID=COSM584740	165	1	0.0156313	1	85	23	0.769621	1
39	chr2	85836146	85836146	C	T	exonic	C2orf68		synonymous SNV	C2orf68:NM_001013649:exon4:c.G423A:p	DBSNP+COSMIC	ID=COSM340801	188	1	0.013729	1	93	25	0.765651	1
39	chr6	394966	394966	C	A	exonic	IRF4		nonsynonymous SNV	IRF4:NM_001195286:exon3:c.C362A:p.P12	COSMIC	ID=COSM637856	220	1	0.0117411	1	91	34	0.982973	1
39	chr3	179426662	179426662	G	A	exonic	USP13		nonsynonymous SNV	USP13:NM_003940:exon6:c.G722A:p.G24	DBSNP+COSMIC	ID=COSM681817	340	1	0.0105419	1	152	33	0.823015	1
39	chr11	128360417	128360417	G	C	exonic	ETS1		nonsynonymous SNV	ETS1:NM_001330451:exon2:c.C137G:p.S4	NOVEL		445	1	0.00806007	1	258	58	0.84685	1
39	chr20	25656655	25656655	C	T	exonic	ZNF337		synonymous SNV	ZNF337:NM_001290261:exon4:c.G1269A:	NOVEL		353	1	0.00732992	1	293	52	0.695424	1
39	chr1	23637060	23637060	G	A	exonic	HNRNPR		stopgain	HNRNPR:NM_001297621:exon9:c.C1309T:	NOVEL		367	0	0	1	225	69	0.848154	1
39	chr1	68611681	68611681	G	C	exonic	WLS		nonsynonymous SNV	WLS:NM_001193334:exon8:c.C876G:p.I29	NOVEL		137	0	0	1	72	23	0.874937	1
39	chr1	173454617	173454617	G	A	exonic	PRDX6		nonsynonymous SNV	PRDX6:NM_004905:exon3:c.G370A:p.E124	COSMIC	ID=COSM714457	144	0	0	1	112	25	0.977126	1
39	chr1	179561789	179561789	G	A	exonic	TDRD5		synonymous SNV	TDRD5:NM_001199085:exon2:c.G39A:p.K1	DBSNP+COSMIC	ID=COSM108352	104	0	0	1	112	17	0.705652	1
39	chr1	185112529	185112529	T	C	exonic	TRMT1L		synonymous SNV	TRMT1L:NM_001202423:exon7:c.A351G:p	DBSNP		322	0	0	1	301	96	1.29483	1
39	chr1	186141215	186141215	G	C	exonic	HMCN1		nonsynonymous SNV	HMCN1:NM_031935:exon102:c.G15766C:p	NOVEL		313	0	0	1	238	81	1.35965	1
39	chr1	192321259	192321259	T	C	exonic	RGS21		synonymous SNV	RGS21:NM_001039152:exon4:c.T171C:p.F	NOVEL		107	0	0	1	112	25	0.977126	1
39	chr1	229407098	229407098	G	A	exonic	RAB4A		synonymous SNV	RAB4A:NM_004578:exon1:c.G12A:p.T4T	NOVEL		65	0	0	1	33	9	0.988687	1
39	chr1	241951326	241951326	G	C	exonic	WDR64		nonsynonymous SNV	WDR64:NM_001367482:exon24:c.G2881C	NOVEL		32	0	0	1	23	8	1.19068	1
39	chr10	5567497	5567497	G	C	exonic	CALML3		stoploss	CALML3:NM_005185:exon1:c.G449C:p.X15	DBSNP		96	0	0	1	55	16	0.814394	1
39	chr10	16870861	16870861	G	A	exonic	CUBN		synonymous SNV	CUBN:NM_001081:exon66:c.C10707T:p.L3	NOVEL		147	0	0	1	83	16	0.58406	1
39	chr10	24722092	24722092	G	A	exonic	KIAA1217		nonsynonymous SNV	KIAA1217:NM_001282767:exon4:c.G722A	NOVEL		111	0	0	1	54	18	0.903468	1
39	chr10	33217145	33217145	G	A	exonic	ITGB1		nonsynonymous SNV	ITGB1:NM_033668:exon4:c.C424T:p.P142	NOVEL		203	0	0	1	133	38	0.803083	1
39	chr10	75567289	75567289	G	A	exonic	NDST2		synonymous SNV	NDST2:NM_001330107:exon3:c.C858T:p.F	NOVEL		146	0	0	1	75	27	0.956613	1
39	chr10	112838884	112838884	G	A	exonic	ADRA2A		stopgain	ADRA2A:NM_000681:exon1:c.G1130A:p.W	NOVEL		110	0	0	1	44	28	1.40539	1
39	chr11	314245	314245	C	T	exonic	IFITM1		synonymous SNV	IFITM1:NM_003641:exon1:c.C75T:p.I25I	NOVEL		387	0	0	1	173	61	1.20276	1
39	chr11	60198331	60198331	G	A	exonic	MS4A5		synonymous SNV	MS4A5:NM_023945:exon2:c.G216A:p.L72	NOVEL		137	0	0	1	71	17	0.891316	1
39	chr11	77885498	77885498	C	T	exonic	KCTD21		nonsynonymous SNV	KCTD21:NM_001029859:exon2:c.G103A:p	DBSNP+COSMIC	ID=COSM475784	355	0	0	1	134	45	1.15991	1
39	chr11	104877891	104877891	A	G	exonic	CASP5		nonsynonymous SNV	CASP5:NM_001136109:exon2:c.T178C:p.S	NOVEL		525	0	0	1	313	66	0.803471	1
39	chr12	9096053	9096053	C	T	exonic	M6PR		nonsynonymous SNV	M6PR:NM_002355:exon5:c.G532A:p.A178	NOVEL		147	0	0	1	142	46	0.884245	1
39	chr12	28116616	28116616	C	T	exonic	PTHLH		synonymous SNV	PTHLH:NM_198964:exon3:c.G189A:p.L63L	NOVEL		193	0	0	1	162	39	0.895229	1
39	chr12	39716543	39716543	C	T	exonic	KIF21A		nonsynonymous SNV	KIF21A:NM_001173463:exon25:c.G3538A:	NOVEL		149	0	0	1	104	18	0.680735	1
39	chr12	44917076	44917076	G	A	exonic	NELL2		stopgain	NELL2:NM_001145108:exon17:c.C1996T:p	NOVEL		129	0	0	1	108	20	0.720917	1
39	chr12	80328709	80328709	C	A	exonic	PPP1R12A		startloss	PPP1R12A:NM_001244992:exon1:c.G3T:p.	NOVEL		155	0	0	1	88	25	1.02077	1
39	chr12	120173057	120173057	C	T	exonic	CIT		nonsynonymous SNV	CIT:NM_007174:exon24:c.G2938A:p.A980	NOVEL		138	0	0	1	78	51	0.912044	2
39	chr13	77672548	77672548	G	A	exonic	MYCBP2		nonsynonymous SNV	MYCBP2:NM_015057:exon56:c.C8741T:p.S	COSMIC	ID=COSM381406	153	0	0	1	109	35	0.878372	1
39	chr13	113964122	113964122	G	A	exonic	LAMP1		nonsynonymous SNV	LAMP1:NM_005561:exon3:c.G348A:p.M11	NOVEL		48	0	0	1	19	9	1.1616	1
39	chr14	24004259	24004259	C	A	exonic	ZFHX2		synonymous SNV	ZFHX2:NM_033400:exon2:c.G276T:p.V92V	NOVEL		220	0	0	1	97	37	0.99786	1
39	chr14	53194274	53194274	C	G	exonic	PSMC6		nonsynonymous SNV	PSMC6:NM_001366414:exon14:c.C806G:p	NOVEL		15	0	0	1	11	4	0.963699	1
39	chr15	49308774	49308774	C	G	exonic	SECISBP2L		nonsynonymous SNV	SECISBP2L:NM_014701:exon10:c.G1409C:	NOVEL		283	0	0	1	176	57	0.88408	1
39	chr15	56155274	56155274	C	T	exonic	NEDD4		nonsynonymous SNV	NEDD4:NM_198400:exon2:c.G1552A:p.G5	NOVEL		57	0	0	1	40	11	0.779463	1
39	chr16	426559	426559	C	T	exonic	TMEM8A		nonsynonymous SNV	TMEM8A:NM_021259:exon5:c.G889A:p.V	NOVEL		71	0	0	1	44	11	0.722774	1
39	chr16	2293080	2293080	G	A	exonic	ECI1		synonymous SNV	ECI1:NM_001178029:exon6:c.C658T:p.L22	DBSNP		46	0	0	1	24	8	0.903468	1
39	chr16	19063121	19063121	G	C	exonic	TMC7		synonymous SNV	TMC7:NM_001324268:exon12:c.G828C:p.L	NOVEL		354	0	0	1	173	60	0.930611	1
39	chr16	74505164	74505164	T	A	exonic	GLG1		synonymous SNV	GLG1:NM_001145666:exon14:c.A2103T:p.	NOVEL		213	0	0	1	93	40	1.08688	1
39	chr16	89804468	89804468	C	T	exonic	ZNF276		synonymous SNV	ZNF276:NM_001113525:exon11:c.C1659T	NOVEL		612	0	0	1	290	101	0.933507	1
39	chr17	1531149	1531149	G	C	exonic	SLC43A2		nonsynonymous SNV	SLC43A2:NM_001284498:exon2:c.C20G:p.	NOVEL		139	0	0	1	53	18	1.16971	1
39	chr17	1611162	1611162	G	T	exonic	TLCD2		nonsynonymous SNV	TLCD2:NM_001164407:exon4:c.C697A:p.P	NOVEL		266	0	0	1	114	35	1.0838	1
39	chr17	7129273	7129273	C	A	exonic	DVL2		nonsynonymous SNV	DVL2:NM_004422:exon15:c.G2122T:p.G70	NOVEL		131	0	0	1	67	19	1.01934	1
39	chr17	18157427	18157427	C	G	exonic	FLII		synonymous SNV	FLII:NM_001256265:exon6:c.G483C:p.L16	NOVEL		115	0	0	1	51	11	0.81859	1
39	chr17	26708563	26708563	G	A	exonic	SARM1		nonsynonymous SNV	SARM1:NM_015077:exon6:c.G809A:p.C27	NOVEL		163	0	0	1	71	15	0.804745	1
39	chr17	48069062	48069062	C	A	exonic	DLX3		nonsynonymous SNV	DLX3:NM_005220:exon3:c.G683T:p.R228L	NOVEL		86	0	0	1	53	14	0.964093	1
39	chr17	57814824	57814824	C	T	exonic	VMP1		nonsynonymous SNV	VMP1:NM_001329394:exon4:c.C223T:p.R	DBSNP+COSMIC	ID=COSM572428	285	0	0	1	158	30	0.736256	1
39	chr17	58740683	58740683	C	T	exonic	PPM1D		nonsynonymous SNV	PPM1D:NM_003620:exon6:c.C1588T:p.P5	COSMIC	ID=COSM593773	772	0	0	1	387	94	0.901671	1
39	chr17	80399713	80399713	C	G	exonic	HEXD		nonsynonymous SNV	HEXD:NM_001369488:exon11:c.C925G:p.R	NOVEL		339	0	0	1	189	53	1.01048	1
39	chr18	29847945	29847945	C	T	exonic	GAREM1		synonymous SNV	GAREM1:NM_001242409:exon6:c.G2520A	NOVEL		241	0	0	1	156	36	0.865101	1
39	chr18	65181268	65181268	C	G	exonic	DSEL		nonsynonymous SNV	DSEL:NM_032160:exon2:c.G578C:p.R193T	NOVEL		249	0	0	1	129	90	0.948056	2
39	chr19	5456701	5456701	G	A	exonic	ZNRF4		nonsynonymous SNV	ZNRF4:NM_181710:exon1:c.G1199A:p.R4	DBSNP		371	0	0	1	252	48	1.21822	1
39	chr19	11485493	11485493	C	G	exonic	SWSAP1		nonsynonymous SNV	SWSAP1:NM_175871:exon1:c.C74G:p.A25	NOVEL		151	0	0	1	63	20	1.11178	1
39	chr19	13941214	13941214	G	A	exonic	ZSWIM4		nonsynonymous SNV	ZSWIM4:NM_023072:exon13:c.G2320A:p.	NOVEL		358	0	0	1	128	52	NA	1
39	chr19	14675659	14675659	A	C	exonic	TECR		nonsynonymous SNV	TECR:NM_138501:exon8:c.A551C:p.Y184S	NOVEL		423	0	0	1	292	37	0.856271	1

39	chr19	14826264	14826264	G	A	exonic	ZNF333		synonymous SNV	ZNF333:NM_001300912:exon8:c.G582A:p	NOVEL		112	0	0	1	126	13	0.712089	1
39	chr19	39860426	39860426	G	A	exonic	SAMD4B		nonsynonymous SNV	SAMD4B:NM_001303614:exon3:c.G328A:p	NOVEL		379	0	0	1	148	69	1.14911	1
39	chr19	45974062	45974062	G	A	exonic	FOSB		nonsynonymous SNV	FOSB:NM_001114171:exon2:c.G302A:p.G	NOVEL		312	0	0	1	107	44	1.05305	1
39	chr19	49469857	49469857	G	A	exonic	FTL		synonymous SNV	FTL:NM_000146:exon4:c.G393A:p.E131E	NOVEL		56	0	0	1	29	11	0.993815	1
39	chr2	73680387	73680387	G	A	exonic	ALMS1		nonsynonymous SNV	ALMS1:NM_015120:exon8:c.G6730A:p.G2	NOVEL		232	0	0	1	118	40	0.914904	1
39	chr2	128918748	128918748	C	G	exonic	UGGT1		nonsynonymous SNV	UGGT1:NM_020120:exon25:c.C2731G:p.Q	NOVEL		77	0	0	1	45	12	0.760815	1
39	chr2	165353954	165353954	T	A	exonic	GRB14		nonsynonymous SNV	GRB14:NM_001303422:exon9:c.A890T:p.C	NOVEL		165	0	0	1	96	12	0.401541	1
39	chr2	210808227	210808227	C	A	exonic	UNC80		nonsynonymous SNV	UNC80:NM_032504:exon44:c.C6841A:p.L2	NOVEL		233	0	0	1	103	29	0.793957	1
39	chr2	219209588	219209588	C	A	exonic	PNKD		nonsynonymous SNV	PNKD:NM_022572:exon9:c.C970A:p.L324M	NOVEL		330	0	0	1	183	53	0.81159	1
39	chr2	220349659	220349659	C	T	exonic	SPEG		nonsynonymous SNV	SPEG:NM_005876:exon30:c.C7474T:p.R24	DBSNP		27	0	0	1	11	6	1.27548	1
39	chr20	4202730	4202730	T	A	exonic	ADRA1D		nonsynonymous SNV	ADRA1D:NM_000678:exon2:c.A1159T:p.I3	NOVEL		191	0	0	1	116	37	1.11577	1
39	chr20	25457440	25457440	C	T	exonic	NINL		synonymous SNV	NINL:NM_025176:exon17:c.G2487A:p.L82	NOVEL		277	0	0	1	164	52	1.11075	1
39	chr20	44052876	44052876	G	A	exonic	PIGT		nonsynonymous SNV	PIGT:NM_001184730:exon8:c.G949A:p.A3	NOVEL		62	0	0	1	37	17	1.45252	1
39	chr20	55027183	55027183	C	T	exonic	CASS4		synonymous SNV	CASS4:NM_001164114:exon5:c.C789T:p.P	DBSNP		222	0	0	1	134	31	0.866849	1
39	chr21	35260467	35260467	C	T	exonic	ITSN1		nonsynonymous SNV	ITSN1:NM_001331010:exon39:c.C5014T:p	NOVEL		129	0	0	1	61	24	1.02039	1
39	chr22	19163732	19163732	G	A	exonic	SLC25A1		synonymous SNV	SLC25A1:NM_001256534:exon8:c.C868T:p	DBSNP		213	0	0	1	74	36	1.18272	1
39	chr22	40283639	40283639	T	A	exonic	ENTHD1		nonsynonymous SNV	ENTHD1:NM_152512:exon2:c.A114T:p.L38	NOVEL		175	0	0	1	111	28	0.727974	1
39	chr3	4683907	4683907	T	A	exonic	ITPR1		nonsynonymous SNV	ITPR1:NM_001099952:exon7:c.T497A:p.F1	NOVEL		187	0	0	1	89	8	0.380526	1
39	chr3	4683908	4683908	C	A	exonic	ITPR1		nonsynonymous SNV	ITPR1:NM_001099952:exon7:c.C498A:p.F1	NOVEL		186	0	0	1	89	8	0.380526	1
39	chr3	14535241	14535241	T	G	exonic	GRIP2		unknown	UNKNOWN	NOVEL		86	0	0	1	43	14	1.13323	1
39	chr3	128751670	128751670	G	C	exonic	EFCC1		nonsynonymous SNV	EFCC1:NM_024768:exon4:c.G1144C:p.E38	NOVEL		85	0	0	1	27	8	1.0546	1
39	chr3	185339627	185339627	G	T	exonic	SENP2		nonsynonymous SNV	SENP2:NM_021627:exon14:c.G1460T:p.R4	NOVEL		228	0	0	1	100	23	0.862757	1
39	chr3	194118430	194118430	A	G	exonic	GP5		synonymous SNV	GP5:NM_004488:exon2:c.T582C:p.A194A	NOVEL		259	0	0	1	129	21	0.645942	1
39	chr4	89912214	89912214	C	T	exonic	FAM13A		nonsynonymous SNV	FAM13A:NM_014883:exon4:c.G515A:p.C1	NOVEL		203	0	0	1	95	30	0.867329	1
39	chr4	113461266	113461266	G	A	exonic	ZGRF1		synonymous SNV	ZGRF1:NM_001350397:exon26:c.C5751T:p	DBSNP		100	0	0	1	62	19	0.847698	1
39	chr5	64920047	64920047	C	T	exonic	TRIM23		nonsynonymous SNV	TRIM23:NM_001656:exon1:c.G55A:p.G19	NOVEL		571	0	0	1	292	62	0.808079	1
39	chr5	74442861	74442861	C	T	exonic	ANKRD31		nonsynonymous SNV	ANKRD31:NM_001164443:exon14:c.G237	NOVEL		405	0	0	1	217	50	0.864021	1
39	chr5	139818138	139818138	G	A	exonic	ANKHD1;ANKHD1-		nonsynonymous SNV	ANKHD1:NM_001197030:exon3:c.G553A:p	NOVEL		331	0	0	1	141	30	0.634013	1
39	chr5	141051685	141051685	G	A	exonic	ARAP3		synonymous SNV	ARAP3:NM_022481:exon10:c.C1569T:p.I52	NOVEL		795	0	0	1	255	87	0.919318	1
39	chr6	116325077	116325077	T	C	exonic	FRK		synonymous SNV	FRK:NM_002031:exon2:c.A429G:p.R143R	NOVEL		171	0	0	1	159	53	0.903468	1
39	chr6	137018378	137018378	T	G	exonic	MAP3K5		nonsynonymous SNV	MAP3K5:NM_005923:exon5:c.A954C:p.L31	NOVEL		171	0	0	1	173	53	0.847501	1
39	chr6	137019722	137019722	G	A	exonic	MAP3K5		synonymous SNV	MAP3K5:NM_005923:exon4:c.C711T:p.L23	NOVEL		194	0	0	1	139	52	0.983881	1
39	chr6	142399743	142399743	A	G	exonic	NMBR		synonymous SNV	NMBR:NM_001324308:exon2:c.T276C:p.I9	NOVEL		74	0	0	1	39	13	0.903468	1
39	chr7	1588264	1588264	G	C	exonic	TMEM184A		synonymous SNV	TMEM184A:NM_001097620:exon7:c.C705	DBSNP		188	0	0	1	85	41	1.17594	1
39	chr7	21750203	21750203	C	T	exonic	DNAH11		nonsynonymous SNV	DNAH11:NM_001277115:exon41:c.C6716T	NOVEL		81	0	0	1	45	21	1.14987	1
39	chr7	75915159	75915159	G	A	exonic	SRRM3		unknown	UNKNOWN	NOVEL		122	0	0	1	60	26	1.09257	1
39	chr7	100663484	100663484	C	T	exonic	MUC17		nonsynonymous SNV	MUC17:NM_001040105:exon1:c.C68T:p.A	NOVEL		164	0	0	1	72	35	1.18211	1
39	chr7	100850946	100850946	C	T	exonic	PLOD3		synonymous SNV	PLOD3:NM_001084:exon17:c.G1848A:p.Q	NOVEL		134	0	0	1	74	26	0.939607	1
39	chr8	19797017	19797017	C	T	exonic	LPL		synonymous SNV	LPL:NM_000237:exon1:c.C66T:p.R22R	NOVEL		192	0	0	1	109	29	0.759437	1
39	chr8	37688351	37688351	G	A	exonic	ADGRA2		stopgain	ADGRA2:NM_032777:exon7:c.G842A:p.W	DBSNP		152	0	0	1	70	34	1.18146	1
39	chr8	143559637	143559637	G	A	exonic	ADGRB1		nonsynonymous SNV	ADGRB1:NM_001702:exon7:c.G1477A:p.E	NOVEL		140	0	0	1	53	33	1.38672	1
39	chr9	34220937	34220937	G	A	exonic	UBAP1		nonsynonymous SNV	UBAP1:NM_016525:exon2:c.G25A:p.D9N,	NOVEL		68	0	0	1	45	12	0.760815	1
39	chr9	34459036	34459036	A	G	exonic	DNAI1		synonymous SNV	DNAI1:NM_001281428:exon1:c.A33G:p.K1	NOVEL		177	0	0	1	95	39	1.0518	1
39	chr9	35811197	35811197	G	C	exonic	SPAG8		synonymous SNV	SPAG8:NM_001039592:exon2:c.C846G:p.L	NOVEL		126	0	0	1	69	20	0.812106	1
39	chr9	139370012	139370012	C	G	exonic	SEC16A		nonsynonymous SNV	SEC16A:NM_001276418:exon2:c.G2056C:p	NOVEL		106	0	0	1	54	30	1.29067	1
39	chr22	29674207	29674207	T	G	splicing	EWSR1	NM_013986:exon6:c.4			NOVEL		120	7	0.14302	1	75	0	0	1
39	chr16	2053123	2053123	T	G	splicing	ZNF598	NM_178167:exon5:c.3			NOVEL		57	0	0	1	30	13	1.09257	1
