# Supplementary material for: Clonal evolution after treatment pressure in multiple myeloma: heterogenous genomic aberrations and transcriptomic convergence
Source: Leukemia. 2022 May 28;36(7):1887–97. doi: 10.1038/s41375-022-01597-y (PMC9252918; doi:10.1038/s41375-022-01597-y)
Supplement: Supplementary file 13 — Table S7 [file 41375_2022_1597_MOESM13_ESM.pdf]

Table S7. Genes differentially expressed between latest available progression sample and earliest(first) sample. For included samples, see Table S1. Only genes with Fold Change >1.5 and adj p-value<0.05 are shown.

GENE_ID	baseMean	log2FoldChange	FoldChange	pvalue	padj	symbol
ENSG00000171483	97.1925769	3.343663283	10.1517974	5.46E-17	1.21E-12	NA
ENSG00000117148	357.658085	4.754264983	26.9883521	1.05E-11	7.75E-08	ACTL8
ENSG00000007968	1762.4316	1.242177191	2.36555252	9.69E-12	7.75E-08	E2F2
ENSG00000198681	565.592898	2.576799079	5.96614516	2.33E-11	1.03E-07	MAGEA1
ENSG00000148773	2033.21545	1.27534615	2.42056888	2.23E-11	1.03E-07	MKI67
ENSG00000214107	69.3014056	3.952783726	15.4848309	4.18E-11	1.31E-07	MAGEB1
ENSG00000213401	178.926731	3.303998699	9.87649195	5.33E-11	1.31E-07	MAGEA12
ENSG00000221867	779.158231	2.223750643	4.67106216	4.09E-11	1.31E-07	MAGEA3
ENSG00000089685	576.882981	1.271541581	2.41419395	5.01E-11	1.31E-07	BIRC5
ENSG00000134802	218.481061	1.644824314	3.12709774	6.89E-11	1.53E-07	SLC43A3
ENSG00000185527	33.1993764	1.683535653	3.21214196	8.48E-11	1.57E-07	PDE6G
ENSG00000205730	190.178705	1.679248616	3.20261109	8.37E-11	1.57E-07	ITPRIPL2
ENSG00000085840	239.118001	1.066401464	2.09420324	1.18E-10	1.86E-07	ORC1
ENSG00000073111	1631.86649	1.025957546	2.03631048	1.91E-10	2.82E-07	MCM2
ENSG00000088325	832.054362	1.133003045	2.19314781	2.10E-10	2.91E-07	TPX2
ENSG00000065833	295.878294	1.292262508	2.44911838	2.75E-10	3.58E-07	ME1
ENSG00000097021	302.218537	0.834118396	1.78276729	3.44E-10	4.23E-07	ACOT7
ENSG00000068489	322.380707	1.090089622	2.12887261	3.72E-10	4.33E-07	PRR11
ENSG00000145386	377.044168	1.162194606	2.23797606	4.14E-10	4.59E-07	CCNA2
ENSG00000265415	28.4559871	1.46983519	2.76990249	5.30E-10	5.21E-07	NA
ENSG00000171848	2580.45911	1.212870429	2.31798371	5.41E-10	5.21E-07	RRM2
ENSG00000146670	645.003815	1.094859613	2.13592296	5.92E-10	5.46E-07	CDCA5
ENSG00000148200	147.69037	1.213783424	2.31945109	6.97E-10	6.17E-07	NR6A1
ENSG00000138031	966.037262	0.856070443	1.81010131	9.11E-10	7.76E-07	ADCY3
ENSG00000137807	440.991699	1.080130968	2.114228	9.49E-10	7.78E-07	KIF23
ENSG00000133019	282.849043	1.799920594	3.4820106	1.35E-09	1.06E-06	CHRM3
ENSG00000166851	415.78699	1.150072358	2.21925025	1.55E-09	1.15E-06	PLK1
ENSG00000103522	172.457191	1.911383065	3.76169548	1.86E-09	1.29E-06	IL21R
ENSG00000013810	849.7723	0.857707144	1.81215599	1.80E-09	1.29E-06	TACC3
ENSG00000073008	139.214568	1.390567421	2.62181778	1.98E-09	1.33E-06	PVR
ENSG00000072110	310.810928	1.426223127	2.68742246	2.26E-09	1.47E-06	ACTN1
ENSG00000099399	225.524033	2.871045781	7.31595287	2.67E-09	1.64E-06	MAGEB2
ENSG00000176890	1549.96862	1.071861288	2.10214369	2.60E-09	1.64E-06	TYMS
ENSG00000232775	28.8896412	1.7846795	3.44541914	2.89E-09	1.73E-06	BMS1P22
ENSG00000186185	277.580201	1.251349004	2.38063923	3.14E-09	1.83E-06	KIF18B
ENSG00000251026	60.6608154	1.579844622	2.98937652	3.53E-09	1.95E-06	NA
ENSG00000154839	321.844745	1.121577959	2.17584827	3.90E-09	2.11E-06	SKA1
ENSG00000164687	644.844098	0.888567647	1.85133714	4.64E-09	2.45E-06	FABP5
ENSG00000162063	255.746049	1.025282892	2.03535845	6.36E-09	3.20E-06	CCNF
ENSG00000126890	558.162172	2.418513437	5.34619862	7.91E-09	3.81E-06	CTAG2
ENSG00000175063	254.59093	1.132177773	2.19189361	8.73E-09	3.97E-06	UBE2C
ENSG00000140859	225.512051	1.119574426	2.17282868	8.71E-09	3.97E-06	KIFC3
ENSG00000117399	382.961236	1.100945147	2.14495168	8.78E-09	3.97E-06	CDC20
ENSG00000165124	39.5580851	1.364880861	2.57555056	9.52E-09	4.21E-06	SVEP1
ENSG00000111206	726.619838	1.007927473	2.01102006	1.05E-08	4.54E-06	FOXM1
ENSG00000150471	203.141739	2.301776421	4.93064512	1.15E-08	4.91E-06	ADGRL3
ENSG00000129173	204.330343	1.205918737	2.30684127	1.18E-08	4.93E-06	E2F8
ENSG00000149635	284.474639	2.321611079	4.99890143	1.32E-08	5.42E-06	OCSTAMP
ENSG00000127564	410.041915	1.198369065	2.29480102	1.35E-08	5.44E-06	PKMYT1
ENSG00000233515	48.6900981	1.561952549	2.9525317	1.90E-08	7.49E-06	LINC01518
ENSG00000077238	420.471739	1.350745909	2.55043955	1.93E-08	7.50E-06	IL4R
ENSG00000162073	315.607315	0.987588987	1.98286848	1.98E-08	7.57E-06	PAQR4
ENSG00000029153	213.954646	1.36307971	2.57233709	2.35E-08	8.62E-06	ARNTL2
ENSG00000204410	883.646322	0.630230524	1.54781229	2.37E-08	8.62E-06	MSH5
ENSG00000155659	122.69138	1.724408595	3.3044464	3.35E-08	1.20E-05	VSIG4
ENSG00000187741	759.384526	0.74484828	1.67579803	3.50E-08	1.23E-05	FANCA
ENSG00000225210	1016.61161	0.932131806	1.90809342	3.86E-08	1.33E-05	DUXAP9
ENSG00000170927	118.592091	1.553372103	2.9350236	4.29E-08	1.42E-05	PKHD1
ENSG00000126215	479.188383	0.611804637	1.52816957	4.22E-08	1.42E-05	XRCC3
ENSG00000167513	375.298242	1.053520323	2.07558832	4.45E-08	1.45E-05	CDT1
ENSG00000152969	22.048389	1.937159919	3.8295103	4.58E-08	1.47E-05	JAKMIP1
ENSG00000206195	582.161947	1.080359539	2.11456299	4.84E-08	1.51E-05	DUXAP8
ENSG00000126752	397.699584	2.119874078	4.34656005	5.02E-08	1.52E-05	SSX1
ENSG00000080986	463.185402	0.935500117	1.91255352	4.96E-08	1.52E-05	NDC80
ENSG00000075218	237.103071	0.966060385	1.95349882	5.57E-08	1.65E-05	GTSE1
ENSG00000166049	104.161205	4.28708118	19.5227067	5.84E-08	1.69E-05	PASD1
ENSG00000104738	1688.11386	0.803392083	1.74519964	5.91E-08	1.69E-05	MCM4
ENSG00000090889	292.6937	1.175155088	2.25817155	6.47E-08	1.79E-05	KIF4A
ENSG00000204379	249.409481	1.775766573	3.42419906	6.71E-08	1.81E-05	XAGE1A
ENSG00000189159	893.776165	0.710796794	1.63670781	6.67E-08	1.81E-05	JPT1
ENSG00000130675	21.8976847	1.678823443	3.2016674	7.16E-08	1.91E-05	MNX1
ENSG00000099194	1183.37119	1.126454832	2.18321594	8.78E-08	2.26E-05	SCD
ENSG00000137766	168.818296	1.969691459	3.91684343	9.20E-08	2.34E-05	UNC13C
ENSG00000005189	140.315633	0.924188156	1.8976161	9.48E-08	2.38E-05	REXO5
ENSG00000137804	1341.1723	0.88438294	1.8459749	9.95E-08	2.47E-05	NUSAP1
ENSG00000092853	264.555399	1.0317639	2.04452244	1.01E-07	2.49E-05	CLSPN
ENSG00000117632	3239.79683	0.867332057	1.82428617	1.04E-07	2.54E-05	STMN1
ENSG00000182583	35.8499456	1.894231017	3.71723789	1.07E-07	2.54E-05	VCX
ENSG00000142945	294.163389	1.055948934	2.07908528	1.06E-07	2.54E-05	KIF2C
ENSG00000135476	433.595059	0.983798444	1.97766552	1.12E-07	2.64E-05	ESPL1
ENSG00000134690	347.076391	1.002569592	2.00356538	1.24E-07	2.78E-05	CDCA8
ENSG00000198033	95.9595235	4.291354183	19.5806151	1.30E-07	2.82E-05	TUBA3C
ENSG00000185480	226.393418	0.695737811	1.61971256	1.30E-07	2.82E-05	PARPBP
ENSG00000197172	685.188597	1.727450244	3.31142055	1.49E-07	3.20E-05	MAGEA6
ENSG00000149503	705.532737	0.671178529	1.59237324	1.51E-07	3.21E-05	INCENP
ENSG00000164045	381.533199	0.790483874	1.72965448	1.61E-07	3.39E-05	CDC25A
ENSG00000118655	139.268393	0.72947055	1.6580305	1.63E-07	3.40E-05	DCLRE1B
ENSG00000038945	218.911418	1.204891651	2.30519956	1.69E-07	3.47E-05	MSR1
ENSG00000143228	397.959357	1.082876906	2.11825593	1.88E-07	3.78E-05	NUF2
ENSG00000137310	946.904654	0.820272546	1.76573953	2.01E-07	4.00E-05	TCF19
ENSG00000225518	17.4860336	1.108468602	2.15616652	2.22E-07	4.24E-05	NA
ENSG00000244306	677.924829	0.870804662	1.82868256	2.21E-07	4.24E-05	DUXAP10
ENSG00000108175	1989.92744	0.959856216	1.94511603	2.34E-07	4.39E-05	ZMIZ1
ENSG00000121152	284.336475	0.961125397	1.94682796	2.50E-07	4.64E-05	NCAPH
ENSG00000075702	526.276897	0.804632241	1.74670048	2.53E-07	4.68E-05	WDR62
ENSG00000269404	100.251732	1.663665142	3.1682038	2.60E-07	4.76E-05	SPIB
ENSG00000173638	591.336938	0.736710202	1.66637165	2.71E-07	4.88E-05	SLC19A1
ENSG00000146918	1149.86158	0.781041894	1.71837141	2.75E-07	4.91E-05	NCAPG2
ENSG00000130881	41.7600118	1.549906319	2.92798126	3.06E-07	5.42E-05	LRP3
ENSG00000137558	111.294201	1.893510235	3.71538119	3.30E-07	5.75E-05	PI15
ENSG00000118513	283.124976	1.203423835	2.30285542	3.55E-07	6.08E-05	MYB
ENSG00000101057	2052.91327	0.876820125	1.83632336	3.56E-07	6.08E-05	MYBL2
ENSG00000196230	13329.4711	0.596580592	1.51212834	3.71E-07	6.23E-05	TUBB
ENSG00000186567	37.7024152	1.1578396	2.23123056	3.92E-07	6.44E-05	CEACAM19
ENSG00000173083	293.444646	1.210633284	2.31439207	4.32E-07	6.90E-05	HPSE
ENSG00000051180	196.748728	0.914008775	1.88427401	4.31E-07	6.90E-05	RAD51
ENSG00000160949	778.208254	0.686142748	1.60897593	4.33E-07	6.90E-05	TONSL
ENSG00000179750	548.648272	0.842236158	1.79282685	4.37E-07	6.91E-05	APOBEC3B
ENSG00000170627	459.949399	1.301002614	2.46400061	4.58E-07	7.20E-05	GTSF1
ENSG00000136982	130.594047	1.135315502	2.19666596	4.66E-07	7.27E-05	DSCC1
ENSG00000272872	82.2248971	1.335365215	2.52339353	5.03E-07	7.48E-05	NA
ENSG00000137486	65.8341554	1.288485217	2.44271444	5.04E-07	7.48E-05	ARRB1
ENSG00000175489	103.020759	1.242493694	2.36607154	5.14E-07	7.48E-05	LRRC25
ENSG00000126787	334.970611	1.090763417	2.12986711	5.12E-07	7.48E-05	DLGAP5
ENSG00000101447	103.631468	1.032173458	2.04510293	5.07E-07	7.48E-05	FAM83D
ENSG00000147443	211.505852	0.971301849	1.960609	5.09E-07	7.48E-05	DOK2
ENSG00000181019	67.9305881	0.941774661	1.92088967	4.95E-07	7.48E-05	NQO1
ENSG00000122952	590.287168	0.916128236	1.88704423	5.17E-07	7.48E-05	ZWINT
ENSG00000152910	61.7736119	3.199926122	9.18911627	5.38E-07	7.74E-05	CNTNAP4
ENSG00000178999	260.31845	0.973264125	1.96327753	5.51E-07	7.87E-05	AURKB
ENSG00000136167	5383.19283	1.099664107	2.14304792	5.62E-07	7.98E-05	LCP1
ENSG00000019169	35.962392	1.786101989	3.44881798	5.67E-07	7.99E-05	MARCO
ENSG00000204382	256.195665	1.594876858	3.02068733	5.72E-07	8.01E-05	XAGE1B
ENSG00000166415	179.961691	1.776932208	3.42696678	5.83E-07	8.12E-05	WDR72
ENSG00000205777	763.107471	2.150508538	4.43984262	5.93E-07	8.16E-05	GAGE1

ENSG00000140525	1613.9344	0.6857316	1.60851746	6.39E-07	8.67E-05	FANCI
ENSG00000071967	173.505792	0.942892135	1.92237812	7.14E-07	9.46E-05	CYBRD1
ENSG00000167900	616.150367	0.961842873	1.94779639	7.29E-07	9.50E-05	TK1
ENSG00000159259	289.624511	0.661517408	1.58174541	7.27E-07	9.50E-05	CHAF1B
ENSG00000135451	310.922612	0.934439407	1.91114787	7.43E-07	9.63E-05	TROAP
ENSG00000149742	63.1876535	2.293491161	4.90241005	7.70E-07	9.82E-05	SLC22A9
ENSG00000175832	107.182138	1.366260848	2.57801534	7.76E-07	9.82E-05	ETV4
ENSG00000170312	403.21745	0.963006348	1.94936784	7.72E-07	9.82E-05	CDK1
ENSG00000122966	369.830658	1.022356695	2.03123435	8.40E-07	0.00010564	CIT
ENSG00000009694	235.232318	1.543139947	2.91428091	9.27E-07	0.00011534	TENM1
ENSG00000138092	349.067177	0.682129042	1.60450584	9.41E-07	0.00011636	CENPO
ENSG00000101412	910.370754	0.914805113	1.88531438	9.82E-07	0.00012019	E2F1
ENSG00000100350	560.418266	0.750019466	1.68181552	1.03E-06	0.00012414	FOXRED2
ENSG00000166111	284.278879	1.094798782	2.1358329	1.10E-06	0.00012984	SVOP
ENSG00000122254	39.7083971	1.652560536	3.14391135	1.14E-06	0.00013413	HS3ST2
ENSG00000160957	747.721124	0.846908065	1.798642	1.15E-06	0.00013521	RECQL4
ENSG00000117650	221.119487	1.035228465	2.04943817	1.16E-06	0.00013562	NEK2
ENSG00000160183	454.762725	1.730036292	3.31736163	1.22E-06	0.00014033	TMPRSS3
ENSG00000237649	529.942715	0.916085794	1.88698871	1.22E-06	0.00014033	KIFC1
ENSG00000213658	389.742871	0.611673825	1.52803101	1.21E-06	0.00014033	LAT
ENSG00000158528	291.13776	2.129457077	4.37552787	1.25E-06	0.0001408	PPP1R9A
ENSG00000110400	469.289633	0.801594381	1.74302635	1.24E-06	0.0001408	NECTIN1
ENSG00000100297	2175.21642	0.606696551	1.52276841	1.25E-06	0.0001408	MCM5
ENSG00000162512	468.908916	0.962544882	1.94874441	1.26E-06	0.00014162	SDC3
ENSG00000176887	65.6642261	1.917964416	3.77889496	1.27E-06	0.00014162	SOX11
ENSG00000227507	136.349956	1.576578218	2.98261594	1.29E-06	0.00014364	LTB
ENSG00000163040	39.8802642	1.003461108	2.00480387	1.30E-06	0.00014364	CCDC74A
ENSG00000103811	1551.98994	1.054528184	2.07703883	1.35E-06	0.00014838	CTSH
ENSG00000054392	173.178219	1.234553965	2.35308586	1.39E-06	0.00015182	HHAT
ENSG00000216490	2747.07702	0.76629039	1.70089065	1.42E-06	0.00015402	IFI30
ENSG00000152270	312.276004	1.172458761	2.25395508	1.47E-06	0.00015859	PDE3B
ENSG00000088827	182.065332	1.427453755	2.68971582	1.51E-06	0.00016268	SIGLEC1
ENSG00000115919	269.144734	0.950731256	1.93285211	1.52E-06	0.00016268	KYNU
ENSG00000174371	317.10256	0.982991334	1.97655943	1.54E-06	0.00016438	EXO1
ENSG00000143344	311.727749	1.002611231	2.00362321	1.59E-06	0.00016849	RGL1
ENSG00000173157	73.3002985	1.769483365	3.40931846	1.65E-06	0.00017269	ADAMTS20
ENSG00000231976	170.390212	1.490336495	2.80954497	1.70E-06	0.0001755	NA
ENSG00000142731	333.7399	0.835757658	1.78479411	1.70E-06	0.0001755	PLK4
ENSG00000161888	245.557001	1.008931667	2.01242033	1.78E-06	0.00018237	SPC24
ENSG00000135144	521.777123	1.385901463	2.61335201	1.80E-06	0.0001834	DTX1
ENSG00000132646	2071.4895	0.646985659	1.56589303	1.81E-06	0.00018369	PCNA
ENSG00000171241	753.257497	0.90221392	1.86893179	1.96E-06	0.00019617	SHCBP1
ENSG00000074416	765.616772	0.893074443	1.85712953	1.98E-06	0.00019678	MGLL
ENSG00000157456	439.530366	0.88749661	1.84996325	2.03E-06	0.00019878	CCNB2
ENSG00000007038	57.3406008	2.304013445	4.93829645	2.12E-06	0.00020628	PRSS21
ENSG00000267508	56.3029651	1.297689053	2.45834782	2.16E-06	0.00020814	ZNF285
ENSG00000046774	333.274969	1.180170624	2.26603575	2.16E-06	0.00020814	MAGEC2
ENSG00000138160	755.090104	0.800649644	1.74188532	2.31E-06	0.00021833	KIF11
ENSG00000198185	67.6687524	1.507571828	2.84331084	2.39E-06	0.00022336	ZNF334
ENSG00000147536	222.490361	0.884439326	1.84604705	2.40E-06	0.00022349	GINS4
ENSG00000127586	833.189081	0.689747115	1.61300076	2.48E-06	0.00023001	CHTF18
ENSG00000198930	90.0488595	1.909450061	3.75665873	2.52E-06	0.00023241	CSAG1
ENSG00000170049	42.3134215	0.810855026	1.75425081	2.53E-06	0.0002327	KCNAB3
ENSG00000144045	85.8631177	1.236076027	2.35556971	2.56E-06	0.00023433	DQX1
ENSG00000104147	133.053415	0.968780051	1.95718489	2.71E-06	0.00024214	OIP5
ENSG00000108797	1389.02491	0.757783697	1.69089104	2.71E-06	0.00024214	CNTNAP1
ENSG00000183763	150.766044	0.673391123	1.59481726	2.72E-06	0.00024214	TRAIP
ENSG00000162769	389.541679	0.586074109	1.50115621	2.75E-06	0.00024336	FLVCR1
ENSG00000123485	362.275649	0.909320981	1.87816132	3.06E-06	0.00026608	HJURP
ENSG00000236362	219.327752	2.490836729	5.62103863	3.08E-06	0.00026647	GAGE2C
ENSG00000215269	219.322996	2.4889813	5.61381414	3.11E-06	0.00026724	GAGE4
ENSG00000183844	279.760844	1.767932256	3.40565491	3.13E-06	0.00026742	FAM3B
ENSG00000259863	111.595799	1.927332883	3.80351391	3.23E-06	0.00027502	NA
ENSG00000267284	22.8226228	1.321578263	2.49939386	3.25E-06	0.00027539	NA
ENSG00000157193	857.97143	0.611117379	1.52744177	3.51E-06	0.0002929	LRP8
ENSG00000152253	133.115593	1.17498592	2.25790677	3.60E-06	0.00029713	SPC25
ENSG00000261008	16.4818413	1.224151504	2.33618011	3.65E-06	0.00029902	LINC01572
ENSG00000253301	37.895292	1.609664762	3.05180919	3.66E-06	0.00029937	LINC01606
ENSG00000065328	219.521374	1.028373141	2.03972286	3.70E-06	0.00030158	MCM10
ENSG00000204832	119.869911	1.482533668	2.79439055	3.79E-06	0.00030517	ST8SIA6-AS1
ENSG00000164236	652.621047	1.450329489	2.73270455	3.80E-06	0.00030517	ANKRD33B
ENSG00000011677	137.718689	1.43147348	2.69722052	3.77E-06	0.00030517	GABRA3
ENSG00000115163	117.094718	1.107595302	2.15486173	3.80E-06	0.00030517	CENPA
ENSG00000109805	526.671172	0.853777269	1.80722642	3.91E-06	0.00031215	NCAPG
ENSG00000047597	991.342625	0.650435718	1.56964218	3.92E-06	0.00031215	XK
ENSG00000198554	243.50003	0.970841372	1.95998331	4.02E-06	0.00031829	WDHD1
ENSG00000131747	1365.12098	1.050797793	2.07167514	4.10E-06	0.00032335	TOP2A
ENSG00000137812	664.098874	0.793226693	1.73294599	4.16E-06	0.00032678	KNL1
ENSG00000084453	149.580912	1.696446656	3.24101715	4.25E-06	0.00033219	SLCO1A2
ENSG00000112984	209.368218	1.040711483	2.05724196	4.29E-06	0.00033464	KIF20A
ENSG00000000938	436.321818	1.338096153	2.52817469	4.35E-06	0.0003377	FGR
ENSG00000168843	295.693562	1.473713894	2.77735943	4.54E-06	0.00035001	FSTL5
ENSG00000258592	8.9336613	1.715701703	3.28456361	4.71E-06	0.00035847	NA
ENSG00000092068	91.6872603	1.438645026	2.71066162	4.71E-06	0.00035847	SLC7A8
ENSG00000167703	979.890862	0.675526813	1.59717989	5.28E-06	0.00039382	SLC43A2
ENSG00000134780	20.9676439	1.602472281	3.03663242	5.38E-06	0.00039828	DAGLA
ENSG00000077585	80.1424652	1.183809882	2.27175914	5.41E-06	0.00039857	GPR137B
ENSG00000183856	275.034092	0.934369677	1.9110555	5.55E-06	0.00040725	IQGAP3
ENSG00000179455	90.8860692	1.572142586	2.97345983	5.99E-06	0.00043654	MKRN3
ENSG00000133048	72.4472757	1.49824066	2.82498001	6.06E-06	0.00043888	CHI3L1
ENSG00000203760	161.814289	0.70005576	1.62456758	6.23E-06	0.00044819	CENPW
ENSG00000162062	158.332835	0.985466361	1.97995324	6.33E-06	0.00045139	TEDC2
ENSG00000106789	100.456701	0.903406143	1.87047689	6.34E-06	0.00045139	CORO2A
ENSG00000106536	48.8360893	1.708760535	3.2687987	6.39E-06	0.00045311	POU6F2
ENSG00000180644	110.502065	1.399776806	2.63860758	6.40E-06	0.00045311	PRF1
ENSG00000251003	39.0669059	1.974784323	3.93069473	6.46E-06	0.000454	NA
ENSG00000008517	920.365821	1.488806247	2.80656651	6.47E-06	0.000454	IL32
ENSG00000167895	2489.42586	1.13284839	2.19291272	6.49E-06	0.000454	TMC8
ENSG00000111665	305.742726	0.687581039	1.6105808	7.02E-06	0.00048155	CDCA3
ENSG00000139734	154.971863	0.862142179	1.81773536	7.06E-06	0.00048236	DIAPH3
ENSG00000138772	204.377453	1.336371507	2.52515423	7.35E-06	0.00049801	ANXA3
ENSG00000152092	150.918508	2.153017698	4.44757119	7.61E-06	0.0005086	ASTN1
ENSG00000167210	12.120513	1.504257927	2.83678719	7.60E-06	0.0005086	LOXHD1
ENSG00000125898	115.956524	0.59783136	1.51343987	7.77E-06	0.00051638	FAM110A
ENSG00000110080	182.150453	0.6997006	1.6241677	7.82E-06	0.00051835	ST3GAL4
ENSG00000179855	22.5549228	1.896291181	3.72254988	7.84E-06	0.00051839	GIPC3
ENSG00000250337	550.448393	0.980112039	1.9726186	7.93E-06	0.00052092	PURPL
ENSG00000122547	74.3480065	1.07983128	2.11378886	7.98E-06	0.00052293	EEPD1
ENSG00000088882	149.820584	1.856208133	3.62054814	8.30E-06	0.0005371	CPXM1
ENSG00000173894	76.7198002	1.353327484	2.55500742	8.37E-06	0.00053726	CBX2
ENSG00000144354	41.5021348	1.509783993	2.84767399	8.54E-06	0.00054327	CDCA7
ENSG00000224738	12.3747317	1.009185523	2.01277446	8.49E-06	0.00054327	NA
ENSG00000169750	48.8682206	0.766535331	1.70117945	9.05E-06	0.00057076	RAC3
ENSG00000160298	230.294279	0.706677803	1.63204157	9.12E-06	0.00057362	C21orf58
ENSG00000164756	51.1505246	1.967094662	3.9097996	9.27E-06	0.00058134	SLC30A8
ENSG00000184451	664.830423	0.951469322	1.93384119	1.00E-05	0.0006222	CCR10
ENSG00000178562	1615.36372	1.049215795	2.06940468	1.01E-05	0.00062496	CD28
ENSG00000105011	616.761225	0.749154092	1.68080702	1.04E-05	0.0006349	ASF1B
ENSG00000198826	958.715418	0.689341676	1.61254752	1.04E-05	0.0006349	ARHGAP11A
ENSG00000100206	71.8640976	1.188710082	2.27948842	1.08E-05	0.00065245	DMC1
ENSG00000188130	544.211842	0.922211667	1.89501815	1.09E-05	0.00065749	MAPK12
ENSG00000106462	1187.9172	0.593417407	1.50881655	1.11E-05	0.00066204	EZH2
ENSG00000183549	29.9996031	1.331293221	2.51628131	1.12E-05	0.00066803	ACSM5
ENSG00000163462	71.9228396	1.138920554	2.20216192	1.12E-05	0.00066803	TRIM46
ENSG00000077152	363.966452	0.781316093	1.71869803	1.16E-05	0.00068286	UBE2T
ENSG00000158639	491.961975	1.897315224	3.72519313	1.17E-05	0.00068606	PAGE5
ENSG00000177465	23.5593585	1.354088034	2.55635471	1.18E-05	0.00069025	ACOT4
ENSG00000169059	52.1711141	1.925820276	3.79952818	1.19E-05	0.00069037	VCX3A

ENSG00000136492	247.570197	0.754475839	1.68701855	1.19E-05	0.00069037	BRIP1
ENSG00000119403	1136.44536	0.715072644	1.64156587	1.19E-05	0.00069037	PHF19
ENSG00000253706	47.5676335	1.472004482	2.77407056	1.25E-05	0.00071845	NA
ENSG00000140968	170.222437	1.186457339	2.27593182	1.32E-05	0.00075104	IRF8
ENSG00000225548	33.5142248	1.742841832	3.34693801	1.39E-05	0.00078627	LINC01980
ENSG00000073282	43.7976202	1.298724411	2.4601127	1.42E-05	0.00079761	TP63
ENSG00000188404	2146.37526	1.163578746	2.24012424	1.42E-05	0.00079761	SELL
ENSG00000014914	192.999988	0.84297107	1.79374035	1.45E-05	0.00081142	MTMR11
ENSG00000145908	297.416145	0.992434141	1.98953895	1.49E-05	0.00083259	ZNF300
ENSG00000181856	11.6359865	1.264129462	2.40182238	1.56E-05	0.00086151	SLC2A4
ENSG00000128578	76.0145856	0.701644949	1.6263581	1.58E-05	0.00086946	STRIP2
ENSG00000169679	1036.34536	0.66993865	1.59100531	1.60E-05	0.00088129	BUB1
ENSG00000165480	193.005162	0.92314419	1.89624344	1.62E-05	0.00088904	SKA3
ENSG00000087237	74.3432279	1.178419863	2.26328751	1.63E-05	0.00088935	CETP
ENSG00000165633	16.0532562	1.140247347	2.2041881	1.64E-05	0.00089126	VSTM4
ENSG00000156127	74.8655199	1.052922504	2.07472842	1.64E-05	0.00089126	BATF
ENSG00000097046	313.473262	0.664808671	1.58535801	1.72E-05	0.00093124	CDC7
ENSG00000035499	283.253114	0.788897268	1.72775334	1.75E-05	0.00094127	DEPDC1B
ENSG00000186074	52.2331749	1.296341418	2.45605253	1.79E-05	0.00096253	CD300LF
ENSG00000247317	23.2741343	1.093512585	2.13392961	1.83E-05	0.0009784	NA
ENSG00000183960	61.2915158	1.563047368	2.95477314	1.87E-05	0.00099353	KCNH8
ENSG00000126264	135.224934	0.905542095	1.87324824	1.87E-05	0.00099362	HCST
ENSG00000123473	338.76946	0.598184568	1.51381045	1.92E-05	0.00101058	STIL
ENSG00000205307	15.9012182	1.991355945	3.97610524	1.99E-05	0.00103508	SAP25
ENSG00000159399	1440.55829	0.829618902	1.77721584	1.99E-05	0.00103508	HK2
ENSG00000163421	57.0673792	1.631610549	3.09858716	2.02E-05	0.00104836	PROK2
ENSG00000131187	382.195415	0.791921029	1.73137836	2.04E-05	0.00105164	F12
ENSG00000011426	430.006343	0.79000799	1.72908404	2.04E-05	0.00105164	ANLN
ENSG00000253898	148.791663	2.105931848	4.30475714	2.07E-05	0.00106134	LINC01419
ENSG00000143476	629.33402	0.899749721	1.86574229	2.08E-05	0.00106151	DTL
ENSG00000140534	203.952595	0.95025807	1.93221826	2.09E-05	0.00106155	TICRR
ENSG00000121316	239.500055	1.118942484	2.17187712	2.10E-05	0.00106296	PLBD1
ENSG00000104970	374.831235	1.560639407	2.94984553	2.13E-05	0.00107261	KIR3DX1
ENSG00000256546	28.5691231	0.892337301	1.85618088	2.16E-05	0.00108237	LOC100506691
ENSG00000160255	854.602467	1.004847321	2.00673112	2.21E-05	0.00110199	ITGB2
ENSG00000092964	152.764875	0.974866123	1.9654588	2.24E-05	0.00110673	DPYSL2
ENSG00000100526	249.029989	0.688939675	1.61209825	2.24E-05	0.00110673	CDKN3
ENSG00000140848	179.146996	0.660510749	1.58064211	2.22E-05	0.00110673	CPNE2
ENSG00000226674	65.3917416	1.221057616	2.33117549	2.32E-05	0.00113506	TEX41
ENSG00000183484	193.122291	1.409159249	2.65582346	2.34E-05	0.0011403	GPR132
ENSG00000125319	125.875205	0.661729412	1.58197786	2.34E-05	0.0011403	HROB
ENSG00000186818	5175.09806	1.090356452	2.12926639	2.36E-05	0.00114835	LILRB4
ENSG00000149926	30.8887206	1.015095444	2.02103659	2.38E-05	0.00114922	TLCD3B
ENSG00000026652	196.497154	0.848534601	1.80067099	2.37E-05	0.00114922	AGPAT4
ENSG00000186417	22.3191275	1.556004873	2.94038461	2.43E-05	0.00116531	GLDN
ENSG00000159450	18.5507348	1.658727016	3.15737806	2.51E-05	0.00119477	TCHH
ENSG00000138792	87.9934472	1.344061022	2.53864915	2.52E-05	0.00119674	ENPEP
ENSG00000148346	328.263372	1.601237014	3.0340335	2.54E-05	0.00120319	LCN2
ENSG00000196639	13.7495413	1.433118368	2.70029751	2.55E-05	0.00120319	HRH1
ENSG00000197077	45.3311288	1.18584761	2.27497014	2.55E-05	0.00120319	KIAA1671
ENSG00000196664	812.149055	1.249151641	2.37701604	2.63E-05	0.00123599	TLR7
ENSG00000167670	1096.23458	0.587369617	1.50250482	2.65E-05	0.00123731	CHAF1A
ENSG00000109881	213.558042	0.705445435	1.63064805	2.68E-05	0.00124488	CCDC34
ENSG00000121053	604.186143	1.544694462	2.91742276	2.71E-05	0.00124885	EPX
ENSG00000121742	69.8478977	1.728086837	3.31288204	2.72E-05	0.00125309	GJB6
ENSG00000265148	107.695607	0.661208176	1.58140641	2.74E-05	0.00125703	TSPOAP1-AS1
ENSG00000117724	1122.58907	0.841832057	1.79232474	2.79E-05	0.00127437	CENPF
ENSG00000227619	66.3501611	1.420335665	2.67647776	2.85E-05	0.00128864	NA
ENSG00000112118	2110.62097	0.601853465	1.5176651	2.87E-05	0.00129755	MCM3
ENSG00000161944	11.6202511	1.706761359	3.26427219	2.89E-05	0.00130314	ASGR2
ENSG00000182511	174.870543	0.88204708	1.84298851	2.95E-05	0.00132641	FES
ENSG00000165617	70.7064382	1.3462634	2.54252754	2.99E-05	0.00133754	DACT1
ENSG00000205683	252.81233	0.987284669	1.98245026	2.99E-05	0.00133754	DPF3
ENSG00000093009	308.756784	0.874219088	1.83301563	3.02E-05	0.00134067	CDC45
ENSG00000156136	528.347603	0.617707629	1.5344351	3.01E-05	0.00134067	DCK
ENSG00000164109	724.898939	0.587133089	1.5022585	3.02E-05	0.00134067	MAD2L1
ENSG00000225968	23.6320509	1.891778815	3.71092493	3.10E-05	0.00137181	ELFN1
ENSG00000182871	256.570825	0.97627384	1.96737754	3.28E-05	0.00143653	COL18A1
ENSG00000041515	14.5844464	1.149292368	2.21805074	3.33E-05	0.001458	MYO16
ENSG00000143653	733.572822	0.841760712	1.79223611	3.38E-05	0.00147676	SCCPDH
ENSG00000165304	309.967636	0.766799381	1.70149084	3.41E-05	0.00148551	MELK
ENSG00000140263	442.968493	0.638188116	1.55637328	3.42E-05	0.00148953	SORD
ENSG00000090376	161.236142	0.870717958	1.82857266	3.44E-05	0.00149402	IRAK3
ENSG00000112320	23.703255	1.238550954	2.35961413	3.48E-05	0.00149741	SOBP
ENSG00000162722	150.855447	1.092893965	2.13301478	3.47E-05	0.00149741	TRIM58
ENSG00000184661	191.910395	0.84615105	1.79769846	3.49E-05	0.00150002	CDCA2
ENSG00000168078	209.013529	0.843044285	1.79383138	3.50E-05	0.00150011	PBK
ENSG00000177990	129.861772	0.743671516	1.67443168	3.58E-05	0.00152822	DPY19L2
ENSG00000235162	277.323069	1.001744488	2.00241984	3.68E-05	0.00155933	C12orf75
ENSG00000187808	21.594919	1.014462559	2.0201502	3.72E-05	0.00157245	SOWAHD
ENSG00000156970	632.236522	0.796007238	1.73628917	3.83E-05	0.00161016	BUB1B
ENSG00000071539	280.758234	0.84754055	1.79943071	3.93E-05	0.00163495	TRIP13
ENSG00000167634	185.693317	2.061208434	4.17335728	3.95E-05	0.0016395	NLRP7
ENSG00000124191	259.595672	1.421733996	2.67907319	3.97E-05	0.00164366	TOX2
ENSG00000134057	526.457869	0.744367908	1.67524013	4.03E-05	0.00165987	CCNB1
ENSG00000172985	464.707463	1.385257743	2.61218621	4.17E-05	0.00170231	SH3RF3
ENSG00000149557	71.5321453	0.983573925	1.97735777	4.16E-05	0.00170231	FEZ1
ENSG00000129810	138.259464	0.779740432	1.71682196	4.18E-05	0.00170231	SGO1
ENSG00000259129	56.6664056	1.3609281	2.56850361	4.22E-05	0.00171271	LINC00648
ENSG00000256417	20.4972592	1.722149562	3.29927621	4.34E-05	0.00171805	NA
ENSG00000227953	36.0816145	1.560494363	2.94954897	4.30E-05	0.00171805	NA
ENSG00000239998	248.322839	1.268946715	2.40985562	4.34E-05	0.00171805	LILRA2
ENSG00000254101	95.5674063	1.023977668	2.03351787	4.32E-05	0.00171805	LINC02055
ENSG00000154096	347.537412	2.208079278	4.62059704	4.46E-05	0.00175355	THY1
ENSG00000146267	41.3744836	1.27785094	2.4247751	4.59E-05	0.00180208	FAXC
ENSG00000196584	314.941965	0.675231022	1.59685246	4.60E-05	0.00180318	XRCC2
ENSG00000186638	165.694572	0.620498553	1.53740637	4.61E-05	0.00180318	KIF24
ENSG00000184371	179.574469	0.870610636	1.82843664	4.70E-05	0.00183123	CSF1
ENSG00000151651	1142.43656	0.835831117	1.78488499	4.74E-05	0.0018429	ADAM8
ENSG00000198203	46.6055579	1.330276859	2.51450925	4.83E-05	0.00186007	SULT1C2
ENSG00000213468	128.700269	0.705029903	1.63017845	4.82E-05	0.00186007	FIRRE
ENSG00000115457	56.8652204	1.278050727	2.42511091	4.85E-05	0.00186117	IGFBP2
ENSG00000129195	93.0976691	1.035209466	2.04941118	4.84E-05	0.00186117	PIMREG
ENSG00000160883	214.072489	1.068274294	2.09692359	4.92E-05	0.00188469	HK3
ENSG00000158869	241.428577	0.985390753	1.97984948	4.95E-05	0.00188514	FCER1G
ENSG00000100504	330.60505	0.931276521	1.90696256	4.95E-05	0.00188514	PYGL
ENSG00000271936	22.8516615	0.930827532	1.90636918	4.93E-05	0.00188514	NA
ENSG00000161800	685.105435	0.646093257	1.56492472	4.98E-05	0.0018895	RACGAP1
ENSG00000232006	17.2374433	1.283973909	2.435088	5.02E-05	0.00190095	NA
ENSG00000176208	330.651145	0.599388439	1.51507419	5.09E-05	0.00192519	ATAD5
ENSG00000069011	174.72358	1.383658137	2.60929152	5.20E-05	0.00195326	PITX1
ENSG00000268173	22.5681212	4.327635902	20.0792837	5.22E-05	0.00195416	NA
ENSG00000168995	29.8515151	1.547886551	2.92388496	5.22E-05	0.00195416	SIGLEC7
ENSG00000163449	9.35995069	1.089576074	2.12811494	5.25E-05	0.00196079	TMEM169
ENSG00000174837	62.4824286	1.085499574	2.1221102	5.38E-05	0.00199289	ADGRE1
ENSG00000116771	169.600621	0.782571048	1.72019373	5.47E-05	0.00201755	AGMAT
ENSG00000171608	360.028851	0.74785851	1.67929828	5.60E-05	0.00204586	PIK3CD
ENSG00000172243	439.63774	1.016639351	2.02320057	5.64E-05	0.00205308	CLEC7A
ENSG00000171320	139.911528	0.832516909	1.7807894	5.66E-05	0.00205308	ESCO2
ENSG00000076382	1065.7468	0.633455803	1.55127644	5.66E-05	0.00205308	SPAG5
ENSG00000160932	5595.25195	0.739477633	1.66957122	5.85E-05	0.00210835	LY6E
ENSG00000251629	43.7968647	1.736508625	3.33227768	6.01E-05	0.00216212	LINC02241
ENSG00000259692	10.7665789	1.331744677	2.51706884	6.04E-05	0.00216675	NA
ENSG00000110651	1379.57619	0.758798942	1.69208136	6.06E-05	0.00216763	CD81
ENSG00000112742	258.959717	0.749186803	1.68084513	6.25E-05	0.00221363	TTK
ENSG00000088305	163.077145	0.840165743	1.7902558	6.32E-05	0.00223027	DNMT3B
ENSG00000169710	3261.66873	0.592873423	1.50824774	6.33E-05	0.00223201	FASN
ENSG00000163823	730.820126	1.012902983	2.01796756	6.43E-05	0.0022646	CCR1

ENSG00000011332	37.5522631	0.810274077	1.75354454	6.47E-05	0.0022695	DPF1
ENSG00000100031	1620.88579	0.609578665	1.52581353	6.59E-05	0.00230096	GGT1
ENSG00000167767	16.7107884	2.535913198	5.79943839	6.62E-05	0.0023062	KRT80
ENSG00000188060	56.57687	0.979365279	1.9715978	6.62E-05	0.0023062	RAB42
ENSG00000100479	104.282271	0.815153893	1.75948583	6.73E-05	0.00233245	POLE2
ENSG00000223812	13.1630383	2.671293636	6.37000116	6.95E-05	0.00238682	NA
ENSG00000250874	32.0376771	1.279493878	2.427538	6.97E-05	0.00238918	NA
ENSG00000072840	219.956456	1.382792667	2.60772668	7.11E-05	0.00242285	EVC
ENSG00000153404	282.640273	1.159430539	2.23369242	7.15E-05	0.00242826	PLEKHG4B
ENSG00000121210	453.618114	0.808105393	1.75091056	7.27E-05	0.00245817	TMEM131L
ENSG00000002746	31.4994028	1.95949996	3.88927153	7.34E-05	0.00247923	HECW1
ENSG00000156886	81.9449101	1.333832842	2.5207147	7.59E-05	0.00254988	ITGAD
ENSG00000163154	28.5718313	1.224558476	2.33683922	7.68E-05	0.00257801	TNFAIP8L2
ENSG00000130203	1267.68939	1.006426861	2.0089294	7.72E-05	0.00258491	APOE
ENSG00000163009	27.3460065	0.916218618	1.88716245	7.74E-05	0.0025857	NA
ENSG00000138795	345.880963	1.143882877	2.20974957	7.97E-05	0.00265794	LEF1
ENSG00000177191	20.2758221	0.686429697	1.60929599	7.99E-05	0.00266005	B3GNT8
ENSG00000140836	97.366778	0.776014929	1.71239429	8.42E-05	0.00276872	ZFHX3
ENSG00000160207	16.6703706	0.787559682	1.72615221	8.47E-05	0.00277699	HSF2BP
ENSG00000204936	248.055061	1.55153642	2.93129145	8.71E-05	0.00278265	CD177
ENSG00000133789	136.058347	0.815825931	1.76030562	8.53E-05	0.00278265	SWAP70
ENSG00000106868	227.916124	0.742513074	1.6730877	8.71E-05	0.00278265	SUSD1
ENSG00000148908	313.139476	0.736947212	1.66664543	8.50E-05	0.00278265	RGS10
ENSG00000177575	678.791962	0.975387429	1.96616913	8.78E-05	0.00278603	CD163
ENSG00000119969	473.372879	0.645633757	1.56442637	8.84E-05	0.00279364	HELLS
ENSG00000175643	120.837516	0.902411453	1.86918771	8.88E-05	0.00279947	RMI2
ENSG00000102384	95.1875267	0.847326939	1.7991643	8.89E-05	0.00279947	CENPI
ENSG00000254811	18.8410647	1.395444915	2.63069668	8.96E-05	0.00281358	LOC101928535
ENSG00000150722	24.9459813	1.226735045	2.34036743	8.95E-05	0.00281358	PPP1R1C
ENSG00000232109	24.3040495	1.459094723	2.7493579	9.01E-05	0.00282564	VN1R54P
ENSG00000150394	93.4720667	1.284500995	2.43597782	9.16E-05	0.0028686	CDH8
ENSG00000225530	43.2625342	1.577760865	2.98506193	9.17E-05	0.00286926	NA
ENSG00000067057	340.190806	1.027013873	2.03780199	9.27E-05	0.00287392	PFKP
ENSG00000170458	215.046622	0.908237141	1.87675086	9.20E-05	0.00287392	CD14
ENSG00000028137	457.019469	0.89812116	1.86363736	9.24E-05	0.00287392	TNFRSF1B
ENSG00000189057	405.864672	0.821240173	1.76692423	9.29E-05	0.00287392	FAM111B
ENSG00000173369	805.235934	1.203656825	2.30322735	9.80E-05	0.00299673	C1QB
ENSG00000087586	223.86523	0.822268016	1.76818351	9.76E-05	0.00299673	AURKA
ENSG00000160117	215.297302	0.620191774	1.53707949	9.79E-05	0.00299673	ANKLE1
ENSG00000150540	60.2637418	0.949495522	1.93119724	0.00010025	0.00305773	HNMT
ENSG00000132669	39.4286652	1.143705101	2.20947729	0.00010322	0.00312657	RIN2
ENSG00000225988	113.082996	1.370014642	2.58473189	0.00010388	0.00314251	LAMP5-AS1
ENSG00000186193	525.101818	0.674399903	1.5959328	0.00010814	0.00324906	SAPCD2
ENSG00000234722	701.957784	1.112678998	2.16246832	0.00010853	0.0032562	LINC01287
ENSG00000164125	46.8859953	1.157442878	2.23061709	0.0001097	0.00328692	GASK1B
ENSG00000094804	976.789888	0.59970227	1.5154038	0.00011004	0.00329263	CDC6
ENSG00000145526	56.9114621	1.547532886	2.92316828	0.00011059	0.00329569	CDH18
ENSG00000082482	37.9252814	2.731007578	6.63919156	0.00011234	0.00332107	KCNK2
ENSG00000238269	135.366554	1.587631303	3.00555476	0.00011492	0.0033885	PAGE2B
ENSG00000092470	525.655492	0.613857797	1.53034592	0.00011584	0.00340633	WDR76
ENSG00000124507	531.896895	1.029570309	2.04141615	0.00011762	0.0034367	PACSIN1
ENSG00000141576	820.229666	0.588348582	1.50352471	0.00011765	0.0034367	RNF157
ENSG00000188001	71.5013302	1.056973718	2.08056263	0.00011865	0.00345237	TPRG1
ENSG00000162496	31.1403828	1.206836361	2.308309	0.00011902	0.00345419	DHRS3
ENSG00000008441	102.012042	0.712684542	1.63885083	0.00012059	0.00348596	NFIX
ENSG00000169860	37.7278815	1.049912373	2.07040409	0.00012256	0.00353836	P2RY1
ENSG00000053108	63.5050444	1.164441636	2.24146448	0.00012309	0.00354335	FSTL4
ENSG00000099282	21.3085426	1.088910731	2.12713372	0.00012296	0.00354335	TSPAN15
ENSG00000108679	1734.00634	0.768569778	1.70358009	0.00012343	0.00354335	LGALS3BP
ENSG00000186603	151.424458	0.654512084	1.57408351	0.00012402	0.00355272	HPDL
ENSG00000128510	12.8707116	3.218100053	9.30560567	0.00012519	0.00357571	CPA4
ENSG00000136295	414.888334	0.661562098	1.58179441	0.00012521	0.00357571	TTYH3
ENSG00000156414	16.7183734	1.751430032	3.36692138	0.00012709	0.00362179	TDRD9
ENSG00000187621	168.281589	0.731412748	1.6602641	0.00012769	0.00362958	NA
ENSG00000120949	52.8387963	1.271385776	2.41393324	0.00012861	0.00365101	TNFRSF8
ENSG00000137491	250.075977	1.162640276	2.23866751	0.00012909	0.00366004	SLCO2B1
ENSG00000159335	535.066146	0.62995488	1.54751659	0.00012977	0.00366513	PTMS
ENSG00000134827	31.6044658	1.595068181	3.02108794	0.0001307	0.00368664	TCN1
ENSG00000171405	38.5957993	1.392097555	2.62459998	0.0001313	0.00368947	XAGE5
ENSG00000100116	235.557297	0.726569191	1.65469944	0.00013123	0.00368947	GCAT
ENSG00000198054	74.1699423	1.902518556	3.73865295	0.00013167	0.00369526	DSCR8
ENSG00000136011	51.2138543	1.131641836	2.19107951	0.00013243	0.00370274	STAB2
ENSG00000184949	68.907553	0.669864253	1.59092327	0.00013244	0.00370274	FAM227A
ENSG00000228340	71.1255525	0.856571313	1.81072984	0.00013731	0.0038244	MIR646HG
ENSG00000108846	52.6980645	1.035003742	2.04911896	0.0001402	0.0038904	ABCC3
ENSG00000196154	3610.15878	0.853004949	1.80625922	0.00014045	0.00389227	S100A4
ENSG00000203747	538.233893	1.110506213	2.15921397	0.00014137	0.00390489	FCGR3A
ENSG00000165996	14.0271348	1.214657097	2.32085614	0.00014346	0.00393143	HACD1
ENSG00000101003	211.608947	0.839486982	1.78941372	0.00014511	0.00396195	GINS1
ENSG00000249395	81.3539288	1.185927765	2.27509654	0.00014673	0.00399636	CASC9
ENSG00000231793	27.2629797	0.715349874	1.64188135	0.00014895	0.00405185	NA
ENSG00000189212	77.8892752	0.731173682	1.659989	0.0001509	0.00407985	NA
ENSG00000180549	24.7970059	1.718885685	3.29182054	0.0001535	0.00414006	FUT7
ENSG00000100628	166.300639	1.160567972	2.23545417	0.00015333	0.00414006	ASB2
ENSG00000163046	63.0745827	1.406501914	2.65093613	0.00015485	0.00414601	ANKRD30BL
ENSG00000115112	29.5517096	1.245727744	2.37138144	0.00015957	0.00424176	TFCP2L1
ENSG00000254979	36.2939681	1.424766594	2.68471063	0.00016407	0.00434043	NA
ENSG00000150760	93.971633	1.181461192	2.26806375	0.00016719	0.00440723	DOCK1
ENSG00000236279	30.2505539	2.283974119	4.87017671	0.00016836	0.00442242	CLEC2L
ENSG00000067191	165.968884	0.762907669	1.6969072	0.00016918	0.00443837	CACNB1
ENSG00000230561	21.1056097	1.001988881	2.00275908	0.00017135	0.00447948	CCDC192
ENSG00000133119	279.437443	0.622021009	1.53902963	0.00017228	0.00449325	RFC3
ENSG00000133710	356.026555	0.855953705	1.80995484	0.00017532	0.00454053	SPINK5
ENSG00000198542	40.7315458	1.442922454	2.71871035	0.00017609	0.0045552	ITGBL1
ENSG00000118193	234.502627	0.810842194	1.7542352	0.00017897	0.00461479	KIF14
ENSG00000038427	512.969813	0.862835177	1.81860872	0.0001793	0.00461647	VCAN
ENSG00000255197	17.178199	1.058372034	2.08258017	0.00017958	0.00461852	NA
ENSG00000106003	38.0714661	1.167188703	2.24573656	0.00018093	0.00463172	LFNG
ENSG00000267534	292.45236	0.645346579	1.56411499	0.00018206	0.00464438	S1PR2
ENSG00000237594	10.7649076	1.260087021	2.39510187	0.00018291	0.00465533	LOC150051
ENSG00000198885	123.78613	0.923433907	1.89662428	0.00018287	0.00465533	ITPRIPL1
ENSG00000110987	72.6440428	1.034624654	2.0485806	0.00018369	0.00466995	BCL7A
ENSG00000118257	154.782729	0.955709553	1.93953331	0.00018486	0.00468894	NRP2
ENSG00000215548	76.6500786	0.839547804	1.78948916	0.00018579	0.00470696	NA
ENSG00000054793	106.22091	1.099781347	2.14322208	0.00018652	0.00472002	ATP9A
ENSG00000186174	377.814899	0.725588997	1.65357559	0.00018754	0.00472985	BCL9L
ENSG00000181938	174.671034	0.655889501	1.57558709	0.00019391	0.0048573	GINS3
ENSG00000155254	281.86674	0.64443866	1.56313097	0.00019376	0.0048573	MARVELD1
ENSG00000270038	10.320868	1.19935303	2.29636668	0.00019421	0.00485913	NA
ENSG00000167601	127.943236	0.914425303	1.88481811	0.00019804	0.00493277	AXL
ENSG00000196924	19027.8136	0.671080351	1.59226488	0.00019845	0.00493505	FLNA
ENSG00000273162	8.90134307	1.014869129	2.02071958	0.00020089	0.0049842	NA
ENSG00000266036	37.8908277	0.730557066	1.65927966	0.00020123	0.0049842	NA
ENSG00000111752	426.466538	0.636799608	1.55487608	0.00020106	0.0049842	PHC1
ENSG00000176884	25.9064932	1.630402628	3.0959939	0.00020274	0.00501027	GRIN1
ENSG00000182853	12.0548085	0.906109494	1.87398512	0.00020374	0.00502376	VMO1
ENSG00000258927	30.2543703	1.236617435	2.35645386	0.00020509	0.00505161	NA
ENSG00000169607	314.723674	0.6877627	1.61078361	0.00021163	0.00518959	CKAP2L
ENSG00000095002	710.17862	0.606221957	1.52226756	0.00021246	0.00520413	MSH2
ENSG00000167100	23.0613666	0.902136287	1.86883123	0.00021425	0.00524218	SAMD14
ENSG00000260997	68.4728559	1.053971246	2.07623716	0.00021469	0.00524709	NA
ENSG00000164023	60.3904616	1.155596447	2.22776406	0.00021842	0.00532335	SGMS2
ENSG00000142185	226.747333	0.91641691	1.88742185	0.00021853	0.00532335	TRPM2
ENSG00000182912	107.759687	1.320132516	2.49689043	0.00022099	0.00535979	NA
ENSG00000165495	37.7745281	1.11627372	2.1678632	0.0002229	0.0054002	PKNOX2
ENSG00000184500	91.746036	0.958724441	1.94359071	0.0002305	0.00553575	PROS1
ENSG00000141524	5025.35298	0.924090794	1.89748804	0.00023238	0.00556877	TMC6
ENSG00000150782	36.8029747	1.0283575	2.03970074	0.00023427	0.00560206	IL18

ENSG00000188483	129.490419	0.759344025	1.69272079	0.00023506	0.00561476	IER5L
ENSG00000185615	3556.09292	1.371850225	2.58802262	0.00023783	0.00565895	PDIA2
ENSG00000174600	61.5448969	1.216067705	2.32312648	0.00023803	0.00565895	CMKLR1
ENSG00000170439	7.12941948	1.853578229	3.61395422	0.00023918	0.00566841	METTL7B
ENSG00000007237	291.252318	0.952705107	1.93549839	0.00023908	0.00566841	GAS7
ENSG00000205642	23.5837317	1.354523111	2.55712575	0.00023962	0.00566875	VCX3B
ENSG00000224195	8.99933892	0.713758785	1.64007158	0.0002415	0.00569783	NA
ENSG00000163535	265.314049	0.654279279	1.57382953	0.00024214	0.00570399	SGO2
ENSG00000186081	60.990921	2.50961229	5.69467019	0.00024414	0.00573882	KRT5
ENSG00000012223	1454.86297	1.155625439	2.22780883	0.00024835	0.00580221	LTF
ENSG00000179344	402.790193	1.041074821	2.05776013	0.00024831	0.00580221	HLA-DQB1
ENSG00000081237	2784.47725	0.857961896	1.81247601	0.00025042	0.00583069	PTPRC
ENSG00000187116	82.704336	1.150093276	2.21928243	0.00025658	0.00594303	LILRA5
ENSG00000106484	400.692729	0.66355	1.58397548	0.00026155	0.00603052	MEST
ENSG00000223518	17.7648526	1.701706101	3.25285407	0.00026331	0.00604824	NA
ENSG00000133800	65.3727606	1.123746151	2.17912076	0.00026496	0.00607348	LYVE1
ENSG00000125384	154.600691	1.008129207	2.01130128	0.00026819	0.00611594	PTGER2
ENSG00000121351	7.44843115	1.586330505	3.00284603	0.00026861	0.00611908	IAPP
ENSG00000224689	34.9699212	1.568502616	2.96596714	0.00027007	0.0061398	NA
ENSG00000178026	67.4939554	0.731790916	1.66069935	0.00027908	0.00632504	LRRC75B
ENSG00000139832	61.3284552	1.026192585	2.03664225	0.00028274	0.00638844	RAB20
ENSG00000140092	17.0068759	1.550124167	2.92842342	0.00028564	0.0064419	FBLN5
ENSG00000123700	17.1797133	1.195985723	2.29101312	0.00028882	0.00649265	KCNJ2
ENSG00000127423	45.4536228	0.769189131	1.7043116	0.00028875	0.00649265	AUNIP
ENSG00000158486	58.3318051	0.70355101	1.62850823	0.00029225	0.00654994	DNAH3
ENSG00000061918	46.119963	1.046260294	2.06516964	0.0002937	0.00657036	GUCY1B1
ENSG00000113749	74.7672815	1.233393438	2.35119376	0.00029816	0.00662865	HRH2
ENSG00000235641	21.8582936	1.522720333	2.87332331	0.00030319	0.00671626	NA
ENSG00000152583	121.661113	1.062275363	2.08822239	0.00030505	0.00672812	SPARCL1
ENSG00000126759	209.655801	0.603258481	1.51914384	0.00030487	0.00672812	CFP
ENSG00000205609	547.896947	1.284566842	2.436089	0.0003079	0.00677711	EIF3CL
ENSG00000225675	14.5408815	0.897549901	1.86289957	0.00031306	0.00687025	LINC01771
ENSG00000248740	9.6650532	1.68445197	3.21418277	0.00031395	0.00688288	NA
ENSG00000177238	117.149887	0.927364254	1.90179831	0.00031464	0.00689116	TRIM72
ENSG00000173218	173.43783	0.985595559	1.98013056	0.00031654	0.00692611	VANGL1
ENSG00000204136	99.0947988	1.098982419	2.14203554	0.00031755	0.00693724	GGTA1
ENSG00000134198	57.6956362	1.084580091	2.12075813	0.00031768	0.00693724	TSPAN2
ENSG00000273373	77.9372612	0.683467039	1.6059946	0.00031906	0.00695378	NA
ENSG00000122386	161.445269	0.843406634	1.79428198	0.00032052	0.00697869	ZNF205
ENSG00000249790	179.663992	0.715118821	1.64161842	0.00032334	0.00701925	NA
ENSG00000134256	20.228329	0.78430579	1.72226338	0.00032512	0.00705103	CD101
ENSG00000139567	34.3841957	1.233821598	2.35189165	0.00032626	0.00706894	ACVRL1
ENSG00000101916	49.7402803	1.004622466	2.00641838	0.00032858	0.00711221	TLR8
ENSG00000105643	172.494087	0.777454858	1.71410425	0.0003297	0.00712239	ARRDC2
ENSG00000169994	9.05496452	1.35936816	2.56572787	0.00033534	0.00720544	MYO7B
ENSG00000115526	96.4064201	1.185578448	2.27454574	0.00034085	0.00729218	CHST10
ENSG00000004399	297.075707	0.782255927	1.71981803	0.00035335	0.00750162	PLXND1
ENSG00000118971	7819.34904	0.99835059	1.99771474	0.00036872	0.00772496	CCND2
ENSG00000144395	87.3706478	0.717941487	1.64483342	0.00037428	0.00781852	CCDC150
ENSG00000174669	67.907154	0.726000992	1.65404787	0.00037761	0.0078658	SLC29A2
ENSG00000127124	156.026032	0.74033471	1.67056337	0.00037987	0.00790557	HIVEP3
ENSG00000102021	15.1107187	10.33820177	1294.5202	0.00038233	0.00794919	LUZP4
ENSG00000156269	38.3975827	1.812749287	3.51311131	0.00038589	0.0080008	NAA11
ENSG00000124126	1487.75137	0.91492738	1.88547416	0.00038807	0.00802207	PREX1
ENSG00000100644	1569.65886	0.695332622	1.61925772	0.00038746	0.00802207	HIF1A
ENSG00000233791	84.7985946	0.675681799	1.59735148	0.00038837	0.00802207	LINC01136
ENSG00000023902	1358.08795	0.720955996	1.64827389	0.00039101	0.00806905	PLEKHO1
ENSG00000261761	70.3677364	1.082163861	2.11720925	0.00039806	0.008184	LINC02616
ENSG00000213160	63.6757044	0.653548676	1.57303272	0.00040228	0.00825541	KLHL23
ENSG00000157985	367.561086	1.105056206	2.15107258	0.00040337	0.00826949	AGAP1
ENSG00000163808	320.186406	0.598831977	1.51448992	0.00040727	0.0083193	KIF15
ENSG00000204472	265.580815	0.944540895	1.92457634	0.00041019	0.00836362	AIF1
ENSG00000169575	11.6961699	2.221077017	4.66241368	0.00041062	0.00836469	VPREB1
ENSG00000267665	17.7142604	1.648808716	3.13574603	0.00041101	0.00836489	LOC400622
ENSG00000109265	22.3383682	1.69356384	3.23454737	0.00041475	0.00842551	CRACD
ENSG00000135636	190.278099	0.970090541	1.95896353	0.00041896	0.00847998	DYSF
ENSG00000160179	304.876354	0.71746139	1.64428615	0.00042594	0.00858438	ABCG1
ENSG00000230185	22.5310201	0.595135403	1.51061436	0.00042535	0.00858438	NA
ENSG00000272143	21.1977575	1.106623007	2.15340997	0.00043559	0.00872094	FGF14-AS2
ENSG00000079215	60.871903	0.916253311	1.88720783	0.00044409	0.00886704	SLC1A3
ENSG00000101439	6657.36612	0.641021854	1.55943331	0.00045182	0.0089889	CST3
ENSG00000145685	192.960176	0.63164177	1.54932711	0.00045681	0.00907999	LHFPL2
ENSG00000101695	222.84094	0.718153964	1.64507569	0.00045868	0.00910564	RNF125
ENSG00000173372	721.333378	0.980300696	1.97287657	0.00046367	0.00917523	C1QA
ENSG00000153253	684.336695	1.141297378	2.20579295	0.00046616	0.00919975	SCN3A
ENSG00000196735	387.151129	0.906711728	1.87476755	0.0004659	0.00919975	HLA-DQA1
ENSG00000104974	36.0851412	1.0413049	2.05808833	0.00046714	0.0092028	LILRA1
ENSG00000196834	79.6218096	1.239338513	2.36090258	0.00046772	0.0092059	POTEI
ENSG00000166483	889.621444	0.673358882	1.59478162	0.00047275	0.00925371	WEE1
ENSG00000100427	131.834155	1.5584437	2.94535943	0.00047833	0.00933999	MLC1
ENSG00000205628	23.4791045	1.699083921	3.24694719	0.00048159	0.00938982	LINC01446
ENSG00000072952	37.5891379	0.904619413	1.87205058	0.00048173	0.00938982	IRAG1
ENSG00000253522	195.282149	1.057222169	2.08092096	0.00048417	0.009414	MIR3142HG
ENSG00000100292	1679.42659	0.826894445	1.77386282	0.00048387	0.009414	HMOX1
ENSG00000158270	46.9070731	0.938420613	1.91642908	0.00048629	0.00944547	COLEC12
ENSG00000159189	672.694861	1.05570699	2.07873664	0.00049869	0.00961898	C1QC
ENSG00000251381	60.0849196	1.055683396	2.07870264	0.00050017	0.00961937	LINC00958
ENSG00000140678	342.050011	0.865479325	1.8219449	0.00050032	0.00961937	ITGAX
ENSG00000163106	9.74854404	1.528937644	2.88573265	0.00050165	0.00962575	HPGDS
ENSG00000166825	221.68843	0.828591461	1.77595061	0.00050378	0.00964984	ANPEP
ENSG00000145850	392.757889	0.852499789	1.80562687	0.0005066	0.00967039	TIMD4
ENSG00000267751	18.4744719	0.623128269	1.54021128	0.00050649	0.00967039	LOC105372233
ENSG00000236663	61.5319741	1.032339754	2.04533868	0.00051155	0.00973127	FRGCA
ENSG00000181374	6.21376302	1.681355414	3.20729135	0.00051251	0.00973989	CCL13
ENSG00000254349	16.8113404	1.460804668	2.75261849	0.00051376	0.00973989	MIR2052HG
ENSG00000221869	128.078222	0.959603338	1.94477512	0.00052486	0.00992486	CEBPD
ENSG00000085265	647.358465	1.051517502	2.07270888	0.00052829	0.0099727	FCN1
ENSG00000172292	934.346941	0.75670399	1.68962606	0.00053061	0.00998979	CERS6
ENSG00000196209	32.5558077	1.105659876	2.15197285	0.00053488	0.01004387	SIRPB2
ENSG00000136231	91.2558335	1.064651648	2.09166477	0.00053569	0.01004387	IGF2BP3
ENSG00000136943	42.5852305	0.861310826	1.81668819	0.00053557	0.01004387	CTSV
ENSG00000132205	99.8851187	0.836372533	1.78555495	0.00054628	0.01018653	EMILIN2
ENSG00000134755	35.8586905	0.996008711	1.99447454	0.00054816	0.01018985	DSC2
ENSG00000164742	129.267041	0.959069846	1.94405609	0.00054854	0.01018985	ADCY1
ENSG00000136960	220.977131	1.133534467	2.19395581	0.00055411	0.01027615	ENPP2
ENSG00000100985	767.203017	1.138070702	2.20086507	0.00055841	0.01032628	MMP9
ENSG00000109743	63.2026805	0.971506009	1.96088647	0.00056062	0.01034452	BST1
ENSG00000070371	61.4944229	0.751927196	1.68404092	0.00056107	0.01034452	CLTCL1
ENSG00000114200	753.200897	1.337624943	2.52734908	0.00056238	0.01036002	BCHE
ENSG00000138758	1369.15939	0.647771999	1.56674675	0.00056413	0.0103836	SEPTIN11
ENSG00000268849	15.0758053	1.731400934	3.320501	0.00056603	0.01040885	NA
ENSG00000137825	42.0215953	0.719431941	1.64653359	0.00056691	0.01040885	ITPKA
ENSG00000087842	79.4544934	0.650733693	1.56996641	0.00056649	0.01040885	PIR
ENSG00000204252	70.3635871	0.931628493	1.90742786	0.00056868	0.01043177	HLA-DOA
ENSG00000136235	595.5216	0.661014697	1.58119434	0.0005691	0.01043177	GPNMB
ENSG00000166803	427.008836	0.634552064	1.55245566	0.00058443	0.01061604	PCLAF
ENSG00000120885	382.802685	0.888412785	1.85113843	0.00058527	0.01062272	CLU
ENSG00000240322	6.22496922	0.935626448	1.912721	0.00058654	0.01063666	RN7SL481P
ENSG00000163221	595.588135	1.133489699	2.19388773	0.00059549	0.01074654	S100A12
ENSG00000090382	2302.75216	0.976991422	1.96835634	0.00060285	0.01086153	LYZ
ENSG00000169896	334.498517	1.051787426	2.07309672	0.00060518	0.01087708	ITGAM
ENSG00000162551	114.972717	1.047703346	2.06723635	0.00061042	0.01094461	ALPL
ENSG00000165905	27.5833292	1.258578579	2.39259893	0.00061132	0.01094582	LARGE2
ENSG00000168754	66.5445222	1.280775146	2.42969487	0.00061484	0.01098821	FAM178B
ENSG00000253424	9.59348995	0.775526701	1.71181489	0.00062173	0.01108444	NA
ENSG00000104918	28.9279603	1.458147394	2.74755316	0.0006259	0.01111786	RETN
ENSG00000120645	219.92192	1.074662438	2.1062292	0.00062712	0.01111786	IQSEC3
ENSG00000271590	19.5005126	0.826376889	1.77322658	0.00062941	0.01113185	NA

ENSG00000100162	167.223349	0.608058156	1.52420627	0.0006335	0.01116841	CENPM
ENSG00000189223	915.629356	0.614967979	1.531524	0.00064737	0.01139482	PAX8-AS1
ENSG00000131721	24.9394401	2.558125552	5.88941996	0.00065355	0.01145802	RHOXF2
ENSG00000249550	123.961578	0.65047668	1.56968675	0.00065805	0.01148241	LINC01234
ENSG00000133816	106.260337	0.86976014	1.82735906	0.00066834	0.0116026	MICAL2
ENSG00000271550	20.8456736	0.853244287	1.80655889	0.00066859	0.0116026	BNIP3P11
ENSG00000176912	23.8516599	0.800210019	1.7413546	0.00067037	0.01162406	TYMSOS
ENSG00000011028	57.2494426	0.968309532	1.95654668	0.00067661	0.01168643	MRC2
ENSG00000119121	35.9400205	1.054641378	2.0772018	0.00068222	0.01175371	TRPM6
ENSG00000204482	179.809444	0.979542803	1.97184042	0.00068629	0.01179847	LST1
ENSG00000043591	23.589962	1.3058226	2.47224651	0.00068978	0.01184011	ADRB1
ENSG00000106025	123.82676	0.878754957	1.83878775	0.00069301	0.01188267	TSPAN12
ENSG00000176046	73.1049496	1.009443231	2.01313404	0.00069817	0.01193525	NUPR1
ENSG00000167880	26.3915466	0.814382446	1.75854523	0.00070036	0.01194775	EVPL
ENSG00000075213	451.807691	1.014656266	2.02042145	0.00070153	0.01194929	SEMA3A
ENSG00000254788	24.2094702	0.631957675	1.5496664	0.00070104	0.01194929	CKLF-CMTM1
ENSG00000166927	281.267754	0.928794777	1.903685	0.00070585	0.01200436	MS4A7
ENSG00000215784	140.985829	0.916877026	1.8880239	0.00073155	0.01236541	FAM72D
ENSG00000137261	17.4964503	0.826586305	1.77348399	0.00074093	0.01247633	KIAA0319
ENSG00000204165	31.2206839	0.591802177	1.50712824	0.00074297	0.0124822	CXorf65
ENSG00000173801	680.01441	0.681495257	1.60380113	0.00074402	0.01248507	JUP
ENSG00000187244	60.5704871	0.789643265	1.72864697	0.00075008	0.01257308	BCAM
ENSG00000229618	20.3515185	0.907885916	1.87629402	0.00075271	0.01258848	NA
ENSG00000111110	244.100003	0.786724739	1.7251535	0.00075244	0.01258848	PPM1H
ENSG00000153993	256.283884	0.983675119	1.97749647	0.00076469	0.0127312	SEMA3D
ENSG00000169224	36.0371271	1.439171087	2.71165021	0.00077054	0.01279973	GCSAML
ENSG00000196126	668.597592	0.867347311	1.82430546	0.0007737	0.01282344	HLA-DRB1
ENSG00000125730	29.7745245	0.965172186	1.95229651	0.00077559	0.01284513	C3
ENSG00000198947	159.509856	0.880974941	1.84161941	0.00079458	0.01307168	DMD
ENSG00000266903	59.1842073	0.779917126	1.71703224	0.00079416	0.01307168	NA
ENSG00000172322	48.1997028	1.297959175	2.45880815	0.00079879	0.01308251	CLEC12A
ENSG00000259845	22.1886424	0.870929013	1.82884019	0.00079672	0.01308251	NA
ENSG00000224420	98.1182169	0.616301755	1.53294056	0.00079802	0.01308251	ADM5
ENSG00000100599	225.188283	0.863782909	1.81980379	0.00080466	0.01313981	RIN3
ENSG00000211670	156.103592	1.15343987	2.22443643	0.00082287	0.01337805	IGLV3-9
ENSG00000147324	174.40622	0.760404656	1.69396569	0.00082519	0.01340454	MFHAS1
ENSG00000242252	43.6080883	0.667399556	1.58820766	0.00083089	0.01346881	BGLAP
ENSG00000228526	53.7304805	1.05168777	2.07295352	0.00083274	0.01347589	MIR34AHG
ENSG00000159164	58.4411075	0.85659035	1.81075373	0.0008462	0.01361724	SV2A
ENSG00000129450	29.2330282	1.193748447	2.28746306	0.00085114	0.01367697	SIGLEC9
ENSG00000226941	15.7068033	9.69477879	828.741748	0.00086697	0.01389089	RBMY1J
ENSG00000186310	111.003226	0.861676349	1.81714853	0.00087227	0.01395547	NAP1L3
ENSG00000254044	9.52765335	1.092703216	2.13273278	0.00088069	0.01401946	NA
ENSG00000188610	137.634404	0.64934625	1.5684573	0.00088318	0.0140389	FAM72B
ENSG00000106123	50.2649054	1.133362252	2.19369393	0.00088747	0.0140629	EPHB6
ENSG00000188822	17.3365918	0.925902071	1.8998718	0.00088723	0.0140629	CNR2
ENSG00000184735	46.063455	1.234471937	2.35295208	0.00089326	0.0141282	DDX53
ENSG00000086967	621.707092	1.313458399	2.48536613	0.00089875	0.01417452	MYBPC2
ENSG00000196565	284.22703	1.06877443	2.09765065	0.00093766	0.01465243	HBG2
ENSG00000113327	180.341728	1.047658444	2.06717201	0.00093657	0.01465243	GABRG2
ENSG00000272711	46.237765	0.818806713	1.76394639	0.00093722	0.01465243	NA
ENSG00000183117	93.5092157	1.350982251	2.5508574	0.00094265	0.01470966	CSMD1
ENSG00000174175	39.7611673	1.257568727	2.39092475	0.00095842	0.01493476	SELP
ENSG00000106665	136.77711	0.747551253	1.67894067	0.00096163	0.0149743	CLIP2
ENSG00000161929	54.4759324	0.758533273	1.6917698	0.00096265	0.01497954	SCIMP
ENSG00000224659	166.253438	1.833548815	3.56412717	0.00096919	0.01503908	GAGE2E
ENSG00000000971	456.969656	0.937806998	1.91561415	0.00097335	0.01508691	CFH
ENSG00000182621	383.855926	0.897967567	1.86343897	0.00097363	0.01508691	PLCB1
ENSG00000256663	16.0041625	1.056881517	2.08042967	0.00097604	0.01510272	NA
ENSG00000036672	221.769542	0.931805456	1.90766184	0.00097738	0.01510272	USP2
ENSG00000118113	345.262954	1.222472527	2.3334629	0.00098236	0.01515844	MMP8
ENSG00000179242	32.1959227	1.039707109	2.05581025	0.00099386	0.01528274	CDH4
ENSG00000164867	110.514409	0.732416808	1.66141998	0.00099344	0.01528274	NOS3
ENSG00000043462	672.56033	0.727136583	1.65535034	0.00100605	0.01543799	LCP2
ENSG00000158525	31.2667734	1.109594942	2.15785054	0.00101217	0.01549963	CPA5
ENSG00000165457	122.010435	1.071155497	2.10111554	0.00102279	0.01560828	FOLR2
ENSG00000165891	463.091642	0.653996411	1.57352098	0.00102914	0.01568362	E2F7
ENSG00000073754	721.352795	0.841362551	1.79174155	0.00103842	0.01578161	CD5L
ENSG00000089012	22.6686909	1.348334433	2.54618003	0.00104759	0.01591001	SIRPG
ENSG00000101082	68.2064885	0.765695613	1.70018957	0.00106859	0.0161624	SLA2
ENSG00000198851	777.126239	0.967851599	1.95592574	0.00108104	0.01632848	CD3E
ENSG00000254302	10.8081836	1.195869318	2.29082828	0.0010954	0.01650377	NA
ENSG00000066279	799.723448	0.658467509	1.57840508	0.00109657	0.01650663	ASPM
ENSG00000141506	318.503096	0.972704271	1.9625158	0.0010988	0.01652908	PIK3R5
ENSG00000134508	534.252537	0.70985193	1.63563624	0.00110077	0.01654744	CABLES1
ENSG00000197614	7.55348173	1.472817352	2.77563401	0.00110152	0.01654745	MFAP5
ENSG00000226476	38.570169	1.250903705	2.37990454	0.00110442	0.01655568	LINC01748
ENSG00000180113	63.2126001	1.2003772	2.29799745	0.00110746	0.01655568	TDRD6
ENSG00000166813	48.4012446	1.169706558	2.24965935	0.00110347	0.01655568	KIF7
ENSG00000140368	103.69025	0.714026034	1.64037542	0.00110656	0.01655568	PSTPIP1
ENSG00000272049	7.06881279	0.978463126	1.9703653	0.001111	0.01657693	NA
ENSG00000064199	63.1552521	0.630292178	1.54787844	0.00112546	0.01670306	SPA17
ENSG00000153822	9.67940585	1.78651738	3.44981113	0.00112674	0.01670495	KCNJ16
ENSG00000243479	34.7313453	1.116075946	2.16756604	0.00112778	0.01670495	MNX1-AS1
ENSG00000221866	15.8661927	1.573790617	2.97685844	0.00113393	0.01674752	PLXNA4
ENSG00000102024	193.409669	1.270096066	2.41177625	0.0011345	0.01674752	PLS3
ENSG00000112394	79.8100276	0.75032197	1.6821682	0.00113365	0.01674752	SLC16A10
ENSG00000169397	224.197112	1.430224962	2.69488734	0.00113739	0.01676785	RNASE3
ENSG00000149571	15.3386126	0.853542963	1.80693294	0.00113957	0.01678869	KIRREL3
ENSG00000171860	59.3140235	0.658578521	1.57852654	0.00114727	0.01689103	C3AR1
ENSG00000259803	1132.58827	1.191295469	2.28357705	0.00115083	0.0169321	SLC22A31
ENSG00000177469	159.697195	0.885512024	1.84742017	0.00115453	0.01696405	CAVIN1
ENSG00000180178	63.8296281	1.021547739	2.0300957	0.00116693	0.0170742	NA
ENSG00000166845	96.6121932	0.763784681	1.69793906	0.00117171	0.01709633	C18orf54
ENSG00000186281	462.669921	0.688839564	1.61198639	0.00117111	0.01709633	GPAT2
ENSG00000198829	13.7020086	1.470776674	2.77171068	0.00117802	0.01716118	SUCNR1
ENSG00000197249	344.72744	0.89758343	1.86294287	0.00118097	0.01717057	SERPINA1
ENSG00000196372	114.048068	0.636327763	1.55436763	0.00117956	0.01717057	ASB13
ENSG00000236234	31.4526077	0.678800022	1.60080771	0.0011895	0.01722901	NA
ENSG00000227039	36.049724	1.143955687	2.20986109	0.0011966	0.01728392	ITGB2-AS1
ENSG00000258053	6.49264426	1.500068787	2.82856199	0.001203	0.01734242	NA
ENSG00000267795	49.4975333	1.15873765	2.23261989	0.00120839	0.01738617	SMIM22
ENSG00000149131	282.034242	0.917134468	1.88836084	0.00121265	0.01739051	SERPING1
ENSG00000214071	12.3569561	9.40804368	679.365281	0.00122351	0.01742262	LOC442445
ENSG00000256995	22.1174455	0.914915506	1.88545864	0.00123788	0.01757073	NA
ENSG00000157557	253.385297	0.837684058	1.7871789	0.00124906	0.01768415	ETS2
ENSG00000154864	211.207143	0.792616611	1.73221333	0.00125891	0.01778948	PIEZO2
ENSG00000254388	6.50872998	1.255669974	2.38778009	0.0012612	0.01781037	NA
ENSG00000183580	199.838508	1.36685488	2.57907706	0.00129925	0.01820846	FBXL7
ENSG00000198796	98.692164	1.346417107	2.54279844	0.0013103	0.01831705	ALPK2
ENSG00000118508	168.245168	0.82103766	1.76667622	0.00131503	0.01837148	RAB32
ENSG00000168546	44.9782975	1.253641969	2.38442593	0.00132039	0.01843461	GFRA2
ENSG00000260442	35.8377914	0.620913792	1.53784893	0.00132175	0.01844165	ATP2A1-AS1
ENSG00000206129	34.9601199	1.45454689	2.7407047	0.00137872	0.01899678	LOC642484
ENSG00000147246	199.532673	1.342579903	2.53604422	0.00137537	0.01899678	HTR2C
ENSG00000187144	14.5713252	0.950486528	1.93252426	0.00137953	0.01899678	SPATA21
ENSG00000267128	16.1392651	0.791005917	1.73028048	0.00137255	0.01899678	RNF157-AS1
ENSG00000139354	46.8640374	0.683998088	1.60658586	0.00137719	0.01899678	GAS2L3
ENSG00000185585	43.152304	0.992212506	1.98923333	0.00138407	0.01904755	OLFML2A
ENSG00000082397	122.763622	0.89189692	1.85561437	0.00139593	0.01910395	EPB41L3
ENSG00000178175	12.4987581	1.384852153	2.61145194	0.00139994	0.01910933	ZNF366
ENSG00000123201	19.2195362	1.155036633	2.22689978	0.00139862	0.01910933	GUCY1B2
ENSG00000185933	13.5659624	1.049526473	2.06985036	0.00140041	0.01910933	CALHM1
ENSG00000178662	32.9936262	1.482986987	2.79526873	0.00141993	0.01930367	CSRNP3
ENSG00000142512	53.0392644	0.775974837	1.7123467	0.00141887	0.01930367	SIGLEC10
ENSG00000140749	105.807722	0.669492029	1.59051285	0.00142296	0.0193186	IGSF6
ENSG00000104833	78.4159741	1.19533307	2.28997694	0.00142393	0.01931991	TUBB4A
ENSG00000138316	47.7051111	1.067083401	2.09519337	0.00144564	0.01953069	ADAMTS14

ENSG00000172794	523.883263	0.757950055	1.69108603	0.00145271	0.01958866	RAB37
ENSG00000152767	421.202033	0.612671481	1.52908805	0.00145347	0.01958866	FARP1
ENSG00000167613	243.986272	0.815489451	1.75989512	0.00145579	0.01960147	LAIR1
ENSG00000144852	44.4792197	0.713215172	1.63945371	0.00145879	0.0196022	NR1I2
ENSG00000130202	121.621838	0.836414963	1.78560746	0.00146089	0.01961701	NECTIN2
ENSG00000231528	8.45640575	1.850057371	3.60514521	0.00146687	0.01968535	FAM225A
ENSG00000114948	107.229555	1.395405629	2.63062504	0.00148925	0.01988934	ADAM23
ENSG00000170677	231.727606	0.860702977	1.81592293	0.00149568	0.01996312	SOCS6
ENSG00000246465	26.5046714	0.823307731	1.76945826	0.0015144	0.02016433	NA
ENSG00000111962	12.5280401	0.957910852	1.94249496	0.00152886	0.02030805	UST
ENSG00000150275	21.8577315	1.431637737	2.69752763	0.00153528	0.02038025	PCDH15
ENSG00000102466	178.208829	1.296828355	2.45688164	0.0015389	0.02038025	FGF14
ENSG00000172037	114.25202	0.880685936	1.84125052	0.0015375	0.02038025	LAMB2
ENSG00000137819	17.5928803	0.725238144	1.6531735	0.00156221	0.02059052	PAQR5
ENSG00000227674	69.1703658	0.662825877	1.58318064	0.00156157	0.02059052	LINC00355
ENSG00000011600	386.635095	0.874898888	1.83387955	0.00157091	0.02066822	TYROBP
ENSG00000178033	36.7035515	1.120158391	2.17370836	0.00157725	0.02069188	CALHM5
ENSG00000187678	97.2021596	0.998260517	1.99759002	0.00157792	0.02069188	SPRY4
ENSG00000093134	36.7615876	0.990371428	1.98669641	0.00157831	0.02069188	VNN3
ENSG00000254509	9.35960488	0.679939726	1.60207282	0.00158675	0.02077159	NA
ENSG00000147036	37.0714069	0.743074035	1.67373837	0.00161871	0.02109579	LANCL3
ENSG00000164237	183.413673	0.657746418	1.57761636	0.00162283	0.0211254	CMBL
ENSG00000140479	163.386974	0.9909629	1.98751108	0.00162777	0.02116486	PCSK6
ENSG00000119686	30.7569735	0.900886286	1.86721271	0.00164417	0.02136553	FLVCR2
ENSG00000101306	6.22665364	1.071709174	2.10192206	0.00164843	0.02139574	MYLK2
ENSG00000129353	949.264904	0.655855386	1.57554983	0.00165258	0.02143702	SLC44A2
ENSG00000236409	7.03317478	0.911085846	1.8804603	0.00165892	0.02148917	NA
ENSG00000006756	148.087278	0.628774203	1.54625065	0.00165804	0.02148917	ARSD
ENSG00000074181	59.6066302	0.818283245	1.76330647	0.00166954	0.02159381	NOTCH3
ENSG00000147437	28.032466	0.690799756	1.61417809	0.00167075	0.02159691	GNRH1
ENSG00000196526	92.8742777	0.782123211	1.71965983	0.00167891	0.02165497	AFAP1
ENSG00000014257	36.2726502	0.935902712	1.9130873	0.00168125	0.02166235	ACP3
ENSG00000189409	56.8280367	0.737994512	1.66785574	0.00169423	0.02177321	MMP23B
ENSG00000162747	263.017325	1.113935056	2.16435186	0.00169543	0.02177602	FCGR3B
ENSG00000130208	174.473384	0.937184909	1.91478832	0.0017112	0.02192776	APOC1
ENSG00000235568	121.244541	0.775122799	1.71133571	0.00171816	0.02196605	NFAM1
ENSG00000272281	63.924913	1.038992149	2.0547917	0.00172706	0.02204165	NA
ENSG00000164692	801.687051	0.915380298	1.88606618	0.00173162	0.02208717	COL1A2
ENSG00000197992	114.145917	1.590512364	3.01156284	0.00174388	0.02219234	CLEC9A
ENSG00000231419	39.0683805	1.169987012	2.25009671	0.00174293	0.02219234	LINC00689
ENSG00000231752	36.4774142	1.416793669	2.66991473	0.00175001	0.02224478	EMBP1
ENSG00000214429	12.7834444	1.324915127	2.50518149	0.00175983	0.02228015	NA
ENSG00000142347	421.815423	0.834341242	1.78304269	0.00175539	0.02228015	MYO1F
ENSG00000162458	28.7100491	0.723244179	1.65089021	0.00175736	0.02228015	FBLIM1
ENSG00000050555	12.4616259	1.384248492	2.61035947	0.00178349	0.02248966	LAMC3
ENSG00000121211	87.3510473	0.621420794	1.53838947	0.00178748	0.02252716	MND1
ENSG00000254221	43.5591337	0.825637923	1.77231854	0.00179222	0.02256123	PCDHGB1
ENSG00000267698	12.5825357	0.653578491	1.57306522	0.00179998	0.02260747	LOC101927572
ENSG00000181631	61.1349858	1.02333324	2.03260973	0.0018312	0.02296054	P2RY13
ENSG00000233379	124.533158	1.057040563	2.08065903	0.00183615	0.02299659	NA
ENSG00000117594	31.311086	1.134260691	2.19506048	0.00184742	0.02303621	HSD11B1
ENSG00000237978	26.4711804	0.965767482	1.95310225	0.00184936	0.02304463	NA
ENSG00000171621	141.321159	0.957197316	1.94153446	0.00186117	0.02315271	SPSB1
ENSG00000088992	130.681172	0.846794314	1.79850019	0.00186906	0.02319012	TESC
ENSG00000226465	8.33435814	1.117591445	2.16984418	0.00188982	0.02339835	NA
ENSG00000090534	9.79578226	6.573364827	95.2313598	0.00189609	0.02345542	THPO
ENSG00000024526	249.897193	0.729357379	1.65790045	0.00190533	0.02353021	DEPDC1
ENSG00000189064	275.526806	2.41751769	5.34250995	0.00192167	0.02369237	GAGE2B
ENSG00000105352	12.5140305	1.340881085	2.53305971	0.00193787	0.02382464	CEACAM4
ENSG00000138771	1308.91506	1.001515803	2.00210245	0.00194215	0.0238387	SHROOM3
ENSG00000124839	27.5336679	1.816772996	3.52292313	0.00195624	0.02394527	RAB17
ENSG00000120669	107.946717	0.888679791	1.85148106	0.00196802	0.02404962	SOHLH2
ENSG00000158163	8.66407618	1.292019927	2.44870661	0.00197074	0.02406951	DZIP1L
ENSG00000106823	24.7729393	1.003152149	2.00437458	0.0020021	0.02427852	ECM2
ENSG00000170190	45.7291045	0.704840179	1.62996409	0.00201396	0.02434237	SLC16A5
ENSG00000189229	30.5294586	0.978717054	1.97071213	0.00201842	0.02438287	NA
ENSG00000143842	24.3459049	0.921849183	1.89454208	0.00202464	0.02443135	SOX13
ENSG00000154654	217.800037	0.894654883	1.85916509	0.00203009	0.02447045	NCAM2
ENSG00000257335	183.68912	0.918605026	1.89028665	0.0020421	0.02448666	MGAM
ENSG00000196549	286.482409	0.83992268	1.78995421	0.00203927	0.02448666	MME
ENSG00000158402	95.2361965	0.632460881	1.55020701	0.00203734	0.02448666	CDC25C
ENSG00000102780	239.581721	0.688410549	1.61150711	0.00205155	0.02456869	DGKH
ENSG00000170390	15.1642378	1.048808347	2.06882032	0.00206633	0.0246923	DCLK2
ENSG00000127990	263.994168	1.047009519	2.06624241	0.00206852	0.02470512	SGCE
ENSG00000158715	23.7919108	0.715158878	1.641664	0.00208934	0.02488662	SLC45A3
ENSG00000134940	6.20875848	0.996032702	1.99450771	0.00209969	0.02496958	ACRV1
ENSG00000160360	62.1650254	0.822382079	1.76832332	0.00209935	0.02496958	GPSM1
ENSG00000144677	95.0452322	0.936007664	1.91322648	0.00210708	0.02503058	CTDSPL
ENSG00000228065	44.4045382	0.830526136	1.77833378	0.00213616	0.0253353	NA
ENSG00000242498	43.1421767	0.700943266	1.62556728	0.00214696	0.02540896	ARPIN
ENSG00000230257	30.3489074	1.54443035	2.91688872	0.00214882	0.02541732	NFE4
ENSG00000147381	179.122199	2.290608845	4.89262546	0.00215712	0.02546213	MAGEA4
ENSG00000103534	44.7247202	0.994490834	1.99237724	0.00215464	0.02546213	TMC5
ENSG00000135046	4804.37416	0.81052452	1.75384897	0.00217136	0.02554752	ANXA1
ENSG00000140932	26.338959	0.885285531	1.84713016	0.00217661	0.02559564	CMTM2
ENSG00000197472	14.2895359	1.166693602	2.24496601	0.00219305	0.0257207	ZNF695
ENSG00000101938	140.22981	1.018089122	2.02523472	0.00221244	0.0258956	CHRDL1
ENSG00000148468	819.05486	0.828353938	1.77565824	0.00221381	0.0258956	FAM171A1
ENSG00000150893	31.6088547	1.548011185	2.92413757	0.00221623	0.0259102	FREM2
ENSG00000158292	21.9602783	0.840333539	1.79046403	0.00225683	0.02627388	GPR153
ENSG00000189023	82.588975	1.573362365	2.97597491	0.00227869	0.02645884	MAGEB16
ENSG00000248672	295.375717	1.408091452	2.6538585	0.00229032	0.02655212	LY75-CD302
ENSG00000143079	36.3827789	0.904968412	1.8725035	0.00230298	0.02665707	CTTNBP2NL
ENSG00000198502	445.075982	0.834288062	1.78297696	0.00231736	0.0267465	HLA-DRB5
ENSG00000248302	44.3898723	1.066378008	2.0941692	0.00233436	0.02686571	NA
ENSG00000143341	44.8185272	1.387406138	2.61607905	0.00233655	0.02687703	HMCN1
ENSG00000112280	9.33987872	1.359708842	2.56633382	0.00235363	0.02704532	COL9A1
ENSG00000163814	18.8622295	0.948484537	1.92984441	0.00235821	0.02705593	CDCP1
ENSG00000269994	48.3757823	1.105470961	2.15169108	0.00236066	0.02706024	LOC440173
ENSG00000162894	793.845262	0.825393755	1.77201861	0.00237032	0.02714662	FCMR
ENSG00000259316	64.3087675	0.83957521	1.78952315	0.00238638	0.02728277	NA
ENSG00000007350	19.3382708	1.49668099	2.82192763	0.00239173	0.02730802	TKTL1
ENSG00000182732	19.1753857	1.23593515	2.3553397	0.00240155	0.02736881	RGS6
ENSG00000131378	202.30806	0.615151707	1.53171906	0.00240119	0.02736881	RFTN1
ENSG00000233901	19.1583789	0.944567518	1.92461185	0.00241075	0.02744532	LINC01503
ENSG00000111344	104.796541	1.098872641	2.14187256	0.00241837	0.02750387	RASAL1
ENSG00000101336	197.252705	0.803071447	1.74481182	0.00243212	0.02760352	HCK
ENSG00000153283	63.4690062	0.961305963	1.94707163	0.00243547	0.02761323	CD96
ENSG00000255983	15.8130126	1.296629955	2.45654379	0.00246571	0.02789642	NA
ENSG00000164251	11.7212248	1.221990971	2.33268414	0.00246675	0.02789642	F2RL1
ENSG00000236675	20.1468536	0.749884295	1.68165796	0.00247603	0.02798707	MTX1P1
ENSG00000251226	29.2270249	1.193936502	2.28776125	0.00248632	0.02802352	LINC02714
ENSG00000177689	12.2655025	1.184844288	2.27338856	0.00248427	0.02802352	MAGEB10
ENSG00000174611	32.3345184	0.923769382	1.89706536	0.00249221	0.02802352	KY
ENSG00000109072	13.8013652	0.871573007	1.82965673	0.00250832	0.02810815	VTN
ENSG00000110042	178.809441	0.657522298	1.5773713	0.00253129	0.02832253	DTX4
ENSG00000187164	291.046355	0.692734797	1.61634459	0.00254995	0.0284873	SHTN1
ENSG00000059804	1346.1153	0.819176727	1.76439885	0.00262304	0.02911376	SLC2A3
ENSG00000151025	17.9743156	1.456719047	2.74483428	0.00262949	0.02915616	GPR158
ENSG00000134769	98.7440536	0.767198168	1.70196122	0.00262842	0.02915616	DTNA
ENSG00000121594	9.99211642	0.960950471	1.94659192	0.00264331	0.02925076	CD80
ENSG00000034053	122.826148	0.943925244	1.92375522	0.00264472	0.02925179	APBA2
ENSG00000124260	81.1352927	1.241999191	2.36526067	0.00266516	0.02942264	MAGEA10
ENSG00000267426	67.2152364	0.641890817	1.56037287	0.0026728	0.0294593	NA
ENSG00000171659	12.5959007	1.055936915	2.07906796	0.00270123	0.02963995	GPR34
ENSG00000100344	7.32846189	1.077910042	2.1109758	0.00276809	0.03015317	PNPLA3
ENSG00000132170	38.205066	0.86132027	1.81670009	0.00281574	0.03056323	PPARG
ENSG00000173068	106.497589	0.927130654	1.9014904	0.00282528	0.03064046	BNC2

ENSG00000232677	294.522922	0.741943205	1.67242696	0.00285188	0.03084966	LINC00665
ENSG00000167695	38.6353991	0.643976392	1.56263019	0.00285151	0.03084966	TLCD3A
ENSG00000168959	26.5810343	1.145171236	2.2117238	0.00285669	0.03088529	GRM5
ENSG00000109089	24.2294865	0.848915879	1.80114693	0.00286357	0.03091572	CDR2L
ENSG00000049130	25.0833188	1.138243447	2.20112862	0.00286697	0.03092225	KITLG
ENSG00000164796	25.3466641	1.061120347	2.08655124	0.00288348	0.03106997	CSMD3
ENSG00000100079	10.4149915	1.575021029	2.97939835	0.00291586	0.0313351	LGALS2
ENSG00000215270	19.9748727	1.027165365	2.03801598	0.00292813	0.03141441	NA
ENSG00000134365	6.14154373	1.718373872	3.29065293	0.00294794	0.03156493	CFHR4
ENSG00000131650	35.7091051	0.883383253	1.84469622	0.00298245	0.03179746	KREMEN2
ENSG00000126778	38.6727621	0.949207199	1.93081133	0.00299891	0.03187672	SIX1
ENSG00000134222	57.4879722	0.663473417	1.5838914	0.00299739	0.03187672	PSRC1
ENSG00000151948	47.406305	0.875084611	1.83411565	0.00300751	0.03192111	GLT1D1
ENSG00000143546	2958.55493	0.905714409	1.87347199	0.00303705	0.0321767	S100A8
ENSG00000196668	67.6242938	0.778184398	1.71497126	0.0030459	0.03223969	LINC00173
ENSG00000156509	45.7858401	0.686528691	1.60940642	0.00305559	0.0323113	FBXO43
ENSG00000146070	76.3288759	1.06445293	2.09137668	0.00309795	0.0325726	PLA2G7
ENSG00000180891	48.8726415	0.958639319	1.94347604	0.00312378	0.03278188	CUEDC1
ENSG00000250102	22.5733702	1.469650138	2.76954722	0.00313484	0.03286679	NA
ENSG00000169550	58.6894194	1.788632991	3.45487375	0.00315448	0.03300608	MUC15
ENSG00000197933	73.6016582	0.660944173	1.58111705	0.00315307	0.03300608	ZNF823
ENSG00000204282	444.910581	0.78315611	1.72089146	0.00318845	0.03327138	NA
ENSG00000161642	103.455902	0.703205796	1.6281186	0.00318628	0.03327138	ZNF385A
ENSG00000108511	8.28005297	1.234669093	2.35327365	0.00321652	0.03351688	HOXB6
ENSG00000263711	131.226193	1.265668815	2.40438648	0.00321959	0.03353307	LINC02864
ENSG00000105376	168.120666	0.758784013	1.69206385	0.00322448	0.03356826	ICAM5
ENSG00000248810	35.3836261	1.564717051	2.95819478	0.00322895	0.03359895	LINC02432
ENSG00000165949	2809.88969	0.826116567	1.77290664	0.00323857	0.03365163	IFI27
ENSG00000148942	41.7249887	0.862041608	1.81760865	0.0032477	0.03368924	SLC5A12
ENSG00000112319	142.403262	1.46869799	2.76771999	0.00325317	0.03369264	EYA4
ENSG00000108691	76.8445757	1.185603573	2.27458535	0.00325241	0.03369264	CCL2
ENSG00000143776	178.974432	0.891745661	1.85541982	0.00326369	0.03377005	CDC42BPA
ENSG00000184730	149.803873	0.662319084	1.5826246	0.00326658	0.0337842	APOBR
ENSG00000174705	41.0944054	0.825929626	1.77267693	0.003273	0.03380316	SH3PXD2B
ENSG00000204624	19.2963375	1.075838979	2.10794756	0.00332731	0.03426818	DISP3
ENSG00000182798	19.0733606	1.213765926	2.31942296	0.00333182	0.03429872	MAGEB17
ENSG00000138180	314.176897	0.621473989	1.5384462	0.00333391	0.03430287	CEP55
ENSG00000077522	17.1607008	2.118072424	4.34113541	0.00336302	0.03448266	ACTN2
ENSG00000152076	20.002259	0.89040266	1.85369342	0.00336128	0.03448266	CCDC74B
ENSG00000245848	95.5128909	0.816355182	1.76095151	0.00336821	0.0344969	CEBPA
ENSG00000261669	12.3358144	0.863851417	1.81989021	0.00339557	0.03466487	NA
ENSG00000155980	48.5043999	0.730929116	1.65970762	0.00341769	0.03477844	KIF5A
ENSG00000183690	20.8560833	1.126581722	2.18340797	0.00344866	0.03496684	EFHC2
ENSG00000151892	30.871278	1.316827733	2.49117735	0.0034641	0.03507345	GFRA1
ENSG00000172346	27.4754322	1.001306851	2.0018125	0.00348847	0.03523962	CSDC2
ENSG00000137462	100.418119	0.737615016	1.66741708	0.00351052	0.03542509	TLR2
ENSG00000164078	24.1645662	0.790182219	1.72929287	0.00357731	0.03587513	MST1R
ENSG00000173621	136.633274	0.661559475	1.58179153	0.00357903	0.03587619	LRFN4
ENSG00000121933	37.2701461	0.770021286	1.70529494	0.00358226	0.03589234	TMIGD3
ENSG00000271127	39.3075017	1.464854348	2.76035601	0.00359099	0.03594719	NA
ENSG00000232977	8.49389567	0.892806347	1.85678445	0.00359635	0.03598463	LINC00327
ENSG00000162692	806.211691	0.949087688	1.93065139	0.00360165	0.03602141	VCAM1
ENSG00000205212	53.4192587	1.526488988	2.8808389	0.0036093	0.036049	CCDC144NL
ENSG00000234409	9.64858646	0.800924853	1.74221763	0.00362296	0.0361167	CCDC188
ENSG00000113721	91.2086508	0.687246515	1.61020739	0.00361926	0.0361167	PDGFRB
ENSG00000103196	65.6618448	0.75623764	1.68907998	0.00365375	0.03637814	CRISPLD2
ENSG00000060718	63.3300905	1.100526963	2.14433003	0.00368911	0.03661495	COL11A1
ENSG00000109674	42.6026194	0.790487169	1.72965843	0.00369712	0.03667805	NEIL3
ENSG00000230426	8.30400887	1.161833235	2.23741556	0.00370557	0.0367333	LINC01036
ENSG00000204681	334.187439	0.698758303	1.62310722	0.00370601	0.0367333	GABBR1
ENSG00000115705	161.780592	1.349429776	2.54811392	0.00371202	0.03677639	TPO
ENSG00000165092	83.1295431	0.834814278	1.78362741	0.00371457	0.03678522	ALDH1A1
ENSG00000158286	704.405208	0.643899312	1.5625467	0.00372147	0.03683705	RNF207
ENSG00000224141	17.79052	0.848491486	1.80061718	0.00373118	0.03690015	MIR548XHG
ENSG00000104044	40.1652257	0.996309223	1.99489004	0.00377208	0.03718843	OCA2
ENSG00000230453	18.0412519	0.945202174	1.92545869	0.00378704	0.03730268	ANKRD18B
ENSG00000070601	13.8747632	0.922673376	1.89562472	0.00379207	0.03731898	FRMPD1
ENSG00000135218	269.9595	0.813749823	1.75777428	0.00380049	0.0373853	CD36
ENSG00000174945	27.9081134	0.769872517	1.70511911	0.00380836	0.03744608	AMZ1
ENSG00000008516	178.150356	0.856304617	1.81039514	0.00381015	0.03744701	MMP25
ENSG00000120498	20.721956	1.608643418	3.04964945	0.0038215	0.03752529	TEX11
ENSG00000086289	220.712484	0.766417161	1.70104011	0.00383698	0.03762724	EPDR1
ENSG00000149212	144.636481	0.648409565	1.56743929	0.00384466	0.03763585	SESN3
ENSG00000133454	289.039957	1.312186482	2.48317594	0.00384894	0.03766109	MYO18B
ENSG00000167600	9.19521445	1.458600664	2.74841653	0.0038518	0.03767243	CYP2S1
ENSG00000226942	9.05104473	0.596188268	1.51171719	0.0038864	0.03792709	NA
ENSG00000119535	593.865135	0.816123615	1.76066888	0.0039221	0.03820811	CSF3R
ENSG00000143195	269.312544	0.998001885	1.99723194	0.00396136	0.03838788	ILDR2
ENSG00000058335	48.7325049	0.780327897	1.71752119	0.00398394	0.03858731	RASGRF1
ENSG00000232564	15.0106823	0.743372229	1.67408436	0.00399186	0.03863277	MRTFA-AS1
ENSG00000267765	11.335642	1.055387693	2.07827662	0.00400244	0.03869984	NA
ENSG00000154146	163.257609	0.742848546	1.67347679	0.00400403	0.03869984	NRGN
ENSG00000167984	203.51854	0.65811296	1.57801723	0.00400165	0.03869984	NLRC3
ENSG00000196335	81.0653186	0.702296418	1.62709267	0.0040137	0.03877638	STK31
ENSG00000197629	1088.62526	0.716218739	1.64287047	0.00407156	0.03916448	MPEG1
ENSG00000223401	7.62911426	1.050137799	2.07072762	0.00408284	0.03922188	NA
ENSG00000133216	51.0056845	0.75082664	1.68275675	0.00414969	0.03967471	EPHB2
ENSG00000168702	19.8431029	1.871983687	3.66035528	0.00417383	0.03985387	LRP1B
ENSG00000251442	10.1013169	0.934302333	1.9109663	0.00424592	0.04031621	NA
ENSG00000214248	26.7860371	0.678854042	1.60086765	0.0043301	0.04093995	NA
ENSG00000238243	13.5058364	1.491150794	2.81113121	0.0043365	0.04098294	OR2W3
ENSG00000176659	14.9222012	1.145233574	2.21181937	0.00437173	0.04121037	C20orf197
ENSG00000164066	337.967186	0.612454576	1.52885817	0.00437861	0.0412401	INTU
ENSG00000156966	39.1239548	0.651013703	1.57027115	0.00441352	0.04151596	B3GNT7
ENSG00000162888	17.3562319	0.707792861	1.63330346	0.00441851	0.04154525	NA
ENSG00000121005	41.5672425	1.097057296	2.13917913	0.0044802	0.04196819	CRISPLD1
ENSG00000101425	147.09159	0.925783693	1.89971592	0.00448725	0.0419954	BPI
ENSG00000230747	6.28732708	1.261557123	2.39754372	0.00454141	0.04235911	NA
ENSG00000149380	10.4319033	0.700316528	1.62486125	0.00455689	0.04243201	P4HA3
ENSG00000265293	12.4419843	0.753104247	1.68541544	0.00457248	0.04255002	NA
ENSG00000224397	30.5056684	0.893601401	1.85780799	0.00458603	0.04260853	PELATON
ENSG00000248131	20.5147217	1.2696745	2.41107161	0.00459773	0.04263296	NA
ENSG00000166352	163.090763	0.678406273	1.60037087	0.00460048	0.04264066	IFTAP
ENSG00000150764	271.384304	0.621245043	1.53820207	0.00469269	0.04329593	DIXDC1
ENSG00000163220	4637.83074	0.877897664	1.83769541	0.00469772	0.04331347	S100A9
ENSG00000248668	14.3232707	0.680558111	1.60275967	0.00475847	0.04373879	OXCT1-AS1
ENSG00000250038	51.1820868	0.950353172	1.93234564	0.00480275	0.04409091	NA
ENSG00000177138	31.0979027	1.175982691	2.25946732	0.0048089	0.04411078	FAM9B
ENSG00000137878	59.9403273	1.022359244	2.03123793	0.00482656	0.04419953	GCOM1
ENSG00000269834	78.2245998	0.647484762	1.56643484	0.00487246	0.04439956	ZNF528-AS1
ENSG00000225465	22.1716314	0.737385375	1.66715169	0.0048818	0.04442764	NA
ENSG00000163435	336.072808	0.651269699	1.57054981	0.00488357	0.04442764	ELF3
ENSG00000164047	190.425988	1.065252517	2.09253611	0.00491565	0.04462784	CAMP
ENSG00000076706	332.794254	0.684668982	1.60733315	0.00493054	0.04472635	MCAM
ENSG00000184343	82.1127046	0.602821467	1.51868374	0.00492869	0.04472635	SRPK3
ENSG00000102837	89.2837556	1.034719203	2.04871486	0.00494293	0.04479513	OLFM4
ENSG00000273143	93.8789956	0.640812029	1.55920652	0.00494622	0.04479513	NA
ENSG00000124795	404.27686	0.585495148	1.50055391	0.00496463	0.04488841	DEK
ENSG00000167751	42.4266581	0.635367634	1.55333353	0.00498726	0.04505628	KLK2
ENSG00000126733	10.0169345	0.904904126	1.87242006	0.00501004	0.04515526	DACH2
ENSG00000241418	6.60123463	0.807918987	1.75068435	0.00502349	0.04521034	NA
ENSG00000077327	152.407243	0.761249033	1.69495742	0.00503263	0.04522627	SPAG6
ENSG00000237510	49.931888	1.706300285	3.26322912	0.00505408	0.04534546	GPAT2P1
ENSG00000224309	48.1963448	1.04094948	2.05758136	0.00510405	0.04570116	NA
ENSG00000100122	66.1840048	0.653117221	1.57256235	0.00510898	0.04572683	CRYBB1
ENSG00000177706	48.4061563	0.941881541	1.92103199	0.00522	0.04649343	FAM20C
ENSG00000255221	9.61253702	1.263668356	2.40105484	0.00523978	0.04654607	CARD17
ENSG00000103528	12.7258824	1.185009815	2.27364942	0.00523713	0.04654607	SYT17

ENSG00000158825	74.8636096	0.848987702	1.80123661	0.00524255	0.04654607	CDA
ENSG00000244482	57.7015239	0.64849329	1.56753026	0.00524214	0.04654607	LILRA6
ENSG00000146166	45.2120844	0.940828084	1.91962976	0.00525902	0.04667351	LGSN
ENSG00000159708	6.34923055	0.893666731	1.85789212	0.00529537	0.04692243	LRRC36
ENSG00000251357	11.225128	2.097694997	4.2802498	0.00532406	0.04708092	NA
ENSG00000229111	9.08691605	0.785350798	1.72351135	0.00532878	0.04710387	MED4-AS1
ENSG00000182885	124.661645	1.023414943	2.03272485	0.00533254	0.04711822	ADGRG3
ENSG00000133687	275.112669	0.661368042	1.58158165	0.00533892	0.04711822	TMTC1
ENSG00000198125	39.7719153	1.72436476	3.304346	0.00534642	0.04714763	MB
ENSG00000120594	250.118283	0.618557174	1.53533893	0.00534651	0.04714763	PLXDC2
ENSG00000265393	6.93016214	0.762198684	1.69607349	0.00535021	0.04716149	NA
ENSG00000125726	72.5969171	1.094136927	2.13485329	0.00535688	0.04720155	CD70
ENSG00000203435	7.4665633	0.80768377	1.75039894	0.00537032	0.04726353	NA
ENSG00000123080	1218.13888	0.774307118	1.71036842	0.00539401	0.04737782	CDKN2C
ENSG00000138829	83.8623134	0.862058867	1.8176304	0.00539878	0.04738213	FBN2
ENSG00000211898	46675.9414	0.976534798	1.96773344	0.00541096	0.04745468	IGHD
ENSG00000171617	410.561325	0.670902789	1.59206892	0.00541651	0.04746252	ENC1
ENSG00000251322	44.8473727	0.934833348	1.9116698	0.00542598	0.04752667	SHANK3
ENSG00000087253	175.924761	0.717791612	1.64466256	0.00545307	0.04765085	LPCAT2
ENSG00000172594	94.900495	0.80605479	1.74842364	0.00548782	0.04783574	SMPDL3A
ENSG00000136929	145.089322	0.73746992	1.66724939	0.00548747	0.04783574	HEMGN
ENSG00000131400	94.5892603	0.639214901	1.55748136	0.00550601	0.04791867	NAPSA
ENSG00000180871	80.1440894	0.9888988	1.98466953	0.00557083	0.04822318	CXCR2
ENSG00000175294	12.8968145	1.004353197	2.00604393	0.00560207	0.04841788	CATSPER1
ENSG00000138162	68.4460591	0.815054829	1.75936501	0.00563021	0.04862318	TACC2
ENSG00000152953	6.7491044	1.2031134	2.30235995	0.00568671	0.04903459	STK32B
ENSG00000242574	375.531932	0.763041046	1.69706409	0.00578881	0.04968277	HLA-DMB
ENSG00000165171	55.4425814	0.82657875	1.7734747	0.00581206	0.04980512	METTL27
ENSG00000099284	24.5422031	0.65723551	1.57705777	0.00581562	0.04981635	MACROH2A2

ENSG00000182158	26826.5513	-0.749224216	0.59492338	1.06E-10	1.81E-07	CREB3L2
ENSG00000125675	148.814963	-3.013068248	0.12387284	1.47E-09	1.12E-06	GRIA3
ENSG00000166073	400.499208	-1.327405139	0.39848432	3.41E-09	1.94E-06	GPR176
ENSG00000170017	2736.31799	-1.142061147	0.45311176	2.24E-08	8.40E-06	ALCAM
ENSG00000121966	23028.8756	-1.24868062	0.42083289	4.28E-08	1.42E-05	CXCR4
ENSG00000109321	4494.20414	-1.450851586	0.36580543	4.65E-08	1.47E-05	AREG
ENSG00000178764	2508.01873	-0.667431988	0.62962643	5.94E-08	1.69E-05	ZHX2
ENSG00000150051	979.882346	-1.363469334	0.38864656	6.28E-08	1.76E-05	MKX
ENSG00000128590	14585.5339	-0.68032235	0.62402583	8.15E-08	2.12E-05	DNAJB9
ENSG00000164442	14898.8601	-0.675085475	0.62629511	1.21E-07	2.77E-05	CITED2
ENSG00000196376	305.483546	-1.539537669	0.34399567	1.21E-07	2.77E-05	SLC35F1
ENSG00000171105	6788.40061	-0.587627061	0.66543651	1.24E-07	2.78E-05	INSR
ENSG00000162772	8607.24639	-1.142933839	0.45283776	1.27E-07	2.80E-05	ATF3
ENSG00000095110	121.996186	-1.576532514	0.33528678	2.68E-07	4.86E-05	NXPE1
ENSG00000260368	52.7683534	-0.831481102	0.56195203	3.25E-07	5.71E-05	NA
ENSG00000260916	19217.4794	-0.60728174	0.65643236	3.57E-07	6.08E-05	CCPG1
ENSG00000261652	160.333999	-0.843394349	0.55733075	4.73E-07	7.33E-05	C15orf65
ENSG00000137634	718.713546	-1.545844583	0.34249514	5.90E-07	8.16E-05	NXPE4
ENSG00000216863	53.5501793	-1.509224309	0.35130005	6.89E-07	9.25E-05	LY86-AS1
ENSG00000135063	50.5195023	-1.902438291	0.2674909	6.99E-07	9.32E-05	FAM189A2
ENSG00000115963	1538.19112	-1.430319194	0.37104879	1.00E-06	0.00012151	RND3
ENSG00000157514	74626.2322	-0.757155217	0.59166185	1.04E-06	0.00012499	TSC22D3
ENSG00000172818	140.98376	-1.702943973	0.30715867	1.07E-06	0.00012694	OVOL1
ENSG00000157404	1239.86167	-1.16910148	0.44469822	1.63E-06	0.00017105	KIT
ENSG00000072694	6561.46213	-1.10180045	0.46593466	1.67E-06	0.00017362	FCGR2B
ENSG00000164691	5320.71545	-0.72654096	0.60435118	1.93E-06	0.00019448	TAGAP
ENSG00000109787	4280.08509	-0.659392398	0.63314489	2.21E-06	0.00021215	KLF3
ENSG00000153208	1838.21046	-0.888820597	0.54005543	2.30E-06	0.00021833	MERTK
ENSG00000164440	651.403245	-0.776738505	0.58368484	2.32E-06	0.00021889	TXLNB
ENSG00000159958	928.468395	-1.038319714	0.48689422	2.34E-06	0.00021991	TNFRSF13C
ENSG00000114737	762.026837	-1.03817875	0.4869418	2.58E-06	0.00023433	CISH
ENSG00000171132	800.241732	-0.919993116	0.52851154	2.87E-06	0.00025313	PRKCE
ENSG00000082438	2536.85323	-0.844726951	0.55681618	2.92E-06	0.00025663	COBLL1
ENSG00000148841	5239.45615	-0.926489365	0.52613708	2.96E-06	0.00025916	ITPRIP
ENSG00000152078	832.588785	-0.776692517	0.58370344	3.41E-06	0.00028681	TLCD4
ENSG00000166432	2307.03852	-0.808008864	0.57116961	4.48E-06	0.00034701	ZMAT1
ENSG00000172086	987.74512	-0.6279518	0.64709445	4.57E-06	0.00035148	KRCC1
ENSG00000167077	8342.54564	-0.760808857	0.59016536	5.14E-06	0.00038696	MEI1
ENSG00000123358	10445.8254	-1.129371554	0.4571148	5.24E-06	0.0003931	NR4A1
ENSG00000178977	233.245383	-0.709960651	0.61133681	5.42E-06	0.00039857	LINC00324
ENSG00000061455	36.4720734	-1.27938545	0.41197096	6.05E-06	0.00043888	PRDM6
ENSG00000170011	180.920299	-1.51558745	0.34975402	6.50E-06	0.000454	MYRIP
ENSG00000245468	26.4305976	-0.820275401	0.56633382	6.88E-06	0.00047575	NA
ENSG00000151623	166.123059	-1.087549938	0.47055983	7.12E-06	0.00048421	NR3C2
ENSG00000125740	97278.6198	-0.882683091	0.54235783	7.39E-06	0.00049869	FOSB
ENSG00000155465	1537.81509	-0.686779803	0.62123895	7.63E-06	0.0005086	SLC7A7
ENSG00000164142	127.929477	-1.519378517	0.34883616	8.10E-06	0.00052723	FAM160A1
ENSG00000230923	17.3422378	-1.131549511	0.45642524	8.34E-06	0.0005371	LINC00309
ENSG00000259884	84.9636932	-1.54912936	0.34171622	8.33E-06	0.0005371	NA
ENSG00000157680	228.067482	-1.022201226	0.49236454	8.52E-06	0.00054327	DGKI
ENSG00000187699	2532.70982	-0.61486979	0.65298883	8.71E-06	0.00055117	C2orf88
ENSG00000163297	1298.59545	-0.687709803	0.62083861	8.69E-06	0.00055117	ANTXR2
ENSG00000073737	1120.63177	-1.317215499	0.40130875	9.78E-06	0.00060993	DHRS9
ENSG00000123094	785.360648	-0.603242703	0.65827271	1.09E-05	0.00065703	RASSF8
ENSG00000120051	19.5891384	-1.315877612	0.40168107	1.14E-05	0.00067787	CFAP58
ENSG00000185633	192.608205	-0.678900531	0.62464113	1.42E-05	0.00079761	NDUFA4L2
ENSG00000151491	356.400788	-0.634083613	0.64434997	1.91E-05	0.00100692	EPS8
ENSG00000170345	78412.2964	-0.739837855	0.59880665	1.91E-05	0.00100692	FOS
ENSG00000133878	713.2314	-0.985366009	0.50509756	1.93E-05	0.00101424	DUSP26
ENSG00000150347	7971.02577	-0.919768352	0.52859389	2.04E-05	0.00105164	ARID5B
ENSG00000162771	147.125298	-1.244086008	0.42217527	2.06E-05	0.00105756	FAM71A
ENSG00000162415	129.773966	-0.835906913	0.56023075	2.10E-05	0.00106155	ZSWIM5
ENSG00000035720	1225.76793	-0.867853533	0.54796151	2.23E-05	0.00110673	STAP1
ENSG00000230709	231.094862	-1.046200938	0.48424165	2.27E-05	0.00111851	LOC284191
ENSG00000198948	2535.46235	-0.957321941	0.51501204	2.65E-05	0.00123731	MFAP3L
ENSG00000153162	9118.39732	-0.586329279	0.66603538	2.78E-05	0.00127437	BMP6
ENSG00000200087	91.000121	-0.863263378	0.54970771	2.79E-05	0.00127437	SNORA73B
ENSG00000129422	2189.75355	-0.892811835	0.53856343	2.84E-05	0.00128852	MTUS1
ENSG00000203709	7019.14437	-0.632387636	0.64510789	3.58E-05	0.00152822	NA
ENSG00000120129	47391.4595	-0.761405979	0.58992114	3.60E-05	0.00152922	DUSP1
ENSG00000027697	2912.30012	-0.644721502	0.63961625	3.87E-05	0.00161983	IFNGR1
ENSG00000258792	13.7662095	-1.091708647	0.46920535	4.18E-05	0.00170231	LOC105370616
ENSG00000271913	177.781149	-0.698247521	0.61632041	4.33E-05	0.00171805	LOC105378083
ENSG00000091181	1737.83001	-0.727435577	0.60397654	4.29E-05	0.00171805	IL5RA
ENSG00000122787	58.4364642	-1.004228303	0.49853673	4.31E-05	0.00171805	AKR1D1
ENSG00000144645	1836.97486	-0.747900887	0.59546933	4.37E-05	0.00172397	OSBPL10
ENSG00000111859	2768.72774	-0.612193976	0.65420107	4.64E-05	0.00181157	NEDD9
ENSG00000147408	2367.14661	-0.590566692	0.664082	4.77E-05	0.00184902	CSGALNACT1
ENSG00000196468	81.9909237	-1.692646695	0.30935887	4.83E-05	0.00186007	FGF16
ENSG00000173198	2270.34017	-0.600426454	0.65955896	5.15E-05	0.00194033	CYSLTR1
ENSG00000197776	164.307361	-0.628164346	0.64699912	5.26E-05	0.00196079	KLHDC1
ENSG00000126952	11.5720856	-1.412810522	0.37557931	5.56E-05	0.00204127	NXF5
ENSG00000152939	228.800352	-0.611976824	0.65429955	5.65E-05	0.00205308	MARVELD2
ENSG00000249141	73.9897814	-0.840215329	0.55856019	6.05E-05	0.00216675	NA
ENSG00000164949	479.190684	-1.154081308	0.44935224	6.16E-05	0.00219156	GEM
ENSG00000253690	42.9417017	-0.956052232	0.5154655	6.17E-05	0.00219187	NA
ENSG00000170745	1325.21655	-1.229243715	0.42654099	6.27E-05	0.00221952	KCNS3
ENSG00000204290	18.3937148	-0.961024156	0.51369212	6.65E-05	0.00231104	BTNL2
ENSG00000169136	8514.10172	-0.617347187	0.65186848	6.83E-05	0.00235676	ATF5
ENSG00000165312	12374.9113	-0.764449645	0.58867789	7.11E-05	0.00242285	OTUD1
ENSG00000184545	367.926383	-1.096816292	0.46754713	7.47E-05	0.00251824	DUSP8
ENSG00000078081	2645.72349	-0.719586865	0.60727132	8.02E-05	0.0026635	LAMP3
ENSG00000267469	31.4533392	-3.702170157	0.07683087	8.02E-05	0.0026635	NA
ENSG00000117090	4978.5178	-1.057088351	0.48060103	8.29E-05	0.00274048	SLAMF1
ENSG00000237211	15.2489092	-1.589779864	0.33222214	8.46E-05	0.00277699	NA
ENSG00000249741	68.1424952	-0.694344244	0.61799015	8.58E-05	0.00278265	NA
ENSG00000199270	15.5741881	-1.43805708	0.369064	8.76E-05	0.00278265	RNA5S12
ENSG00000199334	15.5741881	-1.43805708	0.369064	8.76E-05	0.00278265	RNA5S11
ENSG00000199337	15.5741881	-1.43805708	0.369064	8.76E-05	0.00278265	RNA5S3

ENSG00000199352	15.5741881	-1.43805708	0.369064	8.76E-05	0.00278265	RNA5S1
ENSG00000199396	15.5741881	-1.43805708	0.369064	8.76E-05	0.00278265	RNA5S5
ENSG00000199910	15.5741881	-1.43805708	0.369064	8.76E-05	0.00278265	RNA5S10
ENSG00000200343	15.5741881	-1.43805708	0.369064	8.76E-05	0.00278265	RNA5S8
ENSG00000200370	15.5741881	-1.43805708	0.369064	8.76E-05	0.00278265	RNA5S17
ENSG00000200381	15.5741881	-1.43805708	0.369064	8.76E-05	0.00278265	RNA5S4
ENSG00000200624	15.5741881	-1.43805708	0.369064	8.76E-05	0.00278265	RNA5S6
ENSG00000201355	15.5741881	-1.43805708	0.369064	8.76E-05	0.00278265	RNA5S14
ENSG00000201588	15.5741881	-1.43805708	0.369064	8.76E-05	0.00278265	RNA5S2
ENSG00000201925	15.5741881	-1.43805708	0.369064	8.76E-05	0.00278265	RNA5S15
ENSG00000202257	15.5741881	-1.43805708	0.369064	8.76E-05	0.00278265	RNA5S16
ENSG00000202521	15.5741881	-1.43805708	0.369064	8.76E-05	0.00278265	RNA5S7
ENSG00000202526	15.5741881	-1.43805708	0.369064	8.76E-05	0.00278265	RNA5S13
ENSG00000196167	620.618672	-0.608143736	0.65604026	9.29E-05	0.00287392	COLCA1
ENSG00000178075	635.42361	-0.760191943	0.59041777	9.26E-05	0.00287392	GRAMD1C
ENSG00000182463	749.468281	-0.877802736	0.54419563	9.33E-05	0.00288273	TSHZ2
ENSG00000050030	373.033627	-0.857769578	0.551805	9.63E-05	0.00296542	NEXMIF
ENSG00000271858	141.943325	-0.592351641	0.66326089	9.67E-05	0.00297309	CYB561D2
ENSG00000256948	851.277362	-1.217881694	0.4299135	9.79E-05	0.00299673	NA
ENSG00000117791	447.755677	-0.706162183	0.61294852	0.0001008	0.0030703	MTARC2
ENSG00000184911	41.275268	-1.033639988	0.48847614	0.0001019	0.00309945	DMRTC1B
ENSG00000206417	624.444943	-0.602253188	0.65872436	0.00010429	0.00315061	NA
ENSG00000235174	34.5759916	-11.28690534	0.00040022	0.00010608	0.00319576	NA
ENSG00000226751	387.206887	-0.602782905	0.65848254	0.00010657	0.00320625	NA
ENSG00000231233	146.370151	-1.128876444	0.45727171	0.00011045	0.00329569	NA
ENSG00000241106	7209.90847	-0.857902296	0.55175424	0.0001117	0.00331997	HLA-DOB
ENSG00000109436	8359.84037	-0.616897934	0.6520715	0.00011222	0.00332107	TBC1D9
ENSG00000251867	75.3936731	-0.784617542	0.58050583	0.00011578	0.00340633	NA
ENSG00000233255	7.55506309	-1.160847652	0.44724968	0.00011622	0.00341321	NA
ENSG00000241644	59.7149722	-0.58663804	0.66589285	0.00011799	0.00344236	INMT
ENSG00000104067	2287.21104	-0.85690406	0.55213614	0.00012033	0.0034829	TJP1
ENSG00000259319	200.672203	-0.665293336	0.63056048	0.00013124	0.00368947	NA
ENSG00000186300	449.410807	-0.614216052	0.65328479	0.00013365	0.00373187	ZNF555
ENSG00000249948	730.719286	-1.101187492	0.46613266	0.00013871	0.00385866	GBA3
ENSG00000151490	228.518373	-0.847604556	0.55570666	0.00014156	0.00390489	PTPRO
ENSG00000112782	239.71577	-0.805789476	0.57204896	0.00014999	0.00406673	CLIC5
ENSG00000184205	7317.65453	-0.586892863	0.66577525	0.0001566	0.00418782	TSPYL2
ENSG00000127528	21770.3672	-0.614378333	0.65321131	0.000158	0.00421505	KLF2
ENSG00000138640	2090.24456	-0.623905854	0.64891173	0.00016123	0.00427562	FAM13A
ENSG00000144619	97.5621062	-1.030975722	0.48937906	0.00017032	0.00445784	CNTN4
ENSG00000128573	819.005403	-0.667124895	0.62976047	0.00018409	0.00467456	FOXP2
ENSG00000064763	1471.34781	-0.690208715	0.61976418	0.00018948	0.00477317	FAR2
ENSG00000174123	908.945764	-0.942896959	0.52018729	0.00019135	0.00481459	TLR10
ENSG00000165259	149.690851	-0.609329397	0.65550133	0.00019312	0.00484841	HDX
ENSG00000134532	143.614514	-0.845645985	0.55646159	0.00019757	0.00492661	SOX5
ENSG00000154874	2960.2253	-0.778455247	0.58299069	0.00020642	0.00507867	CCDC144B
ENSG00000198832	3079.66629	-0.636615147	0.6432203	0.00020686	0.00508376	SELENOM
ENSG00000267107	36.8060861	-0.918424629	0.52908645	0.00021089	0.00517716	PCAT19
ENSG00000105737	321.913885	-0.618656904	0.65127696	0.00021986	0.00533813	GRIK5
ENSG00000125968	376.456808	-0.857323267	0.55197573	0.0002263	0.00546458	ID1
ENSG00000186056	426.602954	-0.594198593	0.66241232	0.00024273	0.00571166	MATN1-AS1
ENSG00000223601	31.0416479	-1.618956951	0.32557076	0.00024454	0.00574223	EBLN1
ENSG00000132837	57.477665	-0.659319244	0.633177	0.00025165	0.00585324	DMGDH
ENSG00000128016	34219.6754	-0.631059671	0.64570197	0.00025815	0.00596671	ZFP36
ENSG00000067082	57505.7397	-0.652191802	0.63631287	0.00025943	0.00599007	KLF6
ENSG00000247774	841.491491	-0.634740535	0.64405664	0.0002707	0.00614775	PCED1B-AS1
ENSG00000213075	24.6980604	-1.069454965	0.47649898	0.0002821	0.0063805	RPL31P11
ENSG00000097096	50.6418545	-0.87786845	0.54417084	0.00028569	0.0064419	SYDE2
ENSG00000188906	2051.32564	-0.814845362	0.56846942	0.00028786	0.00648439	LRRK2
ENSG00000267519	1980.22483	-0.800469732	0.5741622	0.00029415	0.00657036	NA
ENSG00000203734	17.4856434	-0.665054738	0.63066478	0.00029582	0.00659531	ECT2L
ENSG00000156466	62.2276691	-1.587138794	0.33283088	0.00029606	0.00659531	GDF6
ENSG00000104324	1261.43763	-0.597867465	0.6607299	0.00030465	0.00672812	CPQ
ENSG00000135298	424.680876	-1.090622389	0.46955876	0.00030737	0.00677221	ADGRB3
ENSG00000174912	24.7452759	-0.704425545	0.6136868	0.00031031	0.00681663	NA
ENSG00000218052	81.1691269	-0.711555867	0.61066122	0.00035284	0.00749805	ADAMTS7P4
ENSG00000137936	988.962913	-0.730116377	0.60285528	0.00036658	0.00772042	BCAR3
ENSG00000254870	216.68543	-0.758637383	0.59105431	0.00036875	0.00772496	ATP6V1G2-DDX39B
ENSG00000177839	195.505569	-0.767324419	0.58750604	0.0003686	0.00772496	PCDHB9
ENSG00000131016	618.397748	-0.851895341	0.55405637	0.00037124	0.00776965	AKAP12
ENSG00000258352	18.9921525	-0.882887721	0.54228091	0.00038343	0.00796471	NA
ENSG00000080561	407.529475	-0.673621685	0.62693089	0.00038457	0.00798086	MID2
ENSG00000138378	845.973741	-0.707474196	0.61239135	0.00039742	0.00817847	STAT4
ENSG00000142178	29877.6489	-0.64975406	0.63738896	0.00040371	0.00826949	SIK1
ENSG00000244682	993.121847	-0.828391609	0.56315673	0.00040677	0.0083169	NA
ENSG00000260317	29.2918619	-0.820420541	0.56627685	0.00041797	0.0084754	NA
ENSG00000123612	390.646356	-0.649686386	0.63741886	0.00042475	0.00858137	ACVR1C
ENSG00000138119	744.403928	-1.039233925	0.48658578	0.00043808	0.00876283	MYOF
ENSG00000182759	428.758106	-1.26205569	0.41694943	0.00046988	0.00924033	MAFA
ENSG00000091972	2463.12034	-0.705775382	0.61311288	0.00047118	0.00924938	CD200
ENSG00000225217	523.607681	-0.788694257	0.57886777	0.00047307	0.00925371	NA
ENSG00000166603	676.251982	-1.135370035	0.45521814	0.00048981	0.00949725	MC4R
ENSG00000078018	753.117546	-0.847504792	0.55574509	0.00049544	0.00958128	MAP2
ENSG00000198416	136.942066	-0.707074734	0.61256093	0.00049749	0.00961256	NA
ENSG00000170160	3395.22955	-0.789698822	0.57846484	0.00050089	0.00961937	CCDC144A
ENSG00000119508	385.673057	-0.955125994	0.51579654	0.00050077	0.00961937	NR4A3
ENSG00000214140	53.2857543	-1.103967103	0.46523544	0.00050475	0.00966004	PRCD
ENSG00000137078	382.704315	-0.851998262	0.55401684	0.00051681	0.00978932	SIT1
ENSG00000136014	21.0095538	-0.891254836	0.53914497	0.00052995	0.00998979	USP44
ENSG00000240979	19.3838342	-0.934481529	0.52323047	0.00058024	0.01058583	NA
ENSG00000163631	31.1659218	-1.016293503	0.49438487	0.00058177	0.01058583	ALB
ENSG00000110092	42562.5352	-0.694679305	0.61784664	0.00058441	0.01061604	CCND1
ENSG00000135604	1924.66382	-0.682562227	0.62305774	0.00061197	0.01094582	STX11
ENSG00000081377	1170.98912	-0.716657412	0.60850566	0.00062442	0.01110569	CDC14B
ENSG00000228261	22.4267267	-0.954065867	0.5161757	0.0006293	0.01113185	NA
ENSG00000154099	496.857959	-0.815118498	0.5683618	0.00063342	0.01116841	DNAAF1
ENSG00000168671	312.928758	-0.971420118	0.51000379	0.0006371	0.01122293	UGT3A2
ENSG00000169213	1118.73536	-0.695679517	0.61741844	0.00065106	0.01144384	RAB3B
ENSG00000125409	32.2639274	-0.703861635	0.61392672	0.0006517	0.01144384	TEKT3
ENSG00000185112	116.158806	-0.631412237	0.64554419	0.00065287	0.01145529	FAM43A
ENSG00000241163	231.589534	-0.764892101	0.58849738	0.00065632	0.0114794	LINC00877
ENSG00000184588	11237.849	-0.647628279	0.63832883	0.00065745	0.01148241	PDE4B
ENSG00000130518	414.007829	-0.754557414	0.59272819	0.00067661	0.01168643	IQCN
ENSG00000273018	2967.20435	-0.63001132	0.64617135	0.00068262	0.01175371	FAM106A
ENSG00000198650	40.4181348	-0.826662391	0.56383213	0.00069879	0.01193525	TAT
ENSG00000108387	79.0839955	-0.69673314	0.6169677	0.00071344	0.01212276	SEPTIN4
ENSG00000147642	63.6408194	-0.828345975	0.56317454	0.00072011	0.0122093	SYBU
ENSG00000070087	975.178474	-0.729214975	0.60323207	0.00073534	0.01241053	PFN2
ENSG00000162746	941.078499	-0.663118891	0.63151159	0.00077175	0.01280166	FCRLB
ENSG00000087074	42732.1149	-0.632461694	0.64507477	0.00077756	0.01285842	PPP1R15A
ENSG00000108551	665.630912	-0.76638899	0.58788709	0.00078699	0.01298528	RASD1
ENSG00000179715	475.553893	-0.626855201	0.64758649	0.0008025	0.01312006	PCED1B
ENSG00000228962	23.7921174	-1.093810364	0.4685223	0.00083437	0.01347589	NA
ENSG00000104341	849.27673	-0.635169268	0.64386527	0.00085299	0.01369679	LAPTM4B
ENSG00000163624	86.8575534	-0.733970966	0.60124672	0.00088853	0.01406344	CDS1
ENSG00000169184	258.525184	-0.714142714	0.60956724	0.00095006	0.01481496	MN1
ENSG00000196482	679.415149	-0.88093877	0.54301397	0.00096366	0.01498485	ESRRG
ENSG00000122035	97.9635585	-0.634367771	0.64422307	0.00096573	0.01499589	RASL11A
ENSG00000185345	102.167573	-0.756509268	0.59192682	0.00097581	0.01510272	PRKN
ENSG00000141316	394.922656	-1.363005466	0.38877155	0.00106229	0.01608919	SPACA3
ENSG00000171813	360.61598	-0.664146292	0.63106202	0.00111698	0.01661558	PWWP2B
ENSG00000146192	2669.73986	-0.6000155	0.65974687	0.00112262	0.01667211	FGD2
ENSG00000143545	652.518522	-0.617448506	0.6518227	0.00117605	0.01714367	RAB13
ENSG00000152784	211.110475	-0.644463879	0.63973048	0.00121522	0.01739051	PRDM8
ENSG00000250327	35.3196572	-0.646231236	0.63894726	0.0012196	0.01741988	RPSAP70
ENSG00000145632	947.607839	-0.667097853	0.62977227	0.00122017	0.01741988	PLK2
ENSG00000150556	669.922052	-0.937832191	0.52201668	0.00123085	0.01749341	LYPD6B
ENSG00000155974	292.454092	-0.714507933	0.60941295	0.00123231	0.01750287	GRIP1

ENSG00000265817	28.3693532	-0.775821289	0.58405604	0.00125768	0.0177834	FSBP
ENSG00000144031	99.259596	-0.736327931	0.60026526	0.00128512	0.01805614	ANKRD53
ENSG00000176697	43.6696609	-0.777956414	0.5831923	0.00137712	0.01899678	BDNF
ENSG00000120875	6416.86493	-0.729330102	0.60318393	0.00140162	0.01911086	DUSP4
ENSG00000164344	28.8725803	-0.833005908	0.56135841	0.001421	0.01930382	KLKB1
ENSG00000155307	1195.06937	-0.681636638	0.6234576	0.00144852	0.01955766	SAMSN1
ENSG00000139193	2394.44975	-0.745835265	0.59632252	0.0014568	0.01960147	CD27
ENSG00000166897	153.69681	-0.983020225	0.50591951	0.00148517	0.01985881	ELFN2
ENSG00000177606	107718.609	-0.590764237	0.66399108	0.0014968	0.01996608	JUN
ENSG00000071242	587.785137	-0.587179243	0.6656431	0.00152491	0.02027998	RPS6KA2
ENSG00000145147	167.726734	-0.895711813	0.53748194	0.00152871	0.02030805	SLIT2
ENSG00000205929	50.2324119	-0.851103755	0.55436045	0.001537	0.02038025	C21orf62
ENSG00000230500	6.23873836	-1.325105548	0.39911999	0.00156672	0.02062531	NA
ENSG00000104081	8116.35587	-0.696506549	0.61706461	0.00158721	0.02077159	BMF
ENSG00000226571	36.3005511	-1.018031075	0.49378979	0.00159803	0.02087623	NA
ENSG00000114251	1712.78426	-0.767363353	0.58749018	0.00161781	0.02109579	WNT5A
ENSG00000248991	16.5410477	-1.12224	0.45938001	0.00169778	0.02179359	NA
ENSG00000154898	599.835536	-0.87107395	0.5467397	0.00174726	0.02222262	NA
ENSG00000229228	558.944926	-0.711257768	0.61078741	0.00176486	0.02231828	LINC00582
ENSG00000197329	25799.4733	-0.68053701	0.62393299	0.00183273	0.02296667	PELI1
ENSG00000103316	28.6452891	-1.231347046	0.42591958	0.00184764	0.02303621	CRYM
ENSG00000215811	12.4125535	-0.791547432	0.57772409	0.00185197	0.02306422	BTNL10
ENSG00000078589	1022.33923	-0.5966033	0.66130912	0.00186349	0.02316806	P2RY10
ENSG00000179603	39.5324625	-0.96565932	0.51204435	0.0018989	0.02346397	GRM8
ENSG00000235631	139.989222	-0.600791073	0.65939229	0.00194087	0.02383626	RNF148
ENSG00000210196	601.13056	-0.973924823	0.50911913	0.00202725	0.02444962	NA
ENSG00000130600	29.6779942	-1.418635555	0.37406592	0.00204056	0.02448666	H19
ENSG00000196159	513.081378	-0.873379114	0.54586681	0.00213874	0.02533916	FAT4
ENSG00000267543	9.47377828	-0.999366653	0.50021955	0.00218106	0.02562079	NA
ENSG00000164197	101.586542	-0.878427391	0.54396005	0.00218478	0.0256509	RNF180
ENSG00000144868	240.094174	-0.691978594	0.61900433	0.00219897	0.02577646	TMEM108
ENSG00000189423	669.08463	-1.053436231	0.48181919	0.00221873	0.02592579	NA
ENSG00000258130	164.050135	-0.769713375	0.58653399	0.00222246	0.02594201	LOC613038
ENSG00000186847	33.8047091	-1.461228223	0.36318381	0.00223122	0.02601684	KRT14
ENSG00000146666	200.257582	-0.74395378	0.59710072	0.00230053	0.0266426	LINC00525
ENSG00000231858	12.2806121	-0.843328879	0.55735604	0.00231368	0.0267465	LOC105373805
ENSG00000162595	117.330809	-1.249132759	0.42070103	0.00231917	0.0267465	DIRAS3
ENSG00000165899	323.228274	-0.619617795	0.65084333	0.00235713	0.02705593	OTOGL
ENSG00000153060	10.6531854	-0.961995142	0.5133465	0.00235595	0.02705593	TEKT5
ENSG00000122733	24.9256699	-1.054695284	0.48139889	0.00236103	0.02706024	PHF24
ENSG00000260693	38.5407315	-0.699748121	0.61567969	0.00247861	0.02800191	NA
ENSG00000271730	68.5144661	-0.5996925	0.65989459	0.00250174	0.0280705	NA
ENSG00000116741	10561.769	-0.733502516	0.60144198	0.00259488	0.02884461	RGS2
ENSG00000260205	15.7595977	-0.968026849	0.51120475	0.00263287	0.02916436	NA
ENSG00000175505	97.1049627	-0.652846111	0.63602434	0.0026669	0.02942365	CLCF1
ENSG00000271894	584.929354	-0.966789505	0.51164338	0.00275742	0.03010723	NA
ENSG00000182601	70.1137477	-0.871922949	0.54641805	0.00276765	0.03015317	HS3ST4
ENSG00000196458	389.758706	-0.60484215	0.65754332	0.00279506	0.03038345	ZNF605
ENSG00000272024	8.3810137	-0.595813489	0.66167126	0.00282795	0.03065067	NA
ENSG00000228590	41.4178852	-0.965430413	0.5121256	0.00284631	0.03083455	MIR4432HG
ENSG00000132185	9004.56593	-0.724421776	0.60523957	0.00285797	0.03088529	FCRLA
ENSG00000104413	394.024375	-0.9471459	0.51865751	0.00286499	0.03091591	ESRP1
ENSG00000226979	97.602099	-0.626002314	0.64796944	0.0029215	0.03137283	LTA
ENSG00000135069	5238.91625	-0.599314672	0.66006743	0.00295103	0.03158269	PSAT1
ENSG00000255864	12.8872569	-0.918376337	0.52910416	0.00296629	0.03166954	NA
ENSG00000205189	1788.74085	-0.723328697	0.60569831	0.00300084	0.03187672	ZBTB10
ENSG00000255471	6.14191355	-1.135534326	0.45516631	0.00300153	0.03187672	NA
ENSG00000196781	2531.49307	-0.74310356	0.59745271	0.0030103	0.03192385	TLE1
ENSG00000065809	5170.12434	-0.645590957	0.6392309	0.00302624	0.03207758	FAM107B
ENSG00000106952	376.324062	-0.827546572	0.56348669	0.00309003	0.0325048	TNFSF8
ENSG00000184305	225.242525	-0.589702825	0.66447977	0.00317787	0.0332079	CCSER1
ENSG00000115896	578.526074	-0.588250198	0.66514916	0.0031879	0.03327138	PLCL1
ENSG00000177025	18.2305097	-0.688513508	0.62049285	0.00323062	0.03360055	C19orf18
ENSG00000159167	60.0533918	-2.031037427	0.24467906	0.00336371	0.03448266	STC1
ENSG00000263740	13.25235	-0.830058521	0.56250642	0.00337584	0.03455904	RN7SL4P
ENSG00000112799	322.492868	-0.668495873	0.6291623	0.00338146	0.03460059	LY86
ENSG00000253671	12.0778315	-0.742275183	0.59779586	0.00339945	0.03467487	LOC101930275
ENSG00000230910	237.324241	-0.881310081	0.54287423	0.0034061	0.03472431	NA
ENSG00000167642	2393.37299	-0.588886061	0.66485606	0.00340815	0.03472926	SPINT2
ENSG00000241935	67.67145	-0.741831265	0.59797983	0.00344884	0.03496684	HOGA1
ENSG00000185338	151.146057	-0.67092547	0.62810364	0.00346685	0.03508523	SOCS1
ENSG00000197705	796.756709	-1.001250084	0.49956694	0.00347832	0.03515307	KLHL14
ENSG00000187824	228.536227	-0.611626204	0.65445858	0.00352565	0.03550655	TMEM220
ENSG00000224945	9.34684064	-0.760296907	0.59037482	0.00354484	0.03564984	NA
ENSG00000165685	647.879263	-0.816935948	0.56764625	0.0035729	0.0358511	TMEM52B
ENSG00000172548	220.657925	-0.815293006	0.56829306	0.0037673	0.03715784	NIPAL4
ENSG00000197584	228.335372	-0.749740357	0.59471058	0.00384352	0.03763585	KCNMB2
ENSG00000186265	2824.32198	-0.603048762	0.65836121	0.00393088	0.03824977	BTLA
ENSG00000197385	172.646574	-0.716155496	0.6087174	0.00395374	0.03834768	ZNF860
ENSG00000128536	192.33167	-0.686025543	0.62156383	0.00404012	0.03892969	CDHR3
ENSG00000273179	6.90161425	-0.86375132	0.54952182	0.00427987	0.04060376	NA
ENSG00000153234	3619.80792	-0.610616201	0.65491692	0.00436766	0.04121037	NR4A2
ENSG00000245205	8.31149389	-0.649320729	0.63758044	0.00439793	0.04138689	EEF1A1P4
ENSG00000240505	4472.48479	-0.788686806	0.57887076	0.00444362	0.04175645	TNFRSF13B
ENSG00000128045	22.4211791	-0.765130957	0.58839996	0.00445534	0.04180278	RASL11B
ENSG00000228158	6.55551166	-0.961419751	0.51355128	0.00445854	0.04181507	NA
ENSG00000102554	615.490699	-0.705604165	0.61318565	0.00454627	0.04238659	KLF5
ENSG00000182489	225.127085	-0.776050218	0.58396337	0.00459317	0.04262634	XKRX
ENSG00000183230	258.298847	-0.724218008	0.60532506	0.00465232	0.04297715	CTNNA3
ENSG00000197558	66.8973903	-0.692207935	0.61890594	0.00470031	0.04331347	SSPOP
ENSG00000265263	35.5444353	-2.40938579	0.18823597	0.00481318	0.044127	NA
ENSG00000150625	1795.11531	-0.728101806	0.60369769	0.00484641	0.04431856	GPM6A
ENSG00000206077	142.894487	-0.683663295	0.6225824	0.00486105	0.04436858	ZDHHC11B
ENSG00000118523	4184.53238	-0.687266126	0.62102957	0.00488321	0.04442764	CCN2
ENSG00000109819	1130.60168	-0.678552791	0.62479171	0.00501193	0.04515526	PPARGC1A
ENSG00000260093	8.61967898	-0.74624836	0.5961518	0.00520004	0.0463763	NA
ENSG00000255730	338.365647	-0.928691295	0.52533467	0.00527737	0.04681762	NA
ENSG00000170743	11.611023	-0.779239175	0.58267399	0.00530168	0.04694379	SYT9
ENSG00000236051	10.4036402	-0.74466293	0.59680729	0.00531048	0.04697965	MYCBP2-AS1
ENSG00000176490	437.447426	-0.595801277	0.66167686	0.00541347	0.04745468	DIRAS1
ENSG00000270096	18.1763735	-1.056381602	0.48083653	0.00567715	0.0489712	NA
