# Supplementary material for: Clonal evolution after treatment pressure in multiple myeloma: heterogenous genomic aberrations and transcriptomic convergence
Source: Leukemia. 2022 May 28;36(7):1887–97. doi: 10.1038/s41375-022-01597-y (PMC9252918; doi:10.1038/s41375-022-01597-y)
Supplement: Supplementary file 14 — Table S8 [file 41375_2022_1597_MOESM14_ESM.pdf]

Table S8. Proliferative index for all included samples with RNAseq data, in addition to other sample information.

Patient_ID	Diagnosis	Progression	Progression	Progression	Progression	PI Change[§]	TBS	PFS	2nd sample 1st progression	Ig-T/HRD	CCND1 exp	CCND2 exp	Cyto risk[$]	Risk status	Progression <2 y	Progression < 1 y	dead	TTD
65	1.0	3.9				**2.93**	22	19	yes	HRD	MH	N		HR	yes	no	**y**	**0**
63	1.9	4.7				**2.79**	15	3	no	t(8;14)	N	MH	HR (del17)	HR	yes	no*	**y**	**3**
27	1.5	5.5	4.0			**2.50**	17/2	17	yes	t(4;14)	N	MH	HR (t(4;14))	HR	yes	no	**y**	**1**
31	1.1	2.0	3.1			**2.05**	14/5	12	yes	t(4;14)	N	L	HR (t(4;14))	HR	yes	no	**y**	**1**
20	1.9	4.2				**2.32**	50	13	no	t(11;14)	VH	N		HR	no*	no	**y**	**2**
60	1.5	4.2				**2.65**	18	11	no	HRD	MH	N		SR	yes	no*	**y**	**0**
28	1.9	4.5				**2.61**	9	9	yes	HRD	N	L		SR	yes	yes	y	19
46	1.9	4.3				**2.47**	18	7	no	unknown	MH	VH		SR	yes	no*	**y**	**3**
51	1.1	3.2				**2.09**	18	9	no	HRD	MH	L		SR	yes	no*	**y**	**1**
49	1.6	3.6				**2.02**	18	10	no	HRD	MH	N		SR	yes	no*	**y**	**0**
55	0.8	2.3				**1.48**	18	17	yes	HRD	N	MH		SR	yes	no	n	>41
58	2.5	3.9				**1.41**	9	8	yes	HRD	N	MH		SR	yes	yes	**y**	**4**
50	1.5	2.9				**1.45**	36	2	no	t(11;14)	VH	N		SR	no*	no*	n	>29
52	1.4	4.4				**3.06**	39	36	no	HRD	L	N		SR	no	no	n	>27
54	1.8	3.4				**1.53**	33	32	yes	t(6;14)	N	N**		SR	no	no	n	>13
53	0.6	2.1				**1.52**	42	42	yes	HRD	MH	N		SR	no	no	n	>25
18	2.3	2.3	3.7			**1.35**	17/14	16	yes	HRD	N	MH		SR	yes	no	**y**	**0**
10	1.8	3.0				**1.21**	16	11	yes	t(4;14)	N	MH	HR (t(4;14))	HR	yes	no	y	19
24	0.3	1.4				**1.07**	24	2	no	t(11;14)	MH	N		HR	yes	no*	**y**	**10**
15	1.4	1.6	2.3			**0.90**	48/13	45	yes	HRD	MH	N	HR (t(4;14))	HR	no	no	y	46
26	2.51	2.13	3.28			**0.78**	14/6	13	yes	HRD	N	MH		HR	yes	no	**y**	**1**
34		2.5	2.7	3.5	3.3	**0.75**	9/7/7	-	no	HRD	N	N		SR	no	no	**y**	**2**
32	0.2	0.9				**0.69**	18	18	yes	HRD	L	N		SR	yes	no	**y**	**1**
42	1.3	1.9				**0.65**	30	26	yes	HRD	N	L		SR	no	no	y	38
23	0.5	0.4	1.1			**0.52**	25/38	25	yes	HRD	L	N		SR	no	no	y	14
8		1.3	1.8			**0.49**	9	-	no	t(11;14)	VH	N		SR	no	no	**y**	**4**
25	0.3	0.8	0.8			**0.49**	16/23	9	no	HRD	L	N		SR	yes	no	y	27
47	1.8	2.2				**0.42**	21	19	yes	t(4;14)	N	MH	HR (t(4;14))	HR	yes	no	y	31
61	2.9	3.2				0.34	15	15	yes	t(11;14)	VH	N	HR (del17)	HR	yes	no	y	21
59	1.0	1.3				0.31	18	11	no	t(4;14)	N	MH	HR (t(4;14))	HR	yes	no	n	>43
4	1.7	1.9				0.28	39	21	no	t(11;14)	VH	N		HR	no*	no	**y**	**6**
9	1.0	1.2				0.25	38	27	no	t(11;14)	VH	N		SR	no	no	n	>46
43	1.1	1.6	1.3			0.24	30/21	29	yes	t(4;14)	N	MH	HR (t(4;14))	HR	no	no	y	21
48	1.7	1.8				0.18	18	17	yes	HRD	N	MH		SR	yes	no	**y**	**5**
30	0.5	0.6				0.15	12	11	yes	HRD	L	N		SR	yes	yes	n	>47
45	1.3	1.4				0.10	36	37	yes	t(11;14)	VH	N		SR	no	no	n	>7
29	0.5	0.5				-0.01	5	5	yes	HRD	L	N		SR	yes	yes	y	20
33	0.9	0.9				-0.06	18	16	yes	t(11;14)	VH	N		SR	yes	no	y	22
35		1.0	0.9			-0.07	24	-	no	HRD	L	N		SR	no	no	y	26
40	1.7	1.6	1.2	1.7		-0.07	21/12/21	18	yes	t(11;14)	VH	N		SR	yes	no	n	>18
66	2.2	1.9				-0.29	21	7	no	HRD	MH	MH		SR	yes	no*	y	19
36		1.50	1.12			-0.38	36	-	no	HRD	L	N		SR	no	no	y	48
67	1.2	0.8				**-0.42**	18	19	no	HRD	N	L		SR	yes	no	n	>32
44	2.3	1.8				**-0.48**	45	5	no	t(11;14)	VH	N		SR	no*	no*	n	22
57	2.2	1.7				**-0.53**	27	26	yes	t(12;14)	N	VH		SR	no	no	n	>36
62	2.1	1.5				**-0.56**	27	21	no	t(4;14)	L	VH	HR (t(4;14))	HR	no*	no	**y**	**6**
41	1.6	1.1				**-0.57**	36	18	no	t(4;14)	L	L	HR (t(4;14))	HR	no*	no	y	29
56	2.5	1.1				**-1.33**	15	15	yes	t(11;14)	VH	N		SR	yes	no	n	>36
64	3.6	1.7				**-1.95**	3	3	yes	t(4;14)	N	MH	HR (t(4;14))	HR	yes	yes	**y**	**10**

[§] change in PI (Proliferative Index; see methods) from first to last sample. Bold=change >0.4.

[$] Cytogenetic risk, High Risk (HR); t(4;14), t(14;16) or del17p detected by FISH (in-house cohort) or canonical translocation from long insert WGS (CoMMpass)

Ig-T/HRD: Cytogenetic subgroup, includes t(11;14)/CCND1, t(12;14)/ CCND2, t(6;14)/CCND3, t(4;14)/WHSC1(NSD2/MMSET), t(8;14)/MAFA (see Supplementary Table 12).

Risk status: HR: R-ISS 3 (or if not available, ISS 3), SR: Not HR

PFS: Time to first progression (from diagnosis). Only included for diagnosis-progression patients.

TTD = Time To Death or Time To Last Control (if still alive) from last sample.

TBS: Time between samples (months). If more than 2 samples, "/" are used to separate samples pairs, i.e S1-S2/S2-S3

CCND1/2 exp: Expression of CCND1/2 at diagnosis. N: No expression (TPM<2), L: Low (TPM 2-20), MH: Medium high (TPM 20-100), VH: Very high (TPM>100).

Relapse < 2y; yes: relapse within 24 months from diagnosis, if not: no. For patients with many relapses, first sample are used for this definition. *Patient has progressed within 24 months but no BM sample available for that time

Relapse < 1y; yes: relapse within 12 months from diagnosis, if not: no. For patients with many relapses, first sample are used for this definition. *Patient has progressed within 12 months but no BM sample available for that time
