# Supplementary material for: Clonal evolution after treatment pressure in multiple myeloma: heterogenous genomic aberrations and transcriptomic convergence
Source: Leukemia. 2022 May 28;36(7):1887–97. doi: 10.1038/s41375-022-01597-y (PMC9252918; doi:10.1038/s41375-022-01597-y)
Supplement: Supplementary file 15 — Table S9 [file 41375_2022_1597_MOESM15_ESM.pdf]

Table S9. Overview ssGSEA pathway data of selected Hallmark genesets. Includes also measured NFkB index (NKkBi) and gene expression levels for *MYC* (TPM). S1: Early sample, LS: Latest sample. ESD: End stage disease.

						ssGSEA Hallmark genesets			ssGSEA Hallmark genesets		
**Patient_ID**	**Ig-T/HRD**	**ESD pair**	**PI S1**	**PI LS**	**PI Change**	**Glycolysis S1**	**Glycolysis LS**	**Change**	**OXPHOS S1**	**OXPHOS LS**	**Change**
63	t(8;14)	x	1.9	4.7	2.79	-623	183	806	-1013	664	1676
28	HRD		1.9	4.5	2.61	-174	256	430	289	680	391
52	HRD		1.4	4.4	3.06	-766	428	1195	130	842	712
46	unknown	x	1.9	4.3	2.47	-290	209	499	-494	648	1142
20	t(11;14)	x	1.9	4.2	2.32	-566	444	1010	-639	726	1365
60	HRD	x	1.5	4.2	2.65	-700	-18	682	-441	272	713
27	t(4;14)	x	1.5	4.0	2.50	-827	166	993	-1079	766	1845
58	HRD	x	2.5	3.9	1.41	-506	142	648	146	637	491
65	HRD	x	1.0	3.9	2.93	-612	591	1204	-413	709	1122
18	HRD	x	2.3	3.7	1.35	-629	-74	555	319	112	-207
49	HRD	x	1.6	3.6	2.00	-241	-18	222	290	500	210
54	t(6;14)		1.8	3.4	1.53	-592	-169	423	-388	-143	245
34	HRD	x	2.5	3.3	0.75	-89	26	116	385	233	-151
26	HRD	x	2.5	3.3	0.78	-293	-66	227	-329	-143	186
61	t(11;14)		2.9	3.2	0.34	175	170	-5	488	282	-207
51	HRD	x	1.1	3.2	2.09	-669	-103	565	-1090	-537	553
31	t(4;14)	x	1.1	3.1	2.05	-628	162	790	-918	-371	547
10	t(4;14)		1.8	3.0	1.21	-669	-365	304	-648	-556	92
50	t(11;14)		1.5	2.9	1.45	-439	-277	162	-684	113	797
55	HRD		0.8	2.3	1.48	-885	-403	482	-663	-95	568
15	HRD		1.4	2.3	0.90	-264	-731	-467	466	-369	-835
47	t(4;14)		1.8	2.2	0.42	-564	-493	71	-405	-852	-447
53	HRD		0.6	2.1	1.52	-899	-328	571	-536	597	1133
4	t(11;14)	x	1.7	1.9	0.28	-410	-329	81	-291	302	593
42	HRD		1.3	1.9	0.65	-740	-497	243	-575	-400	175
66	HRD		2.2	1.9	-0.29	-437	-302	135	550	555	5
48	HRD	x	1.7	1.8	0.18	-751	-597	154	-554	-509	45
44	t(11;14)		2.3	1.8	-0.48	-439	-432	7	-814	-952	-139
8	t(11;14)	x	1.3	1.8	0.49	-988	-706	281	-1701	-906	795
57	t(12;14)		2.2	1.7	-0.53	-237	-23	213	317	462	145
40	t(11;14)		1.7	1.7	-0.07	-774	-471	304	-1065	-965	100
64	t(4;14)	x	3.6	1.7	-1.95	65	-274	-339	-72	-142	-71
62	t(4;14)	x	2.1	1.5	-0.59	-377	-344	33	-94	-451.86409	-358
24	t(11;14)	x	0.3	1.4	1.07	-737	-732	5	-1598	-648	950
45	t(11;14)		1.3	1.4	0.10	-659	-366	293	-579	-256	323
43	t(4;14)		1.1	1.3	0.24	-811	-412	400	-1160	-1074	85
59	t(4;14)		1.0	1.3	0.31	-695	-643	52	-1089	-1401	-312
9	t(11;14)		1.0	1.2	0.25	-546	-589	-43	-999	-1523	-524
56	t(11;14)		2.5	1.1	-1.33	-656	-622	34	-1374	-1435	-60
36	HRD		1.5	1.1	-0.38	-583	-438	145	-741	-1379	-639
41	t(4;14)		1.6	1.1	-0.57	-842	-773	69	-1142	-1081	61
23	HRD		0.5	1.1	0.52	-794	-351	443	-1257	-467	791
35	HRD		1.0	0.9	-0.07	-758	-711	47	-1077	-1100	-23
32	HRD	x	0.2	0.9	0.69	-815	-401	414	-1418	-189	1229
33	t(11;14)		0.9	0.9	-0.06	-336	-423	-87	-644	-1373	-729
25	HRD		0.3	0.8	0.49	-760	-715	45	-903	-665	238
67	HRD		1.2	0.8	-0.42	-666	-882	-216	-239	-455	-216
30	HRD		0.5	0.6	0.15	-840	-802	38	-1517	-1590	-73
29	HRD		0.5	0.5	-0.01	-1049	-936	113	-924	-1322	-398

	ssGSEA Hallmark genesets			ssGSEA Hallmark genesets			ssGSEA Hallmark genesets			ssGSEA Hallmark genesets			ssGSEA Hallmark genesets		
**Patient_ID**	**MTORC S1**	**MTORC LS**	**Change**	**MYC_TARGETS S1**	**MYC_TARGETS LS**	**Change**	**DNA REPAIR S1**	**DNA REPAIR LS**	**Change**	**RE OXY_SPEC_S1**	**RE OXY_SPEC_LS**	**Change**	**TNFA/NFkB S1**	**TNFA/NFkB LS**	**Change**
63	-758	475	1233	-1058	840	1898	-617	564	1181	-566	31	597	-314	-842	-529
28	87	816	729	266	976	710	-17	492	508	-189	239	428	-778	-360	417
52	-669	625	1294	-676	1038	1714	-901	674	1575	-565	177	742	-176	-1015	-839
46	-332	511	843	-309	986	1295	-371	681	1052	-288	36	324	-8	-553	-545
20	-666	533	1199	-1058	909	1967	-702	518	1220	-533	126	660	-217	-728	-511
60	-646	222	869	-917	432	1349	-539	250	789	-312	-76	236	-165	-1020	-855
27	-775	432	1208	-936	815	1751	-783	558	1341	-488	90	579	-264	-679	-415
58	-642	416	1058	-387	892	1280	-634	546	1179	-420	-77	344	-530	-1099	-569
65	-899	576	1474	-658	921	1580	-1023	555	1578	-517	198	715	-802	-988	-186
18	-537	-101	436	-133	223	356	32	298	266	-109	-16	94	177	-327	-504
49	2	259	258	207	453	246	-147	305	453	-75	-30	45	-906	-396	509
54	-575	-68	506	-670	110	780	-327	138	466	-317	-88	230	-87	-478	-390
34	-277	19	296	-117	131	248	-24	48	71	-37	64	100	-810	-824	-14
26	-328	92	420	-243	539	781	-155	442	597	-186	-146	40	-968	-768	200
61	240	319	79	372	281	-91	367	239	-128	129	226	97	-251	-194	58
51	-912	86	998	-1328	-408	920	-906	-266	640	-476	-217	259	-546	-636	-90
31	-883	119	1002	-1193	54	1247	-975	168	1143	-447	41	488	-863	-1126	-263
10	-850	-544	306	-1166	-885	280	-772	-497	275	-439	-360	79	-298	-346	-49
50	-438	-194	244	-570	-182	388	-408	156	564	-288	-96	192	-17	-426	-409
55	-1042	-483	558	-1243	-596	647	-916	-375	542	-371	17	388	-175	-527	-352
15	120	-428	-548	76	-393	-469	-173	-464	-291	81	-546	-627	-706	-178	528
47	-682	-730	-48	-956	-1146	-191	-716	-904	-187	-451	-391	61	-43	-309	-266
53	-906	19	925	-1270	329	1600	-915	202	1117	-534	-80	455	113	-131	-244
4	-467	-98	369	-594	-446	148	-263	-106	157	-207	139	346	-178	-969	-791
42	-639	-386	253	-903	-543	361	-558	-329	230	-405	54	459	-1060	-954	106
66	-335	-332	3	-258	-263	-5	-202	-200	3	-44	138	183	-129	-391	-262
48	-935	-578	357	-1297	-534	763	-767	-492	275	-552	-237	316	-167	-309	-142
44	-591	-693	-102	-747	-708	40	-313	-545	-232	-150	-382	-232	156	48	-108
8	-1173	-906	267	-1634	-1124	511	-1158	-793	366	-580	-477	102	158	-821	-979
57	-298	-73	225	229	103	-126	-130	57	187	-130	105	235	-363	-756	-394
40	-966	-756	209	-1294	-1399	-105	-787	-507	280	-543	-328	215	169	-834	-1003
64	174	-367	-541	108	-395	-503	26	-272	-298	167	-174	-341	-144	-135	9
62	-406	-301	106	-565	-507	58	-461	-390	70	-294	-472	-178	-231	-689	-458
24	-969	-734	235	-1516	-1008	508	-1151	-648	503	-541	-283	259	-95	-740	-645
45	-663	-324	339	-822	-738	84	-323	-327	-3	-416	-62	354	241	-63	-305
43	-1149	-716	433	-1443	-825	619	-1112	-839	273	-535	-393	142	-205	-159	46
59	-1023	-1054	-30	-1521	-1467	54	-1150	-1093	57	-529	-560	-31	-736	-786	-50
9	-1034	-792	242	-1478	-1418	60	-1079	-1097	-19	-465	-134	331	-424	405	829
56	-854	-876	-22	-1031	-1319	-288	-771	-883	-112	-551	-363	188	-709	-761	-52
36	-771	-798	-27	-1002	-1253	-251	-880	-795	85	-246	-89	157	-829	314	1142
41	-1104	-1004	100	-1533	-1423	110	-1112	-884	228	-609	-237	372	-806	-467	339
23	-1047	-649	398	-1340	-972	367	-954	-400	555	-601	-129	471	444	-955	-1399
35	-1058	-997	62	-1443	-1117	325	-1109	-789	320	-502	-451	51	-488	-332	157
32	-1123	-546	577	-1416	-518	898	-1178	-458	720	-682	-616	66	-898	-958	-60
33	-476	-726	-250	-361	-744	-384	-268	-681	-413	-281	-353	-72	467	271	-196
25	-979	-899	81	-1267	-834	433	-982	-797	185	-585	-574	11	-57	543	600
67	-845	-757	88	-1320	-1447	-128	-712	-907	-196	-524	-483	41	-772	-510	262
30	-955	-1026	-71	-1353	-1520	-167	-1109	-1191	-82	-645	-596	48	398	-193	-591
29	-886	-1030	-144	-1076	-1386	-310	-832	-1082	-250	-648	-534	113	307	323	16

	ssGSEA Hallmark genesets			ssGSEA Hallmark genesets			ssGSEA Hallmark genesets			ssGSEA Hallmark genesets		
**Patient_ID**	**TGF_BETA_S1**	**TGF_BETA_LS**	**Change**	**UV_RESP_S1**	**UV_RESP_LS**	**Change**	**ANDR_RESP_S1**	**ANDR_RESP_LS**	**Change**	**UPR_S1**	**UPR_LS**	**Change**
63	-547	-320	227	-528	-218	311	-404	-197	207	-666	-311	355
28	-228	-252	-24	-586	-482	104	-487	58	545	-38	368	407
52	-381	-524	-143	-243	-619	-376	2	-377	-380	-57	-1	55
46	-114	-402	-288	-207	-461	-254	-138	-465	-327	105	-28	-133
20	-205	-295	-90	-545	-423	123	90	39	-51	301	400	99
60	-230	-205	25	-116	-397	-281	-254	-312	-58	-301	-106	195
27	280	-281	-562	259	-34	-292	190	-24	-215	57	216	159
58	-349	-599	-250	-320	-737	-417	-135	-594	-459	-281	-703	-422
65	-187	-410	-224	-314	-758	-444	-50	-409	-359	129	-384	-513
18	251	-29	-280	211	-41	-251	8	-182	-190	171	-73	-244
49	-471	47	517	-276	19	295	-209	-82	127	-261	-135	125
54	-25	35	60	-100	-22	79	-343	-411	-68	-357	-167	190
34	-484	-288	196	-603	-388	215	-574	-390	184	-486	-150	335
26	-642	-363	279	-494	45	539	-475	-183	292	-516	-86	430
61	-129	-118	10	-268	-202	65	0	28	28	454	383	-71
51	-121	-177	-57	-264	-350	-87	-323	-257	66	-679	-367	312
31	-635	-382	254	-754	-633	121	-679	-530	150	-872	-520	352
10	-296	-361	-66	-292	-421	-128	-287	-293	-6	-463	-565	-102
50	-56	-87	-30	-7	-112	-105	125	-252	-377	16	-51	-67
55	-419	-247	172	-159	-440	-281	-37	-34	3	72	-84	-156
15	-297	-889	-592	-388	-449	-61	-43	-399	-356	293	-338	-631
47	28	-377	-405	58	-501	-559	178	-529	-708	89	-727	-815
53	7	163	156	-4	194	198	37	185	148	-239	77	316
4	-210	-9	201	-185	-51	134	-139	10	149	-441	-154	287
42	-395	3	398	-431	-283	148	-432	-418	13	-501	-413	88
66	206	-323	-529	348	-336	-684	128	-449	-577	-152	-569	-417
48	-127	216	343	37	75	38	60	-317	-377	431	-361	-792
44	30	-124	-154	313	0	-313	-101	-141	-39	-595	-411	185
8	-233	-616	-383	62	-485	-547	-394	-344	50	-560	-174	386
57	-341	-659	-318	-222	-646	-424	-151	-543	-391	126	-475	-601
40	24	-540	-564	176	-331	-508	-95	-409	-314	-387	-726	-339
64	252	-471	-722	171	-377	-548	-64	-278	-214	290	-288	-579
62	-364	10	374	-311	-190	121	-271	-95	176	-9	135	144
24	-513	-613	-100	-514	-316	198	-670	-360	310	-808	-751	57
45	122	-346	-468	5	-448	-453	92	-256	-348	156	186	30
43	-91	-128	-37	-114	1	115	-381	-173	208	-722	-322	400
59	-612	-514	98	-399	-399	0	-240	-293	-53	-194	-478	-284
9	-445	-29	417	-538	-45	493	-406	-292	113	-329	-514	-186
56	-613	-702	-89	-404	-625	-220	-496	-735	-239	-958	-1052	-94
36	-570	114	684	-656	-128	528	-570	-327	243	-698	-399	298
41	-62	-472	-410	-104	-503	-398	-225	-428	-203	-296	-411	-116
23	-153	-672	-519	167	-642	-810	-50	-650	-600	-385	-908	-523
35	-503	-377	127	-431	-163	267	-646	-429	217	-960	-689	271
32	-642	-637	5	-693	-445	248	-647	-265	382	-902	-479	423
33	413	307	-107	30	54	24	336	147	-189	452	25	-427
25	-670	158	828	-629	122	752	-644	200	844	-758	-2	756
67	-329	-120	208	-458	-233	224	-257	-21	236	-522	-293	228
30	78	-368	-446	-33	-572	-539	-192	-532	-340	-163	-713	-550
29	201	-141	-342	114	-170	-284	210	-113	-323	50	-290	-340

	ssGSEA Hallmark genesets			ssGSEA Hallmark genesets									
**Patient_ID**	**HYPOXIA_S1**	**HYPOXIA_LS**	**Change**	**PROT_SECRET_S1**	**PROT_SECRET_LS**	**Change**	**MYC TPM S1**	**MYC TPM LS**	**FC MYC**	**NFkBindex S1**	**NFkBindex LS**	**Change**	**NFkB SNVs**
63	-487	-725	-238	-604	94	698	24.3	118.9	4.89	1.53	1.90	0.37	
28	-719	-268	452	-427	-366	61	126.3	142.0	1.12	1.24	1.17	-0.07	
52	-168	-709	-541	-282	-187	96	33.2	102.9	3.10	1.19	1.09	-0.10	
46	-82	-548	-466	-330	-69	261	91.2	45.9	0.50	2.04	1.74	-0.30	
20	-318	-498	-180	258	431	173	8.6	54.5	7.78	1.16	0.99	-0.17	
60	-225	-617	-391	-174	47	221	48.8	104.8	2.14	1.98	2.17	0.19	
27	73	-298	-371	39	381	342	5.6	85.4	15.24	1.32	1.17	-0.15	
58	-388	-850	-462	-415	-803	-388	15.6	71.6	4.58	0.99	0.78	-0.21	
65	-456	-781	-326	-12	-328	-316	137.3	84.1	0.61	1.07	0.80	-0.26	
18	70	-320	-391	259	138	-121	28.1	50.1	1.78	1.97	1.97	0.00	CYLD (both TP)
49	-432	-209	224	-8	193	201	113.5	261.1	2.30	1.17	1.46	0.29	
54	-94	-277	-184	-460	-141	319	22.7	11.2	0.49	1.74	1.82	0.09	
34	-591	-589	2	-413	-137	275	4.9	25.7	5.22	0.95	1.35	0.40	
26	-716	-497	219	-489	-278	212	12.7	26.1	2.05	1.23	1.08	-0.15	
61	183	13	-169	383	396	13	0.3	0.4	1.37	1.58	1.77	0.19	
51	-480	-562	-82	-630	-205	426	9.6	9.5	0.98	1.32	1.32	0.01	
31	-899	-854	45	-841	-290	551	14.5	56.8	3.91	0.84	0.99	0.15	
10	-396	-499	-102	-470	-469	1	27.8	21.3	0.76	1.80	1.76	-0.04	
50	-70	-222	-152	216	238	22	17.4	13.9	0.80	1.23	1.42	0.19	
55	-97	-234	-136	-220	-39	181	90.0	79.4	0.88	1.43	1.34	-0.09	
15	-339	-209	130	225	-531	-756	197.9	93.9	0.47	1.19	1.14	-0.05	
47	-5	-563	-558	299	-608	-907	13.3	49.4	3.72	1.65	1.88	0.22	
53	67	-1	-68	-516	237	753	20.4	127.1	6.24	1.65	2.04	0.39	TRAF3 (LS only)
4	-152	-533	-381	-317	298	615	13.3	14.1	1.06	1.66	1.48	-0.17	
42	-661	-396	266	-466	-99	367	38.1	39.6	1.04	1.20	1.74	0.53	TRAF3(S1 only)
66	58	-367	-426	282	-670	-952	64.8	44.8	0.69	1.81	1.82	0.02	
48	137	-305	-442	490	-146	-635	18.3	50.4	2.76	1.56	1.82	0.26	
44	3	-156	-159	-96	-186	-90	49.9	83.2	1.67	1.76	1.77	0.01	TRAF3 (both TP)
8	51	-512	-563	-582	-194	387	38.4	59.9	1.56	1.60	1.06	-0.55	
57	16	-338	-354	225	-331	-556	115.7	155.1	1.34	1.59	1.58	-0.01	
40	126	-442	-568	128	-433	-561	6.2	4.3	0.70	1.19	1.49	0.30	
64	31	-79	-110	296	-399	-695	25.0	12.6	0.50	1.74	1.76	0.02	
62	-408	-495	-87	-166	-21	145	12.1	8.0	0.66	1.07	1.05	-0.01	
24	-506	-715	-209	-997	-397	599	14.5	26.4	1.82	1.22	1.64	0.43	
45	162	-386	-548	44	-41	-85	14.2	14.4	1.01	1.31	1.35	0.03	
43	-272	-92	180	-353	-399	-46	178.9	100.0	0.56	1.26	1.57	0.31	
59	-588	-437	151	-517	-518	-1	26.2	28.0	1.07	1.68	1.71	0.03	
9	-497	17	514	-89	-455	-366	10.8	32.5	3.01	0.97	1.63	0.66	
56	-665	-913	-248	-730	-999	-269	24.7	19.2	0.78	1.29	1.41	0.12	
36	-661	143	804	-717	-331	386	32.8	24.6	0.75	0.93	1.46	0.53	
41	-451	-536	-86	-341	-739	-398	34.9	28.7	0.82	1.12	1.33	0.22	
23	148	-779	-927	-668	-889	-221	100.1	10.9	0.11	1.78	1.26	-0.53	
35	-421	-201	220	-752	-788	-36	31.5	28.2	0.89	1.36	1.53	0.17	
32	-859	-694	165	-958	-236	721	76.0	53.9	0.71	1.08	1.24	0.16	
33	414	145	-269	427	-80	-507	15.1	4.7	0.31	1.70	1.55	-0.15	
25	-137	330	468	-1024	-263	761	71.2	131.5	1.85	1.32	1.66	0.34	
67	-588	-224	363	-630	-39	592	23.8	16.1	0.68	1.46	1.54	0.09	TRAF3 (both TP)
30	33	-254	-287	-442	-714	-272	39.3	20.3	0.52	1.70	1.48	-0.21	CYLD (both TP)
29	266	151	-116	57	-549	-605	153.6	76.5	0.50	1.64	1.56	-0.09	
