# Supplementary material for: Clonal evolution after treatment pressure in multiple myeloma: heterogenous genomic aberrations and transcriptomic convergence
Source: Leukemia. 2022 May 28;36(7):1887–97. doi: 10.1038/s41375-022-01597-y (PMC9252918; doi:10.1038/s41375-022-01597-y)
Supplement: Supplementary file 16 — Table S10 [file 41375_2022_1597_MOESM16_ESM.pdf]

Table S10. Table showing the Proliferative Index for the earliest and latest sample, and evolution patterns.

Patient ID	Ig-T/HRD	PI Sample 1	PI latest S*	PI Change[#]	Evolution pattern[$]	Clonal change	Dead	TTD
52	HRD	1.4	4.4	**3.06**	Differental	Y	n	>27
65	HRD	1.0	3.9	**2.93**	Differental	Y	**y**	**0**
63	t(8;14)	1.9	4.7	**2.79**	Differental	Y	**y**	**3**
60	HRD	1.5	4.2	**2.65**	Differental	Y	**y**	**0**
28	HRD	1.9	4.5	**2.61**	Differental	Y	y	19
27	t(4;14)	1.5	4.0	**2.50**	Linear	Y	**y**	**1**
46	unknown	1.9	4.3	**2.47**	Differental	Y	**y**	**3**
20	t(11;14)	1.9	4.2	**2.32**	Differental	Y	**y**	**2**
51	HRD	1.1	3.2	**2.09**	Differental	Y	**y**	**1**
31	t(4;14)	1.1	3.1	**2.05**	Differental	Y	**y**	**1**
49	HRD	1.6	3.6	**2.00**	Differental	Y	**y**	**0**
54	t(6;14)	1.8	3.4	**1.53**	Differental	Y	n	>13
53	HRD	0.6	2.1	**1.52**	Differental	Y	n	>25
55	HRD	0.8	2.3	**1.48**	Differental	Y	n	>41
50	t(11;14)	1.5	2.9	**1.45**	Differental	Y	n	>29
58	HRD	2.5	3.9	**1.41**	Differental	Y	**y**	**4**
18	HRD	2.3	3.7	**1.35**	Differental	Y	**y**	**0**
10	t(4;14)	1.8	3.0	**1.21**	Stable	N	y	19
24	t(11;14)	0.3	1.4	**1.07**	Differental	Y	**y**	**10**
15	HRD	1.4	2.3	**0.90**	Differental	Y	y	46
26	HRD	2.5	3.3	**0.78**	Differental	Y	**y**	**1**
34	HRD	2.5	3.3	**0.75**	Differental	Y	**y**	**2**
32	HRD	0.2	0.9	**0.69**	Differental	Y	**y**	**1**
42	HRD	1.3	1.9	**0.65**	Differental	Y	y	38
23	HRD	0.5	1.1	**0.52**	Differental	Y	y	14
8	t(11;14)	1.3	1.8	**0.49**	Linear	N	**y**	**4**
25	HRD	0.3	0.8	**0.49**	Differential*	N	y	27
47	t(4;14)	1.8	2.2	**0.42**	Differental	N	y	31
61	t(11;14)	2.9	3.2	0.34	Linear	Y	y	21
59	t(4;14)	1.0	1.3	0.31	Stable	N	n	>43
4	t(11;14)	1.7	1.9	0.28	Differental	Y	**y**	**6**
9	t(11;14)	1.0	1.2	0.25	Linear	Y	n	>46
43	t(4;14)	1.1	1.3	0.24	Differental	Y	y	21
48	HRD	1.7	1.8	0.18	Differental	Y	**y**	**5**
30	HRD	0.5	0.6	0.15	Stable	N	n	>47
45	t(11;14)	1.3	1.4	0.10	Stable	N	n	>7
29	HRD	0.5	0.5	-0.01	Stable	N	y	20
33	t(11;14)	0.9	0.9	-0.06	Differental	Y	y	22
35	HRD	1.0	0.9	-0.07	Differental	Y	y	26
40	t(11;14)	1.7	1.7	-0.07	Linear	Y	n	>18
66	HRD	2.2	1.9	-0.29	Stable	N	y	19
36	HRD	1.5	1.1	-0.38	Linear	Y	y	48
67	HRD	1.2	0.8	**-0.42**	Stable	Y	n	>32
44	t(11;14)	2.3	1.8	**-0.48**	Linear	N	n	22
57	t(12;14)	2.2	1.7	**-0.53**	Linear	Y	n	>36
41	t(4;14)	1.6	1.1	**-0.57**	Differental	Y	y	29
62	t(4;14)	2.1	1.5	**-0.59**	Linear	Y	**y**	**6**
56	t(11;14)	2.5	1.1	**-1.33**	Stable	N	n	>36
64	t(4;14)	3.6	1.7	**-1.95**	Differental	Y	**y**	**10**

PI: Proliferative index, measured using a gene expression signature (Zhan et al.,Blood, 2006).
*Latest available progression sample
[#] change in PI (Proliferative Index; see methods) from first to last sample. Bold=change >0.4
[$]If many samples for each patient, the evolution pattern showing the most change is shown (differential>linear>stable). *Linear evolution at the tp where transcription increase occur, see table S5.
Cytogenetic subgroup (Suplementary Table 12)
Differential = differential clonal response
Linear = linear evolution
Stable = stable evolution
TTD = Time to death or to last control (patients still alive) from latest sample. Bold: Patients with a end-stage disease sample (<12 months from death)
