# Supplementary material for: Clonal evolution after treatment pressure in multiple myeloma: heterogenous genomic aberrations and transcriptomic convergence
Source: Leukemia. 2022 May 28;36(7):1887–97. doi: 10.1038/s41375-022-01597-y (PMC9252918; doi:10.1038/s41375-022-01597-y)
Supplement: Supplementary file 17 — Table S11 [file 41375_2022_1597_MOESM17_ESM.pdf]

Table S11. Mutations in ABCB transporters, nuclear exportins, relevant treatment targets including CD38, BCL2 and SLAMF7 (Figure 5), CGA genes and inhibitory and stimulatory receptors (Figure 6A).
RNA expression are shown for samples with genomic mutation present, when available. NA: no data, CCF: Cancer Cell Fraction

Patient_ID	Gene	Gene Class	Chr	ExonicFunc.refGene	S1 CCF	RNA depth*	RNA alt freq	S2 CCF	RNA depth	RNA alt freq	S3 CCF	RNA depth	RNA alt freq	S4 CCV	RNA depth	RNA alt freq S4
36	XPO4	Nuclear exportin	chr13	nonsynonymous SNV	0.0123101	8	0.559	0.409194	4	0						
34	ABCA1	ABC transporter	chr9	nonsynonymous SNV	0.749639	5	0.6	0	15	0	0	15	0	0		
34	ABCA5	ABC transporter	chr17	nonsynonymous SNV	0			0			0.26834	0	0	0		
34	ABCG1	ABC transporter	chr21	nonsynonymous SNV	0			0			0.252859	7	0	0		
35	ABCA1	ABC transporter	chr9	nonsynonymous SNV	0			0.523729	0	0						
35	ABCC1	ABC transporter	chr16	nonsynonymous SNV	1.08084	3	0.091	0.908798	0	0						
4	CD38	Drug target	chr4	nonsynonymous SNV	0	266	0	0.881623	1370	0.416						
5	CFTR	ABC transporter	chr7	nonsynonymous SNV	0.0275494	*NA*	*NA*	0.841492	*NA*	*NA*						
9	ABCC9	ABC transporter	chr12	nonsynonymous SNV	1.02923	0	0	0.772415	1	0						
9	ABCF3	ABC transporter	chr3	nonsynonymous SNV	0.0524541	23	0	0.648549	16	0						
24	ABCG1	ABC transporter	chr21	nonsynonymous SNV	0			0.448335	3	0						
24	CSE1L	ABC transporter	chr20	nonsynonymous SNV	0.0580441	8	0	0.787664	20	0.443						
26	CFTR	ABC transporter	chr7	nonsynonymous SNV	1.27114	0	0	1.08694	0	0	1.08656	0	0			
27	ABCA2	ABC transporter	chr9	nonsynonymous SNV	0.202985	1	0	0			0					
28	ABCA1	ABC transporter	chr9	nonsynonymous SNV	0.00822707	2	0	0.81095	0	0						
28	XPO1	Nuclear exportin	chr2	nonsynonymous SNV	0.300398	67	0.102	0	0	0						
31	ABCC9	ABC transporter	chr12	nonsynonymous SNV	1.24884	0	0	0.921358	1	1	0.946819	1	0			
31	ABCC9	ABC transporter	chr12	nonsynonymous SNV	1.13506	0	0	0.954433	1	1	1.14146	0	0			
31	ABCG2	ABC transporter	chr4	nonsynonymous SNV	0			0.132193	0	0	0	2	0			
41	ABCG2	ABC transporter	chr4	stopgain	0.36134	0	0	0								
43	ABCA5	ABC transporter	chr17	nonsynonymous SNV	1.21217	22	0.32	0.809013	0	0	1.09972	22	0.5			
43	ABCC9	ABC transporter	chr12	nonsynonymous SNV	1.05707	0	0	0.895412	0	0	0.940018	0	0			
47	ABCG1	ABC transporter	chr21	nonsynonymous SNV	0.892131	*NA*	*NA*	0.924271	*NA*	*NA*						
53	ABCB8	ABC transporter	chr7	nonsynonymous SNV	0.885919	109	0.34	0.917726	125	0.3						
57	ABCA1	ABC transporter	chr9	nonsynonymous SNV	1.05424	49	0.47	1.02484	30	0.47						
57	ABCG2	ABC transporter	chr4	nonsynonymous SNV	1.01725	126	0.52	1.07101	117	0.46						
58	XPOT	Nuclear exportin	chr12	nonsynonymous SNV	0.735421	315	0.37	0.583223	*NA*	*NA*	1.04349	787	0.55			
61	MCL1	Drug target	chr1	nonsynonymous SNV	0.829188	1039	0.26	0.904074	2482	0.29						
61	XPO6	Nuclear exportin	chr16	stopgain	0.665247	23	0.09	1.19122	503	0.14						
58	MORC1	CGA	chr3	nonsynonymous SNV	0			0			0.277968	0	0			
61	MORC1	CGA	chr3	nonsynonymous SNV	0.842231	0	0	0.994493	13	0.38						
44	WDR64	CGA	chr1	nonsynonymous SNV	0			0.459131	57	0.18						
28	CD274	Inhibitory	chr9	nonsynonymous SNV	1.03467	14	0.5	0.994641	52	0.71						
36	CTCFL	CGA	chr20	nonsynonymous SNV	0.085256	0	0	0								
10	MORC1	CGA	chr3	nonsynonymous SNV	0.170751	0	0	0.0192873	0	0	0.0474536	0	0			
9	TNFSF9	Stimulatory	chr19	nonsynonymous SNV	1.07927	0	0	1.22824	4	0						
36	TNFSF9	Stimulatory	chr19	nonsynonymous SNV	0.226112	1	0	0.091955	0	0						
39	WDR64	CGA	chr1	nonsynonymous SNV	0			1.19068	*NA*	*NA*						

*For estimates of RNA depth and RNA alt frequency; for in-house cohort; see methods. For CoMMpass samples, data were from the file "MMRF_CoMMpass_IA13a_IGV_All_Canonical_Variants.mut".
