# Supplementary material for: Clonal evolution after treatment pressure in multiple myeloma: heterogenous genomic aberrations and transcriptomic convergence
Source: Leukemia. 2022 May 28;36(7):1887–97. doi: 10.1038/s41375-022-01597-y (PMC9252918; doi:10.1038/s41375-022-01597-y)
Supplement: Supplementary file 18 — Table S12 [file 41375_2022_1597_MOESM18_ESM.pdf]

Table S12. Comparison of Translocation subgroup with detected primary translocations from a) FISH data for own-institutional samples, and b) long-insert WGS for CoMMpass samples. Nd: not detected. NA: no data

A)

Patient_ID	RNAseq-Ig-T	Exome CN	FISH data	Ig-T/HRD	Comment
3	*NA*	*NA*	del17	*NA*	
4	CCND1	nd	t(11;14)	t(11;14)	
5	*NA*	*NA*	nd	*NA*	
7	*NA*	*NA*	nd	*NA*	
8	CCND1	nd	nd	t(11;14)	
9	CCND1	nd	nd*	t(11;14)	* = FISH: tested for t(4;14) only.
10	WHSC1	nd	t(4;14)	t(4;14)	
13	*NA*	*NA*	nd	*NA*	
14	*NA*	*NA*	nd	*NA*	
15	nd	HRD	t(4;14)	HRD	RNAseq: No expression of WHSC1 or FGFR3 (TPM<3)
17	CCND1	nd	nd	t(11;14)	
18	nd	HRD	nd	HRD	
19	*NA*	*NA*	nd	*NA*	
20	CCND1	nd	t(11;14)	t(11;14)	
23	nd	HRD	nd	HRD	
24	CCND1	nd	t(11:14)	t(11;14)	
25	nd	HRD	nd	HRD	
26	nd	HRD	nd	HRD	
27	WHSC1	nd	t(4;14)	t(4;14)	
28	nd	HRD	nd	HRD	
29	nd	HRD	nd	HRD	
30	nd	HRD	nd	HRD	
31	WHSC1	nd	t(4;14)	t(4;14)	
32	nd	HRD	nd	HRD	
33	CCND1	nd	t(11;14)	t(11;14)	
34	nd	HRD	nd	HRD	
35	nd	HRD	nd	HRD	
36	nd	HRD	nd	HRD	
39	*NA*	*NA*	t(4;14)	t(4;14)	

RNAseq-Ig-T: Estimates of Canonical Translocations by expression on RNAseq

Ig-T/HRD: cytogenetic subgroup, based on RNAseq-Ig-T data and CNV data (from WES) (HRD).

B)

Patient_ID	FISHseq	RNAseq-Ig-T	Exome-FISH_CN	Ig-T/HRD
40	CCND1	CCND1	nd	t(11;14)
41	WHSC1	WHSC1	nd	t(4;14)
42	nd	nd	HRD	HRD
43	WHSC1	WHSC1	nd	t(4;14)
44	CCND1	CCND1	nd	t(11;14)
45	CCND1	CCND1	nd	t(11;14)
46	*NA*	*NA*	*unknown*	*NA*
47	WHSC1	WHSC1	nd	t(4;14)
48	nd	nd	HRD	HRD
49	nd	nd	HRD	HRD
50	CCND1	CCND1	nd	t(11;14)
51	nd	nd	HRD	HRD
52	nd	nd	HRD	HRD
53	nd	nd	HRD	HRD
54	CCND3	CCND3	nd	t(6;14)
55	nd	nd	HRD	HRD
56	CCND1	CCND1	HRD	t(11;14)
57	CCND2	CCND2	nd	t(12;14)
58	nd	nd	HRD	HRD
59	WHSC1	WHSC1	nd	t(4;14)
60	MAFB	nd	HRD	HRD
61	CCND1	CCND1	nd	t(11;14)
62	WHSC1	WHSC1	HRD	t(4;14)
63	MAFA	MAFA	nd	t(8;14)
64	WHSC1	WHSC1	nd	t(4;14)
65	nd	nd	HRD	HRD
66	nd	nd	HRD	HRD
67	nd	nd	HRD	HRD

FISHseq: Canonical Translocations identified by long-insert WGS.

RNAseq-Ig-T: Estimates of Canonical Translocations by expression on RNAseq

Ig-T/HRD: cytogenetic subgroup

If both a HRD Call and a canonical translocation found, translocation is chosen for the cytogenetic group.
